# The dietary ligands, omega-3 fatty acid endocannabinoids and short-chain fatty acids prevent cytokine-induced reduction of human hippocampal neurogenesis and alter the expression of genes involved in neuroinflammation and neuroplasticity

**DOI:** 10.1038/s41380-025-03119-5

**Published:** 2025-07-16

**Authors:** Gargi Mandal, Silvia Alboni, Nadia Cattane, Moira Marizzoni, Samantha Saleri, Nikita Arslanovski, Nicole Mariani, Madeline Kirkpatrick, Annamaria Cattaneo, Carmine M. Pariante, Alessandra Borsini

**Affiliations:** 1 Section of Stress, Psychiatry and Immunology Laboratory, Institute of Psychiatry, Psychology and Neuroscience, Department of Psychological Medicine, King’s College London, London, UK; 2 Department of Life Sciences, University of Modena and Reggio Emilia, Modena, Italy; 3 Biological Psychiatry Laboratory, IRCCS Fatebenefratelli, Brescia, Italy; 4 Department of Behavioural Science and Health, University College London, London, UK

**Keywords:** Molecular biology, Cell biology, Stem cells, Neuroscience

## Abstract

The dietary ligands, omega-3 fatty acid endocannabinoids (eCBs) eicosapentaenoyl ethanolamide (EPEA) and docosahexaenoyl ethanolamide (DHEA), and short-chain fatty acids (SCFAs) acetate, propionate and butyrate, have anti-inflammatory and antidepressant properties. However, the molecular mechanisms underlying their action in the human brain remain elusive. Here, we treated human hippocampal neurons (HPC0A07/03 C) with eCBs (EPEA (300 pM) or DHEA (700 pM)), or SCFAs (acetate (200 uM), propionate (30 uM), butyrate (20 uM)), followed by interleukin (IL)1β (10,000 pg/ml) or IL6 (50 pg/ml). We found that treatment with either eCBs or SCFAs prevented IL1β- and IL6-induced reduction in neurogenesis and increase in apoptosis. These effects were mediated by IL1β-induced production of IL6, interferon-gamma (IFNγ) and tumour necrosis factor-alpha (TNFα), and by IL6-induced IL1β, IL8 and IL13, all of which were prevented by treatment with eCBs. In contrast, IL1β-induced production of IL6, IL12 and fractalkine (CX3CL1), and IL6-induced production of CX3CL1, were prevented by SCFAs. Treatment with IL1β and IL6 also increased the production of candidate kynurenine pathway metabolites, such as kynurenine (KYN) and nicotinic acid (NICA), which again were prevented by eCBs and SCFAs. We then conducted mRNA sequencing analysis to investigate cellular genes and signalling pathways relevant for the neuro-inflammatory changes previously observed, and putatively prevented by eCB and SCFA treatment. We found that IL1β decreased the expression of the neuroplasticity gene, FRY microtubule binding gene (*FRY)*, and increased the expression of the neuroinflammation gene, U3 small nucleolar ribonucleoprotein homolog C subunit processome component (*UTP14C)*, and both these effects were prevented by either acetate or propionate. Similarly, the expression of the proinflammatory gene, ADAM metallopeptidase with thrombospondin type 1 motif 1 (*ADAMTS1*), was increased by IL6, an effect that was prevented by either EPEA or acetate. Altogether, we identify novel anti-inflammatory and neurogenic mechanisms mediating the effect of eCBs and SCFAs on human hippocampal neurogenesis, which can be significant as potential future treatment candidates in the context of neuropsychiatric disorders.

## Introduction

There is mounting evidence indicating chronic, low-grade inflammation in major depressive disorder (MDD) [1]. Moreover, 30% of individuals with MDD have increased inflammation and poor response to antidepressants [2], and due to the heterogeneity of depression, no type of treatment is universally efficacious for all individuals [3]. This necessitates the identification of novel approaches that target biological and environmental factors which are safe for everyday use [4]. Dietary ligands, such as omega-3 fatty acid endocannabinoids (eCBs) (eicosapentaenoyl ethanolamide (EPEA) and docosahexaenoyl ethanolamide (DHEA)) and short-chain fatty acids (SCFAs), are considered promising strategies [5–7] which impact on inflammatory mechanisms.

Dietary intake of omega-3 fatty acids (eicosapentaenoic acid (EPA) and docosahexaenoic acid (DHA)), the precursors of eCBs, have been shown to provide beneficial antidepressant effects in clinical studies [6, 8]. Moreover, we have demonstrated that in vitro treatment of human hippocampal neurons with EPA and DHA, like with antidepressants, can prevent reduction in neurogenesis caused by proinflammatory cytokines [9]. The derivatisation of the carboxylic acid end of EPA and DHA with ethanolamine results in the formation of two principal omega-3 eCBs, EPEA and DHEA, respectively, which have potent anti-inflammatory and synaptogenic properties [10]. In a double-blind, randomised controlled trial consisting of EPA, DHA supplementation to patients with MDD, an increase in plasma levels of EPEA was positively associated with rates of clinical remission of depression [11]. Moreover, EPEA and DHEA reduce IL6 levels and monocyte chemoattractant protein-1 (MCP-1) in lipopolysaccharide (LPS)-stimulated adipocytes [12], and DHEA promotes synaptogenesis and neurite growth in rodent cortical neurons, being 50-100-fold more effective than DHA [13]. However, most previous research on the eCBs were conducted in non-neuronal cell lines or in rodent models, thus warranting further investigation in human brain cells, especially in the context of hippocampal neurogenesis.

On the other hand, SCFAs, like acetate, propionate, and butyrate, are the primary metabolites produced by the synthesis of dietary fibre in the gut microbiota, and they have been shown to be reduced in MDD patients [5, 14]. SCFAs, such as butyrate, are also enriched in dairy products [15]. Systemic circulation of acetate, propionate and butyrate from the gut lumen to the brain plays a key role in regulating the blood-brain barrier and neuro-immuno-endocrine functions [16, 17]. Indeed, a recent in vivo study demonstrated that acetate supplementation significantly improves depression-like behaviour in mice that were subjected to chronic social failure stress [18]. Additionally, repeated treatment with sodium butyrate reduces LPS-induced depressive-like behaviours through regulating hippocampal microglial activation [19]. However, similar to the eCBs, most of prior research in this area has been conducted in animal models, and the extent to which SCFAs influence hippocampal neurogenesis in human brain cells is still not fully understood.

Mechanistically, the antidepressant action of both eCBs and SCFAs could stem from their anti-inflammatory activity, therefore being potentially beneficial to at least a sub-group of patients with depression characterised by low-grade inflammation [6, 20, 21]. Immune activation in these sub-groups of individuals is characterised by increased production of inflammatory cytokines, including IL1β and IL6, both in the periphery and in the cerebrospinal fluid [21–23]. Increases in circulating proinflammatory cytokines can subsequently dysregulate blood-brain barrier integrity, alter signalling of serotonin and glutamate, which are pertinent for depressive symptoms, and activate the kynurenine pathway [24, 25]. Furthermore, inflammation results in the suppression hippocampal neurogenesis and the proliferation and survival of new neurons in the dentate gyrus [26, 27], and reduced neurogenesis is associated with decreased antidepressant efficacy [28]. Previously, using a validated human hippocampal neuronal cell model, we have demonstrated the effect of IL1β, IL6 and interferon-alpha (IFNα) on reducing cell proliferation and neurogenesis and increasing apoptosis via activation of the downstream inflammatory signalling pathways [9, 29–33] and the production of neurotoxic kynurenine metabolites [9]. However, it is unknown whether eCBs and SCFAs can modulate these pathways in hippocampal neurons in the presence of an inflammatory challenge.

Considering the limited evidence for the role of these dietary ligands in the brain, and especially in the context of inflammation-induced depression, we used our aforementioned in vitro model which consists of exposing immortalised human hippocampal cell line HPC0A07/03 C to candidate ‘depressogenic’ cytokines, namely IL1β and IL6 [6, 9, 29, 33, 34], with or without pre-treatment with eCBs or SCFAs. We subsequently measured downstream candidate mechanisms, including cytokines and kynurenine pathway metabolites, and conducted gene expression analysis, with a particular focus on neuroinflammatory and neuroplasticity genes.

## Methods

### Cell culture

Multipotent human hippocampal progenitor cell line HPC0A07/03C (provided by ReNeuron, Surrey, UK) was used. previously validated using a hippocampal newborn neuron specific marker, Prospero homeobox protein 1 (Prox1) [35]. Cells were allowed to proliferate in reduced modified media (for details on media reagents see Supplementary Materials) with the growth factors epidermal growth factor (EGF), basic fibroblast growth factor (bFGF) and 4 hydroxytamoxifen (4-OHT). To initiate differentiation, growth factors and 4-OHT were removed. The Supplementary Materials contains additional information on the cells.

### Cell assay

**Fig. 1:**
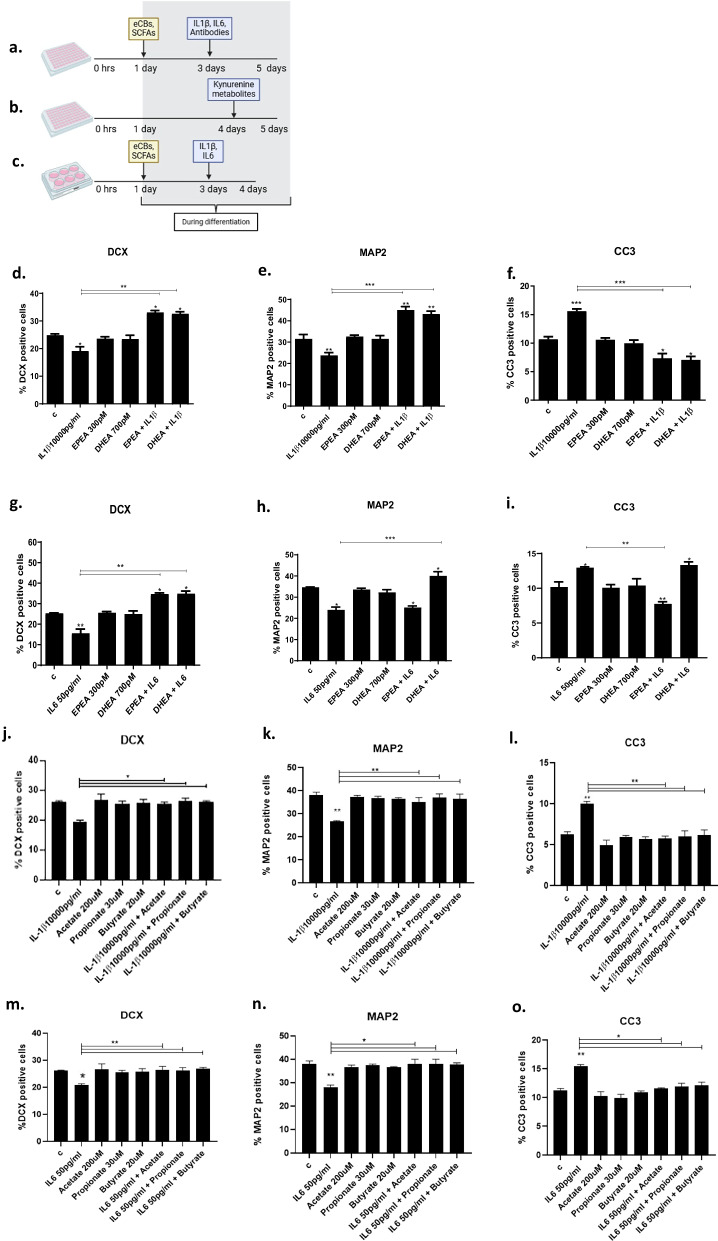
Timeline of the cellular assays performed and measurement of cellular markers of neurogenesis and apoptosis after the treatment of hippocampal cells with eCBs and SCFAs. **a**. HPC0A07/03C cells were plated at a density of 15,000 per well in a 96-well plate for 24 h and then treated with EPEA (300 pM), DHEA (700 pM), acetate (200 uM), propionate (30 uM) and butyrate (20 uM) for 2days, after which the compounds are removed, and the cells are treated with IL1β (10,000 pg/ml) and IL6 (50 pg/ml) for additional 2 days. In a different set of experiments, cells were treated with IL1β (10,000 pg/ml) and IL6 (50 pg/ml) and antibodies against specific cytokines (IL6A (0.1 ug/ml), IFNγA (0.06 ug/ml), TNFαA (0.01 ug/ml), IL1βA (0.1 ug/ml), IL8A (0.1 ug/ml), IL13A (0.1 ug/ml), CX3CL1A (1 ug/ml) and IL12A (0.3 ug/ml)) for 2 days. Subsequently, the cell supernatant was collected to measure the cytokine levels and the kynurenine pathway metabolites, and the cells were fixed with 4% PFA. **b**. In another set of experiments, which were conducted in a similar manner, the cells were plated in a 96-well plate for 24 h. This was followed by a 3-day treatment with just media, after which the cells were treated with kynurenine pathway metabolites (KYN (0.15 uM, 1.2 uM), NICA (0.2 uM, 1.3 uM)) for 1 day. The cells were then fixed, and the cell supernatant was collected. **c**. Cells were plated at a density of 300,000 cells per well in a 6-well plate for 24 h, then pre-treated with eCBs and SCFAs for 2 days, followed by treatment with cytokine for an additional 24 h. The cell lysates were then collected and the RNA extracted for RNA sequencing and PCR analyses. **d**–**o**. Pre-treatment of cells with EPEA (300 pM), DHEA (700 pM), acetate (200 uM), propionate (30 uM) and butyrate (20 uM) followed by IL1β, IL6 prevented the reduction in neurogenesis (DCX + and Map2 + cells) and/or increase in apoptosis (CC3 + cells) induced by the cytokines alone. Two-way ANOVA with Bonferroni’s post hoc test was performed. Data are shown as mean ± SEM; *p < 0.05, **p < 0.01, ***p < 0.001, compared with vehicle treatment or as indicated.

Similar to previous experiments [6], cells were plated on 96 well plates (Nunclon) at a density of 1.5 × 10^4^ cells per well. After 1 day proliferation, cells were left to differentiate for a total of 4 days. Differentiated cells were pre-treated with either eCBs (EPEA (300 pM), DHEA (700 pM)) or SCFAs (acetate (200 μM), propionate (30 μM), butyrate (20 μM)) for 2 days, followed by treatment with either IL1β (10,000 pg/ml) or IL6 (50 pg/ml), with or without candidate cytokine antibodies, IL6 Antibody (A) (0.1 ug/ml), interferon gamma (IFNγ)A (0.06 ug/ml), tumor necrosis factor alpha (TNFα) A (0.01 ug/ml), IL1βA (0.1 ug/ml), IL8A (0.1 ug/ml), IL13A (0.1 ug/ml), fractalkine (CX3CL1) A (1 ug/ml), and IL12A (0.3 ug/ml)) for additional 2 days to conduct neurogenesis, immunoassays and kynurenine pathway analyses (Fig. 1a). The concentration of eCBs and SCFAs was identified from previous studies [11, 35]. Cells were also treated with candidate kynurenine pathway metabolites (kynurenine (KYN) (1.2 uM), nicotinic acid (NICA) (1.3 uM, 0.2 uM) on day 4 during differentiation (Fig. 1b). For RNA sequencing analysis, cells were plated in 6-well plates (Nunclon) at a density of 30 × 10^4^ cells per well for 1day in proliferation media, then pre-treated with eCBs and SCFAs in differentiation media at the aforementioned concentration for 2 days, and subsequently treated with cytokines for additional 1 day (Fig. 1c). Cells were washed with warm PBS and fixed with 4% PFA for 20 min at room temperature, and supernatant was collected for subsequent measurement of cytokines and kynurenine pathway metabolites.

### Immunocytochemistry and quantification of immunofluorescence

Fixed cells were stained for markers of immature and mature neurons using doublecortin (DCX, rabbit anti-DCX, 1:500, Abcam) and microtubule-associated protein 2 (MAP2, mouse anti-MAP2 [HM], 1:500), whereas apoptotic cells were examined using cleaved caspase 3 (CC3; rabbit anti-CC3, 1:500, Abcam). Secondary antibodies (Alexa 488 donkey anti-rabbit; 1:1000, and Alexa 555 donkey anti-mouse, 1:1000, Invitrogen) were used the next day and all cells were labelled using DAPI dye, as in previous publication (see also Supplementary Materials) [6]. The number of DCX, MAP2, and CC3 + cells over total DAPI + cells was counted using an insight automated imaging platform (CellInsight) (see Supplementary Fig. 1 for representative images).

### Multiplex cytokine assay

The concentration of 10 candidate cytokines, IL1β, IL2, IL4, IL6, IL8, IL10, IL12, IL13, IFNγ, TNFα, were measured in the supernatant using the V-PLEX assay from Meso Scale Discover (MSD) (Gaithersburg, MD). The chemokine CX3CL1 was measured using U-PLEX assay, again from MSD. The SPECTOR imaging machine was used to detect fluorescent signal, according to the manufacturer’s instructions. For additional information, see also Supplementary Materials.

### Liquid chromatography

The analysis of tryptophan (TRP), KYN, anthranilic acid (ANA), kynurenic acid (KYNA), 3-hydroxykynurenine (3-HK), 3-hydroxyanthranilic acid (3-HANA), NICA, quinolinic acid (QUIN), nicotinamide (NIC), in the supernatant was performed using an Agilent HP 1200 liquid chromatograph (Agilent, Milan, Italy) consisting of a binary pump, an autosampler and a thermostated column compartment. Separation was performed with a Discovery® HS-F5 column (150 × 2.1 mm, 3 μm, Supelco, Milan, Italy). Detection was performed using an Agilent 6410 triple quadrupole-mass spectrometer with an electrospray ion (ESI) source operated in the positive ion mode. Extended details can be found in the Supplementary Materials and in previous publications [9]. Of all the metabolites measured, only TRP, KYN, NICA and NIC were above detection limit.

### RNA isolation, cDNA synthesis and quantitative real-time PCR (qPCR)

RNA was extracted by using the RNeasy Micro Kit (Qiagen, Crawley, UK) following manufacturer’s instructions. RNA quality and quantity were assessed by evaluation of the A260/280 and A260/230 ratios using a Nanodrop spectrometer (Nanodrop Technologies, Wilmington, DE, USA). For cDNA synthesis, 1 μg of RNA was reverse transcribed using Superscript III enzyme (Invitrogen, Carslbad, CA, USA), as previously described [36]. Subsequently, both target and housekeeping gene expression levels were analysed by TaqMan RT-PCR instrument (CFX384 real time system, Bio-Rad) using the iScript one-step RTPCR kit for probes (Bio-Rad). For additional information, see also Supplementary Materials.

### Gene expression and pathway analysis

Transcriptome library preparation was performed by using the Illumina Stranded mRNA Prep Ligation kit. Libraries were sequenced on a NextSeq 2000 Illumina platform. The quality of the raw data was checked by using FastQ and the raw read counts were quantified at the transcript level using Salmon (v 1.4.0). Next, the transcript-level differential expression was assessed using DESeq2 (v1.30.1) in RStudio 4.2. Differentially expressed genes (DEGs) were identified by using an adjusted *p*-value ≤ 0.1 and a log_2_FC ± 0.59 (FC ± 1.5) as threshold, meaning a 50% modulation in the gene expression levels. We used the obtained lists of DEGs to perform pathway analyses by using Ingenuity Pathway Analysis (IPA) (Qiagen). Additionally, Venn diagram for gene expression analysis were made using Venny 2.1 [37]. For additional information, see also Supplementary Materials.

### Statistical analysis

Statistical analyses were performed with GraphPad Prism version 8 for the cellular assays and consisted of one-way/two-way analysis of variance followed by Bonferroni’s *post hoc* analyses where appropriate. Variance was similar between the groups that have been statistically compared. Data are presented as mean ± SEM, and *p* values  ≤ 0.05 were considered significant.

## Results

### EPEA and DHEA prevented IL1β- and IL6-induced reduction in neurogenesis and increase in apoptosis

As we previously demonstrated [6], treatment of cells with IL1β (10,000 pg/ml) for 2 days during differentiation resulted in a decrease in the number of DCX + (−5%, *p* < 0.01, vs vehicle, Fig. 1d) and MAP2 + (−8%, *p* < 0.01, vs vehicle, Fig. 1e) cells, and an increase in the number of CC3 + cells (+5%, *p* < 0.01, vs vehicle, Fig. 1f). Moreover, pre-treatment of cells with either EPEA (300 pM) or DHEA (700 pM) prevented IL1β-induced decrease in DCX + and MAP2 + (Fig. 1d, e) and increase in CC3 + cells (Fig. 1f).

In accordance with our previous findings [6], IL6 also negatively affected neurogenesis and increased apoptosis (50 pg/mL) (DCX: −10%, *p* < 0.05, vs vehicle, Fig. 1g; MAP2: −9%, *p* < 0.01, vs vehicle, Fig. 1h; CC3: +2%, *p* < 0.01, vs vehicle, Fig. 1i). Similarly, EPEA and DHEA were able to prevent IL6-induced decrease on DCX + . However, DHEA only was able to prevent the decrease in MAP2 + cells, and EPEA only was able to prevent the increase in CC3 + cells (Fig. 1g–i).

Altogether, these findings show that EPEA and DHEA can equally prevent the detrimental effect of IL1β and IL6 in the context of human hippocampal neurogenesis.

### SCFAs prevented IL1β- and IL6-induced reduction in neurogenesis and increase in apoptosis

Treatment of cells with IL1β (10,000 pg/mL) for 2 days during differentiation resulted in a decrease in the number of DCX + (−7%, *p* < 0.01, vs vehicle, Fig. 1j) and MAP2 + cells (−12%, *p* < 0.01, vs vehicle, Fig. 1k), and an increase in CC3 + cells (+4%, *p* < 0.01, vs vehicle, Fig. 1l). Moreover, pre-treatment of cells with either acetate (200 μM), propionate (30 μM) or butyrate (20 μM) prevented IL1β-induced decease in DCX + and MAP2 + (Fig. 1j, k) and increase in CC3 + cells (Fig. 1l).

Similar effects were observed for IL6 (50 pg/mL) (DCX: −6%, *p* < 0.05, vs vehicle, Fig. 1m; MAP2: −10%, *p* < 0.01, vs vehicle, Fig. 1n; CC3: +4%, *p* < 0.01, vs vehicle, Fig. 1o). Pre-treatment with acetate, propionate, and butyrate were able to prevent IL6-induced decrease in DCX + and MAP2 + cells (Fig. 1m, n), and increase in CC3 + cells (Fig. 1o).

These findings show that all three SCFAs can equally prevent the detrimental effect of IL1β and IL6 in the context of human hippocampal neurogenesis.

### EPEA and DHEA prevent IL1β- and IL6-induced increases in candidate cytokines

**Fig. 2:**
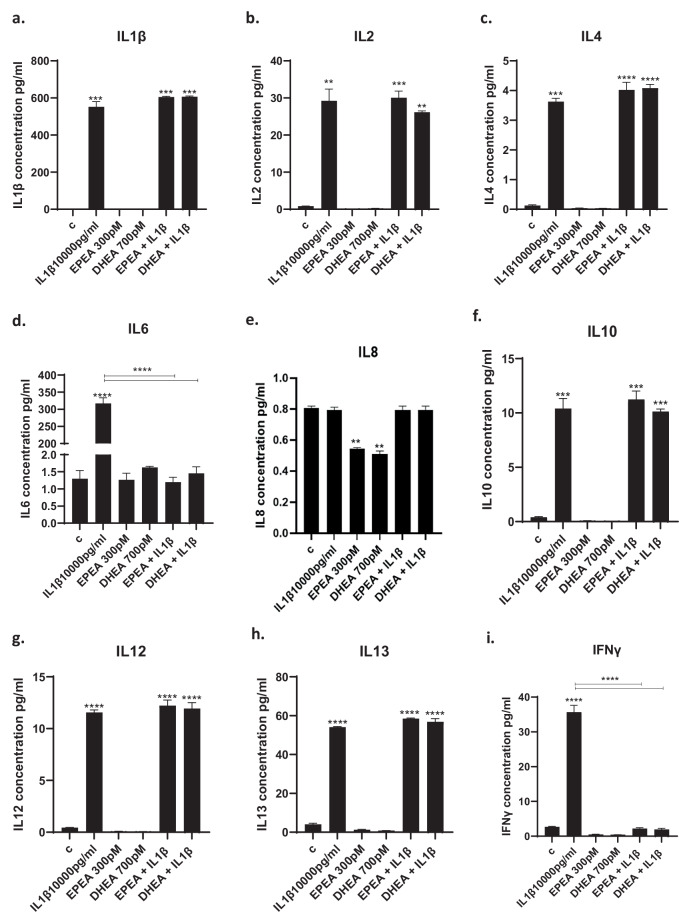

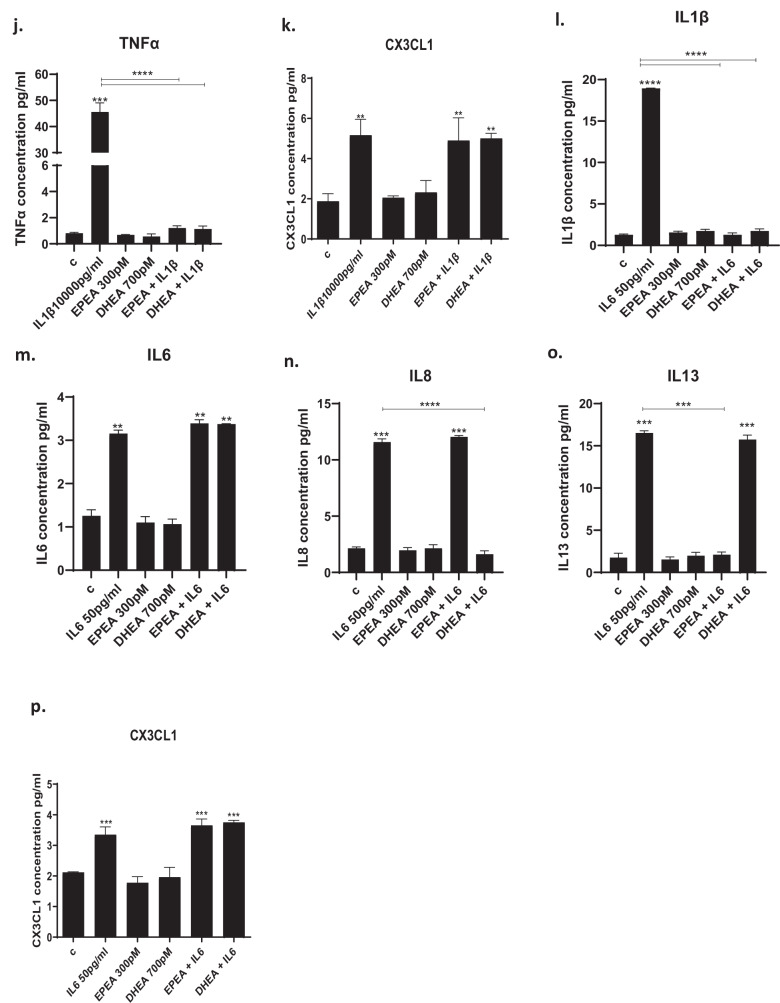
Cytokine levels in the cell supernatant after treatment with eCBs. **a**–**k**. Treatment of cells with IL1β increased the production of IL1β, IL2, IL4, IL6, IL8, IL10, IL12, IL13, IFNγ, TNFα, and CX3CL1, and treatment with EPEA (300 pM) and DHEA (700 pM) prevented the IL1β-mediated increase in IL6, IFNγ, TNFα. **l**–**p**. Treatment of cells with IL6 increased the production of IL1β, IL6, IL8, IL13, and CX3CL1, and treatment with either EPEA or DHEA was able to decrease IL1β levels, treatment with DHEA only decreased IL8 levels, whereas, treatment with EPEA only decreased IL13 levels induced increase of cytokine levels. Two-way ANOVA with Bonferroni’s post hoc test was performed. Data are shown as mean ± SEM; **p < 0.01, ***p < 0.001, ****p < 0.0001, compared with vehicle treatment or as indicated.

We measured levels of candidate cytokines in the supernatant after 4 days of differentiation. IL1β increased the concentration of all candidate cytokines, with the exception of IL8 in the cell supernatant (Fig. 2a–k). Interestingly, pre-treatment with EPEA and DHEA only prevented IL1β-induced increase in IL6 (EPEA: from 317 to 1.2 pg/ml, *p* < 0.01; DHEA: from 317 to 1.8 pg/ml, *p* < 0.01; Fig. 2d), IFNγ (EPEA: from 36 to 2 pg/ml, *p* < 0.01; DHEA: from 36 to 2 pg/ml, *p* < 0.01; Fig. 2i) and TNFα (EPEA: from 45 to 2.7 pg/ml, *p* < 0.01; DHEA: from 45 to 2.3 pg/ml, *p* < 0.01; Fig. 2j), but did not affect the levels of the other cytokines (Figs. 2a–c, e–h, k).

In contract, IL6 increased the concentration of IL1β, IL6, IL8, IL13, and CX3CL1 (Fig. 2l–p). Pre-treatment with EPEA and DHEA prevented IL6-induced increase in IL1β (EPEA: from 19 to 1.3 pg/ml, *p* < 0.01; DHEA: from 19 to 1.7 pg/ml, *p* < 0.01; Fig. 2l), whereas DHEA prevented IL6-induced IL8 (from 11 to 1.2 pg/ml, *p* < 0.01; Fig. 2n), EPEA prevented IL6-induced IL13 (from 16.5 to 2 pg/ml, *p* < 0.01, Fig. 2o). However, neither of the two did affect the levels of the other cytokines (Fig. 2m, p).

Altogether, these findings indicate that EPEA and DHEA modulate different inflammatory cytokines when in the presence of IL1β vs IL6.

### SCFAs prevent IL1β- and IL6-induced increase in candidate cytokines

**Fig. 3:**
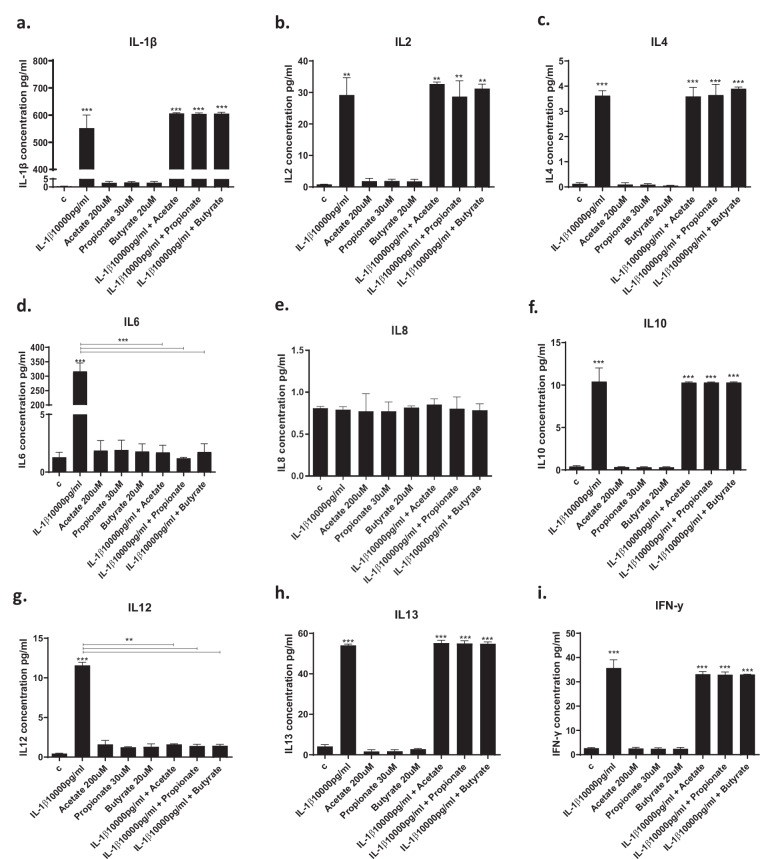

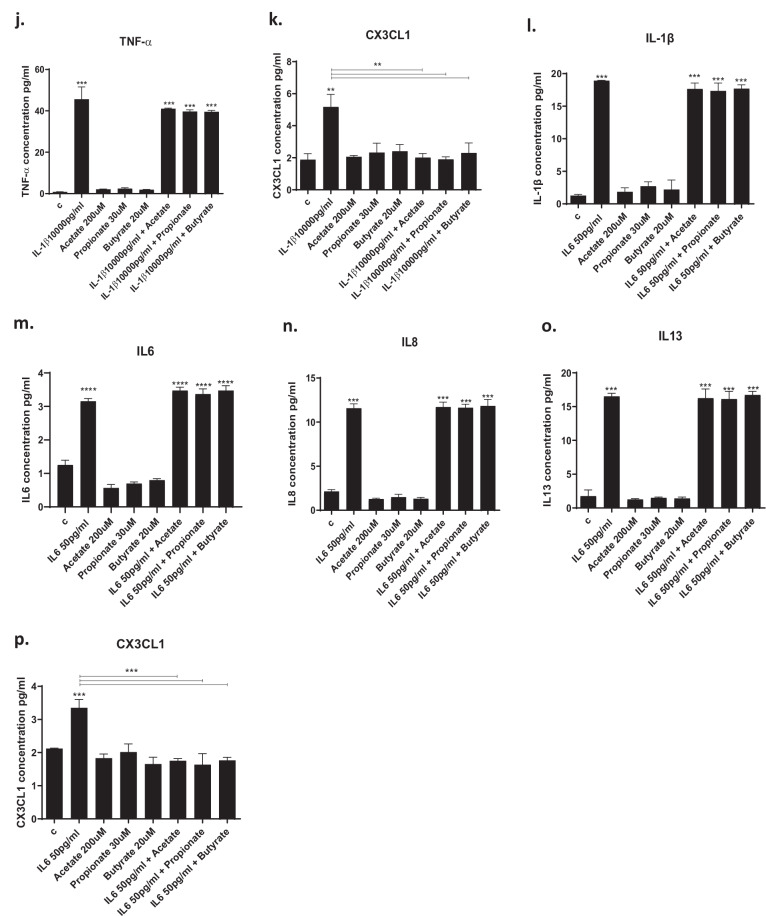
Cytokine levels in the cell supernatant after treatment with SCFAs. **a**–**k**. Treatment of cells with IL1β increased the production of IL1β, IL2, IL4, IL6, IL8, IL10, IL12, IL13, IFNγ, TNFα and CX3CL1, and treatment with acetate (200 μM), propionate (30 μM) and butyrate (20 μM) prevented the IL1β-mediated increase in IL6, IL12 and CX3CL1. **l**–**p**. Treatment of cells with IL6 increased the production of IL1β, IL6, IL8, IL13 and CX3CL1, and treatment with acetate, propionate and butyrate were able to decrease CX3CL1 levels. Two-way ANOVA with Bonferroni’s post hoc test was performed. Data are shown as mean ± SEM; **p < 0.01, ***p < 0.001, ****p < 0.0001, compared with vehicle treatment or as indicated.

IL1β increased the concentration of all cytokines, with the exception of IL8, in the cell supernatant (Fig. 3a–k). Interestingly, pre-treatment with SCFAs prevented only IL1β-induced increase in IL6 (acetate: from 317 to 1.7 pg/ml, *p* < 0.01; propionate: from 317 to 1.2 pg/ml, *p* < 0.01, butyrate: from 317 to 1.75 pg/ml, *p* < 0.01, Fig. 3d), IL12 (acetate: from 11.6 to 1.6 pg/ml, *p* < 0.01; propionate: from 11.6 to 1.4 pg/ml, *p* < 0.01; butyrate: from 11.6 to 1.4 pg/ml, *p* < 0.01; Fig. 3g) and CX3CL1 (acetate: from 5 to 2 pg/ml, *p* < 0.01; propionate: from 5 to 1.9 pg/ml, *p* < 0.01; butyrate: from 5 to 2.3 pg/ml, *p* < 0.01; Fig. 3k). However, none of the three SCFAs affected the levels of the other cytokines (Figs. 3a–c, e, f, h–j).

In contrast, IL6 increased the concentration of IL1β, IL6, IL8, IL13, and CX3CL1 (Fig. 3l–p). Interestingly, acetate, propionate, and butyrate prevented only IL6-induced increase in CX3CL1 (acetate: from 3.3 to 1.7 pg/ml, *p* < 0.01; propionate: from 3.3 to 1.6 pg/ml, *p* < 0.01; butyrate: from 3.3 to 1.8 pg/ml, *p* < 0.01; Fig. 3p), however, none of the three SCFAs affected the levels of the other cytokines (Fig. 3l–o).

Overall, these findings indicate that SCFAs modulate similar inflammatory cytokines when in the presence of IL1β vs IL6, with CX3CL1 being the one commonly regulated.

### Treatment with antibodies against candidate cytokines prevented IL1β- and IL6-induced reduction in neurogenesis and increase in apoptosis

**Fig. 4:**
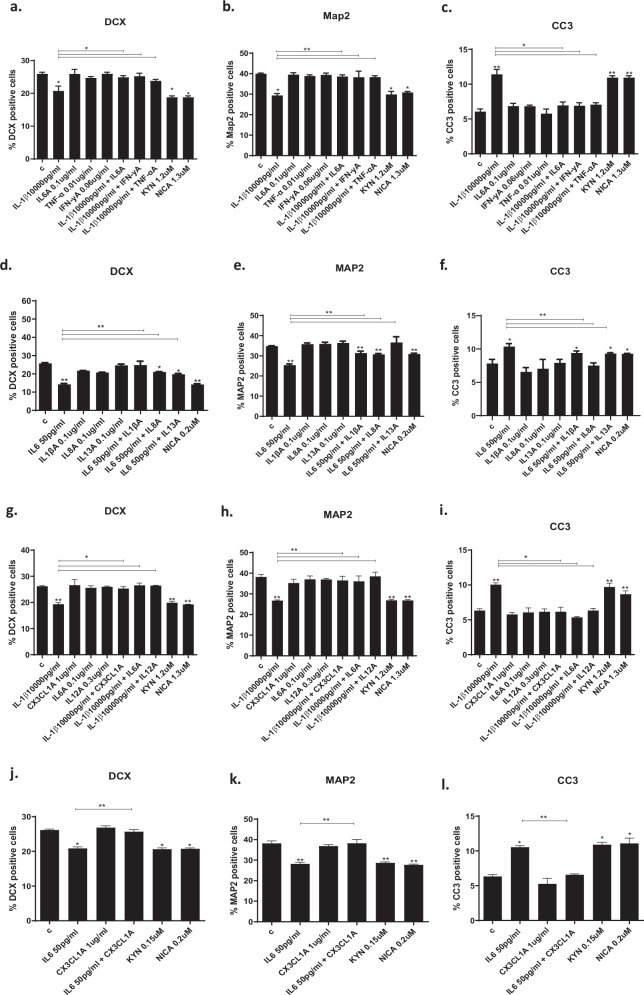
Treatment of hippocampal cells with antibodies against specific cell cytokines. **a**–**c** Treatment of cells with IL1β (10,000 pg/ml) and IL6A (0.1 ug/ml), IFNγA (0.06 ug/ml) and TNFαA (0.01 ug/ml) prevented the IL1β-induced decrease in DCX + and MAP2 + cells and increase in CC3 + cells. Treatment of cells with KYN (1.2 uM), NICA (1.3 uM), at the same concentration as when measured in the supernatant, decreased DCX + , MAP2 + cells and increased CC3 + cells. **d**–**f** Treatment of cells with IL6 (50 pg/ml) and IL1βA (0.1 ug/ml), IL8A (0.1 ug/ml) or IL13A (0.1 ug/ml) prevented the IL6-induced decrease in DCX + and MAP2 + cells and increase in CC3 + cells. Treatment NICA (0.2 uM) decreased DCX + , MAP2 + cells and increased CC3 + cells vs vehicle. **g**–**i** Treatment of cells with IL1β and CX3CL1A (1 ug/ml), IL6A (0.1 ug/ml) and IL12A (0.3 ug/ml) prevented the IL1β-induced decrease in DCX + and MAP2 + cells and increase in CC3 + cells. Treatment of cells with candidate kynurenine pathway metabolites, KYN (1.2 uM) and NICA (1.3 uM), decreased DCX + , MAP2 + cells and increased CC3 + cells. **j**–**l** Treatment of cells with IL6 and CX3CL1A (1 ug/ml) prevented the IL6-induced decrease in DCX + and MAP2 + cells and increase in CC3 + . Treatment with KYN (0.15 uM) and NICA (0.2 uM) decreased DCX + , MAP2 + cells and increased CC3 + cells. Two-way ANOVA with Bonferroni’s post hoc test was performed. Data are shown as mean ± SEM; *p < 0.05, **p < 0.01, compared with vehicle treatment or as indicated.

In order to confirm that the reduced production of the downstream cytokines previously identified in cell supernatant was indeed responsible for the beneficial effects exerted by EPEA and DHEA in the presence of IL1β, we treated cells with antibodies against these same downstream cytokines and measured neurogenesis and apoptosis again. Similar to treatment with EPEA and DHEA, treatment of cells with IL6 antibody (A) (0.1 ug/ml), IFNγA (0.06 ug/ml), and TNFαA (0.01 ug/ml) prevented the IL1β-induced decrease in DCX + (IL6A: +4%, *p* < 0.01 vs IL1β, IFNγA: +5%, *p* < 0.01 vs IL1β, TNFαA: +3%, *p* < 0.01 vs IL1β; Fig. 4a) and MAP2 + cells (IL6A: +9%, *p* < 0.01 vs IL1β, IFNγA: +9%, *p* < 0.01 vs IL1β, TNFαA: +9%, *p* < 0.01 vs IL1β; Fig. 4b) and increase in CC3 + cells (IL6A: −5%, *p* < 0.01 vs IL1β, IFNγA: −5%, *p* < 0.01 vs IL1β, TNFαA: −4%, *p* < 0.01 vs IL1β; Fig. 4c). Similar to treatment with EPEA and DHEA, treatment of cells with IL1βA (0.1 ug/ml), IL8A (0.1 ug/ml) or IL13A (0.1 ug/ml) prevented the IL6-induced decrease in DCX + (IL1βA: +10%, *p* < 0.01 vs IL6, IL8A: +6%, *p* < 0.01 vs IL6, IL13A: +5%, *p* < 0.01 vs IL6; Fig. 4d) and MAP2 + cells (IL1βA: +6%, *p* < 0.01 vs IL6, IL8A: +5%, *p* < 0.01 vs IL6, IL13A: +11%, *p* < 0.01 vs IL6; Fig. 4e) and increase in CC3 + cells (IL1βA: −1%, *p* < 0.01 vs IL6, IL8A: −3%, *p* < 0.01 vs IL6, IL13A: −1%, *p* < 0.01 vs IL6; Fig. 4f).

Similar to treatment with SCFAs, treatment of cells with CX3CL1A (1 ug/ml), IL6A (0.1 ug/ml) and IL12A (0.3 ug/ml) prevented the IL1β-induced decrease in DCX + (CX3CL1A: +6%, *p* < 0.01 vs IL1β, IL6A: +7%, *p* < 0.01 vs IL1β, IL12A: +7%, *p* < 0.01 vs IL1β; Fig. 4g) and MAP2 + cells (CX3CL1A: +10%, *p* < 0.01 vs IL1β, IL6A: +10%, *p* < 0.01 vs IL1β, IL12A: +12%, *p* < 0.01 vs IL1β; Fig. 4h) and increase in CC3 + cells (CX3CL1A: −4%, *p* < 0.01 vs IL1β, IL6A: −5%, *p* < 0.01 vs IL1β, IL12A: −4%, *p* < 0.01 vs IL1β; Fig. 4i). Furthermore, like treatment with SCFAs, treatment of cells with CX3CL1A (1 ug/ml) prevented the IL6-induced decrease in DCX + (+5, *p* < 0.01 vs IL6; Fig. 4j) and MAP2 + cells (+10, *p* < 0.01 vs IL6; Fig. 4k) and increase in CC3 + cells (−4, *p* < 0.01 vs IL6; Fig. 4l).

Overall, these results suggest that the detrimental effect exerted by IL1β and IL6 in neurogenesis and apoptosis are mediated by the aforementioned downstream cytokines, which, when inhibited by a selective antibody, could prevent IL1β- and IL6-induced effects, similar to treatment with either eCBs or SCFAs.

### EPEA and DHEA prevent IL1β- and IL6-induced activation of the kynurenine pathway

**Fig. 5:**
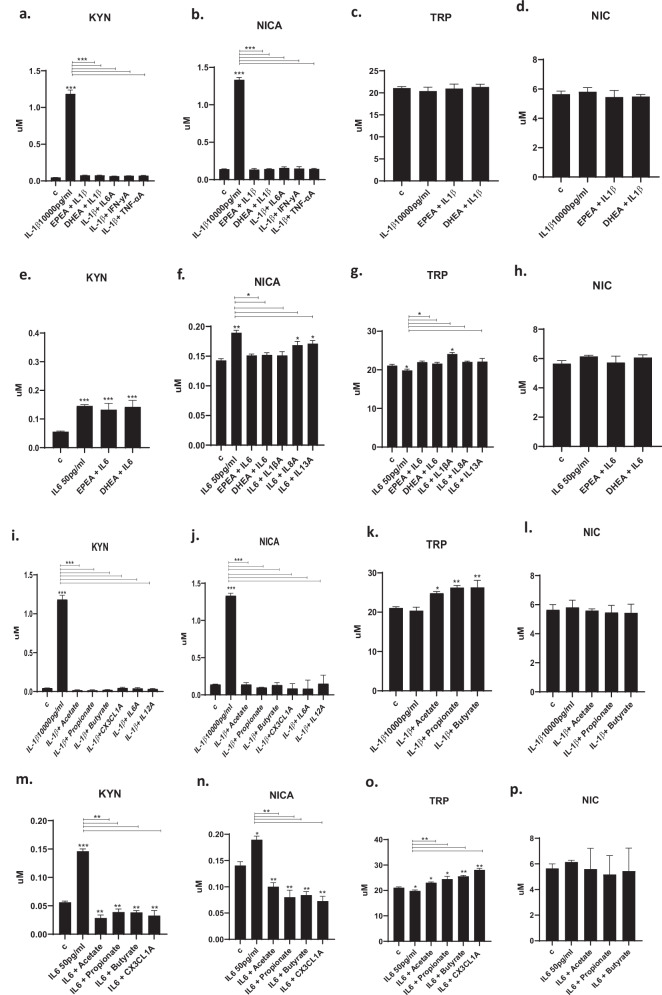
Measurement of kynurenine pathway metabolites in the cell supernatant after treatment with eCBs and SCFAs. **a**–**d**. We measured the regulation of KYN, NICA, TRP, NIC metabolites. Pre-treatment of EPEA (300 pM) or DHEA (700 pM) followed by IL1β (10,000 pg/ml), or direct treatment with IL1β (10,000 pg/ml) and antibodies against IL6 (IL6A, 0.1 ug/ml), IFNγ (IFNγA, 0.06 ug/ml) and TNFα (TNFαA, 0.01 ug/ml) prevented IL1β-induced increase in KYN and NICA. **e**–**h**. Pre-treatment of EPEA or DHEA followed by IL6 (50 pg/ml), or direct treatment with IL6 (50 pg/ml) and antibodies IL1βA (0.1 ug/ml), IL8A (0.1 ug/ml) or IL13A (0.1 ug/ml) prevented IL6-induced increase in NICA and IL6-induced decrease in TRP. **i**–**l**. Pre-treatment of acetate (200 uM), propionate (30 uM), or butyrate (20 uM) followed by IL1β, or direct treatment with IL1β and CX3CL1A (1 ug/ml), IL6A (0.1 ug/ml) and IL12A (0.3 ug/ml), prevented IL1β- induced increase in KYN and NICA. **m**–**p**. Pre-treatment with acetate, propionate or butyrate followed by IL6, or direct treatment with IL6 and CX3CL1A (1 ug/ml), prevented the increase in KYN and NICA, and the decrease in TRP. Two-way ANOVA with Bonferroni’s post hoc test was performed. Data are shown as mean ± SEM; *p < 0.05, **p < 0.01, ***p < 0.001, compared with vehicle treatment or as indicated.

Having shown the ability of eCBs and SCFAs to inhibit the IL1β- and IL6-induced production of downstream cytokines, which are responsible for the detrimental effects observed on neurogenesis and apoptosis, we subsequently measured candidate metabolites of the kynurenine pathway, in the presence of IL1β or IL6, either alone or in pre-treatment with eCBS or SCFAs. Specifically, treatment with IL1β increased KYN (+1.2 uM, *p* < 0.001, vs vehicle, Fig. 5a) and NICA (+1.3 uM, *p* < 0.001, vs vehicle, Fig. 5b), but was unable to change TRP (Fig. 5c) and NIC levels (Fig. 5d). In addition, treatment with EPEA and DHEA increased KYN levels (EPEA: +0.02 uM, *p* < 0.01, vs vehicle, Supplementary Fig. 2a; DHEA: +0.02 uM, *p* < 0.01, vs vehicle, Supplementary Fig. 2a), whereas EPEA decreased NICA levels (+0.02 uM, *p* < 0.01, vs vehicle, Supplementary Fig. 2b). However, we did not observe any change in TRP (Supplementary Fig. 2c) and NIC levels (Supplementary Fig. 2d).

Interestingly, pre-treatment with EPEA, DHEA, or with an antibody against IL6 (IL6A, 0.1 μg/ml), IFNγ (IFNγA, 0.06 μg/ml) or TNFα (TNFαA, 0.01 μg/ml) prevented IL1β-induced increase in KYN and NICA (Fig. 5a, b).

Similarly, treatment with IL6 increased KYN (+0.1 uM, *p* < 0.001, vs vehicle, Fig. 5e) and NICA (+0.2 uM, *p* < 0.001, vs vehicle, Fig. 5f), as well as decreased TRP (−19 uM, *p* < 0.001, vs vehicle, Fig. 5g), but did not change NIC levels (Fig. 5h). Pre-treatment with EPEA, DHEA, or with an antibody against IL1β (IL1βA, 0.1 μg/ml), IL8 (IL8A, 0.1 μg/ml), or IL13 (IL13A, 0.1 μg/ml), prevented IL6-induced increase in NICA and decrease in TRP (Fig. 5f, g).

Overall, this suggests that activation of the kynurenine pathway by IL1β or IL6 is indeed mediated by the production of the aforementioned downstream cytokines, which, when inhibited with a selective antibody, can prevent the effects on the pathway, like the action of the eCBs.

### SCFAs prevent IL1β- and IL6-induced activation of the kynurenine pathway

As previously discussed, IL1β increased KYN, NICA, but did not change TRP and NIC levels (Fig. 5i–l). In addition, treatment with propionate and butyrate increased NICA levels (propionate: +0.02 uM, *p* < 0.01, vs vehicle, Supplementary Fig. 2f; butyrate: +0.02 uM, *p* < 0.01, vs vehicle, Supplementary Fig. 2f). However, we did not observe any change in KYN (Supplementary Fig. 2e), TRP (Supplementary Fig. 2g), and NIC (Supplementary Fig. 2h).

However, in this case, pre-treatment with either acetate, propionate, butyrate, or CX3CL1A (1 μg/ml), IL6A (0.1 μg/ml), or IL12A (0.3 μg/ml) prevented IL1β-induced increase in KYN and NICA (Fig. 5i, j).

Similarly, and as previously discussed, treatment with IL6 increased KYN, NICA, and decreased TRP, but was unable to change NIC levels (Fig. 5m–p). Pre-treatment with acetate, propionate, butyrate, or CX3CL1A (1 μg/ml) prevented IL6-induced increase in KYN, NICA, and decrease in TRP (Fig. 5m–o).

Overall, this suggests that activation of the kynurenine pathway by IL1β or IL6 is indeed mediated by the production of the aforementioned downstream cytokines, which, when inhibited with a selective antibody, can prevent the activation of the pathway, similar to the action of the SCFAs.

### Treatment of cells with IL1β- or IL6-induced kynurenine metabolites decreases neurogenesis and increases apoptosis

In order to test whether these identified kynurenine metabolites could have detrimental effects on neurogenesis and apoptosis, we exposed cells directly to the same concentrations of KYN and NICA, previously identified upon treatment with IL1β or IL6 alone. Results showed that KYN (1.2 uM) and NICA (1.3 mM) decreased the percentage of DCX + cells (KYN: −7% vs vehicle, *p* < 0.01, NICA: −7% vs vehicle, *p* < 0.01; Fig. 4a) and MAP2 + cells (KYN: −11% vs vehicle, *p* < 0.05, NICA: −10% vs vehicle, *p* < 0.01; Fig. 4b), and increased the percentage of CC3 + cells (KYN: +4% vs vehicle, *p* < 0.01, NICA: 5% vs vehicle, *p* < 0.05; Fig. 4c) to the same level as treatment with IL1β. Additionally, treatment with KYN (0.15 uM) and NICA (0.2 uM) decreased the percentage of DCX + cells (KYN: −8% vs vehicle, *p* < 0.05, NICA: −7% vs vehicle, *p* < 0.05; Fig. 4j) and MAP2 + cells (KYN: −10% vs vehicle, *p* < 0.01, NICA: −8% vs vehicle, *p* < 0.01; Fig. 4k), and increased the percentage of CC3 + cells (KYN: +5% vs vehicle, *p* < 0.01, NICA: +5% vs vehicle, *p* < 0.01; Fig. 4l) to the same level as treatment with IL6.

Overall, these results suggest that eCBs and SCFAs prevent the anti-neurogenic effects of IL1β and IL6 via inhibition of cytokine production and subsequent inhibition of the cytokine-activated kynurenine pathway.

### eCBs prevented IL1β- and IL6-induced increase in the expression of inflammatory genes and altered the expression of distinct genes involved in neurogenesis and synaptic functions

**Fig. 6:**
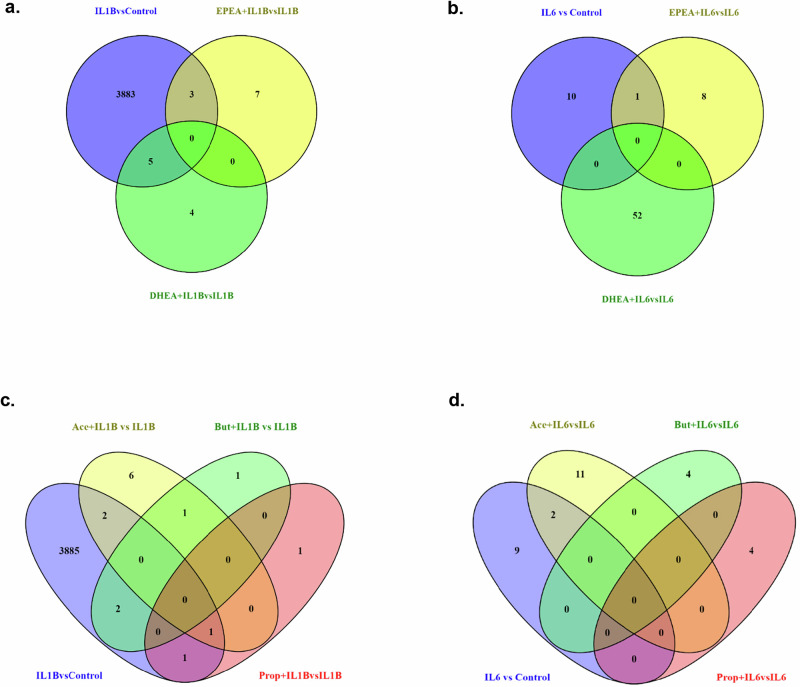
Venn diagrams indicating the number of differentially expressed genes (DEGs) in various conditions. **a**. Number of DEGs in IL1β vs Control, EPEA + IL1β vs IL1β and DHEA + IL1β vs IL1β conditions and the commons ones within them. **b**. Number of DEGs in IL6 vs Control, EPEA + IL6 vs IL6 and DHEA + IL6 vs IL6 conditions and the commons ones within them. **c**. Number of DEGs in IL1β vs Control, Acetate + IL1β vs IL1β, Propionate + IL1β vs IL1β and Butyrate + IL1β vs IL1β conditions and the commons ones within them. **d**. Number of DEGs in IL6 vs Control, Acetate + IL6 vs IL6, Propionate + IL6 vs IL6 and Butyrate + IL6 vs IL6 conditions and the commons ones within them.

We conducted transcriptomic analyses to investigate how treatment with the cytokines alone (IL1β or IL6) would affect gene expression and consequently modulate downstream signalling pathways relevant for the neuro-inflammatory changes that we previously observed, and ultimately to investigate if pre-treatment with eCBs or SCFAs was able to prevent any of these changes. Firstly, we found 3891 differentially expressed genes (DEGs) altered in IL1β vs control (Supplementary Table 1), 10 in cells pre-treated with EPEA followed by IL1β when compared with IL1β vs control (Supplementary Table 2), and 9 in DHEA followed by IL1β compared with IL1β (Supplementary Table 3, Fig. 6a). We also found 458 DEGs modulated in EPEA (Supplementary Table 4), 395 DEGs in DHEA alone vs control (Supplementary Table 5, Supplementary Fig. 3a). In addition, we found 262 pathways (Supplementary Table 6) and 25 networks in IL1β vs control (Supplementary Fig. 4a), 87 pathways in EPEA (Supplementary Table 7) and 79 in DHEA (Supplementary Table 8), and 6 networks in EPEA and 8 in DHEA (Supplementary Fig. 4b, c), both vs control, which are the only comparison from which we had DEGs > 50 and networks were therefore generated.

Amongst the numerous genes *uniquely* modulated in IL1β vs control, there was an increase in the expression of interferon-induced protein with tetratricopeptide 3 (*IFIT3*) (log2Fc: +4.67, adjusted p: 1.45e–265, Supplementary Table 1), vascular cell adhesion molecule 1 (*VCAM1*) (log2Fc: +4.98, adjusted p:1.37e–186, Supplementary Table 1), and signal transducer and activator of transcription 1 (*STAT1*) (log2Fc: +2.41, adjusted p: 6.65e–162, Supplementary Table 1), which are all involved in inflammation and belong to the interferon gamma (z-score: 6.10, p: 2e–16, Supplementary Table 6) and interferon alpha/beta signalling pathways (z-score: 6.32, p: 1.58e–15, Supplementary Table 6), and in cell-to-cell interactions and immune cell trafficking networks (Supplementary Fig. 4a). Amongst the genes *uniquely* modulated in cells pre-treated with EPEA followed by IL1β when compared with IL1β alone, there was an increase in the expression of cytoplasmic linker associated protein 2 (*CLASP2)* gene (log2Fc: +0.68, adjusted p: 0.033, Supplementary Table 2), which is a key regulator of axo-dendritic outgrowth and synaptic activity and formation [38], whereas, in cells treated with DHEA followed by IL1β compared with IL1β alone, there was an increase in the expression of NMDA subunit 1 (*GRIN1*) (log2Fc: +1.67, adjusted p: 0.0001, Supplementary Table 3), which is important for neuroblast development [39].

However, more importantly, there were 2 *common*, relevant genes that were differentially modulated in cells exposed to IL1β alone when compared with EPEA plus IL1β condition. The first gene, U3 small nucleolar ribonucleoprotein homolog C subunit processome component (*UTP14C)*, which is associated with increased DNA methylation in schizophrenia [40], was increased in IL1β alone (log2Fc: +11.78, adjusted p: 0.0004, Supplementary Table 1 and Supplementary Fig. 5a) but decreased in cells exposed to EPEA plus IL1β (log2Fc: −11.59, adjusted p: 0.098, Supplementary Table 2 and Supplementary Fig. 5a), whereas, the second gene, FRY microtubule binding gene (*FRY)*, which is involved in neuron projection development, was decreased in IL1β (log2Fc: −2.08; adjusted p: 1.47e–18, Supplementary Table 1), but increased in EPEA plus IL1β (log2Fc: +1.29, adjusted p: 0.04, Supplementary Table 2), indicating the beneficial action of EPEA in the context of IL1β. Furthermore, there was one *common*, relevant gene that was differentially modulated in cells exposed to IL1β alone, when compared with DHEA plus IL1β. In particular, the gene MMS22 like DNA repair gene (*MMS22L)*, which is involved in tumorigenesis, was increased in IL1β alone (Fc: +1.05, adjusted p: 5.03e–8, Supplementary Table 1 and Supplementary Fig. 5b), but decreased in DHEA plus IL1β (Fc: −0.96, adjusted p: 0.054, Supplementary Table 3 and Supplementary Fig. 5b).

Similarly, we found 11 DEGs in IL6 vs control (Supplementary Table 9), 9 in cells pretreated with EPEA followed by IL6 (Supplementary Table 10), and 52 in DHEA followed by IL6 compared to IL6 conditions (Supplementary Table 11, Fig. 6b). Amongst the genes *uniquely* modulated in IL6 vs control, there was an increase in the expression of colony stimulating factor 1 receptor (*CSF1R*) (log2Fc: +6.6, adjusted *p* < 0.001, Supplementary Table 9), which regulates the production, differentiation, and function of microglia which is relevant in the context of neuroinflammation [41], whereas, EPEA plus IL6 vs IL6, there was an increase in the expression of potassium gated channel subunit B1 (*KCNB1*) (log2FC: +2.78, adjusted p: 0.014, Supplementary Table 10), which has been shown to regulate hippocampal neurogenesis [42]. Finally, in cells exposed to DHEA plus IL6 vs IL6, we found an increase in the expression of neurexin 3 (*NRXN3)* (log2Fc: +1.17, adjusted p: 0.090, Supplementary Table 11), which is involved in synaptic adhesion and stabilisation [43], as well as a modulation of 4 networks (Supplementary Fig. 4d).

Interestingly, there was only 1 *common*, relevant gene that was differentially modulated in IL6 alone when compared with EPEA plus IL6. In particular, the expression of ADAM metallopeptidase with thrombospondin type 1 motif 1 (*ADAMTS1*) was increased in IL6 (log2FC: 15.51, adjusted p: 0.024, Supplementary Table 9, and Supplementary Fig. 5c), but decreased in EPEA plus IL6 (log2Fc: −16.24, adjusted p: 0.048, Supplementary Table 10, and Supplementary Fig. 5c).

Overall, we found that EPEA and DHEA were able to prevent the modulation of neuroinflammatory and neuroplasticity genes involved in the detrimental effects of IL1β and IL6.

### SCFAs prevented IL1β- and IL6-induced increase in the expression of inflammatory genes, and the expression of distinct genes involved in neurogenesis and receptor functions

Having previously discussed genes which were uniquely modulated by treatment with eCBs, we report findings for uniquely and commonly modulated genes by pre-treatment with SCFAs followed by IL1β vs IL1β alone. Overall, we found 10 DEGs altered in acetate plus IL1β vs IL1β (Supplementary Table 12), 3 in propionate plus IL1β vs IL1β (Supplementary Table 13) and 4 in butyrate plus IL1β vs IL1β (Supplementary Table 14, Fig. 6c). We also found 498 DEGs modulated by acetate (Supplementary Table 15), 386 propionate (Supplementary Table 16), 479 butyrate alone vs control (Supplementary Table 17, Supplementary Fig. 3b), and 119 pathways in acetate (Supplementary Table 18), 107 in propionate (Supplementary Table 19), 129 in butyrate (Supplementary Tables 20), and 11 networks in acetate, 8 in propionate, 8 in butyrate vs control (Supplementary Fig. 4e–g).

Amongst the genes *uniquely* modulated in propionate plus IL1β vs IL1β, there was a decrease in casein kinase 1 epsilon (*CSNK1E*) (log2Fc: −4.27, adjusted p: 0.08, Supplementary Table 13), which is involved in encephalopathy and in hypoxia [44]. Amongst the genes *uniquely* modulated in butyrate plus IL1β vs IL1β, there was a decrease in the expression of protocadherin 19 (*PCDH19)* (log2Fc: −0.89, adjusted p: 0.007 Supplementary Table 14), which is involved in signal transduction at synapses and refractory epilepsy [45].

Interestingly, there was only 1 *common*, relevant gene that was differentially modulated in IL1β alone, when compared with acetate plus IL1β and propionate plus IL1β. In particular, the same aforementioned neuroplasticity gene, *FRY*, was decreased in IL1β alone (log2Fc: −2.08; adjusted p: 1.47e–18, Supplementary Table 1 and Supplementary Fig. 5d), whereas it was increased in acetate plus IL1β (log2Fc: +1.44, adjusted p: 0.007, Supplementary Table 12 and Supplementary Fig. 5d) and propionate plus IL1β (log2Fc: 1.364, adjusted p: 0.04, Supplementary Table 13 and Supplementary Fig. 5d). Furthermore, we also saw an increase in the expression of retinoid-related orphan receptor alpha (*RORA*) in IL1β alone (log2FC: +1.43, adjusted p: 0.0002, Supplementary Table 1 and Supplementary Fig. 5e), whereas it was decreased in butyrate plus IL1β (log2Fc: −1.52, adjusted p: 0.057, Supplementary Table 14 and Supplementary Fig. 5e).

Similarly, we found 13 DEGs in acetate plus IL6 vs IL6 (Supplementary Table 21), 4 in propionate plus IL6 vs IL6 (Supplementary Table 22) and 4 in butyrate plus IL6 vs IL6 conditions (Supplementary Table 23, Fig. 6d). Amongst the genes *uniquely* modulated in acetate plus IL6 vs IL6, we observed a decrease in the expression of poly (ADP-ribose) polymerase family member 8 (*PARP8*) (log2Fc: −17.7, adjusted p: 3.09e–07, Supplementary Table 21), which is involved in apoptosis. Additionally, in propionate plus IL6 vs IL6, we found a decrease in CXC motif chemokine ligand 10 (*CXCL10*) (log2Fc: −2.91, adjusted p: 0.021, Supplementary Table 22), also involved in inflammation [46]. Whereas, amongst the genes *uniquely* modulated in butyrate plus IL6 vs IL6, we observed a decrease in GTF2I repeat domain containing 2 (*GTF2IRD2*) gene (log2Fc: −0.85, adjusted p: 0.0201, Supplementary Table 23), which regulates transcription of synaptic proteins [47].

Again, there was only 1 *common* gene that was differentially modulated in IL6 alone when compared with acetate plus IL6, but none with propionate and butyrate. In particular, the same aforementioned neuroinflammatory gene *ADAMTS1*, was increased with IL6 alone (log2FC: 15.51, adjusted p:0.024, Supplementary Table 9, and Supplementary Fig. 5c), whereas it was decreased in acetate plus IL6 (log2Fc: −27.3, adjusted p: 4.29e–10, Supplementary Table 21, and Supplementary Fig. 5c). Overall, we found that SCFA increased the expression of genes involved in synaptic receptors and decreased the expression of inflammatory genes, as well as prevented the detrimental effects of IL1β and IL6.

## Discussion

This is the first study which provides evidence that eCBs and SCFAs can regulate cytokine-mediated reduction of human hippocampal neurogenesis and apoptosis through altering genes and proteins belonging to neuroinflammatory and neuroplasticity signalling pathways as well as the prevention of the production of candidate metabolites of the kynurenine pathway. In particular, we found that treatment with either eCBs or SCFAs prevented IL1β- and IL6-induced reduction in neurogenesis and increase in apoptosis. These effects were mediated by IL1β-induced production (release) of IL6, IFN-γ, and TNF-α, and by IL6-induced production of IL1β, IL8, and IL13, all of which were prevented by treatment with eCBs. In contrast, IL1β-induced production of IL6, IL12, and CX3CL1, and IL6-induced production of CX3CL1 were instead prevented by SCFAs. Treatment with IL1β and IL6 also increased the production of candidate kynurenine pathway metabolites, such as KYN and NICA, which again were prevented by eCBs and SCFAs. We then conducted transcriptomic analysis to investigate genes and signalling pathways relevant for the neuro-inflammatory changes previously observed, and putatively prevented by eCBs and SCFAs treatment. In particular, we found that the expression of *FRY* and *UTP14C*, which are involved respectively in neuroplasticity and neuroinflammation, were differentially modulated in cells treated with IL1β alone when compared to cells pre-treated with either EPEA, acetate, or propionate followed by IL1β. Similarly, we found that the expression of *ADAMTS1*, again involved in inflammatory responses, was increased in IL6 alone when compared to cells pre-treated with either EPEA, or acetate followed by the cytokine. Altogether, we identify novel anti-inflammatory and neurogenic mechanisms mediating the effect of eCBs and SCFAs on human hippocampal neurogenesis, which can be of value as potential future treatment candidates in the context of neuropsychiatric symptoms.

Firstly, we demonstrate that IL1β and IL6 were able to alter neurogenesis and apoptosis in our human hippocampal neuronal model. This is in line with our previous findings, exposing the same cells to these cytokines [6, 9, 33]. Interestingly, however, treatment alone with EPEA and DHEA did not affect neither neurogenesis nor apoptosis. This this effect could be probably due to the fact that eCBs are known to regulate internal homeostasis and without the presence of a challenge they are not exerting their beneficial properties [48]. In line with this, for the first time, we were able to show that both EPEA and DHEA were equally efficacious in preventing IL1β-induced reduction in neurogenesis and increase in apoptosis. Interestingly, we showed that both EPEA and DHEA can prevent IL6-induced reduction in neurogenesis, whereas only EPEA, but not DHEA, prevented IL6-induced increase in apoptosis. In particular, EPEA could prevent IL6-induced increase in only DCX + cells, but not MAP2 + cells, whereas DHEA could prevent both DCX + and MAP + cells, thus suggesting that DHEA is more pro-neurogenic than EPEA, whereas EPEA could be more anti-apoptotic in the context of IL6. This is consistent with our previous studies where we demonstrate that EPA, the precursor of EPEA, can prevent the increase of IL1β-/IL6- and cortisol-induced apoptosis, whereas DHA, precursor of DHEA, can prevent reduction in neurogenesis independent of the type of cytokine or cortisol exposure [6, 9, 49]. This has also been demonstrated in clinical trials consisting of individuals with MDD and high inflammation in which treatment with EPA improved depressive symptoms more than DHA, and a meta-analysis study of RCTs on omega-3 fatty acids demonstrated reduced depression severity with formulations containing pure EPA or greater than 60% EPA, but not with supplements containing pure DHA or greater than 60% DHA [8, 20, 50]. In contrast, while treatment alone with SCFAs did not exert any effect on neither neurogenesis nor apoptosis similar to eCBs, all three SCFAs were equally efficacious in preventing *both* IL1β- and IL6-induced decrease in neurogenesis and increase in apoptosis. This is particularly relevant since this is the first evidence showing that eCBs as well as SCFAs can be differentially effective in preventing inflammation-induced detrimental neurogenic changes in the context of human hippocampal neurogenesis. Altogether, both eCBs and SCFAs can regulate IL1β- and IL6-induced reduction in neurogenesis and increase in apoptosis.

Furthermore, we demonstrate that the effect of EPEA and DHEA in preventing IL1β-induced decrease in neurogenesis was mediated by reducing the production of downstream cytokines, namely IL6, IFNγ, and TNFα, which were activated by IL1β. These findings are in agreement with a previous preclinical study whereby DHEA treatment reduced IL1β and IL6 levels alongside increasing animal hippocampal neurogenesis [51]. Increased levels of IL6, IFNγ, and TNFα have been found in patients with MDD [1, 52, 53]. We also observed that all three SCFAs equally reduced IL1β- and IL6-induced production of CX3CL1 (also known as fractalkine), which is in line with a previous study that showed that butyrate inhibited IL1β-induced CX3CL1 expression [54]. High levels of fractalkine were observed in a clinical study consisting of individuals with moderate-severe depression [55, 56]. We also found that both eCBs and SCFAs modulated IL1β-induced downstream production of IL6. Importantly, we were able to confirm and extend results obtained in previous studies which indicated that eCBs and SCFAs could decrease IL6 levels in adipocytes and murine macrophages [12, 57], by demonstrating the activity of these dietary ligands in neuronal cells. This is particularly relevant in the context of MDD, since previous studies have shown significantly increased plasma IL6 levels in these patients [58]. Overall, our results indicate that eCBs and SCFA differentially modulate the production of cytokines and highlight the capacity of both eCBs and SCFAs in reducing IL6, which could play a key role in mediating their anti-depressant actions.

In terms of kynurenine pathway metabolites, eCBs and SCFAs were able to prevent IL1β-induced increase in KYN production. This is consistent with previous research from our laboratory which demonstrates that EPA and DHA can reduce kynurenine levels [9], and expand previous research on SCFAs which demonstrates that butyrate can inhibit indoleamine 2,3-dioxygenase-1 (IDO-1) expression and downstream kynurenine pathway activation in intestinal epithelial cells [59]. Similarly, eCBs and SCFAs were able to prevent IL6-induced reduction of TRP, which is in line with previous studies which indicate that SCFAs can regulate the expression levels of tryptophan 5-hydroxylase 1 and 5-hydroxytryptamine biosynthesis, although these studies were mainly conducted in intestinal cells [60–62]. These findings have clinical relevance since previous studies have demonstrated that higher levels of plasma kynurenine pathway metabolites were associated with increased depressive symptoms [63], and a recent mendelian randomisation study specifically indicated that elevated levels of kynurenine have a causal relationship with an increased risk of developing depression [64]. However, eCBs and SCFAs were also able to prevent IL1β- and IL6-induced increase in NICA, which in most studies is considered neuroprotective [65, 66]. Indeed, NICA plays a key role in sustaining nicotinamide adenine dinucleotide (NAD+) levels during neuroinflammation [65, 66]. This mechanism is vital for cell survival and shielding against inflammatory damage, making it a potential therapeutic target for modulating neuroinflammatory responses [67]. One possible explanation for this finding could be due to the activation of neuroprotective compensatory mechanisms as a consequence of the detrimental exposure of our cells to IL1β and IL6, which might have altered the internal cell homeostasis. Indeed, both eCBs and SCFAs are acting towards constant maintenance of homeostasis [68, 69], which might explain their ability to prevent the production of the aforementioned metabolites by inhibition of the downstream cytokines previously activated by IL1β (IL6, IFNγ, TNFα, and CX3CL1) and IL6 (IL1β, IL8, IL13, and CX3CL1). Ultimately, this provides us with an additional understanding of the complex mechanisms being targeted by eCBs and SCFAs in the presence of IL1b, when compared with IL6.

On a transcriptomic level, we showed that IL1β and IL6 are able to *uniquely* increase the expression of genes involved in inflammation (*IFIT3, STAT1*) and microglial activation (*CSFIR1*), respectively, and belonging to the interferon and neuroinflammation signalling pathways. Interestingly, this is in line with our previous finding demonstrating the ability of IL1β to induce the production of IFN proteins in cell supernatant, and mediating the activation of the kynurenine pathway [6, 9, 70]. Accordingly, in our previous study, we demonstrated that IFN proteins can subsequently reduce neurogenesis and enhance apoptosis via downstream activation of *STAT1*, which further stimulated interferon-related ubiquitin-like proteins, such as interferon (IFN)-stimulated gene 15 *(ISG15)*, ubiquitin Specific Peptidase 18 *(USP18S)*, in addition to *IL6* genes [36]. Previous clinical research have demonstrated an increase in mRNA transcripts for *STAT1* [21] and *USP18S* [71] in individuals with depression. Of note, IL1β had a more pronounced transcriptional effect on cells when compared with IL6, while both IL1β and IL6 were able to affect neurogenesis. This result suggests that changes also at protein level, such as those in cytokines, might have significantly contributed to the effects of IL6.

Furthermore, when cells are treated with eCBs and SCFAs followed by IL1β or IL6, we found an increase in genes involved in synaptic protein interactions and neuroplasticity (*NRXN3)*, and a decrease in genes involved in apoptosis, oxidative stress, and inflammation (*PARP8, CXCL10)*. Decreased levels of NRXN3 proteins were found in the CSF of MDD patients in comparison to the controls; therefore, the addition of these dietary ligands could be beneficial in restoring the levels of these synaptic proteins to homeostatic levels and alleviating depressive symptoms [72]. Moreover, an increase in the expression of poly(ADP-ribose) polymerase (PARP) genes has been demonstrated in the white matter of patients with MDD [73], and preclinical studies using PARP inhibitors demonstrated their antidepressant-like effects in the forced swim test, which were similar to the effect of fluoxetine [74]. Taken together, eCBs and SCFAs increase the expression of synaptic proteins and decrease those associated with inflammation, which could promote an antidepressant effect.

Interestingly, we found *common* genes modulated by both eCBs and SCFAs, when in presence of the cytokines. *FRY*, a gene involved in cell morphogenesis and neuron projection development [75], was downregulated in cells treated with IL1β, but it was upregulated in cells exposed to EPEA, acetate, or propionate plus IL1β. Of relevance, previous evidence has shown that depletion of FRY was able to dramatically affect synapse development and microtubule sliding in motor neurons [75, 76], suggesting that eCBs and SCFAs can regulate synapse development and neuroplasticity. Additionally, we observed an increase in *ADAMTS1* in cells treated with IL6, but a decrease in cells exposed to EPEA or acetate plus IL6. *ADAMTS1* is an enzyme which plays a role in the degradation of extracellular matrix in the brain and has been shown to increase after cerebral artery occlusion [77], suggesting that immunomodulatory role of EPEA and acetate could be through regulating metalloproteinases. Although previous clinical studies have not specifically investigated the role of ADAMTS1, studies have shown an increase in the expression of other matrix metalloproteinases (MMPs), namely MMP2 and MMP7, in individuals with depression compared to control groups [78]. Therefore, the regulation of metalloproteinases by eCBs and SCFA could be an important mechanism for reducing depressive symptoms.

The results of this study indicate that eCBs and SCFAs may uniquely modulate outcomes of neurogenesis, inflammatory cytokines, and kynurenine pathway metabolites, depending on whether they are in the presence of IL1β or IL6. However, both of the dietary ligands share similarities in their pro-neurogenic and anti-inflammatory mode of actions which could be beneficial for psycho-cardiometabolic multimorbidity. Numerous clinical studies have demonstrated that omega-3 fatty acids have been beneficial for the improvement of symptoms of depression, particularly in people with higher baseline inflammation levels [79–81], and their anti-inflammatory effect has been beneficial for reducing [76] and waist circumference in obese adults [82]. Furthermore, clinical trials with SCFAs interventions in patients with depression have not been investigated thus far, however, studies on their beneficial role in people with obesity and other cardiometabolic disorders are currently underway [83]. While administration of SCFAs may be absorbed in the stomach before reaching the intestine [84], intravenous administration of butyrate or mixtures of SCFAs has so far been shown to be effective in reducing both gastric and peripheral inflammation in patients with inflammatory bowel disease [85]. Considering the above, the results from our study could help elucidate the mechanisms underlying the inflammatory changes seen in clinical studies and can help direct future studies in which participants are stratified on the basis of their baseline inflammation levels. This would eventually pave the way for more effective and personalised treatment interventions.

Nevertheless, this study has a few limitations. Firstly, the in vitro model, whilst being of invaluable importance for our understanding of molecular mechanisms occurring in the hippocampus, may not fully recapitulate the brain milieu of an adult organism in vivo, especially because of the absence of microglia cells. However, while theoretically, this system may differ from the scenario of an adult in vivo environment and the adult neurogenic niche, over the years we have been able to replicate all our results with this in vitro model in either animal or clinical studies, including changes in neurogenesis by cortisol, IL1β, IFN-α, and antidepressants and changes in stress- and antidepressant-regulated genes in both the whole-blood mRNA of depressed patients and the hippocampal mRNA of animal models of depression [9, 34, 86, 87]. Therefore, we are confident that our results are relevant to the human brain. Of note, we did not assess the effect of eCBs and SCFAs on astrogliogenesis, and in the future, we want to expand our study to understand their effect on astrocytic markers and neuroinflammation. Also, it remains unclear whether the observed effects are specific to hippocampal progenitor cells or if they extend to other neuronal populations. Further investigation is needed to determine the cell-type specificity of these effects. Finally, the aim of this study was to investigate the distinct effect of omega-derived eCBs and SCFAs on neuroinflammation, rather than studying a possible interaction between the two systems. Future investigations are needed to determine any cross-link between eCBs and SCFAs.

In conclusion, this first study to identify the underlying mechanisms by which dietary ligands, namely eCBs and SCFAs, mitigate the detrimental effects of inflammation on a human hippocampal model of neurogenesis. We demonstrate that EPEA, DHEA, acetate, propionate, and butyrate are equally effective in preventing the anti-neurogenic and proinflammatory effects of cytokines, IL1β and IL6, via modulating the downstream kynurenine pathway and the expression of distinct genes. Altogether, this study opens avenues for future research which involve targeting these signalling mechanisms to develop novel therapeutics for individuals with depression.

## Supplementary information

Supplementary Materials

## Data Availability

All data generated or analysed during this study are included in this published article and its supplementary information files.
