# Supplementary material for: The dietary ligands, omega-3 fatty acid endocannabinoids and short-chain fatty acids prevent cytokine-induced reduction of human hippocampal neurogenesis and alter the expression of genes involved in neuroinflammation and neuroplasticity
Source: Mol Psychiatry. 2025 Jul 16;30(11):5338–55. doi: 10.1038/s41380-025-03119-5 (PMC12532594; doi:10.1038/s41380-025-03119-5)
Supplement: Supplementary file 1 — Supplementary Materials [file 41380_2025_3119_MOESM1_ESM.pdf]

For submission to Molecular Psychiatry- Supplementary Materials

The dietary ligands, omega-3 fatty acid endocannabinoids and short-chain fatty acids, prevent cytokine-induced reduction of human hippocampal neurogenesis and alter the expression of genes involved in neuroinflammation and neuroplasticity

Gargi Mandal, MSc[1], Silvia Alboni, PhD[2], Nadia Cattane, PhD[3], Moira Marizzoni[3], MSc, Samantha Saleri[3], MSc, Nikita Arslanovski, BSc[4], Nicole Mariani, MSc[1], Madeline Kirkpatrick, MSc[1], Annamaria Cattaneo, PhD[3], Carmine M. Pariante, PhD[1], Alessandra Borsini, PhD[1*]

[1] Section of Stress, Psychiatry and Immunology Laboratory, Institute of Psychiatry, Psychology and Neuroscience, Department of Psychological Medicine, King's College London, UK

[2]Department of Life Sciences, University of Modena and Reggio Emilia, Modena, Italy

[3]Biological Psychiatry Laboratory, IRCCS Fatebenefratelli, Brescia, Italy

[4]Department of Behavioural Science and Health, University College London, UK

* Corresponding Author

Alessandra Borsini, PhD

Stress, Psychiatry and Immunology Lab & Perinatal Psychiatry

Institute of Psychiatry, Psychology and Neuroscience, King's College London

G.32.01, The Maurice Wohl Clinical Neuroscience Institute

Cutcombe Road, London, SE5 9RT

Tel: 020 7848 0726

Email: alessandra.borsini@kcl.ac.uk

METHODS

Cell culture

Human hippocampal multipotent progenitor cell line HPC0A07/03 (HPC; ReNeuron Ltd, Surrey, UK), sourced from 12-week-old female fetal tissue in accordance with UK and USA ethical and legal guidelines, was used for the experiments. This cell line has been conditionally immortalised via infection with a retroviral vector pLNCX2, encoding the c-MycERTAM transgene construct, which is solely activated by the synthetic steroid 4-hydroxytamoxifen (4-OHT). HPCs were cultured in reduced modified medium (RMM), namely Dulbecco's Modified Eagle's Media/F12 (DMEM: F12, Sigma), supplemented with 0.03% human albumin solution (Zenalb), 100 µg/mL human apo-transferrin, 16.2 µg/mL human putrescine diHCl, 5 µg/mL human recombinant insulin, 60 ng/mL progesterone, 2 mM L-glutamine and 40 ng/mL sodium selenite. For proliferation, the medium also included 10 ng/mL human basic fibroblast growth factor (bFGF), 20 ng/mL human epidermal growth factor (EGF) and 100nM 4-OHT. Consequently, 4-OHT, bFGF and EGF were removed from the culture medium, enabling the cells to differentiate into neurons, astrocytes, and oligodendrocytes. Cells were grown in $25cm^2$ filtered cap culture flasks (Nunclon, Roskilde, Denmark) at 37 °C in 5% CO2 and regulatory passaged at 80% confluence before being transferred to plates. All cultures were regularly checked for mycoplasma infection.

Immunocytochemistry

Neuronal differentiation was assessed with DCX and MAP2, and apoptosis with CC3 after 6 days of differentiation upon treatment with EPEA (300 pM), DHEA (700 pM), Acetate (200 uM), Propionate (30 uM) and Butyrate (20 uM), and cytokines (10,000 pg/ml) and IL6 (50 pg/ml)). For immunocytochemistry, PFA-fixed cells were incubated for 1hr at room temperature in blocking solution, consisting of phosphate buffered saline (PBS), 5% normal donkey serum (D9963, Sigma) and 0.3% 7 Triton-X (93443, Sigma). Cells were then incubated in 30µl of primary antibodies (rabbit anti-DCX, 1:500; mouse anti-MAP2 [HM], 1:500, Abcam, rabbit anti-CC3, 1:500, Abcam) at 4°C overnight. The next day, cells were incubated in blocking solution for 30 minutes, secondary antibodies (Alexa 488 donkey anti-rabbit; 1:1000; Alexa donkey 555 anti-mouse, 1:1000, Invitrogen) for 2 hours, and then 50µl of 300µm 4',6-diamidino-2-phenylindole solution diluted in PBS (DAPI 33342 dye (0.02 mg/mL, Sigma-Aldrich) for 5 minutes at RT. The number of DCX, MAP2 and CC3 over total DAPI positive cells was counted with CellInsight NXT High Content Screening (HCS) platform (ThermoScientific) and Studio Cell Analysis Software (Thermo Scientific). Three independent experiments were conducted on a minimum of 3

independent cultures, and each condition was tested in triplicate, as done in previous experiments with these cells [1–6].

Automated Quantification of Immunofluorescence

An automated approach using CellInsight NXT High Content Screening Platform (ThermoScientific) was employed to quantify cell number, markers of differentiation, and cell death (Figure Supplementary 1). The iDEV workflow facility within the Cell Insight scanning software is characterized by the following steps: acquire images; primary object identification channel 1 (DAPI); primary target selection channel 2 (CC3 and DCX); and primary target selection channel 3 (MAP2). Once focused on nuclear staining (DAPI) to determine fields of interest, next exposures were adjusted in channels 2 and 3 to obtain the optimum exposure times for all wells and conditions across the 96-well plate. Image saturation was set within the 20% to 30% range to avoid under- or overexposure. Following this, images at 10x magnification were acquired that represent positive and negative staining. Based on values from the negative staining controls and good positive staining, distinct thresholds were set for average intensity of target regions of interest (nuclear or cell body) to delineate positive populations in both channels 2 and 3. These settings were applied to each well upon scanning of the plate and to all corresponding plates within an experiment, ensuring reproducibility and unbiased comparisons.

Multiplex Cytokine Measurement

Cell supernatants of differentiated cells were run on the Human ProInflammatory Multileplex Very-Sensitive Kit from Meso Scale Discovery using to the manufacturers' instructions for the measurement of 10 candidate cytokines. The chemokine CX3CL1 was measured using U-PLEX assay, again from MSD. In terms of the method, 50 µL of prepared samples was added into each well of the MSD plate, which was subsequently incubated for 2 hours with vigorous shaking at 700 rpm at RT. The plate was then washed 3 times with 150 µL/well of Wash Buffer, and 25 µL of detection antibody solution was added to each well followed by another 2 hours incubation with vigorous shaking at 700 rpm at RT. Finally, the plate was washed 3 times with 150 µL/well of Wash Buffer, and 150 µL of 2x Read Buffer T was added to each well. The plate was analysed using the SECTOR Imager machine for the measurement of IL1β, IL2, IL4, IL6, IL8, IL10, IL12, IL13, TNFα and IFNγ.

Liquid Chromatography

The concentration of kynurenine pathway metabolites was measured at day 4 of differentiation. Fifty µL of supernatants were added with an equal volume of ice-cold 1 M perchloric acid ($HClO_4$) fortified with a mix of the following stable isotope-labeled internal standard (final concentration 1 µM): L-kynurenine-d4, kunurenic acid-d5 (Buchem BV) and L-Tryptophan-d5 (Sigma-Aldrich). Samples were centrifuged (15000 × g, 15 min) and the supernatants were collected and directly injected into LC-MS/MS. The analyses of kynurenine (KYN), tryptophan (TRP), anthranilic acid (ANA), kynurenic acid (KYNA), 3-hydroxy kynurenine (3-HK), 3-Hydroxyanthranilic Acid (3-HANA), quinolinic acid (QUIN), nicotinic acid (NICA) and nicotinamide (NIC) in the supernatant were performed using an Agilent HP 1200 liquid chromatograph (Agilent, Milan, Italy) consisting of a binary pump, an autosampler and a thermostated column compartment. Chromatographic separations were carried out using a Discovery HS-F5 column (3µm particle size, 150 x 2.1 mm, Supelco, Milan, Italy) using 0.1% formic acid in water and acetonitrile (ACN) as mobile phase. The HPLC analyses were carried out using a linear elution profile of 15 min from 5% to 90% of ACN. The column was washed with 90% ACN for 3.5 minutes, then equilibrated for 5 minutes with 5% ACN. The flow rate was 0.5 mL/min. The injection volume was 20 µL. An Agilent 6410 triple quadrupole-mass spectrometer with an electrospray ion source operating in positive mode was used for detection. The SRM pairs were 205–>188, 209–>192, 138–>120, 190->144, 225->208, 123->80, 124–>80, 154->136 and 168->78 for Trp, KYN, ANA, KYNA, 3HK, NICA,NIC, 3HANA and QUIN respectively. The calibration curves were constructed using calibration standards and were linear over the concentration range of 0.0064–5.000 µM for ANA, KYNA, 3HK, 3HANA, QUIN and NICA; 0.0128–10.00 µM for KYN, and NIC and 0.1280–100 µM for TRP, with a correlation coefficient (r2) included between the values 0.9979 and 0.9991. Of all the metabolites measured, only TRP, KYN, NICA and NIC were above detection limit.

RNA isolation, cDNA synthesis and Quantitative real-time PCR (qPCR)

RNA was isolated from cells in 6-well plates using the RNeasy Micro Kit (Qiagen, Crawley, UK) following manufactures instructions. Samples were stored at -80°C from 5 biological replicates for a total of 18 conditions (control, IL1β, acetate+IL1β, butyrate+ IL1β, propionate+IL1β, EPEA+IL1β, DHEA+IL1β, IL6, acetate+IL6, butyrate+IL6, propionate+IL6, EPEA+IL6, DHEA+IL6, Acetate, Butyrate, Propionate, EPEA, DHEA). RNA quality and quantity were assessed by evaluation of the A260/280 and A260/230 ratios using a Nanodrop spectrometer (Nanodrop Technologies, Wilmington, DE, USA). For cDNA synthesis, 1 µg of RNA was reverse transcribed using Superscript III enzyme (Invitrogen, Carslbad, CA, USA).

Briefly, a mix of 1 µg RNA, 250 ng random hexamers (Life Technologies), and 1 mM dNTP mix (Thermo Scientific) was used to made up a 13-µL solution with nuclease-free H_2O (Sigma-Aldrich). The mix was incubated for 5 minutes at 65°C on a heated block to denature RNA secondary structure. Subsequently, it was placed on ice and incubated for 1 minute to allow the annealing process. A volume of 7 µL consisting of 1x First Strand Buffer (Invitrogen), 5 mM dithiothreitol (Life Technologies), 40 units RNaseOUT (Life Technologies), 200 units SuperScript III Reverse Transcriptase (Life Technologies), and 3 µL of nuclease-free H_2O (Sigma-Aldrich) was made and added to the samples. Samples now containing a total volume of 20 µL were incubated at 25ºC for 5 minutes, 50ºC for 1 hour, 55ºC for 30 minutes, and finally 70 ºC for 15 minutes to terminate the reaction. Samples were diluted to a concentration of 1:10 in nuclease-free H_2O for subsequent qPCR analysis.

Gene expression and Pathway analysis

Transcriptome library preparation was performed by using the Illumina Stranded mRNA Prep Ligation kit. Libraries were sequenced on a NextSeq 2000 Illumina platform. The quality of the raw data was checked by using FastQ and the raw read counts were quantified at the transcript level using Salmon (v 1.4.0). Next, the transcript-level differential expression was assessed using DESeq2 (v1.30.1) in R. Filtering of low-abundance data was conducted and genes with less than ten reads across all the analysed samples were removed. All samples were of good quality, as confirmed by QCs. By performing bioinformatics analyses, we obtained several lists of differentially expressed genes (DEGs), one for each comparison which will be described below. DEGs were identified by using an adjusted p-value ≤ 0.1 and a log2 FC ± 0.59 (FC ± 1.5) as threshold, meaning a 50% modulation in the gene expression levels. We used the obtained lists of DEGs to perform pathway analyses by using Ingenuity Pathway Analysis (IPA) (Qiagen).

RESULTS

eCBs alter genes involved in inflammation and cell proliferation, differentiation and cell survival

We observed 458 DEGs to be significantly regulated in EPEA vs control (Supplementary Table 4), 395 DEG in DHEA vs control (Supplementary Table 5), and 312 genes in common between EPEA and DHEA (Figure Supplementary 3a), as well as 118 pathways modulated in EPEA (Supplementary Table 7) and 113 in DHEA (Supplementary Table 8), and 6 networks in EPEA and 8 in DHEA vs control (Supplementary Figure 4b-c).

Amongst the numerous genes *uniquely* modulated in EPEA vs control, there was a decrease in the expression of intercellular adhesion molecule 1 (*ICAM1*) (log2Fc: -0.707, adjusted p: 0.02, Supplementary Table 4), which plays a key role in inflammatory response [7], and belong to the pathogen induced cytokine storm signalling (z-score: -3.545, p: 1.17e-6, Supplementary Table 7) and neuroinflammation signalling pathways (z-score: -1.155, p: 5.89e-04, Supplementary Table 7), as well as immune cell trafficking networks (Supplementary figure 4b). Amongst the numerous genes *uniquely* modulated in DHEA vs control, there was a decrease in the expression of toll-like receptor 4 (*TLR4*) (log2Fc: -0.76, adjusted p: 0.03, Supplementary Table 5), which is involved in driving inflammatory response [8], and it belongs to immunogenic cell death signalling pathway (z-score: -2.236, p: 2.34e-02, Supplementary Table 8). Interestingly, amongst the many *common* genes that are modulated in the same direction in EPEA and DHEA vs control, we found that the expression of dickkopf-1 (*DKK1*), a Wnt-signalling pathway inhibitor associated with reduced neurogenesis, to be decreased in both EPEA (log2Fc: -9.01, adjusted p: 4.99e-05, Supplementary Table 4) and DHEA vs control (log2Fc: -10.31, adjusted p: 2.08e-21, Supplementary Table 5) [9].

SCFAs alter genes involved in inflammation and cell proliferation, differentiation and cell survival

We observed 498 DEGs in acetate (Supplementary Table 15), 386 DEGs in propionate (Supplementary Table 16), 479 DEGs in butyrate vs control (Supplementary Table 17) and 251 genes in common between the SCFAs (Supplementary Figure 3b), as well as 119 pathways modulated in acetate (Supplementary Table 18), 107 in propionate (Supplementary Table 19), 129 in butyrate (Supplementary Table 20), and 11 networks in acetate, 8 in propionate, 8 in butyrate (Supplementary Figure 4e-g) vs control. Amongst the numerous genes uniquely modulated in acetate vs control, there was a decrease in the expression of C-C chemokine receptor type 1 (*CCR1*) (log2Fc: -1.88, adjusted p: 0.081, Supplementary Table 15), which regulates neuroinflammation in acetate vs control [13], which belongs to pathogen induced cytokine storm signalling pathway (z-score: -3.710, p: 4.79e-05, Supplementary Table 18). Amongst the genes *uniquely* modulated by propionate vs control, there was a decrease in serpin family E member 1 (*SERPINE1*) (log2Fc: -1.24, adjusted p: 5.56e-16, Supplementary Table 16), which been shown to be elevated in ischaemic stroke [14], which belongs to acute phase response pathway (z-score: -1.633, p: 2.88e-3, Supplementary Table 19) and inflammatory response network. Amongst the uniquely modulated genes in butyrate vs control, there was in increase in neurotrophic tyrosine receptor kinase (*NTRK2*) (log2Fc: +1.79 , adjusted p: 5.11e-6, Supplementary Table

17), which is a susceptibility gene for psychiatric disorders and is involved in cell proliferation, survival and differentiation [15], as well as an increase in synaptogenesis signalling (z-score: 0.277, p: 4.47e-2, Supplementary Table 20). These results suggest that among the SCFAs, acetate and propionate may have higher anti-inflammatory properties, and butyrate may have a stronger proneurogenic effect. Amongst, the many *common* genes between acetate, propionate and butyrate, we found an increase in complexin 2 (*CPLX2),* which regulates synaptic signalling and synaptic plasticity, with acetate (log2Fc: +2.97, adjusted p: 0.000547, Supplementary Table 15), propionate (log2Fc: +2.56, adjusted p: 0.000306, Supplementary Table 16) and Butyrate (log2Fc: +2.76, adjusted p: 0.017, Supplementary Table 17) [16].

References

1. Anacker C, Cattaneo A, Luoni A, Musaelyan K, Zunszain PA, Milanesi E, et al. Glucocorticoid-Related Molecular Signaling Pathways Regulating Hippocampal Neurogenesis. Neuropsychopharmacology. 2013;38:872–883.
2. Borsini A, Alboni S, Horowitz MA, Tojo LM, Cannazza G, Su K-P, et al. Rescue of IL-1β-induced reduction of human neurogenesis by omega-3 fatty acids and antidepressants. Brain Behav Immun. 2017;65:230–238.
3. Borsini A, Cattaneo A, Malpighi C, Thuret S, Harrison NA, Zunszain PA, et al. Interferon-Alpha Reduces Human Hippocampal Neurogenesis and Increases Apoptosis via Activation of Distinct STAT1-Dependent Mechanisms. Int J Neuropsychopharmacol. 2018;21:187–200.
4. Borsini A, Stangl D, Jeffries AR, Pariante CM, Thuret S. The role of omega-3 fatty acids in preventing glucocorticoid-induced reduction in human hippocampal neurogenesis and increase in apoptosis. Transl Psychiatry. 2020;10.
5. Borsini A, Nicolaou A, Camacho-Muñoz D, Kendall AC, Di Benedetto MG, Giacobbe J, et al. Omega-3 polyunsaturated fatty acids protect against inflammation through production of LOX and CYP450 lipid mediators: relevance for major depression and for human hippocampal neurogenesis. Mol Psychiatry. 2021. June 2021. https://doi.org/10.1038/s41380-021-01160-8.
6. Du Preez A, Lefèvre-Arbogast S, González-Domínguez R, Houghton V, de Lucia C, Low DY, et al. Impaired hippocampal neurogenesis in vitro is modulated by dietary-related endogenous factors and associated with depression in a longitudinal ageing cohort study. Mol Psychiatry. 2022;27:3425–3440.
7. Bui TM, Wiesolek HL, Sumagin R. ICAM-1: A master regulator of cellular responses in inflammation, injury resolution, and tumorigenesis. J Leukoc Biol. 2020;108:787–799.
8. Trotta T, Porro C, Calvello R, Panaro MA. Biological role of Toll-like receptor-4 in the brain. J Neuroimmunol. 2014;268:1–12.
9. Seib DRM, Corsini NS, Ellwanger K, Plaas C, Mateos A, Pitzer C, et al. Loss of Dickkopf-1 Restores Neurogenesis in Old Age and Counteracts Cognitive Decline. Cell Stem Cell. 2013;12:204–214.
10. Beffert U, Dillon GM, Sullivan JM, Stuart CE, Gilbert JP, Kambouris JA, et al. Microtubule Plus-End Tracking Protein CLASP2 Regulates Neuronal Polarity and Synaptic Function. J Neurosci. 2012;32:13906–13916.
11. Napoli AJ, Laderwager S, Zoodsma JD, Biju B, Mucollari O, Schubel SK, et al. Developmental loss of NMDA receptors results in supernumerary forebrain neurons through delayed maturation of transit-amplifying neuroblasts. Sci Rep. 2024;14:3395.
12. Adanty C, Qian J, Al-Chalabi N, Fatemi AB, Gerretsen P, Graff A, et al. Sex differences in schizophrenia: a longitudinal methylome analysis. J Neural Transm. 2022;129:105–114.
13. Yan J, Zuo G, Sherchan P, Huang L, Ocak U, Xu W, et al. CCR1 Activation Promotes Neuroinflammation Through CCR1/TPR1/ERK1/2 Signaling Pathway After Intracerebral Hemorrhage in Mice. Neurotherapeutics. 2020;17:1170–1183.
14. Kubota M, Yoshida Y, Kobayashi E, Matsutani T, Li S-Y, Zhang B-S, et al. Serum anti-SERPINE1 antibody as a potential biomarker of acute cerebral infarction. Sci Rep. 2021;11:21772.
15. Spalek K, Coynel D, Freytag V, Hartmann F, Heck A, Milnik A, et al. A common NTRK2 variant is associated with emotional arousal and brain white-matter integrity in healthy young subjects. Transl Psychiatry. 2016;6:e758–e758.
16. Begemann M, Grube S, Papiol S, Malzahn D, Krampe H, Ribbe K, et al. Modification of Cognitive Performance in Schizophrenia by Complexin 2 Gene Polymorphisms. Arch Gen Psychiatry. 2010;67:879–888.

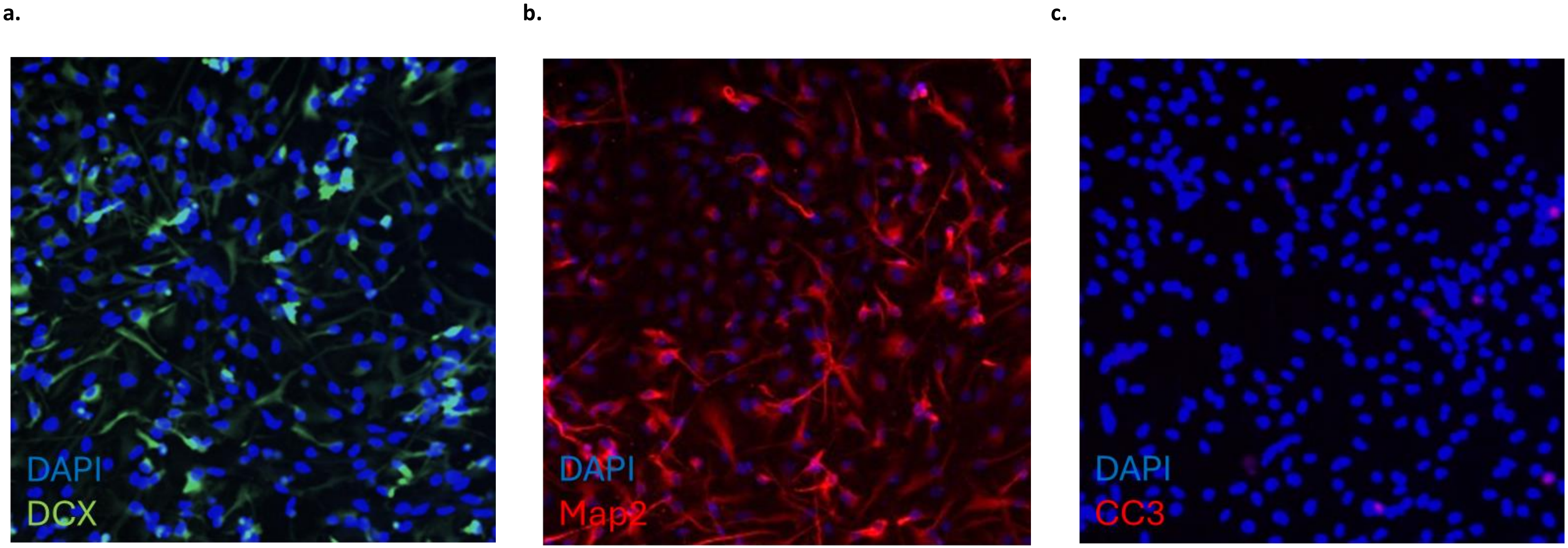

Supplementary Figure 1. Representative immunostaining images of neurogenic and apoptotic markers in control condition. Cells were treated with media containing EGF, bFGF, 4-OHT for 1 day during proliferation, followed by 4 days during differentiation with media without growth factors. Neuroblasts were detected by DCX (green) over the total number of cells DAPI (blue) (a), mature neurons were detected by Map2 (red) over the total number of cells DAPI (blue) (b), whereas apoptotic cells were stained by CC3 (red) over the total number of cells DAPI (blue) (c). Scalebar: 10µm.

Supplementary Figure 2. Changes in kynurenine pathway metabolites concentrations in presence of eCBs, SCFAs or cytokines antibodies alone (a-h). One-way ANOVA with Bonferroni's post hoc test was performed. Data are shown as mean±SEM; *p<0.05, **p<0.01, compared with vehicle treatment.

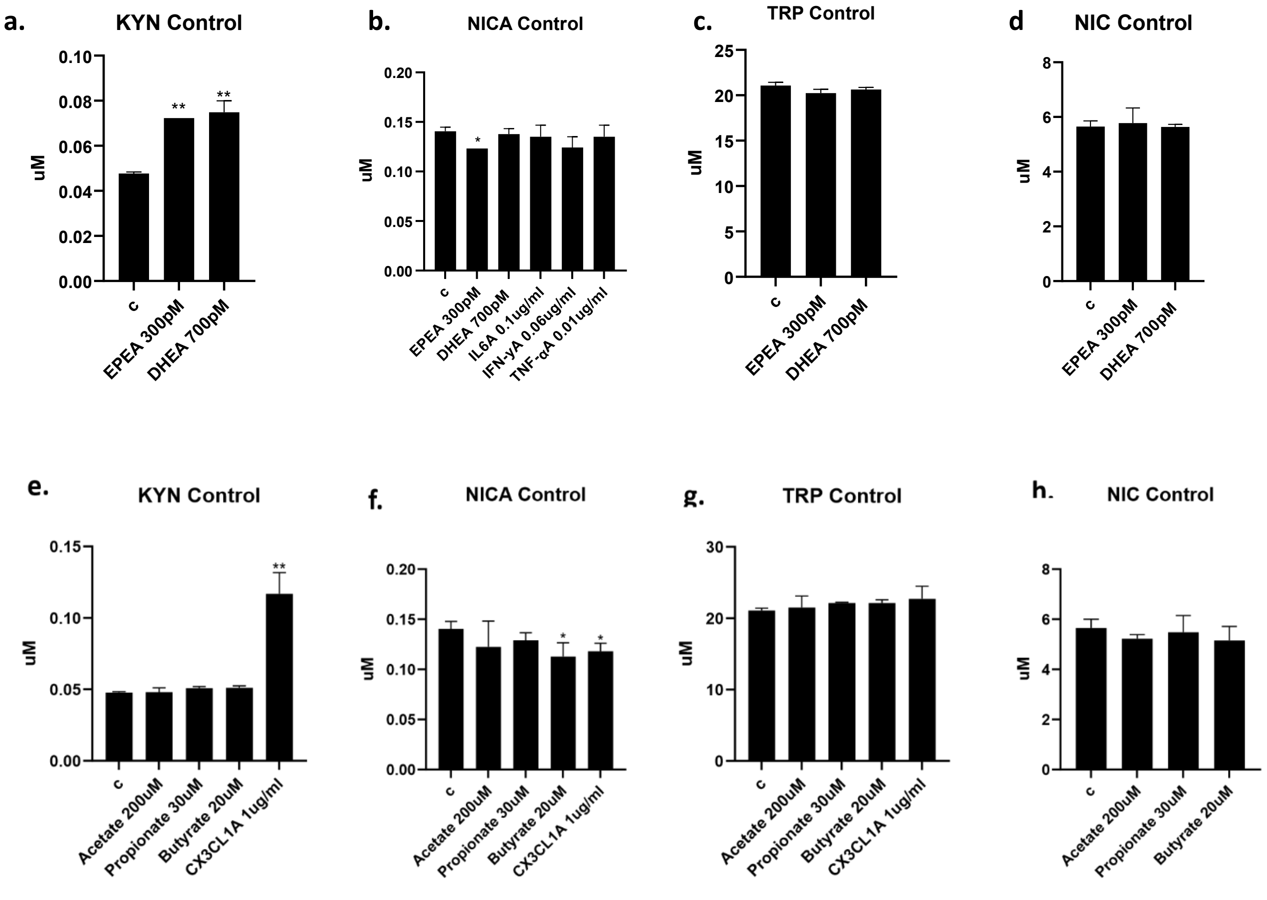

Supplementary Figure 3. Venn diagram of genes modulated upon treatment of cells with eCBs or SCFAs, when compared with control condition (a-b).

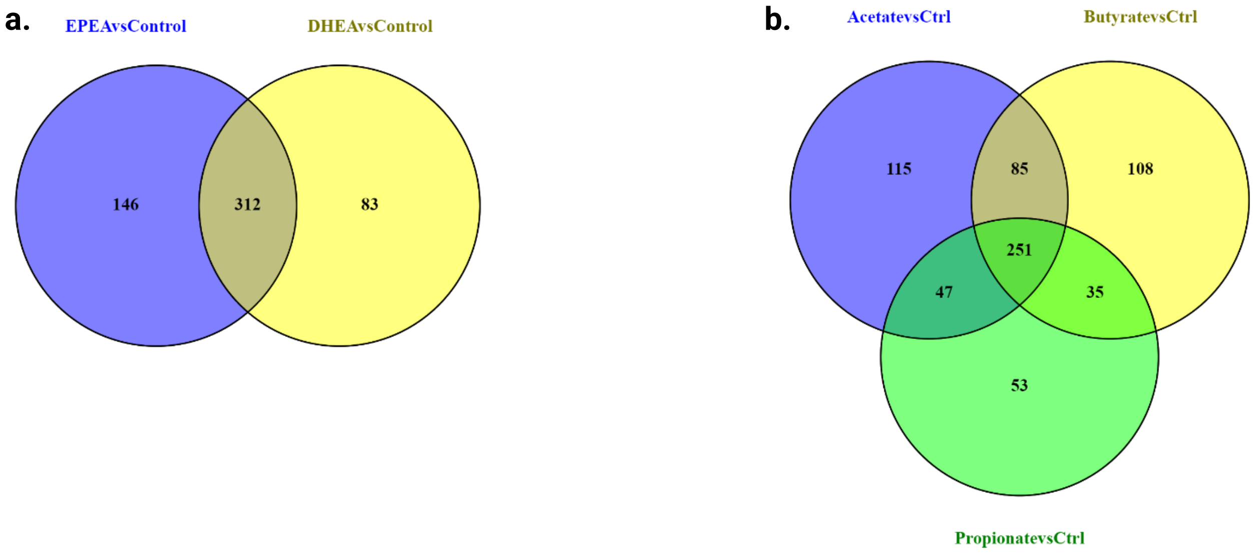

Supplementary figure 4: Network diagrams for a. IL1B vs Control, b. EPEA vs Control, c. DHEA vs control, d. DHEA+IL6 vs IL6, e. Acetate vs Control, f. Propionate vs Control, g. Butyrate vs Control

a. IL1B vs Control

Network 1

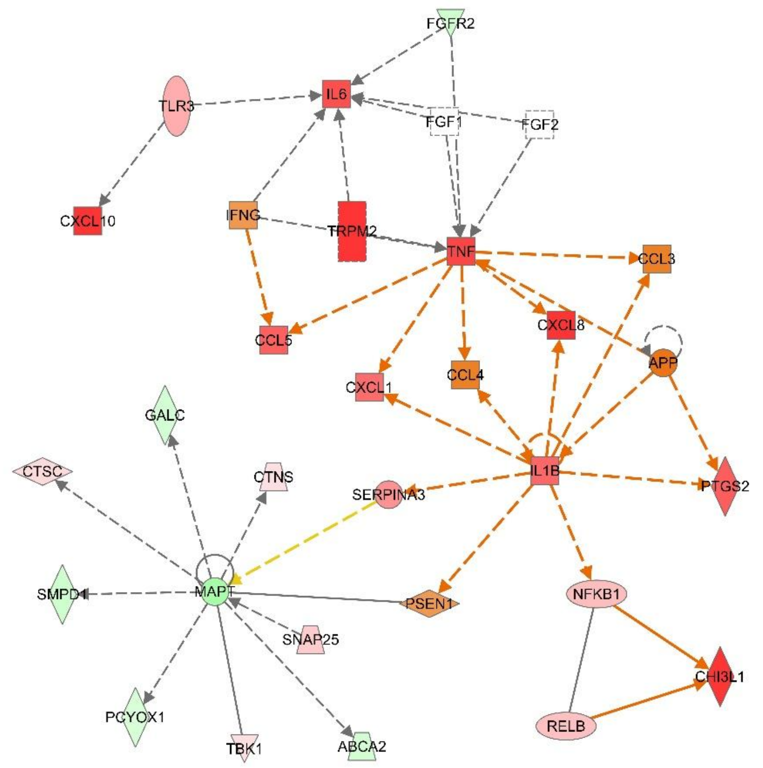

© 2000-2024 QIAGEN. All rights reserved.

Network 2

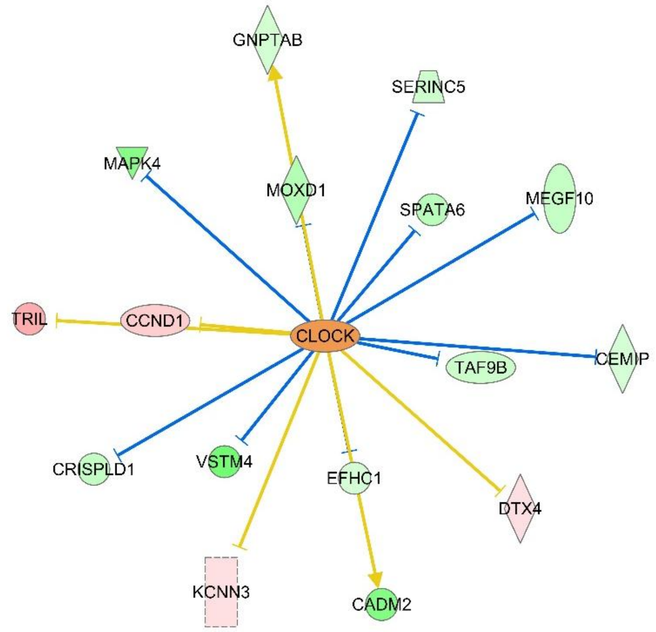

© 2000-2024 QIAGEN. All rights reserved.

Network 3

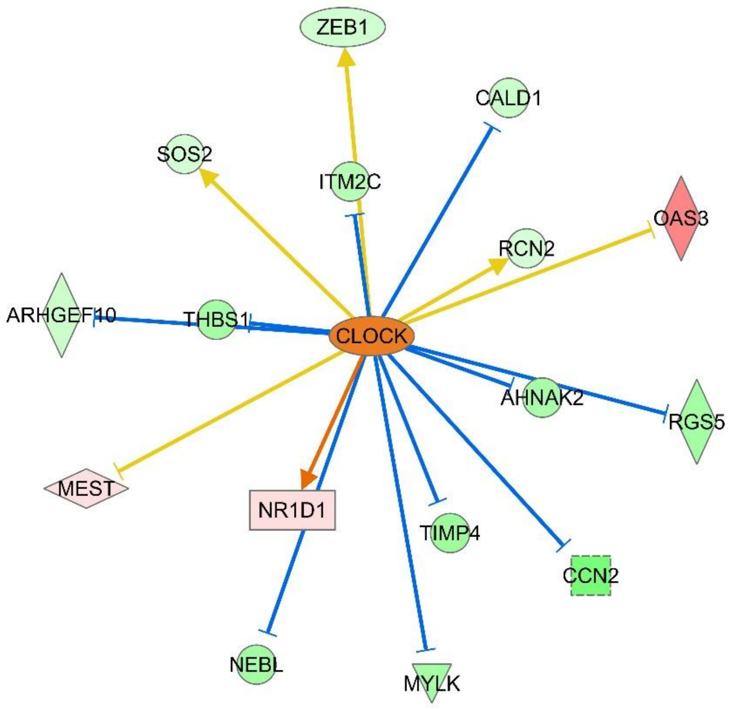

© 2000-2024 QIAGEN. All rights reserved.

Network 4

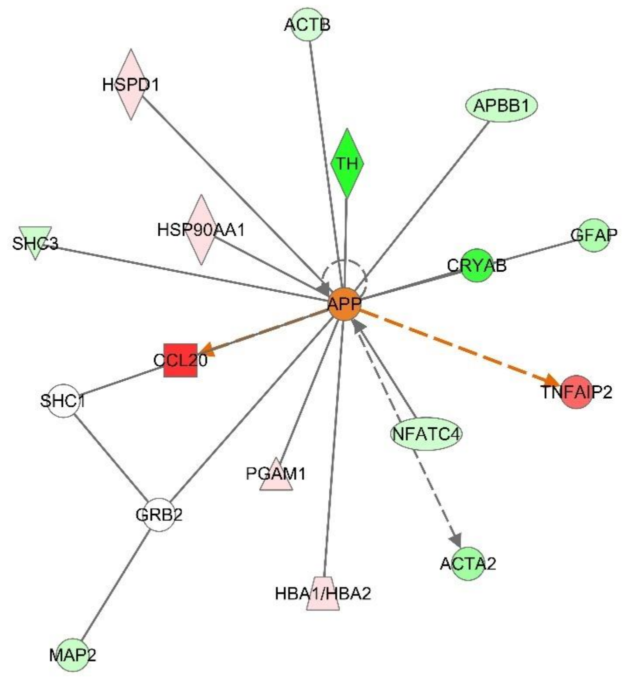

© 2000-2024 QIAGEN. All rights reserved.

Network 5

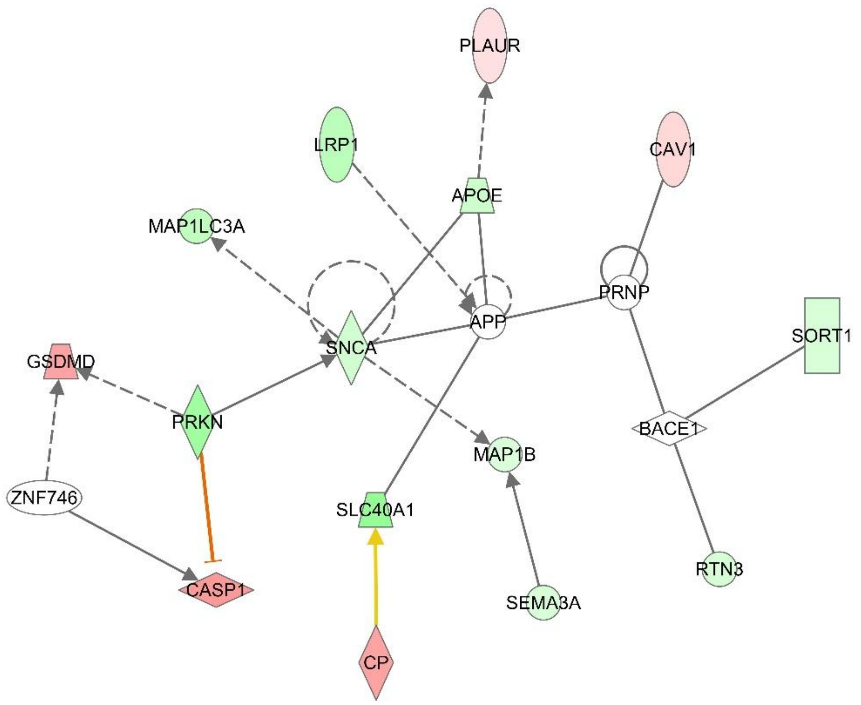

© 2000-2024 QIAGEN. All rights reserved.

Network 6

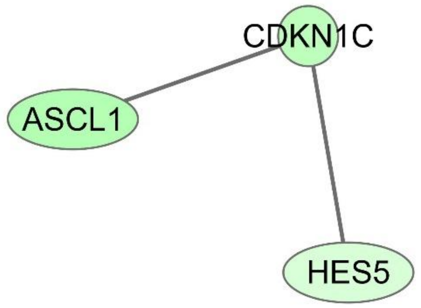

© 2000-2024 QIAGEN. All rights reserved.

Network 7

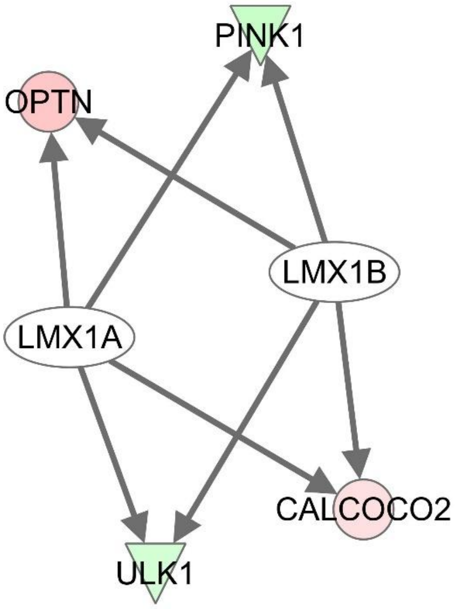

© 2000-2024 QIAGEN. All rights reserved.

Network 8

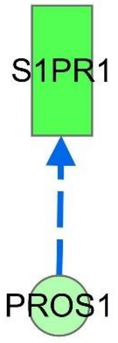

© 2000-2024 QIAGEN....

Network 9

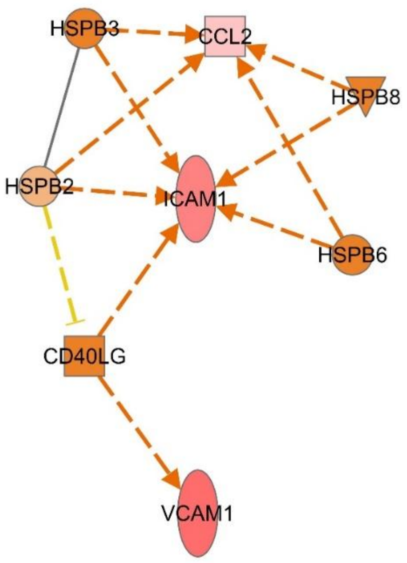

© 2000-2024 QIAGEN. All rights reserved.

Network 10

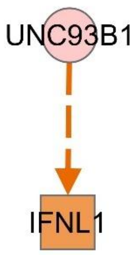

© 2000-2024 QIAGEN. All...

Network 11

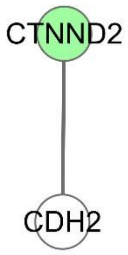

© 2000-2024 QIAGEN. ...

Network 12

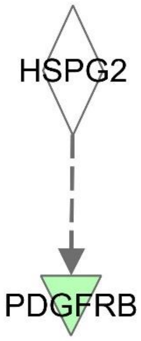

© 2000-2024 QIAGEN. A...

Network 13

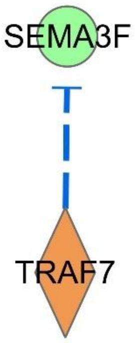

© 2000-2024 QIAGEN. A...

Network 14

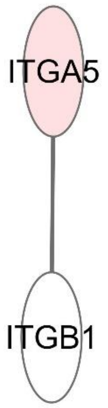

© 2000-2024 QIAGE...

Network 15

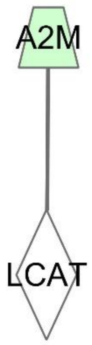

© 2000-2024 QIAG...

Network 16

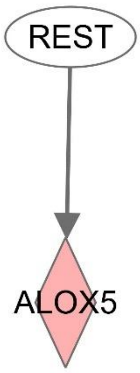

© 2000-2024 QIAGEN. ...

Network 17

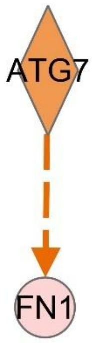

© 2000-2024 QIAG...

Network 18

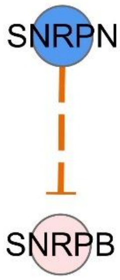

© 2000-2024 QIAGEN. ...

Network 19

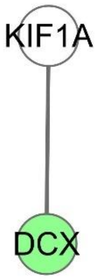

© 2000-2024 QIAGE...

Network 20

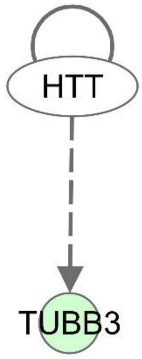

© 2000-2024 QIAGEN. ...

Network 21

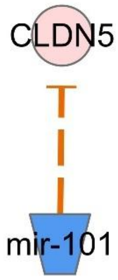

© 2000-2024 QIAGEN....

Network 22

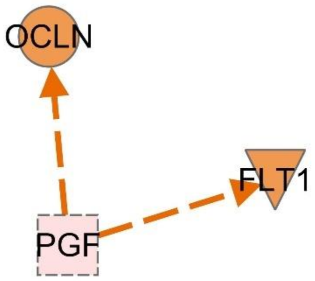

© 2000-2024 QIAGEN. All rights reserved.

Network 23

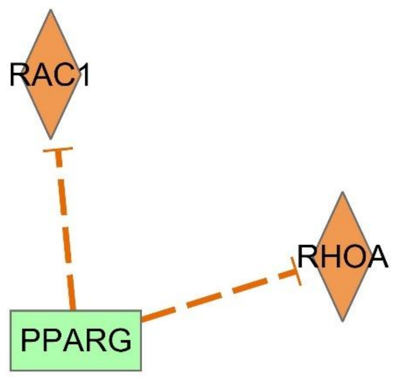

© 2000-2024 QIAGEN. All rights reserved.

Network 24

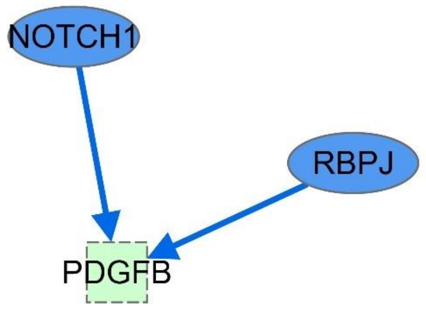

© 2000-2024 QIAGEN. All rights reserved.

Network 25

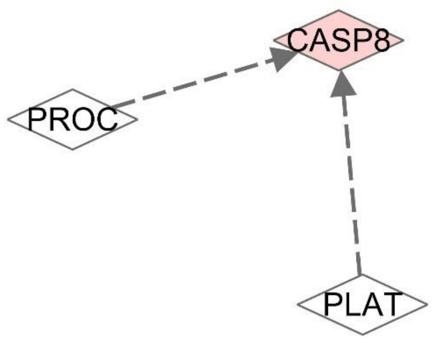

© 2000-2024 QIAGEN. All rights reserved.

b. EPEA vs control

Network 1

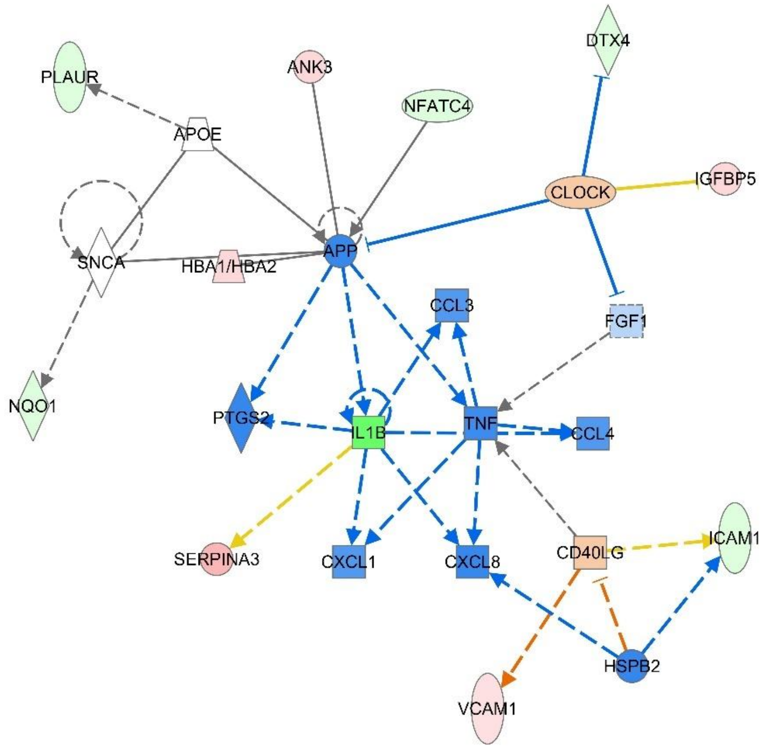

© 2000-2024 QIAGEN. All rights reserved.

Network 2

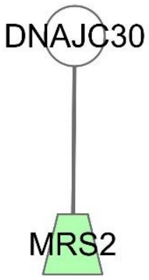

© 2000-2024 QIAGEN. All...

Network 3

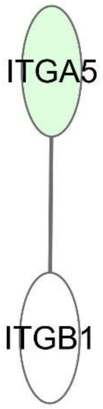

© 2000-2024 QIAGE...

Network 4

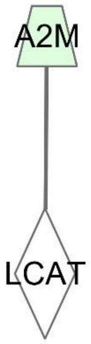

© 2000-2024 QIAG...

Network 5

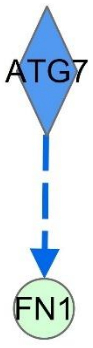

© 2000-2024 QIAG...

Network 6

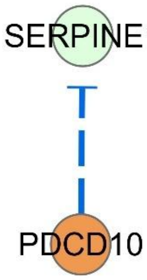

© 2000-2024 QIAGEN. All r...

c. DHEA vs control

Network 1

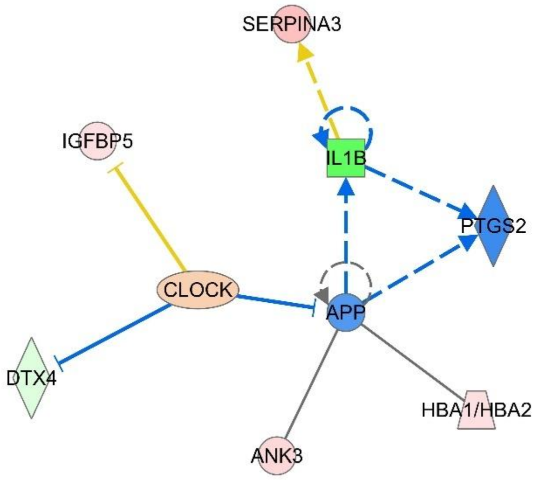

© 2000-2024 QIAGEN. All rights reserved.

Network 2

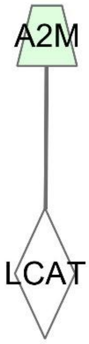

© 2000-2024 QIAG...

Network 3

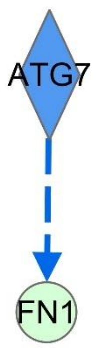

© 2000-2024 QIAG...

Network 4

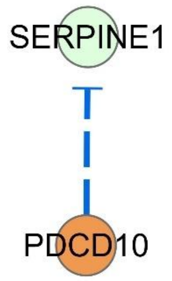

© 2000-2024 QIAGEN. All r...

Network 5

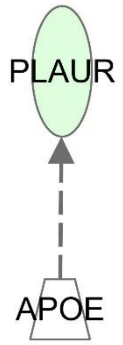

© 2000-2024 QIAGEN....

Network 6

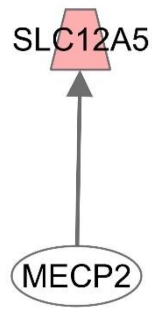

© 2000-2024 QIAGEN. Al...

Network 7

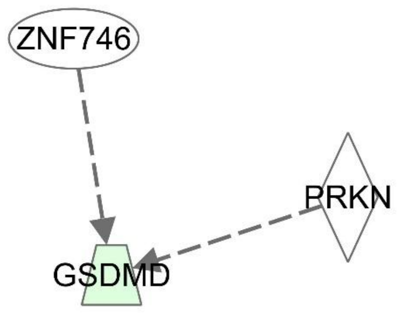

© 2000-2024 QIAGEN. All rights reserved.

Network 8

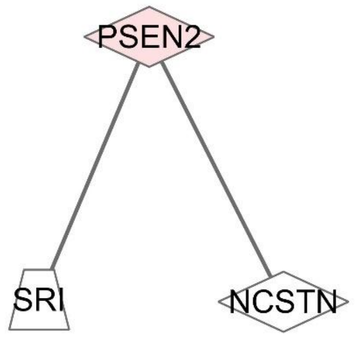

© 2000-2024 QIAGEN. All rights reserved.

d. DHEA+IL6 vs IL6

Network 1

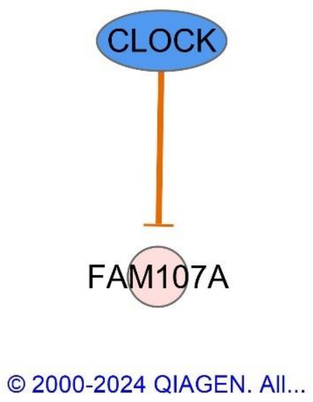

Network 2

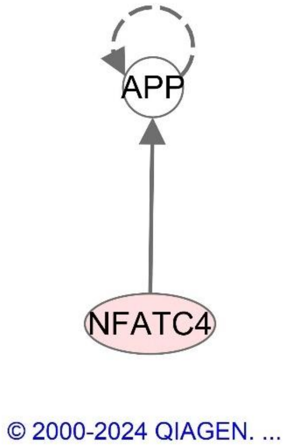

Network 3

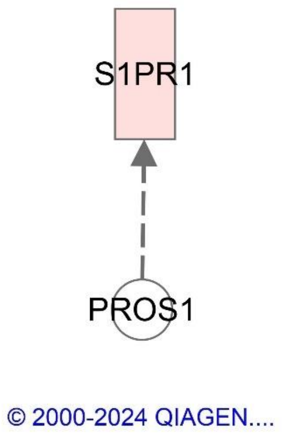

Network 4

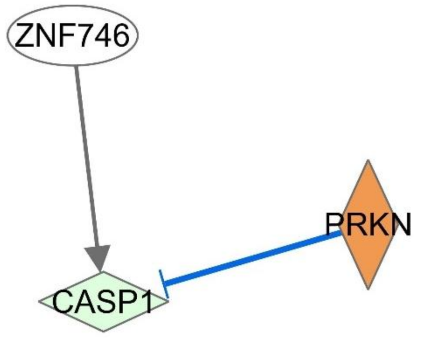

© 2000-2024 QIAGEN. All rights reserved.

e. Acetate vs control

Network 1

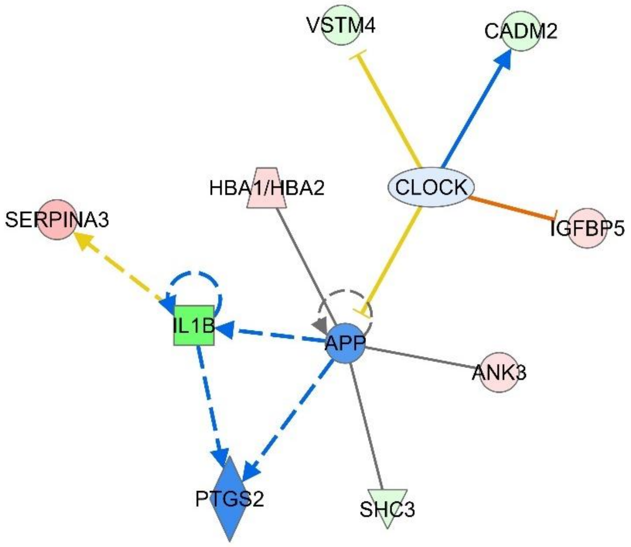

© 2000-2024 QIAGEN. All rights reserved.

Network 2

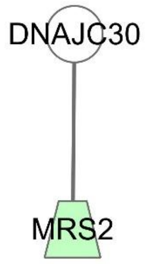

© 2000-2024 QIAGEN. All...

Network 3

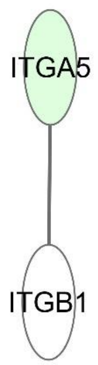

© 2000-2024 QIAGE...

Network 4

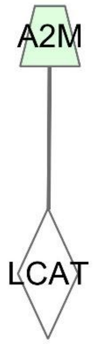

© 2000-2024 QIAG...

Network 5

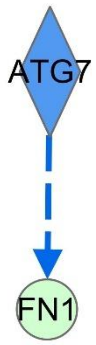

© 2000-2024 QIAG...

Network 6

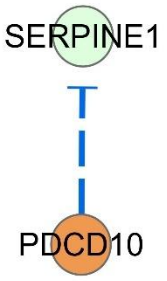

© 2000-2024 QIAGEN. All r...

Network 7

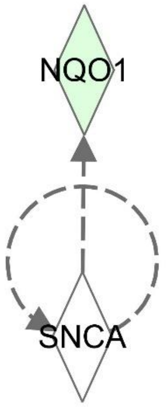

© 2000-2024 QIAGEN. Al...

Network 8

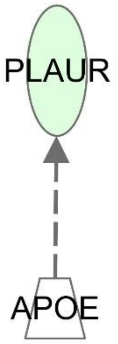

© 2000-2024 QIAGEN....

Network 9

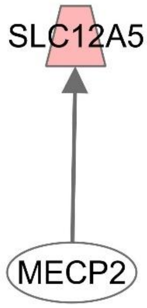

© 2000-2024 QIAGEN. Al...

Network 10

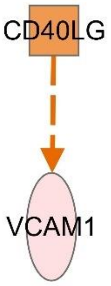

© 2000-2024 QIAGEN. ...

Network 11

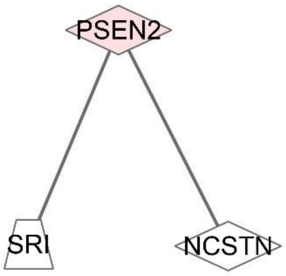

© 2000-2024 QIAGEN. All rights reserved.

f. Propionate vs control

Network 1

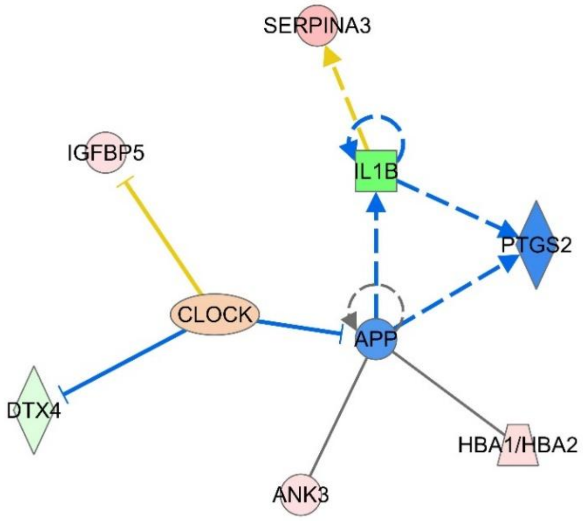

© 2000-2024 QIAGEN. All rights reserved.

Network 2

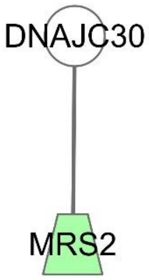

© 2000-2024 QIAGEN. All...

Network 3

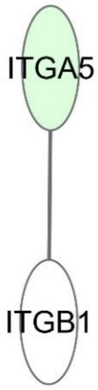

© 2000-2024 QIAGE...

Network 4

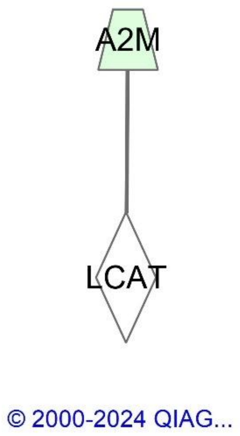

Network 5

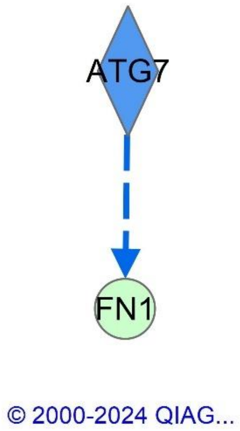

Network 6

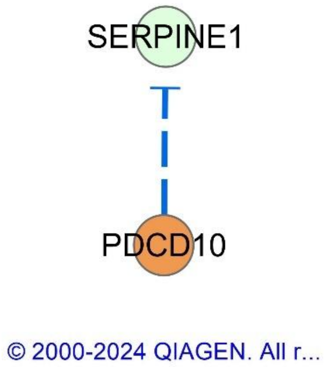

Network 7

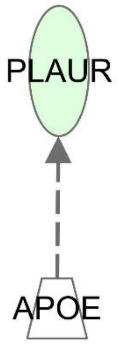

© 2000-2024 QIAGEN....

Network 8

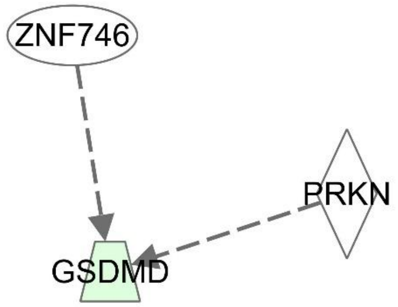

© 2000-2024 QIAGEN. All rights reserved.

g. Butyrate vs control

Network 1

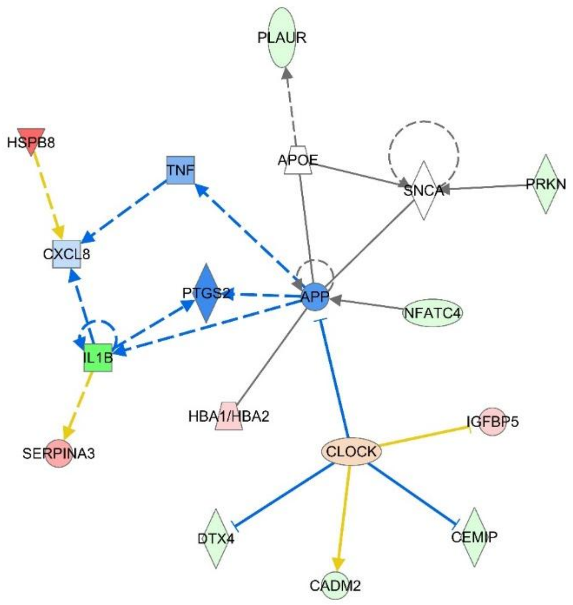

© 2000-2024 QIAGEN. All rights reserved.

Network 2

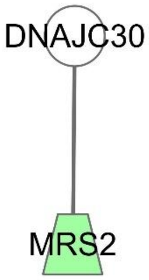

© 2000-2024 QIAGEN. All...

Network 3

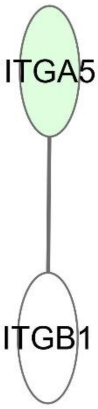

© 2000-2024 QIAGE...

Network 4

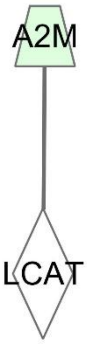

© 2000-2024 QIAG...

Network 5

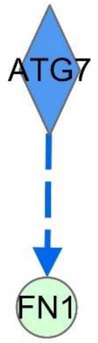

© 2000-2024 QIAG...

Network 6

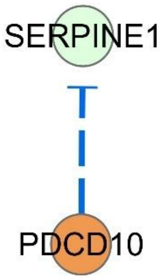

© 2000-2024 QIAGEN. All r...

Network 7

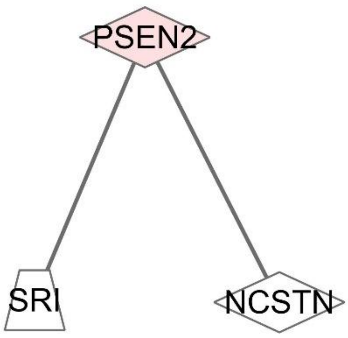

© 2000-2024 QIAGEN. All rights reserved.

Network 8

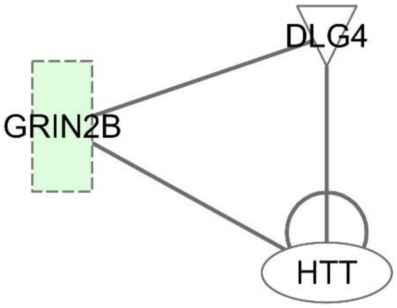

© 2000-2024 QIAGEN. All rights reserved.

Supplementary Figure 5. qPCR validation of candidate genes selected from the RNAseq analysis (a-e). The Independent Samples t Test was performed. Data are shown as mean±SEM; *p<0.05, **p<0.01, ***p<0.001, compared with vehicle treatment.

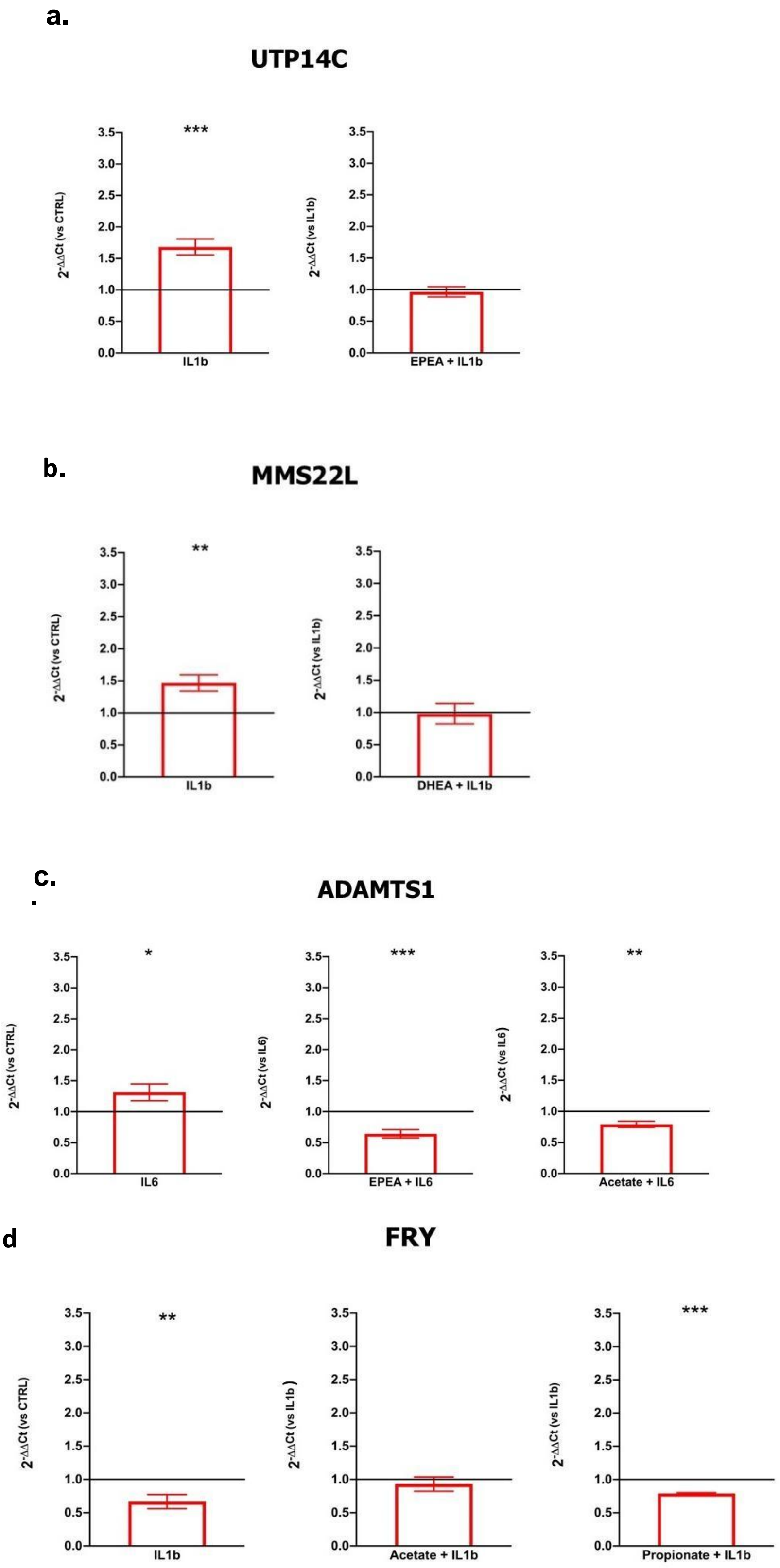

e.

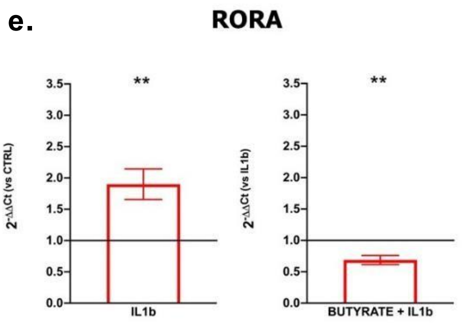
RORA
**
**
3.5
3.0
2.5
2.0
1.5
1.0
0.5
0.0
2^-ΔΔCt (vs CTRL)
2^-ΔΔCt (vs IL1b)
IL1b
BUTYRATE + IL1b

Supplementary Table 1: DEGs in IL1β vs Control conditions (log2FoldChange ≤ -0.59 or ≥ +0.59, adjusted p-value< 0.1)

Gene symbol	Gene name	log2FoldChange	padj
LY6E	lymphocyte antigen 6 family member E	2.6248273	3.52E-299
APOL6	apolipoprotein L6	4.246496751	2.58E-295
UBE2L6	ubiquitin conjugating enzyme E2 L6	3.316575436	7.35E-284
HLA-C	major histocompatibility complex; class I; C	2.923457771	2.33E-278
TAP1	transporter 1; ATP binding cassette subfamily B member	2.97037902	4.32E-277
SLC15A3	solute carrier family 15 member 3	3.834434759	1.16E-267
NT5E	5'-nucleotidase ecto	2.740244613	1.46E-266
C1R	complement C1r	3.437148706	3.84E-266
IFIT3	interferon induced protein with tetratricopeptide repeats 3	4.670907872	1.45E-265
SAMHD1	SAM and HD domain containing deoxynucleoside triphosphate triphosphohydrolase 1	2.817361828	4.14E-261
USP18	ubiquitin specific peptidase 18	3.908229882	1.07E-253
ZNFX1	zinc finger NFX1-type containing 1	2.263330881	8.55E-253
LGALS9	galectin 9	4.88775181	1.11E-251
WDFY1	WD repeat and FYVE domain containing 1	2.170458437	4.58E-232
BATF2	basic leucine zipper ATF-like transcription factor 2	5.375752571	1.51E-230
OPTN	optineurin	1.894750937	4.46E-229
LGMN	legumain	2.259005519	4.24E-224
PARP12	poly(ADP-ribose) polymerase family member 12	2.547283465	3.49E-222
HELZ2	helicase with zinc finger 2	3.47743185	1.12E-221
HLA-A	major histocompatibility complex; class I; A	2.541415341	1.01E-210
NFKBIZ	NFKB inhibitor zeta	3.818774239	3.44E-209
CHRNA1	cholinergic receptor nicotinic alpha 1 subunit	4.301561505	1.28E-207
RNF213	ring finger protein 213	3.338607155	3.43E-206
TNFAIP3	TNF alpha induced protein 3	4.04135031	1.29E-205
NFKBIA	NFKB inhibitor alpha	2.780833495	2.53E-203
DHX58	DExH-box helicase 58	5.813066605	1.89E-202
TAP2	transporter 2; ATP binding cassette subfamily B member	2.993430257	1.97E-202
BST2	bone marrow stromal cell antigen 2	3.581689926	1.64E-200
PSMB9	proteasome 20S subunit beta 9	3.720346427	4.24E-197
TRIM21	tripartite motif containing 21	2.721006131	1.91E-194

PML	PML nuclear body scaffold	2.403951851	1.22E-193
SAMD9	sterile alpha motif domain containing 9	3.425046607	1.07E-187
VCAM1	vascular cell adhesion molecule 1	4.981681838	1.37E-186
IFI27	interferon alpha inducible protein 27	4.320851718	1.51E-186
IFIT1	interferon induced protein with tetratricopeptide repeats 1	4.005294924	1.48E-180
APOL1	apolipoprotein L1	5.115487407	3.84E-180
PIK3AP1	phosphoinositide-3-kinase adaptor protein 1	4.641149602	4.11E-175
CD82	CD82 molecule	4.385645929	3.79E-171
RIGI	RNA sensor RIG-I	3.260252282	3.70E-167
ICAM1	intercellular adhesion molecule 1	4.253593418	6.31E-167
PLSCR1	phospholipid scramblase 1	2.649409525	1.45E-165
PARP10	poly(ADP-ribose) polymerase family member 10	2.669994826	3.13E-164
NMI	N-myc and STAT interactor	3.789732867	6.57E-164
SLC2A6	solute carrier family 2 member 6	1.824918673	1.13E-163
TLR3	toll like receptor 3	2.778222116	2.61E-163
RASGRP3	RAS guanyl releasing protein 3	3.628415052	5.87E-162
STAT1	signal transducer and activator of transcription 1	2.413922452	6.65E-162
DDX60	DExD/H-box helicase 60	2.737970875	2.70E-157
HERC6	HECT and RLD domain containing E3 ubiquitin protein ligase family member 6	3.602380825	2.41E-156
SP100	SP100 nuclear antigen	4.12413592	4.42E-155
IRF7	interferon regulatory factor 7	2.577913374	9.52E-155
IFI30	IFI30 lysosomal thiol reductase	4.010077183	2.98E-154
SLC39A14	solute carrier family 39 member 14	2.149525598	6.19E-154
UBA7	ubiquitin like modifier activating enzyme 7	2.827452204	7.58E-151
IL11	interleukin 11	4.122326384	1.15E-149
IFI44L	interferon induced protein 44 like	2.876051889	1.39E-149
EIF2AK2	eukaryotic translation initiation factor 2 alpha kinase 2	1.819668862	3.59E-146
THEMIS2	thymocyte selection associated family member 2	3.796185668	6.09E-146
DTX3L	deltex E3 ubiquitin ligase 3L	1.861711103	1.22E-144
NFKB1	nuclear factor kappa B subunit 1	2.215572551	3.38E-144
NFE2L3	NFE2 like bZIP transcription factor 3	3.114835735	5.92E-143
BTN3A2	butyrophilin subfamily 3 member A2	1.802892944	8.13E-142
B2M	beta-2-microglobulin	2.394430481	1.63E-140
ERAP1	endoplasmic reticulum aminopeptidase 1	1.891547853	3.39E-140
TAPBP	TAP binding protein	1.524912345	1.94E-139
VAT1	vesicle amine transport 1	-1.591129449	3.64E-139
ISG15	ISG15 ubiquitin like modifier	3.151332555	4.45E-138

IL18BP	interleukin 18 binding protein	3.814047136	2.34E-137
PLAAT4	phospholipase A and acyltransferase 4	2.728841867	2.32E-136
SGPP2	sphingosine-1-phosphate phosphatase 2	4.330919325	6.71E-134
SHFL	shiftless antiviral inhibitor of ribosomal frameshifting	2.835214993	7.90E-131
CEBPB	CCAAT enhancer binding protein beta	3.491937501	9.15E-130
IL32	interleukin 32	3.076120046	1.48E-129
MGLL	monoglyceride lipase	3.043396314	8.70E-129
SERPING1	serpin family G member 1	4.791358673	4.06E-128
CCL2	C-C motif chemokine ligand 2	2.005014239	1.92E-127
PSME2	proteasome activator subunit 2	1.854603041	4.37E-126
SECTM1	secreted and transmembrane 1	3.000396394	4.97E-126
HSPA1B	heat shock protein family A (Hsp70) member 1B	1.187680072	5.01E-122
TFPI2	tissue factor pathway inhibitor 2	2.395486034	6.34E-120
SP110	SP110 nuclear body protein	2.205439637	7.87E-120
HERC5	HECT and RLD domain containing E3 ubiquitin protein ligase 5	5.763387341	5.67E-118
SEMA5A	semaphorin 5A	-2.922592196	9.82E-117
BTN3A3	butyrophilin subfamily 3 member A3	2.182792004	1.27E-116
IER3	immediate early response 3	2.977726536	2.75E-116
APOL2	apolipoprotein L2	2.166714389	5.64E-115
IFITM3	interferon induced transmembrane protein 3	2.125348008	5.51E-113
NLRC5	NLR family CARD domain containing 5	2.30803282	1.58E-110
MMRN1	multimerin 1	-3.365284898	5.90E-109
CD38	CD38 molecule	3.097631867	2.50E-108
TRIM25	tripartite motif containing 25	1.389824889	3.09E-106
CDC25B	cell division cycle 25B	1.104367963	3.53E-106
IFITM1	interferon induced transmembrane protein 1	4.668826925	5.83E-106
TNIP1	TNFAIP3 interacting protein 1	1.585405104	8.62E-105
MFSD12	major facilitator superfamily domain containing 12	1.43154359	3.43E-104
ETV7	ETS variant transcription factor 7	4.465047938	1.19E-103
IFIT5	interferon induced protein with tetratricopeptide repeats 5	2.445927282	2.05E-103
RELB	RELB proto-oncogene; NF-kB subunit	2.089233401	2.76E-103
CRYAB	crystallin alpha B	-3.194427847	8.79E-103
PSMB8	proteasome 20S subunit beta 8	2.593471843	1.40E-100
TDRD7	tudor domain containing 7	3.28510112	2.10E-100
RSAD2	radical S-adenosyl methionine domain containing 2	5.991448726	5.76E-100
HLA-F	major histocompatibility complex; class I; F	4.218448722	7.92E-100

ZCCHC2	zinc finger CCHC-type containing 2	2.111546445	8.47E-100
LGALS3BP	galectin 3 binding protein	1.482014155	9.07E-100
TRIM22	tripartite motif containing 22	1.848229555	1.02E-99
GCH1	GTP cyclohydrolase 1	3.300214642	2.23E-99
UNC93B1	unc-93 homolog B1; TLR signaling regulator	1.782949216	1.36E-97
FBLN5	fibulin 5	2.507313201	4.08E-96
ZC3H12A	zinc finger CCCH-type containing 12A	3.632029488	4.82E-96
GMPR	guanosine monophosphate reductase	4.567402642	5.77E-96
SERPINE2	serpin family E member 2	2.249865233	4.52E-93
NAPA	NSF attachment protein alpha	1.291012508	2.11E-92
CASP1	caspase 1	3.421406816	1.74E-90
IFIH1	interferon induced with helicase C domain 1	4.234344577	4.64E-90
IRF1	interferon regulatory factor 1	2.795838431	1.08E-89
TRIL	TLR4 interactor with leucine rich repeats	2.774755648	1.69E-88
IFI44	interferon induced protein 44	2.755904429	7.56E-88
ERICH3	glutamate rich 3	3.881468885	1.06E-87
DRAM1	DNA damage regulated autophagy modulator 1	1.678605702	1.25E-87
GBP5	guanylate binding protein 5	5.067890279	3.77E-87
TAGLN	transgelin	-1.737049765	4.61E-87
IFI6	interferon alpha inducible protein 6	2.333267237	1.60E-86
BTN3A1	butyrophilin subfamily 3 member A1	1.967116505	1.45E-85
ADAR	adenosine deaminase RNA specific	1.289674275	7.58E-84
TYMP	thymidine phosphorylase	4.17127235	1.07E-83
CAV1	caveolin 1	1.321295607	6.02E-83
LIF	LIF interleukin 6 family cytokine	1.745279343	1.19E-82
PSME1	proteasome activator subunit 1	1.693934909	1.37E-82
STAT5A	signal transducer and activator of transcription 5A	4.278946018	1.98E-82
MX1	MX dynamin like GTPase 1	4.578171065	2.72E-82
IRAK2	interleukin 1 receptor associated kinase 2	2.911236223	2.53E-79
NT5DC2	5'-nucleotidase domain containing 2	-1.140902354	2.74E-78
OAS2	2'-5'-oligoadenylate synthetase 2	5.404271898	1.66E-77
ERAP2	endoplasmic reticulum aminopeptidase 2	1.895699346	2.13E-77
PRUNE2	prune homolog 2 with BCH domain	-1.833961828	4.53E-77
CP	ceruloplasmin	3.142696593	5.00E-77
FNDC4	fibronectin type III domain containing 4	-1.831007546	1.28E-76
IRF2	interferon regulatory factor 2	1.835308012	1.79E-76
CLDN1	claudin 1	2.157696964	1.87E-76
OAS1	2'-5'-oligoadenylate synthetase 1	4.7200441	2.20E-76

DDX60L	DExD/H-box 60 like	3.498438394	1.20E-75
CEBPD	CCAAT enhancer binding protein delta	1.900244393	6.89E-75
TNFAIP2	TNF alpha induced protein 2	5.18077087	2.11E-74
CCND1	cyclin D1	1.662188579	9.93E-74
DRD4	dopamine receptor D4	2.201700909	2.45E-73
PTGES	prostaglandin E synthase	3.195474533	9.55E-73
CMTR1	cap methyltransferase 1	1.050046579	1.67E-72
PATL1	PAT1 homolog 1; processing body mRNA decay factor	1.245036303	1.76E-72
ABAT	4-aminobutyrate aminotransferase	-1.454932115	1.95E-72
BID	BH3 interacting domain death agonist	1.753556284	3.86E-72
N4BP1	NEDD4 binding protein 1	1.64389209	5.19E-72
PIM2	Pim-2 proto-oncogene; serine/threonine kinase	1.490340734	1.07E-70
PTX3	pentraxin 3	2.381198524	2.92E-70
NFKB2	nuclear factor kappa B subunit 2	1.970265102	2.99E-70
ID3	inhibitor of DNA binding 3	-1.567753764	5.14E-70
HAS3	hyaluronan synthase 3	2.025513941	1.73E-69
JAK2	Janus kinase 2	3.05872031	1.86E-69
SPATS2L	spermatogenesis associated serine rich 2 like	1.351439577	6.25E-69
SPSB1	splA/ryanodine receptor domain and SOCS box containing 1	3.256079343	1.05E-68
MYC	MYC proto-oncogene; bHLH transcription factor	1.285291092	1.21E-68
SLC25A28	solute carrier family 25 member 28	1.890149574	4.61E-68
MAP6	microtubule associated protein 6	-1.396515654	4.95E-68
C6orf62	chromosome 6 open reading frame 62	0.850347519	2.38E-67
TRAF1	TNF receptor associated factor 1	3.303597396	8.20E-67
PSMA6	proteasome 20S subunit alpha 6	1.600016412	1.75E-66
TUBB2B	tubulin beta 2B class IIb	-0.721651339	1.86E-66
MAGED4	MAGE family member D4	-1.446356496	2.77E-66
AGTRAP	angiotensin II receptor associated protein	1.570108782	2.84E-66
CCL5	C-C motif chemokine ligand 5	5.436946162	3.43E-66
TENT5A	terminal nucleotidyltransferase 5A	2.100263046	7.22E-66
S1PR1	sphingosine-1-phosphate receptor 1	-2.834998507	3.15E-65
GFAP	glial fibrillary acidic protein	-1.324526764	1.05E-64
PDXK	pyridoxal kinase	1.122070416	2.59E-64
ACTA2	actin alpha 2; smooth muscle	-1.557016172	3.06E-64
ACTG1	actin gamma 1	-1.037235607	4.45E-64
GSDMD	gasdermin D	3.081525184	5.82E-64
ASPHD2	aspartate beta-hydroxylase domain containing 2	3.112468997	7.31E-64

NUB1	negative regulator of ubiquitin like proteins 1	1.756413608	7.39E-64
CYLD	CYLD lysine 63 deubiquitinase	1.481519974	7.63E-64
SLC30A1	solute carrier family 30 member 1	1.022221233	7.80E-64
CA2	carbonic anhydrase 2	1.874423106	1.91E-63
STAT2	signal transducer and activator of transcription 2	1.684607204	1.96E-63
FHL1	four and a half LIM domains 1	-1.260462831	1.21E-62
SEPTIN5	septin 5	-2.063474203	1.79E-62
IFITM2	interferon induced transmembrane protein 2	3.333057716	2.67E-62
NT5C3A	5'-nucleotidase; cytosolic IIIA	2.029771063	4.38E-62
TRIM14	tripartite motif containing 14	1.371326693	5.70E-62
NRP2	neuropilin 2	1.49188624	6.92E-62
PSMB10	proteasome 20S subunit beta 10	1.695447211	2.58E-61
ADORA2A	adenosine A2a receptor	3.803760333	3.38E-61
GABBR2	gamma-aminobutyric acid type B receptor subunit 2	-1.249227863	5.37E-61
CXCL16	C-X-C motif chemokine ligand 16	2.050961456	7.70E-61
CADM2	cell adhesion molecule 2	-2.036090019	9.23E-61
HSPH1	heat shock protein family H (Hsp110) member 1	0.797242278	1.03E-60
CSF1	colony stimulating factor 1	1.355187447	1.35E-60
OGFR	opioid growth factor receptor	1.210630581	1.72E-60
PTK2B	protein tyrosine kinase 2 beta	1.50677638	2.78E-60
GFPT2	glutamine-fructose-6-phosphate transaminase 2	1.063337495	4.74E-60
SP140L	SP140 nuclear body protein like	3.569303511	6.00E-60
SLFN5	schlafen family member 5	5.032351058	6.37E-60
TTC3	tetratricopeptide repeat domain 3	-1.072874466	7.87E-60
ADAP1	ArfGAP with dual PH domains 1	4.227896277	9.57E-60
CHI3L2	chitinase 3 like 2	3.080379483	1.04E-59
TRIM26	tripartite motif containing 26	1.055681933	1.51E-59
JUNB	JunB proto-oncogene; AP-1 transcription factor subunit	1.884012613	4.23E-59
NNAT	neuronatin	-1.939201361	4.75E-59
CMPK2	cytidine/uridine monophosphate kinase 2	5.244129035	1.98E-58
IL15RA	interleukin 15 receptor subunit alpha	3.417134796	6.64E-58
MOB3C	MOB kinase activator 3C	1.996719755	1.46E-57
RND2	Rho family GTPase 2	-1.427984023	1.47E-57
CERK	ceramide kinase	-1.194108613	2.73E-57
CTSC	cathepsin C	1.183886346	3.44E-57
ATP6V0E2	ATPase H+ transporting V0 subunit e2	-0.977185235	7.20E-57
TNFRSF14	TNF receptor superfamily member 14	3.834971528	9.04E-57

RPE65	retinoid isomerohydrolase RPE65	-2.721158454	2.15E-56
MLC1	modulator of VRAC current 1	-1.380022155	2.92E-56
PDGFRL	platelet derived growth factor receptor like	2.766680752	1.26E-55
B4GALT1	beta-1;4-galactosyltransferase 1	1.368746568	6.60E-55
CEACAM1	CEA cell adhesion molecule 1	3.580382638	9.21E-55
RABGAP1L	RAB GTPase activating protein 1 like	3.219798281	9.39E-55
POLR3G	RNA polymerase III subunit G	1.513342613	1.10E-54
C1S	complement C1s	4.376426057	1.37E-54
ESR1	estrogen receptor 1	1.45532513	1.92E-54
C1QTNF1	C1q and TNF related 1	1.713956799	2.09E-54
NAMPT	nicotinamide phosphoribosyltransferase	1.942174043	3.59E-54
PSMA2	proteasome 20S subunit alpha 2	1.209156867	6.42E-54
CNTN2	contactin 2	2.699382535	1.09E-53
GNB4	G protein subunit beta 4	0.849589842	1.46E-53
UXS1	UDP-glucuronate decarboxylase 1	1.166434783	4.72E-53
ACO1	aconitase 1	-1.399425879	4.78E-53
BHLHE41	basic helix-loop-helix family member e41	3.048246327	6.32E-53
SCARB2	scavenger receptor class B member 2	1.039545648	6.58E-53
FN1	fibronectin 1	1.44405919	9.48E-53
DLL4	delta like canonical Notch ligand 4	4.541617328	9.49E-53
ABTB2	ankyrin repeat and BTB domain containing 2	1.520477221	1.48E-52
APOBEC3G	apolipoprotein B mRNA editing enzyme catalytic subunit 3G	6.512053888	1.78E-52
HLA-K	major histocompatibility complex; class I; K (pseudogene)	2.572467734	3.00E-52
RHOU	ras homolog family member U	2.066516316	3.69E-52
PIK3CD	phosphatidylinositol-4;5-bisphosphate 3-kinase catalytic subunit delta	1.674991252	7.46E-52
M6PR	mannose-6-phosphate receptor; cation dependent	0.836730309	8.45E-52
PEA15	proliferation and apoptosis adaptor protein 15	-0.707837386	1.70E-51
ECE1	endothelin converting enzyme 1	1.284976443	1.95E-51
WTAP	WT1 associated protein	1.470705817	7.02E-51
PPM1K	protein phosphatase; Mg2+/Mn2+ dependent 1K	2.385372655	8.24E-51
PBX1	PBX homeobox 1	-1.291387325	1.23E-50
TGFB3	transforming growth factor beta 3	1.434122031	1.24E-50
GACAT2	gastric cancer associated transcript 2	-1.78696775	1.85E-50
GBP2	guanylate binding protein 2	5.228192802	1.94E-50
SBNO2	strawberry notch homolog 2	1.07785742	2.80E-50
GLRX	glutaredoxin	1.65386574	3.92E-50
TRANK1	tetratricopeptide repeat and ankyrin repeat containing 1	5.547196176	6.27E-50

CCN2	cellular communication network factor 2	-2.193244415	1.80E-49
TNFRSF1B	TNF receptor superfamily member 1B	3.806482825	1.85E-49
CSRP1	cysteine and glycine rich protein 1	-1.250064123	2.05E-49
DNAJA1	DnaJ heat shock protein family (Hsp40) member A1	0.830363567	4.90E-49
LINC01963	long intergenic non-protein coding RNA 1963	-1.721854416	7.45E-49
MX2	MX dynamin like GTPase 2	6.407787489	8.57E-49
CYP2J2	cytochrome P450 family 2 subfamily J member 2	3.810863729	1.66E-48
TOX	thymocyte selection associated high mobility group box	-1.223229807	1.95E-48
SLC12A7	solute carrier family 12 member 7	1.212494861	2.06E-48
LINC00662	long intergenic non-protein coding RNA 662	-1.068982949	2.56E-48
NEURL3	neuralized E3 ubiquitin protein ligase 3	4.333588344	2.79E-48
GRWD1	glutamate rich WD repeat containing 1	0.964331982	3.42E-48
TMEM62	transmembrane protein 62	1.804955498	4.32E-48
THY1	Thy-1 cell surface antigen	-1.361140604	4.82E-48
AK1	adenylate kinase 1	-1.084781262	7.70E-48
RNF19B	ring finger protein 19B	1.061428924	1.24E-47
GTPBP1	GTP binding protein 1	1.13054093	1.34E-47
JADE2	jade family PHD finger 2	1.998017153	2.10E-47
H2BC12	H2B clustered histone 12	1.629806141	3.45E-47
OAF	out at first homolog	1.504692949	6.61E-47
HCP5	HLA complex P5	2.492515922	6.73E-47
SMAD9	SMAD family member 9	-2.799343557	3.62E-46
NDRG4	NDRG family member 4	-1.047551989	4.08E-46
MASTL	microtubule associated serine/threonine kinase like	1.166097862	4.53E-46
TMEM140	transmembrane protein 140	5.007417464	4.53E-46
TRIM69	tripartite motif containing 69	1.518932871	8.31E-46
HSP90AA1	heat shock protein 90 alpha family class A member 1	0.767851342	9.44E-46
RNF114	ring finger protein 114	1.391387715	1.38E-45
TPM4	tropomyosin 4	-0.681494659	2.02E-45
NUDT3	nudix hydrolase 3	-0.892980857	2.70E-45
TIMP3	TIMP metallopeptidase inhibitor 3	-1.076481826	3.04E-45
ATP9A	ATPase phospholipid transporting 9A (putative)	-1.284031656	3.66E-45
ZC3HAV1	zinc finger CCCH-type containing; antiviral 1	0.840224232	4.31E-45
CFB	complement factor B	5.973873527	4.55E-45
CHST10	carbohydrate sulfotransferase 10	-0.95844809	6.39E-45
PLEKHA4	pleckstrin homology domain containing A4	1.149481251	6.64E-45

PIANP	PILR alpha associated neural protein	-1.825331674	7.03E-45
PARP9	poly(ADP-ribose) polymerase family member 9	1.994480921	7.85E-45
GNG12	G protein subunit gamma 12	-1.009666443	9.88E-45
SNN	stannin	-1.192770838	1.87E-44
SLC29A1	solute carrier family 29 member 1 (Augustine blood group)	0.961389276	3.27E-44
PDLIM1	PDZ and LIM domain 1	1.023575774	6.10E-44
PLCD3	phospholipase C delta 3	-0.983955698	6.38E-44
PHF11	PHD finger protein 11	1.973133848	7.82E-44
STAP2	signal transducing adaptor family member 2	3.271521938	8.71E-44
MMP7	matrix metallopeptidase 7	4.266330978	1.11E-43
ARHGEF6	Rac/Cdc42 guanine nucleotide exchange factor 6	-1.091113811	1.27E-43
SLC25A19	solute carrier family 25 member 19	1.319839388	1.43E-43
IL4I1	interleukin 4 induced 1	3.991902211	2.16E-43
GRB14	growth factor receptor bound protein 14	-1.707506281	2.25E-43
PELI2	pellino E3 ubiquitin protein ligase family member 2	-1.954894138	2.39E-43
CRIP2	cysteine rich protein 2	-0.803460657	2.82E-43
FOLR1	folate receptor alpha	-1.675216052	2.93E-43
SOCS1	suppressor of cytokine signaling 1	3.576451916	3.75E-43
DRAP1	DR1 associated protein 1	1.10574587	5.63E-43
UCP2	uncoupling protein 2	-1.278440835	9.50E-43
ARHGAP29	Rho GTPase activating protein 29	1.026482849	1.07E-42
RGS16	regulator of G protein signaling 16	1.930480063	1.11E-42
BLZF1	basic leucine zipper nuclear factor 1	1.442633335	2.19E-42
PCDH8	protocadherin 8	3.043354966	3.02E-42
OASL	2'-5'-oligoadenylate synthetase like	6.747099892	5.93E-42
IDO1	indoleamine 2;3-dioxygenase 1	5.073762652	8.11E-42
TOP1	DNA topoisomerase I	0.753836325	1.04E-41
DBN1	drebrin 1	-1.082821313	1.28E-41
IKBKE	inhibitor of nuclear factor kappa B kinase subunit epsilon	3.438837952	1.50E-41
PHKB	phosphorylase kinase regulatory subunit beta	-1.060726949	1.82E-41
ACTB	actin beta	-0.690793567	2.55E-41
CST3	cystatin C	0.861838779	2.72E-41
TNFRSF10D	TNF receptor superfamily member 10d	-2.064627553	4.89E-41
TLL2	tolloid like 2	2.773906981	8.96E-41
PARP4	poly(ADP-ribose) polymerase family member 4	0.753766847	1.33E-40
OTUD4	OTU deubiquitinase 4	0.970552301	1.62E-40
SLC43A2	solute carrier family 43 member 2	1.262818198	1.62E-40

SLC39A10	solute carrier family 39 member 10	-1.076741243	2.23E-40
SCRN1	secernin 1	-0.77942331	3.47E-40
ADAM9	ADAM metallopeptidase domain 9	-1.100626498	4.80E-40
DNPEP	aspartyl aminopeptidase	0.971357483	5.03E-40
USP15	ubiquitin specific peptidase 15	1.500110932	6.24E-40
ADGRE5	adhesion G protein-coupled receptor E5	0.918214229	8.68E-40
TUBA1A	tubulin alpha 1a	-0.978998993	8.75E-40
TNFSF10	TNF superfamily member 10	6.530223005	1.03E-39
APOBEC3B	apolipoprotein B mRNA editing enzyme catalytic subunit 3B	1.272411455	1.24E-39
MYCN	MYCN proto-oncogene; bHLH transcription factor	-1.68374673	1.64E-39
NOCT	nocturnin	1.144632044	1.68E-39
ARF3	ADP ribosylation factor 3	0.611195119	1.82E-39
RTN3	reticulon 3	-0.67958438	1.87E-39
TPM1	tropomyosin 1	-1.218323347	2.75E-39
KCNH2	potassium voltage-gated channel subfamily H member 2	-1.688663216	2.82E-39
RTP4	receptor transporter protein 4	7.030798987	2.94E-39
SERPINB8	serpin family B member 8	1.558205835	2.97E-39
HAPLN3	hyaluronan and proteoglycan link protein 3	2.186521958	3.23E-39
SQOR	sulfide quinone oxidoreductase	5.413166632	4.51E-39
ZNF106	zinc finger protein 106	-1.059498013	4.74E-39
ENHO	energy homeostasis associated	-1.838811559	5.28E-39
TUBB3	tubulin beta 3 class III	-0.738465737	6.02E-39
NAMPTP1	nicotinamide phosphoribosyltransferase pseudogene 1	1.894068623	6.02E-39
ARB2A	ARB2 cotranscriptional regulator A	-1.243181757	8.38E-39
H1-0	H1,0 linker histone	2.199327958	8.38E-39
MDM2	MDM2 proto-oncogene	0.798930179	9.59E-39
OSMR	oncostatin M receptor	0.749905403	1.29E-38
CD274	CD274 molecule	3.842222058	1.47E-38
CBR3	carbonyl reductase 3	1.947354376	1.68E-38
FGFBP3	fibroblast growth factor binding protein 3	-0.970263387	1.72E-38
PDLIM7	PDZ and LIM domain 7	-1.101279274	1.98E-38
LNPEP	leucyl and cystinyl aminopeptidase	1.30035326	2.75E-38
LOC728392	uncharacterized LOC728392	-1.144684113	2.93E-38
NR1D1	nuclear receptor subfamily 1 group D member 1	1.112893508	3.49E-38
ETS2	ETS proto-oncogene 2; transcription factor	2.19987917	3.64E-38
RASSF2	Ras association domain family member 2	-1.095362149	3.93E-38
RTN1	reticulon 1	-1.746151103	4.07E-38

SSTR2	somatostatin receptor 2	5.700609862	5.36E-38
TGFB1I1	transforming growth factor beta 1 induced transcript 1	-1.422845751	9.49E-38
PROM1	prominin 1	-0.959460359	9.97E-38
ISG20	interferon stimulated exonuclease gene 20	5.634502474	1.05E-37
MOV10	Mov10 RISC complex RNA helicase	1.246322224	1.15E-37
GRIK3	glutamate ionotropic receptor kainate type subunit 3	-2.104038563	2.25E-37
ARL4D	ADP ribosylation factor like GTPase 4D	-1.8947217	2.76E-37
PTPRF	protein tyrosine phosphatase receptor type F	-0.874654775	3.15E-37
DCLK2	doublecortin like kinase 2	-1.271071301	3.85E-37
GBP3	guanylate binding protein 3	3.902031032	5.37E-37
NBN	nibrin	0.832673134	6.52E-37
SDAD1	SDA1 domain containing 1	0.753602789	7.88E-37
TNFSF13B	TNF superfamily member 13b	4.511446574	1.04E-36
RHOC	ras homolog family member C	-0.867307965	1.38E-36
KLF9	KLF transcription factor 9	1.734085651	1.60E-36
ACP2	acid phosphatase 2; lysosomal	0.891307549	1.84E-36
ITM2C	integral membrane protein 2C	-1.166788259	1.99E-36
MAPK4	mitogen-activated protein kinase 4	-1.954909075	2.14E-36
UTP6	UTP6 small subunit processome component	0.935611046	2.40E-36
TAPBPL	TAP binding protein like	3.114350785	2.87E-36
NCOA7	nuclear receptor coactivator 7	2.102802806	3.81E-36
CD164	CD164 molecule	0.69361768	3.92E-36
ZNF710-AS1	ZNF710 antisense RNA 1	-2.318457904	4.14E-36
APC2	APC regulator of WNT signaling pathway 2	-1.645790524	4.18E-36
KIAA1549	KIAA1549	-1.085912201	4.30E-36
CXorf38	chromosome X open reading frame 38	0.994950745	4.89E-36
C15orf62	chromosome 15 open reading frame 62	2.981864928	6.02E-36
CD68	CD68 molecule	5.000939985	6.89E-36
PCBP4	poly(rC) binding protein 4	-1.251267945	9.59E-36
TAF4B	TATA-box binding protein associated factor 4b	1.436314325	9.59E-36
SCIN	scinderin	3.786976695	1.02E-35
MT2A	metallothionein 2A	3.863244442	1.59E-35
MMP24OS	MMP24 opposite strand	-1.160928681	1.89E-35
ADPRS	ADP-ribosylserine hydrolase	1.04885885	1.95E-35
NNMT	nicotinamide N-methyltransferase	2.452257227	1.97E-35
MMP15	matrix metallopeptidase 15	-1.512194447	3.09E-35
HK2	hexokinase 2	0.99398648	3.86E-35
PRICKLE2	prickle planar cell polarity protein 2	-1.190044244	4.33E-35

LAMP3	lysosomal associated membrane protein 3	5.086119015	4.75E-35
YBX3	Y-box binding protein 3	0.813211607	5.92E-35
NPIPB9	nuclear pore complex interacting protein family member B9	19.11236576	7.98E-35
CDO1	cysteine dioxygenase type 1	-1.462585318	9.34E-35
LOX	lysyl oxidase	1.035643945	9.49E-35
AIF1L	allograft inflammatory factor 1 like	-1.25437934	9.76E-35
SFRP4	secreted frizzled related protein 4	-1.294568001	1.05E-34
ICOSLG	inducible T cell costimulator ligand	3.02004914	1.19E-34
GUCY1A2	guanylate cyclase 1 soluble subunit alpha 2	2.164510641	1.37E-34
PLEKHB1	pleckstrin homology domain containing B1	-1.123196371	1.56E-34
ABCA4	ATP binding cassette subfamily A member 4	4.164465785	2.76E-34
SUZ12	SUZ12 polycomb repressive complex 2 subunit	0.746358205	3.35E-34
SLIT2	slit guidance ligand 2	-1.397228106	3.67E-34
EPSTI1	epithelial stromal interaction 1	4.825822382	3.69E-34
MYD88	MYD88 innate immune signal transduction adaptor	1.008830105	4.29E-34
CHMP5	charged multivesicular body protein 5	1.198427054	6.52E-34
ATP1B1	ATPase Na+/K+ transporting subunit beta 1	-1.201510326	7.11E-34
SOX4	SRY-box transcription factor 4	-1.014342639	1.12E-33
LOC1027239	ICOS ligand	3.747279241	1.43E-33
NACAD	NAC alpha domain containing	-1.16769471	1.45E-33
IL17RD	interleukin 17 receptor D	-1.51050942	1.48E-33
SCRG1	stimulator of chondrogenesis 1	-2.231405854	1.49E-33
RBCK1	RANBP2-type and C3HC4-type zinc finger containing 1	0.971818497	1.68E-33
DCBLD1	discoidin; CUB and LCCL domain containing 1	1.083950183	1.85E-33
MT1X	metallothionein 1X	1.538118814	1.93E-33
SLC18A2	solute carrier family 18 member A2	6.07527571	2.37E-33
PSMA3	proteasome 20S subunit alpha 3	1.031738775	3.82E-33
PITPNM3	PITPNM family member 3	-1.683276291	4.26E-33
HES4	hes family bHLH transcription factor 4	1.169220368	4.74E-33
CADM4	cell adhesion molecule 4	-0.813953894	5.10E-33
BCL3	BCL3 transcription coactivator	1.61633975	5.17E-33
CHPT1	choline phosphotransferase 1	0.869347253	6.03E-33
ELF1	E74 like ETS transcription factor 1	1.287909397	6.55E-33
MAP3K8	mitogen-activated protein kinase kinase kinase 8	1.518182959	8.32E-33

LBH	LBH regulator of WNT signaling pathway	-1.485716999	8.96E-33
DCP1A	decapping mRNA 1A	0.947760311	9.61E-33
CSAG3	CSAG family member 3	6.332995768	1.03E-32
MVP	major vault protein	1.136768349	1.09E-32
CCDC167	coiled-coil domain containing 167	-1.331384654	1.14E-32
MXRA8	matrix remodeling associated 8	-1.927900093	1.33E-32
CCND3	cyclin D3	0.906346584	1.80E-32
GDPD5	glycerophosphodiester phosphodiesterase domain containing 5	-2.182010426	1.97E-32
TIMP1	TIMP metallopeptidase inhibitor 1	1.303519982	2.07E-32
KAT7	lysine acetyltransferase 7	-1.221920409	2.15E-32
PHYHIPL	phytanoyl-CoA 2-hydroxylase interacting protein like	-1.573866303	3.03E-32
SLC18B1	solute carrier family 18 member B1	1.176647325	4.07E-32
SCARA3	scavenger receptor class A member 3	-0.777804104	4.52E-32
SMCR8	SMCR8-C9orf72 complex subunit	0.770370514	4.69E-32
CCL20	C-C motif chemokine ligand 20	6.943830023	5.31E-32
MAF	MAF bZIP transcription factor	-1.009058985	5.81E-32
STOM	stomatin	1.079243829	5.90E-32
METRN	meteorin; glial cell differentiation regulator	-1.054786776	6.15E-32
ADD3	adducin 3	-1.057811115	7.04E-32
PIM3	Pim-3 proto-oncogene; serine/threonine kinase	0.964373476	7.04E-32
KIAA1217	KIAA1217	0.940017872	8.16E-32
DCAKD	dephospho-CoA kinase domain containing	-0.831941339	8.91E-32
MRPL39	mitochondrial ribosomal protein L39	1.243877773	9.02E-32
SEPTIN11	septin 11	-0.820515072	9.82E-32
WBP1	WW domain binding protein 1	-1.409467804	1.09E-31
DDX21	DExD-box helicase 21	0.738799323	1.32E-31
SLC7A5	solute carrier family 7 member 5	1.167768575	1.38E-31
GJD3	gap junction protein delta 3	4.900386674	1.47E-31
SEPTIN3	septin 3	-2.038903899	1.94E-31
MAPK3	mitogen-activated protein kinase 3	-0.914376603	3.18E-31
RGS2	regulator of G protein signaling 2	2.021663961	3.39E-31
ZMYND15	zinc finger MYND-type containing 15	3.244892256	3.50E-31
TRAFD1	TRAF-type zinc finger domain containing 1	0.755003409	4.59E-31
FSCN1	fascin actin-bundling protein 1	-1.018545228	4.86E-31
MYLK	myosin light chain kinase	-1.462031582	5.89E-31
TMOD2	tropomodulin 2	-1.156537317	6.03E-31
ZFP36L2	ZFP36 ring finger protein like 2	0.687374113	6.16E-31
CA8	carbonic anhydrase 8	-1.566233122	6.25E-31

IRF9	interferon regulatory factor 9	1.209808278	6.44E-31
CRTAC1	cartilage acidic protein 1	4.909175055	6.97E-31
TMEM98	transmembrane protein 98	-1.428572543	7.53E-31
TMT1A	thiol methyltransferase 1A	-1.286513667	9.03E-31
PDE1C	phosphodiesterase 1C	-1.368103265	9.30E-31
LRATD1	LRAT domain containing 1	1.682098431	1.73E-30
TRIM38	tripartite motif containing 38	3.965688508	1.79E-30
GPC2	glypican 2	-1.279027988	1.99E-30
PPA1	inorganic pyrophosphatase 1	0.981093427	2.07E-30
DDAH2	DDAH family member 2; ADMA-independent	-1.010546808	2.12E-30
NEXN	nexilin F-actin binding protein	1.434785188	3.17E-30
SCG2	secretogranin II	-1.557821987	3.82E-30
NUP205	nucleoporin 205	0.608842134	4.03E-30
GNG2	G protein subunit gamma 2	-1.358806707	4.50E-30
HM13	histocompatibility minor 13	0.611561694	5.57E-30
TNFRSF8	TNF receptor superfamily member 8	2.095927107	5.68E-30
TRIM34	tripartite motif containing 34	1.988547888	5.95E-30
SMCHD1	structural maintenance of chromosomes flexible hinge domain containing 1	0.760198364	5.95E-30
ELMOD2	ELMO domain containing 2	-1.102298377	6.47E-30
CHST11	carbohydrate sulfotransferase 11	1.081115222	7.15E-30
NCAM1	neural cell adhesion molecule 1	-1.445649875	8.02E-30
B3GAT1	beta-1;3-glucuronyltransferase 1	-1.584001741	8.45E-30
EPHA5	EPH receptor A5	-3.782320304	9.70E-30
FGFR3	fibroblast growth factor receptor 3	-1.365285018	1.01E-29
PRR11	proline rich 11	-0.807582557	1.36E-29
RPIA	ribose 5-phosphate isomerase A	1.077503323	1.49E-29
B3GAT2	beta-1;3-glucuronyltransferase 2	-2.274710186	1.50E-29
PRKD2	protein kinase D2	1.236918599	1.63E-29
LGALS8	galectin 8	1.45362395	1.68E-29
EHD4	EH domain containing 4	1.0300708	1.73E-29
AEN	apoptosis enhancing nuclease	0.717656988	1.74E-29
FNBP1L	formin binding protein 1 like	-0.756139117	2.04E-29
ITGB3	integrin subunit beta 3	1.127409552	2.49E-29
ZNF267	zinc finger protein 267	1.566062249	2.90E-29
ADGRG1	adhesion G protein-coupled receptor G1	-1.189278609	2.94E-29
ST3GAL4	ST3 beta-galactoside alpha-2;3-sialyltransferase 4	1.206648668	3.14E-29
ALDH6A1	aldehyde dehydrogenase 6 family member A1	-1.148659793	3.38E-29
BAZ1A	bromodomain adjacent to zinc finger domain 1A	0.766048541	3.70E-29
GCLM	glutamate-cysteine ligase modifier subunit	1.668034642	3.91E-29

CITED2	Cbp/p300 interacting transactivator with Glu/Asp rich carboxy-terminal domain 2	-1.111009094	4.58E-29
LRP8	LDL receptor related protein 8	0.936227556	4.80E-29
NADK	NAD kinase	0.652006646	5.20E-29
GRIA1	glutamate ionotropic receptor AMPA type subunit 1	-0.845502349	5.22E-29
TMEM35A	transmembrane protein 35A	-2.282926793	5.28E-29
SEPTIN8	septin 8	-0.833644865	6.59E-29
LOC1027244	salt inducible kinase 1B (putative)	1.090110632	6.75E-29
SUSD6	sushi domain containing 6	1.093980011	7.54E-29
NOS2	nitric oxide synthase 2	2.49093085	8.42E-29
SEPTIN4	septin 4	1.787752622	8.73E-29
B4GALT5	beta-1;4-galactosyltransferase 5	0.728267923	9.40E-29
CFLAR	CASP8 and FADD like apoptosis regulator	1.454921322	1.00E-28
SORL1	sortilin related receptor 1	-1.7370427	1.06E-28
EHD2	EH domain containing 2	-1.002144078	1.07E-28
GRHL3	grainyhead like transcription factor 3	-1.803385887	1.40E-28
TFAP2C	transcription factor AP-2 gamma	1.319875014	1.43E-28
PRR5L	proline rich 5 like	-1.153759509	1.51E-28
HSPD1	heat shock protein family D (Hsp60) member 1	0.723512515	1.67E-28
DUSP5	dual specificity phosphatase 5	1.648065187	1.68E-28
SLC35F1	solute carrier family 35 member F1	-0.831122994	2.21E-28
FERMT1	FERM domain containing kindlin 1	0.926982672	2.84E-28
NUDCD1	NudC domain containing 1	1.1622832	2.99E-28
CD24	CD24 molecule	-1.393647623	3.10E-28
BAALC	BAALC binder of MAP3K1 and KLF4	-1.780502701	3.24E-28
HSP90B1	heat shock protein 90 beta family member 1	0.655191823	3.26E-28
EFEMP2	EGF containing fibulin extracellular matrix protein 2	-0.858967	3.27E-28
SUMF1	sulfatase modifying factor 1	-1.074087999	3.43E-28
TCIM	transcriptional and immune response regulator	3.894926934	3.76E-28
HAS2	hyaluronan synthase 2	2.144163377	3.78E-28
ARL3	ADP ribosylation factor like GTPase 3	-1.004240462	3.81E-28
ABCF2	ATP binding cassette subfamily F member 2	0.676051426	3.97E-28
PSMB8-AS1	PSMB8 antisense RNA 1 (head to head)	3.000918802	3.98E-28
PDLIM4	PDZ and LIM domain 4	0.855132722	4.10E-28
F2RL1	F2R like trypsin receptor 1	0.793054719	4.14E-28
SELENOI	selenoprotein I	0.622407283	4.19E-28
FAM171A2	family with sequence similarity 171 member A2	-1.45437554	4.30E-28
NOC3L	NOC3 like DNA replication regulator	0.855005013	4.30E-28

FKBP4	FKBP prolyl isomerase 4	0.662074773	4.38E-28
SPRY4	sprouty RTK signaling antagonist 4	1.038341211	4.69E-28
XYLT1	xylosyltransferase 1	-1.091491099	5.10E-28
CCND2	cyclin D2	-0.761279638	5.11E-28
ZMAT3	zinc finger matrin-type 3	-0.923204802	5.60E-28
SEL1L3	SEL1L family member 3	-0.943987861	6.20E-28
NOP14	NOP14 nucleolar protein	0.754210851	7.19E-28
PMAIP1	phorbol-12-myristate-13-acetate-induced protein 1	1.48732694	7.19E-28
DANCR	differentiation antagonizing non-protein coding RNA	-1.945547654	7.34E-28
ZNF618	zinc finger protein 618	0.96710731	7.34E-28
FOSL1	FOS like 1; AP-1 transcription factor subunit	1.1054729	8.93E-28
XAF1	XIAP associated factor 1	6.035706829	9.63E-28
CD2AP	CD2 associated protein	0.862557827	9.77E-28
DCX	doublecortin	-1.855057495	1.06E-27
PROS1	protein S	-1.271785157	1.16E-27
GYG2	glycogenin 2	-1.539388505	1.18E-27
FAM171B	family with sequence similarity 171 member B	-0.926469211	1.22E-27
NKAIN1	sodium/potassium transporting ATPase interacting 1	2.860102258	1.33E-27
DSTN	destrin; actin depolymerizing factor	-0.752769293	1.87E-27
ENAH	ENAH actin regulator	-0.781892326	2.03E-27
DDAH1	dimethylarginine dimethylaminohydrolase 1	-0.738743249	2.20E-27
B3GLCT	beta 3-glucosyltransferase	-1.117789293	2.25E-27
GABARAP	GABA type A receptor-associated protein	-0.828005724	2.35E-27
KLHDC8A	kelch domain containing 8A	-1.507389994	2.49E-27
SCO2	synthesis of cytochrome C oxidase 2	1.235377232	2.67E-27
LAMB3	laminin subunit beta 3	1.828597844	3.13E-27
GBP4	guanylate binding protein 4	6.403124951	3.60E-27
MXD3	MAX dimerization protein 3	-1.283031976	3.84E-27
CIRBP	cold inducible RNA binding protein	-0.715992227	4.16E-27
CHSY1	chondroitin sulfate synthase 1	0.757384364	4.58E-27
ENC1	ectodermal-neural cortex 1	-0.696721098	4.67E-27
PALM	paralemmin	-1.352164887	4.78E-27
SNRPA1	small nuclear ribonucleoprotein polypeptide A'	0.75520543	4.85E-27
UBE2Z	ubiquitin conjugating enzyme E2 Z	0.733279704	4.98E-27
LINC00641	long intergenic non-protein coding RNA 641	-1.566289145	5.04E-27
CALD1	caldesmon 1	-0.862751969	5.66E-27
JAM2	junctional adhesion molecule 2	1.200455988	5.67E-27

KCNMB4	potassium calcium-activated channel subfamily M regulatory beta subunit 4	-1.792503054	6.44E-27
NEDD4L	NEDD4 like E3 ubiquitin protein ligase	-1.020477475	6.61E-27
MOSPD3	motile sperm domain containing 3	-1.045444758	6.71E-27
SHISA5	shisa family member 5	0.710111582	7.01E-27
KCNIP4	potassium voltage-gated channel interacting protein 4	2.678899649	7.11E-27
PINK1	PTEN induced kinase 1	-0.89695287	7.69E-27
TYRO3	TYRO3 protein tyrosine kinase	-0.804486	7.97E-27
ETV1	ETS variant transcription factor 1	-1.007603515	7.97E-27
IL7R	interleukin 7 receptor	2.608290777	8.07E-27
PODXL2	podocalyxin like 2	0.856908327	8.57E-27
DHFR	dihydrofolate reductase	-0.700008106	8.59E-27
SH2B3	SH2B adaptor protein 3	1.47697561	1.22E-26
EDNRB	endothelin receptor type B	-2.139437739	1.29E-26
ANKIB1	ankyrin repeat and IBR domain containing 1	0.936493895	1.77E-26
MFSD2A	MFSD2 lysolipid transporter A; lysophospholipid	0.976813807	1.87E-26
LMO4	LIM domain only 4	0.948289364	1.96E-26
AFF2	ALF transcription elongation factor 2	-1.073189629	1.97E-26
PCDH1	protocadherin 1	3.744154946	2.15E-26
H2AC6	H2A clustered histone 6	1.785601677	2.43E-26
SIRT2	sirtuin 2	-0.822067345	2.46E-26
POU3F4	POU class 3 homeobox 4	-1.557919602	2.89E-26
CHROMR	cholesterol induced regulator of metabolism RNA	1.278761312	3.01E-26
HCFC1R1	host cell factor C1 regulator 1	-0.789387923	3.58E-26
TRAF3	TNF receptor associated factor 3	0.699738226	3.62E-26
FAM168B	family with sequence similarity 168 member B	-0.722909777	3.66E-26
FZD5	frizzled class receptor 5	0.74516409	3.66E-26
SMOX	spermine oxidase	0.998958763	3.66E-26
SAA2	serum amyloid A2	7.576869384	3.83E-26
PA2G4	proliferation-associated 2G4	0.595505722	3.90E-26
LGR4	leucine rich repeat containing G protein-coupled receptor 4	0.621154659	4.11E-26
PANTR1	POU3F3 adjacent non-coding transcript 1	-1.064408626	4.27E-26
ZEB1	zinc finger E-box binding homeobox 1	-0.753482778	4.28E-26
WDR49	WD repeat domain 49	3.412046707	4.64E-26
SDC4	syndecan 4	0.82577955	5.34E-26
ARMH4	armadillo like helical domain containing 4	-1.772840704	5.69E-26
APBB1	amyloid beta precursor protein binding family B member 1	-0.892954526	5.78E-26

DCTN2	dynactin subunit 2	-0.63745217	5.85E-26
FEM1C	fem-1 homolog C	0.783960328	6.00E-26
RRP1	ribosomal RNA processing 1	0.803420515	6.09E-26
SLC11A2	solute carrier family 11 member 2	0.754558178	6.57E-26
REEP1	receptor accessory protein 1	-2.150605532	6.72E-26
ANKRD6	ankyrin repeat domain 6	-1.874471739	7.46E-26
NINJ1	ninjurin 1	0.815698428	8.54E-26
MEGF10	multiple EGF like domains 10	-0.947822626	8.59E-26
IL6ST	interleukin 6 cytokine family signal transducer	0.700820504	9.04E-26
KCTD7	potassium channel tetramerization domain containing 7	-1.275276743	9.05E-26
LPAR4	lysophosphatidic acid receptor 4	-2.279479958	9.17E-26
DHX37	DEAH-box helicase 37	1.008871272	1.01E-25
ENDOD1	endonuclease domain containing 1	0.854529969	1.12E-25
RPF2	ribosome production factor 2 homolog	0.73825808	1.21E-25
COLGALT2	collagen beta(1-O)galactosyltransferase 2	-0.772520931	1.22E-25
GET1	guided entry of tail-anchored proteins factor 1	-0.980021834	1.22E-25
SLC30A7	solute carrier family 30 member 7	0.877154198	1.33E-25
SFXN3	sideroflexin 3	-0.953824182	1.38E-25
CNP	2';3'-cyclic nucleotide 3' phosphodiesterase	0.643533522	1.39E-25
FBXO6	F-box protein 6	2.333425502	1.48E-25
PHACTR4	phosphatase and actin regulator 4	0.734999329	1.51E-25
FXYD6	FXYD domain containing ion transport regulator 6	-0.753039935	1.95E-25
SLC38A5	solute carrier family 38 member 5	1.097443519	2.46E-25
ARHGAP33	Rho GTPase activating protein 33	-1.520556845	3.23E-25
CD47	CD47 molecule	0.878988505	3.96E-25
ATL2	atlastin GTPase 2	0.857094529	4.02E-25
CD46	CD46 molecule	0.837631118	4.50E-25
EPB41L1	erythrocyte membrane protein band 4,1 like 1	-1.070095592	4.61E-25
CNN2	calponin 2	-1.067033624	4.82E-25
PALLD	palladin; cytoskeletal associated protein	-1.064156426	5.00E-25
ALDH2	aldehyde dehydrogenase 2 family member	-0.987333968	5.20E-25
STK40	serine/threonine kinase 40	0.610542574	5.50E-25
CLIC4	chloride intracellular channel 4	0.844410967	5.71E-25
SPATA13	spermatogenesis associated 13	0.769094543	5.98E-25
BAK1	BCL2 antagonist/killer 1	0.99382948	6.39E-25
UCK2	uridine-cytidine kinase 2	0.849365969	6.63E-25
GTPBP2	GTP binding protein 2	0.816621359	7.18E-25

WDR4	WD repeat domain 4	0.972703934	7.28E-25
MFAP5	microfibril associated protein 5	-1.880575827	7.50E-25
CASP7	caspase 7	0.822256711	8.04E-25
HDAC5	histone deacetylase 5	-1.051686131	8.48E-25
MRPL44	mitochondrial ribosomal protein L44	0.929700603	1.03E-24
S100B	S100 calcium binding protein B	-1.62329598	1.09E-24
PRXL2B	peroxiredoxin like 2B	-0.738523951	1.26E-24
TNFRSF9	TNF receptor superfamily member 9	3.047492489	1.27E-24
GDPD2	glycerophosphodiester phosphodiesterase domain containing 2	-1.916203457	1.29E-24
TBK1	TANK binding kinase 1	0.803201065	1.35E-24
SLC44A2	solute carrier family 44 member 2	-0.921555521	1.40E-24
DDX10	DEAD-box helicase 10	0.625722098	1.59E-24
CNN1	calponin 1	-2.679881532	1.61E-24
JAKMIP2	janus kinase and microtubule interacting protein 2	-1.13645125	1.62E-24
PGAM2	phosphoglycerate mutase 2	-3.330554872	1.63E-24
RICTOR	RPTOR independent companion of MTOR complex 2	0.939636099	1.71E-24
CTPS1	CTP synthase 1	0.650483806	1.76E-24
AP1S2	adaptor related protein complex 1 subunit sigma 2	-0.949638356	1.87E-24
CSDC2	cold shock domain containing C2	-1.58202744	1.99E-24
BCAT1	branched chain amino acid transaminase 1	0.775299034	2.06E-24
PLPP5	phospholipid phosphatase 5	-1.017659324	2.20E-24
SLC40A1	solute carrier family 40 member 1	-1.71499718	2.27E-24
PRR36	proline rich 36	-1.280182576	2.32E-24
MEST	mesoderm specific transcript	0.662457918	2.41E-24
EIF4EBP2	eukaryotic translation initiation factor 4E binding protein 2	-0.689523789	2.55E-24
APPL2	adaptor protein; phosphotyrosine interacting with PH domain and leucine zipper 2	-1.244359767	2.85E-24
AHNAK2	AHNAK nucleoprotein 2	-1.412920293	2.90E-24
PITPNB	phosphatidylinositol transfer protein beta	0.756017365	2.90E-24
SOX9	SRY-box transcription factor 9	-0.818986598	3.13E-24
FBXL5	F-box and leucine rich repeat protein 5	-1.059700204	3.49E-24
SRC	SRC proto-oncogene; non-receptor tyrosine kinase	-0.964102455	3.49E-24
CACFD1	calcium channel flower domain containing 1	-1.373743946	3.67E-24
CTNNBIP1	catenin beta interacting protein 1	-0.983082364	4.00E-24
PI4K2B	phosphatidylinositol 4-kinase type 2 beta	1.184220863	4.15E-24

TXLNG	taxilin gamma	0.637205902	4.38E-24
SLC22A17	solute carrier family 22 member 17	-1.060614769	4.40E-24
DCHS1	dachsous cadherin-related 1	-1.228145957	5.33E-24
FAM200B	family with sequence similarity 200 member B	-1.088237434	5.62E-24
DPH2	diphthamide biosynthesis 2	0.802858368	5.96E-24
CCR1	C-C motif chemokine receptor 1	3.64648753	6.34E-24
LRP10	LDL receptor related protein 10	0.649969646	6.44E-24
TMEM109	transmembrane protein 109	0.780022668	6.83E-24
TCOF1	treacle ribosome biogenesis factor 1	0.686267248	7.59E-24
BLTP3A	bridge-like lipid transfer protein family member 3A	0.758786719	8.69E-24
SLC25A32	solute carrier family 25 member 32	0.996345932	9.19E-24
SERINC5	serine incorporator 5	-0.785311061	1.11E-23
LYAR	Ly1 antibody reactive	1.131615233	1.13E-23
DLK1	delta like non-canonical Notch ligand 1	-0.759475691	1.20E-23
HLA-G	major histocompatibility complex; class I; G	2.893920028	1.46E-23
STUM	stum; mechanosensory transduction mediator homolog	1.962360075	1.46E-23
ARID5A	AT-rich interaction domain 5A	1.101978426	1.48E-23
CRMP1	collapsin response mediator protein 1	-1.733938195	1.58E-23
ALDH4A1	aldehyde dehydrogenase 4 family member A1	-0.885601033	1.66E-23
CCDC71L	coiled-coil domain containing 71 like	1.35514575	1.78E-23
AK2	adenylate kinase 2	0.850554981	1.78E-23
DGLUCY	D-glutamate cyclase	0.98057888	1.80E-23
MICAL1	microtubule associated monooxygenase; calponin and LIM domain containing 1	-1.023940851	2.41E-23
TMTC2	transmembrane O-mannosyltransferase targeting cadherins 2	-1.210083377	2.55E-23
TPM2	tropomyosin 2	-0.85239519	2.71E-23
FMR1	fragile X messenger ribonucleoprotein 1	0.805154995	2.79E-23
VIM	vimentin	-0.653182251	3.07E-23
MTF1	metal regulatory transcription factor 1	1.119725124	3.63E-23
PSMA5	proteasome 20S subunit alpha 5	0.96731094	3.66E-23
SLC5A6	solute carrier family 5 member 6	0.724418146	3.73E-23
FUT9	fucosyltransferase 9	-1.633492615	3.82E-23
DPYD	dihydropyrimidine dehydrogenase	0.758020565	4.24E-23
SHB	SH2 domain containing adaptor protein B	0.63317869	4.28E-23
NLGN3	neuroligin 3	-1.338730455	4.38E-23
DDX24	DEAD-box helicase 24	0.595628612	4.67E-23
PTPRA	protein tyrosine phosphatase receptor type A	0.699490571	5.24E-23

MAP2	microtubule associated protein 2	-0.920015689	5.66E-23
NEFH	neurofilament heavy chain	1.422018324	6.01E-23
SAPCD2	suppressor APC domain containing 2	-0.994104369	6.27E-23
PLXNB3	plexin B3	-1.354582346	6.40E-23
MAGED1	MAGE family member D1	-0.71790295	6.48E-23
TOM1L2	target of myb1 like 2 membrane trafficking protein	-1.125654359	9.72E-23
RAB30	RAB30; member RAS oncogene family	1.642584328	1.01E-22
SLC27A2	solute carrier family 27 member 2	1.741635114	1.11E-22
ARF5	ADP ribosylation factor 5	-0.767661714	1.17E-22
ANPEP	alanyl aminopeptidase; membrane	4.539755405	1.23E-22
H2AC25	H2A clustered histone 25	1.313986422	1.25E-22
ATP13A2	ATPase cation transporting 13A2	-0.820436458	1.28E-22
SDC3	syndecan 3	-0.760772633	1.36E-22
PSD4	pleckstrin and Sec7 domain containing 4	1.08661845	1.38E-22
BCO1	beta-carotene oxygenase 1	3.176399621	1.52E-22
RRP12	ribosomal RNA processing 12 homolog	0.772775388	1.78E-22
LMOD1	leiomodin 1	-4.402481744	1.84E-22
KLHL25	kelch like family member 25	-0.626136427	2.01E-22
MAK16	MAK16 homolog	0.857851807	2.51E-22
GAMT	guanidinoacetate N-methyltransferase	-0.98195813	2.52E-22
IFNGR2	interferon gamma receptor 2	0.778351518	2.52E-22
HSH2D	hematopoietic SH2 domain containing	5.747258358	2.73E-22
PSAP	prosaposin	-0.63466156	2.74E-22
CD70	CD70 molecule	2.737245562	3.41E-22
PAFAH1B3	platelet activating factor acetylhydrolase 1b catalytic subunit 3	-0.767566489	4.21E-22
PDPN	podoplanin	0.960486328	4.28E-22
TIFA	TRAF interacting protein with forkhead associated domain	1.928344299	4.31E-22
CASTOR2	cytosolic arginine sensor for mTORC1 subunit 2	-1.237231523	4.92E-22
SLC47A2	solute carrier family 47 member 2	-2.607362582	5.04E-22
PDP1	pyruvate dehydrogenase phosphatase catalytic subunit 1	0.65717114	5.21E-22
INTS7	integrator complex subunit 7	0.715603079	6.42E-22
IGSF11	immunoglobulin superfamily member 11	-1.426402594	6.89E-22
DACT3	dishevelled binding antagonist of beta catenin 3	-1.388695361	7.18E-22
RCN2	reticulocalbin 2	-0.661592177	7.37E-22
FOXJ1	forkhead box J1	-0.888050019	7.46E-22
TMEM256	transmembrane protein 256	-1.238841186	7.60E-22
P2RX4	purinergic receptor P2X 4	1.134499481	8.10E-22
PAQR8	progestin and adipoQ receptor family member 8	-1.329817275	8.34E-22

PMM2	phosphomannomutase 2	0.765418948	8.47E-22
COA7	cytochrome c oxidase assembly factor 7	1.040859015	9.90E-22
SOX1	SRY-box transcription factor 1	-0.688100026	1.02E-21
SEMA5B	semaphorin 5B	-1.073110911	1.03E-21
NIP7	nucleolar pre-rRNA processing protein NIP7	0.915135421	1.03E-21
LDLRAD3	low density lipoprotein receptor class A domain containing 3	-0.639006698	1.18E-21
KIF5C	kinesin family member 5C	-0.90105679	1.43E-21
KIDINS220	kinase D interacting substrate 220	-0.745365344	1.43E-21
FAM168A	family with sequence similarity 168 member A	-0.861371626	1.50E-21
TTYH1	tweety family member 1	-0.661385727	1.56E-21
RCL1	RNA terminal phosphate cyclase like 1	1.085558362	1.78E-21
RNF149	ring finger protein 149	0.984611149	1.80E-21
ILK	integrin linked kinase	-0.751701305	1.90E-21
SERPINA3	serpin family A member 3	3.707334169	2.09E-21
TNF	tumor necrosis factor	6.325803226	2.13E-21
DNAJC2	DnaJ heat shock protein family (Hsp40) member C2	0.787515804	2.21E-21
CD83	CD83 molecule	1.766940871	2.26E-21
SNHG19	small nucleolar RNA host gene 19	-1.902975032	2.43E-21
NAF1	nuclear assembly factor 1 ribonucleoprotein	0.825966917	2.46E-21
POLR1B	RNA polymerase I subunit B	1.080116994	2.50E-21
PDZD4	PDZ domain containing 4	-1.402482373	2.60E-21
CPNE2	copine 2	-1.520166884	2.60E-21
APOL3	apolipoprotein L3	5.562474182	2.64E-21
EFR3A	EFR3 homolog A	0.599267909	2.75E-21
MLKL	mixed lineage kinase domain like pseudokinase	3.867525173	3.47E-21
SNTB1	syntrophin beta 1	-1.115272594	3.50E-21
HES7	hes family bHLH transcription factor 7	1.597339829	3.89E-21
RBM20	RNA binding motif protein 20	-0.880657892	3.91E-21
GRPEL1	GrpE like 1; mitochondrial	0.722709257	3.99E-21
HOMER3	homer scaffold protein 3	-0.767282239	4.16E-21
GDF15	growth differentiation factor 15	1.180291017	4.31E-21
ADPGK	ADP dependent glucokinase	0.709416931	4.54E-21
BISPR	BST2 interferon stimulated positive regulator	2.188961584	5.08E-21
GNAO1	G protein subunit alpha o1	-1.304134452	6.07E-21
GBX2	gastrulation brain homeobox 2	2.757363666	6.27E-21
HMGB3	high mobility group box 3	-0.994830794	6.30E-21
GPR137B	G protein-coupled receptor 137B	-1.118837222	6.50E-21

PPP1R1B	protein phosphatase 1 regulatory inhibitor subunit 1B	-1.2345204	7.21E-21
PWP2	PWP2 small subunit processome component	0.658344042	7.47E-21
EPHB2	EPH receptor B2	0.833648722	7.57E-21
TRIM45	tripartite motif containing 45	-1.422667681	7.65E-21
EGR3	early growth response 3	2.602283866	7.94E-21
IGFBP4	insulin like growth factor binding protein 4	1.528172711	8.77E-21
KCNJ13	potassium inwardly rectifying channel subfamily J member 13	-4.153367556	9.24E-21
CAT	catalase	-0.757938918	9.95E-21
PDCD5	programmed cell death 5	0.866623993	1.04E-20
CDKN2C	cyclin dependent kinase inhibitor 2C	-1.190632638	1.08E-20
PPAN	peter pan homolog	0.760683413	1.11E-20
CARS1	cysteinyl-tRNA synthetase 1	0.679281658	1.20E-20
TMEM47	transmembrane protein 47	-0.863487479	1.24E-20
ITPKC	inositol-trisphosphate 3-kinase C	0.795319397	1.25E-20
ASNS	asparagine synthetase (glutamine-hydrolyzing)	1.305639517	1.28E-20
KLHL7	kelch like family member 7	-0.918687777	1.28E-20
COL4A5	collagen type IV alpha 5 chain	-1.231003579	1.32E-20
LRRC17	leucine rich repeat containing 17	-0.977161221	1.39E-20
PDE8B	phosphodiesterase 8B	-29.99010211	1.45E-20
RAB39A	RAB39A; member RAS oncogene family	1.73479255	1.48E-20
TCEA2	transcription elongation factor A2	-0.777538311	1.52E-20
LRRC4	leucine rich repeat containing 4	-1.546218993	1.59E-20
MIR9-2HG	MIR9-2 host gene	-0.899315438	1.68E-20
DYNLT1	dynein light chain Tctex-type 1	1.003907144	1.71E-20
THTPA	thiamine triphosphatase	0.88964243	1.84E-20
SMIM14	small integral membrane protein 14	1.363857701	1.84E-20
TIMP2	TIMP metallopeptidase inhibitor 2	-1.240701138	1.90E-20
MIR1915HG	MIR1915 host gene	-1.419666098	1.95E-20
GSDME	gasdermin E	-1.343834834	1.96E-20
CTNND2	catenin delta 2	-1.606753227	2.00E-20
FGFR2	fibroblast growth factor receptor 2	-0.79615723	2.01E-20
MGAT1	alpha-1;3-mannosyl-glycoprotein 2-beta-N-acetylglucosaminyltransferase	0.642006854	2.37E-20
OTOF	otoferlin	2.678190028	2.58E-20
CYB5D2	cytochrome b5 domain containing 2	-0.951799384	2.58E-20
MPDZ	multiple PDZ domain crumbs cell polarity complex component	-0.965670616	2.60E-20
FLNA	filamin A	-0.95629723	2.74E-20
PLD2	phospholipase D2	-1.05847783	2.84E-20
MT1G	metallothionein 1G	6.502541872	2.87E-20

CNDP2	carnosine dipeptidase 2	0.717103069	2.93E-20
NCLN	nicalin	0.743805497	2.93E-20
NOP58	NOP58 ribonucleoprotein	0.590996653	3.00E-20
RIPOR3	RIPOR family member 3	2.69907838	3.36E-20
PTPN12	protein tyrosine phosphatase non-receptor type 12	0.777732075	3.61E-20
PGAM1	phosphoglycerate mutase 1	0.729938701	3.62E-20
MYCBP2	MYC binding protein 2	1.059144039	3.82E-20
RPLP0	ribosomal protein lateral stalk subunit P0	-0.596437262	4.14E-20
CAPS	calcyphosine	-1.794022728	4.52E-20
EPOP	elongin BC and polycomb repressive complex 2 associated protein	0.992770555	4.76E-20
TEAD4	TEA domain transcription factor 4	0.749935501	4.86E-20
ITGB4	integrin subunit beta 4	-0.803817604	4.90E-20
SYT12	synaptotagmin 12	0.892952802	4.99E-20
SERPINB6	serpin family B member 6	-0.710163755	5.07E-20
GPR162	G protein-coupled receptor 162	-1.992619822	5.10E-20
H3-3A	H3,3 histone A	-0.648674702	5.15E-20
DDN	dendrin	1.160464719	5.17E-20
BCCIP	BRCA2 and CDKN1A interacting protein	0.828503441	5.75E-20
DHRS2	dehydrogenase/reductase 2	3.539894242	6.01E-20
TRIM8	tripartite motif containing 8	0.622405116	6.30E-20
MET	MET proto-oncogene; receptor tyrosine kinase	1.444922385	7.10E-20
PFKFB3	6-phosphofructo-2-kinase/fructose-2;6-biphosphatase 3	0.789585747	7.61E-20
BMP7	bone morphogenetic protein 7	-1.033130943	7.76E-20
RUBCN	rubicon autophagy regulator	0.839957516	7.78E-20
GNAS	GNAS complex locus	-0.599301957	8.00E-20
SLC1A5	solute carrier family 1 member 5	0.81966871	9.09E-20
TAF9B	TATA-box binding protein associated factor 9b	-0.821828095	9.24E-20
TRO	trophinin	-0.879380707	9.24E-20
HIP1	huntingtin interacting protein 1	-0.871158352	9.41E-20
RYR2	ryanodine receptor 2	-1.980280636	9.48E-20
EGR2	early growth response 2	1.202287953	9.87E-20
COL26A1	collagen type XXVI alpha 1 chain	-0.956101908	1.01E-19
SHC3	SHC adaptor protein 3	-0.838762763	1.01E-19
MTFP1	mitochondrial fission process 1	1.348885571	1.01E-19
SNCAIP	synuclein alpha interacting protein	-0.72649235	1.02E-19
MYL6	myosin light chain 6	-0.819151302	1.04E-19
BAIAP2-DT	BAIAP2 divergent transcript	-1.047497079	1.04E-19
PAQR6	progestin and adipoQ receptor family member 6	-1.376008398	1.07E-19

UTP20	UTP20 small subunit processome component	0.625003657	1.15E-19
GPR180	G protein-coupled receptor 180	0.843350071	1.18E-19
ZCCHC24	zinc finger CCHC-type containing 24	-0.790326679	1.19E-19
RRP1B	ribosomal RNA processing 1B	0.596639143	1.19E-19
AHSA1	activator of HSP90 ATPase activity 1	0.860454173	1.38E-19
KCNQ5	potassium voltage-gated channel subfamily Q member 5	0.858236881	1.40E-19
NRARP	NOTCH regulated ankyrin repeat protein	0.701391497	1.45E-19
CAV2	caveolin 2	0.646259779	1.47E-19
ADGRB2	adhesion G protein-coupled receptor B2	-0.960294947	1.55E-19
SAMD11	sterile alpha motif domain containing 11	-2.188098829	1.59E-19
SHISA4	shisa family member 4	-1.200285557	1.62E-19
PLAAT2	phospholipase A and acyltransferase 2	4.414001449	1.74E-19
PRSS33	serine protease 33	-19.52852644	1.84E-19
TP53INP2	tumor protein p53 inducible nuclear protein 2	-1.037064648	1.89E-19
PTPRG	protein tyrosine phosphatase receptor type G	-1.051774417	1.90E-19
ASIC1	acid sensing ion channel subunit 1	-1.072469026	1.94E-19
DPY19L3	dpy-19 like C-mannosyltransferase 3	-0.961623473	2.10E-19
DPYSL4	dihydropyrimidinase like 4	-0.667623729	2.15E-19
CXCL3	C-X-C motif chemokine ligand 3	6.702174708	2.28E-19
UBE2E3	ubiquitin conjugating enzyme E2 E3	-0.604933744	2.37E-19
PKD1P6-NP	PKD1P6-NPIPP1 readthrough	1.065740954	2.56E-19
CHORDC1	cysteine and histidine rich domain containing 1	0.726112203	2.57E-19
DNAJA3	DnaJ heat shock protein family (Hsp40) member A3	0.704021187	2.77E-19
BMERB1	bMERB domain containing 1	-0.645849263	2.77E-19
PRKD1	protein kinase D1	-0.961154237	2.94E-19
NOC4L	nucleolar complex associated 4 homolog	0.825638627	2.96E-19
COL7A1	collagen type VII alpha 1 chain	1.881772493	2.98E-19
HSPE1	heat shock protein family E (Hsp10) member 1	0.703707106	3.07E-19
BCL2L13	BCL2 like 13	0.906830108	3.23E-19
PPP2R2A	protein phosphatase 2 regulatory subunit Balpha	0.639432275	3.49E-19
NDFIP1	Nedd4 family interacting protein 1	-0.733902615	3.64E-19
CRISPLD1	cysteine rich secretory protein LCCL domain containing 1	-0.976356872	3.67E-19
STMP1	short transmembrane mitochondrial protein 1	-0.649721295	4.07E-19
MRPS6	mitochondrial ribosomal protein S6	-0.650647802	4.29E-19
GNG7	G protein subunit gamma 7	-1.51030206	4.57E-19

SIAH3	siah E3 ubiquitin protein ligase family member 3	-1.95254825	4.57E-19
PTTG1IP	PTTG1 interacting protein	-0.602144593	4.71E-19
TMSB15A	thymosin beta 15A	-1.707873721	5.08E-19
PES1	pescadillo ribosomal biogenesis factor 1	0.65879092	5.15E-19
LDHA	lactate dehydrogenase A	0.886377038	5.16E-19
NDUFA9	NADH:ubiquinone oxidoreductase subunit A9	0.97533536	5.18E-19
MAPK8IP2	mitogen-activated protein kinase 8 interacting protein 2	0.775522794	5.39E-19
TNFAIP6	TNF alpha induced protein 6	6.964995401	5.45E-19
MOXD1	monooxygenase DBH like 1	-1.195633362	6.00E-19
RBM19	RNA binding motif protein 19	0.762585221	6.06E-19
TP53INP1	tumor protein p53 inducible nuclear protein 1	-0.75771088	6.12E-19
CXCL11	C-X-C motif chemokine ligand 11	7.103971638	6.18E-19
DNAJB6	DnaJ heat shock protein family (Hsp40) member B6	0.818797369	6.52E-19
SYNGR2	synaptogyrin 2	1.005492403	7.78E-19
TOP2A	DNA topoisomerase II alpha	-0.781827885	7.78E-19
TTYH3	tweety family member 3	-0.720318299	7.81E-19
NAV1	neuron navigator 1	-1.0095878	8.17E-19
PLD6	phospholipase D family member 6	0.779792572	8.30E-19
SALL2	spalt like transcription factor 2	-0.640631917	8.32E-19
ACAA2	acetyl-CoA acyltransferase 2	-0.726716838	9.09E-19
FARP1	FERM; ARH/RhoGEF and pleckstrin domain protein 1	-0.693875584	1.02E-18
GAL	galanin and GMAP prepropeptide	5.597127259	1.10E-18
NUAK1	NUAK family kinase 1	-0.879403053	1.11E-18
CASP4	caspase 4	3.771942956	1.12E-18
AMER1	APC membrane recruitment protein 1	1.057224919	1.14E-18
ALPL	alkaline phosphatase; biomineralization associated	-1.638272273	1.20E-18
GPM6B	glycoprotein M6B	-0.615312487	1.20E-18
IGFBPL1	insulin like growth factor binding protein like 1	-1.399470144	1.35E-18
METTL13	methyltransferase 13; eEF1A N-terminus and K55	0.667565047	1.38E-18
TMEM87A	transmembrane protein 87A	0.65931701	1.46E-18
VASH1	vasohibin 1	-1.118034632	1.47E-18
FRY	FRY microtubule binding protein	-2.085988092	1.47E-18
C21orf62	chromosome 21 open reading frame 62	-0.956004481	1.51E-18
KAT6B	lysine acetyltransferase 6B	-0.770043905	1.64E-18
NCEH1	neutral cholesterol ester hydrolase 1	0.942875935	1.65E-18
OGFOD1	2-oxoglutarate and iron dependent oxygenase domain containing 1	0.733666427	1.66E-18

ARNT2	aryl hydrocarbon receptor nuclear translocator 2	-0.788791213	1.66E-18
PDCD11	programmed cell death 11	0.615198013	1.66E-18
TMEM50A	transmembrane protein 50A	0.650834577	1.69E-18
APCDD1L	APC down-regulated 1 like	2.026837006	1.72E-18
ZYX	zyxin	-0.796410953	1.75E-18
RIPK2	receptor interacting serine/threonine kinase 2	0.969158181	1.78E-18
ASCL1	achaete-scute family bHLH transcription factor 1	-1.248083091	1.82E-18
PBXIP1	PBX homeobox interacting protein 1	-0.764609207	1.90E-18
ARRDC3	arrestin domain containing 3	1.114560203	2.03E-18
STARD4	StAR related lipid transfer domain containing 4	0.630315795	2.20E-18
EFR3B	EFR3 homolog B	-1.066881401	2.27E-18
KCNJ10	potassium inwardly rectifying channel subfamily J member 10	-1.018866744	2.32E-18
NSUN5	NOP2/Sun RNA methyltransferase 5	0.75818802	2.46E-18
PSMG1	proteasome assembly chaperone 1	0.888203237	2.54E-18
PLEKHS1	pleckstrin homology domain containing S1	3.665071575	2.56E-18
TRIM35	tripartite motif containing 35	0.954868571	2.65E-18
TAB3	TGF-beta activated kinase 1 (MAP3K7) binding protein 3	0.863208798	2.76E-18
CAPN5	calpain 5	-1.002282773	2.97E-18
ITSN1	intersectin 1	-0.991428068	3.04E-18
CEBPZ	CCAAT enhancer binding protein zeta	0.745266533	3.22E-18
ATP10A	ATPase phospholipid transporting 10A (putative)	0.919530844	3.53E-18
BACE2	beta-secretase 2	0.874958258	3.79E-18
WNT7B	Wnt family member 7B	1.155404522	3.87E-18
CADM3	cell adhesion molecule 3	2.891340571	4.02E-18
NMT2	N-myristoyltransferase 2	-0.825573674	4.31E-18
TCF7L1	transcription factor 7 like 1	-0.93497213	5.07E-18
ANXA6	annexin A6	-0.662549934	5.95E-18
ARPIN	actin related protein 2/3 complex inhibitor	-0.819902454	5.99E-18
SYBU	syntabulin	-1.1241365	6.18E-18
ERP44	endoplasmic reticulum protein 44	0.590599748	6.18E-18
DLG1	discs large MAGUK scaffold protein 1	-0.744161287	6.20E-18
CD320	CD320 molecule	0.648802546	6.50E-18
EPB41L5	erythrocyte membrane protein band 4,1 like 5	-0.743255199	7.08E-18
ELAVL3	ELAV like RNA binding protein 3	-1.527456042	8.98E-18
PCDHGC5	protocadherin gamma subfamily C; 5	-1.084289803	9.45E-18
LIPA	lipase A; lysosomal acid type	0.694652353	9.67E-18

ARRDC4	arrestin domain containing 4	-0.592924016	9.67E-18
MFGE8	milk fat globule EGF and factor V/VIII domain containing	-0.873757082	1.06E-17
ELMO1	engulfment and cell motility 1	-0.82158093	1.07E-17
EIF3L	eukaryotic translation initiation factor 3 subunit L	-0.627680674	1.08E-17
TMEM8B	transmembrane protein 8B	-1.17927353	1.10E-17
CHL1	cell adhesion molecule L1 like	-1.076353997	1.14E-17
PLD5	phospholipase D family member 5	-1.300952127	1.19E-17
ZFYVE26	zinc finger FYVE-type containing 26	0.881905261	1.19E-17
CLN6	CLN6 transmembrane ER protein	0.688714639	1.22E-17
TGFB2	transforming growth factor beta 2	-0.6034647	1.33E-17
EBI3	Epstein-Barr virus induced 3	4.103935803	1.33E-17
SUN2	Sad1 and UNC84 domain containing 2	-1.13844064	1.38E-17
PNP	purine nucleoside phosphorylase	0.898881032	1.44E-17
TREX1	three prime repair exonuclease 1	1.052525645	1.49E-17
PRKCQ-AS1	PRKCQ antisense RNA 1	1.564574576	1.59E-17
LUCAT1	lung cancer associated transcript 1	3.207973756	1.64E-17
MBOAT2	membrane bound O-acyltransferase domain containing 2	-0.856839181	1.76E-17
KRT75	keratin 75	1.154058787	1.77E-17
CARD16	caspase recruitment domain family member 16	1.613598804	1.77E-17
IGSF3	immunoglobulin superfamily member 3	-0.878978085	1.84E-17
C15orf48	chromosome 15 open reading frame 48	5.505906294	1.92E-17
FAM13C	family with sequence similarity 13 member C	-1.196429629	1.93E-17
ELOA	elongin A	0.649309953	1.94E-17
TGS1	trimethylguanosine synthase 1	0.613650462	2.05E-17
CD74	CD74 molecule	0.978845373	2.13E-17
ACSL4	acyl-CoA synthetase long chain family member 4	0.714482789	2.15E-17
GM2A	ganglioside GM2 activator	-0.852909571	2.19E-17
MATN2	matrilin 2	-1.531641028	2.29E-17
FAM171A1	family with sequence similarity 171 member A1	-0.823352798	2.65E-17
RRAD	RRAD; Ras related glycolysis inhibitor and calcium channel regulator	1.211061864	2.69E-17
SOX2-OT	SOX2 overlapping transcript	-1.001552067	3.06E-17
PODXL	podocalyxin like	0.652427924	3.19E-17
RET	ret proto-oncogene	4.284478026	3.27E-17
PTPRS	protein tyrosine phosphatase receptor type S	-0.78873538	3.33E-17
TCAF1	TRPM8 channel associated factor 1	-0.778787957	3.39E-17
EFNB3	ephrin B3	-0.929997833	3.45E-17
NACC2	NACC family member 2	-0.713553631	3.45E-17

GABRB3	gamma-aminobutyric acid type A receptor subunit beta3	-1.499520115	3.54E-17
GAB1	GRB2 associated binding protein 1	-0.766744968	3.54E-17
COX20	cytochrome c oxidase assembly factor COX20	-0.792977391	3.72E-17
THSD1	thrombospondin type 1 domain containing 1	0.790921314	3.80E-17
TRMT1	tRNA methyltransferase 1	0.590111035	3.95E-17
ZBP1	Z-DNA binding protein 1	7.930417272	4.05E-17
CX3CL1	C-X3-C motif chemokine ligand 1	0.760915238	4.05E-17
GPSM1	G protein signaling modulator 1	-0.776122609	4.08E-17
GALNT18	polypeptide N-acetylgalactosaminyltransferase 18	0.832719324	4.35E-17
SPAG9	sperm associated antigen 9	-0.790202247	4.42E-17
SLC25A22	solute carrier family 25 member 22	0.726491318	4.45E-17
SLC16A2	solute carrier family 16 member 2	-0.659967442	4.53E-17
ESRRA	estrogen related receptor alpha	0.745911576	4.61E-17
CMTM4	CKLF like MARVEL transmembrane domain containing 4	-0.907393291	4.72E-17
SLC4A3	solute carrier family 4 member 3	-1.055318285	4.74E-17
H2BC21	H2B clustered histone 21	0.917106778	4.85E-17
FREM2	FRAS1 related extracellular matrix 2	-1.315068439	5.01E-17
SELENOO	selenoprotein O	0.719069201	5.12E-17
SP9	Sp9 transcription factor	-0.741022102	5.28E-17
SEC16B	SEC16 homolog B; endoplasmic reticulum export factor	3.733632267	5.30E-17
B3GNT2	UDP-GlcNAc:betaGal beta-1;3-N-acetylglucosaminyltransferase 2	0.914985305	5.60E-17
NCAN	neurocan	-1.018111	6.21E-17
NAV2	neuron navigator 2	-1.204206913	6.21E-17
CCDC86	coiled-coil domain containing 86	0.738003791	6.67E-17
ETV6	ETS variant transcription factor 6	0.795845317	6.68E-17
CDC42EP1	CDC42 effector protein 1	-0.705776745	7.18E-17
DGCR6	DiGeorge syndrome critical region gene 6	-1.446708677	7.18E-17
FLT3LG	fms related receptor tyrosine kinase 3 ligand	2.31973865	7.81E-17
SQSTM1	sequestosome 1	0.598412337	8.13E-17
IRF2BPL	interferon regulatory factor 2 binding protein like	0.629598468	8.26E-17
ANOS1	anosmin 1	-1.702048608	8.99E-17
NECTIN2	nectin cell adhesion molecule 2	0.720702129	9.44E-17
CIMAP1B	ciliary microtubule associated protein 1B	6.00951108	9.69E-17
SUPV3L1	Suv3 like RNA helicase	0.841732496	1.02E-16
CLN8-AS1	CLN8 antisense RNA 1	-1.466059033	1.08E-16
ITGB5	integrin subunit beta 5	-1.142831409	1.13E-16

PPP2R1B	protein phosphatase 2 scaffold subunit Abeta	0.63023794	1.16E-16
ALDH5A1	aldehyde dehydrogenase 5 family member A1	-0.892162969	1.19E-16
MYH10	myosin heavy chain 10	-0.924198808	1.21E-16
TSPAN12	tetraspanin 12	-1.389024785	1.21E-16
TRIM56	tripartite motif containing 56	0.790383928	1.24E-16
ARL6IP1	ADP ribosylation factor like GTPase 6 interacting protein 1	-0.602109525	1.32E-16
SPAG16	sperm associated antigen 16	-0.953891294	1.37E-16
SYTL2	synaptotagmin like 2	-1.380281862	1.39E-16
NOD1	nucleotide binding oligomerization domain containing 1	1.055952504	1.46E-16
PKD1P6	polycystin 1; transient receptor potential channel interacting pseudogene 6	1.136614078	1.65E-16
BCL7C	BAF chromatin remodeling complex subunit BCL7C	-0.661014786	1.76E-16
INPPL1	inositol polyphosphate phosphatase like 1	-0.819073971	1.77E-16
GSTO1	glutathione S-transferase omega 1	0.848418337	1.82E-16
FAM120C	family with sequence similarity 120 member C	0.88479515	1.83E-16
EFL1	elongation factor like GTPase 1	0.831276285	1.86E-16
BBX	BBX high mobility group box domain containing	0.696729523	1.92E-16
ILDR2	immunoglobulin like domain containing receptor 2	-1.102315751	1.97E-16
ARSJ	arylsulfatase family member J	0.730868622	1.98E-16
ELK4	ETS transcription factor ELK4	0.778776774	2.00E-16
AHNAK	AHNAK nucleoprotein	-1.188344527	2.03E-16
AMD1	adenosylmethionine decarboxylase 1	0.622391858	2.17E-16
CCDC117	coiled-coil domain containing 117	0.732511985	2.18E-16
MIAT	myocardial infarction associated transcript	-1.490490417	2.50E-16
SLC29A4	solute carrier family 29 member 4	-1.121069431	2.84E-16
BACH2	BTB domain and CNC homolog 2	-1.154320156	3.12E-16
DAB1	DAB adaptor protein 1	-1.358484375	3.27E-16
ACBD7	acyl-CoA binding domain containing 7	-0.940256163	3.28E-16
ANKS1B	ankyrin repeat and sterile alpha motif domain containing 1B	-1.487613681	3.33E-16
VDR	vitamin D receptor	1.695740565	3.45E-16
UBALD1	UBA like domain containing 1	0.76800749	3.59E-16
MYO10	myosin X	-0.762327056	3.68E-16
LONRF2	LON peptidase N-terminal domain and ring finger 2	-0.783850803	3.84E-16
GINS4	GINS complex subunit 4	-0.782218771	3.87E-16

PIMREG	PICALM interacting mitotic regulator	-1.207951144	3.88E-16
MTO1	mitochondrial tRNA translation optimization 1	0.762504379	3.92E-16
GCNT1	glucosaminyl (N-acetyl) transferase 1	-1.003700477	3.94E-16
SLC16A6	solute carrier family 16 member 6	3.434747328	3.96E-16
LINC00511	long intergenic non-protein coding RNA 511	-0.763309665	3.98E-16
PLEKHG4B	pleckstrin homology and RhoGEF domain containing G4B	-0.931759824	3.98E-16
ANK1	ankyrin 1	-0.995959971	4.01E-16
ATP10D	ATPase phospholipid transporting 10D (putative)	0.955279385	4.23E-16
ECH1	enoyl-CoA hydratase 1	-0.877354272	4.39E-16
HOMER1	homer scaffold protein 1	0.803381375	4.62E-16
LRIF1	ligand dependent nuclear receptor interacting factor 1	0.765651893	4.66E-16
IP6K3	inositol hexakisphosphate kinase 3	3.75016299	4.71E-16
WDR36	WD repeat domain 36	0.794426583	4.76E-16
NIPAL3	NIPA like domain containing 3	-0.801535843	4.82E-16
OLMALINC	oligodendrocyte maturation-associated long intergenic non-coding RNA	-0.876495045	4.95E-16
MAGED2	MAGE family member D2	-0.689189644	4.95E-16
MTSS2	MTSS I-BAR domain containing 2	-0.808257071	5.03E-16
SLFN12	schlafen family member 12	0.80513715	5.15E-16
RBM34	RNA binding motif protein 34	0.840388424	5.45E-16
DBF4B	DBF4 zinc finger B	0.712675221	5.92E-16
TMEM229B	transmembrane protein 229B	2.78276405	6.21E-16
MAP2K3	mitogen-activated protein kinase kinase 3	0.591865785	6.39E-16
COL6A2	collagen type VI alpha 2 chain	1.047975051	7.71E-16
BAHCC1	BAH domain and coiled-coil containing 1	0.766897079	7.80E-16
FJX1	four-jointed box kinase 1	-0.601648508	7.81E-16
WDR43	WD repeat domain 43	0.597351226	8.00E-16
H2AZ2	H2A,Z variant histone 2	-0.61295453	8.04E-16
FAM210A	family with sequence similarity 210 member A	0.826546963	8.10E-16
ATP1A2	ATPase Na+/K+ transporting subunit alpha 2	-3.134831638	8.15E-16
MAX	MYC associated factor X	0.764079728	8.36E-16
REL	REL proto-oncogene; NF-kB subunit	1.110800379	9.22E-16
SMARCD3	SWI/SNF related; matrix associated; actin dependent regulator of chromatin; subfamily d; member 3	-0.616206748	9.65E-16
ATAT1	alpha tubulin acetyltransferase 1	-0.845629853	9.83E-16
SLC19A2	solute carrier family 19 member 2	0.847035149	9.83E-16
ALOX5	arachidonate 5-lipoxygenase	2.646286677	1.01E-15

MDFIC	MyoD family inhibitor domain containing	-1.226333399	1.02E-15
PCSK5	proprotein convertase subtilisin/kexin type 5	-1.096012616	1.08E-15
WDR74	WD repeat domain 74	0.603229088	1.16E-15
GTF2IP4	general transcription factor IIi pseudogene 4	-0.987042777	1.17E-15
Aug-01	argonaute RISC component 1	-0.865498714	1.18E-15
SLC16A9	solute carrier family 16 member 9	-0.907962511	1.26E-15
MARCHF2	membrane associated ring-CH-type finger 2	-0.811985185	1.27E-15
SLC2A10	solute carrier family 2 member 10	-1.134288261	1.28E-15
TLR4	toll like receptor 4	1.284913265	1.30E-15
PLOD3	procollagen-lysine;2-oxoglutarate 5-dioxygenase 3	0.71918386	1.32E-15
TWNK	twinkle mtDNA helicase	0.829110105	1.40E-15
CA12	carbonic anhydrase 12	0.598419978	1.45E-15
CARD10	caspase recruitment domain family member 10	-1.084800566	1.50E-15
ANGPTL1	angiopoietin like 1	1.248288071	1.54E-15
SYNM	synemin	-0.831029768	1.66E-15
RUNX1	RUNX family transcription factor 1	0.674262467	1.69E-15
LGI2	leucine rich repeat LGI family member 2	2.442354119	1.70E-15
DLX1	distal-less homeobox 1	-0.913781727	1.76E-15
WLS	Wnt ligand secretion mediator	-1.02936851	1.79E-15
PKIA	cAMP-dependent protein kinase inhibitor alpha	-1.096057256	1.86E-15
HDC	histidine decarboxylase	-2.101743416	1.92E-15
IER5L	immediate early response 5 like	1.00669468	1.96E-15
RRP9	ribosomal RNA processing 9; U3 small nucleolar RNA binding protein	0.70194355	2.11E-15
AQP1	aquaporin 1 (Colton blood group)	-1.422893783	2.12E-15
NCR3LG1	natural killer cell cytotoxicity receptor 3 ligand 1	0.702436846	2.36E-15
KRBA2	KRAB-A domain containing 2	-1.189742179	2.41E-15
HSPA4L	heat shock protein family A (Hsp70) member 4 like	0.674531466	2.44E-15
CALCOCO2	calcium binding and coiled-coil domain 2	1.030321643	2.44E-15
IMP4	IMP U3 small nucleolar ribonucleoprotein 4	0.684481545	2.52E-15
TTLL1	TTL family tubulin polyglutamylase complex subunit L1	-0.901865018	2.55E-15
BRIX1	biogenesis of ribosomes BRX1	0.690180625	2.70E-15
ZIC4	Zic family member 4	1.173636407	2.73E-15
RFFL	ring finger and FYVE like domain containing E3 ubiquitin protein ligase	0.733020941	2.91E-15
NATD1	N-acetyltransferase domain containing 1	-1.011211445	2.93E-15

BIRC3	baculoviral IAP repeat containing 3	3.473263547	2.97E-15
ICA1L	islet cell autoantigen 1 like	-1.293895283	3.02E-15
C1QL1	complement C1q like 1	-0.770872083	3.07E-15
PLPPR3	phospholipid phosphatase related 3	-1.810294321	3.26E-15
NBPF1	NBPF member 1	-0.679320244	3.32E-15
CA14	carbonic anhydrase 14	-2.00305786	3.43E-15
GPC4	glypican 4	-0.717828077	3.46E-15
ABCA13	ATP binding cassette subfamily A member 13	2.587476353	3.51E-15
EML1	EMAP like 1	0.640921281	3.55E-15
TFAM	transcription factor A; mitochondrial	0.729158101	3.65E-15
AKAP12	A-kinase anchoring protein 12	-0.778580686	3.76E-15
MYORG	myogenesis regulating glycosidase (putative)	-0.734550281	3.81E-15
PTPN2	protein tyrosine phosphatase non-receptor type 2	0.815203846	3.95E-15
PNO1	partner of NOB1 homolog	0.878429674	4.05E-15
COX10	cytochrome c oxidase assembly factor heme A:farnesyltransferase COX10	0.759236201	4.09E-15
PKNOX2	PBX/knotted 1 homeobox 2	2.177094083	4.51E-15
PPIF	peptidylprolyl isomerase F	0.674672808	4.64E-15
THG1L	tRNA-histidine guanylyltransferase 1 like	1.006028585	4.79E-15
DLGAP5	DLG associated protein 5	-0.774421503	4.80E-15
MMP16	matrix metallopeptidase 16	-1.052707132	4.84E-15
CEP43	centrosomal protein 43	0.682117051	5.01E-15
GPR137C	G protein-coupled receptor 137C	-1.728843695	5.07E-15
YPEL3	yippee like 3	-0.992336213	5.07E-15
MEGF8	multiple EGF like domains 8	-0.873544179	5.14E-15
PSMF1	proteasome inhibitor subunit 1	0.692910617	5.17E-15
MEGF9	multiple EGF like domains 9	-0.796342371	5.32E-15
SLC3A2	solute carrier family 3 member 2	0.896447686	5.32E-15
NR4A1	nuclear receptor subfamily 4 group A member 1	-1.427789114	5.44E-15
LRRC20	leucine rich repeat containing 20	-0.597841904	5.57E-15
GPBP1	GC-rich promoter binding protein 1	0.615575913	5.81E-15
PUS7L	pseudouridine synthase 7 like	0.667157164	5.83E-15
LRP1	LDL receptor related protein 1	-1.097607398	5.90E-15
S100A11	S100 calcium binding protein A11	0.591179623	5.92E-15
COQ8A	coenzyme Q8A	0.698747933	5.97E-15
PLEKHF1	pleckstrin homology and FYVE domain containing 1	0.832244941	6.06E-15
LIN7A	lin-7 homolog A; crumbs cell polarity complex component	-1.593015293	6.22E-15
ALG2	ALG2 alpha-1;3/1;6-mannosyltransferase	0.666118669	6.30E-15

FABP5	fatty acid binding protein 5	0.873675907	6.36E-15
RGS6	regulator of G protein signaling 6	-0.771648831	6.37E-15
ZMYND10	zinc finger MYND-type containing 10	-1.32037201	6.75E-15
GAR1	GAR1 ribonucleoprotein	0.593058947	6.76E-15
BCOR	BCL6 corepressor	0.812356071	7.09E-15
CDK5R1	cyclin dependent kinase 5 regulatory subunit 1	-0.86916161	7.27E-15
RCBTB2	RCC1 and BTB domain containing protein 2	-1.138008741	7.45E-15
MOB1B	MOB kinase activator 1B	-0.705659194	7.57E-15
UTP25	UTP25 small subunit processome component	0.690494948	7.67E-15
CLEC19A	C-type lectin domain containing 19A	-1.312000845	7.87E-15
CEP70	centrosomal protein 70	-1.131602446	7.88E-15
SBK1	SH3 domain binding kinase 1	0.79432824	7.88E-15
CXADR	CXADR Ig-like cell adhesion molecule	-0.59205164	7.95E-15
PHLDA2	pleckstrin homology like domain family A member 2	1.292424522	8.05E-15
LASP1	LIM and SH3 protein 1	-0.913448315	8.06E-15
NFIB	nuclear factor I B	-0.597188437	8.25E-15
ATF5	activating transcription factor 5	0.70560188	8.44E-15
PDLIM2	PDZ and LIM domain 2	-0.955570333	8.95E-15
ERO1A	endoplasmic reticulum oxidoreductase 1 alpha	0.661922633	9.35E-15
NXN	nucleoredoxin	-0.896766565	9.55E-15
INSYN2A	inhibitory synaptic factor 2A	1.101139446	9.77E-15
ARHGAP19	Rho GTPase activating protein 19	-0.701659564	1.05E-14
HEXD	hexosaminidase D	1.040108858	1.06E-14
FN3K	fructosamine 3 kinase	-0.890949054	1.07E-14
MAP1LC3A	microtubule associated protein 1 light chain 3 alpha	-1.019744954	1.16E-14
TM7SF2	transmembrane 7 superfamily member 2	-0.840661136	1.18E-14
INHBA	inhibin subunit beta A	4.331285712	1.19E-14
CHCHD4	coiled-coil-helix-coiled-coil-helix domain containing 4	0.85733693	1.23E-14
SORT1	sortilin 1	-0.641005023	1.28E-14
ITPRIP	inositol 1;4;5-trisphosphate receptor interacting protein	0.849724547	1.33E-14
FAM114A1	family with sequence similarity 114 member A1	-1.031101974	1.34E-14
SYNC	syncoilin; intermediate filament protein	-1.255906012	1.35E-14
SORBS2	sorbin and SH3 domain containing 2	-0.999649831	1.36E-14
XRN1	5'-3' exoribonuclease 1	1.3768767	1.37E-14
A2M	alpha-2-macroglobulin	-0.780189907	1.45E-14
STMN1	stathmin 1	-0.801978198	1.46E-14

MTHFD2	methylenetetrahydrofolate dehydrogenase (NADP+ dependent) 2; methenyltetrahydrofolate cyclohydrolase	0.631912209	1.46E-14
PUS1	pseudouridine synthase 1	0.729267915	1.62E-14
TENT4B	terminal nucleotidyltransferase 4B	0.926436711	1.63E-14
PLEKHG5	pleckstrin homology and RhoGEF domain containing G5	-0.878168707	1.74E-14
ARVCF	ARVCF delta catenin family member	-0.847366326	1.89E-14
SPON2	spondin 2	2.142270061	1.91E-14
LIMCH1	LIM and calponin homology domains 1	-0.679122623	1.92E-14
FUT4	fucosyltransferase 4	1.632290746	1.99E-14
TXNIP	thioredoxin interacting protein	0.665441298	1.99E-14
CDH24	cadherin 24	-1.15420408	2.24E-14
SP6	Sp6 transcription factor	4.565912532	2.29E-14
USP31	ubiquitin specific peptidase 31	0.632564927	2.41E-14
PTPN21	protein tyrosine phosphatase non-receptor type 21	-0.69836644	2.45E-14
ATPSCKMT	ATP synthase c subunit lysine N-methyltransferase	-1.20307218	2.50E-14
FOXC1	forkhead box C1	0.78716177	2.54E-14
ETS1	ETS proto-oncogene 1; transcription factor	2.453712318	2.60E-14
PHF2	PHD finger protein 2	-0.656819001	2.79E-14
COL11A1	collagen type XI alpha 1 chain	-0.962830648	2.81E-14
C2orf72	chromosome 2 open reading frame 72	-2.263199266	2.88E-14
NGDN	neuroguidin	0.750913692	2.88E-14
VSIG10L	V-set and immunoglobulin domain containing 10 like	1.669231842	2.94E-14
DENND2D	DENN domain containing 2D	2.423639617	2.97E-14
RAB42	RAB42; member RAS oncogene family	1.831636438	3.04E-14
H2BC5	H2B clustered histone 5	1.146430794	3.05E-14
SOAT1	sterol O-acyltransferase 1	0.737991001	3.06E-14
YJU2	YJU2 splicing factor homolog	0.655983048	3.10E-14
ANKRD1	ankyrin repeat domain 1	-1.357100095	3.12E-14
DCUN1D5	defective in cullin neddylation 1 domain containing 5	0.70089953	3.20E-14
FBXO31	F-box protein 31	0.625488202	3.30E-14
TRIB1	tribbles pseudokinase 1	0.798497728	3.33E-14
RUSC2	RUN and SH3 domain containing 2	-0.859109037	3.64E-14
SCD5	stearoyl-CoA desaturase 5	-0.905042528	3.65E-14
ITPRIPL2	ITPRIP like 2	0.813130122	3.74E-14
TBC1D16	TBC1 domain family member 16	-0.653933641	3.76E-14
XPO4	exportin 4	0.602080577	3.79E-14
SPTLC2	serine palmitoyltransferase long chain base subunit 2	0.673034375	4.00E-14

ORAI1	ORAI calcium release-activated calcium modulator 1	0.796602052	4.07E-14
HEATR5A	HEAT repeat containing 5A	-0.81535542	4.49E-14
LIMK2	LIM domain kinase 2	0.881959826	4.53E-14
CAB39L	calcium binding protein 39 like	-1.261058245	4.71E-14
IPPK	inositol-pentakisphosphate 2-kinase	0.708935813	4.77E-14
YRDC	yrdC N6-threonylcarbamoyltransferase domain containing	0.966696421	5.09E-14
FAM222A	family with sequence similarity 222 member A	-1.144270882	5.38E-14
PPARG	peroxisome proliferator activated receptor gamma	-1.351521751	5.38E-14
MRPL2	mitochondrial ribosomal protein L2	0.636329645	5.58E-14
KYNU	kynureninase	4.720301702	5.59E-14
EIF2S1	eukaryotic translation initiation factor 2 subunit alpha	0.654128567	5.79E-14
TPRA1	transmembrane protein adipocyte associated 1	0.658425854	5.82E-14
CPE	carboxypeptidase E	-1.013840125	6.04E-14
ASS1	argininosuccinate synthase 1	0.762572822	6.54E-14
MMP25	matrix metallopeptidase 25	1.536976283	6.83E-14
FTH1	ferritin heavy chain 1	0.858672102	6.86E-14
SLCO5A1	solute carrier organic anion transporter family member 5A1	1.718255806	6.86E-14
CERCAM	cerebral endothelial cell adhesion molecule	-0.656492113	6.87E-14
C1QTNF5	C1q and TNF related 5	-1.647848718	6.88E-14
USP25	ubiquitin specific peptidase 25	0.954373101	6.92E-14
MRM3	mitochondrial rRNA methyltransferase 3	0.725468648	6.92E-14
FAM117A	family with sequence similarity 117 member A	0.669672957	7.40E-14
SMOC1	SPARC related modular calcium binding 1	-0.767169398	7.88E-14
XPR1	xenotropic and polytropic retrovirus receptor 1	-0.605936492	7.99E-14
SLC1A2	solute carrier family 1 member 2	-0.718872009	8.02E-14
CCNJ	cyclin J	0.612763693	8.02E-14
FAM219B	family with sequence similarity 219 member B	-0.676210844	8.44E-14
C1RL	complement C1r subcomponent like	1.07139906	8.49E-14
SLC25A37	solute carrier family 25 member 37	0.597812425	8.73E-14
GPATCH4	G-patch domain containing 4 (gene/pseudogene)	0.709440366	9.00E-14
NOP16	NOP16 nucleolar protein	0.637045152	9.29E-14
CSNK1G1	casein kinase 1 gamma 1	0.724062139	9.35E-14
SLC44A5	solute carrier family 44 member 5	-1.640014778	9.45E-14

ZNF436	zinc finger protein 436	0.606165779	9.58E-14
MYADM	myeloid associated differentiation marker	-0.622803449	9.65E-14
NBEA	neurobeachin	-0.949751505	1.02E-13
EMC7	ER membrane protein complex subunit 7	0.83144178	1.02E-13
RDH13	retinol dehydrogenase 13	0.81861401	1.03E-13
POLR1G	RNA polymerase I subunit G	0.67159303	1.05E-13
NOD2	nucleotide binding oligomerization domain containing 2	6.81933789	1.10E-13
BBC3	BCL2 binding component 3	0.622302121	1.10E-13
KLHDC8B	kelch domain containing 8B	-0.710157848	1.13E-13
PLSCR3	phospholipid scramblase 3	-0.816843815	1.14E-13
RCC1	regulator of chromosome condensation 1	0.673582851	1.14E-13
TENT5B	terminal nucleotidyltransferase 5B	-1.126734731	1.15E-13
BCAR3	BCAR3 adaptor protein; NSP family member	-0.710828566	1.17E-13
ID1	inhibitor of DNA binding 1	-1.224978657	1.18E-13
TMPRSS5	transmembrane serine protease 5	-1.73810888	1.22E-13
MIR9-1HG	MIR9-1 host gene	-1.134671033	1.27E-13
HSPA9	heat shock protein family A (Hsp70) member 9	0.630997225	1.35E-13
THBS3	thrombospondin 3	-0.927980027	1.35E-13
TMT1B	thiol methyltransferase 1B	-0.684340685	1.38E-13
DNAAF9	dynein axonemal assembly factor 9	-0.850770145	1.45E-13
HABP4	hyaluronan binding protein 4	-0.992173766	1.45E-13
SLC43A3	solute carrier family 43 member 3	3.201287822	1.49E-13
NTRK2	neurotrophic receptor tyrosine kinase 2	1.498457477	1.53E-13
DES	desmin	2.417120486	1.57E-13
C14orf132	chromosome 14 open reading frame 132	-1.582212138	1.59E-13
EFNB1	ephrin B1	-0.728381616	1.59E-13
DLX2	distal-less homeobox 2	-0.713882962	1.67E-13
COL9A3	collagen type IX alpha 3 chain	-0.669240621	1.71E-13
TMTC4	transmembrane O-mannosyltransferase targeting cadherins 4	-1.07567418	1.73E-13
BST1	bone marrow stromal cell antigen 1	-0.775779657	1.75E-13
PLCL2	phospholipase C like 2	0.811643121	1.75E-13
COL14A1	collagen type XIV alpha 1 chain	-1.108151592	1.79E-13
AQP4	aquaporin 4	-1.17179535	1.95E-13
SLIT3	slit guidance ligand 3	-1.287751568	1.97E-13
SRXN1	sulfiredoxin 1	0.590611347	2.03E-13
CACNG4	calcium voltage-gated channel auxiliary subunit gamma 4	-0.621084092	2.06E-13
PSMC4	proteasome 26S subunit; ATPase 4	0.609009439	2.06E-13
PDE2A	phosphodiesterase 2A	-0.72864878	2.16E-13

TMEM106C	transmembrane protein 106C	-0.653630981	2.18E-13
CASP8	caspase 8	1.569339263	2.23E-13
ZBTB42	zinc finger and BTB domain containing 42	0.946572956	2.24E-13
C11orf87	chromosome 11 open reading frame 87	-1.751353655	2.27E-13
SEPTIN7	septin 7	-0.599834429	2.33E-13
TBC1D5	TBC1 domain family member 5	-0.611869458	2.38E-13
GCA	grancalcin	1.253828443	2.48E-13
SOX8	SRY-box transcription factor 8	-0.619628867	2.51E-13
DEAF1	DEAF1 transcription factor	0.690753646	2.56E-13
KLF12	KLF transcription factor 12	-1.154344525	2.68E-13
SPEG	striated muscle enriched protein kinase	-0.802863287	2.73E-13
DUS3L	dihydrouridine synthase 3 like	0.832384467	2.76E-13
PVR	PVR cell adhesion molecule	0.622862172	2.94E-13
NPTXR	neuronal pentraxin receptor	-1.278458332	2.95E-13
PLEKHH1	pleckstrin homology; MyTH4 and FERM domain containing H1	-1.702374004	3.04E-13
MSI1	musashi RNA binding protein 1	-0.633131522	3.14E-13
YARS2	tyrosyl-tRNA synthetase 2	0.686814424	3.24E-13
RNF213-AS1	RNF213 antisense RNA 1	-3.647373266	3.32E-13
NPIPP1	nuclear pore complex interacting protein pseudogene 1	0.836017465	3.50E-13
HGSNAT	heparan-alpha-glucosaminide N-acetyltransferase	-0.736089196	3.64E-13
RBM47	RNA binding motif protein 47	1.303668254	3.90E-13
IL1R1	interleukin 1 receptor type 1	1.750129458	4.01E-13
MFAP4	microfibril associated protein 4	-1.148727232	4.07E-13
TMEM231	transmembrane protein 231	0.831244365	4.09E-13
SIDT2	SID1 transmembrane family member 2	-0.664480732	4.42E-13
INKA2	inka box actin regulator 2	-0.891139198	4.73E-13
SAA1	serum amyloid A1	5.810769679	4.93E-13
GADD45G	growth arrest and DNA damage inducible gamma	-1.451132523	4.99E-13
SLPI	secretory leukocyte peptidase inhibitor	5.033735792	5.13E-13
KCNA2	potassium voltage-gated channel subfamily A member 2	-2.293800251	5.39E-13
PALB2	partner and localizer of BRCA2	0.613046439	5.40E-13
TIMM50	translocase of inner mitochondrial membrane 50	0.59579416	5.49E-13
DTX4	deltex E3 ubiquitin ligase 4	0.817475913	5.49E-13
PARP8	poly(ADP-ribose) polymerase family member 8	3.907885121	5.60E-13
NSMCE4A	NSE4 homolog A; SMC5-SMC6 complex component	0.653886419	5.71E-13
ENY2	ENY2 transcription and export complex 2 subunit	0.634632645	5.80E-13

ATOSA	atos homolog A	-0.675721973	5.96E-13
FAM182A	family with sequence similarity 182 member A	-1.603787497	6.03E-13
PNRC1	proline rich nuclear receptor coactivator 1	0.61360212	6.12E-13
STK24	serine/threonine kinase 24	0.654362409	6.18E-13
RFTN2	raftlin family member 2	-2.211930992	6.30E-13
CNTNAP3	contactin associated protein family member 3	-1.057860699	6.30E-13
GSTM2	glutathione S-transferase mu 2	-1.148580909	6.31E-13
GRPEL2	GrpE like 2; mitochondrial	0.747636412	6.51E-13
SMAP1	small ArfGAP 1	-0.605597222	6.71E-13
OPHN1	oligophrenin 1	-1.487909621	6.89E-13
SDC1	syndecan 1	-0.734325153	6.91E-13
SFXN5	sideroflexin 5	-0.651805466	6.92E-13
ARHGAP26	Rho GTPase activating protein 26	1.183051583	7.00E-13
NOL4L	nucleolar protein 4 like	-0.95509398	7.37E-13
ROGDI	rogdi atypical leucine zipper	-0.895443802	7.51E-13
GPR176	G protein-coupled receptor 176	-0.697065833	8.15E-13
CIMAP3	ciliary microtubule associated protein 3	-1.1778019	8.24E-13
SMARCC2	SWI/SNF related; matrix associated; actin dependent regulator of chromatin subfamily c member 2	-0.619798157	8.52E-13
REXO4	REX4 homolog; 3'-5' exonuclease	0.651446427	8.76E-13
ZNF107	zinc finger protein 107	0.781110279	8.82E-13
MTCL2	microtubule crosslinking factor 2	-0.816519004	8.83E-13
AIM2	absent in melanoma 2	7.861892297	9.12E-13
UTP4	UTP4 small subunit processome component	0.599172347	9.14E-13
MRAS	muscle RAS oncogene homolog	-1.218112798	9.28E-13
ERVK3-1	endogenous retrovirus group K3 member 1	0.631396672	9.68E-13
ACTG2	actin gamma 2; smooth muscle	-2.033115504	9.94E-13
SLC16A3	solute carrier family 16 member 3	2.848643566	9.98E-13
NFKBIB	NFKB inhibitor beta	0.842286226	1.00E-12
PHLDB1	pleckstrin homology like domain family B member 1	-0.635017136	1.02E-12
DUSP2	dual specificity phosphatase 2	0.813308974	1.06E-12
PGK1	phosphoglycerate kinase 1	0.735830296	1.07E-12
RAB26	RAB26; member RAS oncogene family	-1.898082108	1.08E-12
PTCH1	patched 1	-1.132603342	1.10E-12
COL4A6	collagen type IV alpha 6 chain	-1.040153369	1.10E-12
SLC15A2	solute carrier family 15 member 2	-1.791276219	1.13E-12
NFAT5	nuclear factor of activated T cells 5	0.603024636	1.14E-12
SEMA3F	semaphorin 3F	-1.667657128	1.20E-12

RGMA	repulsive guidance molecule BMP co-receptor a	-0.683690341	1.20E-12
ATP1B2	ATPase Na+/K+ transporting subunit beta 2	-0.929341504	1.22E-12
MLLT3	MLLT3 super elongation complex subunit	0.758363161	1.25E-12
SCAMP1-AS	SCAMP1 antisense RNA 1	1.432072881	1.27E-12
TSC22D1	TSC22 domain family member 1	0.706447491	1.31E-12
DDI2	DNA damage inducible 1 homolog 2	0.635964182	1.32E-12
FITM2	fat storage inducing transmembrane protein 2	-0.813704335	1.34E-12
STIM1	stromal interaction molecule 1	-0.682179463	1.37E-12
CCDC40	coiled-coil domain containing 40	-1.319728619	1.37E-12
SHLD2	shieldin complex subunit 2	0.613132748	1.39E-12
NRBP2	nuclear receptor binding protein 2	-0.926696205	1.42E-12
FZD1	frizzled class receptor 1	-0.653509401	1.50E-12
C3	complement C3	6.693913184	1.52E-12
TMEM168	transmembrane protein 168	-0.666094963	1.52E-12
TLE5	TLE family member 5; transcriptional modulator	-0.622957082	1.54E-12
XRCC3	X-ray repair cross complementing 3	0.624896734	1.54E-12
CAP2	cyclase associated actin cytoskeleton regulatory protein 2	-0.960719835	1.61E-12
CCDC177	coiled-coil domain containing 177	-1.729284737	1.62E-12
USP42	ubiquitin specific peptidase 42	0.604648844	1.70E-12
NECAP1	NECAP endocytosis associated 1	0.8501851	1.78E-12
LTV1	LTV1 ribosome biogenesis factor	0.620570385	1.79E-12
GRHPR	glyoxylate and hydroxypyruvate reductase	-0.610095195	1.85E-12
NUSAP1	nucleolar and spindle associated protein 1	-0.604476419	1.91E-12
POU3F3	POU class 3 homeobox 3	-0.671585448	1.97E-12
RAB7B	RAB7B; member RAS oncogene family	-1.332277057	2.00E-12
CXCL14	C-X-C motif chemokine ligand 14	-0.767423995	2.26E-12
ZIC2	Zic family member 2	-0.771978226	2.30E-12
TRMT6	tRNA methyltransferase 6 non-catalytic subunit	0.620924143	2.39E-12
MTM1	myotubularin 1	-0.842900688	2.55E-12
CBX2	chromobox 2	-0.855348541	2.76E-12
LINC02015	long intergenic non-protein coding RNA 2015	7.420128721	2.76E-12
PGAP1	post-GPI attachment to proteins inositol deacylase 1	-0.664916912	2.78E-12
PYROXD2	pyridine nucleotide-disulphide oxidoreductase domain 2	-1.557376336	2.82E-12
MMUT	methylmalonyl-CoA mutase	-0.667733084	3.09E-12

MYH14	myosin heavy chain 14	-0.908385377	3.19E-12
TEAD3	TEA domain transcription factor 3	-0.646966456	3.21E-12
RAB11B	RAB11B; member RAS oncogene family	-0.590019828	3.34E-12
APH1B	aph-1 homolog B; gamma-secretase subunit	-0.907691888	3.56E-12
PLP1	proteolipid protein 1	-1.641176817	3.72E-12
C21orf58	chromosome 21 open reading frame 58	-0.726394428	3.72E-12
ATP6V1G2	ATPase H+ transporting V1 subunit G2	-1.950020005	3.75E-12
PSMD11	proteasome 26S subunit; non-ATPase 11	0.653668646	3.86E-12
NOL8	nucleolar protein 8	0.634345034	3.92E-12
P2RY11	purinergic receptor P2Y11	0.895592369	3.98E-12
CCDC194	coiled-coil domain containing 194	4.506249205	4.02E-12
HIC1	HIC ZBTB transcriptional repressor 1	1.103693963	4.03E-12
MRPL14	mitochondrial ribosomal protein L14	0.755897553	4.30E-12
EFNA1	ephrin A1	1.020662344	4.40E-12
GDF1	growth differentiation factor 1	-0.738882975	4.44E-12
PFN2	profilin 2	-0.628570669	4.60E-12
SOX1-OT	SOX1 overlapping transcript	-1.809156251	4.73E-12
NKD1	NKD inhibitor of WNT signaling pathway 1	-1.511024117	4.91E-12
BBS2	Bardet-Biedl syndrome 2	-0.717359855	5.01E-12
AK3	adenylate kinase 3	0.601172976	5.12E-12
RGS20	regulator of G protein signaling 20	-0.735574904	5.15E-12
DUSP16	dual specificity phosphatase 16	0.669783945	5.26E-12
CNTN3	contactin 3	-1.032211675	5.35E-12
GPR63	G protein-coupled receptor 63	1.011102043	5.40E-12
GNAI1	G protein subunit alpha i1	0.603148819	5.58E-12
SMURF1	SMAD specific E3 ubiquitin protein ligase 1	0.590238078	5.60E-12
C1QBP	complement C1q binding protein	0.642084526	5.75E-12
ESPN	espin	-1.450099125	5.90E-12
MTSS1	MTSS I-BAR domain containing 1	-0.948479473	6.04E-12
CSAG2	CSAG family member 2	7.433644256	6.19E-12
MPPED2	metallophosphoesterase domain containing 2	-1.593046367	6.34E-12
SOS2	SOS Ras/Rho guanine nucleotide exchange factor 2	-0.685093683	6.36E-12
ZSCAN18	zinc finger and SCAN domain containing 18	-0.622827659	6.41E-12
PLAUR	plasminogen activator; urokinase receptor	0.754452644	6.47E-12
HES1	hes family bHLH transcription factor 1	-0.644837151	6.53E-12
SCHIP1	schwannomin interacting protein 1	-0.597623302	6.56E-12
PDK3	pyruvate dehydrogenase kinase 3	0.78889051	6.56E-12

FHOD3	formin homology 2 domain containing 3	-0.609044039	6.71E-12
EXOC3L1	exocyst complex component 3 like 1	4.901668929	6.96E-12
ARHGEF26	Rho guanine nucleotide exchange factor 26	-0.684240151	7.24E-12
PI3	peptidase inhibitor 3	5.240785506	7.27E-12
SLC34A2	solute carrier family 34 member 2	3.716789789	7.29E-12
BBLN	bublin coiled coil protein	0.703944402	7.43E-12
IFIT2	interferon induced protein with tetratricopeptide repeats 2	11.34214429	7.47E-12
RTL8C	retrotransposon Gag like 8C	-0.680168703	7.69E-12
HSPA1A	heat shock protein family A (Hsp70) member 1A	0.968454619	7.71E-12
DEDD2	death effector domain containing 2	0.732021945	7.75E-12
SPATA6	spermatogenesis associated 6	-1.144914229	7.80E-12
BCKDHB	branched chain keto acid dehydrogenase E1 subunit beta	-0.72844316	7.92E-12
ITGAE	integrin subunit alpha E	-0.956101219	8.08E-12
GLIPR2	GLI pathogenesis related 2	-0.629098214	8.19E-12
MMP24	matrix metallopeptidase 24	-1.696542539	8.52E-12
WDTC1	WD and tetratricopeptide repeats 1	-0.607608651	8.56E-12
C2orf68	chromosome 2 open reading frame 68	-0.735923511	8.70E-12
HINT3	histidine triad nucleotide binding protein 3	-0.602703022	8.78E-12
DDX54	DEAD-box helicase 54	0.597050886	8.87E-12
FEZF1-AS1	FEZF1 antisense RNA 1	-1.552636703	8.93E-12
P4HA1	prolyl 4-hydroxylase subunit alpha 1	0.645979476	9.03E-12
HPRT1	hypoxanthine phosphoribosyltransferase 1	0.783440481	9.14E-12
PLK3	polo like kinase 3	0.753989444	9.18E-12
FAS	Fas cell surface death receptor	0.661781468	9.70E-12
MAP2K5	mitogen-activated protein kinase kinase 5	-0.734503083	9.88E-12
BCL2L14	BCL2 like 14	2.555247677	1.01E-11
IL1B	interleukin 1 beta	5.145429675	1.03E-11
DENND5B	DENN domain containing 5B	-0.779162154	1.09E-11
SNRPB	small nuclear ribonucleoprotein polypeptides B and B1	0.615168802	1.09E-11
MAFF	MAF bZIP transcription factor F	0.720189401	1.12E-11
TMEM178A	transmembrane protein 178A	-1.655788352	1.12E-11
KLHL22	kelch like family member 22	-0.61343466	1.14E-11
CNR1	cannabinoid receptor 1	-1.668639283	1.14E-11
CDC25A	cell division cycle 25A	0.600347376	1.15E-11
PIGW	phosphatidylinositol glycan anchor biosynthesis class W	0.701653672	1.16E-11
PPP1R3C	protein phosphatase 1 regulatory subunit 3C	-1.101312305	1.16E-11

MYO5C	myosin VC	-0.980904191	1.17E-11
MAGED4B	MAGE family member D4B	-0.927139428	1.21E-11
ZSWIM4	zinc finger SWIM-type containing 4	0.854105129	1.21E-11
RUNX2	RUNX family transcription factor 2	0.706565073	1.24E-11
CXCL10	C-X-C motif chemokine ligand 10	7.443924346	1.28E-11
TRMT9B	tRNA methyltransferase 9B (putative)	-1.676412394	1.31E-11
MRPL32	mitochondrial ribosomal protein L32	0.687171066	1.31E-11
RETREG1	reticulophagy regulator 1	-0.989194729	1.37E-11
TGM2	transglutaminase 2	4.890293955	1.44E-11
PCDHA12	protocadherin alpha 12	-1.810791245	1.46E-11
TICAM1	TIR domain containing adaptor molecule 1	0.815191141	1.58E-11
PLXNA4	plexin A4	-1.167683593	1.58E-11
ALDH1A3	aldehyde dehydrogenase 1 family member A3	1.524941435	1.63E-11
APC	APC regulator of WNT signaling pathway	-0.674198318	1.64E-11
OLFM2	olfactomedin 2	-0.718251569	1.66E-11
PTPRZ1	protein tyrosine phosphatase receptor type Z1	-0.698880697	1.73E-11
EBNA1BP2	EBNA1 binding protein 2	0.596802918	1.73E-11
SHISAL1	shisa like 1	-0.989875022	1.74E-11
ABCA2	ATP binding cassette subfamily A member 2	-0.72878736	1.78E-11
CAMKV	CaM kinase like vesicle associated	0.864035691	1.79E-11
PHIP	pleckstrin homology domain interacting protein	-0.602027309	1.83E-11
CDC20	cell division cycle 20	-0.662032661	1.85E-11
HP1BP3	heterochromatin protein 1 binding protein 3	-0.731081368	1.90E-11
SEMA4G	semaphorin 4G	-0.884228123	1.93E-11
TRPM2	transient receptor potential cation channel subfamily M member 2	7.485776993	2.00E-11
PFKM	phosphofructokinase; muscle	-0.660391812	2.03E-11
CRAT	carnitine O-acetyltransferase	-0.670670468	2.09E-11
HOPX	HOP homeobox	-0.881308606	2.09E-11
ANKLE1	ankyrin repeat and LEM domain containing 1	0.637862544	2.12E-11
RNF152	ring finger protein 152	3.944023957	2.18E-11
PPID	peptidylprolyl isomerase D	0.594168086	2.24E-11
CARMIL1	capping protein regulator and myosin 1 linker 1	-0.71284131	2.26E-11
ADORA2B	adenosine A2b receptor	0.618697767	2.29E-11
RHPN2	rhophilin Rho GTPase binding protein 2	0.607138332	2.29E-11
RAB43	RAB43; member RAS oncogene family	0.981522842	2.32E-11
TNFRSF25	TNF receptor superfamily member 25	-1.108898851	2.33E-11

PIF1	PIF1 5'-to-3' DNA helicase	-1.093532112	2.62E-11
DOCK11	dedicator of cytokinesis 11	-1.079590784	2.70E-11
SLC7A6	solute carrier family 7 member 6	0.599548216	2.82E-11
EMG1	EMG1 N1-specific pseudouridine methyltransferase	0.727370736	2.82E-11
MIR4435-2H	MIR4435-2 host gene	-0.633193356	2.83E-11
ZSWIM7	zinc finger SWIM-type containing 7	-0.995545982	2.85E-11
PRRG4	proline rich and Gla domain 4	4.382612623	2.96E-11
KLC4	kinesin light chain 4	-0.62847383	2.97E-11
AP5B1	adaptor related protein complex 5 subunit beta 1	0.641102668	3.18E-11
NKX3-1	NK3 homeobox 1	2.308567001	3.29E-11
SNHG15	small nucleolar RNA host gene 15	0.738805167	3.32E-11
LNX1	ligand of numb-protein X 1	1.542218235	3.34E-11
PROCR	protein C receptor	1.287221626	3.39E-11
PCDH7	protocadherin 7	-0.835451727	3.44E-11
NELL1	neural EGFL like 1	7.637543121	3.47E-11
CHD3	chromodomain helicase DNA binding protein 3	-0.783073349	3.51E-11
CCN1	cellular communication network factor 1	-0.772123869	3.53E-11
TMEM243	transmembrane protein 243	1.063342468	3.60E-11
ST6GALNAC	ST6 N-acetylgalactosaminide alpha-2;6-sialyltransferase 5	-1.214345037	3.61E-11
TSPAN11	tetraspanin 11	-0.598857416	3.74E-11
ZNF614	zinc finger protein 614	0.666397525	3.74E-11
TP53I11	tumor protein p53 inducible protein 11	-1.76343289	3.88E-11
ACSL5	acyl-CoA synthetase long chain family member 5	4.935369991	3.93E-11
ALG3	ALG3 alpha-1;3- mannosyltransferase	0.614804238	3.95E-11
SDC2	syndecan 2	-0.63125332	4.05E-11
ELL2	elongation factor for RNA polymerase II 2	0.904625046	4.07E-11
GSTM3	glutathione S-transferase mu 3	-1.018138335	4.38E-11
PGM2L1	phosphoglucomutase 2 like 1	0.688853971	4.42E-11
EEF1AKMT4	EEF1A lysine methyltransferase 4	1.142072075	4.50E-11
C5	complement C5	-1.474883421	4.50E-11
PRPS2	phosphoribosyl pyrophosphate synthetase 2	0.613754963	4.52E-11
CXCL5	C-X-C motif chemokine ligand 5	6.975767408	4.58E-11
ITGA7	integrin subunit alpha 7	-0.73559321	4.59E-11
C4orf33	chromosome 4 open reading frame 33	1.313082584	4.63E-11
DUSP7	dual specificity phosphatase 7	0.703073173	4.77E-11
PLCD1	phospholipase C delta 1	-0.819276922	4.91E-11
LINC02593	long intergenic non-protein coding RNA 2593	-2.832520638	4.98E-11

GPM6A	glycoprotein M6A	-0.624802562	5.11E-11
ZSCAN16-A:	ZSCAN16 antisense RNA 1	-1.342869523	5.13E-11
PDGFRB	platelet derived growth factor receptor beta	-1.177634636	5.37E-11
NPW	neuropeptide W	0.758995621	5.38E-11
RAB15	RAB15; member RAS oncogene family	-1.140638672	5.67E-11
ABHD4	abhydrolase domain containing 4; N-acyl phospholipase B	-0.768446053	5.71E-11
UTP15	UTP15 small subunit processome component	0.633214265	5.97E-11
BOLA3	bolA family member 3	0.731184367	6.01E-11
LOXL3	lysyl oxidase like 3	-0.815470334	6.14E-11
COL9A2	collagen type IX alpha 2 chain	-1.276653647	6.19E-11
PCDHB10	protocadherin beta 10	-0.896748463	6.39E-11
MICB	MHC class I polypeptide-related sequence B	1.822881661	6.96E-11
PPARGC1B	PPARG coactivator 1 beta	1.099148541	7.01E-11
ZKSCAN1	zinc finger with KRAB and SCAN domains 1	-0.672052174	7.12E-11
PHF5A	PHD finger protein 5A	0.664052001	7.19E-11
ZFHX2	zinc finger homeobox 2	0.982033548	7.43E-11
SOX6	SRY-box transcription factor 6	-1.465306371	7.49E-11
TNFSF9	TNF superfamily member 9	2.486527152	7.57E-11
BCAT2	branched chain amino acid transaminase 2	-0.676345319	7.62E-11
TSFM	Ts translation elongation factor; mitochondrial	0.646275701	8.02E-11
RIN1	Ras and Rab interactor 1	0.590231181	8.02E-11
TMEM59L	transmembrane protein 59 like	-1.041326278	8.09E-11
RNF144B	ring finger protein 144B	3.78704916	8.09E-11
DDX28	DEAD-box helicase 28	0.606140102	8.18E-11
RNASEL	ribonuclease L	2.006194993	8.41E-11
IQGAP3	IQ motif containing GTPase activating protein 3	-1.117444823	8.69E-11
MAP1A	microtubule associated protein 1A	-0.636455868	8.76E-11
BLTP1	bridge-like lipid transfer protein family member 1	-0.863019673	8.82E-11
OR52K3P	olfactory receptor family 52 subfamily K member 3 pseudogene	4.935690987	9.04E-11
CIB2	calcium and integrin binding family member 2	-1.107913532	9.11E-11
POMP	proteasome maturation protein	0.635889721	9.20E-11
SLC9A6	solute carrier family 9 member A6	-0.740873575	9.28E-11
RORB	RAR related orphan receptor B	1.253200965	9.30E-11
NBPF14	NBPF member 14	-0.755966159	9.34E-11
NPM3	nucleophosmin/nucleoplasmin 3	0.815339024	9.44E-11

CTIF	cap binding complex dependent translation initiation factor	-0.590157506	9.51E-11
CPNE5	copine 5	-1.89372519	1.00E-10
DLL3	delta like canonical Notch ligand 3	-0.95897738	1.02E-10
MRPS23	mitochondrial ribosomal protein S23	0.672792072	1.04E-10
POP1	POP1 homolog; ribonuclease P/MRP subunit	0.727383885	1.04E-10
CORO1A	coronin 1A	-1.046206701	1.06E-10
SCUBE3	signal peptide; CUB domain and EGF like domain containing 3	-1.646178593	1.10E-10
CH25H	cholesterol 25-hydroxylase	5.316573332	1.12E-10
TMEM132E	transmembrane protein 132E	-0.926216381	1.13E-10
TLE3	TLE family member 3; transcriptional corepressor	-1.243122443	1.13E-10
GABBR1	gamma-aminobutyric acid type B receptor subunit 1	-0.926272343	1.15E-10
PITPNA-AS1	PITPNA antisense RNA 1	-1.545041556	1.24E-10
LTBP2	latent transforming growth factor beta binding protein 2	-0.743976379	1.26E-10
CLIP3	CAP-Gly domain containing linker protein 3	-0.769516361	1.29E-10
DDRGK1	DDRGK domain containing 1	0.664071729	1.30E-10
NUP153-AS1	NUP153 antisense RNA 1	1.535210322	1.35E-10
WHRN	whirlin	1.14713334	1.37E-10
ADAMTS15	ADAM metallopeptidase with thrombospondin type 1 motif 15	-0.783955443	1.39E-10
PSTPIP2	proline-serine-threonine phosphatase interacting protein 2	0.7290565	1.43E-10
SNCA	synuclein alpha	-0.749705767	1.43E-10
FGD1	FYVE; RhoGEF and PH domain containing 1	-0.630215982	1.43E-10
NR2E1	nuclear receptor subfamily 2 group E member 1	-1.10510109	1.44E-10
LINC01224	long intergenic non-protein coding RNA 1224	-1.319247088	1.44E-10
LMTK3	lemur tyrosine kinase 3	-0.710328289	1.47E-10
SPC24	SPC24 component of NDC80 kinetochore complex	-0.605004717	1.53E-10
MRPL17	mitochondrial ribosomal protein L17	0.646597792	1.55E-10
NRGN	neurogranin	-1.116147656	1.56E-10
FZD9	frizzled class receptor 9	1.123929089	1.57E-10
TRIT1	tRNA isopentenyltransferase 1	0.625289809	1.57E-10
FAM111A-DT	FAM111A divergent transcript	1.492165276	1.57E-10
ALDH3A1	aldehyde dehydrogenase 3 family member A1	-1.899372653	1.63E-10
RRP7A	ribosomal RNA processing 7 homolog A	0.681994543	1.63E-10

EVL	Enah/Vasp-like	-0.605580471	1.64E-10
DUSP9	dual specificity phosphatase 9	0.827678661	1.64E-10
UST	uronyl 2-sulfotransferase	0.612582394	1.66E-10
LYN	LYN proto-oncogene; Src family tyrosine kinase	1.465032864	1.70E-10
ARHGEF4	Rho guanine nucleotide exchange factor 4	-0.959121906	1.71E-10
ZDHHC15	zinc finger DHHC-type palmitoyltransferase 15	-1.029317997	1.71E-10
KIAA0232	KIAA0232	-0.608052998	1.73E-10
ANAPC15	anaphase promoting complex subunit 15	-0.771909375	1.74E-10
BPHL	biphenyl hydrolase like	-0.85518601	1.80E-10
VEGFC	vascular endothelial growth factor C	4.274852261	1.84E-10
CTNNA1	catenin alpha 1	-0.601377724	1.85E-10
PAX8-AS1	PAX8 antisense RNA 1	1.914994949	1.90E-10
ARL10	ADP ribosylation factor like GTPase 10	-0.605291796	1.98E-10
PRSS23	serine protease 23	-0.699502362	2.00E-10
NDUFAF4	NADH:ubiquinone oxidoreductase complex assembly factor 4	0.869897108	2.08E-10
MIRLET7IHC	MIRLET7I host gene	-1.559000886	2.11E-10
CYB5RL	cytochrome b5 reductase like	-0.923353475	2.12E-10
MACROD2	mono-ADP ribosylhydrolase 2	-1.877797224	2.34E-10
GEMIN7	gem nuclear organelle associated protein 7	0.727257493	2.35E-10
UAP1L1	UDP-N-acetylglucosamine pyrophosphorylase 1 like 1	-0.972389192	2.37E-10
CLASP1	cytoplasmic linker associated protein 1	-0.78760661	2.38E-10
GRIK1	glutamate ionotropic receptor kainate type subunit 1	-2.998256898	2.51E-10
LYPD1	LY6/PLAUR domain containing 1	0.762609939	2.59E-10
TMEM51	transmembrane protein 51	0.771123135	2.63E-10
S100A3	S100 calcium binding protein A3	1.341264775	2.66E-10
TRNP1	TMF1 regulated nuclear protein 1	-0.679699734	2.67E-10
TTC27	tetratricopeptide repeat domain 27	0.641050235	2.72E-10
CDCA3	cell division cycle associated 3	-0.872433481	2.77E-10
ABCC1	ATP binding cassette subfamily C member 1	0.628918614	2.83E-10
MKI67	marker of proliferation Ki-67	-0.672396389	2.89E-10
ANKRD33B	ankyrin repeat domain 33B	0.710072604	2.90E-10
ZNF358	zinc finger protein 358	-0.594148222	2.90E-10
GALNT5	polypeptide N-acetylgalactosaminyltransferase 5	-1.432926526	2.96E-10
C2CD4A	C2 calcium dependent domain containing 4A	3.465896114	3.01E-10
EPPK1	epiplakin 1	-1.639957013	3.02E-10
MYEF2	myelin expression factor 2	-0.61572309	3.07E-10

IMMP1L	inner mitochondrial membrane peptidase subunit 1	-1.388369969	3.09E-10
PHYH	phytanoyl-CoA 2-hydroxylase	-0.962461338	3.18E-10
PCDHB5	protocadherin beta 5	-1.079088529	3.29E-10
TRMT10C	tRNA methyltransferase 10C; mitochondrial RNase P subunit	0.714473429	3.34E-10
HBA1	hemoglobin subunit alpha 1	1.014656512	3.35E-10
RALYL	RALY RNA binding protein like	-2.017744599	3.35E-10
TPPP3	tubulin polymerization promoting protein family member 3	-1.394971569	3.47E-10
ABR	ABR activator of RhoGEF and GTPase	0.88629667	3.55E-10
ARX	aristaless related homeobox	-0.89553793	3.55E-10
CHST15	carbohydrate sulfotransferase 15	-0.751060686	3.60E-10
CSF2	colony stimulating factor 2	6.027200804	3.62E-10
DAAM1	dishevelled associated activator of morphogenesis 1	-0.832890108	3.65E-10
NFATC2	nuclear factor of activated T cells 2	-1.273109719	3.65E-10
REEP6	receptor accessory protein 6	-0.895244636	3.65E-10
PHLPP1	PH domain and leucine rich repeat protein phosphatase 1	-0.625161144	3.81E-10
C15orf39	chromosome 15 open reading frame 39	0.825481316	3.82E-10
LYSMD2	LysM domain containing 2	0.961168159	3.82E-10
ENTPD1-AS	ENTPD1 antisense RNA 1	0.763293285	3.91E-10
NDRG2	NDRG family member 2	-1.800455437	3.96E-10
DLX5	distal-less homeobox 5	-1.293875861	3.99E-10
STON1-GTF	STON1-GTF2A1L readthrough	-1.270324081	4.14E-10
L2HGDH	L-2-hydroxyglutarate dehydrogenase	0.776667114	4.14E-10
NPB	neuropeptide B	-1.380984153	4.14E-10
CCDC59	coiled-coil domain containing 59	0.79198571	4.21E-10
OLFML2A	olfactomedin like 2A	-0.857176053	4.32E-10
MIR600HG	MIR600 host gene	-2.351701615	4.44E-10
PFDN2	prefoldin subunit 2	0.604187502	4.52E-10
TAF8	TATA-box binding protein associated factor 8	0.712346402	4.67E-10
IQSEC2	IQ motif and Sec7 domain ArfGEF 2	-0.839088913	5.48E-10
PCYOX1	prenylcysteine oxidase 1	-0.627090681	5.49E-10
KLHL18	kelch like family member 18	0.725378065	5.50E-10
DAB2	DAB adaptor protein 2	-1.009638887	5.57E-10
HDAC9	histone deacetylase 9	0.892497509	5.72E-10
RGPD2	RANBP2 like and GRIP domain containing 2	20.31676354	5.91E-10
CYB5R2	cytochrome b5 reductase 2	1.290022305	6.03E-10
CD34	CD34 molecule	1.011814895	6.07E-10
DEPP1	DEPP autophagy regulator 1	1.655363721	6.09E-10
RBBP9	RB binding protein 9; serine hydrolase	-0.610560416	6.12E-10

IL1RAP	interleukin 1 receptor accessory protein	0.929192908	6.17E-10
AMOT	angiomotin	-0.899890217	6.21E-10
ADGRV1	adhesion G protein-coupled receptor V1	-0.861205992	6.86E-10
SKIDA1	SKI/DACH domain containing 1	-0.840106286	6.93E-10
NIBAN1	niban apoptosis regulator 1	0.623090404	6.93E-10
TXNDC16	thioredoxin domain containing 16	-0.906602141	7.19E-10
FSTL1	follistatin like 1	-0.707482102	7.21E-10
AFG2B	AFG2 AAA ATPase homolog B	0.619683358	7.23E-10
CLEC11A	C-type lectin domain containing 11A	-1.57411792	7.34E-10
YDJC	YdjC chitooligosaccharide deacetylase homolog	0.622645736	7.38E-10
GTF2B	general transcription factor IIB	0.758238275	7.54E-10
CCDC74B	coiled-coil domain containing 74B	-1.514768587	7.69E-10
FBLN7	fibulin 7	-0.997424726	7.94E-10
MAD2L1BP	MAD2L1 binding protein	0.638233179	8.49E-10
KCNG1	potassium voltage-gated channel modifier subfamily G member 1	-0.958781447	8.59E-10
PLCE1	phospholipase C epsilon 1	-1.178006784	8.83E-10
NIPBL-DT	NIPBL divergent transcript	-0.639779764	8.86E-10
TRAPPC6A	trafficking protein particle complex subunit 6A	-0.852034162	9.08E-10
CTNNBL1	catenin beta like 1	0.646153353	9.63E-10
IL20RA	interleukin 20 receptor subunit alpha	1.532970502	1.00E-09
IQCN	IQ motif containing N	-1.832973137	1.01E-09
CRHBP	corticotropin releasing hormone binding protein	-1.67959386	1.02E-09
RPP38	ribonuclease P/MRP subunit p38	0.716663922	1.02E-09
DENND5A	DENN domain containing 5A	0.724356672	1.05E-09
BLACAT1	BLACAT1 overlapping LEMD1 locus	-1.381960428	1.08E-09
IPP	intracisternal A particle-promoted polypeptide	-0.973208312	1.13E-09
CLXN	calaxin	-1.411583925	1.13E-09
ADAMTS7	ADAM metallopeptidase with thrombospondin type 1 motif 7	-0.740750843	1.15E-09
RELT	RELT TNF receptor	0.676588284	1.19E-09
PEG10	paternally expressed 10	-0.698866519	1.22E-09
ZBTB21	zinc finger and BTB domain containing 21	0.65672617	1.22E-09
ZNF385C	zinc finger protein 385C	3.747545609	1.23E-09
KAT2A	lysine acetyltransferase 2A	0.592695652	1.24E-09
TMEM198B	transmembrane protein 198B (pseudogene)	-0.868572044	1.25E-09
PRKCA	protein kinase C alpha	-0.631676343	1.26E-09
ALKBH7	alkB homolog 7	-0.809678204	1.27E-09
GSTM4	glutathione S-transferase mu 4	-0.814277264	1.28E-09

CNTNAP3B	contactin associated protein family member 3B	-1.213287693	1.30E-09
SPTAN1	spectrin alpha; non-erythrocytic 1	-0.62415944	1.31E-09
NIT1	nitrilase 1	-0.597309298	1.31E-09
C22orf46P	CTA-216E10,6	-0.750904607	1.35E-09
MICA	MHC class I polypeptide-related sequence A	0.707859479	1.36E-09
SLC45A4	solute carrier family 45 member 4	0.850426609	1.36E-09
PLA2G4A	phospholipase A2 group IVA	4.473705518	1.36E-09
CALB2	calbindin 2	-1.629686079	1.37E-09
RAB11FIP1	RAB11 family interacting protein 1	-0.860699589	1.38E-09
TMEM52	transmembrane protein 52	1.730802202	1.39E-09
CD40	CD40 molecule	4.724306263	1.40E-09
CCNG2	cyclin G2	-0.632113044	1.41E-09
AHRR	aryl hydrocarbon receptor repressor	-2.699574532	1.41E-09
PPM1E	protein phosphatase; Mg2+/Mn2+ dependent 1E	-0.715093499	1.43E-09
DTWD1	DTW domain containing 1	0.631392731	1.46E-09
CDON	cell adhesion associated; oncogene regulated	-0.918576898	1.47E-09
MAP1B	microtubule associated protein 1B	-0.605902589	1.49E-09
SH3BP2	SH3 domain binding protein 2	-0.615411059	1.50E-09
VNN3P	vanin 3; pseudogene	6.200466887	1.52E-09
TLNRD1	talin rod domain containing 1	0.609460014	1.53E-09
ELOVL3	ELOVL fatty acid elongase 3	5.816038886	1.54E-09
PXDN	peroxidasin	0.841106433	1.58E-09
CHI3L1	chitinase 3 like 1	6.847828296	1.63E-09
PC	pyruvate carboxylase	-0.688978048	1.63E-09
KLK10	kallikrein related peptidase 10	4.000025263	1.68E-09
PRR5	proline rich 5	0.73552639	1.68E-09
KCNN3	potassium calcium-activated channel subfamily N member 3	0.682340494	1.71E-09
C4A	complement C4A (Rodgers blood group)	1.078317318	1.74E-09
CHRDL1	chordin like 1	-1.054423825	1.90E-09
PARD6G	par-6 family cell polarity regulator gamma	-0.717914217	1.96E-09
GNPTAB	N-acetylglucosamine-1-phosphate transferase subunits alpha and beta	-0.720690454	1.99E-09
PIGZ	phosphatidylinositol glycan anchor biosynthesis class Z	-1.749005192	2.02E-09
CLEC18A	C-type lectin domain family 18 member A	-1.305196655	2.17E-09
SEMA6C	semaphorin 6C	-1.409164622	2.20E-09
ZSWIM8	zinc finger SWIM-type containing 8	-0.611803623	2.37E-09
SLC12A6	solute carrier family 12 member 6	-1.208938229	2.50E-09
COX3	cytochrome c oxidase subunit III	-0.819579641	2.56E-09

SNTA1	syntrophin alpha 1	-0.677651852	2.57E-09
CFI	complement factor I	-0.730487876	2.64E-09
SOX13	SRY-box transcription factor 13	0.989037716	2.73E-09
MST1	macrophage stimulating 1	-1.215019081	2.73E-09
JPH2	junctophilin 2	-0.787325373	2.75E-09
LOC1027249	uncharacterized LOC102724908	5.868961927	2.77E-09
P2RX5	purinergic receptor P2X 5	1.078372305	2.79E-09
POU2F2	POU class 2 homeobox 2	3.609261116	2.81E-09
SLIT1	slit guidance ligand 1	-1.791400938	2.86E-09
THBS1	thrombospondin 1	-1.47211067	2.87E-09
BIN3	bridging integrator 3	0.663789685	2.88E-09
VGF	VGF nerve growth factor inducible	0.741919393	2.89E-09
HAUS7	HAUS augmin like complex subunit 7	0.707581881	3.04E-09
CXCL12	C-X-C motif chemokine ligand 12	1.272434114	3.10E-09
VPS13B	vacuolar protein sorting 13 homolog B	-0.666532397	3.11E-09
NCALD	neurocalcin delta	-1.16425059	3.13E-09
UTP23	UTP23 small subunit processome component	0.746409547	3.21E-09
GNAZ	G protein subunit alpha z	-0.77495345	3.25E-09
DLG2	discs large MAGUK scaffold protein 2	-1.702175721	3.33E-09
EXOC3L4	exocyst complex component 3 like 4	5.607386259	3.38E-09
RNF169	ring finger protein 169	0.597134345	3.43E-09
RGS3	regulator of G protein signaling 3	0.605807083	3.44E-09
AGBL5	AGBL carboxypeptidase 5	-0.638563251	3.51E-09
PDSS1	decaprenyl diphosphate synthase subunit 1	0.592918625	3.61E-09
SMPD1	sphingomyelin phosphodiesterase 1	-0.781221602	3.62E-09
STARD5	StAR related lipid transfer domain containing 5	2.17908232	3.67E-09
ARHGAP27	Rho GTPase activating protein 27	1.735548128	3.70E-09
ANO10	anoctamin 10	-0.888764581	3.71E-09
SYTL3	synaptotagmin like 3	1.875160335	3.78E-09
BNIP3	BCL2 interacting protein 3	0.782163083	3.79E-09
EFNA5	ephrin A5	-0.657998315	3.83E-09
GLRX2	glutaredoxin 2	0.746720337	3.86E-09
IL1A	interleukin 1 alpha	4.461829666	3.93E-09
MAPK8IP1	mitogen-activated protein kinase 8 interacting protein 1	-0.661740225	3.94E-09
RASGRP1	RAS guanyl releasing protein 1	1.662027076	4.05E-09
VSTM4	V-set and transmembrane domain containing 4	-2.313040797	4.11E-09
LTBP3	latent transforming growth factor beta binding protein 3	-0.746506404	4.11E-09
PCYT1B	phosphate cytidylyltransferase 1B; choline	-0.689937795	4.15E-09

CYP1B1	cytochrome P450 family 1 subfamily B member 1	0.780390874	4.55E-09
CDCP1	CUB domain containing protein 1	1.683100396	4.59E-09
NDUFAF2	NADH:ubiquinone oxidoreductase complex assembly factor 2	0.937313767	4.72E-09
GPATCH3	G-patch domain containing 3	0.684874936	4.90E-09
CTSL	cathepsin L	0.629477174	4.99E-09
PDE3A	phosphodiesterase 3A	-1.435800387	5.02E-09
TPD52	tumor protein D52	0.691017031	5.10E-09
MCHR1	melanin concentrating hormone receptor 1	4.58803893	5.45E-09
QRICH2	glutamine rich 2	-1.269660414	5.47E-09
JMJD6	jumonji domain containing 6; arginine demethylase and lysine hydroxylase	0.594402549	5.51E-09
CPNE4	copine 4	-0.77558526	5.78E-09
CSAG1	chondrosarcoma associated gene 1	7.164441742	5.86E-09
STON1	stonin 1	-0.671556612	5.90E-09
MATK	megakaryocyte-associated tyrosine kinase	0.778297142	5.98E-09
SCRN3	secernin 3	-0.620312331	6.04E-09
NRM	nurim	-0.613139967	6.42E-09
TNS2	tensin 2	-1.283618319	6.44E-09
ADAMTS8	ADAM metallopeptidase with thrombospondin type 1 motif 8	1.187225192	6.51E-09
BLOC1S1	biogenesis of lysosomal organelles complex 1 subunit 1	-0.603983683	6.60E-09
ST7	suppression of tumorigenicity 7	0.639345973	6.71E-09
IL23A	interleukin 23 subunit alpha	3.919185097	6.75E-09
GGT5	gamma-glutamyltransferase 5	3.931615871	7.17E-09
VPS37D	VPS37D subunit of ESCRT-I	-1.115665592	7.32E-09
SNX24	sorting nexin 24	-0.608884741	7.39E-09
TMEM268	transmembrane protein 268	0.778539362	7.58E-09
ZBTB2	zinc finger and BTB domain containing 2	0.601076763	7.64E-09
POLQ	DNA polymerase theta	0.667733941	7.82E-09
CXCL8	C-X-C motif chemokine ligand 8	6.881655678	7.86E-09
PAPPA	pappalysin 1	1.970237136	7.86E-09
GSTA4	glutathione S-transferase alpha 4	-0.643942448	7.89E-09
BMAL2	basic helix-loop-helix ARNT like 2	0.648830272	7.99E-09
SEMA6D	semaphorin 6D	-0.87834682	8.22E-09
LINC02035	long intergenic non-protein coding RNA 2035	-1.172478033	8.58E-09
KIAA1549L	KIAA1549 like	-0.733538425	8.79E-09
HOGA1	4-hydroxy-2-oxoglutarate aldolase 1	-1.561861466	8.79E-09
EXT1	exostosin glycosyltransferase 1	0.637194401	8.99E-09
CACNA1E	calcium voltage-gated channel subunit alpha1 E	-1.180991753	9.05E-09

NEBL	nebulette	-1.504654447	9.17E-09
BMP8B	bone morphogenetic protein 8b	-0.813662572	9.26E-09
CTXND1	cortexin domain containing 1	-1.842796419	9.29E-09
NNT	nicotinamide nucleotide transhydrogenase	-0.662438535	9.31E-09
BDH2	3-hydroxybutyrate dehydrogenase 2	-0.635406522	9.56E-09
PROSER2	proline and serine rich 2	0.821513394	9.71E-09
GDF10	growth differentiation factor 10	-2.503539624	9.76E-09
NANOS1	nanos C2HC-type zinc finger 1	-1.202550909	9.80E-09
LCN2	lipocalin 2	7.097301441	1.01E-08
MRPS12	mitochondrial ribosomal protein S12	0.641152764	1.02E-08
FBXL2	F-box and leucine rich repeat protein 2	-0.878374915	1.04E-08
PELO	pelota mRNA surveillance and ribosome rescue factor	0.668996395	1.05E-08
ST3GAL5	ST3 beta-galactoside alpha-2;3-sialyltransferase 5	-1.187092361	1.05E-08
APOBEC3D	apolipoprotein B mRNA editing enzyme catalytic subunit 3D	3.085903244	1.05E-08
EXOSC5	exosome component 5	0.768795097	1.08E-08
NOPCHAP1	NOP protein chaperone 1	0.913324552	1.08E-08
C12orf76	chromosome 12 open reading frame 76	-0.712269325	1.09E-08
SLC38A3	solute carrier family 38 member 3	-0.952422059	1.09E-08
DAZAP1	DAZ associated protein 1	0.600497835	1.12E-08
PCSK6	proprotein convertase subtilisin/kexin type 6	1.035924403	1.12E-08
LOC1019295	uncharacterized LOC101929563	-2.876765398	1.13E-08
PRKCQ	protein kinase C theta	2.026067344	1.16E-08
TMEFF2	transmembrane protein with EGF like and two follistatin like domains 2	-0.941662504	1.16E-08
CASQ1	calsequestrin 1	-1.698612673	1.17E-08
SLCO3A1	solute carrier organic anion transporter family member 3A1	-0.930685797	1.19E-08
FBXL14	F-box and leucine rich repeat protein 14	0.67309718	1.22E-08
SAT2	spermidine/spermine N1-acetyltransferase family member 2	-0.903611954	1.22E-08
ATAD3B	ATPase family AAA domain containing 3B	0.599008828	1.23E-08
KLHDC7B	kelch domain containing 7B	7.027463166	1.25E-08
MAP4K2	mitogen-activated protein kinase kinase kinase kinase 2	-0.674285875	1.26E-08
ASRGL1	asparaginase and isoaspartyl peptidase 1	-0.870459104	1.26E-08
EEF1E1	eukaryotic translation elongation factor 1 epsilon 1	0.615118782	1.26E-08
ULK1	unc-51 like autophagy activating kinase 1	-0.705602074	1.28E-08

CDK19	cyclin dependent kinase 19	-0.738352748	1.28E-08
FRAT2	FRAT regulator of WNT signaling pathway 2	0.754453076	1.32E-08
DUSP1	dual specificity phosphatase 1	0.793184939	1.38E-08
COMMD6	COMM domain containing 6	-0.599672461	1.38E-08
YPEL1	yippee like 1	-1.634082035	1.38E-08
RUNX3	RUNX family transcription factor 3	4.445445388	1.40E-08
MAOB	monoamine oxidase B	-1.3267204	1.41E-08
PLXNA3	plexin A3	-0.865407808	1.42E-08
ARHGEF17	Rho guanine nucleotide exchange factor 17	-0.737705233	1.44E-08
GTPBP10	GTP binding protein 10	0.685691275	1.47E-08
ZNF587B	zinc finger protein 587B	0.629262275	1.50E-08
GPD1	glycerol-3-phosphate dehydrogenase 1	-1.652455762	1.52E-08
IFT27	intraflagellar transport 27	-0.681158678	1.52E-08
VPS9D1-AS	VPS9D1 antisense RNA 1	0.731841979	1.53E-08
TEK	TEK receptor tyrosine kinase	-1.515442873	1.54E-08
PERP	p53 apoptosis effector related to PMP22	-0.752594978	1.54E-08
HECW1	HECT; C2 and WW domain containing E3 ubiquitin protein ligase 1	-0.669012314	1.55E-08
TRIM5	tripartite motif containing 5	1.094525123	1.57E-08
SRRD	SRR1 domain containing	0.760770214	1.58E-08
EPB41L3	erythrocyte membrane protein band 4,1 like 3	-0.591221367	1.60E-08
KLHDC9	kelch domain containing 9	-1.487129086	1.62E-08
LINC00863	long intergenic non-protein coding RNA 863	-0.829342733	1.62E-08
TRPV3	transient receptor potential cation channel subfamily V member 3	-1.344362933	1.62E-08
IL6	interleukin 6	5.982968793	1.63E-08
LTB4R2	leukotriene B4 receptor 2	1.212288411	1.78E-08
TMEM94	transmembrane protein 94	-0.658907507	1.82E-08
EMP2	epithelial membrane protein 2	-0.630439822	1.84E-08
SLC22A23	solute carrier family 22 member 23	0.636313002	1.85E-08
DNAJC6	DnaJ heat shock protein family (Hsp40) member C6	0.655793157	1.88E-08
SWI5	SWI5 homologous recombination repair protein	-0.837436961	1.91E-08
ZNF296	zinc finger protein 296	1.278566653	2.05E-08
NIN	ninein	-0.674223079	2.17E-08
NYNRIN	NYN domain and retroviral integrase containing	-1.721033686	2.25E-08
MFAP3L	microfibril associated protein 3 like	1.000182267	2.25E-08
FCHO1	FCH and mu domain containing endocytic adaptor 1	0.879414728	2.36E-08
PDK2	pyruvate dehydrogenase kinase 2	-0.659242535	2.38E-08

FASTKD3	FAST kinase domains 3	0.763421834	2.44E-08
DST	dystonin	-0.689945268	2.46E-08
RAB6B	RAB6B; member RAS oncogene family	-1.08147571	2.50E-08
STARD13	StAR related lipid transfer domain containing 13	-0.708333747	2.56E-08
C21orf91	chromosome 21 open reading frame 91	0.700864337	2.57E-08
HHAT	hedgehog acyltransferase	-0.861762691	2.72E-08
SFRP5	secreted frizzled related protein 5	3.68033088	2.74E-08
AMOTL2	angiomotin like 2	-0.672608936	2.75E-08
AIRE	autoimmune regulator	6.662237372	2.77E-08
MMP13	matrix metallopeptidase 13	6.770225481	2.82E-08
TTLL3	tubulin tyrosine ligase like 3	-0.856587211	2.84E-08
SLC2A4	solute carrier family 2 member 4	2.011981795	2.85E-08
TMEM150A	transmembrane protein 150A	-0.678211856	2.89E-08
POU3F1	POU class 3 homeobox 1	0.692304488	2.91E-08
KLF5	KLF transcription factor 5	0.611482102	2.92E-08
CCS	copper chaperone for superoxide dismutase	-0.744318973	3.00E-08
STK32B	serine/threonine kinase 32B	-0.744488225	3.05E-08
STEAP1	STEAP family member 1	1.387195626	3.11E-08
PRC1	protein regulator of cytokinesis 1	-0.674023409	3.19E-08
RIMS2	regulating synaptic membrane exocytosis 2	2.25290835	3.19E-08
SPTBN5	spectrin beta; non-erythrocytic 5	1.433794246	3.20E-08
VGLL3	vestigial like family member 3	-1.733904594	3.22E-08
CELSR2	cadherin EGF LAG seven-pass G-type receptor 2	-0.634547888	3.24E-08
GINS3	GINS complex subunit 3	0.593984185	3.27E-08
ERMAP	erythroblast membrane associated protein (Scianna blood group)	-0.726520147	3.30E-08
DCT	dopachrome tautomerase	-2.310265855	3.38E-08
TOE1	target of EGR1; exonuclease	0.723414067	3.49E-08
PPP2R3A	protein phosphatase 2 regulatory subunit B''alpha	-0.918117478	3.49E-08
MSRB2	methionine sulfoxide reductase B2	-0.940613159	3.52E-08
HHEX	hematopoietically expressed homeobox	0.814201772	3.57E-08
FLNC	filamin C	-0.920312569	3.58E-08
TNIK	TRAF2 and NCK interacting kinase	-0.730998431	3.81E-08
ERMP1	endoplasmic reticulum metallopeptidase 1	-0.707021911	3.86E-08
ERBB4	erb-b2 receptor tyrosine kinase 4	-0.739056501	3.96E-08
MRM1	mitochondrial rRNA methyltransferase 1	0.737830607	4.01E-08
SELENBP1	selenium binding protein 1	-0.867213383	4.10E-08
APOBEC3C	apolipoprotein B mRNA editing enzyme catalytic subunit 3C	0.820166786	4.12E-08

CRNDE	colorectal neoplasia differentially expressed	-0.60960528	4.18E-08
PDZK1IP1	PDZK1 interacting protein 1	6.085785521	4.20E-08
NAA20	N-alpha-acetyltransferase 20; NatB catalytic subunit	0.590888223	4.20E-08
FLG	filaggrin	-0.883091918	4.23E-08
LRRIQ1	leucine rich repeats and IQ motif containing 1	-1.841675065	4.24E-08
PLEKHA6	pleckstrin homology domain containing A6	-1.470860852	4.25E-08
FAM216A	family with sequence similarity 216 member A	0.667529577	4.35E-08
RTL5	retrotransposon Gag like 5	-0.840047574	4.40E-08
PRODH	proline dehydrogenase 1	-1.063688453	4.48E-08
PDE6D	phosphodiesterase 6D	-0.661037616	4.48E-08
ATP1A3	ATPase Na+/K+ transporting subunit alpha 3	0.749524446	4.66E-08
JAKMIP2-AS	JAKMIP2 antisense RNA 1	0.666740598	4.69E-08
LINC01109	long intergenic non-protein coding RNA 1109	-0.994125494	4.77E-08
ANKRD34B	ankyrin repeat domain 34B	-1.420673108	4.79E-08
ROR1	receptor tyrosine kinase like orphan receptor 1	-1.870249607	4.82E-08
BCAN	brevican	-2.064480472	4.90E-08
SPACA9	sperm acrosome associated 9	-1.067145036	4.90E-08
MMS22L	MMS22 like; DNA repair protein	1.048351663	5.03E-08
KDSR	3-ketodihydrosphingosine reductase	0.728055183	5.06E-08
CLUH	clustered mitochondria homolog	0.66850958	5.15E-08
SLC35F2	solute carrier family 35 member F2	0.725276458	5.41E-08
STXBP2	syntaxin binding protein 2	1.603384349	5.41E-08
MAPK11	mitogen-activated protein kinase 11	-0.71497869	5.44E-08
PTPRD	protein tyrosine phosphatase receptor type D	-0.921013737	5.45E-08
UNC80	unc-80 homolog; NALCN channel complex subunit	-2.206590251	5.46E-08
WNT5A	Wnt family member 5A	2.103133794	5.53E-08
SGF29	SAGA complex associated factor 29	0.617683807	5.55E-08
GOLGA6L17	golgin A6 family like 17; pseudogene	-1.324123816	5.59E-08
H2BC26	H2B clustered histone 26	2.137116495	5.64E-08
DENND1C	DENN domain containing 1C	1.721047804	5.69E-08
EEPD1	endonuclease/exonuclease/phosphatase family domain containing 1	-1.257504541	5.80E-08
JMJD7-PLA2	JMJD7-PLA2G4B readthrough	-1.364406645	5.97E-08
ATOSB	atos homolog B	-0.633479605	6.08E-08
ERBIN	erbb2 interacting protein	-0.690103451	6.09E-08
CDAN1	codanin 1	-0.617606182	6.19E-08

CADPS2	calcium dependent secretion activator 2	3.142111995	6.29E-08
AS3MT	arsenite methyltransferase	-1.269773369	6.56E-08
KBTBD8	kelch repeat and BTB domain containing 8	1.17749278	6.81E-08
DIPK1C	divergent protein kinase domain 1C	-1.761552807	7.03E-08
ECSIT	ECSIT signaling integrator	0.614569668	7.19E-08
LINC00052	long intergenic non-protein coding RNA 52	-1.219099671	7.80E-08
MTCL1	microtubule crosslinking factor 1	-0.674532852	8.55E-08
PFKFB4	6-phosphofructo-2-kinase/fructose-2;6-biphosphatase 4	0.633987711	8.60E-08
ATF1	activating transcription factor 1	0.721073243	8.80E-08
C2orf74-DT	C2orf74 divergent transcript	-0.805719729	8.86E-08
RUFY3	RUN and FYVE domain containing 3	-0.595721764	8.98E-08
POLR2H	RNA polymerase II; I and III subunit H	0.650231138	9.24E-08
CLTCL1	clathrin heavy chain like 1	-1.098774169	9.25E-08
ELF3	E74 like ETS transcription factor 3	4.74708212	9.31E-08
TMEFF1	transmembrane protein with EGF like and two follistatin like domains 1	-0.9755375	9.31E-08
SLCO4A1	solute carrier organic anion transporter family member 4A1	0.646118622	9.31E-08
SLC25A53	solute carrier family 25 member 53	-0.982182164	9.62E-08
GPR27	G protein-coupled receptor 27	-0.658714735	9.70E-08
KLHL8	kelch like family member 8	0.693262936	9.94E-08
MYO5B	myosin VB	-1.318820332	1.11E-07
C3AR1	complement C3a receptor 1	4.680769513	1.12E-07
PWWP3A	PWWP domain containing 3A; DNA repair factor	-0.594888512	1.14E-07
FAM181B	family with sequence similarity 181 member B	-1.801888815	1.14E-07
LAMB2	laminin subunit beta 2	-0.687784428	1.14E-07
LAMA1	laminin subunit alpha 1	0.672966677	1.17E-07
ZNF528-AS1	ZNF528 antisense RNA 1	-1.350424253	1.20E-07
CLP1	cleavage factor polyribonucleotide kinase subunit 1	0.668852105	1.22E-07
ITPKB	inositol-trisphosphate 3-kinase B	-0.694038942	1.22E-07
PTPRN2	protein tyrosine phosphatase receptor type N2	-0.806458561	1.23E-07
ZNF471	zinc finger protein 471	-0.961455526	1.25E-07
NALF2	NALCN channel auxiliary factor 2	1.342511281	1.27E-07
ITPR3	inositol 1;4;5-trisphosphate receptor type 3	0.940793991	1.27E-07
CALHM6	calcium homeostasis modulator family member 6	5.897805532	1.29E-07
LRRC49	leucine rich repeat containing 49	-0.749300367	1.34E-07
H4C8	H4 clustered histone 8	1.497806265	1.36E-07

SVEP1	sushi; von Willebrand factor type A; EGF and pentraxin domain containing 1	-0.616483497	1.37E-07
CERS4	ceramide synthase 4	-0.65487769	1.46E-07
RRP15	ribosomal RNA processing 15 homolog	0.646011047	1.48E-07
BDNF-AS	BDNF antisense RNA	-1.285790556	1.56E-07
TBC1D12	TBC1 domain family member 12	-0.59221448	1.57E-07
LMBR1L	limb development membrane protein 1 like	-0.810215228	1.57E-07
ABCB11	ATP binding cassette subfamily B member 11	-1.152318365	1.61E-07
EVA1A	eva-1 homolog A; regulator of programmed cell death	0.607519636	1.62E-07
ATP8A1	ATPase phospholipid transporting 8A1	1.066621721	1.68E-07
APOBEC3F	apolipoprotein B mRNA editing enzyme catalytic subunit 3F	3.142175942	1.68E-07
IFT22	intraflagellar transport 22	-0.631705141	1.71E-07
SLC22A4	solute carrier family 22 member 4	1.570092347	1.73E-07
DLX6	distal-less homeobox 6	-1.369905962	1.75E-07
KRT80	keratin 80	-0.744776057	1.76E-07
RRAGD	Ras related GTP binding D	-0.73988449	1.76E-07
ODAD3	outer dynein arm docking complex subunit 3	-1.552918762	1.80E-07
CBX4	chromobox 4	-0.708676225	1.90E-07
SEPTIN1	septin 1	-1.776864019	1.91E-07
MB21D2	Mab-21 domain containing 2	0.963844613	1.96E-07
KIF6	kinesin family member 6	2.952269472	1.98E-07
C8orf34	chromosome 8 open reading frame 34	-1.504014282	2.00E-07
CLEC2B	C-type lectin domain family 2 member B	2.123292588	2.00E-07
C19orf38	chromosome 19 open reading frame 38	4.819028752	2.02E-07
SEZ6L	seizure related 6 homolog like	-1.60855663	2.03E-07
SELENOT	selenoprotein T	0.695094914	2.03E-07
KCNIP3	potassium voltage-gated channel interacting protein 3	-1.487911204	2.19E-07
COA6	cytochrome c oxidase assembly factor 6	0.651450548	2.21E-07
CENPP	centromere protein P	0.853009085	2.24E-07
CBX8	chromobox 8	-0.604821358	2.31E-07
DONSON	DNA replication fork stabilization factor DONSON	-0.602709916	2.33E-07
KREMEN2	kringle containing transmembrane protein 2	0.946163648	2.34E-07
LEMD1	LEM domain containing 1	-1.637581756	2.38E-07
SORCS2	sortilin related VPS10 domain containing receptor 2	-1.017753015	2.39E-07
CDC25C	cell division cycle 25C	-0.767409892	2.42E-07
ZNF703	zinc finger protein 703	-0.798346239	2.48E-07
EMID1	EMI domain containing 1	-0.737773422	2.49E-07

RCAN3	RCAN family member 3	-1.398833884	2.51E-07
PRPSAP1	phosphoribosyl pyrophosphate synthetase associated protein 1	-0.701100791	2.55E-07
SNAI2	snail family transcriptional repressor 2	-0.78592664	2.57E-07
SLC6A16	solute carrier family 6 member 16	-1.451284911	2.64E-07
TMEM108	transmembrane protein 108	-0.680325594	2.65E-07
LAMA5	laminin subunit alpha 5	-0.77787915	2.68E-07
HEBP2	heme binding protein 2	-0.727266671	2.73E-07
TOX3	TOX high mobility group box family member 3	-1.268335321	2.78E-07
PCDHGB7	protocadherin gamma subfamily B; 7	-0.793833682	2.80E-07
KLF4	KLF transcription factor 4	3.57053368	2.84E-07
TNS2-AS1	TNS2 antisense RNA 1	-1.831271065	2.95E-07
SFXN4	sideroflexin 4	0.62890832	2.95E-07
CBARP	CACN subunit beta associated regulatory protein	-0.708975037	2.96E-07
ALDH3B1	aldehyde dehydrogenase 3 family member B1	-0.750556678	2.97E-07
H3-7	H3,7 histone (putative)	1.230718957	3.02E-07
SERPINA1	serpin family A member 1	3.573384911	3.07E-07
CHSY3	chondroitin sulfate synthase 3	1.253930577	3.07E-07
EPHX1	epoxide hydrolase 1	-0.611230975	3.13E-07
TMEM134	transmembrane protein 134	-0.736439113	3.13E-07
CECR2	CECR2 histone acetyl-lysine reader	-0.77539768	3.17E-07
ZBTB8B	zinc finger and BTB domain containing 8B	-1.906097169	3.23E-07
FAXDC2	fatty acid hydroxylase domain containing 2	-0.991873977	3.29E-07
TCAF1P1	TRPM8 channel associated factor 1 pseudogene 1	-0.94287711	3.31E-07
MEFV	MEFV innate immunity regulator; pyrin	5.425950226	3.32E-07
SIDT1	SID1 transmembrane family member 1	2.762713499	3.35E-07
ACACB	acetyl-CoA carboxylase beta	-1.157445591	3.38E-07
PIK3IP1	phosphoinositide-3-kinase interacting protein 1	-0.772504398	3.38E-07
DOC2B	double C2 domain beta	3.42050517	3.44E-07
FAR2	fatty acyl-CoA reductase 2	1.554456312	3.57E-07
HYI	hydroxypyruvate isomerase (putative)	-0.888012913	3.62E-07
AZI2	5-azacytidine induced 2	0.643889361	3.64E-07
SYT14	synaptotagmin 14	-1.201764183	3.67E-07
SCEL	sciellin	-4.158839355	3.74E-07
RAB3D	RAB3D; member RAS oncogene family	-0.699211213	3.76E-07
COL8A2	collagen type VIII alpha 2 chain	-1.151082249	3.77E-07
SENP7	SUMO specific peptidase 7	-0.875996155	3.90E-07
LRR1	leucine rich repeat protein 1	0.687452473	3.90E-07

RBPMS2	RNA binding protein; mRNA processing factor 2	-0.786860316	3.95E-07
ALKBH8	alkB homolog 8; tRNA methyltransferase	0.603372282	3.97E-07
SHF	Src homology 2 domain containing F	-1.101259576	4.01E-07
EPHA5-AS1	EPHA5 antisense RNA 1	-5.058207971	4.03E-07
MAGI2	membrane associated guanylate kinase; WW and PDZ domain containing 2	-1.579567074	4.06E-07
CRYBG1	crystallin beta-gamma domain containing 1	1.439256378	4.09E-07
CR1L	complement C3b/C4b receptor 1 like	4.860260666	4.16E-07
SLC22A5	solute carrier family 22 member 5	0.604952397	4.18E-07
LRRC3	leucine rich repeat containing 3	1.264876553	4.22E-07
WAS	WASP actin nucleation promoting factor	-2.019661647	4.22E-07
DISP3	dispatched RND transporter family member 3	-1.888077703	4.25E-07
FAM83G	family with sequence similarity 83 member G	0.606793718	4.29E-07
MYO5A	myosin VA	-0.752573119	4.29E-07
RHBDF2	rhomboid 5 homolog 2	1.859371436	4.32E-07
FLYWCH2	FLYWCH family member 2	-0.621515639	4.42E-07
NTMT1	N-terminal Xaa-Pro-Lys N-methyltransferase 1	0.629103577	4.52E-07
TWIST2	twist family bHLH transcription factor 2	0.814886252	4.61E-07
CGN	cingulin	-0.966184354	4.67E-07
GUCY1A1	guanylate cyclase 1 soluble subunit alpha 1	0.854392552	4.78E-07
CLVS2	clavesin 2	-3.290248886	4.82E-07
CENPE	centromere protein E	-0.6178734	4.90E-07
SPATA20	spermatogenesis associated 20	-0.735674819	4.97E-07
TGM4	transglutaminase 4	2.655765788	5.07E-07
CTSS	cathepsin S	6.110530406	5.09E-07
COL17A1	collagen type XVII alpha 1 chain	1.377064241	5.09E-07
CCDC191	coiled-coil domain containing 191	-0.841192174	5.14E-07
TACC2	transforming acidic coiled-coil containing protein 2	-0.767138749	5.18E-07
PCDHGA9	protocadherin gamma subfamily A; 9	-0.876903946	5.20E-07
CACNA1H	calcium voltage-gated channel subunit alpha1 H	-0.68575023	5.44E-07
GLI2	GLI family zinc finger 2	-0.73383055	5.45E-07
YBX2	Y-box binding protein 2	-1.175989164	5.50E-07
FGD3	FYVE; RhoGEF and PH domain containing 3	-1.917381448	5.53E-07
PRSS56	serine protease 56	2.973586165	5.54E-07
AFAP1L2	actin filament associated protein 1 like 2	-2.336082363	5.54E-07
ZMAT1	zinc finger matrin-type 1	-1.421126633	5.55E-07
TDO2	tryptophan 2;3-dioxygenase	1.568603264	5.85E-07

ADM2	adrenomedullin 2	2.059557054	6.19E-07
INSM1	INSM transcriptional repressor 1	-0.759286327	6.23E-07
EIF3FP3	eukaryotic translation initiation factor 3 subunit F pseudogene 3	-0.629002833	6.28E-07
COL15A1	collagen type XV alpha 1 chain	3.83849478	6.34E-07
RRAS	RAS related	0.730300591	6.35E-07
LINC00472	long intergenic non-protein coding RNA 472	-0.742284294	6.41E-07
H2BC11	H2B clustered histone 11	1.810037976	6.42E-07
BTG1	BTG anti-proliferation factor 1	0.939251384	6.46E-07
GPRASP2	G protein-coupled receptor associated sorting protein 2	-0.755525572	6.51E-07
STC1	stanniocalcin 1	-0.913646935	6.77E-07
ENPP2	ectonucleotide pyrophosphatase/phosphodiesterase 2	-1.332716285	6.83E-07
SOX7	SRY-box transcription factor 7	0.988554929	6.92E-07
UCN2	urocortin 2	2.757543291	6.93E-07
ADCY8	adenylate cyclase 8	-0.770478703	6.97E-07
MEIS3	Meis homeobox 3	-0.71045422	7.10E-07
FBXL16	F-box and leucine rich repeat protein 16	-0.608424865	7.22E-07
PLA2G4C	phospholipase A2 group IVC	4.197558754	7.26E-07
LRP1B	LDL receptor related protein 1B	-0.985919788	7.41E-07
STAC	SH3 and cysteine rich domain	-0.612081627	7.46E-07
MT1H	metallothionein 1H	6.177260927	7.59E-07
SALL3	spalt like transcription factor 3	-0.860062358	7.68E-07
DHRS7	dehydrogenase/reductase 7	-0.724045722	7.91E-07
STRA6	signaling receptor and transporter of retinol STRA6	1.1494405	7.99E-07
NDUFA6-DT	NDUFA6 divergent transcript	-1.140758478	8.23E-07
HIVEP2	HIVEP zinc finger 2	0.624314475	8.37E-07
SH3BP5-AS	SH3BP5 antisense RNA 1	-1.003412311	8.37E-07
IGDCC3	immunoglobulin superfamily DCC subclass member 3	-0.676416067	8.46E-07
TBX1	T-box transcription factor 1	2.113999385	8.49E-07
EXD3	exonuclease 3'-5' domain containing 3	-0.833447678	8.52E-07
FRZB	frizzled related protein	-2.395491206	8.67E-07
LRRN2	leucine rich repeat neuronal 2	-1.371439209	8.71E-07
RGS5	regulator of G protein signaling 5	-1.484201013	8.75E-07
ANKRD63	ankyrin repeat domain 63	-1.294187247	8.85E-07
PDCD2L	programmed cell death 2 like	0.677168908	8.97E-07
IL18R1	interleukin 18 receptor 1	4.643833216	9.03E-07
CCDC13	coiled-coil domain containing 13	-1.470368778	9.22E-07
EHF	ETS homologous factor	3.431097544	9.55E-07
SLC37A1	solute carrier family 37 member 1	0.999115438	9.60E-07
HPD	4-hydroxyphenylpyruvate dioxygenase	-1.341690427	9.69E-07

KIF5A	kinesin family member 5A	-0.762517482	9.85E-07
DGKA	diacylglycerol kinase alpha	-0.703701171	1.03E-06
ZDHHC2	zinc finger DHHC-type palmitoyltransferase 2	-0.849096578	1.03E-06
SNX30	sorting nexin family member 30	-0.732990418	1.06E-06
ROS1	ROS proto-oncogene 1; receptor tyrosine kinase	5.260761617	1.07E-06
RIMS3	regulating synaptic membrane exocytosis 3	-1.191963824	1.08E-06
GET1-SH3B	GET1-SH3BGR readthrough	-3.458748009	1.09E-06
CAVIN2	caveolae associated protein 2	1.694751431	1.10E-06
PCSK4	proprotein convertase subtilisin/kexin type 4	-1.158412844	1.10E-06
ZBED5-AS1	ZBED5 antisense RNA 1	-1.314854096	1.14E-06
CTRL	chymotrypsin like	1.444230617	1.14E-06
MTFR2	mitochondrial fission regulator 2	0.869007102	1.15E-06
ACSS1	acyl-CoA synthetase short chain family member 1	1.158571294	1.16E-06
PPFIA2	PTPRF interacting protein alpha 2	-0.762220814	1.17E-06
MSH5	mutS homolog 5	-0.830712779	1.20E-06
MPND	MPN domain containing	-0.695034906	1.25E-06
RPL17-C18o	RPL17-C18orf32 readthrough	-0.731798449	1.26E-06
DENND6B	DENN domain containing 6B	-0.657593114	1.29E-06
APCDD1L-D	APCDD1L divergent transcript	2.233450697	1.29E-06
RNF141	ring finger protein 141	-0.614140173	1.31E-06
DOK3	docking protein 3	1.145428242	1.32E-06
NEO1	neogenin 1	-0.708604691	1.33E-06
CCDC28B	coiled-coil domain containing 28B	-0.814931211	1.33E-06
TLCD1	TLC domain containing 1	0.615849531	1.36E-06
SIAH2	siah E3 ubiquitin protein ligase 2	0.740480404	1.45E-06
USP28	ubiquitin specific peptidase 28	0.7140842	1.48E-06
SLC29A3	solute carrier family 29 member 3	1.26936543	1.51E-06
GOSR1	golgi SNAP receptor complex member 1	0.609005005	1.51E-06
SLC6A15	solute carrier family 6 member 15	1.424443604	1.51E-06
IL1RN	interleukin 1 receptor antagonist	6.994158409	1.54E-06
INSYN1	inhibitory synaptic factor 1	-2.410182941	1.58E-06
ATCAY	ATCAY kinesin light chain interacting caytaxin	-1.498960681	1.61E-06
MIA2	MIA SH3 domain ER export factor 2	0.746766458	1.62E-06
ADAMTS14	ADAM metallopeptidase with thrombospondin type 1 motif 14	0.941735713	1.63E-06
RGPD5	RANBP2 like and GRIP domain containing 5	-0.707020513	1.70E-06
SVIP	small VCP interacting protein	-0.661973218	1.71E-06
ISM2	isthmin 2	1.677924263	1.72E-06

REXO5	RNA exonuclease 5	-1.040072492	1.74E-06
SERINC2	serine incorporator 2	-1.32321125	1.76E-06
SH2B2	SH2B adaptor protein 2	0.656751018	1.78E-06
PNMA8B	PNMA family member 8B	-1.218862723	1.80E-06
AKAP5	A-kinase anchoring protein 5	-0.904684035	1.81E-06
GPX3	glutathione peroxidase 3	-0.941826262	1.82E-06
CPZ	carboxypeptidase Z	-0.87064843	1.85E-06
HKDC1	hexokinase domain containing 1	-0.663077564	1.85E-06
TNFRSF4	TNF receptor superfamily member 4	5.720750757	1.87E-06
ADCY6	adenylate cyclase 6	-0.609749518	1.88E-06
IL4R	interleukin 4 receptor	0.676640884	1.88E-06
RNF150	ring finger protein 150	-1.072410115	1.90E-06
RDH14	retinol dehydrogenase 14	0.631002591	1.91E-06
TAF5	TATA-box binding protein associated factor 5	0.62680961	1.93E-06
PALM3	paralemmin 3	-1.388205943	1.97E-06
ARHGEF10	Rho guanine nucleotide exchange factor 10	-0.84233964	1.99E-06
C16orf74	chromosome 16 open reading frame 74	-1.78405967	2.01E-06
TMEM107	transmembrane protein 107	-0.881897521	2.03E-06
HCK	HCK proto-oncogene; Src family tyrosine kinase	6.046559838	2.09E-06
KBTBD7	kelch repeat and BTB domain containing 7	-0.808030106	2.14E-06
BSG-AS1	BSG antisense RNA 1	-1.209855419	2.16E-06
EPB41L4A	erythrocyte membrane protein band 4,1 like 4A	-1.681520994	2.18E-06
ADCY2	adenylate cyclase 2	-1.034633252	2.24E-06
FOXP1	forkhead box P1	1.025158598	2.25E-06
OSER1-DT	OSER1 divergent transcript	-1.007166246	2.26E-06
EFHC1	EF-hand domain containing 1	-0.680938376	2.28E-06
DIRAS1	DIRAS family GTPase 1	-0.72693627	2.30E-06
CTNND1	catenin delta 1	-0.648655651	2.31E-06
C3orf18	chromosome 3 open reading frame 18	-1.249466651	2.32E-06
NAIP	NLR family apoptosis inhibitory protein	-0.841029124	2.32E-06
LRRC23	leucine rich repeat containing 23	-0.858569228	2.39E-06
CCNJL	cyclin J like	-0.644965715	2.40E-06
CDKN2B	cyclin dependent kinase inhibitor 2B	0.880796459	2.45E-06
BDKRB2	bradykinin receptor B2	4.827309957	2.46E-06
AP1G2-AS1	AP1G2 antisense RNA 1	1.070203872	2.47E-06
FAM43A	family with sequence similarity 43 member A	0.830465763	2.47E-06
CTSO	cathepsin O	0.745332149	2.49E-06
ARSL	arylsulfatase L	-0.933829455	2.50E-06

LINC00205	long intergenic non-protein coding RNA 205	-0.832551244	2.51E-06
FAM228B	family with sequence similarity 228 member B	-1.313641762	2.52E-06
PCDHB16	protocadherin beta 16	-1.108726973	2.54E-06
CRYM	crystallin mu	-1.763907316	2.60E-06
NTSR2	neurotensin receptor 2	2.15306385	2.61E-06
TENM2	teneurin transmembrane protein 2	-0.844772469	2.62E-06
PIK3R1	phosphoinositide-3-kinase regulatory subunit 1	0.604315709	2.63E-06
ANO8	anoctamin 8	-0.619528762	2.69E-06
CDKN2A	cyclin dependent kinase inhibitor 2A	0.721119269	2.69E-06
PRR16	proline rich 16	-0.903181794	2.71E-06
LRRC15	leucine rich repeat containing 15	-0.89383193	2.82E-06
SLC25A35	solute carrier family 25 member 35	-1.530448543	2.84E-06
TMEM117	transmembrane protein 117	-0.789182802	2.89E-06
SYT1	synaptotagmin 1	-0.606411065	2.94E-06
RASL11B	RAS like family 11 member B	1.210542075	2.95E-06
HLA-DRB1	major histocompatibility complex; class II; DR beta 1	0.646042844	3.00E-06
PRSS35	serine protease 35	-1.546649402	3.04E-06
A1BG	alpha-1-B glycoprotein	-1.515088871	3.15E-06
LAYN	layilin	-0.616513958	3.19E-06
CD101	CD101 molecule	-1.248730861	3.20E-06
PWWP2B	PWWP domain containing 2B	0.605361369	3.23E-06
TERT	telomerase reverse transcriptase	0.973230751	3.26E-06
ANGPT1	angiopoietin 1	1.541537241	3.27E-06
SCML1	Scm polycomb group protein like 1	0.63626538	3.31E-06
GAD2	glutamate decarboxylase 2	-1.546844333	3.40E-06
MR1	major histocompatibility complex; class I-related	1.5113462	3.43E-06
PAK1	p21 (RAC1) activated kinase 1	-0.709481751	3.44E-06
PPIP5K1	diphosphoinositol pentakisphosphate kinase 1	-0.735365584	3.50E-06
LINC00632	long intergenic non-protein coding RNA 632	-1.223456331	3.51E-06
SERTAD4	SERTA domain containing 4	-1.897481877	3.62E-06
HLA-DRA	major histocompatibility complex; class II; DR alpha	0.634184877	3.72E-06
LRRC9	leucine rich repeat containing 9	-2.412729189	3.72E-06
ZNF528	zinc finger protein 528	-0.916516013	3.79E-06
CIITA	class II major histocompatibility complex transactivator	0.610482392	3.82E-06
CNTROB	centrobin; centriole duplication and spindle assembly protein	-0.685383981	3.82E-06
EMSY-DT	EMSY divergent transcript	-1.484240491	3.83E-06

RMI2	RecQ mediated genome instability 2	0.626881144	3.98E-06
ELF4	E74 like ETS transcription factor 4	0.63605426	4.00E-06
IL12RB1	interleukin 12 receptor subunit beta 1	4.769667483	4.04E-06
CACNA1C	calcium voltage-gated channel subunit alpha1 C	-1.7011856	4.05E-06
DDIT3	DNA damage inducible transcript 3	1.014283264	4.07E-06
TMEM121B	transmembrane protein 121B	-1.094458183	4.20E-06
PCDHGA10	protocadherin gamma subfamily A; 10	-0.865260686	4.21E-06
MIF4GD	MIF4G domain containing	-0.762747399	4.23E-06
EFHD1	EF-hand domain family member D1	-1.042398248	4.23E-06
ERCC6L2	ERCC excision repair 6 like 2	0.725846105	4.24E-06
GAREM1	GRB2 associated regulator of MAPK1 subtype 1	0.708632546	4.25E-06
HSPA12A	heat shock protein family A (Hsp70) member 12A	-0.90475717	4.38E-06
SEMA3A	semaphorin 3A	-0.6359656	4.41E-06
ZDHHC14	zinc finger DHHC-type palmitoyltransferase 14	0.621835462	4.47E-06
ZNF845	zinc finger protein 845	0.606717665	4.50E-06
ABCA5	ATP binding cassette subfamily A member 5	-1.327243179	4.72E-06
CACNA1S	calcium voltage-gated channel subunit alpha1 S	6.132683438	4.74E-06
CXCL2	C-X-C motif chemokine ligand 2	6.770567759	4.78E-06
FILIP1L	filamin A interacting protein 1 like	1.03689409	4.82E-06
C1orf54	chromosome 1 open reading frame 54	-1.426927748	4.84E-06
NAT16	N-acetyltransferase 16 (putative)	-3.116959985	4.84E-06
C2orf27A	chromosome 2 open reading frame 27A	1.118026206	4.87E-06
P4HA2	prolyl 4-hydroxylase subunit alpha 2	0.604166756	4.96E-06
SSC5D	scavenger receptor cysteine rich family member with 5 domains	-0.843591585	5.00E-06
LINC01918	long intergenic non-protein coding RNA 1918	2.295395988	5.01E-06
POU2F3	POU class 2 homeobox 3	5.991990646	5.07E-06
EFS	embryonal Fyn-associated substrate	-1.12744049	5.11E-06
TMEM154	transmembrane protein 154	2.459078686	5.11E-06
TMCC2	transmembrane and coiled-coil domain family 2	-0.72623748	5.12E-06
PRRT2	proline rich transmembrane protein 2	-0.737529011	5.23E-06
GAD1	glutamate decarboxylase 1	-0.893183545	5.31E-06
ITPR1	inositol 1;4;5-trisphosphate receptor type 1	0.595430126	5.35E-06
H2BC6	H2B clustered histone 6	1.957949869	5.41E-06
EEF1A2	eukaryotic translation elongation factor 1 alpha 2	-0.967647961	5.52E-06
NTN3	netrin 3	-1.429868324	5.59E-06

EMILIN3	elastin microfibril interfacer 3	0.890918638	5.61E-06
VEPH1	ventricular zone expressed PH domain containing 1	-3.357790292	5.62E-06
SLC19A3	solute carrier family 19 member 3	1.084665094	5.66E-06
ISCA1	iron-sulfur cluster assembly 1	0.624897038	5.69E-06
SERPINB12	serpin family B member 12	-4.707969257	5.74E-06
PHEX	phosphate regulating endopeptidase X-linked	-1.826639188	5.95E-06
MSH5-SAPC	MSH5-SAPCD1 readthrough (NMD candidate)	-0.793448772	5.99E-06
ARC	activity regulated cytoskeleton associated protein	-0.693873967	6.01E-06
CCDC74A	coiled-coil domain containing 74A	-0.782579967	6.01E-06
PACRG	parkin coregulated	-1.645348911	6.07E-06
TAGLN3	transgelin 3	-0.669009418	6.09E-06
MLF1	myeloid leukemia factor 1	-0.829177343	6.26E-06
TFCP2L1	transcription factor CP2 like 1	-1.42946194	6.36E-06
NPS	neuropeptide S	1.641365666	6.37E-06
PDGFA	platelet derived growth factor subunit A	-1.404388083	6.44E-06
BTC	betacellulin	4.120591825	6.81E-06
PSMG3-AS1	PSMG3 antisense RNA 1 (head to head)	-0.808835044	6.83E-06
LINC01551	long intergenic non-protein coding RNA 1551	-0.595903306	7.13E-06
FBXO39	F-box protein 39	5.903322051	7.19E-06
DPF3	double PHD fingers 3	-0.61857896	7.30E-06
TEC	tec protein tyrosine kinase	1.541081591	7.33E-06
RHBDF1	rhomboid 5 homolog 1	-0.742375719	7.36E-06
NMNAT1	nicotinamide nucleotide adenylyltransferase 1	-0.680045843	7.39E-06
ASB2	ankyrin repeat and SOCS box containing 2	4.182945697	7.39E-06
CFAP90	cilia and flagella associated protein 90	-1.133043918	7.41E-06
DHFR2	dihydrofolate reductase 2	-0.879057709	7.59E-06
PCDHA10	protocadherin alpha 10	-1.791297615	7.73E-06
SLC2A5	solute carrier family 2 member 5	3.573106337	7.84E-06
PDLIM3	PDZ and LIM domain 3	-1.512148262	7.86E-06
H2BC18	H2B clustered histone 18	1.643531469	8.06E-06
FHIP1A	FHF complex subunit HOOK interacting protein 1A	0.962646583	8.20E-06
ELOVL7	ELOVL fatty acid elongase 7	3.113686269	8.45E-06
VAMP5	vesicle associated membrane protein 5	0.652607658	8.55E-06
SPATA18	spermatogenesis associated 18	-0.758402514	8.64E-06
C2orf76	chromosome 2 open reading frame 76	-0.856687948	8.69E-06
PLPPR5	phospholipid phosphatase related 5	-0.880901755	8.84E-06
TFB2M	transcription factor B2; mitochondrial	0.591540693	8.87E-06

ST18	ST18 C2H2C-type zinc finger transcription factor	-1.434056592	8.89E-06
PDZD2	PDZ domain containing 2	3.235988185	9.01E-06
GPR141	G protein-coupled receptor 141	3.486900826	9.36E-06
INKA1	inka box actin regulator 1	1.69533355	9.54E-06
EIF3J-DT	EIF3J divergent transcript	-0.805611853	9.55E-06
CXCL1	C-X-C motif chemokine ligand 1	4.951168399	9.55E-06
RTL10	retrotransposon Gag like 10	-0.650893422	9.79E-06
IQCK	IQ motif containing K	-0.610682081	9.85E-06
KCNIP1	potassium voltage-gated channel interacting protein 1	-0.791312037	1.00E-05
AUNIP	aurora kinase A and ninein interacting protein	0.670984676	1.01E-05
DNMT3B	DNA methyltransferase 3 beta	-1.005960212	1.03E-05
LINC01159	long intergenic non-protein coding RNA 1159	-0.98715391	1.04E-05
DCLRE1C	DNA cross-link repair 1C	0.766848323	1.04E-05
LOC730338	uncharacterized LOC730338	3.638545137	1.07E-05
PCDH12	protocadherin 12	6.245088515	1.09E-05
CFAP43	cilia and flagella associated protein 43	-2.042564859	1.10E-05
DSG2	desmoglein 2	-0.593180134	1.10E-05
SLC4A8	solute carrier family 4 member 8	-0.783505637	1.11E-05
FIBCD1	fibrinogen C domain containing 1	-1.099242621	1.12E-05
APOE	apolipoprotein E	-0.82425003	1.12E-05
FAHD2B	fumarylacetoacetate hydrolase domain containing 2B	-0.970799626	1.14E-05
NPIPA7	nuclear pore complex interacting protein family member A7	0.69356663	1.14E-05
IQCG	IQ motif containing G	-0.632573111	1.14E-05
CXCR4	C-X-C motif chemokine receptor 4	0.641538529	1.17E-05
UQCC4	ubiquinol-cytochrome c reductase complex assembly factor 4	0.769684486	1.18E-05
KCNQ4	potassium voltage-gated channel subfamily Q member 4	0.848580167	1.19E-05
SH3BGRL2	SH3 domain binding glutamate rich protein like 2	0.6427387	1.19E-05
DGKE	diacylglycerol kinase epsilon	1.093582449	1.23E-05
AMBRA1	autophagy and beclin 1 regulator 1	0.71325988	1.23E-05
TMEM38B	transmembrane protein 38B	0.786663048	1.26E-05
CTNS	cystinosin; lysosomal cystine transporter	1.041301466	1.28E-05
PPP3CC	protein phosphatase 3 catalytic subunit gamma	0.724091873	1.30E-05
SDK2	sidekick cell adhesion molecule 2	-0.905153378	1.33E-05
KIN	Kin17 DNA and RNA binding protein	0.69606133	1.35E-05
DNAI3	dynein axonemal intermediate chain 3	-1.024852513	1.37E-05
LRRC51	leucine rich repeat containing 51	-1.094465872	1.39E-05

C5AR1	complement C5a receptor 1	1.535980191	1.39E-05
CHAC1	ChaC glutathione specific gamma-glutamylcyclotransferase 1	1.370443908	1.40E-05
CUBN	cubilin	-1.622902289	1.41E-05
C11orf96	chromosome 11 open reading frame 96	0.686074144	1.44E-05
RALGPS2	Ral GEF with PH domain and SH3 binding motif 2	-0.8766139	1.45E-05
CPEB3	cytoplasmic polyadenylation element binding protein 3	1.72073235	1.47E-05
CEND1	cell cycle exit and neuronal differentiation 1	-0.636474812	1.47E-05
RUFY4	RUN and FYVE domain containing 4	5.189327484	1.49E-05
MINDY1	MINDY lysine 48 deubiquitinase 1	-1.434200748	1.52E-05
MALAT1	metastasis associated lung adenocarcinoma transcript 1	-0.656843769	1.55E-05
AR	androgen receptor	-0.998767899	1.57E-05
PCDHA13	protocadherin alpha 13	-5.587629221	1.61E-05
PPM1K-DT	PPM1K divergent transcript	4.579153744	1.63E-05
JHY	junctional cadherin complex regulator	-0.954805573	1.65E-05
EGLN3	egl-9 family hypoxia inducible factor 3	-0.990492315	1.66E-05
CEP170B	centrosomal protein 170B	-0.637952859	1.69E-05
ALG5	ALG5 dolichyl-phosphate beta-glucosyltransferase	0.82405297	1.71E-05
CENPF	centromere protein F	-0.653623771	1.72E-05
SHC4	SHC adaptor protein 4	-1.318012084	1.73E-05
PARD6B	par-6 family cell polarity regulator beta	0.927325211	1.75E-05
LCIIAR	lung cancer immune cell infiltration associated lncRNA	1.288025884	1.75E-05
CSRNP3	cysteine and serine rich nuclear protein 3	-1.483728188	1.76E-05
ID2	inhibitor of DNA binding 2	-0.625896344	1.81E-05
ARL15	ADP ribosylation factor like GTPase 15	-0.836961081	1.83E-05
ASAP2	ArfGAP with SH3 domain; ankyrin repeat and PH domain 2	-0.590238991	1.85E-05
TESK1	testis associated actin remodelling kinase 1	-0.639813707	1.85E-05
TOP6BL	TOP6B like initiator of meiotic double strand breaks	-0.820221256	1.86E-05
XKRX	XK related X-linked	-1.337833826	1.87E-05
SLC6A6	solute carrier family 6 member 6	1.322994666	1.88E-05
CAMKK1	calcium/calmodulin dependent protein kinase kinase 1	-0.660314869	1.90E-05
ZNF33B	zinc finger protein 33B	-0.613503719	1.91E-05
RPS10-NUD	RPS10-NUDT3 readthrough	2.047277578	1.92E-05
IGF2	insulin like growth factor 2	2.511708889	1.93E-05
CA9	carbonic anhydrase 9	1.986522114	1.94E-05

CCND2-AS1	CCND2 antisense RNA 1	-1.827290762	1.95E-05
ADAMTS13	ADAM metallopeptidase with thrombospondin type 1 motif 13	-1.414632584	1.98E-05
ACAP3	ArfGAP with coiled-coil; ankyrin repeat and PH domains 3	-0.65199115	2.01E-05
FAM110A	family with sequence similarity 110 member A	-0.911890188	2.03E-05
DNAJC27-A	DNAJC27 antisense RNA 1	-3.571182934	2.08E-05
STC2	stanniocalcin 2	0.788527478	2.08E-05
PDCD1LG2	programmed cell death 1 ligand 2	1.713150987	2.09E-05
SLC7A2	solute carrier family 7 member 2	0.71265596	2.09E-05
BRSK1	BR serine/threonine kinase 1	-0.742016641	2.10E-05
ESAM	endothelial cell adhesion molecule	-1.662469461	2.10E-05
MACC1	MET transcriptional regulator MACC1	5.132099131	2.18E-05
ACVR2A	activin A receptor type 2A	0.688286749	2.21E-05
DGKG	diacylglycerol kinase gamma	2.850654566	2.21E-05
INTS4P2	integrator complex subunit 4 pseudogene 2	-1.827150211	2.22E-05
NEGR1	neuronal growth regulator 1	-0.800517979	2.22E-05
MAPT	microtubule associated protein tau	-1.413351605	2.24E-05
CHAC2	ChaC glutathione specific gamma-glutamylcyclotransferase 2	0.696706131	2.28E-05
ELOVL2	ELOVL fatty acid elongase 2	-0.734598652	2.35E-05
LOC1164352	uncharacterized LOC116435278	-2.463845172	2.35E-05
MSANTD2-A	MSANTD2 antisense RNA 1	-1.451041736	2.36E-05
CEP131	centrosomal protein 131	-0.600057929	2.39E-05
RND1	Rho family GTPase 1	1.336976998	2.43E-05
FCGRT	Fc gamma receptor and transporter	-0.598555189	2.43E-05
ATP2B2	ATPase plasma membrane Ca2+ transporting 2	-0.813699314	2.44E-05
PRLR	prolactin receptor	3.673124533	2.44E-05
EPHX2	epoxide hydrolase 2	-1.244867143	2.51E-05
RGS11	regulator of G protein signaling 11	-1.453042244	2.53E-05
BCL2A1	BCL2 related protein A1	5.827272113	2.53E-05
SEC11C	SEC11 homolog C; signal peptidase complex subunit	0.642076653	2.54E-05
RGCC	regulator of cell cycle	-1.666763224	2.60E-05
TTC9	tetratricopeptide repeat domain 9	-0.826078273	2.63E-05
DDN-AS1	DDN and PRKAG1 antisense RNA 1	1.068031437	2.77E-05
NPL	N-acetylneuraminate pyruvate lyase	1.44401113	2.82E-05
ACSF2	acyl-CoA synthetase family member 2	-1.027461252	2.86E-05
CDC42EP2	CDC42 effector protein 2	1.041190609	2.87E-05
FRMD4B	FERM domain containing 4B	0.783148986	2.87E-05
PAQR5	progestin and adipoQ receptor family member 5	2.053117574	2.91E-05

MRPS18C	mitochondrial ribosomal protein S18C	0.678350985	2.95E-05
ROM1	retinal outer segment membrane protein 1	-0.716023846	2.96E-05
GAS2L3	growth arrest specific 2 like 3	-0.607925565	2.96E-05
NFKBIE	NFKB inhibitor epsilon	1.024134478	3.00E-05
SEPT5-GP1	SEPT5-GP1BB readthrough	-1.719504724	3.04E-05
SLC28A3	solute carrier family 28 member 3	4.304010706	3.07E-05
LSM6	LSM6 homolog; U6 small nuclear RNA and mRNA degradation associated	0.614644198	3.09E-05
LACC1	laccase domain containing 1	1.90653388	3.11E-05
ESRG	embryonic stem cell related	-1.905982571	3.12E-05
KIFC2	kinesin family member C2	-0.809175585	3.19E-05
SGSM1	small G protein signaling modulator 1	-3.4229123	3.25E-05
ZNF211	zinc finger protein 211	-0.646771396	3.26E-05
GIHCG	GIHCG inhibitor of miR-200b/200a/429 expression	-0.894925066	3.26E-05
DIAPH2	diaphanous related formin 2	0.620384465	3.27E-05
GRID2	glutamate ionotropic receptor delta type subunit 2	-3.495377905	3.30E-05
SAMD14	sterile alpha motif domain containing 14	-1.8509868	3.32E-05
DTX2	deltex E3 ubiquitin ligase 2	0.848546282	3.33E-05
CDC14B	cell division cycle 14B	-0.851184136	3.34E-05
FIGN	fidgetin; microtubule severing factor	-0.658814286	3.38E-05
ZNF593	zinc finger protein 593	0.593590709	3.41E-05
IRAG1	inositol 1;4;5-triphosphate receptor associated 1	-2.404925212	3.41E-05
CCHCR1	coiled-coil alpha-helical rod protein 1	-0.604269792	3.42E-05
SYNGR3	synaptogyrin 3	0.940982273	3.47E-05
ST8SIA5	ST8 alpha-N-acetyl-neuraminide alpha-2;8-sialyltransferase 5	-1.385271436	3.47E-05
LPL	lipoprotein lipase	0.605753136	3.53E-05
SHLD2P1	shieldin complex subunit 2 pseudogene 1	1.482542953	3.55E-05
ZNF350	zinc finger protein 350	0.845972134	3.56E-05
SLCO1C1	solute carrier organic anion transporter family member 1C1	-3.110741179	3.66E-05
PHC3	polyhomeotic homolog 3	0.628738031	3.77E-05
RSPH4A	radial spoke head component 4A	-0.870070133	3.86E-05
CELF3	CUGBP Elav-like family member 3	-1.427076904	3.92E-05
TSPY26P	testis specific protein Y-linked 26; pseudogene	-0.713840614	3.97E-05
OVOS2	alpha-2-macroglobulin like 1 pseudogene	3.863859942	3.97E-05
ZNF749	zinc finger protein 749	-0.703084712	4.06E-05
E2F8	E2F transcription factor 8	-0.764424301	4.09E-05
FBXL15	F-box and leucine rich repeat protein 15	0.614004012	4.10E-05

CREB3L4	cAMP responsive element binding protein 3 like 4	-0.902315847	4.16E-05
CSF3	colony stimulating factor 3	6.747374643	4.32E-05
TRAM2-AS1	TRAM2 antisense RNA 1 (head to head)	-0.707411187	4.34E-05
FAM222A-A:	FAM222A antisense RNA 1	2.226148511	4.41E-05
PRRT3	proline rich transmembrane protein 3	-0.708875475	4.42E-05
PDE8B	phosphodiesterase 8B	-0.984374899	4.50E-05
WNT3	Wnt family member 3	-0.714429058	4.53E-05
PAX8	paired box 8	1.932213064	4.56E-05
MSRB3	methionine sulfoxide reductase B3	-0.904902262	4.61E-05
DLGAP2	DLG associated protein 2	-3.440267249	4.66E-05
CLEC18C	C-type lectin domain family 18 member C	-2.014283347	4.66E-05
VAT1L	vesicle amine transport 1 like	-2.631142503	4.68E-05
PEX11G	peroxisomal biogenesis factor 11 gamma	-1.175206815	5.00E-05
ELAVL2	ELAV like RNA binding protein 2	-1.123899654	5.06E-05
ADSS1	adenylosuccinate synthase 1	-1.658008772	5.09E-05
CCDC121	coiled-coil domain containing 121	-0.938740423	5.14E-05
IRS1	insulin receptor substrate 1	-0.675383246	5.14E-05
SAMD15	sterile alpha motif domain containing 15	-0.705811128	5.16E-05
FAM200C	family with sequence similarity 200 member C	-0.668922273	5.34E-05
UXT-AS1	UXT antisense RNA 1	-2.47958542	5.34E-05
HSPA2	heat shock protein family A (Hsp70) member 2	-0.790523641	5.41E-05
H2BC4	H2B clustered histone 4	1.478882777	5.43E-05
TESC	tescalcin	-1.146155731	5.44E-05
RNF112	ring finger protein 112	-1.31442663	5.45E-05
TIMP4	TIMP metallopeptidase inhibitor 4	-1.628593456	5.46E-05
LACTB2	lactamase beta 2	0.868391964	5.48E-05
VAV3	vav guanine nucleotide exchange factor 3	-1.574390341	5.48E-05
ZFHX4-AS1	ZFHX4 antisense RNA 1	-0.746913166	5.52E-05
CRYL1	crystallin lambda 1	-0.818967458	5.53E-05
DRAIC	downregulated RNA in cancer; inhibitor of cell invasion and migration	-2.394509102	5.66E-05
DPY19L2	dpy-19 like 2	-0.8169149	5.69E-05
WDR31	WD repeat domain 31	-0.983195384	5.74E-05
CFH	complement factor H	3.145461043	5.85E-05
PCAT6	prostate cancer associated transcript 6	-1.584874981	5.87E-05
SLC25A42	solute carrier family 25 member 42	-0.757579846	5.89E-05
TMEM44	transmembrane protein 44	-0.859977737	5.90E-05
IL17RE	interleukin 17 receptor E	1.876255361	5.95E-05
POLR1H	RNA polymerase I subunit H	0.687826037	5.99E-05

LRRC37A2	leucine rich repeat containing 37 member A2	1.03661841	6.06E-05
SSTR3	somatostatin receptor 3	-1.682785199	6.06E-05
KRT18	keratin 18	-0.986267802	6.08E-05
TMEM169	transmembrane protein 169	-0.685906956	6.11E-05
PARD3B	par-3 family cell polarity regulator beta	-0.621464549	6.13E-05
SRPRB	SRP receptor subunit beta	0.66095974	6.13E-05
PLA2G6	phospholipase A2 group VI	-0.853443939	6.17E-05
ZNF829	zinc finger protein 829	0.61585799	6.30E-05
NECTIN3	nectin cell adhesion molecule 3	-0.613786518	6.34E-05
GLI4	GLI family zinc finger 4	-0.623972821	6.39E-05
GRAMD1C	GRAM domain containing 1C	-1.060729846	6.41E-05
ERV3-1	endogenous retrovirus group 3 member 1; envelope	-0.680399237	6.44E-05
UBE3D	ubiquitin protein ligase E3D	0.800436746	6.45E-05
MAL2	mal; T cell differentiation protein 2	-1.56722469	6.55E-05
TNFAIP8L1	TNF alpha induced protein 8 like 1	-0.632945294	6.57E-05
KIAA0040	KIAA0040	5.049511282	6.60E-05
C7orf57	chromosome 7 open reading frame 57	-1.880202941	6.64E-05
PSMG4	proteasome assembly chaperone 4	0.592208918	6.65E-05
VIM-AS1	VIM antisense RNA 1	-0.948158435	6.65E-05
NOMO3	NODAL modulator 3	-0.762406525	6.66E-05
SYNGR1	synaptogyrin 1	-0.73927791	6.78E-05
SPEN-AS1	SPEN antisense RNA 1	-1.004540309	6.80E-05
C3orf52	chromosome 3 open reading frame 52	1.026332014	6.90E-05
CD302	CD302 molecule	-0.786323993	6.93E-05
FSCN2	fascin actin-bundling protein 2; retinal	-3.20340972	6.94E-05
SIX4	SIX homeobox 4	0.701911025	6.99E-05
PURG	purine rich element binding protein G	-1.22868396	7.02E-05
PCOLCE	procollagen C-endopeptidase enhancer	-0.897574245	7.05E-05
CD55	CD55 molecule (Cromer blood group)	0.841052336	7.27E-05
ADD2	adducin 2	-0.665864384	7.28E-05
KCNJ2-AS1	KCNJ2 antisense RNA 1	-1.566421366	7.30E-05
DSC2	desmocollin 2	-1.935851225	7.54E-05
HVCN1	hydrogen voltage gated channel 1	3.209628084	7.56E-05
ZNF69	zinc finger protein 69	-0.838590217	7.86E-05
ZNF185	zinc finger protein 185 with LIM domain	-1.093861291	8.01E-05
PDK4	pyruvate dehydrogenase kinase 4	-1.138925402	8.10E-05
ST6GALNAC	ST6 N-acetylgalactosaminide alpha-2;6-sialyltransferase 3	-1.01613582	8.26E-05
VAC14-AS1	VAC14 antisense RNA 1	-1.645359392	8.52E-05
ARHGEF39	Rho guanine nucleotide exchange factor 39	-0.711709194	8.53E-05
ACSBG1	acyl-CoA synthetase bubblegum family member 1	1.124074214	8.63E-05

NTSR1	neurotensin receptor 1	-1.470573751	8.76E-05
GLB1L3	galactosidase beta 1 like 3	0.811240991	8.81E-05
ANKRD13C-	ANKRD13C divergent transcript	-0.662479717	8.82E-05
DGKB	diacylglycerol kinase beta	-2.467971258	8.83E-05
LINC01089	long intergenic non-protein coding RNA 1089	-0.870363623	8.84E-05
CACNA1A	calcium voltage-gated channel subunit alpha1 A	-0.813575391	8.88E-05
MHENCR	melanoma highly expressed competing endogenous lncRNA for miR-425 and miR-489	-1.060535143	9.34E-05
TOX-DT	TOX divergent transcript	-0.595393998	9.36E-05
GABRE	gamma-aminobutyric acid type A receptor subunit epsilon	1.436498424	9.48E-05
LINC03025	long intergenic non-protein coding RNA 3025	5.01912965	9.58E-05
SERPINF1	serpin family F member 1	-0.937204694	9.87E-05
MAST1	microtubule associated serine/threonine kinase 1	-0.945263014	0.0001012
ITGB7	integrin subunit beta 7	-2.342392359	0.00010231
LINC01905	long intergenic non-protein coding RNA 1905	5.532695323	0.00010234
TGFA	transforming growth factor alpha	-0.629333738	0.00010244
SHLD3	shieldin complex subunit 3	0.984112823	0.00010279
GRPR	gastrin releasing peptide receptor	-1.121968277	0.00010399
MCTP2	multiple C2 and transmembrane domain containing 2	2.090095374	0.00010533
DEPDC1B	DEP domain containing 1B	-0.603785781	0.00010585
FAM66C	family with sequence similarity 66 member C	-1.162090274	0.00010629
SSPN	sarcospan	0.803125603	0.00010643
NBL1	NBL1; DAN family BMP antagonist	-0.601710731	0.00010805
BMPER	BMP binding endothelial regulator	1.523734877	0.00011001
PTCHD1	patched domain containing 1	-0.736070499	0.00011177
SLC41A2	solute carrier family 41 member 2	1.09165829	0.0001128
CLCF1	cardiotrophin like cytokine factor 1	0.625524177	0.00011361
ZNF436-AS1	ZNF436 antisense RNA 1	-1.193565541	0.00011392
LPP-AS2	LPP antisense RNA 2	-1.233720528	0.00011499
ZNF487	zinc finger protein 487	-1.075059727	0.00011677
NME5	NME/NM23 family member 5	-0.853307929	0.00011981
GFOD1	Gfo/Idh/MocA-like oxidoreductase domain containing 1	0.672405734	0.00012138
LOC1249002	collagen alpha-1(III) chain	0.827458947	0.00012199
CABLES1	Cdk5 and Abl enzyme substrate 1	0.796448205	0.00012344
MAGI2-AS3	MAGI2 antisense RNA 3	-0.634439713	0.00012344
LOC1027247	uncharacterized LOC102724768	-3.692777818	0.00012424

A4GALT	alpha 1;4-galactosyltransferase (P blood group)	2.704042443	0.00012447
GRAMD2B	GRAM domain containing 2B	0.817440798	0.00012472
BMT2	base methyltransferase of 25S rRNA 2 homolog	0.719841271	0.00012568
ARNT2-DT	ARNT2 divergent transcript	-0.67029201	0.00012632
DENND2A	DENN domain containing 2A	-0.772615844	0.00013288
FAM72D	family with sequence similarity 72 member D	0.683335758	0.00013303
FAM131B	family with sequence similarity 131 member B	-1.30426575	0.00013382
TGFBI	transforming growth factor beta induced	0.933547858	0.00013436
MARCHF3	membrane associated ring-CH-type finger 3	1.304604912	0.00013885
GDPD1	glycerophosphodiester phosphodiesterase domain containing 1	0.728771573	0.00014021
HSD17B14	hydroxysteroid 17-beta dehydrogenase 14	-1.081527671	0.00014265
TMEM163	transmembrane protein 163	-0.846616405	0.00014278
IDNK	IDNK gluconokinase	-0.925783965	0.00014278
SELENOP	selenoprotein P	-4.108402214	0.00014437
NKD2	NKD inhibitor of WNT signaling pathway 2	1.065700684	0.00014764
PCDHB6	protocadherin beta 6	-1.924832397	0.00014875
PPM1J	protein phosphatase; Mg2+/Mn2+ dependent 1J	0.68076939	0.00015222
PHKG1	phosphorylase kinase catalytic subunit gamma 1	-1.39893589	0.0001556
PLD1	phospholipase D1	0.873860452	0.00015649
BCORL1	BCL6 corepressor like 1	-0.720019764	0.00015764
OLIG1	oligodendrocyte transcription factor 1	2.450995078	0.00016732
PRANCR	progenitor renewal associated non-coding RNA	-1.552063135	0.00016779
MORN5	MORN repeat containing 5	-5.344909731	0.00016879
ABLIM2	actin binding LIM protein family member 2	-1.757745596	0.00016922
FOXS1	forkhead box S1	2.647009715	0.00017331
SEMA3D	semaphorin 3D	-1.880413759	0.00017384
TMCO4	transmembrane and coiled-coil domains 4	0.813456106	0.00017493
BCHE	butyrylcholinesterase	-1.241015188	0.00018487
GNRH2	gonadotropin releasing hormone 2	2.882100585	0.00018746
PPP2R2B	protein phosphatase 2 regulatory subunit Bbeta	-0.912065852	0.00018782
MPP2	MAGUK p55 scaffold protein 2	-0.643275248	0.00018808

SANBR	SANT and BTB domain regulator of CSR	-0.604883501	0.00018961
PCDHB8	protocadherin beta 8	-1.296551528	0.00019024
RIPK4	receptor interacting serine/threonine kinase 4	2.828896463	0.00019044
MATCAP1	microtubule associated tyrosine carboxypeptidase 1	-0.601161995	0.00019076
ZNF394	zinc finger protein 394	0.621045743	0.00019169
IL7	interleukin 7	4.326336405	0.0001926
HERC3	HECT and RLD domain containing E3 ubiquitin protein ligase 3	0.623543233	0.00019264
MAN1A2	mannosidase alpha class 1A member 2	-0.64988362	0.00019377
PANX3	pannexin 3	2.168322032	0.00019661
PTPRN	protein tyrosine phosphatase receptor type N	2.652382838	0.00019945
FAIM2	Fas apoptotic inhibitory molecule 2	-1.366019272	0.00019994
CHRFAM7A	CHRNA7 (exons 5-10) and FAM7A (exons A-E) fusion	-1.741932684	0.00020197
DOK6	docking protein 6	-1.660799345	0.00020235
CYLD-AS1	CYLD antisense RNA 1	2.976452342	0.00020428
PAK6	p21 (RAC1) activated kinase 6	-1.283073954	0.00020646
KCNK15	potassium two pore domain channel subfamily K member 15	2.211152047	0.00021029
RORA	RAR related orphan receptor A	1.428562834	0.00021315
NAV3	neuron navigator 3	0.693494952	0.0002156
BAZ2B-AS1	BAZ2B antisense RNA 1	-1.149005284	0.00021796
UBD	ubiquitin D	4.372404951	0.00022022
MYO7A	myosin VIIA	-1.651006547	0.00022417
LYRM9	LYR motif containing 9	-1.139724857	0.0002287
CFAP95-DT	CFAP95 divergent transcript	3.749220599	0.00023173
BCAS1	brain enriched myelin associated protein 1	4.313484003	0.00023195
SCNN1G	sodium channel epithelial 1 subunit gamma	-1.457370838	0.00023681
CASP6	caspase 6	-0.736864521	0.00024456
CNTFR	ciliary neurotrophic factor receptor	-0.671690042	0.00024587
SERTM2	serine rich and transmembrane domain containing 2	2.278099976	0.00024751
CAHM	colon adenocarcinoma hypermethylated	-1.434225039	0.00024801
HGFAC	HGF activator	-1.680122049	0.00024827
PNMA8C	PNMA family member 8C	-0.743351032	0.00024848
LRRC32	leucine rich repeat containing 32	2.906939793	0.000249
RNF144A	ring finger protein 144A	-0.638042029	0.00025613
BBOF1	basal body orientation factor 1	-0.721894611	0.00025644
HRH2	histamine receptor H2	0.814303802	0.00025718
CDH8	cadherin 8	-2.286210477	0.00025975

BTD	biotinidase	-0.759992005	0.00026131
SLC2A11	solute carrier family 2 member 11	-0.628783846	0.00026538
CFAP221	cilia and flagella associated protein 221	-1.250868113	0.00027196
CD7	CD7 molecule	4.777566895	0.00027199
LAP3P2	leucine aminopeptidase 3 pseudogene 2	3.514251217	0.00027695
LINS1	lines homolog 1	0.620551964	0.00027818
TSPAN10	tetraspanin 10	-1.349579781	0.00028082
RASA4	RAS p21 protein activator 4	-0.652131408	0.00028145
FILIP1	filamin A interacting protein 1	-2.133302227	0.00028865
ITGA5	integrin subunit alpha 5	0.641570447	0.00029372
LINC00467	long intergenic non-protein coding RNA 467	-0.73923171	0.00029879
SPINT1	serine peptidase inhibitor; Kunitz type 1	1.434813239	0.00029906
PCDH20	protocadherin 20	-1.299796629	0.00029937
GPR139	G protein-coupled receptor 139	4.303610387	0.00030235
BEND7	BEN domain containing 7	-1.032071112	0.00030526
DNASE1	deoxyribonuclease 1	-0.623438904	0.00030704
SH3TC2	SH3 domain and tetratricopeptide repeats 2	-1.738383255	0.00031129
PECR	peroxisomal trans-2-enoyl-CoA reductase	-0.803177454	0.00031172
IQCH-AS1	IQCH antisense RNA 1	-1.087470861	0.00031266
S100A2	S100 calcium binding protein A2	0.671077424	0.00032178
NEIL1	nei like DNA glycosylase 1	-1.754857722	0.00032383
FOXO6	forkhead box O6	-1.51499102	0.00032452
PTHLH	parathyroid hormone like hormone	-0.612598916	0.00032518
SLC7A11	solute carrier family 7 member 11	0.673932047	0.00032628
LHX6	LIM homeobox 6	2.325050736	0.00032864
ADAM33	ADAM metallopeptidase domain 33	-3.674762049	0.00034317
LOC1249004	endogenous retrovirus group K member 5 Gag polyprotein-like	-2.445687143	0.00034956
DBP	D-box binding PAR bZIP transcription factor	-0.739952711	0.00035176
FRS3	fibroblast growth factor receptor substrate 3	-0.681234623	0.00035228
TLCD2	TLC domain containing 2	-0.972906481	0.00035383
PINLYP	phospholipase A2 inhibitor and LY6/PLAUR domain containing	1.689289982	0.00035794
BBS9	Bardet-Biedl syndrome 9	-0.751296927	0.00035897
MECOM	MDS1 and EVI1 complex locus	4.023168591	0.00036122
ZFP14	ZFP14 zinc finger protein	-0.803585616	0.00036179
TGM1	transglutaminase 1	1.033168254	0.00036179
GVQW3	GVQW motif containing 3	-0.78665434	0.00036286
MYEOV	myeloma overexpressed	3.248924709	0.00036434

SMCO4	single-pass membrane protein with coiled-coil domains 4	-0.782529042	0.00036739
LINC01686	long intergenic non-protein coding RNA 1686	3.495830137	0.00036878
LINC00907	long intergenic non-protein coding RNA 907	-1.985436957	0.00037199
SDHAF4	succinate dehydrogenase complex assembly factor 4	-0.642274801	0.00037243
NPTX1	neuronal pentraxin 1	-0.77872763	0.00037411
LOC1249053	uncharacterized LOC124905349	-4.760811539	0.00037935
DRC3	dynein regulatory complex subunit 3	-0.926965081	0.00038156
NRSN1	neurensin 1	-2.835487649	0.00038232
AMN	amnion associated transmembrane protein	1.358996557	0.00038473
PPARA	peroxisome proliferator activated receptor alpha	-0.920246109	0.00038765
LRRC27	leucine rich repeat containing 27	-0.657063175	0.00039067
UTP14C	UTP14C small subunit processome component	11.77794689	0.00039372
C8orf88	chromosome 8 open reading frame 88	-0.771815355	0.00039771
PKD1P5	polycystin 1; transient receptor potential channel interacting pseudogene 5	1.746122449	0.00039804
LINC00488	long intergenic non-protein coding RNA 488	-1.202289574	0.00040356
ADAMTSL4	ADAMTS like 4	0.611780469	0.00040611
CRIP1	cysteine rich protein 1	-3.125168293	0.00041399
SOX9-AS1	SOX9 antisense RNA 1	-1.152115634	0.00041453
ADPRH	ADP-ribosylarginine hydrolase	1.047600999	0.00041919
LINC02200	long intergenic non-protein coding RNA 2200	-1.535687218	0.00042988
ZNF578	zinc finger protein 578	-1.282540951	0.00043223
FLJ20021	uncharacterized LOC90024	-1.11923117	0.00043489
MYL9	myosin light chain 9	-1.096023404	0.00043745
ADGRB3	adhesion G protein-coupled receptor B3	-0.860115398	0.00043745
TMEM267	transmembrane protein 267	0.666037916	0.00044514
MYLK-AS1	MYLK antisense RNA 1	-1.048915291	0.00044604
PCDH10	protocadherin 10	-1.266027048	0.00044945
SLC6A20	solute carrier family 6 member 20	-1.185753654	0.00045314
KRT17	keratin 17	1.916647483	0.00046252
TACR2	tachykinin receptor 2	3.33757678	0.00046359
RALGPS1	Ral GEF with PH domain and SH3 binding motif 1	-1.113722098	0.00046499
SCNN1D	sodium channel epithelial 1 subunit delta	-0.996670189	0.00046932
ASPA	aspartoacylase	2.301344612	0.00046939

TNRC6C	trinucleotide repeat containing adaptor 6C	-0.614916952	0.00047012
ANKRD37	ankyrin repeat domain 37	0.925761957	0.0004727
DKKL1	dickkopf like acrosomal protein 1	-2.958953806	0.00047279
BCL11B	BCL11 transcription factor B	-1.876144016	0.00047826
G0S2	G0/G1 switch 2	0.752417329	0.00047826
STKLD1	serine/threonine kinase like domain containing 1	-1.873928664	0.00047861
PIRT	phosphoinositide interacting regulator of transient receptor potential channels	-3.086106927	0.00047881
PAG1	phosphoprotein membrane anchor with glycosphingolipid microdomains 1	-0.963537202	0.00048936
WNT11	Wnt family member 11	-1.535356951	0.00048936
LST1	leukocyte specific transcript 1	4.285133736	0.00049031
GCHFR	GTP cyclohydrolase I feedback regulator	0.864493156	0.00049546
COL1A1	collagen type I alpha 1 chain	0.864464067	0.00049901
PBLD	phenazine biosynthesis like protein domain containing	-0.600734267	0.00050363
STIMATE-M	STIMATE-MUSTN1 readthrough	1.047702646	0.0005039
RIOX1	ribosomal oxygenase 1	0.619282106	0.00050557
NR4A2	nuclear receptor subfamily 4 group A member 2	0.973652561	0.00050973
GP1BA	glycoprotein Ib platelet subunit alpha	3.33745193	0.00051249
L1CAM	L1 cell adhesion molecule	0.993852982	0.00052074
ZBTB7C	zinc finger and BTB domain containing 7C	-1.210491095	0.00052074
LEFTY2	left-right determination factor 2	-1.277390365	0.0005321
PFKFB2	6-phosphofructo-2-kinase/fructose-2;6-biphosphatase 2	-0.715108015	0.00053319
C17orf67	chromosome 17 open reading frame 67	1.141151235	0.00053512
INHBE	inhibin subunit beta E	3.487105875	0.00054422
IL17RC	interleukin 17 receptor C	0.596480407	0.0005528
PLB1	phospholipase B1	0.640333802	0.00055535
TMEM178B	transmembrane protein 178B	-0.703736682	0.00055749
TTC21B	tetratricopeptide repeat domain 21B	-0.953738851	0.00055763
SBNO1-AS1	SBNO1 antisense RNA 1	-1.39062197	0.00055863
ANKDD1A	ankyrin repeat and death domain containing 1A	-1.141292049	0.00056557
FBLL1	fibrillarin like 1	-0.601111115	0.00056801
PDE10A	phosphodiesterase 10A	2.067235086	0.00056801
PDZRN4	PDZ domain containing ring finger 4	-2.465627641	0.00057756
CDKN1C	cyclin dependent kinase inhibitor 1C	-1.066771447	0.00057811
TM6SF2	transmembrane 6 superfamily member 2	-1.06120993	0.00059043
TUBD1	tubulin delta 1	0.635355265	0.00059386

HLA-DOB	major histocompatibility complex; class II; DO beta	1.395647489	0.00059666
ABCD2	ATP binding cassette subfamily D member 2	-1.881435961	0.00060624
GSAP	gamma-secretase activating protein	2.251504913	0.00061207
B3GNT7	UDP-GlcNAc:betaGal beta-1;3-N-acetylglucosaminyltransferase 7	1.626446779	0.00062558
VWA3A	von Willebrand factor A domain containing 3A	-2.203614318	0.00062866
PRR22	proline rich 22	0.791276102	0.00063888
GMDS-DT	GMDS divergent transcript	-0.973287669	0.00064726
CRELD1	cysteine rich with EGF like domains 1	0.733625627	0.00065186
HS3ST3A1	heparan sulfate-glucosamine 3-sulfotransferase 3A1	0.615662051	0.00065654
TNFRSF10A	TNF receptor superfamily member 10a	1.131287295	0.00066763
KCNIP2	potassium voltage-gated channel interacting protein 2	-1.347023484	0.00067004
RAC2	Rac family small GTPase 2	2.001233298	0.00067173
ANKAR	ankyrin and armadillo repeat containing	1.615623022	0.00067275
LRRC37A2	leucine rich repeat containing 37 member A2	0.614347041	0.00067493
P2RY1	purinergic receptor P2Y1	-0.711585568	0.00067547
LRRC3B	leucine rich repeat containing 3B	-4.449587149	0.00067843
KIFC3	kinesin family member C3	0.602055817	0.0006816
FAM227B	family with sequence similarity 227 member B	-0.97815705	0.00068238
GREM1	gremlin 1; DAN family BMP antagonist	-1.253846891	0.00068398
LIF-AS2	LIF antisense RNA 2	1.357098794	0.00068693
MGAT5B	alpha-1;6-mannosylglycoprotein 6-beta-N-acetylglucosaminyltransferase B	-0.796694801	0.00069525
CARF	calcium responsive transcription factor	-0.714358928	0.00070526
CASC18	cancer susceptibility 18	-1.823219818	0.00070533
CCL8	C-C motif chemokine ligand 8	4.319328129	0.0007107
PCDHGB1	protocadherin gamma subfamily B; 1	-0.670713594	0.00071618
VIPR1	vasoactive intestinal peptide receptor 1	-1.303770972	0.00072821
ZNF561-AS1	ZNF561 antisense RNA 1 (head to head)	-0.614006092	0.00073038
HLA-DRB5	major histocompatibility complex; class II; DR beta 5	0.623093916	0.00073978
DCDC2	doublecortin domain containing 2	-1.839787386	0.00076825
SLC2A1-DT	SLC2A1 divergent transcript	-2.210015055	0.00076843
CFAP126	cilia and flagella associated protein 126	-0.949018364	0.00077004
OLFM1	olfactomedin 1	1.712144785	0.00077623
PROK2	prokineticin 2	1.14638832	0.0007835
ZNF232-AS1	ZNF232 antisense RNA 1	-1.470019585	0.00078402

KCNB1	potassium voltage-gated channel subfamily B member 1	-2.373789327	0.00078856
POT1-AS1	POT1 antisense RNA 1	-2.072589317	0.00079449
CDHR1	cadherin related family member 1	-0.619758106	0.00079583
ARHGEF37	Rho guanine nucleotide exchange factor 37	-0.828836024	0.00079656
PLEKHG2	pleckstrin homology and RhoGEF domain containing G2	-0.910204923	0.00079764
IL34	interleukin 34	1.758711794	0.00080232
CPA2	carboxypeptidase A2	4.460408926	0.00080729
CEMIP	cell migration inducing hyaluronidase 1	-0.656811296	0.00080946
AGAP2	ArfGAP with GTPase domain; ankyrin repeat and PH domain 2	-1.080672618	0.00083598
PLXDC2	plexin domain containing 2	-1.332447729	0.00084703
MND1	meiotic nuclear divisions 1	0.643909738	0.00084876
EIF4EBP3	eukaryotic translation initiation factor 4E binding protein 3	0.983447318	0.00085538
SP2-DT	SP2 divergent transcript	-1.078986525	0.00085538
FLJ32255	uncharacterized LOC643977	3.53287409	0.0008556
SPP1	secreted phosphoprotein 1	-1.455067142	0.00085894
ABHD18	abhydrolase domain containing 18	0.665389909	0.00086333
SETD1A	SET domain containing 1A; histone lysine methyltransferase	1.183238538	0.00086799
LINC01011	long intergenic non-protein coding RNA 1011	-1.130567494	0.00087431
ZNF529-AS1	ZNF529 antisense RNA 1	-1.009777578	0.00087688
FIGNL2	fidgetin like 2	-0.708594474	0.00088037
TTLL7	tubulin tyrosine ligase like 7	-0.618466488	0.00088826
MGAT3	beta-1;4-mannosyl-glycoprotein 4-beta-N-acetylglucosaminyltransferase	-0.835024182	0.00089146
MPP1	MAGUK p55 scaffold protein 1	-0.698643015	0.00089169
QPRT	quinolinate phosphoribosyltransferase	-2.400539457	0.00089648
CBLN1	cerebellin 1 precursor	-1.384927464	0.000898
MMP14	matrix metallopeptidase 14	-0.716141483	0.00090443
FST	follistatin	1.917290105	0.00093682
AMBN	ameloblastin	-1.103269453	0.00093833
PRKG1	protein kinase cGMP-dependent 1	-1.013864879	0.00093977
RHBDL2	rhomboid like 2	1.423170584	0.00094581
SMG1P1	SMG1 pseudogene 1	0.705121323	0.00094619
DRAXIN	dorsal inhibitory axon guidance protein	-0.618805294	0.00095305
PALS1	protein associated with LIN7 1; MAGUK p55 family member	-0.672305376	0.00095305
PIK3R5	phosphoinositide-3-kinase regulatory subunit 5	2.549674117	0.0009547
CMC4	C-X9-C motif containing 4	-0.93059517	0.00096715
ZNF446	zinc finger protein 446	-0.601601692	0.00096715

PAIP2B	poly(A) binding protein interacting protein 2B	-1.088779181	0.00098432
C9orf50	chromosome 9 open reading frame 50	-2.753919084	0.00098495
ZNF337-AS1	ZNF337 antisense RNA 1	-1.689943642	0.00101323
PRSS12	serine protease 12	-1.039008468	0.00102655
STAMBPL1	STAM binding protein like 1	0.682428208	0.00102772
DDO	D-aspartate oxidase	0.708596017	0.00104317
AGMAT	agmatinase	0.954687361	0.00105144
VPS13B-DT	VPS13B divergent transcript	-1.097909984	0.00105185
TMEM35B	transmembrane protein 35B	0.724879265	0.0010633
ZNF850	zinc finger protein 850	-1.012930592	0.00107061
LRRN4CL	LRRN4 C-terminal like	-1.48842832	0.00107353
ARMC3	armadillo repeat containing 3	-0.726462573	0.00107353
COL4A4	collagen type IV alpha 4 chain	2.076627237	0.00107411
ZNF319	zinc finger protein 319	-0.652143274	0.00107721
DGKI	diacylglycerol kinase iota	-0.96232323	0.00107858
USP13	ubiquitin specific peptidase 13	0.62695888	0.00108274
CRACDL	CRACD like	-1.075562453	0.00109792
TGDS	TDP-glucose 4;6-dehydratase	0.663581756	0.00111092
IMPG2	interphotoreceptor matrix proteoglycan 2	-2.948269586	0.00112602
CYTL1	cytokine like 1	-2.22331487	0.00113202
LY6S-AS1	LY6S antisense RNA 1	0.779579112	0.00113467
ZFAND2A-D	ZFAND2A divergent transcript	-0.649775394	0.00114608
FBXO24	F-box protein 24	-4.062442568	0.00115701
LINC03011	long intergenic non-protein coding RNA 3011	-1.165345483	0.00115763
TMPO-AS1	TMPO antisense RNA 1	-0.707557496	0.00115822
DNER	delta/notch like EGF repeat containing	1.031555297	0.0011749
NFE2L1-DT	NFE2L1 divergent transcript	-1.773106323	0.00118726
IFT172	intraflagellar transport 172	-0.629209092	0.0011905
ZNF808	zinc finger protein 808	0.690528198	0.00119969
LGALSL	galectin like	-0.646649975	0.00120135
SYCE1L	synaptonemal complex central element protein 1 like	-1.262929236	0.00120793
PTOV1-AS1	PTOV1 antisense RNA 1	-1.377058029	0.00122487
MAP3K6	mitogen-activated protein kinase kinase kinase 6	-0.668289525	0.00122618
SCN1A	sodium voltage-gated channel alpha subunit 1	-0.805859216	0.0012286
TRIM52-AS1	TRIM52 antisense RNA 1 (head to head)	-0.862968662	0.0012406
CYP21A2	cytochrome P450 family 21 subfamily A member 2	1.847288739	0.00125371
WNT10A	Wnt family member 10A	3.501508374	0.00126036
TEKT2	tektin 2	-1.005375006	0.00126286

JMJD7	jumonji domain containing 7	-0.714897049	0.0012651
RSC1A1	regulator of solute carriers 1	0.940444938	0.00127286
LINC01315	long intergenic non-protein coding RNA 1315	-1.211884286	0.00127916
GRAP	GRB2 related adaptor protein	-1.649293373	0.00128371
ADAMTS9-A	ADAMTS9 antisense RNA 2	-1.112402402	0.00128465
EDN2	endothelin 2	-1.147425538	0.00129866
ANKRD20A1	ankyrin repeat domain 20 family member A1	-4.045443689	0.00129929
NAP1L3	nucleosome assembly protein 1 like 3	-0.690600737	0.00132907
CPA4	carboxypeptidase A4	0.667354162	0.00134038
M1AP	meiosis 1 associated protein	3.528960487	0.00134274
TH	tyrosine hydroxylase	-3.543951113	0.00135387
IQGAP2	IQ motif containing GTPase activating protein 2	0.816577181	0.00136793
PSME2P1	proteasome activator subunit 2 pseudogene 1	2.079135613	0.00137869
SYNPO2	synaptopodin 2	1.582651848	0.00138498
MCM3AP-AS	MCM3AP antisense RNA 1	-0.662367445	0.00138789
ZCCHC12	zinc finger CCHC-type containing 12	-1.169454572	0.00140045
ATXN7	ataxin 7	0.901265129	0.0014009
VIPR2	vasoactive intestinal peptide receptor 2	4.041292118	0.00140952
GOLGA8N	golgin A8 family member N	-1.923190706	0.00143751
LINC01102	long intergenic non-protein coding RNA 1102	-1.367894313	0.00145142
PCDHB12	protocadherin beta 12	-1.260599195	0.00145715
LINC02541	long intergenic non-protein coding RNA 2541	2.208262009	0.00146125
PCDHGA6	protocadherin gamma subfamily A; 6	-0.950016146	0.00146205
TSPOAP1	TSPO associated protein 1	-1.598603117	0.00146626
CYTB	cytochrome b	-0.710134319	0.00146626
LOC1278142	RBM27-POU4F3	0.595295435	0.00148915
ZFAND2A	zinc finger AN1-type containing 2A	0.6197757	0.00149759
ACTG1P25	actin gamma 1 pseudogene 25	-2.062592985	0.00150061
RARA-AS1	RARA antisense RNA 1	-1.163711212	0.00151019
C16orf46	chromosome 16 open reading frame 46	1.228775691	0.00151606
ZBED3-AS1	ZBED3 antisense RNA 1	-0.795072143	0.00151861
TRIM73	tripartite motif containing 73	-1.334755011	0.00151891
RPP25	ribonuclease P and MRP subunit p25	0.753801741	0.00154639
OSBP2	oxysterol binding protein 2	-0.810627503	0.00155204
MT1F	metallothionein 1F	2.21341724	0.00158739
GPR88	G protein-coupled receptor 88	0.873154334	0.00159178
TSGA10	testis specific 10	-1.320840049	0.00159814
LINC00908	long intergenic non-protein coding RNA 908	3.847171783	0.00160319

LINC01138	long intergenic non-protein coding RNA 1138	1.160094972	0.00160857
RAB27B	RAB27B; member RAS oncogene family	2.325486542	0.00161309
ZNF425	zinc finger protein 425	-0.62506235	0.00163716
PAX5	paired box 5	5.221698403	0.00165236
CTDSPL	CTD small phosphatase like	-0.599750728	0.00165316
HLX	H2,0 like homeobox	1.478649645	0.00166696
MYRF	myelin regulatory factor	-1.121779093	0.00166912
KCNAB2	potassium voltage-gated channel subfamily A regulatory beta subunit 2	-0.840082058	0.00169545
SLC45A1	solute carrier family 45 member 1	-0.886989135	0.00171854
RBMS1	RNA binding motif single stranded interacting protein 1	0.735023972	0.00172221
LOC1053733	uncharacterized LOC105373383	-1.571000839	0.00174561
L3MBTL1	L3MBTL histone methyl-lysine binding protein 1	-0.685346448	0.001754
OSBPL7	oxysterol binding protein like 7	-0.596051803	0.00177915
ADGRA2	adhesion G protein-coupled receptor A2	-1.167584182	0.00178072
MELTF-AS1	MELTF antisense RNA 1	-1.075014003	0.00180947
CEP126	centrosomal protein 126	-0.910518107	0.00182122
AMN1	antagonist of mitotic exit network 1 homolog	-0.668679361	0.00182308
AXIN2	axin 2	-0.921114841	0.00182474
MINCR	MYC-induced long non-coding RNA	-0.813297219	0.00183641
TRAF3IP2-A	TRAF3IP2 antisense RNA 1	-0.651457773	0.00184689
TMEM130	transmembrane protein 130	-1.380241715	0.0018518
ESRRB	estrogen related receptor beta	1.728563895	0.00185456
LINC00487	long intergenic non-protein coding RNA 487	4.606929091	0.0018664
RLBP1	retinaldehyde binding protein 1	-2.163265147	0.00186998
TBCE	tubulin folding cofactor E	0.619503018	0.00187056
ADRA2B	adrenoceptor alpha 2B	1.532366157	0.00187063
CROCCP3	CROCC pseudogene 3	-1.095887917	0.00188987
LYPD5	LY6/PLAUR domain containing 5	2.30665823	0.00189715
LINC02018	long intergenic non-protein coding RNA 2018	-1.360486423	0.00189715
NYAP1	neuronal tyrosine phosphorylated phosphoinositide-3-kinase adaptor 1	-0.851653242	0.0019195
BANCR	BRAF-activated non-protein coding RNA	1.980178305	0.00195352
EHBP1-AS1	EHBP1 antisense RNA 1	-1.215826249	0.00195603
SNAI3	snail family transcriptional repressor 3	1.589974288	0.00197668
GPER1	G protein-coupled estrogen receptor 1	-1.069072293	0.0019836
CKMT1B	creatine kinase; mitochondrial 1B	2.044423126	0.00199172
SPSB2	splA/ryanodine receptor domain and SOCS box containing 2	-0.635644484	0.00203217
TNFAIP8	TNF alpha induced protein 8	0.927263809	0.0020431

VAX2	ventral anterior homeobox 2	-0.946332913	0.00204591
NFIA	nuclear factor I A	-0.638403707	0.00204813
SPATA13	spermatogenesis associated 13	1.091403841	0.00205452
ALG14	ALG14 UDP-N-acetylglucosaminyltransferase subunit	-0.62581115	0.00210868
CAMK2N1	calcium/calmodulin dependent protein kinase II inhibitor 1	1.143655159	0.00214684
PTGR2	prostaglandin reductase 2	-0.623537673	0.00215212
LOC1019273	uncharacterized LOC101927383	-0.65029799	0.00215366
CSAD	cysteine sulfinic acid decarboxylase	-0.741312646	0.00215799
PRDM1	PR/SET domain 1	0.794480054	0.00216433
C4orf19	chromosome 4 open reading frame 19	-1.882961599	0.00217035
INTS6-AS1	INTS6 antisense RNA 1	-0.901573942	0.00219282
MRPS22	mitochondrial ribosomal protein S22	0.620467663	0.0022025
BCYRN1	brain cytoplasmic RNA 1	-1.591834293	0.00220447
ZNF606-AS1	ZNF606 antisense RNA 1	-0.745659445	0.00220915
ST8SIA4	ST8 alpha-N-acetyl-neuraminide alpha-2;8-sialyltransferase 4	-1.275795385	0.00221625
WNT9B	Wnt family member 9B	-1.442441537	0.00223712
SRPX2	sushi repeat containing protein X-linked 2	0.824350871	0.0022565
MVP-DT	MVP divergent transcript	1.445571738	0.00226339
LSAMP	limbic system associated membrane protein	-0.608111443	0.0023021
NIPAL2	NIPA like domain containing 2	0.836320247	0.00231019
ARMH1	armadillo like helical domain containing 1	-1.142252571	0.00232587
PXMP4	peroxisomal membrane protein 4	-0.839215691	0.00234047
TNNC1	troponin C1; slow skeletal and cardiac type	-1.364791362	0.00234465
CASC2	cancer susceptibility 2	-0.665521576	0.00235316
TUNAR	TCL1 upstream neural differentiation-associated RNA	-0.852183373	0.00237572
SPEF1	sperm flagellar 1	-0.749031493	0.00237572
PRX	periaxin	-1.591074502	0.00238521
HES5	hes family bHLH transcription factor 5	-0.638845801	0.00239661
KCNK12	potassium two pore domain channel subfamily K member 12	-2.30949419	0.00240447
LETM2	leucine zipper and EF-hand containing transmembrane protein 2	-0.685618082	0.00240447
FOSB	FosB proto-oncogene; AP-1 transcription factor subunit	-1.266019198	0.00241543
ITGA4	integrin subunit alpha 4	0.779397948	0.00241912
DNAI1	dynein axonemal intermediate chain 1	-1.730280116	0.00247126
PMEPA1	prostate transmembrane protein; androgen induced 1	0.733744624	0.00247745

FANCB	FA complementation group B	0.691032338	0.00247872
KIAA1614	KIAA1614	-0.950154475	0.00250184
LINC01114	long intergenic non-protein coding RNA 1114	-2.330148489	0.00251395
PEAR1	platelet endothelial aggregation receptor 1	-2.735864766	0.00257825
LBHD1	LBH domain containing 1	0.662394659	0.00258906
LOC100507(	uncharacterized LOC100507053	-1.466221242	0.00259802
SLFN12L	schlafen family member 12 like	2.484755196	0.00261308
CACNA1I	calcium voltage-gated channel subunit alpha1 I	2.682425676	0.00261329
TICAM2	TIR domain containing adaptor molecule 2	1.535672228	0.00263483
GARIN5A	golgi associated RAB2 interactor 5A	-1.254517026	0.00266793
SPRED3	sprouty related EVH1 domain containing 3	0.655501469	0.00269705
VMAC	vimentin type intermediate filament associated coiled-coil protein	-0.908167667	0.00270884
CRB1	crumbs cell polarity complex component 1	-0.898203088	0.00273055
SLC27A6	solute carrier family 27 member 6	-2.210175937	0.00274086
FAT3	FAT atypical cadherin 3	-0.610901258	0.00284864
BTG3-AS1	BTG3 antisense RNA 1	-0.962905434	0.00285485
LINC01182	long intergenic non-protein coding RNA 1182	1.949001145	0.00286428
PCDHB14	protocadherin beta 14	-0.611044997	0.00289471
GDNF	glial cell derived neurotrophic factor	2.213150871	0.00289585
PCDHB7	protocadherin beta 7	-1.394586758	0.00292718
AKAP6	A-kinase anchoring protein 6	-0.622106975	0.0029315
PPM1H	protein phosphatase; Mg2+/Mn2+ dependent 1H	-2.107212973	0.00297012
OPLAH	5-oxoprolinase; ATP-hydrolysing	-0.629521717	0.00298113
CYGB	cytoglobin	-0.739947099	0.00298208
MAGEA2	MAGE family member A2	4.980566696	0.00298707
HGF	hepatocyte growth factor	3.103438381	0.00301661
CA4	carbonic anhydrase 4	-2.18293366	0.00302352
SH3D21	SH3 domain containing 21	-0.688910939	0.00304411
SNAI3-AS1	SNAI3 antisense RNA 1	-0.794506762	0.00304557
MAP1LC3C	microtubule associated protein 1 light chain 3 gamma	-0.826581345	0.0030587
OTULINL	OTU deubiquitinase with linear linkage specificity like	1.149690293	0.00305944
COPB2-DT	COPB2 divergent transcript	-0.998892164	0.00306761
MBNL1-AS1	MBNL1 antisense RNA 1	-0.967954135	0.0030699
ITGA1	integrin subunit alpha 1	1.424892409	0.00315437
SFMBT2	Scm like with four mbt domains 2	0.666432999	0.00318638

TP63	tumor protein p63	3.422661498	0.00319798
APOLD1	apolipoprotein L domain containing 1	-0.957279	0.00322476
JCAD	junctional cadherin 5 associated	0.7702124	0.00327487
ADRB1	adrenoceptor beta 1	1.474382558	0.00327901
LOC653653	adaptor related protein complex 1 subunit sigma 2 pseudogene	-7.250715967	0.00329
LARGE2	LARGE xylosyl- and glucuronyltransferase 2	1.393866596	0.00333707
WNT2B	Wnt family member 2B	-1.70752304	0.00334194
SLC6A1	solute carrier family 6 member 1	-2.772810482	0.00334986
LRP4-AS1	LRP4 antisense RNA 1	-2.844741284	0.00335916
EFHC2	EF-hand domain containing 2	-1.130707305	0.00336068
SNAP25	synaptosome associated protein 25	1.697460956	0.0034113
CHL1-AS2	CHL1 antisense RNA 2	-1.450905511	0.00343838
GCNA	germ cell nuclear acidic peptidase	-0.984684734	0.00344456
OPRL1	opioid related nociceptin receptor 1	-1.233823439	0.0034799
TRPC1	transient receptor potential cation channel subfamily C member 1	-0.632048749	0.00350338
FGF22	fibroblast growth factor 22	-1.549712667	0.00355114
CILK1	ciliogenesis associated kinase 1	1.09740358	0.00356082
FAM47E	family with sequence similarity 47 member E	-1.461228437	0.00359917
PSME2P2	proteasome activator subunit 2 pseudogene 2	2.111003817	0.0036405
LTBP4	latent transforming growth factor beta binding protein 4	-0.750433998	0.00371464
CIBAR2	CBY1 interacting BAR domain containing 2	-1.815452826	0.00371902
GOLGA8O	golgin A8 family member O	-1.369787384	0.00375342
HS3ST5	heparan sulfate-glucosamine 3-sulfotransferase 5	-2.00498541	0.00376213
CRACD	capping protein inhibiting regulator of actin dynamics	-0.914144586	0.00379716
ZC2HC1A	zinc finger C2HC-type containing 1A	-0.73934365	0.00380373
ZMAT4	zinc finger matrin-type 4	-1.739535953	0.00382567
SCGN	secretagogin; EF-hand calcium binding protein	-1.95870599	0.00384283
GP1BB	glycoprotein Ib platelet subunit beta	-4.758206317	0.00384823
ATP6AP1-D	ATP6AP1 divergent transcript	-1.167877986	0.00388223
CBFA2T3	CBFA2/RUNX1 partner transcriptional co-repressor 3	-1.660127776	0.00393713
IL15	interleukin 15	3.065430819	0.00393977
SLC24A1	solute carrier family 24 member 1	-0.717109626	0.00397236
ARAP2	ArfGAP with RhoGAP domain; ankyrin repeat and PH domain 2	0.7468963	0.00398216
FRMD6	FERM domain containing 6	0.67553009	0.00398563

CLMP	CXADR like membrane protein	1.068635262	0.00401487
TKTL1	transketolase like 1	-1.179402817	0.00402159
SYTL5	synaptotagmin like 5	-0.856375906	0.00402181
RSPH1	radial spoke head component 1	-0.665173394	0.00404907
PCDHGA5	protocadherin gamma subfamily A; 5	-1.205538827	0.00410628
KIF17	kinesin family member 17	-0.743413376	0.00420249
LINC00158	long intergenic non-protein coding RNA 158	2.470591565	0.00420577
FRRS1L	ferric chelate reductase 1 like	-1.311990773	0.00428583
SCNN1B	sodium channel epithelial 1 subunit beta	-1.300627628	0.00440497
LINC00887	long intergenic non-protein coding RNA 887	-2.375661674	0.00440497
EOLA2-DT	EOLA2 divergent transcript	-1.280638003	0.00443206
ANO7	anoctamin 7	0.897082619	0.00443875
RHEBL1	RHEB like 1	1.228617774	0.0044447
NRXN2	neurexin 2	-1.076787136	0.00448552
OLIG2	oligodendrocyte transcription factor 2	1.560237401	0.00448578
ST6GALNAC	ST6 N-acetylgalactosaminide alpha-2;6-sialyltransferase 2	-1.521022805	0.00450827
ZNF222	zinc finger protein 222	0.766117953	0.00450932
NGFR	nerve growth factor receptor	-1.284214053	0.00451003
SCML2	Scm polycomb group protein like 2	0.590948497	0.00451182
GPSM2	G protein signaling modulator 2	-0.764197186	0.00459965
NAALADL2	N-acetylated alpha-linked acidic dipeptidase like 2	-1.007478724	0.00461908
STX3	syntaxin 3	0.595756755	0.00467555
ADAM8	ADAM metallopeptidase domain 8	1.35229823	0.00468714
ARHGAP24	Rho GTPase activating protein 24	2.228508747	0.00474542
RPLP0P2	ribosomal protein lateral stalk subunit P0 pseudogene 2	3.084080039	0.00474619
LINC01111	long intergenic non-protein coding RNA 1111	-1.385093362	0.00475382
ACKR2	atypical chemokine receptor 2	-2.152897456	0.00475771
NFATC4	nuclear factor of activated T cells 4	-0.773622865	0.00477417
CYRIA	CYFIP related Rac1 interactor A	-1.072787978	0.0048074
TMEM221	transmembrane protein 221	-1.103774885	0.00483078
ZBTB47	zinc finger and BTB domain containing 47	-0.778163058	0.00484384
LINC01431	long intergenic non-protein coding RNA 1431	-2.73426278	0.00487484
NKAIN4	sodium/potassium transporting ATPase interacting 4	-0.618736531	0.00489547
ZNF426-DT	ZNF426 divergent transcript	-0.990939878	0.00492293
SPTSSB	serine palmitoyltransferase small subunit B	-1.618418784	0.00493275
APLN	apelin	-0.884316934	0.00499432

LDHD	lactate dehydrogenase D	-2.77621163	0.00509984
PCDHB13	protocadherin beta 13	-0.987358522	0.00511656
MYT1	myelin transcription factor 1	-1.56925898	0.00512937
ACTG1P17	actin gamma 1 pseudogene 17	-3.130101061	0.00513623
PRKN	parkin RBR E3 ubiquitin protein ligase	-1.546280075	0.00515341
ADAM21	ADAM metallopeptidase domain 21	-3.499043568	0.00518575
ALKAL2	ALK and LTK ligand 2	1.424279624	0.00521639
LINC02487	long intergenic non-protein coding RNA 2487	-1.712051036	0.0052258
CATSPERG	cation channel sperm associated auxiliary subunit gamma	1.495226092	0.00522648
FAM241A	family with sequence similarity 241 member A	0.933555415	0.00522741
FBLN2	fibulin 2	0.592128726	0.00525727
ATP2A1-AS1	ATP2A1 antisense RNA 1	-0.753543215	0.00535045
HECW2	HECT; C2 and WW domain containing E3 ubiquitin protein ligase 2	-0.931623677	0.0054092
FANK1	fibronectin type III and ankyrin repeat domains 1	-0.84085236	0.00544542
SSH3	slingshot protein phosphatase 3	-0.636333258	0.005454
CFAP20DC	CFAP20 domain containing	-1.506133677	0.00546619
RPL32P29	ribosomal protein L32 pseudogene 29	-0.895626662	0.00546619
ATOH8	atonal bHLH transcription factor 8	-0.65574406	0.00548398
P2RX6	purinergic receptor P2X 6	-0.969550121	0.00552132
ETV2	ETS variant transcription factor 2	-1.198034901	0.00556481
NRXN3	neurexin 3	-1.110898373	0.00556499
STING1	stimulator of interferon response cGAMP interactor 1	1.641075166	0.00557995
C3orf80	chromosome 3 open reading frame 80	1.31330248	0.00561972
SHC2	SHC adaptor protein 2	-0.848122643	0.00563366
ZNF491	zinc finger protein 491	-0.939270737	0.0056912
SRGN	serglycin	2.894081117	0.00573551
LINC00174	long intergenic non-protein coding RNA 174	-0.7118214	0.00582687
HLA-DQA1	major histocompatibility complex; class II; DQ alpha 1	1.642862581	0.00591605
LOC1053793	uncharacterized LOC105379362	-0.918899205	0.00594903
CCDC153	coiled-coil domain containing 153	-2.236027351	0.00604823
MCOLN2	mucolipin TRP cation channel 2	1.110912231	0.00606748
ENKUR	enkurin; TRPC channel interacting protein	-0.634335398	0.00609115
DHH	desert hedgehog signaling molecule	-1.760107512	0.00611965
RBP1	retinol binding protein 1	-0.682287393	0.00612996
INHBB	inhibin subunit beta B	-0.894065982	0.00614178
NFE2	nuclear factor; erythroid 2	-1.653440364	0.00616545
CLDN5	claudin 5	1.291638343	0.006199

ILRUN-AS1	ILRUN antisense RNA 1	-1.406941806	0.00620932
SPOCK2	SPARC (osteonectin); cwcv and kazal like domains proteoglycan 2	-0.643427638	0.0062154
LOC1249066	uncharacterized LOC124906608	-2.696751347	0.0062356
LINC01750	long intergenic non-protein coding RNA 1750	-0.784697019	0.00633096
ATP10B	ATPase phospholipid transporting 10B (putative)	-1.763794667	0.00633564
LINC00707	long intergenic non-protein coding RNA 707	-0.966766839	0.00641934
SYT7	synaptotagmin 7	0.763476555	0.00643536
TMEM150C	transmembrane protein 150C	-1.581846452	0.00645807
MIR17HG	miR-17-92a-1 cluster host gene	0.945487328	0.00658764
TOX2	TOX high mobility group box family member 2	-1.12420487	0.00660719
LRRTM3	leucine rich repeat transmembrane neuronal 3	-1.380020917	0.00660725
PDE1A	phosphodiesterase 1A	-2.979088484	0.00661307
SPRN	shadow of prion protein	-1.068940711	0.00674486
PTGS2	prostaglandin-endoperoxide synthase 2	5.480287259	0.00674622
LCP2	lymphocyte cytosolic protein 2	1.968823827	0.0067603
SNHG25	small nucleolar RNA host gene 25	-1.286372398	0.00683595
DSC3	desmocollin 3	-1.386333637	0.0068795
RCSD1	RCSD domain containing 1	-1.95173254	0.00700807
CALB1	calbindin 1	-0.980785892	0.00702479
LPAR1	lysophosphatidic acid receptor 1	-0.902105364	0.007052
SPMIP1	sperm microtubule inner protein 1	-1.108826294	0.00709546
CCDC65	coiled-coil domain containing 65	-1.305459394	0.00713648
EPCAM	epithelial cell adhesion molecule	1.293879477	0.00716251
NRG2	neuregulin 2	0.756442608	0.00724936
EEIG2	EEIG family member 2	-0.63387065	0.00734198
MIR9-3HG	MIR9-3 host gene	1.421845624	0.007363
CPM	carboxypeptidase M	1.010213789	0.007417
AHI1-DT	AHI1 divergent transcript	-1.55817435	0.00748054
HPS1-AS1	HPS1 antisense RNA 1	1.615033556	0.00748304
PLPPR1	phospholipid phosphatase related 1	-1.926705641	0.00748934
TMPRSS7	transmembrane serine protease 7	-2.00978924	0.00763243
DHRS12	dehydrogenase/reductase 12	-0.855280639	0.00766556
NPR3	natriuretic peptide receptor 3	2.455809811	0.0076867
TGFBR3	transforming growth factor beta receptor 3	1.480615157	0.00776928
LOC1079866	uncharacterized LOC107986626	-2.393966365	0.00779074
BAIAP3	BAI1 associated protein 3	-0.988742627	0.00784261
TIGD3	tigger transposable element derived 3	-1.031546679	0.00787803
LOC1053732	LOC105373289	-1.742406381	0.00788627

ARHGAP45	Rho GTPase activating protein 45	1.490714673	0.0078959
DACT1	dishevelled binding antagonist of beta catenin 1	-0.680061576	0.00793744
CCDC159	coiled-coil domain containing 159	-0.695340162	0.00793807
DNAAF1	dynein axonemal assembly factor 1	-0.936893135	0.00799872
HELB	DNA helicase B	0.673200741	0.00804616
CHADL	chondroadherin like	-1.346168143	0.00817823
LINC03033	long intergenic non-protein coding RNA 3033	1.914255984	0.00822933
CTSH	cathepsin H	-0.868492097	0.00827357
CA5B	carbonic anhydrase 5B	-0.647162281	0.00828053
PPP1R13B	protein phosphatase 1 regulatory subunit 13B	0.689078813	0.00839685
ADRA1A	adrenoceptor alpha 1A	-2.478516231	0.00840243
RESF1	retroelement silencing factor 1	0.640352906	0.00849625
DLC1	DLC1 Rho GTPase activating protein	-0.795557535	0.00850221
MESP2	mesoderm posterior bHLH transcription factor 2	2.421398808	0.00857142
TNFRSF6B	TNF receptor superfamily member 6b	8.946956209	0.00857313
TMEM102	transmembrane protein 102	1.266559214	0.00860807
CAMK2N2	calcium/calmodulin dependent protein kinase II inhibitor 2	-1.021922758	0.00863958
ZNF862	zinc finger protein 862	-0.606562112	0.00865514
PIP5KL1	phosphatidylinositol-4-phosphate 5-kinase like 1	-1.017715508	0.00866692
FOXP4-AS1	FOXP4 antisense RNA 1	-2.91303623	0.00876637
USP43	ubiquitin specific peptidase 43	0.768270558	0.00878745
MEIOB	meiosis specific with OB-fold	1.956674406	0.00887361
ABCG2	ATP binding cassette subfamily G member 2 (Junior blood group)	1.49601872	0.00894207
PITX3	paired like homeodomain 3	2.900801616	0.00895347
CD63-AS1	CD63 antisense RNA 1	-0.63308003	0.00899286
SAPCD1	suppressor APC domain containing 1	-1.045428965	0.00902215
SIX1	SIX homeobox 1	1.776641386	0.00903736
SNHG18	small nucleolar RNA host gene 18	-1.168985014	0.00903736
GALNT15	polypeptide N-acetylgalactosaminyltransferase 15	1.806426309	0.00905009
HRK	harakiri; BCL2 interacting protein	-1.171730519	0.00907527
GNAO1-DT	GNAO1 divergent transcript	-1.927139235	0.00912754
PCDHAC1	protocadherin alpha subfamily C; 1	-1.20705466	0.00920445
CLSTN2	calsyntenin 2	-2.515320911	0.0092434
KCNH1	potassium voltage-gated channel subfamily H member 1	-1.138094857	0.00927504
SEC24B-AS	SEC24B antisense RNA 1	-1.03858659	0.00928076
LIMS3	LIM zinc finger domain containing 3	-0.752170453	0.00931229
CCDC122	coiled-coil domain containing 122	1.178330474	0.00931945

DZIP1L	DAZ interacting zinc finger protein 1 like	-0.622033608	0.00936008
MAPK8IP3	mitogen-activated protein kinase 8 interacting protein 3	-0.646787176	0.00937568
PCP4	Purkinje cell protein 4	-1.1040528	0.00937568
LUM	lumican	-1.522226206	0.00938708
HYKK	hydroxylysine kinase	-1.372166183	0.00939025
CEROX1	cytoplasmic endogenous regulator of oxidative phosphorylation 1	-0.658585749	0.00939669
TEX22	testis expressed 22	-1.15291261	0.00946409
PTPRR	protein tyrosine phosphatase receptor type R	2.749682297	0.00948519
TRPM3	transient receptor potential cation channel subfamily M member 3	-1.551329936	0.00949621
CAMK2A	calcium/calmodulin dependent protein kinase II alpha	-1.48480227	0.00950994
GCNT3	glucosaminyl (N-acetyl) transferase 3; mucin type	3.635959605	0.00958328
ICAM4	intercellular adhesion molecule 4 (Landsteiner-Wiener blood group)	1.839100993	0.00960836
PCDHB11	protocadherin beta 11	-0.964056624	0.00982574
TTC41P	tetratricopeptide repeat domain 41; pseudogene	-1.399798257	0.00994737
FEZF1	FEZ family zinc finger 1	-0.71500294	0.00995249
C1QTNF12	C1q and TNF related 12	-1.588500744	0.0101355
NTN4	netrin 4	-1.010896401	0.01019314
ELAVL4	ELAV like RNA binding protein 4	-2.005944394	0.01019892
GABRG3	gamma-aminobutyric acid type A receptor subunit gamma3	-0.663346248	0.01029912
TNFRSF11A	TNF receptor superfamily member 11a	1.77736191	0.01033331
CIMIP2C	ciliary microtubule inner protein 2C	1.253116666	0.01033532
SELPLG	selectin P ligand	-0.995197365	0.01035456
TCAF2	TRPM8 channel associated factor 2	3.272456555	0.01038287
MIR3936HG	MIR3936 host gene	-1.469183655	0.01038884
LOH12CR2	loss of heterozygosity on chromosome 12; region 2	-1.39951617	0.01045558
ANKRD24	ankyrin repeat domain 24	-1.099920754	0.01047899
RILP	Rab interacting lysosomal protein	0.765569601	0.01052559
WNK4	WNK lysine deficient protein kinase 4	2.015858899	0.01061142
CPED1	cadherin like and PC-esterase domain containing 1	2.395466432	0.01062621
CSPG4P12	chondroitin sulfate proteoglycan 4 pseudogene 12	-1.246701426	0.01064242
MUC16	mucin 16; cell surface associated	1.98615806	0.01066099
FAT2	FAT atypical cadherin 2	-0.809628552	0.01071043
ULK2	unc-51 like autophagy activating kinase 2	-0.639663433	0.01084417

ATP8	ATP synthase F0 subunit 8	-0.598600972	0.01087846
C16orf96	chromosome 16 open reading frame 96	-1.376289986	0.01093559
LOC729998	eukaryotic translation elongation factor 1 gamma pseudogene	-0.606746095	0.01110099
IER3-AS1	IER3 antisense RNA 1	-1.610034537	0.01115472
LINC02021	long intergenic non-protein coding RNA 2021	-2.052713135	0.01119726
ENTPD3-AS	ENTPD3 antisense RNA 1	-0.721226028	0.01126403
PCDHGA11	protocadherin gamma subfamily A; 11	-1.8296955	0.01130801
ANKRD23	ankyrin repeat domain 23	-0.921598481	0.01132601
AVIL	advillin	-0.892858251	0.01134926
ZNF214	zinc finger protein 214	-1.109784598	0.01146195
CTNNA1-AS	CTNNA1 antisense RNA 1	-2.703905339	0.01146553
SRCIN1	SRC kinase signaling inhibitor 1	-0.800065418	0.01151941
ASAH1-AS1	ASAH1 antisense RNA 1	-1.40240085	0.0116955
P2RY2	purinergic receptor P2Y2	-0.958440922	0.0117001
H2BC8	H2B clustered histone 8	0.901321358	0.01174719
PCDHGA1	protocadherin gamma subfamily A; 1	-0.889086232	0.0117741
TNFSF12	TNF superfamily member 12	-0.720569608	0.01183894
KLHL4	kelch like family member 4	-1.998378555	0.01185549
MFNG	MFNG O-fucosylpeptide 3-beta-N-acetylglucosaminyltransferase	-1.510763572	0.01190103
GIPR	gastric inhibitory polypeptide receptor	-2.737558903	0.01191131
PTAFR	platelet activating factor receptor	2.314730411	0.01196582
LINC02076	long intergenic non-protein coding RNA 2076	-2.865286299	0.01198295
FBXO38-DT	FBXO38 divergent transcript	-1.628785638	0.01198295
MIA	MIA SH3 domain containing	2.532621053	0.01199951
RAB20	RAB20; member RAS oncogene family	0.762007955	0.01204503
PKN2-AS1	PKN2 antisense RNA 1	1.740729027	0.01207032
MTATP6P1	MT-ATP6 pseudogene 1	-0.636515903	0.01214408
CLGN	calmegin	-0.976897762	0.0121607
LRAT	lecithin retinol acyltransferase	-0.782219281	0.01219488
CD200	CD200 molecule	-2.39345526	0.01223069
LOC1053791	uncharacterized LOC105379199	-2.468559868	0.01228796
CNNM1	cyclin and CBS domain divalent metal cation transport mediator 1	0.652690914	0.01229492
CALY	calcyon neuron specific vesicular protein	-1.206546393	0.01233856
LOC1249044	uncharacterized LOC124904411	1.359644294	0.01246647
MAL	mal; T cell differentiation protein	0.926282056	0.01258435
C1orf232	chromosome 1 open reading frame 230	-2.817406923	0.01291473
DYSF	dysferlin	1.733989799	0.01296526
MYO15B	myosin XVB	-0.86211125	0.01298214
SNX32	sorting nexin 32	-0.781435983	0.01301216

OXTR	oxytocin receptor	0.620241301	0.01312585
WDR5B-DT	WDR5B divergent transcript	-1.156476882	0.01319935
KRT16	keratin 16	3.068418296	0.01323444
BEST3	bestrophin 3	-0.685765553	0.01327976
CFAP54	cilia and flagella associated protein 54	-0.897630842	0.01347586
NPTN-IT1	NPTN intronic transcript 1	-0.971360428	0.01354403
MAGOH-DT	MAGOH divergent transcript	-1.632640458	0.01367793
SAXO2	stabilizer of axonemal microtubules 2	-1.38155354	0.0137461
SLC24A3	solute carrier family 24 member 3	-1.972114816	0.01375015
ZNF575	zinc finger protein 575	-0.95865505	0.01389398
AK3P3	AK3 pseudogene 3	-1.926808344	0.01404552
HS1BP3	HCLS1 binding protein 3	-1.221935933	0.01413622
PTPRD-AS1	PTPRD antisense RNA 1	-0.984864176	0.01419454
GTF2IRD2P	GTF2I repeat domain containing 2 pseudogene 1	-1.953470475	0.01423889
FBLIM1	filamin binding LIM protein 1	-1.333721622	0.01426548
LINC02192	long intergenic non-protein coding RNA 2192	-1.255073105	0.01452011
VWA3B	von Willebrand factor A domain containing 3B	-1.152291999	0.01455839
EXTL1	exostosin like glycosyltransferase 1	-2.822537124	0.01460654
CARMIL3	capping protein regulator and myosin 1 linker 3	-0.915227646	0.01468966
ZNF843	zinc finger protein 843	-1.201346579	0.01468999
PAMR1	peptidase domain containing associated with muscle regeneration 1	-1.789328691	0.01472681
KY	kyphoscoliosis peptidase	-1.143680175	0.0147852
BCDIN3D-AS	BCDIN3D antisense RNA 1	-1.704145175	0.01480619
SMIM5	small integral membrane protein 5	-1.003116096	0.01487115
RSPH14	radial spoke head 14 homolog	-1.250022936	0.01487243
TMSB15B	thymosin beta 15B	-0.838522784	0.01489357
KIF26A	kinesin family member 26A	-1.239503788	0.01496334
LOC1225267	uncharacterized LOC122526782	0.875937541	0.01503505
GABRP	gamma-aminobutyric acid type A receptor subunit pi	2.608029288	0.01539162
IRS4	insulin receptor substrate 4	-1.723740692	0.01549667
HABP2	hyaluronan binding protein 2	-2.442368884	0.01556079
WNT10B	Wnt family member 10B	1.738326767	0.01559859
KSR1	kinase suppressor of ras 1	0.666777432	0.01561442
PAFAH1B2P	PAFAH1B2 pseudogene 2	-1.033345878	0.01567993
TCIRG1	T cell immune regulator 1; ATPase H+ transporting V0 subunit a3	0.709034041	0.01590946
MAGEE1	MAGE family member E1	-0.899000225	0.01591654
ZBTB46	zinc finger and BTB domain containing 46	-1.023741606	0.01592495

SPNS2	SPNS lysolipid transporter 2; sphingosine-1-phosphate	-2.343216401	0.01597341
ATP6V1C2	ATPase H+ transporting V1 subunit C2	1.450566484	0.01604559
LURAP1L	leucine rich adaptor protein 1 like	1.603244408	0.01617548
LOC729870	uncharacterized LOC729870	-1.655617643	0.01635109
FBN3	fibrillin 3	-1.817054811	0.01637923
RASEF	RAS and EF-hand domain containing	0.816420725	0.0164221
RGPD1	RANBP2 like and GRIP domain containing 1	3.187454521	0.01644944
PAPLN	papilin; proteoglycan like sulfated glycoprotein	-1.150641103	0.01651972
KAZALD1	Kazal type serine peptidase inhibitor domain 1	-0.590300445	0.016593
CYP46A1	cytochrome P450 family 46 subfamily A member 1	-0.59037305	0.0166044
EMC1-AS1	EMC1 antisense RNA 1	-2.157030968	0.01707949
PIERCE2	piercer of microtubule wall 2	-1.415041291	0.01732911
UGT8	UDP glycosyltransferase 8	-0.633332381	0.01737724
ENPP1	ectonucleotide pyrophosphatase/phosphodiesterase 1	0.739433192	0.01746878
QRFPR	pyroglutamylated RFamide peptide receptor	2.373996149	0.01747735
LINC00606	long intergenic non-protein coding RNA 606	-2.177581267	0.0175135
PTPRM	protein tyrosine phosphatase receptor type M	-0.755975542	0.01751384
CCR3	C-C motif chemokine receptor 3	3.30724147	0.01768658
RPL23AP49	ribosomal protein L23a pseudogene 49	-2.103588262	0.01768658
FRG1-DT	FRG1 divergent transcript	-1.897391465	0.01770303
TOGARAM2	TOG array regulator of axonemal microtubules 2	-1.027410003	0.01770303
ANXA2R-AS	ANXA2R antisense RNA 1	1.916588407	0.01783898
PCDHB3	protocadherin beta 3	-1.27905262	0.01784967
EGFL8	EGF like domain multiple 8	-0.730070311	0.01793355
RASGRF2-A	RASGRF2 antisense RNA 1	-0.987422402	0.01793455
RGS14	regulator of G protein signaling 14	0.734974162	0.01800043
SPOCK1	SPARC (osteonectin); cwcv and kazal like domains proteoglycan 1	-0.71266813	0.01811554
MROH8	maestro heat like repeat family member 8	-0.867862469	0.01820741
LOC101928(	uncharacterized LOC101928059	-2.295580667	0.0182512
PDK1-AS1	PDK1 and ITGA6 antisense RNA 1	1.889687732	0.01826296
WDFY3-AS2	WDFY3 antisense RNA 2	-0.623730565	0.01832156
HS3ST1	heparan sulfate-glucosamine 3-sulfotransferase 1	1.964626971	0.01835198
KHDC1-AS1	KHDC1 antisense RNA 1	-0.641842389	0.0183532

LDAF1	lipid droplet assembly factor 1	-1.136637019	0.01872416
LRRC2	leucine rich repeat containing 2	-0.615880363	0.0187334
GPR146	G protein-coupled receptor 146	1.192783212	0.01880576
ZDHHC22	zinc finger DHHC-type palmitoyltransferase 22	-1.475676898	0.01917307
FAM156B	family with sequence similarity 156 member B	-0.932877175	0.01918303
SYTL1	synaptotagmin like 1	-0.754056977	0.01925589
VXN	vexin	1.631947066	0.01944622
CLDND2	claudin domain containing 2	-1.436871574	0.01944816
ITGA2B	integrin subunit alpha 2b	-2.37419075	0.01972633
ANKRD2	ankyrin repeat domain 2	-1.158293326	0.01979213
ANKRD36C	ankyrin repeat domain 36C	-1.088894464	0.01980599
C2orf92	chromosome 2 open reading frame 92	-0.926556882	0.01981712
C16orf95-DT	C16orf95 divergent transcript	-1.905007123	0.01988272
EFCC1	EF-hand and coiled-coil domain containing 1	-2.880582407	0.02001264
HID1	HID1 domain containing	-0.686876436	0.02005654
LOC730183	uncharacterized LOC730183	-0.873380902	0.02009024
ZNF497	zinc finger protein 497	-0.650483476	0.02009038
ESRRG	estrogen related receptor gamma	-2.177443798	0.02012351
COL6A3	collagen type VI alpha 3 chain	3.415482709	0.02013352
RCAN2	regulator of calcineurin 2	-0.74503016	0.02016534
OSR1	odd-skipped related transcription factor 1	-1.943785301	0.02029088
LAMA5-AS1	LAMA5 antisense RNA 1	-1.952719036	0.02064434
LOC154761	family with sequence similarity 115; member C pseudogene	1.428675266	0.02071603
RIMBP3	RIMS binding protein 3	-1.168796885	0.02117027
KLRG2	killer cell lectin like receptor G2	0.727062307	0.02137155
SLC26A11	solute carrier family 26 member 11	-0.6072113	0.02140767
FGFR4	fibroblast growth factor receptor 4	-0.609731808	0.02147745
FGGY	FGGY carbohydrate kinase domain containing	-0.683064866	0.02153616
LINC01563	long intergenic non-protein coding RNA 1563	-2.22803262	0.02170291
GSTT2	glutathione S-transferase theta 2 (gene/pseudogene)	-1.279435441	0.02170703
SEZ6L2	seizure related 6 homolog like 2	-0.635075568	0.0217245
SH2D6	SH2 domain containing 6	-1.665559942	0.02183901
DNAI7	dynein axonemal intermediate chain 7	-0.925654121	0.02190582
STS	steroid sulfatase	1.435364803	0.02195911
GPRASP1	G protein-coupled receptor associated sorting protein 1	-0.753594787	0.02196383
OXCT1-AS1	OXCT1 antisense RNA 1	-1.096162137	0.02199429

ENTPD1	ectonucleoside triphosphate diphosphohydrolase 1	-1.584170453	0.02208808
AK8	adenylate kinase 8	-0.950622203	0.02223456
TMEM181	transmembrane protein 181	-2.492646835	0.02225562
PDE9A	phosphodiesterase 9A	-0.696996561	0.02226518
MGAT4A	alpha-1;3-mannosyl-glycoprotein 4-beta-N-acetylglucosaminyltransferase A	0.910870181	0.02232814
LINC00525	long intergenic non-protein coding RNA 525	3.035131063	0.02250351
DNAH12	dynein axonemal heavy chain 12	-1.657625084	0.02262004
PATJ	PATJ crumbs cell polarity complex component	-0.681013168	0.02263001
EFCAB11	EF-hand calcium binding domain 11	-0.620291828	0.02267494
COLEC12	collectin subfamily member 12	-0.797923141	0.02271197
MST1L	macrophage stimulating 1 like (pseudogene)	-1.172873954	0.02272666
LNC-LBCS	lncRNA bladder and prostate cancer suppressor; hnRNPK interacting	-1.023079208	0.02276061
RASA4	RAS p21 protein activator 4	-0.777324105	0.0228108
CCDC169	coiled-coil domain containing 169	-1.264678451	0.02294961
ASGR1	asialoglycoprotein receptor 1	-1.281768278	0.02298172
SMIM14-DT	SMIM14 divergent transcript	-1.59416568	0.0230608
LTB4R	leukotriene B4 receptor	0.959878104	0.02308421
ABCC9	ATP binding cassette subfamily C member 9	-1.503629684	0.0231447
FOXP2	forkhead box P2	-1.623097226	0.02355301
TBX15	T-box transcription factor 15	1.516107384	0.02358491
MSS51	MSS51 mitochondrial translational activator	-0.957420462	0.02361154
MEIOC	meiosis specific with coiled-coil domain	1.570020315	0.02363378
CCDC183-A	CCDC183 antisense RNA 1	0.834843883	0.02367113
RDM1	RAD52 motif containing 1	-1.003510224	0.02368176
ZNF560	zinc finger protein 560	0.839606607	0.02374441
LOC100419	zinc finger protein 41 pseudogene	1.556234246	0.02384754
MINAR1	membrane integral NOTCH2 associated receptor 1	0.603987723	0.02394531
MRO	maestro	-1.52283685	0.02398549
CFAP70	cilia and flagella associated protein 70	-0.765474616	0.02399303
PROCA1	protein interacting with cyclin A1	-0.846029532	0.02411349
LINC02606	long intergenic non-protein coding RNA 2606	-0.890090468	0.02415255
SBF2-AS1	SBF2 antisense RNA 1	-1.037535726	0.02442923
MISP	mitotic spindle positioning	2.012156028	0.02456744
ADCY5	adenylate cyclase 5	-1.43496078	0.02467364
KCTD21-AS	KCTD21 antisense RNA 1	-0.935572401	0.02471931
TSACC	TSSK6 activating cochaperone	-2.307412642	0.02473699

NTN5	netrin 5	-1.656884427	0.02475267
DNASE1L2	deoxyribonuclease 1 like 2	-0.879988433	0.02487196
ADAMTS4	ADAM metallopeptidase with thrombospondin type 1 motif 4	-1.003363176	0.02505146
TTC23L	tetratricopeptide repeat domain 23 like	-1.456271252	0.02508061
ALPK1	alpha kinase 1	1.368230248	0.02508568
UPK2	uroplakin 2	-1.623623759	0.02514227
PPP1R14B-/	PPP1R14B antisense RNA 1	-0.722353116	0.02516188
PKDCC	protein kinase domain containing; cytoplasmic	-0.735272336	0.02517214
DMC1	DNA meiotic recombinase 1	-2.646139804	0.02534113
LINC00638	long intergenic non-protein coding RNA 638	-0.929885201	0.02542217
H4C4	H4 clustered histone 4	2.408844812	0.0254259
SNORC	secondary ossification center associated regulator of chondrocyte maturation	-0.906734184	0.02556272
DPP3-DT	DPP3 divergent transcript	-0.924050012	0.02556451
ISLR2	immunoglobulin superfamily containing leucine rich repeat 2	1.228594249	0.02614652
HS3ST4	heparan sulfate-glucosamine 3-sulfotransferase 4	-0.818606523	0.02652133
DNAH9	dynein axonemal heavy chain 9	-0.706013893	0.02671457
TMEM26	transmembrane protein 26	-2.192468713	0.02674756
PARM1	prostate androgen-regulated mucin-like protein 1	-1.268342359	0.02677495
LRCOL1	leucine rich colipase like 1	-0.861525862	0.0268269
MMEL1	membrane metalloendopeptidase like 1	-1.80604123	0.02685495
LINC00310	long intergenic non-protein coding RNA 310	-1.827889126	0.02689745
GRID2IP	Grid2 interacting protein	-2.358545914	0.02691623
ZFP69B	ZFP69 zinc finger protein B	-0.658120877	0.02692538
PNPLA7	patatin like phospholipase domain containing 7	-0.788132693	0.02704347
TMEM106A	transmembrane protein 106A	0.960251593	0.02710623
KNDC1	kinase non-catalytic C-lobe domain containing 1	-2.535938244	0.02711872
CFAP74	cilia and flagella associated protein 74	-1.351160978	0.02714733
POMK	protein O-mannose kinase	0.953216099	0.02717271
GALR2	galanin receptor 2	1.348864116	0.02720642
SEMA4A	semaphorin 4A	-2.007449786	0.02721374
DOCK10	dedicator of cytokinesis 10	1.422561873	0.0273712
PBX4	PBX homeobox 4	-0.916621171	0.02750369
ECEL1	endothelin converting enzyme like 1	1.734037086	0.02753822
LINC02615	long intergenic non-protein coding RNA 2615	-2.058834788	0.02761801

LRRC7	leucine rich repeat containing 7	-1.845159857	0.02775739
LOC1249009	uncharacterized LOC124900957	-2.310455126	0.02782763
JMJD1C-AS	JMJD1C antisense RNA 1	-2.083104299	0.02791174
LRRC14B	leucine rich repeat containing 14B	-1.42051339	0.02793292
DPP4	dipeptidyl peptidase 4	2.542874372	0.0280143
MYO1G	myosin IG	2.348427878	0.02814308
RBMS3	RNA binding motif single stranded interacting protein 3	-0.923720081	0.02817756
DKFZP434A	uncharacterized LOC26102	-2.020622075	0.02843714
MDGA2	MAM domain containing glycosylphosphatidylinositol anchor 2	-0.688186027	0.02854551
PRPH	peripherin	1.444243889	0.02864071
OBSCN	obscurin; cytoskeletal calmodulin and titin-interacting RhoGEF	-0.649797011	0.02871858
ATL1	atlastin GTPase 1	-0.666405863	0.02874318
EML5	EMAP like 5	0.795837547	0.02894785
LPAR3	lysophosphatidic acid receptor 3	0.904360721	0.02922418
RPLP0P6	ribosomal protein lateral stalk subunit P0 pseudogene 6	-1.474538176	0.02935642
PCDHGB3	protocadherin gamma subfamily B; 3	-1.431747612	0.02946591
ZNF345	zinc finger protein 345	-0.652745273	0.02956149
BBOX1-AS1	BBOX1 antisense RNA 1	1.891730724	0.02994228
FTCD	formimidoyltransferase cyclodeaminase	-1.280600615	0.02997888
CCDC190	coiled-coil domain containing 190	-2.790738524	0.03011938
PRIMA1	proline rich membrane anchor 1	-1.052792382	0.03020894
RHBDL1	rhomboid like 1	-0.730158264	0.03032651
MYCNOS	MYCN opposite strand	-2.033973439	0.03051774
B3GAT1-DT	B3GAT1 divergent transcript	-2.636075611	0.03056603
CLEC18B	C-type lectin domain family 18 member B	-0.854193955	0.0305795
TMEM151B	transmembrane protein 151B	-1.589075202	0.03074062
NAGS	N-acetylglutamate synthase	1.024980954	0.03074764
KIAA0319	KIAA0319	-1.081734841	0.03100103
LINC00173	long intergenic non-protein coding RNA 173	1.146866095	0.03108776
SAMD13	sterile alpha motif domain containing 13	-1.007676023	0.03146516
KCNT2	potassium sodium-activated channel subfamily T member 2	0.815712508	0.03173887
NTNG2	netrin G2	1.138483441	0.03193882
PDGFB	platelet derived growth factor subunit B	-0.801337202	0.03257771
SLC25A5-AS	SLC25A5 antisense RNA 1	-0.869921803	0.03262672
H4C14	H4 clustered histone 14	0.984980028	0.03268421
TSC22D3	TSC22 domain family member 3	-1.211424687	0.03283591
GOT1-DT	GOT1 divergent transcript	-0.862056892	0.0332319
MYH7B	myosin heavy chain 7B	-1.825749829	0.03329368

NMU	neuromedin U	-1.429757925	0.03334468
RTP1	receptor transporter protein 1	-2.59308664	0.03338767
MCF2L	MCF,2 cell line derived transforming sequence like	-1.166857723	0.03376939
RORA-AS1	RORA antisense RNA 1	-1.63618513	0.03389428
CPB2-AS1	CPB2 antisense RNA 1	-0.776483542	0.0339491
SERPINI2	serpin family I member 2	2.032020546	0.03427876
TMEM229A	transmembrane protein 229A	-1.461543231	0.03433194
RTL9	retrotransposon Gag like 9	-0.864234143	0.03455516
SLC4A4	solute carrier family 4 member 4	-0.633463096	0.03456502
PCLO	piccolo presynaptic cytomatrix protein	0.924036608	0.03467271
S100A13	S100 calcium binding protein A13	0.599077895	0.03470462
VRK2	VRK serine/threonine kinase 2	2.771946317	0.03474926
POLR2J4	RNA polymerase II subunit J4 (pseudogene)	-0.935704477	0.03489583
SLC9A7P1	solute carrier family 9 member 7 pseudogene 1	-2.154909647	0.03509364
TLE6	TLE family member 6; subcortical maternal complex member	-1.163019429	0.03509364
DYNC1I1	dynein cytoplasmic 1 intermediate chain 1	-1.368928272	0.0352634
CHRD	chordin	-1.907619028	0.0353577
RNF207-AS1	RNF207 antisense RNA 1	-0.625879618	0.03540466
TNNI3K	TNNI3 interacting kinase	-2.823003401	0.03543203
LOC1001322	uncharacterized LOC100132249	-0.64851996	0.03559171
LYPD6B	LY6/PLAUR domain containing 6B	0.795587032	0.03559516
SPEF2	sperm flagellar 2	-0.702326238	0.03560413
ANXA2R-OT	ANXA2R overlapping transcript 1	-1.074844652	0.03566477
ANKUB1	ankyrin repeat and ubiquitin domain containing 1	-0.992671293	0.03594508
CYP2E1	cytochrome P450 family 2 subfamily E member 1	-1.431871781	0.03602757
GATA2	GATA binding protein 2	0.678035491	0.03610116
PANX2	pannexin 2	0.663025695	0.03615231
SEMA3G	semaphorin 3G	-0.724619924	0.03618009
GOLGA6L4	golgin A6 family like 4	-1.131262346	0.03620864
NUDT16-DT	NUDT16 divergent transcript	-1.874989096	0.03620864
KIAA0825	KIAA0825	-1.093450284	0.03689308
ZNF782	zinc finger protein 782	0.593201151	0.03710511
ZFP2	ZFP2 zinc finger protein	-0.863825125	0.03710511
TUBB4A	tubulin beta 4A class IVa	-1.266921621	0.03729697
ABHD1	abhydrolase domain containing 1	-1.096077411	0.03729697
BNIPL	BCL2 interacting protein like	-1.828838804	0.03733408
LOC1079861	uncharacterized LOC107986163	-0.739734827	0.03744506
LOC1053721	uncharacterized LOC105372165	-1.526467461	0.03763439

H2BC17	H2B clustered histone 17	1.759404175	0.03790757
UNC5A	unc-5 netrin receptor A	-1.184216512	0.03825983
CPA5	carboxypeptidase A5	1.67240132	0.03840916
RPL23AP21	ribosomal protein L23a pseudogene 21	-1.00560351	0.03851079
RN7SL832P	RNA; 7SL; cytoplasmic 832; pseudogene	-1.525892455	0.03858783
DGCR5	DiGeorge syndrome critical region gene 5	-1.498656015	0.03880668
FLG-AS1	FLG antisense RNA 1	-2.160564385	0.03901337
DKFZp451B(	uncharacterized LOC401282	-1.391043376	0.03908392
UCKL1-AS1	UCKL1 antisense RNA 1	-2.187653068	0.03916962
LINC00910	long intergenic non-protein coding RNA 910	0.659943327	0.03930544
CHD9NB	CHD9 neighbor	-0.635350843	0.03943775
EDRF1-DT	EDRF1 divergent transcript	-0.883865794	0.03988636
RIBC2	RIB43A domain with coiled-coils 2	-1.117905614	0.04001725
GALC	galactosylceramidase	-0.703287934	0.04115307
KALRN	kalirin RhoGEF kinase	-0.876191571	0.04151839
LINC02482	long intergenic non-protein coding RNA 2482	-0.797360193	0.0415189
PGF	placental growth factor	0.896142144	0.04158045
GPR157	G protein-coupled receptor 157	0.751807047	0.04180848
ADAMTSL3	ADAMTS like 3	0.601714911	0.04186945
MPP4	MAGUK p55 scaffold protein 4	-1.338781706	0.04209191
WDR86	WD repeat domain 86	-0.929432653	0.04220312
TPI1P2	triosephosphate isomerase 1 pseudogene 2	-0.890757263	0.04223281
FMO1	flavin containing dimethylaniline monoxygenase 1	-1.764595487	0.0422551
EIF2AK3-DT	EIF2AK3 divergent transcript	-1.06606834	0.04234783
CALML6	calmodulin like 6	-1.603911261	0.04235886
LINC00391	long intergenic non-protein coding RNA 391	-0.780555563	0.04239393
LOC1249045	uncharacterized LOC124904535	-1.416824505	0.04245049
PLCG2	phospholipase C gamma 2	0.768481391	0.04288753
LINC01239	long intergenic non-protein coding RNA 1239	2.806011306	0.04294923
FOXN3-AS1	FOXN3 antisense RNA 1	-0.825749669	0.0429784
IZUMO4	IZUMO family member 4	-0.762664656	0.04317759
VSTM2L	V-set and transmembrane domain containing 2 like	1.00973547	0.04322231
ZNF567	zinc finger protein 567	0.833862533	0.04380579
LAMA2	laminin subunit alpha 2	0.865786671	0.0440677
FBXL9P	F-box and leucine rich repeat protein; pseudogene	-0.636954984	0.0440677

HNRNPA1L2	heterogeneous nuclear ribonucleoprotein A1 like 2	-7.008261057	0.04414238
LOC1005062	uncharacterized LOC100506207	-1.229540946	0.04434436
LOC1027235	uncharacterized LOC102723566	-1.744996666	0.044377
UGDH-AS1	UGDH antisense RNA 1	-0.604169033	0.04438829
C8orf34-AS1	C8orf34 antisense RNA 1	-0.998862035	0.04446198
FSTL4	follistatin like 4	-1.759616663	0.04478526
PDZK1	PDZ domain containing 1	1.215055123	0.04492963
PTGS1	prostaglandin-endoperoxide synthase 1	-0.894598934	0.04493327
LOC1053723	uncharacterized LOC105372321	-1.972172968	0.0449932
MCIDAS	multiciliate differentiation and DNA synthesis associated cell cycle protein	0.786863324	0.04502038
LOC1053702	uncharacterized LOC105370259	-3.012022616	0.0450215
LOC1249022	uncharacterized LOC124902204	-1.006540724	0.04517753
NOTCH2NLR	notch 2 N-terminal like R	-0.703624276	0.04518035
TOLLIP-DT	TOLLIP divergent transcript	-0.839671299	0.04526928
CBLN3	cerebellin 3 precursor	-1.16785243	0.04583012
DNAAF11	dynein axonemal assembly factor 11	-0.62801488	0.04601061
UBE2R2-AS	UBE2R2 antisense RNA 1	-1.896191387	0.04642336
GHR	growth hormone receptor	0.697373861	0.04673038
C1RL-AS1	C1RL antisense RNA 1	1.135522698	0.04681828
NUDT17	nudix hydrolase 17	-0.611347689	0.04689392
GATA6	GATA binding protein 6	1.182927422	0.04696271
PCDHGA8	protocadherin gamma subfamily A; 8	-0.998304644	0.04697974
THSD4	thrombospondin type 1 domain containing 4	-0.706652075	0.04701285
NPAS4	neuronal PAS domain protein 4	-2.351802131	0.04720452
SRRM2-AS1	SRRM2 antisense RNA 1	-1.199433015	0.04735261
LRRC74B	leucine rich repeat containing 74B	-3.697760958	0.04742128
NIPSNAP3B	nipsnap homolog 3B	-1.25836577	0.04842093
DLEC1	DLEC1 cilia and flagella associated protein	-1.521470065	0.04857298
COLQ	collagen like tail subunit of asymmetric acetylcholinesterase	-1.926078293	0.04859991
SYNPR	synaptoporin	-1.137090055	0.04882236
REPS2	RALBP1 associated Eps domain containing 2	-1.778001142	0.04888589
LINC02133	long intergenic non-protein coding RNA 2133	-1.470097551	0.0489652
TTC39A	tetratricopeptide repeat domain 39A	1.100254954	0.04925592
KLHL35	kelch like family member 35	0.743238483	0.04937946
LINC01410	long intergenic non-protein coding RNA 1410	-1.095962386	0.04943305
MAGEH1	MAGE family member H1	-0.959394368	0.04965292
LOC644656	uncharacterized LOC644656	-0.967470332	0.04969326

PCDHGA7	protocadherin gamma subfamily A; 7	-1.299071293	0.04969815
ZNF41	zinc finger protein 41	-0.771585297	0.04975371
LPAR2	lysophosphatidic acid receptor 2	-0.810121877	0.04985004
VTCN1	V-set domain containing T cell activation inhibitor 1	-1.204823464	0.04999791
LOC1053724	uncharacterized LOC105372421	-2.449515615	0.05003063
C1orf21	chromosome 1 open reading frame 21	-0.618252079	0.05005851
COL28A1	collagen type XXVIII alpha 1 chain	-1.581220353	0.05007567
SNHG21	small nucleolar RNA host gene 21	-0.688574057	0.05016084
LRRC8C-DT	LRRC8C divergent transcript	-1.025032086	0.05046414
FGF19	fibroblast growth factor 19	-1.401074166	0.05058343
PCBP3	poly(rC) binding protein 3	2.075489159	0.05058979
H2BC20P	H2B clustered histone 20; pseudogene	1.355118	0.0506964
CFP	complement factor properdin	1.351023146	0.05077152
ACOT11	acyl-CoA thioesterase 11	-1.920502955	0.0512978
HCN2	hyperpolarization activated cyclic nucleotide gated potassium and sodium channel 2	-0.629495243	0.05153215
VWF	von Willebrand factor	-1.372530278	0.05165298
N4BP2L2-IT	N4BPL2 intronic transcript 2	-1.057383157	0.05165983
SOD2-OT1	SOD2 overlapping transcript 1	-1.399791996	0.05209699
CABCOCO1	ciliary associated calcium binding coiled-coil 1	-1.397272298	0.05228476
TRIM6	tripartite motif containing 6	1.548825977	0.05276826
RASSF5	Ras association domain family member 5	1.331932271	0.05278858
MIF4GD-DT	MIF4GD divergent transcript	-0.684971539	0.05279827
LOC1009966	uncharacterized LOC100996660	-1.27225009	0.05287198
ZNF594-DT	ZNF594 divergent transcript	-0.671875493	0.05288408
ADNP-AS1	ADNP antisense RNA 1	-0.911461313	0.05311943
ERICH6-AS1	ERICH6 antisense RNA 1	-1.384547477	0.05314692
SYT6	synaptotagmin 6	-1.678015561	0.05331328
SATB1	SATB homeobox 1	-0.882564743	0.05331328
SMIM10L2B	small integral membrane protein 10 like 2B	-1.329322658	0.05343996
CRACR2B	calcium release activated channel regulator 2B	-1.282994143	0.05344805
FBXO15	F-box protein 15	-1.304302544	0.05384893
PCDHAC2	protocadherin alpha subfamily C; 2	-0.614155288	0.05433038
TBX2-AS1	TBX2 antisense RNA 1	-0.928510969	0.05452454
ZDHHC12-D	ZDHHC12 divergent transcript	-0.841312786	0.05459131
LOC1019294	uncharacterized LOC101929427	-1.897581134	0.05467514
CCDC15-DT	CCDC15 divergent transcript	-0.781447943	0.05482759
UPK3B	uroplakin 3B	-1.435327853	0.05499228
TXNDC2	thioredoxin domain containing 2	1.537195795	0.05508306

PNCK	pregnancy up-regulated nonubiquitous CaM kinase	-0.601634528	0.05525946
CFAP276	cilia and flagella associated protein 276	-1.937336859	0.05553246
ZFR2	zinc finger RNA binding protein 2	0.770329507	0.05583173
HSD17B8	hydroxysteroid 17-beta dehydrogenase 8	-1.580424005	0.05593146
MORN3	MORN repeat containing 3	-0.719335797	0.05612759
MESP1	mesoderm posterior bHLH transcription factor 1	0.644495548	0.05626829
C2orf88	chromosome 2 open reading frame 88	0.887169178	0.05638897
ZNF230-DT	ZNF230 divergent transcript	-1.668377285	0.05644279
CCDC180	coiled-coil domain containing 180	-1.675322023	0.05646224
CDK15	cyclin dependent kinase 15	2.103204423	0.05661505
ATP13A4	ATPase 13A4	-2.381600615	0.05666856
HORMAD2	HORMA domain containing 2	-2.092605027	0.05694567
LURAP1	leucine rich adaptor protein 1	-0.696599746	0.056957
LMO1	LIM domain only 1	-1.398605773	0.05704473
CRYGS	crystallin gamma S	-0.916259698	0.0570935
MYLK3	myosin light chain kinase 3	-1.779846328	0.05714496
ADAMTS12	ADAM metallopeptidase with thrombospondin type 1 motif 12	-0.793246839	0.05716675
TBC1D26	TBC1 domain family member 26	-2.024715858	0.05771527
RPS2P14	ribosomal protein S2 pseudogene 14	-1.079455859	0.05776305
OPCML	opioid binding protein/cell adhesion molecule like	-2.150813019	0.05806477
ABCG4	ATP binding cassette subfamily G member 4	-1.759366259	0.05843766
CCDC88B	coiled-coil domain containing 88B	-0.653294243	0.05854768
ACTRT3	actin related protein T3	0.990111727	0.05917207
STXBP6	syntaxin binding protein 6	-1.018981645	0.05928767
ANKRD44	ankyrin repeat domain 44	-0.81023591	0.05941352
KLF14	KLF transcription factor 14	1.314327476	0.05948532
TINCR	TINCR ubiquitin domain containing	-0.976309366	0.05950715
GLYATL2	glycine-N-acyltransferase like 2	0.935968757	0.05962856
SH3TC1	SH3 domain and tetratricopeptide repeats 1	-1.781053577	0.05987706
EEF1A1P19	eukaryotic translation elongation factor 1 alpha 1 pseudogene 19	-1.264709283	0.05987706
FRAT1	FRAT regulator of WNT signaling pathway 1	-0.842964552	0.05995441
RGR	retinal G protein coupled receptor	-1.037028952	0.06001953
GDA	guanine deaminase	1.277975129	0.06043329
MAB21L2	mab-21 like 2	1.913069537	0.06047423
NLRP1	NLR family pyrin domain containing 1	2.23567013	0.06047423
ISY1-RAB43	ISY1-RAB43 readthrough	0.880574894	0.06068018
LOC1079849	uncharacterized LOC107984948	-0.986262874	0.06078731

COMMD3-BI	COMMD3-BMI1 readthrough	-3.055402893	0.06088928
LRGUK	leucine rich repeats and guanylate kinase domain containing	-0.679913272	0.06176081
ARHGEF16	Rho guanine nucleotide exchange factor 16	1.15544624	0.06176081
ZNF559-ZNF	ZNF559-ZNF177 readthrough	1.324748201	0.06200452
CCDC102B	coiled-coil domain containing 102B	-0.689129127	0.06210439
MITF	melanocyte inducing transcription factor	-0.729029029	0.06254437
TRIM7	tripartite motif containing 7	0.845755698	0.06288342
LINC01460	long intergenic non-protein coding RNA 1460	1.664961367	0.06311088
CYP27B1	cytochrome P450 family 27 subfamily B member 1	0.823266629	0.06314072
SPON1	spondin 1	-0.900214784	0.06320748
RASL11A	RAS like family 11 member A	0.816156955	0.06350225
GSTO2	glutathione S-transferase omega 2	-0.878572492	0.06350225
GOLGA8R	golgin A8 family member R	-2.428029182	0.06350225
KISS1R	KISS1 receptor	1.512717987	0.06360871
ZNF19	zinc finger protein 19	-0.758956627	0.06373802
RIMBP2	RIMS binding protein 2	1.173145659	0.06384369
YPEL3-DT	YPEL3 divergent transcript	-0.967562698	0.06456159
PCDH17	protocadherin 17	0.666995535	0.06467467
NPAS2	neuronal PAS domain protein 2	1.273054272	0.06468141
YTHDF3-DT	YTHDF3 divergent transcript	-0.901895286	0.06476439
ELOA-AS1	ELOA antisense RNA 1	-0.669966578	0.06517314
CLMN	calmin	0.598499045	0.06522103
KIRREL3	kirre like nephrin family adhesion molecule 3	-0.681441021	0.06589338
CYP24A1	cytochrome P450 family 24 subfamily A member 1	2.468200748	0.06608987
SIGIRR	single Ig and TIR domain containing	1.069184075	0.06617302
RPRM	reprimo; TP53 dependent G2 arrest mediator homolog	-2.684284816	0.06644222
CBX3P2	CBX3 pseudogene 2	-1.464812285	0.06662911
CFAP96	cilia and flagella associated protein 96	0.684974678	0.06698884
TNKS2-DT	TNKS2 divergent transcript	-0.995617577	0.06710317
VAMP8	vesicle associated membrane protein 8	-1.523754165	0.06748842
CCDC150	coiled-coil domain containing 150	-0.597572355	0.06762884
EFHB	EF-hand domain family member B	-1.618667608	0.06778225
LOC1009964	uncharacterized LOC100996437	-1.094998007	0.06822573
CAPN10-DT	CAPN10 divergent transcript	-0.594588433	0.0685166
TPD52L1	TPD52 like 1	-0.848283731	0.06889756
CHRM4	cholinergic receptor muscarinic 4	-4.016671435	0.06898929
DRP2	dystrophin related protein 2	-0.858598822	0.0689964
TFPI	tissue factor pathway inhibitor	-0.796473809	0.06919073

NFAM1	NFAT activating protein with ITAM motif 1	0.743126019	0.06948514
CABP7	calcium binding protein 7	-1.011538918	0.06951008
ZNF22-AS1	ZNF22 antisense RNA 1	-0.614827483	0.06965615
ZNF135	zinc finger protein 135	-0.879004387	0.07034692
ACTA1	actin alpha 1; skeletal muscle	-1.639000692	0.07048367
ROBO2	roundabout guidance receptor 2	-0.691822236	0.07085257
SLC32A1	solute carrier family 32 member 1	-0.675754063	0.07090271
PCDHB15	protocadherin beta 15	-0.751868592	0.07098953
FOXO4	forkhead box O4	-0.625746387	0.07118127
PCK2	phosphoenolpyruvate carboxykinase 2; mitochondrial	0.64153266	0.07134088
ATG9B	autophagy related 9B	0.779640605	0.07144684
CARD8-AS1	CARD8 antisense RNA 1	0.843113692	0.07190398
SUGT1P4-S	SUGT1P4-STRA6LP-CCDC180 readthrough	-1.205218827	0.07206131
PELATON	plaque enriched lncRNA in atherosclerotic and inflammatory bowel macrophage regulation	2.00753038	0.07209235
TAS2R20	taste 2 receptor member 20	-0.988330839	0.07222207
ELAPOR1	endosome-lysosome associated apoptosis and autophagy regulator 1	-0.97320247	0.07234786
CDH22	cadherin 22	1.630893183	0.07265504
CHD5	chromodomain helicase DNA binding protein 5	-1.173509462	0.07270282
ALDH1L2	aldehyde dehydrogenase 1 family member L2	-0.874921228	0.07271192
TRIM2	tripartite motif containing 2	-0.935060193	0.07286711
LY6G5C	lymphocyte antigen 6 family member G5C	0.708480407	0.07290455
SYTL4	synaptotagmin like 4	-1.001510373	0.07292748
ODAD2	outer dynein arm docking complex subunit 2	-1.084864848	0.07293469
TTC21A	tetratricopeptide repeat domain 21A	-0.755686294	0.07320373
LINC03016	long intergenic non-protein coding RNA 3016	-0.721647709	0.07421725
C2	complement C2	1.257978044	0.07427791
KBTBD6-DT	KBTBD6 divergent transcript	-1.420094139	0.07428056
COL5A2	collagen type V alpha 2 chain	-0.618048056	0.07437329
FNDC5	fibronectin type III domain containing 5	-1.092229753	0.07450841
FREM1	FRAS1 related extracellular matrix 1	1.820151818	0.07454425
CLEC12A-AS	CLEC12A antisense RNA 1	-1.402069854	0.07454425
LINC01778	long intergenic non-protein coding RNA 1778	-0.672634436	0.07462392
HTATIP2	HIV-1 Tat interactive protein 2	1.651280418	0.07487893

TESK2	testis associated actin remodelling kinase 2	1.301710172	0.0761123
ERO1B	endoplasmic reticulum oxidoreductase 1 beta	0.665116133	0.07698184
IDH1-AS1	IDH1 antisense RNA 1	-1.804161323	0.07731289
ADAMTS2	ADAM metallopeptidase with thrombospondin type 1 motif 2	1.879040447	0.07731289
SCART1	scavenger receptor family member expressed on T cells 1	-0.808655585	0.0779163
PILRA	paired immunoglobin like type 2 receptor alpha	-1.091805179	0.07822443
LAMA3	laminin subunit alpha 3	1.02523024	0.07822759
C3orf70	chromosome 3 open reading frame 70	-0.940131063	0.0783059
FLJ16779	uncharacterized LOC100192386	-0.903408563	0.0785188
MSX1	msh homeobox 1	1.683943959	0.07853106
HDAC4-AS1	HDAC4 antisense RNA 1	-1.669422451	0.07904548
LINC00548	long intergenic non-protein coding RNA 548	-1.683230346	0.07921751
C6orf163	chromosome 6 open reading frame 163	-1.30786536	0.07941509
NKX6-2	NK6 homeobox 2	0.76298087	0.07967943
LOC1122678	uncharacterized LOC112267871	-0.650005494	0.07987643
LPIN2	lipin 2	0.713094801	0.08031579
DENND1B	DENN domain containing 1B	-0.728459526	0.08058891
NXPH3	neurexophilin 3	-2.177300269	0.08094226
RAG1	recombination activating 1	-1.59452745	0.08116157
IL10RA	interleukin 10 receptor subunit alpha	1.428206315	0.08160658
PCDHGA12	protocadherin gamma subfamily A; 12	-0.894544511	0.08177144
RNF157-AS1	RNF157 antisense RNA 1	-0.625481139	0.08179613
TENM2-AS1	TENM2 antisense RNA 2	-1.129232041	0.08239793
ADGRL3	adhesion G protein-coupled receptor L3	0.86573876	0.0825746
LRRK1	leucine rich repeat kinase 1	0.714186149	0.0825746
WIPF3	WAS/WASL interacting protein family member 3	-0.732951952	0.08336199
PSORS1C1	psoriasis susceptibility 1 candidate 1	1.104611422	0.08342016
SMPD3	sphingomyelin phosphodiesterase 3	0.986823275	0.08353413
FPGT-TNNI3	FPGT-TNNI3K readthrough	-1.262619424	0.08425186
MGARP	mitochondria localized glutamic acid rich protein	-0.731116618	0.08511948
DMTF1-AS1	DMTF1 antisense RNA 1	-1.487504458	0.08529639
CORT	cortistatin	-1.798277187	0.08531368
RBFADN	RBFA downstream neighbor	-0.672767613	0.08537214
FAM167A	family with sequence similarity 167 member A	1.002446326	0.08537214
DDX11-AS1	DDX11 antisense RNA 1	-1.428311222	0.08541967
KLHDC1	kelch domain containing 1	-1.105187037	0.08605897
PCDHGA2	protocadherin gamma subfamily A; 2	-0.595898969	0.08620263

MTCO1P42	MT-CO1 pseudogene 42	-0.667874725	0.08631914
PRR18	proline rich 18	-0.60201598	0.08640184
MRAP2	melanocortin 2 receptor accessory protein 2	-1.507835617	0.08648181
HDAC2-AS2	HDAC2 and HS3ST5 antisense RNA 2	-0.948127476	0.08733371
LOC1019288	uncharacterized LOC101928847	-0.950051987	0.08766618
SCN3A	sodium voltage-gated channel alpha subunit 3	-0.844982295	0.08771051
RPL6P27	ribosomal protein L6 pseudogene 27	-1.254014806	0.08820557
RIMBP3B	RIMS binding protein 3B	1.024830203	0.08842211
IGSF3P2	IGSF3 pseudogene 2	-1.000504094	0.08916764
TMEM132E-	TMEM132E divergent transcript	-2.419839055	0.08920643
LINC01331	long intergenic non-protein coding RNA 1331	-1.322522181	0.090581
TVP23C-CD	TVP23C-CDRT4 readthrough	0.607599801	0.09115191
ARRDC3-AS	ARRDC3 antisense RNA 1	-0.782907858	0.09122123
C2orf50	chromosome 2 open reading frame 50	0.884357457	0.0912424
GCK	glucokinase	-0.960938849	0.0912652
NUPR1	nuclear protein 1; transcriptional regulator	0.611767646	0.09135657
U2AF1	U2 small nuclear RNA auxiliary factor 1	1.240365018	0.09152044
ACE2	angiotensin converting enzyme 2	3.258450525	0.09154148
FAM106A	family with sequence similarity 106 member A	-1.859337343	0.0915633
LINC01783	long intergenic non-protein coding RNA 1783	-0.890991621	0.09202043
LINC01144	long intergenic non-protein coding RNA 1144	-1.037109211	0.09221405
CSMD1	CUB and Sushi multiple domains 1	-0.815549348	0.09276228
GUCY1B2	guanylate cyclase 1 soluble subunit beta 2 (pseudogene)	-1.397557428	0.09328136
LINC02249	long intergenic non-protein coding RNA 2249	-1.163760697	0.0933943
AOC3	amine oxidase copper containing 3	1.264992597	0.09345556
GEMIN7-AS	GEMIN7 antisense RNA 1	-0.684860081	0.09347807
GRIP2	glutamate receptor interacting protein 2	1.170753561	0.09375705
ANO9	anoctamin 9	1.242215397	0.09389852
IFNE	interferon epsilon	-1.336370379	0.09464691
CFAP298-TC	CFAP298-TCP10L readthrough	1.456197645	0.09493313
KLF2	KLF transcription factor 2	-0.932087611	0.09511342
GRM8	glutamate metabotropic receptor 8	1.003706802	0.09520077
NEAT1	nuclear paraspeckle assembly transcript 1	-0.717166412	0.09544087
FRAS1	Fraser extracellular matrix complex subunit 1	-1.74090228	0.09595475
TMEM220	transmembrane protein 220	-0.767196569	0.0960792

NR1H4	nuclear receptor subfamily 1 group H member 4	1.36412995	0.09670821
LOC105374(	uncharacterized LOC105374085	-1.173354116	0.09724174
CAPN9	calpain 9	-0.941994377	0.09735362
NKPD1	NTPase KAP family P-loop domain containing 1	-1.096291247	0.09766444
SLC13A3	solute carrier family 13 member 3	0.770101435	0.0979716
ACOXL-AS1	ACOXL antisense RNA 1	-1.925903849	0.09803856
MYBPC2	myosin binding protein C2	0.872390368	0.09832113
EFCAB5	EF-hand calcium binding domain 5	1.377630439	0.09840216
PODNL1	podocan like 1	-1.296197697	0.09877855
CD180	CD180 molecule	0.683197007	0.09877855
FTH1P10	ferritin heavy chain 1 pseudogene 10	1.702547	0.09882638
CCDC183	coiled-coil domain containing 183	-1.149743736	0.09894517
ZNF763	zinc finger protein 763	-0.907462876	0.09900668
PDZD7	PDZ domain containing 7	-0.929243495	0.09902935
CHST6	carbohydrate sulfotransferase 6	-0.906432443	0.09907861
DRC7	dynein regulatory complex subunit 7	-1.058880003	0.09948263
ZNF488	zinc finger protein 488	-1.5565606	0.09983048

Supplementary Table 2: DEGs in EPEA+IL1β vs IL1β conditions (log2FoldChange ≤ -0.59 or ≥ +0.59, adjusted p-value< 0.1)

Gene symbol	Gene name	log2FoldChange	padj
UTP14C	UTP14C small subunit processome component	-11.59355615	0.0981839
FBN1	fibrillin 1	0.597081917	0.0003662
CLASP2	cytoplasmic linker associated protein 2	0.627125077	0.033083
HYCC2	hyccin PI4KA lipid kinase complex subunit 2	0.721787256	0.0319454
AHDC1	AT-hook DNA binding motif containing 1	0.722147872	0.0704241
NMNAT2	nicotinamide nucleotide adenylyltransferase 2	0.804210039	0.0436336
FRY	FRY microtubule binding protein	1.293924374	0.0492284
PECAM1	platelet and endothelial cell adhesion molecule 1	2.028216974	0.0389498
GOLGA8N	golgin A8 family member N	2.475198195	0.0171939
C1QTNF3-AMACR	C1QTNF3-AMACR readthrough (NMD candidate)	4.449642921	0.0224984

Supplementary Table 3: DEGs in DHEA+IL1β vs IL1β conditions (log2FoldChange ≤ -0.59 or ≥ +0.59, adjusted p-value< 0.1)

Gene symbol	Gene name	log2FoldChange	padj
TFPI	tissue factor pathway inhibitor	-2.398043068	0.03515
ADAMTS12	ADAM metallopeptidase with thrombospondin type 1 motif 12	-2.078570987	0.04334
RANBP3L	RAN binding protein 3 like	-2.058438324	0.09013
DOCK10	dedicator of cytokinesis 10	-1.633977681	0.07624
MMS22L	MMS22 like; DNA repair protein	-0.961953249	0.05411
DNAH1	dynein axonemal heavy chain 1	-0.907839274	0.07624
H2BC19P	H2B clustered histone 19; pseudogene	0.885949465	0.09974
IQCH-AS1	IQCH antisense RNA 1	1.407405134	0.01553
GRIN1	glutamate ionotropic receptor NMDA type subunit 1	1.671293285	0.00015

Supplementary Table 4: DEGs in EPEA vs Control conditions (log2FoldChange ≤ -0.59 or ≥ +0.59, adjusted p-value< 0.1)

Gene symbol	Gene name	log2FoldChange	padj
IFIT2	interferon induced protein with tetratricopeptide repeats 2	20.44453764	0.0000749
SPECC1L-ADORA2	SPECC1L-ADORA2A readthrough (NMD candidate)	19.63919414	0.00016646
ZIC3	Zic family member 3	4.699029236	0.0154798
MGAT4C	MGAT4 family member C	3.078381815	0.00012673
HOXB1	homeobox B1	2.986165009	0.02533325
MASP1	MBL associated serine protease 1	2.83931948	0.01886872
CPLX2	complexin 2	2.579203113	0.00012125
HEY1	hes related family bHLH transcription factor with YRPW motif 1	2.535170777	0.02155644
KIAA1755	KIAA1755	2.534834463	0.09959495
MYT1	myelin transcription factor 1	2.417711444	0.0030136
COL25A1	collagen type XXV alpha 1 chain	2.333206107	0.01451602
NPL	N-acetylneuraminate pyruvate lyase	2.22561571	0.00010581
GJB2	gap junction protein beta 2	2.184584485	0.05722289
NTNG2	netrin G2	2.160357347	0.01115007
TPTEP2-CSNK1E	TPTEP2-CSNK1E readthrough	2.072477483	0.01546231
NKILA	NF-kappaB interacting lncRNA	1.948065151	0.04179699
NRXN3	neurexin 3	1.828034183	0.00485269
KLHL4	kelch like family member 4	1.789417782	0.04709026
RUNDC3A	RUN domain containing 3A	1.775441951	0.02204242
NTRK2	neurotrophic receptor tyrosine kinase 2	1.769059532	0.00000164
RGS8	regulator of G protein signaling 8	1.757255016	3.13E-08
MMP7	matrix metallopeptidase 7	1.718596252	0.00631939
SERPINA3	serpin family A member 3	1.676043494	0.08448903
DNM3	dynamin 3	1.599156079	0.0043077
DENND1C	DENN domain containing 1C	1.585071494	0.035072
VAV3	vav guanine nucleotide exchange factor 3	1.563296395	0.01649648
RGR	retinal G protein coupled receptor	1.509978573	0.02771826

DIPK1C	divergent protein kinase domain 1C	1.492839778	0.0000397
WNT11	Wnt family member 11	1.480730467	0.01441468
LIPT2	lipoyl(octanoyl) transferase 2	1.407567018	0.08904057
RPS26P19	ribosomal protein S26 pseudogene 19	1.378134571	0.09380924
PKP1	plakophilin 1	1.295921404	0.0000888
SERPINF1	serpin family F member 1	1.287991258	1.63E-08
PCDH1	protocadherin 1	1.276316198	0.00043574
NDRG2	NDRG family member 2	1.264498694	0.00022219
TMEM45A	transmembrane protein 45A	1.250369037	0.08709484
SORCS2	sortilin related VPS10 domain containing receptor 2	1.236481442	0.01894491
DLGAP1	DLG associated protein 1	1.231103776	0.00211377
SPRN	shadow of prion protein	1.221859935	0.00507205
CRMP1	collapsin response mediator protein 1	1.189961461	9.08E-10
ANGPTL1	angiopoietin like 1	1.178615477	3.04E-07
ENTREP2	endosomal transmembrane epsin interactor 2	1.178585177	0.00535985
ARID3A	AT-rich interaction domain 3A	1.155804584	0.00905398
TOX3	TOX high mobility group box family member 3	1.123958632	0.0000363
CACNG7	calcium voltage-gated channel auxiliary subunit gamma 7	1.100481821	1.87E-10
ROR1	receptor tyrosine kinase like orphan receptor 1	1.099230819	0.0018122
RNF180	ring finger protein 180	1.094031677	0.02643163
BCAN	brevican	1.090000564	0.00323686
PMEPA1	prostate transmembrane protein; androgen induced 1	1.066230882	4.16E-08
COL14A1	collagen type XIV alpha 1 chain	1.047242561	0.00025909
RGS16	regulator of G protein signaling 16	1.041716245	0.00000463
TRIM2	tripartite motif containing 2	1.024432351	0.02338474
LRRC3	leucine rich repeat containing 3	0.96968752	0.05265211
DLX5	distal-less homeobox 5	0.965545125	0.00229707
HES2	hes family bHLH transcription factor 2	0.951096263	0.08904057

ASPHD2	aspartate beta-hydroxylase domain containing 2	0.943298587	0.03977171
NFE2L3	NFE2 like bZIP transcription factor 3	0.943106943	0.00059973
SCD5	stearoyl-CoA desaturase 5	0.928232307	6.1E-11
TMEM178B	transmembrane protein 178B	0.926524313	0.01062687
ZNF175	zinc finger protein 175	0.907803345	0.0026991
ZSCAN31	zinc finger and SCAN domain containing 31	0.903322868	0.02338474
GIN1	gypsy retrotransposon integrase 1	0.88404275	0.03555204
SPOCK1	SPARC (osteonectin); cwcv and kazal like domains proteoglycan 1	0.881845602	0.01689855
IGFBP5	insulin like growth factor binding protein 5	0.86645796	0.00000558
ANK3	ankyrin 3	0.862272791	0.0020567
PCDH19	protocadherin 19	0.860653297	0.00014998
ST18	ST18 C2H2C-type zinc finger transcription factor	0.860390975	0.07109771
PLAAT1	phospholipase A and acyltransferase 1	0.85761331	0.08083857
HBA1	hemoglobin subunit alpha 1	0.854789142	0.00107028
ELAVL3	ELAV like RNA binding protein 3	0.845821492	0.01452082
SULF2	sulfatase 2	0.84575138	0.0003288
SSPN	sarcospan	0.819330843	0.06084374
HOXB3	homeobox B3	0.812871652	0.00943074
INSM1	INSM transcriptional repressor 1	0.806982254	0.00312548
APCDD1	APC down-regulated 1	0.800602817	0.03324633
PPP1R13B	protein phosphatase 1 regulatory subunit 13B	0.784032756	0.09881266
KREMEN2	kringle containing transmembrane protein 2	0.7791889	0.07840668
PRDM1	PR/SET domain 1	0.775127187	0.04299478
HDC	histidine decarboxylase	0.763864474	0.06414739
GPR155	G protein-coupled receptor 155	0.749364914	0.00528139
PAK3	p21 (RAC1) activated kinase 3	0.742715637	0.00069032
PCDHGA2	protocadherin gamma subfamily A; 2	0.737711671	0.06203373
CXXC4	CXXC finger protein 4	0.736448553	0.05713594
NEGR1	neuronal growth regulator 1	0.736308942	0.05374476

CD24	CD24 molecule	0.72231861	0.00057634
ESPN	espin	0.720819301	0.03358834
KIAA0513	KIAA0513	0.710707698	0.01514471
GNG2	G protein subunit gamma 2	0.703980211	0.0000698
SHROOM2	shroom family member 2	0.700767699	0.0343788
JAKMIP2-AS1	JAKMIP2 antisense RNA 1	0.69999766	0.0000264
KCNN4	potassium calcium-activated channel subfamily N member 4	0.693466666	0.05988287
CCDC82	coiled-coil domain containing 82	0.686766596	0.00836422
TNFRSF19	TNF receptor superfamily member 19	0.684283915	0.000426
SFMBT2	Scm like with four mbt domains 2	0.681489111	0.06911751
FILIP1L	filamin A interacting protein 1 like	0.678646466	0.07046733
DAB1	DAB adaptor protein 1	0.67750711	7.07E-07
ND3	NADH dehydrogenase subunit 3	0.676582485	0.0000115
VCAM1	vascular cell adhesion molecule 1	0.673034304	0.03166932
ST8SIA5	ST8 alpha-N-acetyl-neuraminide alpha-2;8-sialyltransferase 5	0.663447923	0.03289735
MFAP4	microfibril associated protein 4	0.644685468	0.06380523
RASSF4	Ras association domain family member 4	0.643650516	0.00347467
DMRTA2	DMRT like family A2	0.641335998	0.06841927
PHYHIPL	phytanoyl-CoA 2-hydroxylase interacting protein like	0.630467976	0.01052303
GNG4	G protein subunit gamma 4	0.620615886	3.56E-08
VWA1	von Willebrand factor A domain containing 1	0.61606247	0.01170874
AUTS2	activator of transcription and developmental regulator AUTS2	0.611292949	0.08083857
ACTG2	actin gamma 2; smooth muscle	0.606514749	0.0202614
GZF1	GDNF inducible zinc finger protein 1	0.605003905	0.03741722
NOG	noggin	0.604248223	0.04705526
LRATD1	LRAT domain containing 1	0.60295125	0.04075125
ACVR2A	activin A receptor type 2A	0.599617405	0.02543504

PXDN	peroxidasin	0.593869674	0.00104401
ARHGAP42	Rho GTPase activating protein 42	0.592222526	0.02584853
LRRC4	leucine rich repeat containing 4	-0.591708382	0.00671852
ADAMTS9	ADAM metallopeptidase with thrombospondin type 1 motif 9	-0.595016808	0.01894491
ANK1	ankyrin 1	-0.595406379	0.00022219
PDPN	podoplanin	-0.597065681	5.97E-07
PLXND1	plexin D1	-0.598131269	0.0000882
DTX4	deltex E3 ubiquitin ligase 4	-0.601175007	0.08687547
NQO1	NAD(P)H quinone dehydrogenase 1	-0.602788701	0.00321832
FAM124A	family with sequence similarity 124 member A	-0.603531165	0.00325613
SRGAP1	SLIT-ROBO Rho GTPase activating protein 1	-0.604077054	0.00057634
SEZ6	seizure related 6 homolog	-0.605336967	0.02218888
TENT5B	terminal nucleotidyltransferase 5B	-0.607735804	0.0000178
MBOAT1	membrane bound O-acyltransferase domain containing 1	-0.6108876	0.07840668
HLA-DRB5	major histocompatibility complex; class II; DR beta 5	-0.611739218	0.00803979
HEG1	heart development protein with EGF like domains 1	-0.613587514	0.0020807
HLA-DMB	major histocompatibility complex; class II; DM beta	-0.614460023	0.03555204
ZNF69	zinc finger protein 69	-0.61721966	0.03350201
SDC4	syndecan 4	-0.617246063	0.0000933
KMT2D	lysine methyltransferase 2D	-0.619821902	0.01194506
PGM5	phosphoglucomutase 5	-0.623653208	0.0254716
AXL	AXL receptor tyrosine kinase	-0.627081968	0.00000128
NBPF19	NBPF member 19	-0.628824246	0.01052303
PLEKHG4B	pleckstrin homology and RhoGEF domain containing G4B	-0.631770825	7.17E-08
ITPR3	inositol 1;4;5-trisphosphate receptor type 3	-0.642240396	0.05369311
UBR4	ubiquitin protein ligase E3 component n-recognin 4	-0.642518214	0.01346103
PURPL	p53 upregulated regulator of p53 levels	-0.651746225	0.03361283

MDN1	midasin AAA ATPase 1	-0.655544952	0.00162147
F3	coagulation factor III; tissue factor	-0.65992619	1.77E-14
HERC2	HECT and RLD domain containing E3 ubiquitin protein ligase 2	-0.661477155	0.03707834
NBEA	neurobeachin	-0.6635646	0.0018122
STRIP2	striatin interacting protein 2	-0.664932467	0.02675885
PDE3A	phosphodiesterase 3A	-0.665239954	0.05089497
SLC8A1	solute carrier family 8 member A1	-0.669509556	0.01659525
CBR3	carbonyl reductase 3	-0.671946891	0.07046733
ZNF483	zinc finger protein 483	-0.674374392	0.04480878
LY6H	lymphocyte antigen 6 family member H	-0.676142413	0.07046733
TEK	TEK receptor tyrosine kinase	-0.676824529	0.018704
OPLAH	5-oxoprolinase; ATP-hydrolysing	-0.677275506	0.0661216
GABRQ	gamma-aminobutyric acid type A receptor subunit theta	-0.680569643	1.13E-08
LRRC37B	leucine rich repeat containing 37B	-0.68161407	0.03249212
SNAI2	snail family transcriptional repressor 2	-0.682870155	0.00039863
JMJD7-PLA2G4B	JMJD7-PLA2G4B readthrough	-0.691378912	0.00431691
HECTD4	HECT domain E3 ubiquitin protein ligase 4	-0.69485451	0.04821878
ALPL	alkaline phosphatase; biomineralization associated	-0.695981872	0.02133953
FTX	FTX transcript; XIST regulator	-0.696271753	0.01018825
CELSR1	cadherin EGF LAG seven-pass G-type receptor 1	-0.696692112	0.02585563
FBN2	fibrillin 2	-0.700565762	1.64E-07
ITGA5	integrin subunit alpha 5	-0.701395632	0.00144549
SLC1A2	solute carrier family 1 member 2	-0.706038057	1.86E-11
ZIC4	Zic family member 4	-0.706092445	0.02626934
ICAM1	intercellular adhesion molecule 1	-0.707845248	0.01908543
STK17B	serine/threonine kinase 17b	-0.708718497	0.05304338

B3GALT1	beta-1;3-galactosyltransferase 1	-0.708779769	0.0000363
NBPF14	NBPF member 14	-0.711573898	0.00231035
NAV2	neuron navigator 2	-0.713660627	0.00011455
MYO18A	myosin XVIIIA	-0.714274252	2.85E-09
ARMCX1	armadillo repeat containing X-linked 1	-0.714808564	0.08806995
NEK7	NIMA related kinase 7	-0.717382601	0.04299478
ATP1A2	ATPase Na+/K+ transporting subunit alpha 2	-0.717912298	0.02675885
FOXJ1	forkhead box J1	-0.723734013	3.19E-08
PHF11	PHD finger protein 11	-0.725321314	0.03855706
SMG1P3	SMG1 pseudogene 3	-0.729747755	0.00108998
DOK6	docking protein 6	-0.731758579	0.03726196
LMO7	LIM domain 7	-0.734244006	0.00252453
TRIM66	tripartite motif containing 66	-0.736982385	0.03703447
TTLL3	tubulin tyrosine ligase like 3	-0.736992371	0.00584811
RFX4	regulatory factor X4	-0.742081357	0.00012278
PDE8B	phosphodiesterase 8B	-0.744174437	0.02737
SH3BP5-AS1	SH3BP5 antisense RNA 1	-0.749921095	0.08083857
KANTR	KANTR integral membrane protein	-0.751332362	0.00595689
NPIPB3	nuclear pore complex interacting protein family member B3	-0.75253398	0.01750299
LRRC15	leucine rich repeat containing 15	-0.752550723	0.02759331
B3GALT5	beta-1;3-galactosyltransferase 5	-0.754318896	0.01755754
RALYL	RALY RNA binding protein like	-0.755153291	0.05084678
STX1B	syntaxin 1B	-0.757102678	0.09021322
SORL1	sortilin related receptor 1	-0.760128538	6.77E-09
ZBTB38	zinc finger and BTB domain containing 38	-0.765630032	0.00085046
ENPP2	ectonucleotide pyrophosphatase/phosphodiesterase 2	-0.766981895	0.00761113
C6orf118	chromosome 6 open reading frame 118	-0.767536834	0.00993898
LOXL2	lysyl oxidase like 2	-0.772836639	0.00012466
FKBP11	FKBP prolyl isomerase 11	-0.773032961	0.07711428
CYP1B1	cytochrome P450 family 1 subfamily B member 1	-0.77580727	0.00242909
NUP50-DT	NUP50 divergent transcript	-0.778625146	0.06303954
ITGB3	integrin subunit beta 3	-0.779706368	0.0000123

KIAA1614	KIAA1614	-0.786275737	0.05123122
OBSCN	obscurin; cytoskeletal calmodulin and titin-interacting RhoGEF	-0.796129212	0.02010006
NFATC4	nuclear factor of activated T cells 4	-0.802782304	0.07114679
UNC80	unc-80 homolog; NALCN channel complex subunit	-0.805606489	0.05073386
A2M	alpha-2-macroglobulin	-0.808447963	0.00264004
DNHD1	dynein heavy chain domain 1	-0.814826196	0.00119113
SH3BP1	SH3 domain binding protein 1	-0.817298638	0.04693106
SYNCRIP	synaptotagmin binding cytoplasmic RNA interacting protein	-0.828082487	0.00181679
VPS13D	vacuolar protein sorting 13 homolog D	-0.83142623	8.99E-07
FBN1	fibrillin 1	-0.833081399	0.04821878
MFAP5	microfibril associated protein 5	-0.83435766	0.0000412
FAM227A	family with sequence similarity 227 member A	-0.839585824	0.01071622
XIST	X inactive specific transcript	-0.844851335	0.03761973
SMTN	smoothelin	-0.846714583	0.00068041
CARD16	caspase recruitment domain family member 16	-0.851268312	0.02242663
HKDC1	hexokinase domain containing 1	-0.856441891	0.00000127
PLAUR	plasminogen activator; urokinase receptor	-0.860482363	0.0000212
COCH	cochlin	-0.861632156	0.0433689
MIRLET7IHG	MIRLET7I host gene	-0.86203278	0.02828082
HLA-DPB1	major histocompatibility complex; class II; DP beta 1	-0.862761303	0.00787269
CDH6	cadherin 6	-0.864883573	3.69E-11
MC1R	melanocortin 1 receptor	-0.866461976	0.07928172
SEMA6D	semaphorin 6D	-0.867941039	9.13E-12
ATP8B1	ATPase phospholipid transporting 8B1	-0.869655678	0.08188453
FAM229A	family with sequence similarity 229 member A	-0.871054091	0.04732328
CNR1	cannabinoid receptor 1	-0.873335751	0.02930391
TLR4	toll like receptor 4	-0.874200037	0.01689855
ERBB4	erb-b2 receptor tyrosine kinase 4	-0.8747193	0.00058221

NCKAP5	NCK associated protein 5	-0.875802887	0.00993543
CD101	CD101 molecule	-0.879608133	0.01886872
DNAH1	dynein axonemal heavy chain 1	-0.883328969	0.08083857
RBPMS2	RNA binding protein; mRNA processing factor 2	-0.893970557	0.00668365
TC2N	tandem C2 domains; nuclear	-0.898164025	0.00144549
RBMS1	RNA binding motif single stranded interacting protein 1	-0.911372265	0.07236755
TBC1D2	TBC1 domain family member 2	-0.915638872	0.00054582
NPIPB5	nuclear pore complex interacting protein family member B5	-0.916145969	0.00000505
SPTBN5	spectrin beta; non-erythrocytic 5	-0.916294744	0.04732328
ATOH8	atonal bHLH transcription factor 8	-0.930145912	0.0000964
TGFBI	transforming growth factor beta induced	-0.942192949	0.00528139
B3GNT9	UDP-GlcNAc:betaGal beta-1;3-N-acetylglucosaminyltransferase 9	-0.948705847	0.0154246
GRAMD1A	GRAM domain containing 1A	-0.952005334	0.0000268
CDH1	cadherin 1	-0.96313218	8.77E-10
TRERF1	transcriptional regulating factor 1	-0.966598094	0.00682986
CHRNA9	cholinergic receptor nicotinic alpha 9 subunit	-0.971073746	0.08362336
TRIM71	tripartite motif containing 71	-0.97207504	0.02873363
EEPD1	endonuclease/exonuclease/phosphatase family domain containing 1	-0.972737907	0.07730554
CDC42BPG	CDC42 binding protein kinase gamma	-0.987032596	0.07842289
FAM181A	family with sequence similarity 181 member A	-1.005686797	0.00111929
CFAP126	cilia and flagella associated protein 126	-1.009661856	0.02597261
CXCL12	C-X-C motif chemokine ligand 12	-1.020093145	0.00057634

MICAL2	microtubule associated monooxygenase; calponin and LIM domain containing 2	-1.025189499	0.0000962
FAT3	FAT atypical cadherin 3	-1.02685123	0.00113854
TMEM132E	transmembrane protein 132E	-1.030126772	0.00000454
COL5A1	collagen type V alpha 1 chain	-1.032286077	0.00000154
SFRP4	secreted frizzled related protein 4	-1.035228252	9.54E-11
SYT14	synaptotagmin 14	-1.0444407	0.00213452
RN7SL1	RNA component of signal recognition particle 7SL1	-1.051059674	0.00976158
XYLT1	xylosyltransferase 1	-1.059111931	1.54E-16
IER3	immediate early response 3	-1.062132111	2.61E-14
SYNE2	spectrin repeat containing nuclear envelope protein 2	-1.069161815	9.46E-10
PNPLA7	patatin like phospholipase domain containing 7	-1.069306682	0.02927842
INSYN2B	inhibitory synaptic factor family member 2B	-1.071005231	0.05691431
SDK2	sidekick cell adhesion molecule 2	-1.075835491	0.00642033
G0S2	G0/G1 switch 2	-1.083182587	0.01794031
ANKRD63	ankyrin repeat domain 63	-1.083424019	0.02584853
CAMK4	calcium/calmodulin dependent protein kinase IV	-1.089715543	0.00649495
AHNAK	AHNAK nucleoprotein	-1.093715759	6.55E-11
FUT4	fucosyltransferase 4	-1.094650009	0.0936642
RN7SL2	RNA component of signal recognition particle 7SL2	-1.095632723	0.0202614
NPIPB4	nuclear pore complex interacting protein family member B4	-1.104359489	0.0009977
EMILIN1	elastin microfibril interfacer 1	-1.10686478	0.00019573
DLC1	DLC1 Rho GTPase activating protein	-1.125477143	0.02263178
EPAS1	endothelial PAS domain protein 1	-1.134016533	0.00107028
ST3GAL1	ST3 beta-galactoside alpha-2;3-sialyltransferase 1	-1.159209974	0.03131778
ELMOD1	ELMO domain containing 1	-1.159717748	3.95E-11
STC2	stanniocalcin 2	-1.163756481	5.43E-10
MMRN1	multimerin 1	-1.189590245	6.97E-16

PHLDB2	pleckstrin homology like domain family B member 2	-1.194331505	0.02353029
CCDC80	coiled-coil domain containing 80	-1.200788185	0.00000847
WNT5B	Wnt family member 5B	-1.216009379	0.00000399
SIAH3	siah E3 ubiquitin protein ligase family member 3	-1.219587345	0.000083
COLEC12	collectin subfamily member 12	-1.21998766	0.00304848
ZNF582-DT	ZNF582 divergent transcript	-1.229440725	0.08526658
ZDHHC11	zinc finger DHHC-type containing 11	-1.229546197	0.02114538
RYR2	ryanodine receptor 2	-1.248577852	1.66E-12
COL5A2	collagen type V alpha 2 chain	-1.251470953	0.00139432
FHIP1A	FHF complex subunit HOOK interacting protein 1A	-1.265895969	0.00144549
ADAMTSL1	ADAMTS like 1	-1.268152242	0.00136302
EMP1	epithelial membrane protein 1	-1.28206389	1.26E-12
PRX	periaxin	-1.283322109	0.0205549
LIN9	lin-9 DREAM MuvB core complex component	-1.287991068	0.02241135
IGFBP4	insulin like growth factor binding protein 4	-1.29807923	9.44E-08
ESRG	embryonic stem cell related	-1.303480365	0.00496518
SERPINE1	serpin family E member 1	-1.308014791	9.11E-16
TNXB	tenascin XB	-1.31579736	0.00189297
JCAD	junctional cadherin 5 associated	-1.322077443	0.04314495
PCOLCE	procollagen C-endopeptidase enhancer	-1.3328895	0.0000172
ANKRD36C	ankyrin repeat domain 36C	-1.347064363	0.03246243
FMN1	formin 1	-1.355004226	0.0000885
SLFN5	schlafen family member 5	-1.368006378	0.01639861
EPM2A-DT	EPM2A divergent transcript	-1.374360286	0.0464158
CTDSPL	CTD small phosphatase like	-1.375931593	0.00042267
MOK	MOK protein kinase	-1.376085522	6.73E-08
CLDN11	claudin 11	-1.392359871	0.04043221
HLA-DQB2	major histocompatibility complex; class II; DQ beta 2	-1.408552245	0.0505357
MYRF	myelin regulatory factor	-1.416377639	0.02242663
ABCA13	ATP binding cassette subfamily A member 13	-1.417351274	0.0380323

SLIT3	slit guidance ligand 3	-1.418403312	1.7E-08
NBPF20	NBPF member 20	-1.453371046	6.08E-12
TCIRG1	T cell immune regulator 1; ATPase H+ transporting V0 subunit a3	-1.45515537	0.01886872
GALNT5	polypeptide N-acetylgalactosaminyltransferase 5	-1.458206677	2.18E-15
ZBTB3	zinc finger and BTB domain containing 3	-1.482680675	0.09136508
IL1R1	interleukin 1 receptor type 1	-1.487230136	0.00010204
FUT9	fucosyltransferase 9	-1.515836531	7.86E-13
KRT18	keratin 18	-1.517145709	1.36E-11
RCN3	reticulocalbin 3	-1.558790758	0.0172148
MT2A	metallothionein 2A	-1.559697947	9.4E-16
HMGA2	high mobility group AT-hook 2	-1.561071814	1.89E-12
MGST1	microsomal glutathione S-transferase 1	-1.566399269	1.28E-07
PAPSS2	3'-phosphoadenosine 5'-phosphosulfate synthase 2	-1.577794187	0.00260013
ALDH3A1	aldehyde dehydrogenase 3 family member A1	-1.5999461	0.0000946
ATP2A1	ATPase sarcoplasmic/endoplasmic reticulum Ca2+ transporting 1	-1.605030654	0.01196968
EHBP1L1	EH domain binding protein 1 like 1	-1.616691418	3.94E-12
RPL23AP21	ribosomal protein L23a pseudogene 21	-1.649158606	0.02426829
C2orf50	chromosome 2 open reading frame 50	-1.651844985	0.03650058
TGIF2-RAB5IF	TGIF2-RAB5IF readthrough	-1.654035349	0.03526917
IFNE	interferon epsilon	-1.65698578	0.03911349
DSP	desmoplakin	-1.681962326	2.11E-13
KCNQ1OT1	KCNQ1 opposite strand/antisense transcript 1	-1.719041737	0.00150458
CAVIN2	caveolae associated protein 2	-1.722199758	0.01990836
MYH3	myosin heavy chain 3	-1.740891466	0.00316804
MICB	MHC class I polypeptide-related sequence B	-1.742131779	0.00113854
NWD1	NACHT and WD repeat domain containing 1	-1.748230139	0.00935411

FLG	filaggrin	-1.759545097	2.03E-26
CSTF3-DT	CSTF3 divergent transcript	-1.761906641	0.09558998
TFPI	tissue factor pathway inhibitor	-1.774094369	0.00000494
FAT4	FAT atypical cadherin 4	-1.785264271	0.07735274
IL7R	interleukin 7 receptor	-1.789237353	0.00267407
H1-0	H1,0 linker histone	-1.821322079	1.7E-12
CHST9	carbohydrate sulfotransferase 9	-1.833746668	0.00074481
RPLP0P6	ribosomal protein lateral stalk subunit P0 pseudogene 6	-1.845560575	0.08083857
FLNC	filamin C	-1.860916545	9.6E-23
CHODL	chondrolectin	-1.87028912	0.01280116
CLMP	CXADR like membrane protein	-1.875664517	0.01644754
FN1	fibronectin 1	-1.887210735	2.68E-20
OXTR	oxytocin receptor	-1.920040588	0.0000602
GDF10	growth differentiation factor 10	-1.956490385	0.0003299
BICC1	BicC family RNA binding protein 1	-1.980961362	1.44E-09
HMOX1	heme oxygenase 1	-1.996032966	0.00231014
DNER	delta/notch like EGF repeat containing	-1.99848555	0.0000602
TAS2R4	taste 2 receptor member 4	-2.003135584	0.04314495
HECW2	HECT; C2 and WW domain containing E3 ubiquitin protein ligase 2	-2.031741582	2.03E-12
SEMA7A	semaphorin 7A (John Milton Hagen blood group)	-2.032863387	1.83E-07
PSORS1C1	psoriasis susceptibility 1 candidate 1	-2.053144501	0.05354612
SCAND3	SCAN domain containing 3	-2.063472291	1.12E-07
CD68	CD68 molecule	-2.069366315	0.03526917
ETS1	ETS proto-oncogene 1; transcription factor	-2.157807199	5.16E-12
NEAT1	nuclear paraspeckle assembly transcript 1	-2.172746437	0.00000159
MYL9	myosin light chain 9	-2.186935146	9.28E-14
LRRK1	leucine rich repeat kinase 1	-2.195256913	0.02495436
CCR1	C-C motif chemokine receptor 1	-2.201345232	0.03554689
VWF	von Willebrand factor	-2.272114962	0.01683016
C1orf220	chromosome 1 putative open reading frame 220	-2.283413465	0.02444085

COL6A2	collagen type VI alpha 2 chain	-2.293287191	4.5E-39
CPZ	carboxypeptidase Z	-2.296036458	7.18E-14
DOCK10	dedicator of cytokinesis 10	-2.326885219	0.01071622
GATA2	GATA binding protein 2	-2.348964565	0.0020057
GPNMB	glycoprotein nmb	-2.396499073	0.08908382
TOR4A	torsin family 4 member A	-2.447897935	0.00014148
MRGPRF	MAS related GPR family member F	-2.460927804	3.24E-08
SLITRK2	SLIT and NTRK like family member 2	-2.513533921	0.05073386
CASP4	caspase 4	-2.51513028	0.03939465
TMEM200A	transmembrane protein 200A	-2.515587521	0.08526658
TNNT1	troponin T1; slow skeletal type	-2.516137346	0.09144956
COL1A2	collagen type I alpha 2 chain	-2.523183834	1.13E-23
PAPPA	pappalysin 1	-2.557910729	0.00000386
GYPC	glycophorin C (Gerbich blood group)	-2.606985086	0.02218888
ECM1	extracellular matrix protein 1	-2.611954587	1.18E-09
CDCP1	CUB domain containing protein 1	-2.665132385	0.00089308
MYPN	myopalladin	-2.752004203	0.00018273
GREM1	gremlin 1; DAN family BMP antagonist	-2.790953261	2.92E-63
CAPG	capping actin protein; gelsolin like	-2.798366634	0.00011094
BNC1	basonuclin zinc finger protein 1	-2.810645484	0.0000397
ISL1	ISL LIM homeobox 1	-2.821741134	0.08062494
LOC107986626	uncharacterized LOC107986626	-2.82247292	0.08321326
TBX15	T-box transcription factor 15	-2.830622039	0.02737
WDR38	WD repeat domain 38	-2.8394985	0.01187718
CYBA	cytochrome b-245 alpha chain	-2.851692275	0.00113854
PRSS12	serine protease 12	-2.871046253	2.55E-25
SH2D4A	SH2 domain containing 4A	-2.873831269	0.00015068
EMILIN2	elastin microfibril interfacer 2	-2.990021736	0.05792173
TGM2	transglutaminase 2	-3.006443975	0.00224396
FGF5	fibroblast growth factor 5	-3.01018112	0.00000152

SLC16A3	solute carrier family 16 member 3	-3.128025281	3.15E-09
WNT5A	Wnt family member 5A	-3.210624113	0.00000136
PEAR1	platelet endothelial aggregation receptor 1	-3.213907997	0.01096028
ABLIM3	actin binding LIM protein family member 3	-3.286011831	0.0063053
ARHGAP24	Rho GTPase activating protein 24	-3.375221084	0.02843541
MRS2	magnesium transporter MRS2	-3.426367198	0.06132274
DUXAP10	double homeobox A pseudogene 10	-3.457763316	0.00021644
ROR2	receptor tyrosine kinase like orphan receptor 2	-3.479393672	0.00568708
ANPEP	alanyl aminopeptidase; membrane	-3.481011122	8.84E-19
GALNT6	polypeptide N-acetylgalactosaminyltransferase 6	-3.551444654	0.08371024
MME	membrane metalloendopeptidase	-3.567822514	0.03811319
PARP8	poly(ADP-ribose) polymerase family member 8	-3.573738168	0.01200949
HTATIP2	HIV-1 Tat interactive protein 2	-3.577649672	0.05266185
INHBA	inhibin subunit beta A	-3.605432111	0.05713594
RAC2	Rac family small GTPase 2	-3.650718572	0.00085403
BGN	biglycan	-3.753096844	0.00015313
STRC	stereocilin	-3.802351542	0.05525882
FAM167A	family with sequence similarity 167 member A	-3.893182373	0.00012044
PRLR	prolactin receptor	-3.952103286	0.01315286
COL12A1	collagen type XII alpha 1 chain	-4.01503683	7.72E-21
MUC16	mucin 16; cell surface associated	-4.025392716	0.05119154
LY6K	lymphocyte antigen 6 family member K	-4.170562478	0.000046
FAP	fibroblast activation protein alpha	-4.222710906	0.00134753
RAB3B	RAB3B; member RAS oncogene family	-4.245390823	7.63E-23
ABI3BP	ABI family member 3 binding protein	-4.400391656	0.0000219
PRRX2	paired related homeobox 2	-4.514132022	0.01910976

STAT6	signal transducer and activator of transcription 6	-4.566207267	1.97E-07
SLC43A3	solute carrier family 43 member 3	-4.601618448	1.72E-10
CASP10	caspase 10	-4.625114052	0.00680889
CLEC14A	C-type lectin domain containing 14A	-4.868594437	0.00770617
NLRP1	NLR family pyrin domain containing 1	-4.902421941	0.0464158
LTBR	lymphotoxin beta receptor	-4.987612283	7.54E-08
GPRC5A	G protein-coupled receptor class C group 5 member A	-5.045729254	5.74E-09
AOX1	aldehyde oxidase 1	-5.591523591	3.28E-12
CDK15	cyclin dependent kinase 15	-5.600045389	0.00119705
CGAS	cyclic GMP-AMP synthase	-5.662689489	0.00378838
FOXD1	forkhead box D1	-5.916797996	1.12E-09
VEGFC	vascular endothelial growth factor C	-5.942763755	0.00000152
ADAMTS2	ADAM metallopeptidase with thrombospondin type 1 motif 2	-6.014738863	0.00022417
VRK2	VRK serine/threonine kinase 2	-6.022327375	0.00836422
IGF2	insulin like growth factor 2	-6.048863771	0.0000168
SLC17A9	solute carrier family 17 member 9	-6.221951951	0.00031702
WNK4	WNK lysine deficient protein kinase 4	-6.227344751	0.00000633
FOXF1	forkhead box F1	-6.24723364	0.03554689
SERPINB7	serpin family B member 7	-6.253917511	0.04075125
ENG	endoglin	-6.338889441	4.41E-20
SERPINB2	serpin family B member 2	-6.351626074	0.00000193
PSG4	pregnancy specific beta-1-glycoprotein 4	-6.390210699	0.00041255
CCBE1	collagen and calcium binding EGF domains 1	-6.395723053	1.01E-08
IL1B	interleukin 1 beta	-6.67302459	0.0000933
QNG1	Q-nucleotide N-glycosylase 1	-6.67559355	0.00085403
MSC-AS1	MSC antisense RNA 1	-6.679625179	0.06063745
FOXC2	forkhead box C2	-6.684096799	0.00000505
CCN4	cellular communication network factor 4	-6.751940537	0.04586664
COL3A1	collagen type III alpha 1 chain	-6.883806486	0.02274239
HOXA9	homeobox A9	-7.147705464	0.00768549

FLI1	Fli-1 proto-oncogene; ETS transcription factor	-7.25314425	0.00181679
CDH13	cadherin 13	-7.28135297	9.13E-12
FOXL1	forkhead box L1	-7.285342468	0.00041868
MT1E	metallothionein 1E	-7.465402469	0.00020912
TFAP2A	transcription factor AP-2 alpha	-7.476711817	0.0000183
KRT34	keratin 34	-7.786944414	0.0000648
NR2F2	nuclear receptor subfamily 2 group F member 2	-8.139071005	2.01E-24
COL6A3	collagen type VI alpha 3 chain	-8.579070651	1.67E-24
DKK1	dickkopf WNT signaling pathway inhibitor 1	-9.013314225	0.0000499
TBX3	T-box transcription factor 3	-9.129121874	3.78E-12
FXYD5	FXYD domain containing ion transport regulator 5	-9.887951528	6.54E-19
SRGN	serglycin	-10.34593325	7.75E-22
UTP14C	UTP14C small subunit processome component	-30	1.53E-11

Supplementary Table 5: DEGs in DHEA vs Control conditions (log2FoldChange ≤ -0.59 or ≥ +0.59, adjusted p-value< 0.1)

Gene symbol	Gene name	log2FoldChange	padj
UTP14C	UTP14C small subunit processome component	-14.37363388	0.010816272
DKK1	dickkopf WNT signaling pathway inhibitor 1	-10.31586065	2.08E-21
COL6A3	collagen type VI alpha 3 chain	-9.040110543	0.000384842
TBX3	T-box transcription factor 3	-8.890551621	1.65E-11
MLPH	melanophilin	-8.788476688	3.93E-11
SRGN	serglycin	-8.709068469	2.38E-22
NR2F2	nuclear receptor subfamily 2 group F member 2	-8.148359725	2.71E-23
FOXC2	forkhead box C2	-8.044055745	0.000000154
SERPINB2	serpin family B member 2	-8.031784768	6.63E-09
KRT34	keratin 34	-7.561135468	0.000154228
COL3A1	collagen type III alpha 1 chain	-7.49274345	4.39E-09
FXYD5	FXYD domain containing ion transport regulator 5	-7.403437251	8.74E-14
IL1B	interleukin 1 beta	-7.359460391	0.0000512
CCBE1	collagen and calcium binding EGF domains 1	-7.279818556	4.77E-09
EBF3	EBF transcription factor 3	-6.94715426	0.012038123
HOXA9	homeobox A9	-6.919930112	0.013678586
LINC01116	long intergenic non-protein coding RNA 1116	-6.687023127	0.042534971
MT1E	metallothionein 1E	-6.539047651	0.001275439
FOXF1	forkhead box F1	-6.459499386	0.092918099
CDH13	cadherin 13	-6.278408789	2.18E-10
ENG	endoglin	-6.053783486	5.88E-28
CCN4	cellular communication network factor 4	-6.038329337	0.049093507
PSG4	pregnancy specific beta-1-glycoprotein 4	-5.942289466	0.003170052
FLI1	Fli-1 proto-oncogene; ETS transcription factor	-5.929612484	0.005054463
GPRC5A	G protein-coupled receptor class C group 5 member A	-5.866282852	5.28E-16
VEGFC	vascular endothelial growth factor C	-5.791227349	0.00000279
LTBR	lymphotoxin beta receptor	-5.752243605	5.53E-08
AOX1	aldehyde oxidase 1	-5.726315198	3.63E-10
FOXD1	forkhead box D1	-5.716509727	0.000000602
IGF2	insulin like growth factor 2	-5.702216572	0.000260806

WNK4	WNK lysine deficient protein kinase 4	-5.626335595	0.00000998
CDK15	cyclin dependent kinase 15	-5.546027294	0.002153921
NLRP1	NLR family pyrin domain containing 1	-5.464611123	0.013125166
VRK2	VRK serine/threonine kinase 2	-5.385861377	0.003287609
KRTAP2-3	keratin associated protein 2-3	-5.354674455	0.042278073
SLC17A9	solute carrier family 17 member 9	-5.323037057	0.000174382
FAM167A	family with sequence similarity 167 member A	-5.203276252	0.000000103
RAC2	Rac family small GTPase 2	-5.15164204	0.001480878
PARP8	poly(ADP-ribose) polymerase family member 8	-5.144202578	0.001275439
CLEC14A	C-type lectin domain containing 14A	-5.073042671	0.001444987
ABI3BP	ABI family member 3 binding protein	-5.067665404	0.001295016
TFAP2A	transcription factor AP-2 alpha	-4.867617496	0.072572388
ARHGAP24	Rho GTPase activating protein 24	-4.848987129	0.004476855
CASP10	caspase 10	-4.719127097	0.044124423
HTATIP2	HIV-1 Tat interactive protein 2	-4.628428576	0.01216336
CGAS	cyclic GMP-AMP synthase	-4.569174157	0.013301504
GALNT6	polypeptide N-acetylgalactosaminyltransferase 6	-4.497677492	0.00695451
SLC43A3	solute carrier family 43 member 3	-4.496710959	3.34E-12
RAB3B	RAB3B; member RAS oncogene family	-3.920093418	1.49E-21
LOC12812582	uncharacterized LOC128125822	-3.893015279	0.073643948
PRRX2	paired related homeobox 2	-3.876035255	0.001110398
SLC16A3	solute carrier family 16 member 3	-3.867352019	9.11E-11
LY6K	lymphocyte antigen 6 family member K	-3.867038497	0.0000397
ISL1	ISL LIM homeobox 1	-3.751134752	0.028624454
EMILIN2	elastin microfibril interfacer 2	-3.633604393	0.000436703
BGN	biglycan	-3.466861271	0.000163926
TRPV2	transient receptor potential cation channel subfamily V member 2	-3.387548842	0.072588598
ABLIM3	actin binding LIM protein family member 3	-3.27210719	0.030260683
STAT6	signal transducer and activator of transcription 6	-3.218131923	0.004169822
MYPN	myopalladin	-3.215849287	0.001745816
ROR2	receptor tyrosine kinase like orphan receptor 2	-3.188419239	0.01168197
CYBA	cytochrome b-245 alpha chain	-3.184218513	0.000750795
INHBA	inhibin subunit beta A	-3.110833001	0.024464752

SH2D4A	SH2 domain containing 4A	-3.091020017	0.0000241
CAPG	capping actin protein; gelsolin like	-3.032528977	0.0000612
ANPEP	alanyl aminopeptidase; membrane	-3.007653729	1.08E-15
TNNT1	troponin T1; slow skeletal type	-2.994215361	0.024464752
BNC1	basonuclin zinc finger protein 1	-2.976826353	0.0000325
LRRK1	leucine rich repeat kinase 1	-2.960472556	0.006134115
CALHM2	calcium homeostasis modulator family member 2	-2.807756297	0.003789757
WNT5A	Wnt family member 5A	-2.741173404	0.000000264
ECM1	extracellular matrix protein 1	-2.713220278	1.91E-10
ITGA10	integrin subunit alpha 10	-2.688269891	0.009489528
PEAR1	platelet endothelial aggregation receptor 1	-2.673702448	0.005979712
PAPPA	pappalysin 1	-2.560076062	0.0000449
CLMP	CXADR like membrane protein	-2.55466943	0.000688918
GATA2	GATA binding protein 2	-2.539764363	0.00000269
TBX15	T-box transcription factor 15	-2.479214654	0.035853447
GREM1	gremlin 1; DAN family BMP antagonist	-2.477001874	3.78E-43
COL6A2	collagen type VI alpha 2 chain	-2.460145007	8.96E-38
ETS1	ETS proto-oncogene 1; transcription factor	-2.409507603	2.33E-12
ADAMTS6	ADAM metallopeptidase with thrombospondin type 1 motif 6	-2.404747041	0.0000438
DNAH12	dynein axonemal heavy chain 12	-2.395487499	0.044753467
SLITRK2	SLIT and NTRK like family member 2	-2.376460891	0.070912195
COL1A2	collagen type I alpha 2 chain	-2.362334721	5.52E-13
KCNQ1OT1	KCNQ1 opposite strand/antisense transcript 1	-2.344607253	0.0000288
DUXAP10	double homeobox A pseudogene 10	-2.334622234	0.005826723
C1orf220	chromosome 1 putative open reading frame 220	-2.328184447	0.020785181
TMEM200B	transmembrane protein 200B	-2.2974084	0.043739375
HMSD	histocompatibility minor serpin domain containing	-2.296117679	0.034616614
CPZ	carboxypeptidase Z	-2.285811648	2.91E-13
NEAT1	nuclear paraspeckle assembly transcript 1	-2.22269519	0.000000328
DNER	delta/notch like EGF repeat containing	-2.143234812	0.0000425
MICB	MHC class I polypeptide-related sequence B	-2.123429855	0.000157762
MRGPRF	MAS related GPR family member F	-2.109027728	0.00000171

OXTR	oxytocin receptor	-2.096333403	0.0000282
FAT4	FAT atypical cadherin 4	-2.033349164	0.035020458
RPLP0P6	ribosomal protein lateral stalk subunit P0 pseudogene 6	-2.02555221	0.010614133
LUM	lumican	-1.989893287	0.065505924
EPM2A-DT	EPM2A divergent transcript	-1.94799668	0.017596633
TAS2R4	taste 2 receptor member 4	-1.93550334	0.08310567
FLNC	filamin C	-1.935312189	5.88E-28
FN1	fibronectin 1	-1.929885173	1.67E-17
BICC1	BicC family RNA binding protein 1	-1.925576177	2.85E-10
HMOX1	heme oxygenase 1	-1.918755095	0.003800235
PRSS12	serine protease 12	-1.896977396	1.18E-10
H1-0	H1,0 linker histone	-1.895502926	2.84E-14
MYRF	myelin regulatory factor	-1.891261541	0.001382789
QPRT	quinolinate phosphoribosyltransferase	-1.865899617	0.017692635
GABRE	gamma-aminobutyric acid type A receptor subunit epsilon	-1.86336164	0.016813508
TOR4A	torsin family 4 member A	-1.827148259	0.005446546
CLDN11	claudin 11	-1.818368251	0.001162225
MYL9	myosin light chain 9	-1.774732342	1.53E-09
NWD1	NACHT and WD repeat domain containing 1	-1.749787016	0.005054463
TMEM154	transmembrane protein 154	-1.727764956	0.066831134
TCEA3	transcription elongation factor A3	-1.726189845	0.084724366
MUC20	mucin 20; cell surface associated	-1.71180855	0.095629998
SCAND3	SCAN domain containing 3	-1.708415163	0.022820085
IL7R	interleukin 7 receptor	-1.700472344	0.035103953
SEMA7A	semaphorin 7A (John Milton Hagen blood group)	-1.676417109	0.000169206
CHODL	chondrolectin	-1.674728833	0.016981326
ALDH3A1	aldehyde dehydrogenase 3 family member A1	-1.658564795	0.0000512
MT2A	metallothionein 2A	-1.618773302	1.76E-19
PAX8-AS1	PAX8 antisense RNA 1	-1.61111173	0.099186861
FLG	filaggrin	-1.609628963	3.11E-13
MOK	MOK protein kinase	-1.607451373	1.27E-10
SLIT3	slit guidance ligand 3	-1.575611224	3.27E-08
MGST1	microsomal glutathione S-transferase 1	-1.539843099	0.0000009
DSP	desmoplakin	-1.531174236	7.59E-08
TFPI	tissue factor pathway inhibitor	-1.5296515	0.0000512
SCART1	scavenger receptor family member expressed on T cells 1	-1.528920523	0.0197271
PCOLCE	procollagen C-endopeptidase enhancer	-1.520640774	0.0000633

JCAD	junctional cadherin 5 associated	-1.490094155	0.003148059
MYH3	myosin heavy chain 3	-1.489251729	0.010341072
KRT18	keratin 18	-1.457763656	5.34E-09
TNFRSF1B	TNF receptor superfamily member 1B	-1.453775448	0.016813508
GALNT5	polypeptide N-acetylgalactosaminyltransferase 5	-1.440889862	4.97E-25
HMGA2	high mobility group AT-hook 2	-1.42709518	1.63E-09
RCN3	reticulocalbin 3	-1.421401106	0.035109689
GDF10	growth differentiation factor 10	-1.419480768	0.032686933
MVP-DT	MVP divergent transcript	-1.400987027	0.037507694
PHLDB2	pleckstrin homology like domain family B member 2	-1.379923137	0.001295016
FMN1	formin 1	-1.375195547	0.001232476
CTDSPL	CTD small phosphatase like	-1.374276849	0.000480478
LOC10012953	small nuclear ribonucleoprotein polypeptide N pseudogene	-1.354957367	0.066114215
LRRC2	leucine rich repeat containing 2	-1.35387085	0.004106115
PNPLA7	patatin like phospholipase domain containing 7	-1.349324884	0.00840918
NBPF20	NBPF member 20	-1.313316455	4.88E-08
IGFBP4	insulin like growth factor binding protein 4	-1.305298417	6.89E-08
SP140L	SP140 nuclear body protein like	-1.292165705	0.043620727
H2BC19P	H2B clustered histone 19; pseudogene	-1.289551379	0.000000141
SCEL	sciellin	-1.288253488	0.097470139
ELMOD1	ELMO domain containing 1	-1.285491637	1.22E-18
EHBP1L1	EH domain binding protein 1 like 1	-1.285265344	0.00000269
NPIPB4	nuclear pore complex interacting protein family member B4	-1.27804772	0.000647735
IL1R1	interleukin 1 receptor type 1	-1.276229882	0.013641273
CCDC80	coiled-coil domain containing 80	-1.269080502	0.000412394
SERPINE1	serpin family E member 1	-1.267228956	8.74E-14
TRMT9B	tRNA methyltransferase 9B (putative)	-1.239205622	0.003872374
SIAH3	siah E3 ubiquitin protein ligase family member 3	-1.221685049	0.000176732
SMTN	smoothelin	-1.216186147	1.18E-08
HLA-DQB1	major histocompatibility complex; class II; DQ beta 1	-1.200870786	0.054596779
TMEM132E	transmembrane protein 132E	-1.197962243	4.77E-09
TGFBI	transforming growth factor beta induced	-1.160522094	0.000591063
UNC80	unc-80 homolog; NALCN channel complex subunit	-1.147467814	0.002878543

EMILIN1	elastin microfibril interfacer 1	-1.142860026	0.003595541
FUT9	fucosyltransferase 9	-1.139342391	0.00000137
WNT5B	Wnt family member 5B	-1.09641307	0.000000574
G0S2	G0/G1 switch 2	-1.088260115	0.012309388
CRISPLD2	cysteine rich secretory protein LCCL domain containing 2	-1.08058601	0.040095343
DLC1	DLC1 Rho GTPase activating protein	-1.080016258	0.064622987
XYLT1	xylosyltransferase 1	-1.07996004	4.77E-09
SYNE2	spectrin repeat containing nuclear envelope protein 2	-1.075451722	2.59E-08
ADAMTSL1	ADAMTS like 1	-1.073782101	0.009058929
EMP1	epithelial membrane protein 1	-1.07240061	0.0000181
ANKRD63	ankyrin repeat domain 63	-1.071084504	0.002342124
EHHADH	enoyl-CoA hydratase and 3-hydroxyacyl CoA dehydrogenase	-1.055521746	0.060650655
TC2N	tandem C2 domains; nuclear	-1.051660708	0.000821526
FHIP1A	FHF complex subunit HOOK interacting protein 1A	-1.046994735	0.05560683
CXCL12	C-X-C motif chemokine ligand 12	-1.0456904	0.000539445
COL5A2	collagen type V alpha 2 chain	-1.02571899	0.013374266
MMRN1	multimerin 1	-1.019799595	2.29E-14
AHNAK	AHNAK nucleoprotein	-1.016389015	1.05E-11
TRERF1	transcriptional regulating factor 1	-0.983688326	0.003129972
TNXB	tenascin XB	-0.977917298	0.05560683
SFRP4	secreted frizzled related protein 4	-0.973818572	0.000000176
FAM181A	family with sequence similarity 181 member A	-0.972579144	0.00412721
PDE8B	phosphodiesterase 8B	-0.966356156	0.00088293
NPIPB5	nuclear pore complex interacting protein family member B5	-0.933022324	0.00000229
CHRFAM7A	CHRNA7 (exons 5-10) and FAM7A (exons A-E) fusion	-0.929552978	0.084766303
HLA-DPB1	major histocompatibility complex; class II; DP beta 1	-0.925556873	0.004169822
TRPV3	transient receptor potential cation channel subfamily V member 3	-0.916667643	0.07029148
XIST	X inactive specific transcript	-0.916253742	0.001170814
MC1R	melanocortin 1 receptor	-0.914544228	0.070785967
SYT14	synaptotagmin 14	-0.912985949	0.001975877
TES	testin LIM domain protein	-0.912312529	0.037183796
KANTR	KANTR integral membrane protein	-0.908980507	0.004362395
IER3	immediate early response 3	-0.902278073	0.000000055
FRMD6	FERM domain containing 6	-0.901853474	0.004362395
PLAUR	plasminogen activator; urokinase receptor	-0.899270095	0.0000176

ERBB4	erb-b2 receptor tyrosine kinase 4	-0.898317772	0.000000409
DRC1	dynein regulatory complex subunit 1	-0.893023622	0.0020955
GRAMD1A	GRAM domain containing 1A	-0.890096525	0.021092425
SH3BP1	SH3 domain binding protein 1	-0.886999127	0.003595541
COL5A1	collagen type V alpha 1 chain	-0.884601779	0.014324782
LOXL2	lysyl oxidase like 2	-0.86699542	0.0000022
CDH6	cadherin 6	-0.863193177	2.88E-10
ETS2	ETS proto-oncogene 2; transcription factor	-0.861585729	0.016503496
SORL1	sortilin related receptor 1	-0.852586358	1.43E-09
RYR2	ryanodine receptor 2	-0.85065296	0.000556322
DNAH6	dynein axonemal heavy chain 6	-0.85017173	0.099260747
RBMS1	RNA binding motif single stranded interacting protein 1	-0.849514438	0.057451186
DNHD1	dynein heavy chain domain 1	-0.84868977	0.077546069
FBN1	fibrillin 1	-0.834791341	0.091623521
IL11RA	interleukin 11 receptor subunit alpha	-0.834349293	0.099186861
CNR1	cannabinoid receptor 1	-0.832235488	0.069385195
TBC1D2	TBC1 domain family member 2	-0.827206078	0.001788546
MIRLET7IHG	MIRLET7I host gene	-0.819257647	0.067241073
ATOH8	atonal bHLH transcription factor 8	-0.816170413	0.00336904
PDE3A	phosphodiesterase 3A	-0.815076748	0.02317513
STC2	stanniocalcin 2	-0.808480621	0.000412394
FAM229A	family with sequence similarity 229 member A	-0.807776169	0.032686933
SEMA6D	semaphorin 6D	-0.799264788	0.0000921
FOXJ1	forkhead box J1	-0.797154012	0.00000656
RALYL	RALY RNA binding protein like	-0.796358846	0.091439497
C6orf118	chromosome 6 open reading frame 118	-0.790767964	0.00327254
FKBP11	FKBP prolyl isomerase 11	-0.786372613	0.007535846
A2M	alpha-2-macroglobulin	-0.782885866	0.006526702
CYP1B1	cytochrome P450 family 1 subfamily B member 1	-0.781440992	0.035304698
EPPK1	epiplakin 1	-0.778515934	0.038212813
FTX	FTX transcript; XIST regulator	-0.767608958	0.00412721
VPS13D	vacuolar protein sorting 13 homolog D	-0.766084225	0.002259375
TLR4	toll like receptor 4	-0.766013764	0.03638057
B3GALT1	beta-1;3-galactosyltransferase 1	-0.752977146	0.00000208
ITGB3	integrin subunit beta 3	-0.751749561	0.04069094
DNMT1	DNA methyltransferase 1	-0.74983196	0.001057771
GNAO1	G protein subunit alpha o1	-0.748691123	0.001480878

ALMS1	ALMS1 centrosome and basal body associated protein	-0.747594827	0.002354923
OPLAH	5-oxoprolinase; ATP-hydrolysing	-0.746954258	0.01865007
KMT2D	lysine methyltransferase 2D	-0.743982576	0.000227648
ZBTB38	zinc finger and BTB domain containing 38	-0.743521277	0.00098052
LMO7	LIM domain 7	-0.74133347	0.012247018
SETBP1	SET binding protein 1	-0.72839571	0.097885358
B3GALT5	beta-1;3-galactosyltransferase 5	-0.727934124	0.08110254
HKDC1	hexokinase domain containing 1	-0.724141568	0.0000438
NAV2	neuron navigator 2	-0.723188588	0.001824583
LY6H	lymphocyte antigen 6 family member H	-0.72067768	0.087391428
HSPA1A	heat shock protein family A (Hsp70) member 1A	-0.719081715	0.009589204
GSDMD	gasdermin D	-0.718985924	0.04747803
LRCH3	leucine rich repeats and calponin homology domain containing 3	-0.71127073	0.061473531
NBPF19	NBPF member 19	-0.707900013	0.039621596
CDH1	cadherin 1	-0.706908546	0.004063638
TEK	TEK receptor tyrosine kinase	-0.702773421	0.000819805
FBN2	fibrillin 2	-0.700755917	1.54E-08
HLA-DMB	major histocompatibility complex; class II; DM beta	-0.695192078	0.004000811
PLEKHG4B	pleckstrin homology and RhoGEF domain containing G4B	-0.692972351	2.77E-13
MICAL2	microtubule associated monooxygenase; calponin and LIM domain containing 2	-0.692211368	0.03638057
LRRC15	leucine rich repeat containing 15	-0.692188237	0.005777926
TTLL3	tubulin tyrosine ligase like 3	-0.691686132	0.044721887
AQP4	aquaporin 4	-0.689626852	8.74E-14
MDN1	midasin AAA ATPase 1	-0.689567145	0.005979712
CCDC57	coiled-coil domain containing 57	-0.684225536	0.000605285
SLC1A2	solute carrier family 1 member 2	-0.682614873	0.0000633
CD101	CD101 molecule	-0.678294436	0.045168548
DTX4	deltex E3 ubiquitin ligase 4	-0.674725435	0.021716971
GPR135	G protein-coupled receptor 135	-0.671208246	0.049093507
ITFG2	integrin alpha FG-GAP repeat containing 2	-0.670106866	0.000781459
ELF4	E74 like ETS transcription factor 4	-0.668758482	0.010042611
STRIP2	striatin interacting protein 2	-0.667574306	0.018598321
NBPF14	NBPF member 14	-0.66426778	0.009351737
HEG1	heart development protein with EGF like domains 1	-0.663007825	0.004027961
MYO18A	myosin XVIIIA	-0.655855656	0.000000072

GABRQ	gamma-aminobutyric acid type A receptor subunit theta	-0.651866038	0.001756516
MSH5	mutS homolog 5	-0.650601082	0.02761749
DOK6	docking protein 6	-0.649497668	0.016572376
CARD16	caspase recruitment domain family member 16	-0.647473597	0.03913011
NOTCH2NLA	notch 2 N-terminal like A	-0.645753571	0.026329496
RFX4	regulatory factor X4	-0.642712293	0.004946845
NBEA	neurobeachin	-0.636798901	0.043739375
KMT2A	lysine methyltransferase 2A	-0.635915985	0.085349273
PLXND1	plexin D1	-0.635226804	0.022820085
MYBL1	MYB proto-oncogene like 1	-0.6263942	0.01554188
ENPP2	ectonucleotide pyrophosphatase/phosphodiesterase 2	-0.625006081	0.095398546
ZNF519	zinc finger protein 519	-0.623504637	0.08967352
TENT5B	terminal nucleotidyltransferase 5B	-0.622559595	0.000179704
ADAMTS9	ADAM metallopeptidase with thrombospondin type 1 motif 9	-0.621356906	0.020785181
RALGAPA2	Ral GTPase activating protein catalytic subunit alpha 2	-0.615746174	0.006895464
HLA-DRB5	major histocompatibility complex; class II; DR beta 5	-0.614632539	0.099186861
AAK1	AP2 associated kinase 1	-0.612385647	0.000432635
NAV1	neuron navigator 1	-0.608501498	0.001480878
COCH	cochlin	-0.605738296	0.054121145
SEZ6	seizure related 6 homolog	-0.602381578	0.080231675
AXL	AXL receptor tyrosine kinase	-0.601730639	0.000688918
F3	coagulation factor III; tissue factor	-0.601177202	0.000000113
FAM124A	family with sequence similarity 124 member A	-0.596516457	0.007535846
ITPR3	inositol 1;4;5-trisphosphate receptor type 3	-0.591073573	0.067564082
BAHCC1	BAH domain and coiled-coil containing 1	-0.590989358	0.045409162
ADCY9	adenylate cyclase 9	-0.590628373	0.018217948
GZF1	GDNF inducible zinc finger protein 1	0.591108234	0.001533726
PXDN	peroxidasin	0.592464838	0.001444987
ESPN	espin	0.62069826	0.052124112
DMRTA2	DMRT like family A2	0.62084722	0.06941049
GLCCI1	glucocorticoid induced 1	0.621580214	0.058635659
DAB1	DAB adaptor protein 1	0.624639099	0.00000823
PGM2L1	phosphoglucomutase 2 like 1	0.636307307	0.004475359
TSPAN15	tetraspanin 15	0.641774296	0.081058272
PAPOLG	poly(A) polymerase gamma	0.653151049	0.066114215

PHYHIPL	phytanoyl-CoA 2-hydroxylase interacting protein like	0.656947051	0.00278061
DMD	dystrophin	0.658677897	3.67E-08
KCNN4	potassium calcium-activated channel subfamily N member 4	0.659946159	0.068847673
CD24	CD24 molecule	0.669151126	0.008585706
PSEN2	presenilin 2	0.697461332	0.003636655
WDR70	WD repeat domain 70	0.705202542	0.015904491
ACVR2A	activin A receptor type 2A	0.707585074	0.028513167
ANOS1	anosmin 1	0.710853299	0.014341422
ELAVL3	ELAV like RNA binding protein 3	0.712606979	0.009480714
JAKMIP2-AS1	JAKMIP2 antisense RNA 1	0.721678171	0.001862173
GNG2	G protein subunit gamma 2	0.723055619	0.00000734
ZNF175	zinc finger protein 175	0.724672545	0.039334933
SHROOM2	shroom family member 2	0.72951821	0.034710313
ND3	NADH dehydrogenase subunit 3	0.739900546	0.0000886
SFMBT2	Scm like with four mbt domains 2	0.741025521	0.043629821
TMEM178B	transmembrane protein 178B	0.752513957	0.071617429
TNFRSF19	TNF receptor superfamily member 19	0.757644979	0.000335059
PAK3	p21 (RAC1) activated kinase 3	0.816686292	0.001090801
CXXC4	CXXC finger protein 4	0.823472383	0.097794213
FILIP1L	filamin A interacting protein 1 like	0.839961062	0.037343921
ST18	ST18 C2H2C-type zinc finger transcription factor	0.842043935	0.064631826
SULF2	sulfatase 2	0.847397349	0.000152147
IGFBP5	insulin like growth factor binding protein 5	0.851889905	0.000000175
GIN1	gypsy retrotransposon integrase 1	0.854821384	0.08068552
CCDC82	coiled-coil domain containing 82	0.867483282	0.003491976
KREMEN2	kringle containing transmembrane protein 2	0.868140808	0.027431813
PCDH19	protocadherin 19	0.869557627	0.000195675
HBA1	hemoglobin subunit alpha 1	0.869897836	0.005775717
INSM1	INSM transcriptional repressor 1	0.88322918	0.003129972
BCAN	brevican	0.884073002	0.08192971
HOXB3	homeobox B3	0.922521768	0.006590392
SPOCK1	SPARC (osteonectin); cwcv and kazal like domains proteoglycan 1	0.956041146	0.029236673
TOX3	TOX high mobility group box family member 3	0.957033152	0.000000103
COL14A1	collagen type XIV alpha 1 chain	0.972370175	0.003873316
PRDM1	PR/SET domain 1	0.986089431	0.003189101
RNF180	ring finger protein 180	0.987600079	0.000107729
SCD5	stearoyl-CoA desaturase 5	1.002869794	1.4E-11

SERPINF1	serpin family F member 1	1.004498952	0.000699864
NFE2L3	NFE2 like bZIP transcription factor 3	1.010335181	0.001277472
RGS16	regulator of G protein signaling 16	1.016354249	0.0000512
APCDD1	APC down-regulated 1	1.037282139	0.0000397
DLX5	distal-less homeobox 5	1.047546722	0.000176732
LCORL	ligand dependent nuclear receptor corepressor like	1.051801902	0.000802188
PMEPA1	prostate transmembrane protein; androgen induced 1	1.05414399	1.27E-08
C1orf50	chromosome 1 open reading frame 50	1.061834327	0.010042611
C4B	complement C4B (Chido blood group)	1.062509283	0.052587096
ANK3	ankyrin 3	1.063841705	0.000138116
ENTREP2	endosomal transmembrane epsin interactor 2	1.076275182	0.010341072
PCDH1	protocadherin 1	1.119595237	0.000158127
CACNG7	calcium voltage-gated channel auxiliary subunit gamma 7	1.14103995	0.000000143
CHRNA1	cholinergic receptor nicotinic alpha 1 subunit	1.149468528	0.095398546
ANGPTL1	angiopoietin like 1	1.18102188	0.00000037
NALF2	NALCN channel auxiliary factor 2	1.186567854	0.052927375
NDRG2	NDRG family member 2	1.192516932	0.001057841
ROR1	receptor tyrosine kinase like orphan receptor 1	1.205087694	0.018389817
DLGAP1	DLG associated protein 1	1.215592373	0.000222419
PKP1	plakophilin 1	1.237848308	0.000348475
CRMP1	collapsin response mediator protein 1	1.240474763	3.93E-11
SPRN	shadow of prion protein	1.268370954	0.0135195
NRXN3	neurexin 3	1.443433231	0.042878711
DIPK1C	divergent protein kinase domain 1C	1.447898256	0.000263044
LINC00707	long intergenic non-protein coding RNA 707	1.485082906	0.018455473
DENND1C	DENN domain containing 1C	1.517338767	0.034710313
VAV3	vav guanine nucleotide exchange factor 3	1.525524276	0.091033
DNM3	dynamin 3	1.532842039	0.003754855
SORCS2	sortilin related VPS10 domain containing receptor 2	1.544733757	0.00000153
SPNS2	SPNS lysolipid transporter 2; sphingosine-1-phosphate	1.549950114	0.068847673
RUNDC3A	RUN domain containing 3A	1.572280961	0.067701484

CLVS2	clavesin 2	1.638863639	0.049026632
MMP7	matrix metallopeptidase 7	1.686138724	0.010744612
SERPINA3	serpin family A member 3	1.761819077	0.080270116
NTRK2	neurotrophic receptor tyrosine kinase 2	1.765057885	0.000000667
RGR	retinal G protein coupled receptor	1.887684992	0.001115605
RGS8	regulator of G protein signaling 8	1.887890239	0.00000269
NPL	N-acetylneuraminate pyruvate lyase	2.029700764	0.001529917
CHRD	chordin	2.069479589	0.027984351
COL25A1	collagen type XXV alpha 1 chain	2.095936496	0.017885134
SLC12A5	solute carrier family 12 member 5	2.227695464	0.070912195
RLBP1	retinaldehyde binding protein 1	2.265904237	0.042878711
GJB2	gap junction protein beta 2	2.398949434	0.02759988
MYT1	myelin transcription factor 1	2.64535458	0.00336904
MASP1	MBL associated serine protease 1	2.651801622	0.004134008
CPLX2	complexin 2	2.914099173	0.000191478
MGAT4C	MGAT4 family member C	2.952677534	0.000851678
FBN3	fibrillin 3	3.5147032	0.022403122
ZIC3	Zic family member 3	3.895802849	0.061016846
EIF3EP1	EIF3E pseudogene 1	5.730570994	0.086079692
IFIT2	interferon induced protein with tetratricopeptide repeats 2	21.59278532	0.0000212

Supplementary Table 6: Ingenuity pathway analysis of DEGs in IL1β vs Control condition (p<0.05, z-score>|2|)

Ingenuity Canonical Pathways	-log(p-value)	Ratio	z-score	Molecules
Interferon gamma signaling	1,57E01	5,91E-01	6,102	B2M,CAMK2A,CIITA,GBP1,GBP2,GBP3,GBP4,GBP5,HLA-A,HLA-B,HLA-C,HLA-DQA1,HLA-DRA,HLA-DRB1,HLA-DRB5,HLA-E,HLA-F,HLA-G,ICAM1,IFI30,IFNGR2,IRF1,IRF2,IRF7,IRF9,JAK2,MT2A,NCAM1,OAS1,OAS2,OAS3,OASL,PML,PTAFR,PTPN2,SOCS1,SP100,STAT1,TRIM14,TRIM2,TRIM21,TRIM22,TRIM25,TRIM26,TRIM34,TRIM35,TRIM38,TRIM45,TRIM5,TRIM6,TRIM8,VCAM1
Interferon alpha/beta signaling	1,48E01	6,67E-01	6,325	ADAR,BST2,GBP2,HLA-A,HLA-B,HLA-C,HLA-E,HLA-F,HLA-G,IFI27,IFI35,IFI6,IFIT1,IFIT2,IFIT3,IFIT5,IFITM1,IFITM2,IFITM3,IRF1,IRF2,IRF7,IRF9,ISG15,ISG20,MX1,MX2,OAS1,OAS2,OAS3,OASL,PSMB8,RNASEL,RSAD2,SAMHD1,SOCS1,STAT1,STAT2,USP18,XAF1

Pathogen Induced Cytokine Storm S	1,11E01	3,59E-01	6,226	ADRA1A,AIM2,C3,C5,CASP1,CASP7,CASP8,CCL2,CCL20,CCL5,CCL8,CCR1,CCR3,CD70,CIITA,CLCF1,COL11A1,COL15A1,COL17A1,COL1A1,COL26A1,COL28A1,COL4A4,COL4A5,COL4A6,COL5A2,COL6A2,COL6A3,COL7A1,COL8A2,COL9A2,COL9A3,CX3CL1,CXCL1,CXCL10,CXCL12,CXCL14,CXCL16,CXCL2,CXCL3,CXCL5,CXCL8,CXCR4,DHX58,FTH1,GSDMD,GSDME,HLA-DOB,HLA-DQA1,HLA-DRA,HLA-DRB1,HLA-DRB5,IFIH1,IFNGR2,IL11,IL12RB1,IL15,IL18R1,IL1A,IL1B,IL1R1,IL1RAP,IL1RN,IL23A,IL6,IL6ST,IRF1,IRF7,IRF9,JAK2,LIF,MAPK11,MAPK3,MAPK4,MLKL,MYC,MYD88,NFKB1,NFKB2,NGFR,NLRC5,NOD1,NOD2,NOS2,PGF,PRDM1,RIGI,RYR2,SLC2A4,SLC2A5,SRGN,STAT1,STAT5A,STING1,STXBP2,TBK
Role of Hypercytokinemia/hyperchei	8,58E00	5,32E-01	5,745	CASP1,CCL2,CCL5,CXCL10,CXCL3,CXCL8,EIF2AK2,IFIT2,IFIT3,IL1A,IL1B,IL1RN,IL6,IRF7,IRF9,ISG15,ISG20,MX1,MYD88,NFKB1,NFKB2,OAS1,OAS2,OAS3,RIGI,RSAD2,S1PR1,STAT1,STAT2,TICAM1,TLR3,TLR4,TNF
Interferon Signaling	7,86E00	6,56E-01	3,578	BAK1,IFI35,IFI6,IFIT1,IFIT3,IFITM1,IFITM2,IFITM3,IFNGR2,IRF1,IRF9,ISG15,JAK2,MX1,OAS1,PSMB8,PTPN2,SOCS1,STAT1,STAT2,TAP1

Neuroinflammation Signaling Pathw	7,63E00	3,3E-01	4,900	ACVR2A,APH1B,B2M,BACE2,BIRC3,CALB1,CALB2,CASP1,CASP8,CCL2,CCL5,CD200,CD40,CFLAR,CREB3L4,CX3CL1,CXCL10,CXCL12,CXCL8,FAS,FZD1,GABBR1,GABBR2,GABRB3,GABRE,GABRG3,GABRP,GAD1,GAD2,GDNF,GRIA1,HLA-A,HLA-B,HLA-C,HLA-DOB,HLA-DQA1,HLA-DRA,HLA-DRB1,HLA-DRB5,HLA-E,HLA-F,HLA-G,ICAM1,IFNGR2,IKBKE,IL1B,IL1R1,IL34,IL6,IRAK2,IRF7,JAK2,JMJD7-PLA2G4B,MAPK11,MAPK3,MAPK4,MAPT,MFGE8,MR1,MYD88,NAIP,NFAT5,NFATC2,NFATC4,NFKB1,NFKB2,NOS2,PIK3CD,PIK3R1,PIK3R5,PLA2G4A,PLA2G4C,PLA2G6,PLCG2,PPP3CC,PTGS2,RAC2,REL,RELB,S100B,SLC1A2,SLC6A1,SNCA,STAT1,TBK1,TGFB2,TGFB3,TGFBR3,TICAM1,TICAM2,

Wound Healing Signaling Pathway	7,17E00	3,45E-01	0,918	ACTA2,ACVR2A,CCL5,CD70,CEBPB,CLCF1,COL11A1,COL15A1,COL17A1,COL1A1,COL26A1,COL28A1,COL4A4,COL4A5,COL4A6,COL5A2,COL6A2,COL6A3,COL7A1,COL8A2,COL9A2,COL9A3,CXCL8,FGFR2,FN1,IFNGR2,IL11,IL15,IL1A,IL1B,IL1R1,IL1RAP,IL1RN,IL6,ITGB4,JAK2,KRT17,LAMA1,LAMA2,LAMA3,LAMA5,LAMB3,LIF,MAP2K3,MAP2K5,MAPK3,MRAS,MST1,NFKB1,NFKB2,NFKBIA,NFKBIB,NFKBIE,NGFR,PDGFA,PDGFB,PGF,PRKCA,RRAS,SHC2,SHC3,SNAI2,SOS2,STAT1,TGFA,TGFB2,TGFB3,TGFBR3,TNF,TNFRSF1B,TNFSF10,TNFSF12,TNFSF13B,TNFSF9,VEGFC,VIM
Role of Pattern Recognition Recept	6,95E00	3,92E-01	3,651	C3,C3AR1,C5,C5AR1,CASP1,CCL5,CD70,CLCF1,CXCL8,EIF2AK2,EIF2S1,IFIH1,IL11,IL15,IL1A,IL1B,IL6,IRF7,LIF,MAPK3,MYD88,NFKB1,NFKB2,NOD1,NOD2,OAS1,OAS2,OAS3,PIK3CD,PIK3R1,PIK3R5,PLCG2,PRKCA,PRKCQ,PRKD1,PTX3,REL,RELB,RIGI,RIPK2,RNASEL,TGFB2,TGFB3,TICAM1,TLR3,TLR4,TNF,TNFSF10,TNFSF12,TNFSF13B,TNFSF9

IL-17A Signaling in Fibroblasts	6,66E00	4,44E-01	4,333	ACTA2,ARID5A,CCL2,CCL8,CEBPB,CEBPD,COL1A1,CXCL1,CXCL12,CXCL5,ELF3,FN1,IKBKE,IL17RC,IL1B,IL23A,IL6,JAK2,LOX,MAPK11,MAPK3,NFKB1,NFKB2,NFKBIA,NFKBIB,NFKBIE,NFKBIZ,P4HA1,P4HA2,PRKCA,REL,RELB,THY1,TNF,VCAM1,ZC3H12A
Crosstalk between Dendritic Cells a	6,45E00	4,43E-01	4,025	ACTA1,ACTA2,ACTB,ACTG1,ACTG2,CAMK2A,CD40,CD83,FAS,FSCN1,FSCN2,HLA-A,HLA-B,HLA-C,HLA-DRA,HLA-DRB1,HLA-DRB5,HLA-E,HLA-F,HLA-G,IL15,IL15RA,IL6,MICA,MICB,NECTIN2,NFKB1,NFKB2,REL,RELB,TLR3,TLR4,TNF,TNFRSF1B,TNFSF10
Tumor Microenvironment Pathway	6,36E00	3,57E-01	2,626	CCL2,CCND1,CD274,CFLAR,COL1A1,CSF1,CSF3,CXCL12,CXCL8,CXCR4,FAS,FGF19,FGF22,FN1,FOXO4,FOXO6,HGF,HLA-A,HLA-B,HLA-C,HLA-E,HLA-F,HLA-G,ICAM1,IDO1,IGF2,IL1B,IL6,ITGA5,ITGB3,JAK2,LGALS9,MAPK3,MMP14,MMP15,MMP16,MMP24,MMP25,MRAS,MYC,NFKB1,NFKB2,NOS2,PDCD1LG2,PDGFA,PDGFB,PGF,PIK3CD,PIK3R1,PIK3R5,PTGS2,REL,RELB,RRAS,SLC2A4,SPP1,TGFB2,TGFB3,TNF,VEGFC

IL-27 Signaling Pathway	6,25E00	3,9E-01	3,539	B2M,C5,C5AR1,CASP1,CD274,CD40,EBI3,ENTPD1,GADD45G,HLA-A,HLA-B,HLA-C,HLA-E,HLA-F,HLA-G,IFNGR2,IL1R1,IL1RAP,IL6ST,IRF1,JAK2,LPAR4,MAF,MAPK11,MR1,MYD88,NFKB1,NFKB2,NGFR,NOS2,PIK3CD,PIK3R1,PIK3R5,PTGS2,REL,RELB,RORA,RUNX1,STAT1,TLR3,TLR4,TNFRSF1B,TNFRSF9,TNFSF9,TRIM69,ULK1
Interleukin-10 signaling	5,98E00	5,23E-01	4,796	CCL2,CCL20,CCL5,CCR1,CSF1,CSF3,CXCL1,CXCL10,CXCL2,CXCL8,ICAM1,IL10RA,IL1A,IL1B,IL1R1,IL1RN,IL6,LIF,PTAFR,PTGS2,TIMP1,TNF,TNFRSF1B

Multiple Sclerosis Signaling Pathwa	5,69E00	3,33E-01	4,924	ASIC1,BID,C2,C3,C5,CAPN5,CAPN9,CASP1,CASP8,CD70,CLCF1,CXCL1,CXCL8,CYP27B1,DUSP1,FAS,HLA-A,HLA-B,HLA-C,HLA-DOB,HLA-DQA1,HLA-DRA,HLA-DRB1,HLA-DRB5,HLA-E,HLA-F,HLA-G,IFNGR2,IL11,IL15,IL17RC,IL1A,IL1B,IL23A,IL6,IL7R,IRF1,LIF,MAPK11,MAPK3,MGAT1,NFATC2,NFKB1,NFKB2,NLRP1,P2RX4,PARP10,PARP12,PARP14,PARP4,PARP8,PARP9,PLP1,PPARG,RNF213,STAT1,SUZ12,TGFB2,TGFB3,TLR3,TLR4,TNF,TNFSF10,TNFSF12,TNFSF13B,TNFSF9
Activation of IRF by Cytosolic Patter	4,99E00	4,62E-01	1,706	ADAR,CD40,DHX58,IFIH1,IFIT2,IKBKE,IL6,IRF7,IRF9,ISG15,NFKB1,NFKB2,NFKBIA,NFKBIB,NFKBIE,REL,RELB,RIGI,STAT1,STAT2,TBK1,TNF,TRAF3,ZBP1

Role of Osteoblasts in Rheumatoid	4,96E00	3,18E-01	2,605	ACVR2A,ALPL,APC,APC2,BMP7,CD70,CLCF1,COL1A1,CSNK1G1,CTSC,CTSH,CTSL,CTSO,CTSS,CXCL12,CXCL8,DLX5,FRZB,FZD1,FZD5,FZD9,IL11,IL15,IL1A,IL1B,IL6,IL6ST,JAK2,LIF,LRP1,MAPK3,MMP14,MMP15,MMP16,MMP24,MMP25,PGF,PIK3CD,PIK3R1,PIK3R5,PTGS2,RUNX2,SFRP4,SFRP5,SMAD9,SMURF1,SRC,STAT1,STAT2,STAT5A,TCF7L1,TGFB2,TGFB3,TNF,TNFSF10,TNFSF12,TNFSF13B,TNFSF9,VEGFC,WNT10A,WNT10B,WNT11,WNT2B,WNT3,WNT5A,WNT7B,WNT9B
Assembly of collagen fibrils and oth	4,9E00	4,41E-01	0,392	COL11A1,COL14A1,COL15A1,COL17A1,COL1A1,COL4A4,COL4A5,COL4A6,COL5A2,COL6A2,COL6A3,COL7A1,COL8A2,COL9A2,COL9A3,CTSL,CTSS,DST,ITGB4,LAMA3,LAMB3,LOX,LOXL3,PCOLCE,PXDN,TLL2

CGAS-STING Signaling Pathway	4,89E00	3,61E-01	3,812	ABCC1,ATP6V0E2,ATP6V1C2,ATP6V1G2,CASP1,CCL2,CCL20,CD274,CD70,CLCF1,CXCL8,ENPP1,GSDMD,ICAM1,IDO1,IKBKE,IL11,IL15,IL1A,IL1B,IL6,IRF1,LIF,MAP1LC3A,MAP1LC3C,NFKB1,NFKB2,NFKBIA,PCDH7,STAT1,STING1,TBK1,TCIRG1,TFAM,TGFB2,TGFB3,TNF,TNFSF10,TNFSF12,TNFSF13B,TNFSF9,TREX1,ZBP1
Systemic Lupus Erythematosus in I	4,71E00	3,03E-01	4,650	CCND1,CCND2,CCND3,CD40,CD70,CLCF1,CXCL8,FOXO4,FOXO6,GAB1,HCK,IFIH1,IFIT2,IFIT3,IFNGR2,IL11,IL15,IL1A,IL1B,IL6,IL6ST,INPPL1,IRF7,IRF9,ISG15,ISG20,JAK2,LIF,LYN,MAPK3,MRAS,MYC,MYD88,NFAT5,NFATC2,NFATC4,NFKB1,NFKB2,PAG1,PIK3AP1,PIK3CD,PIK3R1,PIK3R5,PIM2,PLAAT4,PLCG2,PPP3CC,PRKCA,PRKCQ,PRKD1,RAC2,RASGRP1,RASGRP3,REL,RELB,RRAS,SHC2,SHC3,SHF,SOS2,SRC,STAT1,STAT2,STING1,TBK1,TGFB2,TGFB3,TICAM1,TLR3,TNF,TNFSF10,TNFSF12,TNFSF13B,TNFSF9,TRAF1,TRAF3

Smooth Muscle Contraction	4,7E00	4,88E-01	-2,236	ACTA2,ACTG2,ALDH2,ANXA6,CACNA1H,CACNA1I,CALD1,DYSF,GUCY1A1,GUCY1A2,ITGA1,ITGB5,LMOD1,MYL6,MYL9,MYLK,PAK1,TPM1,TPM2,TPM4
Molecular Mechanisms of Cancer	4,68E00	2,51E-01	-0,560	ACVR2A,ADCY2,ADCY5,ADCY6,ADCY8,ADGRA2,ADGRB2,ADGRB3,ADGRE5,ADGRG1,ADGRL3,ADGRV1,ADORA2A,ADORA2B,ADRA1A,ADRA2B,ADRB1,APC,APC2,APH1B,ARHGEF10,ARHGEF16,ARHGEF17,ARHGEF4,ARHGEF6,BAK1,BBC3,BDKRB2,BID,BIRC3,BMP7,BMP8B,C3AR1,C5AR1,CAMK2A,CASP6,CASP7,CASP8,CCND1,CCND2,CCND3,CCR1,CCR3,CDC25A,CDC25B,CDC25C,CDKN2A,CDKN2B,CDKN2C,CELSR2,CFLAR,CHRM4,CNR1,CTNNA1,CTNND1,DHH,DRD4,E2F8,EDN2,EDNRB,F2RL1,FAS,FZD1,FZD5,FZD9,GAB1,GABBR1,GABBR2,GALR2,GHR,GIPR,GNAI1,GNAO1,GNAS,GNAZ,GNB4,GNG12,GNG2,GNG7,GPER1,GPR137B,GPR137C,GPR139,GPR141,GPR146,GPR157,GPR162,GPR176,GPR180,GP

Xenobiotic Metabolism AHR Signali	4,49E00	4,03E-01	-0,577	ABCG2,AHRR,ALDH1A3,ALDH1L2,ALDH2,ALDH3A1,ALDH3B1,ALDH4A1,ALDH5A1,ALDH6A1,CYP1B1,GSTA4,GSTM2,GSTM3,GSTM4,GSTO1,GSTO2,GSTT2/GSTT2B,HDAC5,HSP90AA1,HSP90B1,IL1A,IL1B,IL6,NFKB1,NFKB2,REL,RELB,TNF
Role of Chondrocytes in Rheumatoi	4,47E00	3,46E-01	3,920	ADAMTS4,ASIC1,CASP1,CASP8,CCL2,CEBPB,CEBPD,CEBPZ,CFLAR,CXCL12,CXCL8,CXCR4,FN1,IL17RC,IL18R1,IL1A,IL1B,IL1R1,IL1RAP,IL1RN,IL6,IL6ST,ITGA5,JAK2,LRP1,MAPK11,MAPK3,MLKL,MMP14,MMP15,MMP16,MMP24,MMP25,NFKB1,NFKB2,NGFR,NOS2,PGF,PPP3CC,PTGS2,RUNX2,TNF,TNFRSF1B,VEGFC
TWEAK Signaling	4,47E00	0.5	0,000	BID,BIRC3,CASP6,CASP7,CASP8,IKBKE,NAIP,NFKB1,NFKB2,NFKBIA,NFKBIB,NFKBIE,REL,RELB,TNFRSF25,TNFSF12,TRAF1,TRAF3
Death Receptor Signaling	4,41E00	3,72E-01	0,870	ACTA1,ACTA2,ACTB,ACTG1,ACTG2,BID,BIRC3,CASP6,CASP7,CASP8,CFLAR,FAS,IKBKE,NAIP,NFKB1,NFKB2,NFKBIA,NFKBIB,NFKBIE,PARP10,PARP12,PARP14,PARP4,PARP8,PARP9,REL,RELB,SPTAN1,TBK1,TNF,TNFRSF10A,TNFRSF1B,TNFRSF25,TNFSF10,TNFSF12

Macrophage Classical Activation Si	4,22E00	3,27E-01	5,000	CCL20,CCL5,CD40,CD70,CIITA,CLCF1,CXCL10,CXCL8,GBP2,GBP4,HLA-DOB,HLA-DQA1,HLA-DRA,HLA-DRB1,HLA-DRB5,IFNGR2,IL11,IL15,IL1A,IL1B,IL23A,IL6,IRF1,IRF9,JAK2,LIF,MAF,MYD88,NFKB1,NFKB2,NFKBIA,NFKBIB,NFKBIE,NOS2,PARP14,PARP9,PPARG,SOCS1,STAT1,STAT2,SUZ12,TGFB2,TGFB3,TICAM1,TLR4,TNF,TNFSF10,TNFSF12,TNFSF13B,TNFSF9
NOD1/2 Signaling Pathway	4,22E00	3,27E-01	3,960	BIRC3,CASP1,CCL2,CD70,CLCF1,CXCL8,ERBIN,HSP90AA1,HSP90B1,HSPA1A/HSPA1B,HSPA2,HSPA9,IKBKE,IL11,IL15,IL1A,IL1B,IL6,IRF7,LIF,MAP2K3,MAP3K8,MAPK11,MAPK3,MYD88,NFKB1,NFKB2,NFKBIA,NFKBIB,NFKBIE,NOD1,NOD2,NOS2,RBCK1,RIGI,RIPK2,SLC15A3,TAB3,TBK1,TGFB2,TGFB3,TLR3,TLR4,TNF,TNFAIP3,TNFSF10,TNFSF12,TNFSF13B,TNFSF9,TRAF3

Type I Diabetes Mellitus Signaling	4,13E00	3,41E-01	3,838	BID,CASP8,CPE,FAS,GAD1,GAD2,HLA-A,HLA-B,HLA-C,HLA-DOB,HLA-DQA1,HLA-DRA,HLA-DRB1,HLA-DRB5,HLA-E,HLA-F,HLA-G,HSPD1,IFNGR2,IKBKE,IL1B,IL1R1,IL1RAP,IRF1,JAK2,MAP2K3,MAPK11,MYD88,NFKB1,NFKB2,NFKBIA,NFKBIB,NFKBIE,NGFR,NOS2,PTPRN,REL,RELB,SOCS1,STAT1,TNF,TNFRSF1B
TREM1 Signaling	4,09E00	3,97E-01	4,200	CASP1,CCL2,CD40,CD83,CIITA,CXCL3,CXCL8,ICAM1,IL1B,IL6,ITGA5,JAK2,MAPK3,MYD88,NFKB1,NFKB2,NLRC5,NOD1,NOD2,PLCG2,REL,RELB,SIGIRR,STAT5A,TLR3,TLR4,TNF
TEC Kinase Signaling	4,07E00	3,08E-01	0,649	ACTA1,ACTA2,ACTB,ACTG1,ACTG2,FAS,GNAI1,GNAO1,GNAS,GNAZ,GNB4,GNG12,GNG2,GNG7,HCK,ITGA1,ITGA2B,ITGA4,ITGA5,ITGA7,ITGAE,ITGB3,ITGB4,ITGB5,ITGB7,JAK2,LYN,MRAS,NFKB1,NFKB2,PAK1,PAK6,PIK3CD,PIK3R1,PIK3R5,PLCG2,PRKCA,PRKCQ,PRKD1,PTK2B,RAC2,REL,RELB,RHOC,RHOU,RND1,RND2,SRC,STAT1,STAT2,STAT5A,TEC,TLR4,TNF,TNFRSF10A,TNFRSF25,TNFSF10,TNFSF12,VAV3,WAS

Pulmonary Fibrosis Idiopathic Signa	4,01E00	2,81E-01	0,000	ACTA1,ACTA2,ACTB,ACTG1,ACTG2,ACVR2A,BBC3,CAV1,CCN2,CCND1,COL11A1,COL15A1,COL17A1,COL1A1,COL26A1,COL28A1,COL4A4,COL4A5,COL4A6,COL5A2,COL6A2,COL6A3,COL7A1,COL8A2,COL9A2,COL9A3,CXCL12,EIF4EBP2,FGFR2,FGFR3,FGFR4,FN1,FOXO4,FOXO6,FZD1,FZD5,FZD9,GLI2,HES1,IL11,IL1B,IL6,IL6ST,ILK,JAK2,LPAR1,MAP2K3,MAPK11,MAPK3,MMP14,MMP15,MMP16,MMP24,MMP25,MRAS,NFKB1,NFKB2,PDGFA,PDGFB,PDGFRB,PIK3CD,PIK3R1,PIK3R5,PINK1,PMAIP1,PRKN,PTK2B,REL,RELB,RRAS,SOS2,SUZ12,TCF7L1,TERT,TGFA,TGFB2,TGFB3,TGFBR3,THBS1,VIM,WNT10A,WNT10B,WNT11,WNT2B,WNT3,WNT5A,WNT7B,WNT9B
Role of RIG1-like Receptors in Antiv	3,83E00	4,85E-01	2,714	CASP8,DHX58,IFIH1,IKBKE,IRF7,NFKB1,NFKB2,NFKBIA,NFKBIB,NFKBIE,REL,RELB,RIGI,TBK1,TRAF3,TRIM25

Cachexia Signaling Pathway	3,77E00	2,75E-01	3,545	ACVR2A,ADCY2,ADCY5,ADCY6,ADCY8,ADRB1,CAPN5,CAPN9,CASP1,CASP4,CASP6,CASP7,CASP8,CASQ1,CCL2,CD70,CEBPB,CEBPD,CLCF1,CXCL8,EIF2AK2,EIF2S1,FOXO4,FOXO6,GDF15,GNAS,GUCY1A1,HSP90AA1,HSP90B1,HSPA1A/HSPA1B,HSPA2,HSPA9,IFNGR2,IKBKE,IL11,IL15,IL1A,IL1B,IL1R1,IL1RAP,IL1RN,IL6,INHBA,INHBB,INHBE,IRS1,LIF,LPL,MALAT1,MAPK11,MAPK3,MYD88,NFKB1,NFKB2,NGFR,NOS2,PIK3CD,PIK3R1,PIK3R5,PPARG,PRKCA,PRKCQ,PRKD1,PSMA2,PSMA3,PSMA5,PSMA6,PSMB10,PSMB8,PSMB9,PSMC4,PSMD11,PSME1,PSME2,PSMF1,PTHLH,S1PR1,SERPINA1,SLC2A4,STAT1,STAT2,STAT5A,TGFB2,TGFB3,TGFBR3,TLR4,TNF,TNFRSF1B,TNFSF10,TNFSF12,TNFSF13B,TNFSF9
Elastic fibre formation	3,76E00	4,42E-01	-0,688	BMP7,EFEMP2,EMILIN3,FBLN2,FBLN5,FBN3,FN1,ITGA5,ITGB3,ITGB5,LOX,LOXL3,LTBP2,LTBP3,LTBP4,MFAP4,MFAP5,TGFB2,TGFB3
Integrin cell surface interactions	3,71E00	3,66E-01	0,000	CD47,COL1A1,COL4A4,COL4A5,COL4A6,COL5A2,COL6A2,COL6A3,COL7A1,COL8A2,COL9A2,COL9A3,FN1,ICAM1,ICAM4,ITGA1,ITGA2B,ITGA4,ITGA5,ITGA7,ITGAE,ITGB3,ITGB5,ITGB7,JAM2,LUM,SPP1,THBS1,VCAM1,VWF

IL-12 Signaling and Production in M	3,67E00	2,99E-01	2,840	APOE,APOL1,C3,CD40,CD47,CEBPB,COL1A1,DHX58,EBI3,ETS2,H3-3A/H3-3B,IFIH1,IKBKE,IL10RA,IL12RB1,IL23A,IL6ST,IRF1,JAK2,JMJD6,MAF,MAP2K3,MAP3K8,MAPK11,MAPK3,MST1,MYD88,NFAT5,NFATC2,NFATC4,NFKB1,NFKB2,NOD2,NOS2,PCYOX1,PIK3CD,PIK3R1,PIK3R5,POU2F2,PPARG,PRKCA,PRKCQ,PRKD1,PRLR,REL,RELB,RIGI,RIPK2,SERPINA1,STAT1,TGFB2,TGFB3,THBS1,TICAM2,TLR3,TLR4,TNF,TNFRSF11A,VDR,ZIC2
Extracellular matrix organization	3,67E00	3,43E-01	-1,000	BCAN,CEACAM1,COL11A1,COL1A1,COL4A4,COL4A5,COL4A6,COL5A2,COL6A2,COL6A3,COL7A1,COL9A2,COL9A3,FN1,ITGA1,ITGA2B,ITGA5,ITGA7,ITGB3,ITGB4,ITGB5,LAMA1,LAMA2,LAMA3,LAMA5,LAMB2,LAMB3,LUM,NCAM1,NCAN,NTN4,PDGFA,PDGFB,PTPRS,TGFB2,TGFB3

Antioxidant Action of Vitamin C	3,67E00	3,43E-01	-2,294	GLRX,GSTO1,GSTO2,IKBKE,JAK2,MAPK11,MAPK3,NFKB1,NFKB2,NFKBIA,NFKBIB,NFKBIE,NXN,PLA2G4A,PLA2G4C,PLA2G6,PLAAT2,PLAAT4,PLB1,PLCD1,PLCD3,PLCE1,PLCG2,PLCL2,PLD1,PLD2,PLD5,PLD6,REL,RELB,SELENOT,SLC2A4,SLC2A5,STAT5A,TNF,TXNDC2
Protein Kinase A Signaling	3,65E00	2,68E-01	-1,481	ADCY2,ADCY5,ADCY6,ADCY8,ADD2,ADD3,AKAP12,AKAP5,AKAP6,ATF1,CAMK2A,CDC14B,CDC25A,CDC25B,CDC25C,CREB3L4,DHH,DUSP1,DUSP16,DUSP2,DUSP5,DUSP7,DUSP9,EBI3,ENPP1,FLNA,FLNC,GDPD1,GNAI1,GNAS,GNB4,GNG12,GNG2,GNG7,GUCY1A1,H1-0,H3-3A/H3-3B,HHAT,ITPR1,ITPR3,MAPK3,MPPED2,MYH10,MYL6,MYL9,MYLK,MYLK3,NFAT5,NFATC2,NFATC4,NFKB1,NFKB2,NFKBIA,NFKBIB,NFKBIE,NGFR,PDE10A,PDE1A,PDE1C,PDE2A,PDE3A,PDE6D,PDE8B,PDE9A,PHKB,PHKG1,PLCD1,PLCD3,PLCE1,PLCG2,PLCL2,PLD6,PPP1R1B,PPP1R3C,PPP3CC,PRKCA,PRKCQ,PRKD1,PTCH1,PTGS2,PTK2B,PTPN12,PTPN2,PTPN21,PTPRA,PTPRD,PTPRF,PTPRG,PTPRM,PTPRN,PTPRR,PTP

Role of PKR in Interferon Induction	3,61E00	3,28E-01	1,667	BID,CASP1,CASP8,COLEC12,EIF2AK2,EIF2S1,FAS,HSP90AA1,HSP90B1,HSPA1A/HSPA1B,HSPA2,HSPA9,IFIH1,IFNGR2,IKBKE,IL1B,IRF1,IRF9,MAP2K3,MAPK11,MAPK3,MYD88,NFKB1,NFKB2,NFKBIA,NFKBIB,NFKBIE,NLRP1,PDGFA,PDGFB,PDGFRB,REL,RELB,RIGI,SCARA3,STAT1,STAT2,TLR3,TLR4,TNF,TRAF3
TNFR2 Signaling	3,61E00	4,84E-01	2,714	BIRC3,IKBKE,NAIP,NFKB1,NFKB2,NFKBIA,NFKBIB,NFKBIE,REL,RELB,TBK1,TNF,TNFAIP3,TNFRSF1B,TRAF1
OAS antiviral response	3,6E00	7,78E-01	1,890	FLNA,OAS1,OAS2,OAS3,OASL,RIGI,RNASEL

Role of Osteoclasts in Rheumatoid A	3,6E00	2,81E-01	1,522	ADAM21,ADAM33,ADAM8,ADAM9,BIRC3,CCND1,COL11A1,COL15A1,COL17A1,COL1A1,COL26A1,COL28A1,COL4A4,COL4A5,COL4A6,COL5A2,COL6A2,COL6A3,COL7A1,COL8A2,COL9A2,COL9A3,CREB3L4,CSF1,CTNNA1,FOXO4,FOXO6,FRZB,IKBKE,IL1B,IL1R1,IL1RAP,IL7,ITGA5,ITGB3,LCP2,MAP2K3,MAP2K5,MAPK11,MAPK3,MITF,MMP14,MMP15,MMP16,MMP24,MMP25,MRAS,NAIP,NFKB1,NFKBIA,NFKBIB,NFKBIE,NGFR,PIK3CD,PIK3R1,PIK3R5,PLCG2,PPP3CC,PTK2B,RAC2,RHOC,RHOU,RND1,RND2,RRAS,SFRP4,SFRP5,SHC2,SHC3,SOS2,SPP1,SRC,TEC,TLR4,TNF,TNFRSF11A,TNFRSF1B,VAV3
Arsenate Detoxification I (Glutaredo	3,57E00	1	1,000	AS3MT,GLRX2,GSTO1,LACC1,PNP

Hepatic Fibrosis Signaling Pathway	3,51E00	2,64E-01	2,014	ACTA2,ACVR2A,APC,APC2,CACNA1A,CACNA1C,CACNA1E,CACNA1S,CACNG4,CCL2,CCL5,CCN2,CCND1,CD40,CEBPB,CNR1,COL1A1,CREB3L4,CSNK1G1,CXCL8,FOXO4,FTH1,FZD1,FZD5,FZD9,GLI2,GNAI1,ICAM1,IKBKE,IL1A,IL1B,IL1R1,IL1RAP,IL1RN,IRAK2,ITGA1,ITGA2B,ITGA4,ITGA5,ITGA7,ITGAE,ITGB3,ITGB4,ITGB5,ITGB7,JAK2,KLF9,LRP1,MAP2K3,MAP2K5,MAPK11,MAPK3,MRAS,MYC,MYD88,MYL6,MYL9,MYLK,MYLK3,NFKB1,NFKB2,NFKBIA,NFKBIB,NFKBIE,NGFR,PDGFA,PDGFB,PDGFRB,PGF,PIK3CD,PIK3R1,PIK3R5,PLCG2,PPARG,PRKCA,PRKCQ,PRKD1,PTCH1,RAC2,REL,RELB,RHOC,RHOU,RND1,RND2,RRAS,SOS2,SPP1,TCF7L1,TGFB2,TGFB3,TGFBR3,TIMP1,TLR4,TNF,TNFRSF1B,VCAM1,VEGFC,WNT

Endothelin-1 Signaling	3,5E00	3,01E-01	0,420	ADCY2,ADCY5,ADCY6,ADCY8,CASP1,CASP4,CASP6,CASP7,CASP8,CASQ1,ECE1,EDNRB,GAB1,GNAI1,GNAO1,GNAS,GNAZ,GUCY1A1,GUCY1A2,ITPR1,ITPR3,MAPK11,MAPK3,MAPK4,MRAS,MYC,NOS2,PIK3CD,PIK3R1,PIK3R5,PLA2G4A,PLA2G4C,PLA2G6,PLAAT2,PLAAT4,PLB1,PLCD1,PLCD3,PLCE1,PLCG2,PLCL2,PLD1,PLD2,PLD5,PLD6,PRKCA,PRKCQ,PRKD1,PTGS1,PTGS2,RRAS,SHC2,SHC3,SHF,SRC

Cardiac Hypertrophy Signaling (Enh	3,49E00	2,55E-01	1,063	ACVR2A,ADCY2,ADCY5,ADCY6,ADCY8,ADRA1A,ADRA2B,ADRB1,CACNA1A,CACNA1C,CACNA1E,CACNA1S,CACNG4,CAMK2A,CD70,CLCF1,CXCL8,DIAPH2,DLG1,EDNRB,ENPP1,FGF19,FGF22,FGFR2,FGFR3,FGFR4,FZD1,FZD5,FZD9,GDPD1,GHR,GNAI1,GNAS,GNB4,GNG12,GNG2,GNG7,GUCY1A1,HDAC5,HDAC9,IKBKE,IL10RA,IL11,IL12RB1,IL15,IL15RA,IL17RC,IL17RD,IL17RE,IL18R1,IL1A,IL1B,IL1R1,IL20RA,IL4R,IL6,IL6ST,IL7R,ITGA1,ITGA2B,ITGA4,ITGA5,ITGA7,ITGAE,ITGB3,ITGB4,ITGB5,ITGB7,ITPR1,ITPR3,JAK2,LIF,MAP2K3,MAP2K5,MAP3K6,MAP3K8,MAPK11,MAPK3,MPPED2,MRAS,MYC,NFAT5,NFATC2,NFATC4,NFKB1,NFKB2,NGFR,PDE10A,PDE1A,PDE1C,PDE2A,PDE3A,PDE6D,PDE8B,PDE9A,PIK3CD,PIK3R1,
Role of JAK family kinases in IL-6-ty	3,49E00	3,7E-01	3,272	CCND1,CEBPD,CLCF1,CNTFR,HSP90AA1,HSP90B1,IL11,IL6,IL6ST,IRF1,JAK2,JUNB,LIF,MAPK11,MAPK3,MYC,OSMR,PGF,SERPINA1,SERPINA3,SOCS1,STAT1,STAT5A,TGFB2,TGFB3,TIMP1,VEGFC

IL-10 Signaling	3,45E00	3,15E-01	-3,429	BCL3,CCND1,CCR1,CREB3L4,DUSP1,HLA-A,HLA-B,HLA-C,HLA-DOB,HLA-DQA1,HLA-DRA,HLA-DRB1,HLA-DRB5,HLA-E,HLA-F,HLA-G,ICAM1,IFNGR2,IKBKE,IL10RA,IL1A,IL1B,IL1R1,IL1RAP,IL1RN,IL4R,IL6,IL6ST,MAF,MAP2K3,MAPK11,NFKB1,NFKB2,NFKBIA,NFKBIB,NFKBIE,NOS2,PBX1,PRDM1,REL,RELB,STAT1,STAT5A,TLR4,TNF
Aryl Hydrocarbon Receptor Signalin	3,45E00	3,15E-01	0,784	AHRR,ALDH1A3,ALDH1L2,ALDH2,ALDH3A1,ALDH3B1,ALDH4A1,ALDH5A1,ALDH6A1,CCND1,CCND2,CCND3,CDKN2A,CYP1B1,DCT,DHFR,ESR1,FAS,GSTA4,GSTM2,GSTM3,GSTM4,GSTO1,GSTO2,GSTT2/GSTT2B,HSP90AA1,HSP90B1,IL1A,IL1B,IL6,MAPK3,MDM2,MYC,NCOA7,NFIA,NFIB,NFKB1,NFKB2,REL,RELB,SRC,TGFB2,TGFB3,TGM2,TNF
Inflammasome pathway	3,38E00	5,5E-01	2,714	AIM2,CASP1,CASP8,IL1B,MYD88,NAIP,NFKB1,NFKB2,NLRP1,NOD2,TLR4

Collagen biosynthesis and modifyin	3,34E00	3,73E-01	0,200	ADAMTS14,ADAMTS2,COL11A1,COL14A1,COL15A1,COL17A1,COL1A1,COL26A1,COL28A1,COL4A4,COL4A5,COL4A6,COL5A2,COL6A2,COL6A3,COL7A1,COL8A2,COL9A2,COL9A3,COLGALT2,P4HA1,P4HA2,PCOLCE,PLOD3,TLL2
MIF-mediated Glucocorticoid Regul	3,24E00	4,55E-01	1,941	CD74,JMJD7-PLA2G4B,MAPK3,NFKB1,NFKB2,NFKBIA,NFKBIB,NFKBIE,PLA2G4A,PLA2G4C,PLA2G6,PTGS2,REL,RELB,TLR4
Interleukin-4 and Interleukin-13 sign	3,24E00	3,3E-01	2,058	ALOX5,CCL2,CCND1,CEBPD,CXCL8,FN1,FSCN1,HGF,HSP90AA1,ICAM1,IL1A,IL1B,IL23A,IL4R,IL6,JAK2,JUNB,LAMA5,LIF,MYC,NOS2,PIK3R1,PTGS2,RHOU,RORA,S1PR1,SAA1,SOCS1,STAT1,TIMP1,TNF,TNFRSF1B,VCAM1,VIM,ZEB1

Adrenergic Receptor Signaling Path	3,23E00	2,95E-01	-2,449	ADCY2,ADCY5,ADCY6,ADCY8,ADRA1A,ADRA2B,ADRB1,ATP6V0E2,ATP6V1C2,ATP6V1G2,CACNA1A,CACNA1C,CACNA1E,CACNA1H,CACNA1I,CACNA1S,CACNG4,CD70,CLCF1,CXCL8,GNAI1,GNAO1,GNAS,GUCY1A1,IL11,IL15,IL1A,IL1B,IL4I1,IL6,ITPR1,ITPR3,LIF,MAOB,PLCD1,PLCD3,PLCE1,PLCG2,PLCL2,PRKCA,PRKCQ,PRKD1,SLC18A2,SLC1A2,SMOX,TCIRG1,TGFB2,TGFB3,TH,TNF,TNFSF10,TNFSF12,TNFSF13B,TNFSF9
ILK Signaling	3,19E00	2,92E-01	-1,021	ACTA1,ACTA2,ACTB,ACTG1,ACTG2,ARHGEF6,CCND1,CREB3L4,FBLIM1,FLNA,FLNC,FN1,ILK,IRS1,IRS4,ITGB3,ITGB4,ITGB5,ITGB7,KRT18,MAPK3,MYC,MYH10,MYH14,MYH7B,MYL6,MYL9,MYO10,MYO5C,NFKB1,NFKB2,NOS2,PGF,PIK3CD,PIK3R1,PIK3R5,PPM1J,PPP2R1B,PPP2R2A,PPP2R2B,PPP2R3A,PTGS2,RAC2,REL,RELB,RHOC,RHOU,RICTOR,RND1,RND2,SNAI2,TESK1,TGFB1I1,TNF,VEGFC,VIM
Inhibition of Matrix Metalloproteases	3,03E00	4,52E-01	0,302	A2M,LRP1,MMP14,MMP15,MMP16,MMP24,MMP25,SDC1,SDC2,TFPI2,TIMP1,TIMP2,TIMP3,TIMP4

Interleukin-1 processing	2,99E00	7,5E-01	2,449	CASP1,GSDMD,IL1A,IL1B,NFKB1,NFKB2
Dilated Cardiomyopathy Signaling F	2,95E00	3,01E-01	0,186	ABCC9,ACTA1,ACTA2,ACTB,ACTG1,ACTG2,ADCY2,ADCY5,ADCY6,ADCY8,ADRB1,CACNA1A,CACNA1C,CACNA1E,CACNA1H,CACNA1I,CACNA1S,CACNG4,CAMK2A,CNN1,DES,DSC2,DSG2,GAB1,GNAS,GUCY1A1,ILK,ITPR1,ITPR3,MAPK11,MAPK3,MYH10,MYH14,MYH7B,MYL6,MYL9,MYO10,MYO5C,PDE2A,PDE3A,RBM20,RYR2,TNNC1,TPM1
Acute Phase Response Signaling	2,94E00	2,92E-01	3,000	A2M,C1R,C1S,C2,C3,C4A/C4B,C5,CEBPB,CFB,CP,ECSIT,FN1,IKBKE,IL1A,IL1B,IL1R1,IL1RAP,IL1RN,IL6,IL6ST,JAK2,MAP2K3,MAPK11,MAPK3,MRAS,MYD88,NFKB1,NFKB2,NFKBIA,NFKBIB,NFKBIE,NGFR,OSMR,PIK3CD,PIK3R1,RBP1,REL,RELB,RRAS,SAA1,SAA2,SERPINA1,SERPINA3,SERPINF1,SERPING1,SOCS1,SOS2,TNF,TNFRSF1B,VWF

Macrophage Alternative Activation S	2,93E00	2,93E-01	1,857	ADORA2A,ADORA2B,CEBPB,CIITA,CREB3L4,CSF1,DUSP1,H3-3A/H3-3B,HLA-DOB,HLA-DQA1,HLA-DRA,HLA-DRB1,HLA-DRB5,IL10RA,IL1A,IL1B,IL1RN,IL4R,IL6,IRS1,JAK2,KLF4,LPL,MAF,MAPK11,MAPK3,MYC,NFKB1,NFKB2,NFKBIA,NFKBIB,NFKBIE,NOS2,NR1H4,PIK3CD,PIK3R1,PIK3R5,PPARG,REL,RELB,SOCS1,STAT1,TGFB2,TGFB3,THBS1,TLR4,TNF,TSC22D3,ZC3H12A
Complement System	2,92E00	4,29E-01	0,000	C1QBP,C1R,C1S,C2,C3,C3AR1,C4A/C4B,C5,C5AR1,CD46,CD55,CFB,CFH,CFI,SERPING1
HMGB1 Signaling	2,89E00	2,98E-01	2,121	CCL2,CD70,CLCF1,CXCL8,ICAM1,IFNGR2,IL11,IL15,IL1A,IL1B,IL1R1,IL6,KAT2A,KAT6B,KAT7,LIF,MAP2K3,MAP2K5,MAPK11,MAPK3,MRAS,NFKB1,NFKB2,NGFR,PIK3CD,PIK3R1,PIK3R5,RAC2,REL,RELB,RHOC,RHOU,RND1,RND2,RRAS,TGFB2,TGFB3,TLR4,TNF,TNFRSF1B,TNFSF10,TNFSF12,TNFSF13B,TNFSF9,VCAM1

Dendritic Cell Maturation	2,87E00	2,86E-01	4,627	B2M,CD40,CD83,COL1A1,CREB3L4,FSCN1,FSCN2,HLA-A,HLA-B,HLA-C,HLA-DOB,HLA-DQA1,HLA-DRA,HLA-DRB1,HLA-DRB5,HLA-E,HLA-F,HLA-G,ICAM1,IKBKE,IL15,IL1A,IL1B,IL1RN,IL23A,IL32,IL6,JAK2,MAPK11,MAPK3,MR1,MYD88,NFKB1,NFKB2,NFKBIA,NFKBIB,NFKBIE,NGFR,PIK3CD,PIK3R1,PIK3R5,PLCD1,PLCD3,PLCE1,PLCG2,PLCL2,REL,RELB,STAT1,STAT2,TLR3,TLR4,TNF,TNFRSF1B
Erythropoietin Signaling Pathway	2,84E00	2,95E-01	-0,926	BIRC3,CCND1,CCND2,CD70,CLCF1,CXCL8,HBA1/HBA2,IL11,IL15,IL1A,IL1B,IL6,ITPR1,ITPR3,JAK2,LIF,MAPK3,MDM2,MRAS,NFKB1,NFKB2,NFKBIA,NFKBIB,NFKBIE,PIK3CD,PIK3R1,PIK3R5,PRKCA,PRKCQ,PRKD1,RAC2,REL,RELB,RRAS,SHC2,SHC3,SOS2,SRC,STAT5A,TGFB2,TGFB3,TNF,TNFSF10,TNFSF12,TNFSF13B,TNFSF9
Role of MAPK Signaling in Inhibiting	2,82E00	3,47E-01	2,600	CCL2,CCL5,CXCL10,CXCL8,EIF2AK2,IL1B,IL6,MAP2K3,MAPK11,MAPK3,MYD88,NFKB1,NFKB2,NFKBIA,NFKBIB,NFKBIE,PLA2G4A,PLA2G4C,PLA2G6,PLAAT2,PLAAT4,PLB1,PTGS2,TLR4,TNF

WNT/β-catenin Signaling	2,81E00	2,9E-01	0,309	ACVR2A,APC,APC2,APPL2,AXIN2,CCND1,CDKN2A,CSNK1G1,DKKL1,FRAT1,FRZB,FZD1,FZD5,FZD9,GNAO1,ILK,KREMEN2,LRP1,MDM2,MYC,PPM1J,PPP2R1B,PPP2R2A,PPP2R2B,PPP2R3A,SFRP4,SFRP5,SOX1,SOX13,SOX4,SOX6,SOX7,SOX8,SOX9,SRC,TCF7L1,TGFB2,TGFB3,TGFBR3,TLE3,UBD,WNT10A,WNT10B,WNT11,WNT2B,WNT3,WNT5A,WNT7B,WNT9B
RAF/MAP kinase cascade	2,8E00	2,71E-01	0,970	ACTB,ACTG1,ANGPT1,BTC,CAMK2A,DLG1,DLG2,DUSP1,DUSP16,DUSP2,DUSP5,DUSP7,DUSP9,ERBB4,FGF19,FGF22,FGFR2,FGFR3,FGFR4,FLT3LG,FN1,FRS3,GDNF,HGF,IL17RD,IRS1,ITGA2B,ITGB3,JAK2,KBTBD7,KSR1,LRRC7,MAPK3,MET,MRAS,NCAM1,NRG2,PDGFA,PDGFB,PDGFRB,PEA15,PIK3R1,PPP2R1B,PSMA2,PSMA3,PSMA5,PSMA6,PSMB10,PSMB8,PSMB9,PSMC4,PSMD11,PSME1,PSME2,PSMF1,PTPRA,RASA4,RASGRP1,RASGRP3,RET,SHC2,SHC3,SPRED3,SPTAN1,SPTBN5,SRC,TEK,TGFA,VWF

TNFR1 Signaling	2,78E00	3,8E-01	1,500	BID,BIRC3,CASP6,CASP7,CASP8,IKBKE,MAP4K2,NAIP,NFKB1,NFKB2,NFKBIA,NFKBIB,NFKBIE,PAK1,PAK6,REL,RELB,TNF,TNFAIP3
Autism Signaling Pathway	2,76E00	2,65E-01	2,393	ALDH1A3,ALDH1L2,ALDH2,ALDH3A1,ALDH3B1,ALDH4A1,ALDH5A1,ALDH6A1,APC,B2M,BCKDHB,BCYRN1,CACNA1A,CACNA1C,CACNA1E,CACNA1S,CACNG4,CAMK2A,CAMKK1,CCL2,CD70,CLCF1,CXCL8,FMR1,FOLR1,FZD1,FZD5,FZD9,GLI2,GRM8,HGF,HLA-A,HLA-B,HLA-C,HLA-E,HLA-F,HLA-G,HPRT1,IGF2,IL11,IL15,IL1A,IL1B,IL1RN,IL6,L2HGDH,LIF,MAPK3,MET,MR1,MRAS,NLGN3,NTRK2,PIK3CD,PIK3R1,PIK3R5,PPM1K,PTCH1,RRAS,SCN1A,SOS2,TCF7L1,TGFB2,TGFB3,TNF,TNFSF10,TNFSF12,TNFSF13B,TNFSF9,WNT10A,WNT10B,WNT11,WNT2B,WNT3,WNT5A,WNT7B,WNT9B
RAF-independent MAPK1/3 activati	2,73E00	4,78E-01	2,714	DUSP1,DUSP16,DUSP2,DUSP5,DUSP7,DUSP9,IL6,IL6ST,JAK2,MAPK3,PEA15

G alpha (i) signalling events	2,7E00	2,7E-01	0,000	ADCY2,ADCY5,ADCY6,ADCY8,ADRA2B,APLN,BDKRB2,C3,C3AR1,C5,C5AR1,CCL20,CCL5,CCR1,CCR3,CHRM4,CNR1,CORT,CX3CL1,CXCL1,CXCL10,CXCL12,CXCL16,CXCL2,CXCL3,CXCL5,CXCL8,CXCR4,DRD4,GABBR1,GABBR2,GAL,GALR2,GNAI1,GNAS,GNAZ,GNB4,GNG12,GNG2,GNG7,GPER1,GPSM1,GPSM2,GRM8,LPAR1,LPAR2,LPAR3,MCHR1,NMU,NPB,NPW,OPRL1,PSAP,RGR,RGS11,RGS14,RGS16,RGS20,RGS3,RGS5,RGS6,SAA1,SRC,SSTR2,SSTR3,TAS2R20
Putrescine Degradation III	2,7E00	5,29E-01	-1,000	ALDH1A3,ALDH2,ALDH3A1,ALDH3B1,ALDH4A1,IL4I1,MAOB,SAT2,SMOX
CD40 Signaling	2,66E00	3,48E-01	1,414	ATF1,CD40,ICAM1,IKBKE,MAP2K3,MAP2K5,MAPK11,MAPK3,NFKB1,NFKB2,NFKBIA,NFKBIB,NFKBIE,PIK3CD,PIK3R1,PIK3R5,PTGS1,PTGS2,REL,RELB,TNFAIP3,TRAF1,TRAF3
TAK1-dependent IKK and NF-kapp	2,64E00	3,86E-01	3,638	ALPK1,CASP8,IRAK2,N4BP1,NFKB1,NFKB2,NFKBIA,NFKBIB,NLRC5,NOD1,NOD2,RIPK2,S100B,SAA1,TAB3,TIFA,USP18

Collagen chain trimerization	2,64E00	3,86E-01	-0,728	COL11A1,COL14A1,COL15A1,COL17A1,COL1A1,COL26A1,COL28A1,COL4A4,COL4A5,COL4A6,COL5A2,COL6A2,COL6A3,COL7A1,COL8A2,COL9A2,COL9A3
iNOS Signaling	2,64E00	3,86E-01	3,606	IFNGR2,IKBKE,IRAK2,IRF1,JAK2,MAPK11,MYD88,NFKB1,NFKB2,NFKBIA,NFKBIB,NFKBIE,NOS2,REL,RELB,STAT1,TLR4
GP6 Signaling Pathway	2,62E00	3,05E-01	0,845	COL11A1,COL15A1,COL17A1,COL1A1,COL26A1,COL28A1,COL4A4,COL4A5,COL4A6,COL5A2,COL6A2,COL6A3,COL7A1,COL8A2,COL9A2,COL9A3,ITGA2B,ITGB3,ITPR1,LAMA1,LAMA2,LAMA3,LAMA5,LAMB2,LAMB3,LCP2,LYN,PIK3CD,PIK3R1,PIK3R5,PLCG2,PRKCA,PRKCQ,PRKD1,SCHIP1,VAV3
Cell junction organization	2,6E00	3,22E-01	-2,043	ACTB,ACTG1,ARHGEF6,CADM2,CADM3,CDH24,CDH8,CLDN1,CLDN5,COL17A1,CTNNA1,CTNND1,DST,FBLIM1,FLNA,FLNC,ILK,ITGB4,LAMA3,LAMB3,NECTIN2,NECTIN3,PALS1,PARD6B,PARD6G,PATJ,PVR,SDK2,TESK1

Osteoarthritis Pathway	2,6E00	2,73E-01	3,647	ADAMTS4,ALPL,CASP1,CASP4,CASP6,CASP7,CASP8,CASQ1,CEBPB,CREB3L4,CTNNA1,CXCL8,DLX5,ELF3,FGFR3,FN1,FRZB,FZD1,FZD5,FZD9,GLI2,GREM1,HES1,IL1B,IL1R1,IL1RAP,ITGA1,ITGA2B,ITGA4,ITGA5,ITGA7,ITGAE,ITGB3,ITGB4,ITGB5,ITGB7,LRP1,MTF1,NAMPT,NFKB1,NFKB2,NOS2,PGF,PPARG,PTCH1,PTGS2,PTHLH,REL,RELB,RUNX2,SDC4,SMAD9,SOX9,SPP1,TCF7L1,TIMP3,TLR4,TNF,TNFRSF1B,VEGFC
GP1b-IX-V activation signalling	2,59E00	6,67E-01	0,000	COL1A1,FLNA,GP1BA,PIK3R1,SRC,VWF
Syndecan interactions	2,59E00	4,44E-01	-1,155	COL1A1,COL5A2,FN1,ITGB3,ITGB4,ITGB5,PRKCA,SDC1,SDC2,SDC3,SDC4,THBS1
MIF Regulation of Innate Immunity	2,56E00	3,9E-01	2,138	CD74,JMJD7-PLA2G4B,MAPK3,NFKB1,NFKB2,NFKBIA,NFKBIB,NFKBIE,NOS2,PLA2G4A,PLA2G4C,PLA2G6,PTGS2,REL,RELB,TLR4

Activin Inhibin Signaling Pathway	2,55E00	2,77E-01	1,633	ACVR2A,CCN2,CCND2,CEBPB,COL1A1,CXCR4,EGLN3,FOSB,FOSL1,FST,GATA2,IKBKE,IL11,IL1A,IL1B,IL1R1,IL1RAP,IL1RN,IL6,INHBA,INHBB,LIMK2,MAF,MAP2K3,MAP2K5,MAPK11,MAPK3,MAPK4,MAX,MDM2,MYD88,NFKB1,NFKB2,NFKBIA,NGFR,PAX5,PAX8,PIK3CD,PIK3R1,PIK3R5,PMEPA1,PPARG,PTGS2,SMAD9,SNAI2,TCF7L1,TGFB2,TGFB3,TGFBR3,TLR3,TLR4,TNF,TNFRSF1B,TNFSF13B
Human Embryonic Stem Cell Plurip	2,55E00	2,77E-01	0,272	ACVR2A,APC,APC2,BMP7,BMP8B,DUSP9,ESRRB,FGFR2,FGFR3,FGFR4,FUT4,FZD1,FZD5,FZD9,HSP90AA1,HSP90B1,ID1,ID2,ID3,INHBA,JAK2,KLF2,KLF4,LEFTY2,LIF,MAP2K3,MAP2K5,MRAS,MYC,NTRK2,PDGFA,PDGFB,PDGFRB,PIK3CD,PIK3R1,PIK3R5,PRKCA,PRKCQ,PRKD1,RRAS,SMAD9,SOS2,TERT,TFCP2L1,TGFB2,TGFB3,WNT10A,WNT10B,WNT11,WNT2B,WNT3,WNT5A,WNT7B,WNT9B
Collagen degradation	2,52E00	3,57E-01	-0,894	ADAM9,COL11A1,COL14A1,COL15A1,COL17A1,COL1A1,COL26A1,COL4A4,COL4A5,COL4A6,COL5A2,COL6A2,COL6A3,COL7A1,COL8A2,COL9A2,COL9A3,CTSL,MMP14,MMP15

Pyroptosis Signaling Pathway	2,52E00	3,22E-01	3,780	AIM2,CASP1,CASP4,CASP8,GBP1,GBP2,GBP3,GBP4,GBP5,GSDMD,GSDME,IL1A,IL1B,IL1R1,IRF2,MAPK11,MEFV,NAIP,NFKB1,NFKB2,NGFR,NLRP1,TLR3,TLR4,TNF,TNFRSF1B,TRAF3,TXNIP
IL-1 Signaling	2,52E00	3,16E-01	2,840	ADCY2,ADCY5,ADCY6,ADCY8,ECSIT,GNAI1,GNAO1,GNAS,GNAZ,GNB4,GNG12,GNG2,GNG7,GUCY1A1,IKBKE,IL1A,IL1R1,IL1RAP,IRAK2,MAP2K3,MAPK11,MRAS,MYD88,NFKB1,NFKB2,NFKBIA,NFKBIB,NFKBIE,REL,RELB
Adrenomedullin signaling pathway	2,52E00	2,78E-01	0,745	ADCY2,ADCY5,ADCY6,ADCY8,C3,CEBPB,CFH,GAD1,GNAS,GUCY1A1,GUCY1A2,IL1A,IL1B,IL1RN,ITPR1,ITPR3,KCNH2,KCNN3,MAP2K3,MAP2K5,MAPK11,MAPK3,MAPK4,MATK,MAX,MRAS,MYLK,MYLK3,NFKB1,NFKB2,NPR3,PIK3CD,PIK3R1,PIK3R5,PLCD1,PLCD3,PLCE1,PLCG2,PLCL2,PPARG,PRKG1,PTK2B,REL,RELB,RRAS,SHC2,SHC3,SHF,SMARCC2,SOS2,TFAP2C,TNF

Gap Junction Signaling	2,5E00	2,58E-01	0,342	ACTA1,ACTA2,ACTB,ACTG1,ACTG2,ADCY2,ADCY5,ADCY6,ADCY8,ADRB1,CASP7,CAV1,CCND1,CREB3L4,CSNK1G1,DBN1,DLG1,GAD1,GJD3,GNAI1,GNAS,GRIA1,GRIK1,GRIK3,GUCY1A1,GUCY1A2,IL1B,ITPR1,ITPR3,JMJD7-PLA2G4B,KCNH2,KCNN3,LPAR1,MAP2K5,MAPK3,MRAS,MYC,NFKB1,NFKB2,NGFR,NPR3,OXTR,P2RY1,P2RY11,P2RY2,PDGFRB,PIK3CD,PIK3R1,PIK3R5,PLA2G4A,PLA2G4C,PLA2G6,PLCD1,PLCD3,PLCE1,PLCG2,PLCL2,PPP3CC,PRKCA,PRKCQ,PRKD1,PRKG1,PTPRM,REL,RELB,RRAS,RUNX2,SMARCC2,SOS2,SRC,TCF7L1,TLR4,TNF,TNFRSF1B,TUBA1A,TUBB2B,TUBB3,TUBB4A,YBX3
Semaphorin interactions	2,48E00	3,44E-01	-2,132	CDK5R1,CRMP1,DPYSL4,HSP90AA1,ITGA1,LIMK2,MET,MYH10,MYH14,MYL6,MYL9,PAK1,PLXNA3,PLXNA4,PLXNB3,RHOC,RND1,RRAS,SEMA3A,SEMA4A,SEMA5A,SEMA6D

PI3K/AKT Signaling	2,45E00	2,74E-01	2,414	CCND1,GAB1,GDF15,GHR,HSP90AA1,HSP90B1,IKBKE,IL10RA,IL12RB1,IL15RA,IL17RC,IL17RD,IL17RE,IL18R1,IL1R1,IL20RA,IL4R,IL6ST,IL7R,ILK,INPPL1,ITGA1,ITGA2B,ITGA4,ITGA5,ITGA7,ITGAE,ITGB3,ITGB4,ITGB5,ITGB7,JAK2,MAP3K8,MAPK3,MAPK8IP1,MDM2,MRAS,NFKB1,NFKB2,NFKBIA,NFKBIB,NFKBIE,PIK3CD,PIK3R1,PPM1J,PPP2R1B,PPP2R2A,PPP2R2B,PPP2R3A,PTGS2,REL,RELB,RRAS,SOS2
ISGylation Signaling Pathway	2,44E00	3,18E-01	3,780	CCND1,CHMP5,DTX3L,EIF2AK2,HERC5,IFIH1,IL6,IRF1,IRF2,IRF7,IRF9,ISG15,NFAT5,NFKB1,NFKB2,PRKN,RIGI,SQSTM1,STAT1,STAT2,STING1,TBK1,TLR3,TLR4,TRIM25,UBA7,UBE2L6,USP18
Cytosolic sensors of pathogen-asso	2,4E00	3,7E-01	4,123	AIM2,DTX4,IFI16,IRF7,MYD88,NFKB1,NFKB2,NFKBIA,NFKBIB,STING1,TBK1,TICAM1,TLR3,TREX1,TRIM21,TRIM56,ZBP1

HOTAIR Regulatory Pathway	2,37E00	2,86E-01	0,160	AR,COL1A1,DNMT3B,ESR1,H3-3A/H3-3B,ICAM1,IRF1,JAM2,MDM2,MET,MMP14,MMP15,MMP16,MMP24,MMP25,MYC,NFKB1,NFKB2,NFKBIA,PCDH10,PCDHB5,PIK3CD,PIK3R1,PIK3R5,REL,RELB,RHOC,SNAI2,SPP1,SUZ12,TCF7L1,TLR4,TWIST2,VIM,WNT10A,WNT10B,WNT11,WNT2B,WNT3,WNT5A,WNT7B,WNT9B
FAT10 Cancer Signaling Pathway	2,37E00	3,6E-01	2,673	ACVR2A,CXCR4,IKBKE,IL6,NFKB1,NFKB2,NFKBIA,NFKBIB,NFKBIE,NGFR,REL,RELB,TGFB2,TGFB3,TGFBR3,TNF,TNFRSF1B,UBD
NGF-stimulated transcription	2,36E00	3,85E-01	0,535	ARC,ASCL1,ATF1,CDK5R1,EGR2,EGR3,FOSB,FOSL1,ID1,ID2,ID3,JUNB,RRAD,TRIB1,VGF

Signaling by Rho Family GTPases	2,34E00	2,61E-01	-1,857	ACTA1,ACTA2,ACTB,ACTG1,ACTG2,ARHGEF10,ARHGEF16,ARHGEF17,ARHGEF4,ARHGEF6,CDC42EP1,CDC42EP2,CDH22,CDH24,CDH8,DES,GFAP,GNAI1,GNAO1,GNAS,GNAZ,GNB4,GNG12,GNG2,GNG7,ITGA1,ITGA2B,ITGA4,ITGA5,ITGA7,ITGAE,ITGB3,ITGB4,ITGB5,ITGB7,LIMK2,MAPK3,MRAS,MYL6,MYL9,MYLK,NFKB1,NFKB2,PAK1,PAK6,PIK3CD,PIK3R1,PIK3R5,PIP5KL1,PLD1,PTK2B,RAC2,REL,RELB,RHOC,RHOU,RND1,RND2,SEPTIN1,SEPTIN11,SEPTIN3,SEPTIN4,SEPTIN5,SEPTIN7,SEPTIN8,STMN1,VIM,WAS
Role of JAK1, JAK2 and TYK2 in In	2,32E00	4,55E-01	0,447	IFNGR2,JAK2,NFKB1,NFKB2,PTPN2,REL,RELB,SOCS1,STAT1,STAT2

S100 Family Signaling Pathway	2,32E00	2,33E-01	1,718	ABCC1,ADGRA2,ADGRB2,ADGRB3,ADGRE5,ADGRG1,ADGRL3,ADGRV1,ADORA2A,ADORA2B,ADRA1A,ADRA2B,ADRB1,AHNAK,BCL3,BDKRB2,BTC,C3AR1,C5AR1,CACNA1A,CACNA1C,CACNA1E,CACNA1H,CACNA1I,CACNA1S,CACNG4,CAMK2A,CASP7,CCL20,CCR1,CCR3,CELSR2,CHRM4,CNR1,CREB3L4,CXCL8,DLC1,DRD4,EDNRB,ESR1,F2RL1,FGFR2,FGFR3,FGFR4,FZD1,FZD5,FZD9,GABBR1,GABBR2,GALR2,GIPR,GPER1,GPR137B,GPR137C,GPR139,GPR141,GPR146,GPR157,GPR162,GPR176,GPR180,GPR27,GPR63,GPR88,GRM8,GRPR,HRH2,IL10RA,IL1B,IL23A,IL6,ILK,IRAK2,ITPR1,ITPR3,JMJD7-PLA2G4B,KISS1R,LGR4,LPAR1,LPAR2,LPAR3,LPAR4,LTB4R,LTB4R2,MAP2K3,MAP2K5,MAP3K8,MAPK11,
Type II Diabetes Mellitus Signaling	2,31E00	2,84E-01	1,706	ACSBG1,ACSF2,ACSL4,ACSL5,CACNA1A,CACNA1C,CACNA1E,CACNA1H,CACNA1I,CACNA1S,CACNG4,CEBPB,GCK,IKBKE,IRS1,IRS4,ITPR1,ITPR3,MAPK3,NFKB1,NFKB2,NFKBIA,NFKBIB,NFKBIE,NGFR,PIK3CD,PIK3R1,PIK3R5,PPARG,PRKCA,PRKCQ,PRKD1,REL,RELB,SLC27A2,SLC27A6,SLC2A4,SMPD1,SMPD3,SOCS1,TNF,TNFRSF1B

Toll-like Receptor Signaling	2,29E00	3,24E-01	2,840	ECSIT,EIF2AK2,IL1A,IL1B,IL1RN,IRAK2,MAP2K3,MAPK11,MYD88,NFKB1,NFKB2,NFKBIA,PPARA,REL,RELB,SIGIRR,TICAM1,TICAM2,TLR3,TLR4,TNF,TNFAIP3,TRAF1,UBD
Immunogenic Cell Death Signaling	2,29E00	3,21E-01	3,400	BAK1,CASP1,CASP8,CFLAR,CXCL10,DDIT3,HSP90AA1,HSP90B1,HSPA1A/HSPA1B,HSPA2,HSPA9,IFNGR2,IL1B,IL6,LRP1,MLKL,NFKB1,NFKB2,NGFR,P2RY2,STING1,TLR3,TLR4,TNF,TNFRSF1B
BAG2 Signaling Pathway	2,29E00	3,17E-01	-1,291	HSP90AA1,HSPA1A/HSPA1B,HSPA2,HSPA9,MAPK3,MAPT,MDM2,MYC,NFKB1,NFKB2,PINK1,PRKN,PSMA2,PSMA3,PSMA5,PSMA6,PSMB10,PSMB8,PSMB9,PSMC4,PSMD11,PSME1,PSME2,PSMF1,REL,RELB
Tryptophan Degradation X (Mamma	2,28E00	4,74E-01	-0,333	ALDH1A3,ALDH2,ALDH3A1,ALDH3B1,ALDH4A1,IL4I1,MAOB,RDH14,SMOX

CDX Gastrointestinal Cancer Signa	2,26E00	2,74E-01	-2,064	AXIN2,BMP7,BMP8B,CCND1,CD70,CLCF1,CXCL8,EFNB1,FZD1,FZD5,FZD9,GATA6,IKBKE,IL11,IL15,IL1A,IL1B,IL6,JAK2,KLF5,LIF,MAPK11,MAPK3,NFKB1,NFKB2,NR1H4,PIK3CD,PIK3R1,PIK3R5,PTGS2,RAC2,REL,RELB,TCF7L1,TGFB2,TGFB3,TNF,TNFSF10,TNFSF12,TNFSF13B,TNFSF9,WNT10A,WNT10B,WNT11,WNT2B,WNT3,WNT5A,WNT7B,WNT9B
Caspase activation via Death Recep	2,25E00	0.5	2,828	CASP8,CFLAR,FAS,TICAM1,TICAM2,TLR4,TNFRSF10A,TNFSF10
O-linked glycosylation	2,25E00	2,99E-01	0,354	ADAMTS12,ADAMTS13,ADAMTS14,ADAMTS15,ADAMTS2,ADAMTS4,ADAMTS7,ADAMTS8,ADAMTSL3,ADAMTSL4,B3GLCT,B3GNT2,B3GNT7,B4GALT5,CFP,GALNT15,GALNT18,GALNT5,GCNT1,LARGE2,MUC16,POMK,SEMA5A,SEMA5B,SPON1,SPON2,ST3GAL4,ST6GALNAC2,ST6GALNAC3,THBS1,THSD1,THSD4
Post-translational protein phosphory	2,23E00	3,03E-01	0,730	AMBN,ANO8,APOE,APOL1,C3,C4A/C4B,CCN1,CHRDL1,CP,CSF1,CST3,EVA1A,FN1,FSTL1,HSP90B1,IGFBP4,IL6,LAMB2,MFGE8,MGAT4A,MXRA8,PRSS23,SCG2,SDC2,SERPINA1,SHISA5,SPP1,STC2,TIMP1,VGF

Glutamate Degradation III (via 4-am	2,23E00	0.8	-2,000	ABAT,ALDH5A1,GAD1,GAD2
Semaphorin Neuronal Repulsive Sig	2,22E00	2,84E-01	-2,000	BCAN,CRMP1,DPYSL4,FARP1,GUCY1A1,GUCY1A2,ITGA1,ITGA2B,ITGA4,ITGA5,ITGA7,ITGAE,ITGB3,ITGB4,ITGB5,ITGB7,LIMK2,MAP2K3,MAP2K5,MAPT,MICAL1,MYL6,MYL9,NCAN,NRP2,PAK1,PAK6,PIK3CD,PIK3R1,PIK3R5,PLCG2,PLXNA3,PRKG1,RND1,RRAS,SEMA3A,SEMA3F,SEMA5A,SEMA6C,SEMA6D
Amyloid fiber formation	2,22E00	3,33E-01	1,091	APH1B,APOE,B2M,CALB1,CST3,H2AC6,H2BC11,H2BC12,H2BC17,H2BC21,H2BC26,H2BC5,MFGE8,PRKN,SAA1,SIAH2,SNCA,SNCAIP,SORL1,TGFBI,UBE2L6
OX40 Signaling Pathway	2,22E00	3,33E-01	0,378	B2M,HLA-A,HLA-B,HLA-C,HLA-DOB,HLA-DQA1,HLA-DRA,HLA-DRB1,HLA-DRB5,HLA-E,HLA-F,HLA-G,NFKB1,NFKB2,NFKBIA,NFKBIB,NFKBIE,REL,RELB,TNFRSF4,TRAF3

IL-17 Signaling	2,2E00	2,8E-01	4,938	CCL2,CCL20,CD70,CEBPB,CLCF1,CSF3,CXCL1,CXCL3,CXCL5,CXCL8,HSP90AA1,HSP90B1,IL11,IL15,IL17RC,IL17RE,IL1A,IL1B,IL6,JAK2,LIF,MAP2K3,MAPK11,MAPK3,MRAS,NFKB1,NOS2,PGF,PIK3CD,PIK3R1,PIK3R5,PTGS2,RGS16,RRAS,TGFB2,TGFB3,TNF,TNFSF10,TNFSF12,TNFSF13B,TNFSF9,VEGFC
Relaxin Signaling	2,2E00	2,8E-01	-0,655	ADCY2,ADCY5,ADCY6,ADCY8,ENPP1,GAD1,GDPD1,GNAI1,GNAO1,GNAS,GNAZ,GNB4,GNG12,GNG2,GNG7,GUCY1A1,GUCY1A2,MAPK3,MPPED2,MRAS,NFKB1,NFKB2,NFKBIA,NFKBIB,NFKBIE,NOS2,NPR3,PDE10A,PDE1A,PDE1C,PDE2A,PDE3A,PDE6D,PDE8B,PDE9A,PIK3CD,PIK3R1,PIK3R5,PLD6,REL,RELB,SMARCC2

Hepatic Cholestasis	2,19E00	2,68E-01	2,885	ABCB11,ABCC1,ADCY2,ADCY5,ADCY6,ADCY8,CD70,CLCF1,CXCL8,ESR1,FGF19,FGFR4,GPER1,GUCY1A1,IKBKE,IL11,IL15,IL1A,IL1B,IL1R1,IL1RAP,IL1RN,IL6,IRAK2,LIF,MAP4K2,MAPK3,MYD88,NFKB1,NFKB2,NFKBIA,NFKBIB,NFKBIE,NGFR,NOS2,NR1H4,PPARA,PPP3CC,PRKCA,PRKCQ,PRKD1,REL,RELB,SLCO1C1,TGFB2,TGFB3,TLR4,TNF,TNFRSF1B,TNFSF10,TNFSF12,TNFSF13B,TNFSF9
Response to elevated platelet cytos	2,17E00	2,88E-01	-0,667	A1BG,A2M,ENDOD1,FLNA,FN1,GTPBP2,HABP4,HGF,IGF2,ITGA2B,ITGB3,LEFTY2,LGALS3BP,MAGED2,MMRN1,PCDH7,PDGFA,PDGFB,PRKCA,PROS1,PSAP,RAB27B,SELENOP,SERPINA1,SERPINA3,SERPING1,SRGN,STXBP2,SYTL4,TGFB2,TGFB3,THBS1,TIMP1,TIMP3,VEGFC,VWF

IL-33 Signaling Pathway	2,16E00	2,77E-01	2,466	CASP1,CASP4,CASP6,CASP7,CASP8,CASQ1,CCL2,CCL5,CREB3L4,CXCL8,H2AZ2,H2BC12,H2BC17,H2BC26,H2BC5,H2BC8,ICAM1,ICAM4,IKBKE,IL1A,IL1B,IL1RAP,IL1RN,IL6,JAK2,MAP3K8,MAPK11,MAPK3,MYD88,NFKB1,NFKB2,NFKBIA,NFKBIB,NFKBIE,PIK3CD,PIK3R1,PIK3R5,PLCG2,PTGS2,SIGIRR,TBK1,TNF,VCAM1
Glioblastoma Multiforme Signaling	2,16E00	2,74E-01	0,324	APC,APC2,CCND1,CCND2,CCND3,CDKN2A,E2F8,FZD1,FZD5,FZD9,IGF2,ITPR1,ITPR3,MAPK3,MDM2,MRAS,MYC,PDGFA,PDGFB,PDGFRB,PIK3CD,PIK3R1,PIK3R5,PLCD1,PLCD3,PLCE1,PLCG2,PLCL2,RAC2,RHOC,RHOU,RND1,RND2,RRAS,SHC2,SHC3,SOS2,SRC,WNT10A,WNT10B,WNT11,WNT2B,WNT3,WNT5A,WNT7B,WNT9B
NCAM signaling for neurite out-grov	2,14E00	3,33E-01	-0,447	CACNA1C,CACNA1H,CACNA1I,CACNA1S,CNTN2,COL4A4,COL4A5,COL5A2,COL6A2,COL6A3,COL9A2,COL9A3,GDNF,MAPK3,NCAM1,NCAN,PTPRA,SPTAN1,SPTBN5,SRC

Natural Killer Cell Signaling	2,13E00	2,69E-01	1,155	B2M,COL1A1,HLA-A,HLA-B,HLA-C,HLA-E,HLA-F,HLA-G,HSPA1A/HSPA1B,HSPA2,HSPA9,IL12RB1,IL15,IL18R1,JAK2,LCP2,LIMK2,MAP2K3,MAP3K6,MAP3K8,MAPK11,MAPK3,MICA,MICB,MRAS,MYD88,NECTIN2,NFAT5,NFATC2,NFATC4,NFKB1,NFKB2,PAK1,PAK6,PIK3CD,PIK3R1,PIK3R5,PLCG2,PRKCQ,PTK2B,PVR,RAC2,RASSF5,REL,RELB,RRAS,SOS2,TNFSF10,VAV3,WAS
Regulation of the Epithelial Mesenc	2,13E00	2,69E-01	0,905	CD70,DOCK10,ETS1,FGF19,FGF22,FGFR2,FGFR3,FGFR4,GAB1,HGF,ID2,IKBKE,IL6,JAK2,MAP2K3,MAP2K5,MAPK11,MAPK3,MEST,MET,MRAS,NFKB1,NFKB2,NGFR,PARD6B,PARD6G,PDGFA,PDGFB,PDGFRB,PIK3CD,PIK3R1,PIK3R5,REL,RELB,RRAS,SHC2,SHC3,SMURF1,SNAI2,SOS2,TGFB2,TGFB3,TNF,TNFRSF1B,TNFSF10,TNFSF12,TNFSF13B,TNFSF9,VIM,ZEB1

G alpha (12/13) signalling events	2,13E00	3,12E-01	-3,800	ABR,ADRA1A,ARHGEF10,ARHGEF16,ARHGEF17,ARHGEF26,ARHGEF37,ARHGEF39,ARHGEF4,ARHGEF6,FGD1,FGD3,GNB4,GNG12,GNG2,GNG7,ITSN1,KALRN,MCF2L,OBSCN,PLEKHG2,PLEKHG5,RHOC,SOS2,VAV3
Retinoid metabolism and transport	2,13E00	3,66E-01	-0,775	APOE,BCO1,GPC2,GPC4,LPL,LRAT,LRP1,LRP10,LRP8,PLB1,RBP1,SDC1,SDC2,SDC3,SDC4
IL-23 Signaling Pathway	2,13E00	3,66E-01	2,887	IL12RB1,IL1B,IL23A,JAK2,NFKB1,NFKB2,NFKBIA,PIK3CD,PIK3R1,PIK3R5,REL,RELB,RORA,RUNX1,TNF
DDX58/IFIH1-mediated induction of	2,13E00	3,24E-01	4,264	CASP8,CYLD,HERC5,HSP90AA1,IFIH1,IKBKE,IRF7,ISG15,NFKB1,NFKB2,NFKBIA,NFKBIB,NLRC5,RIGI,S100B,SAA1,TBK1,TNFAIP3,TRAF3,TRIM25,UBA7,UBE2L6
Interleukin-1 family signaling	2,11E00	2,86E-01	5,667	ALOX5,CASP1,IL18BP,IL18R1,IL1A,IL1B,IL1R1,IL1RAP,IL1RN,IRAK2,MAP3K8,MYD88,NFKB1,NFKBIA,NOD1,NOD2,PELI2,PSMA2,PSMA3,PSMA5,PSMA6,PSMB10,PSMB8,PSMB9,PSMC4,PSMD11,PSME1,PSME2,PSMF1,PTPN12,PTPN2,RIPK2,SIGIRR,SQSTM1,TAB3,TBK1

Serotonin Receptor Signaling	2,1E00	2,4E-01	-0,194	ADCY2,ADCY5,ADCY6,ADCY8,ADRA1A,ADRA2B,ADRB1,BST1,CACNA1A,CACNA1C,CACNA1E,CACNA1H,CACNA1I,CACNA1S,CACNG4,CAMK2A,CAV1,CAV2,CD38,CPE,CREB3L4,CXCL8,GCH1,GNAI1,GNAO1,GNAS,GNB4,GNG12,GNG2,GNG7,GUCY1A1,H3-3A/H3-3B,HCK,IL1B,IL4I1,IL6,ITGA2B,ITGB3,JAK2,KALRN,KCNH2,KCNN3,LYN,MAOB,MAP2K3,MAP2K5,MAPK11,MAPK3,MAPT,MPDZ,MRAS,MYC,MYLK,MYLK3,NFKB1,NFKB2,NFKBIA,ORAI1,PAK1,PAK6,PIK3CD,PIK3R1,PIK3R5,PLA2G4A,PLA2G4C,PLA2G6,PLAAT2,PLAAT4,PLAUR,PLB1,PLCD1,PLCD3,PLCE1,PLCG2,PLCL2,PLD1,PLD2,PLD5,PLD6,PRKCA,PRKCQ,PRKD1,PSD4,PTGS1,PTGS2,RAC2,RHOC,RHOU,RND1,RND2,RRAS,RYR2,SLC18A2,SMO
PPAR Signaling	2,1E00	2,97E-01	-2,646	CITED2,HSP90AA1,HSP90B1,IKBKE,IL1A,IL1B,IL1R1,IL1RAP,IL1RN,MAPK3,MRAS,NFKB1,NFKB2,NFKBIA,NFKBIB,NFKBIE,NGFR,PDGFA,PDGFB,PDGFRB,PPARA,PPARG,PTGS2,REL,RELB,RRAS,SOS2,STAT5A,TNF,TNFRSF1B
4-1BB Signaling in T Lymphocytes	2,09E00	3,82E-01	1,890	IKBKE,MAPK11,MAPK3,NFKB1,NFKB2,NFKBIA,NFKBIB,NFKBIE,REL,RELB,TNFRSF9,TNFSF9,TRAF1

MYC Mediated Apoptosis Signaling	2,08E00	3,47E-01	1,213	ADRB1,BBC3,BID,CASP8,CDKN2A,CFLAR,FAS,IKBKE,MAX,MDM2,MYC,NFKB1,NFKB2,NGFR,PMAIP1,TNF,TNFRSF1B
WNT/Ca+ pathway	2,05E00	3,23E-01	-0,688	CAMK2A,CREB3L4,FZD1,FZD5,FZD9,NFAT5,NFATC2,NFATC4,NFKB1,NFKB2,PDE6D,PLCD1,PLCD3,PLCE1,PLCG2,PLCL2,PRKCA,REL,RELB,ROR1,WNT5A
TRAIL signaling	2,05E00	6,25E-01	1,342	CASP8,CFLAR,TNFRSF10A,TNFRSF10D,TNFSF10
Docosahexaenoic Acid (DHA) Signa	2,04E00	2,57E-01	-0,516	ADCY2,ADCY5,ADCY6,ADCY8,APH1B,BCL2A1,BID,BIRC3,CASP7,CASP8,CAT,CCL20,CCND1,CREB3L4,FABP5,FAS,GNAS,GUCY1A1,HGF,IL1B,ITPR1,ITPR3,MAPK3,MFSD2A,NGFR,NTRK2,PDGFB,PIK3CD,PIK3R1,PIK3R5,PLA2G4A,PLA2G4C,PLA2G6,PLAAT2,PLAAT4,PLB1,PLCD1,PLCD3,PLCE1,PLCG2,PLCL2,PRKCA,PRKCQ,PRKD1,PTGS2,REL,SERPINF1,SPTBN5,STXBP2,STXBP6,SYT1,SYT12,SYT14,SYT6,SYT7,TGFA,TGFB2,TNF,TNFAIP2,TNFRSF1B,ULK1
Role of IL-17F in Allergic Inflammat	2,02E00	3,57E-01	1,897	CCL2,CREB3L4,CXCL1,CXCL10,CXCL5,CXCL8,IL11,IL17RC,IL1B,IL6,MAPK3,NFKB1,NFKB2,REL,RELB
Differential Regulation of Cytokine P	2,02E00	0.5	2,646	CCL2,CCL5,CSF3,CXCL1,IL1B,IL6,TNF

IL-15 Production	2,01E00	2,87E-01	1,257	EPHA5,EPHB2,ERBB4,FGFR2,FGFR3,FGFR4,FLT3LG,HCK,IL15,IL6,IRF1,JAK2,LMTK3,LYN,MAP2K3,MAP2K5,MATK,MET,NFKB1,NFKB2,NTRK2,PDGFRB,PTK2B,REL,RELB,RET,ROR1,ROS1,SRC,STAT1,TEC,TEK,TYRO3
Sphingosine-1-phosphate Signaling	2,01E00	2,87E-01	0,000	ADCY2,ADCY5,ADCY6,ADCY8,CASP1,CASP4,CASP6,CASP7,CASP8,CASQ1,GNAI1,GUCY1A1,MAPK3,PDGFA,PDGFB,PDGFRB,PIK3CD,PIK3R1,PIK3R5,PLCD1,PLCD3,PLCE1,PLCG2,PLCL2,PTK2B,RAC2,RHOC,RHOU,RND1,RND2,S1PR1,SMPD1,SMPD3
γ-glutamyl Cycle	2,01E00	5,45E-01	1,633	ANPEP,CHAC1,CHAC2,GCLM,GGT5,OPLAH
IL-17A Signaling in Gastric Cells	2	4,17E-01	1,342	CCL20,CCL5,CXCL1,CXCL10,CXCL8,IL17RC,MAPK11,MAPK3,NFKB1,TNF

Pulmonary Healing Signaling Pathw	1,99E00	2,65E-01	0,283	CCND1,CDC25C,CXCL12,CXCR4,DLK1,FGFR2,FZD1,FZD5,FZD9,HCK,LYN,MAPK11,MAPK3,MMP14,MMP15,MMP16,MMP24,MMP25,MRAS,MYC,MYD88,NFKB1,NFKB2,NFKBIA,NGFR,PGF,PRKCA,PRKCQ,PRKD1,RRAS,SMAD9,SOS2,SRC,STAT5A,TCF7L1,TGFB2,TGFB3,THBS1,TLR4,TNF,TNFRSF1B,VEGFC,WNT10A,WNT10B,WNT11,WNT2B,WNT3,WNT5A,WNT7B,WNT9B
Platelet homeostasis	1,99E00	3,02E-01	0,000	ATP2B2,GNAS,GNB4,GNG12,GNG2,GNG7,GUCY1A1,GUCY1A2,IRAG1,ITPR1,ITPR3,KCNMB4,LRP8,NOS2,ORAI1,P2RX4,P2RX5,P2RX6,PDE10A,PDE1A,PDE2A,PDE9A,PLA2G4A,PPP2R1B,PRKG1,STIM1

G-Protein Coupled Receptor Signal	1,98E00	2,29E-01	0,487	ADCY2,ADCY5,ADCY6,ADCY8,ADGRA2,ADGRB2,ADGRB3,ADGRE5,ADGRG1,ADGRL3,ADGRV1,ADORA2A,ADORA2B,ADRA1A,ADRA2B,ADRB1,AMOT,BDKRB2,C3AR1,C5AR1,CAMK2A,CARD10,CCND1,CCR1,CCR3,CELSR2,CHRM4,CNR1,CREB3L4,DRD4,DUSP1,DUSP9,EDNRB,ENPP1,F2RL1,FOXO4,FOXO6,FZD1,FZD5,FZD9,GABBR1,GABBR2,GALR2,GDPD1,GIPR,GNAI1,GNAO1,GNAS,GNAZ,GNB4,GNG12,GNG2,GNG7,GPER1,GPR137B,GPR137C,GPR139,GPR141,GPR146,GPR157,GPR162,GPR176,GPR180,GPR27,GPR63,GPR88,GRM8,GRPR,GUCY1A1,HCN2,HRH2,IKBKE,KCNH2,KCNN3,KISS1R,LGR4,LPAR1,LPAR2,LPAR3,LPAR4,LTB4R,LTB4R2,MAP2K3,MAP2K5,MAP3K6,MAP3K8,MAPK11,MAPK3,MCHR1,MPPED2
Renin-Angiotensin Signaling	1,97E00	2,83E-01	1,000	ADCY2,ADCY5,ADCY6,ADCY8,CCL2,CCL5,GUCY1A1,ITPR1,ITPR3,JAK2,MAPK11,MAPK3,MRAS,NFKB1,NFKB2,PAK1,PAK6,PIK3CD,PIK3R1,PIK3R5,PLCG2,PRKCA,PRKCQ,PRKD1,PTK2B,REL,RELB,RRAS,SHC2,SHC3,SHF,SOS2,STAT1,TNF
DNA methylation	1,94E00	4,29E-01	1,667	DNMT3B,H2AC6,H2AZ2,H2BC11,H2BC12,H2BC17,H2BC21,H2BC26,H2BC5

Acetylcholine Receptor Signaling Pa	1,93E00	2,63E-01	-2,143	ADAM9,ADCY2,ADCY5,ADCY6,ADCY8,APH1B,CACNA1A,CACNA1C,CACNA1E,CACNA1H,CACNA1I,CACNA1S,CACNG4,CAMK2A,CASP1,CASP4,CASP6,CASP7,CASP8,CASQ1,CHRFAM7A,CHRM4,CHRNA1,CREB3L4,GNAI1,GNAO1,GUCY1A1,IL6,ITPR1,ITPR3,JAK2,MAPK3,NFKB1,NFKB2,NGFR,NOS2,PIK3CD,PIK3R1,PIK3R5,PLCD1,PLCD3,PLCE1,PLCG2,PLCL2,PRKCA,PRKCQ,PRKD1,TH,TNF
B Cell Activating Factor Signaling	1,92E00	3,49E-01	1,667	IKBKE,MAPK11,NFAT5,NFATC2,NFATC4,NFKB1,NFKB2,NFKBIA,NFKBIB,NFKBIE,REL,RELB,TNFSF13B,TRAF1,TRAF3
PD-1, PD-L1 cancer immunotherap	1,89E00	2,9E-01	-3,530	B2M,CD274,HLA-A,HLA-B,HLA-C,HLA-DOB,HLA-DQA1,HLA-DRA,HLA-DRB1,HLA-DRB5,HLA-E,HLA-F,HLA-G,IFNGR2,JAK2,LCP2,MR1,NGFR,PDCD1LG2,PIK3CD,PIK3R1,PIK3R5,PRKCQ,RASGRP1,STAT5A,TGFB2,TGFB3,TNF,TNFRSF1B
IL-7 Signaling Pathway	1,89E00	3,1E-01	2,668	BAK1,CCND1,CCND3,CDC25A,FOXO4,FOXO6,HGF,IL7,IL7R,LYN,MAPK11,MAPK3,MET,MYC,PAX5,PIK3CD,PIK3R1,PIK3R5,SOCS1,SOS2,STAT1,STAT5A

NAD Signaling Pathway	1,89E00	2,73E-01	2,271	BST1,CD38,CEBPB,H1-0,H2BC12,H2BC17,H2BC26,H2BC5,H2BC8,HGF,HSPD1,LDHA,LDHD,NADK,NAMPT,NFKB1,NFKB2,NMNAT1,NT5E,PARP10,PARP12,PARP14,PARP4,PARP8,PARP9,PDGFB,PIK3CD,PIK3R1,PIK3R5,POLR2H,RYR2,SIRT2,SLC29A1,SLC29A3,SLC29A4,SLC7A5,TGFA,TGFB2,TRPM2
Induction of Apoptosis by HIV1	1,89E00	3,17E-01	1,886	BAK1,BBC3,BID,BIRC3,CASP8,CXCR4,FAS,IKBKE,NAIP,NFKB1,NFKB2,NFKBIA,NFKBIB,NFKBIE,NGFR,REL,RELB,TNF,TNFRSF1B,TRAF1
Transcriptional regulation by RUNX:	1,88E00	2,92E-01	3,272	CCN2,CCND1,CDKN2A,HES1,ITGA4,KAT2A,MDM2,MYC,PSMA2,PSMA3,PSMA5,PSMA6,PSMB10,PSMB8,PSMB9,PSMC4,PSMD11,PSME1,PSME2,PSMF1,RUNX1,RUNX3,SMURF1,SPP1,SRC,TCF7L1,TEAD3,TEAD4
Fcγ Receptor-mediated Phagocytos	1,87E00	2,93E-01	-1,177	ACTA1,ACTA2,ACTB,ACTG1,ACTG2,DGKB,HCK,LCP2,LYN,MAPK3,MYO5A,PAK1,PIK3R1,PLA2G6,PLD1,PLD2,PLD5,PLD6,PRKCA,PRKCQ,PRKD1,PTK2B,RAB11B,RAC2,SRC,VAV3,WAS

FXR/RXR Activation	1,86E00	2,67E-01	-4,727	ABCB11,APOE,CASP8,CD70,CLCF1,CXCL8,DDIT3,FGF19,FGFR4,GCLM,GSTA4,GSTM2,GSTM3,GSTM4,GSTO1,GSTO2,GSTT2/GSTT2B,ICAM1,IL11,IL15,IL1A,IL1B,IL1RN,IL6,LIF,LPL,NFKB1,NFKB2,NR1H4,PCK2,PPARA,PPARG,PTGS2,SDC1,SRC,TGFB2,TGFB3,TNF,TNFSF10,TNFSF12,TNFSF13B,TNFSF9,TXNIP
LXR/RXR Activation	1,86E00	2,84E-01	-2,117	A1BG,ABCG4,APOE,APOL1,C3,C4A/C4B,CCL2,IL1A,IL1B,IL1R1,IL1RAP,IL1RN,IL6,LPL,NFKB1,NFKB2,NGFR,NOS2,NR1H4,PCYOX1,PTGS2,REL,RELB,SAA1,SAA2,SERPINA1,SERPINF1,TLR3,TLR4,TNF,TNFRSF1B
STAT3 Pathway	1,85E00	2,74E-01	-0,200	CDC25A,FGFR2,FGFR3,FGFR4,GHR,HGF,IL10RA,IL12RB1,IL15RA,IL17RC,IL17RD,IL17RE,IL18R1,IL1A,IL1B,IL1R1,IL20RA,IL4R,IL6ST,IL7R,JAK2,MAPK11,MAPK3,MRAS,MYC,NGFR,NTRK2,PDGFB,PDGFRB,PTPN2,RRAS,SOCS1,SRC,TGFA,TGFB2,TGFBR3,TNFRSF11A

HGF Signaling	1,84E00	2,75E-01	0,853	CCND1,CDKN2A,ELF1,ELF3,ELF4,ETS1,ETS2,GAB1,HGF,IL6,ITGA1,ITGA2B,ITGA4,ITGA5,ITGA7,ITGAE,ITGB3,ITGB4,ITGB5,ITGB7,MAP3K6,MAP3K8,MAPK3,MET,MRAS,PAK1,PIK3CD,PIK3R1,PIK3R5,PLCG2,PRKCA,PRKCQ,PRKD1,PTGS2,RRAS,SOS2
Colorectal Cancer Metastasis Signa	1,83E00	2,5E-01	0,700	ADCY2,ADCY5,ADCY6,ADCY8,APC,CCND1,FZD1,FZD5,FZD9,GNAI1,GNAO1,GNAS,GNAZ,GNB4,GNG12,GNG2,GNG7,GUCY1A1,IL6,IL6ST,JAK2,LRP1,MAPK3,MMP14,MMP15,MMP16,MMP24,MMP25,MRAS,MYC,NFKB1,NFKB2,NOS2,PGF,PIK3CD,PIK3R1,PIK3R5,PTGS2,RAC2,REL,RELB,RHOC,RHOU,RND1,RND2,RRAS,SOS2,SRC,STAT1,TCF7L1,TGFB2,TGFB3,TLR3,TLR4,TNF,VEGFC,WNT10A,WNT10B,WNT11,WNT2B,WNT3,WNT5A,WNT7B,WNT9B

Glutaminergic Receptor Signaling P	1,83E00	2,44E-01	0,000	ADCY2,ADCY5,ADCY6,ADCY8,CACNA1A,CACNA1C,CACNA1E,CACNA1H,CACNA1I,CACNA1S,CACNG4,CAMK2A,CREB3L4,DGKA,DGKB,DGKE,DGKG,DGKI,FMR1,GABBR1,GABBR2,GABRB3,GABRE,GABRG3,GABRP,GNAI1,GNAO1,GNAS,GOSR1,GRIA1,GRIK1,GRIK3,GRM8,GUCY1A1,HOMER1,IKBKE,ITPR1,ITPR3,MAPK11,MAPK3,NAPA,NFKB1,NFKB2,PIK3CD,PIK3R1,PIK3R5,PLA2G4A,PLA2G4C,PLA2G6,PLAAT2,PLAAT4,PLB1,PLCD1,PLCD3,PLCE1,PLCG2,PLCL2,PLD1,PLD2,PLD5,PLD6,PPP3CC,PRKCA,PRKCQ,PRKD1,SCN1A,SCN3A,SLC1A2,SLC38A3,SLC38A5,SLC7A11,SNAP25,SRC,TGFB2,TGFB3,TRPC1
GPER1 signaling	1,82E00	3,41E-01	-1,807	ADCY2,ADCY5,ADCY6,ADCY8,FN1,GNAI1,GNAS,GNAZ,GNB4,GNG12,GNG2,GNG7,GPER1,ITGA5,SRC

Gαq Signaling	1,82E00	2,65E-01	0,000	ADRA1A,GNAI1,GNAO1,GNAS,GNAZ,GNB4,GNG12,GNG2,GNG7,IKBKE,ITPR1,ITPR3,MAPK3,MRAS,NFATC2,NFATC4,NFKB1,NFKB2,NFKBIA,NFKBIB,NFKBIE,PIK3CD,PIK3R1,PIK3R5,PLCG2,PLD1,PLD2,PLD5,PLD6,PPP3CC,PRKCA,PRKCQ,PRKD1,PTK2B,RAC2,REL,RELB,RGS16,RGS2,RHOC,RHOU,RND1,RND2
G alpha (z) signalling events	1,81E00	3,33E-01	-1,500	ADCY2,ADCY5,ADCY6,ADCY8,ADRA2B,GNAI1,GNAS,GNAZ,GNB4,GNG12,GNG2,GNG7,PRKCA,PRKCQ,RGS16,RGS20
IL-17A Signaling in Airway Cells	1,81E00	3,12E-01	1,807	CCL20,CXCL1,CXCL3,CXCL5,IKBKE,IL17RC,IL6,JAK2,MAPK11,MAPK3,NFKB1,NFKB2,NFKBIA,NFKBIB,NFKBIE,PIK3CD,PIK3R1,PIK3R5,REL,RELB

Senescence Pathway	1,81E00	2,46E-01	2,077	ACVR2A,CACNA1A,CACNA1C,CACNA1E,CACNA1S,CACNG4,CAPN5,CAPN9,CAT,CBX2,CBX8,CCND1,CCND2,CCND3,CDC25A,CDC25B,CDC25C,CDKN2A,CDKN2B,CEBPB,CXCL8,E2F8,ELF1,ELF3,ELF4,ETS1,ETS2,FOXO4,GADD45G,IKBKE,IL1A,IL6,ITPR3,MAP2K3,MAP2K5,MAPK3,MAPK4,MDM2,MRAS,NBN,NFAT5,NFATC2,NFATC4,NFKB1,NFKB2,PDK2,PDK3,PDK4,PIK3CD,PIK3R1,PIK3R5,PML,PPM1J,PPP2R1B,PPP2R2A,PPP2R2B,PPP2R3A,PPP3CC,RASSF5,RRAS,SAA1,SAA2,SMAD9,SQSTM1,STING1,TBK1,TGFB2,TGFB3,TGFBR3,YPEL3
RUNX1 regulates megakaryocyte di	1,81E00	3,27E-01	0,000	GP1BA,H2AC6,H2AZ2,H2BC11,H2BC12,H2BC17,H2BC21,H2BC26,H2BC5,ITGA2B,MOV10,MYL9,PRKCQ,RUNX1,SETD1A,THBS1,TNRC6C
NLR signaling pathways	1,8E00	3,21E-01	3,771	AIM2,BIRC3,CASP1,CASP4,CASP8,CYLD,IRAK2,MAPK11,MEFV,NFKB1,NFKB2,NLRP1,NOD1,NOD2,RIPK2,TAB3,TNFAIP3,TXNIP

Pancreatic Secretion Signaling Path	1,8E00	2,52E-01	0,130	ADCY2,ADCY5,ADCY6,ADCY8,ADORA2A,ADORA2B,AQP1,ARHGEF10,ARHGEF16,ARHGEF17,ARHGEF4,ARHGEF6,ATP2B2,BST1,CA12,CA14,CA2,CA4,CA5B,CA8,CA9,CADPS2,CD38,GNAS,GOSR1,GUCY1A1,IRF2,ITPR1,ITPR3,LIPA,LPL,MAPK3,NAPA,ORAI1,P2RY11,PIK3CD,PIK3R1,PIK3R5,PLCD1,PLCD3,PLCE1,PLCG2,PLCL2,PPP1R3C,PRKCA,PRKCQ,PRKD1,RASGRP3,RGS2,RYR2,SLC4A4,SLC6A6,SNAP25,SRC,STIM1,STXBP2,STXBP6,VIPR1,VIPR2
Growth hormone receptor signaling	1,79E00	4,09E-01	1,667	GHR,IRS1,JAK2,LYN,MAPK3,PRLR,SOCS1,STAT1,STAT5A
WNT/SHH Axonal Guidance Signal	1,79E00	2,69E-01	0,480	ADCY2,ADCY5,ADCY6,ADCY8,CAMK2A,FZD1,FZD5,FZD9,GLI2,GUCY1A1,HCK,ILK,ITPR1,ITPR3,LYN,PARD6B,PARD6G,PIK3CD,PIK3R1,PIK3R5,PLXNA3,PRKCA,PTCH1,SEMA3A,SEMA3D,SEMA3F,SEMA3G,SRC,TCF7L1,TP63,TRPC1,WNT10A,WNT10B,WNT11,WNT2B,WNT3,WNT5A,WNT7B,WNT9B
Reversible hydration of carbon dioxi	1,78E00	0.5	0,000	CA12,CA14,CA2,CA4,CA5B,CA9
Role of IL-17A in Psoriasis	1,78E00	0.5	2,449	CCL20,CXCL1,CXCL3,CXCL5,CXCL8,IL17RC

IL-4 Signaling	1,78E00	2,59E-01	0,000	COL11A1,COL15A1,COL17A1,COL1A1,COL26A1,COL28A1,COL4A4,COL4A5,COL4A6,COL5A2,COL6A2,COL6A3,COL7A1,COL8A2,COL9A2,COL9A3,CREB3L4,FAS,GAB1,GNAI1,HLA-DOB,HLA-DQA1,HLA-DRA,HLA-DRB1,HLA-DRB5,IL4R,IRS1,MAF,MAPK11,MAPK3,MRAS,NFATC2,NFKB1,NFKB2,PIK3CD,PIK3R1,PIK3R5,PRR5,PRR5L,RICTOR,RRAS,SOCS1,SOS2,TGFB2,TGFB3,TGM2,TIMP3,TRPC1
Neuregulin Signaling	1,77E00	2,78E-01	-0,688	BTC,CDK5R1,ERBB4,ERBIN,HSP90AA1,HSP90B1,ITGA1,ITGA2B,ITGA4,ITGA5,ITGA7,ITGAE,ITGB3,ITGB4,ITGB5,ITGB7,MAPK3,MATK,MRAS,MYC,NRG2,PIK3R1,PLCG2,PRKCA,PRKCQ,PRKD1,RRAS,SOS2,SRC,STAT5A,TGFA,TMEFF2
Signaling by ROBO receptors	1,77E00	2,78E-01	-0,707	AKAP5,CAP2,CLASP1,COL4A5,CXCL12,CXCR4,ENAH,EVL,MSI1,PAK1,PAK6,PFN2,PRKCA,PSMA2,PSMA3,PSMA5,PSMA6,PSMB10,PSMB8,PSMB9,PSMC4,PSMD11,PSME1,PSME2,PSMF1,ROBO2,SLIT1,SLIT2,SLIT3,SOS2,SRC,ZSWIM8

Regulation of Insulin-like Growth Fa	1,77E00	2,78E-01	1,061	AMBN,ANO8,APOE,APOL1,C3,C4A/C4B,CCN1,CHRDL1,CP,CSF1,CST3,EVA1A,FN1,FSTL1,HSP90B1,IGF2,IGFBP4,IL6,LAMB2,MFGE8,MGAT4A,MXRA8,PAPPA,PRSS23,SCG2,SDC2,SERPINA1,SHISA5,SPP1,STC2,TIMP1,VGF
Sertoli Cell-Germ Cell Junction Sign	1,76E00	2,52E-01	1,722	A2M,ACTA1,ACTA2,ACTB,ACTG1,ACTG2,CD2AP,CLDN1,CLDN5,CTNNA1,CTNND1,CXADR,FOSB,FOSL1,GUCY1A1,GUCY1A2,ICAM1,IL1A,IL1R1,IL1RAP,ILK,JAM2,LAMA1,LAMA2,LAMA3,LAMA5,LAMB2,LAMB3,MAP2K3,MAP3K6,MAP3K8,MAPK11,MAPK3,MRAS,NECTIN2,NECTIN3,NFKB1,NFKB2,NGFR,NOS2,PIK3CD,PIK3R1,PIK3R5,PPM1J,PPP2R1B,PPP2R2A,PPP2R2B,PPP2R3A,PRKG1,RAC2,RRAS,SRC,TGFB2,TGFB3,TNF,TNFRSF1B,ZYX
Th1 Pathway	1,75E00	2,79E-01	4,131	APH1B,CD274,CD40,DLL4,HLA-A,HLA-B,HLA-DOB,HLA-DQA1,HLA-DRA,HLA-DRB1,HLA-DRB5,ICAM1,ICOSLG/LOC102723996,IFNGR2,IL10RA,IL12RB1,IL18R1,IL6,IRF1,JAK2,LGALS9,NFATC2,NFATC4,NFKB1,PIK3CD,PIK3R1,PIK3R5,PRKCQ,RUNX3,SOCS1,STAT1

PI3K Signaling in B Lymphocytes	1,74E00	2,67E-01	2,401	ATF1,ATF5,C3,CAMK2A,CARD10,CD180,CD40,IKBKE,IL4R,IRS1,IRS4,ITPR1,ITPR3,LYN,MAPK3,MRAS,NFAT5,NFATC2,NFATC4,NFKB1,NFKB2,NFKBIA,NFKBIB,NFKBIE,PIK3AP1,PIK3CD,PIK3R1,PLCD1,PLCD3,PLCE1,PLCG2,PLCL2,PLEKHA4,PPP3CC,REL,RELB,RRAS,TLR4,VAV3
GABA synthesis, release, reuptake	1,72E00	4,21E-01	-2,121	ABAT,ALDH5A1,GAD1,GAD2,SLC32A1,SLC6A1,SNAP25,SYT1
IL-6 Signaling	1,7E00	2,72E-01	3,413	A2M,CEBPB,COL1A1,CXCL8,IKBKE,IL1A,IL1B,IL1R1,IL1RAP,IL1RN,IL6,IL6ST,JAK2,MAP2K3,MAPK11,MAPK3,MRAS,NFKB1,NFKB2,NFKBIA,NFKBIB,NFKBIE,NGFR,PIK3CD,PIK3R1,PIK3R5,REL,RELB,RRAS,SOCS1,SOS2,TNF,TNFAIP6,TNFRSF1B
MSP-RON Signaling in Macrophage	1,7E00	2,77E-01	-2,646	CIITA,CREB3L4,HLA-DOB,HLA-DQA1,HLA-DRA,HLA-DRB1,HLA-DRB5,IFNGR2,IKBKE,JAK2,KLK10,MAPK3,MRAS,MST1,NFKB1,NFKB2,NFKBIZ,NOS2,PIK3CD,PIK3R1,PIK3R5,PTGS2,REL,RELB,RRAS,SBNO2,SOCS1,SOS2,STAT1,TLR4,TNF

Phospholipase C Signaling	1,69E00	2,44E-01	-1,067	ADCY2,ADCY5,ADCY6,ADCY8,AHNAK,ARHGEF10,ARHGEF16,ARHGEF17,ARHGEF4,ARHGEF6,CREB3L4,GNAS,GNB4,GNG12,GNG2,GNG7,GUCY1A1,HDAC5,HDAC9,ITGA1,ITGA2B,ITGA4,ITGA5,ITGA7,ITGAE,ITGB3,ITGB4,ITGB5,ITGB7,ITPR1,ITPR3,JMJD7-PLA2G4B,LCP2,LYN,MAPK3,MRAS,MYL6,MYL9,NFAT5,NFATC2,NFATC4,NFKB1,NFKB2,PLA2G4A,PLA2G4C,PLA2G6,PLCD1,PLCD3,PLCE1,PLCG2,PLD1,PLD2,PLD5,PLD6,PPP3CC,PRKCA,PRKCQ,PRKD1,RAC2,REL,RELB,RHOC,RHOU,RND1,RND2,RRAS,SOS2,SRC,TGM2
Class B/2 (Secretin family receptors	1,69E00	2,84E-01	-0,577	ADGRE5,ADM2,CD55,CRHBP,DHH,FZD1,FZD5,FZD9,GIPR,GNAS,GNB4,GNG12,GNG2,GNG7,PTCH1,PTHLH,UCN2,VIPR1,VIPR2,WNT10A,WNT10B,WNT11,WNT2B,WNT3,WNT5A,WNT7B,WNT9B

Endocannabinoid Cancer Inhibition	1,68E00	2,66E-01	0,493	ADCY2,ADCY5,ADCY6,ADCY8,CASP1,CASP4,CASP6,CASP7,CASP8,CASQ1,CCND1,CCND2,CCND3,CNR1,CREB3L4,DDIT3,GNAI1,GNAO1,GUCY1A1,MAP2K3,MAP2K5,MAPK3,MYC,NOS2,NUPR1,PGF,PIK3CD,PIK3R1,PIK3R5,SMPD1,SMPD3,SNAI2,SPTLC2,SRC,TCF7L1,TWIST2,VEGFC,VIM
Activated PKN1 stimulates transcrip	1,65E00	3,91E-01	1,667	AR,H2AC6,H2AZ2,H2BC11,H2BC12,H2BC17,H2BC21,H2BC26,H2BC5
Differential Regulation of Cytokine P	1,65E00	4,38E-01	2,646	CCL2,CCL5,CSF3,CXCL1,IL1A,IL1B,TNF
Signaling by PDGF	1,65E00	3,1E-01	-0,943	COL4A4,COL4A5,COL5A2,COL6A2,COL6A3,COL9A2,COL9A3,PDGFA,PDGFB,PDGFRB,PIK3R1,PTPN12,SPP1,SRC,STAT1,STAT5A,THBS1,THBS3
April Mediated Signaling	1,65E00	3,33E-01	0,302	IKBKE,MAPK11,NFAT5,NFATC2,NFATC4,NFKB1,NFKB2,NFKBIA,NFKBIB,NFKBIE,REL,RELB,TRAF1,TRAF3
Netrin-1 signaling	1,64E00	3,2E-01	-3,000	ABLIM2,AGAP2,MAPK11,MYO10,NEO1,NTN4,PAK1,PRKCQ,RGMA,SIAH2,SLIT1,SLIT2,SLIT3,SRC,TRPC1,UNC5A

LPS/IL-1 Mediated Inhibition of RXF	1,63E00	2,5E-01	2,840	ABCB11,ACSBG1,ACSF2,ACSL4,ACSL5,ALDH1A3,ALDH1L2,ALDH2,ALDH3A1,ALDH3B1,ALDH4A1,ALDH5A1,ALDH6A1,APOE,CAT,CHST10,CHST11,CHST15,CRAT,CYP2E1,CYP2J2,ECSIT,FABP5,FMO1,GSTA4,GSTM2,GSTM3,GSTM4,GSTO1,GSTO2,GSTT2/GSTT2B,HS3ST1,HS3ST3A1,HS3ST4,HS3ST5,IL1A,IL1B,IL1R1,IL1RAP,IL1RN,IL4I1,MAOB,MYD88,NGFR,NR1H4,PPARA,PPARGC1B,SLC27A2,SLC27A6,SMOX,TLR4,TNF,TNFRSF1B,UST
Thrombin Signaling	1,63E00	2,5E-01	0,845	ADCY2,ADCY5,ADCY6,ADCY8,ARHGEF10,ARHGEF16,ARHGEF17,ARHGEF26,ARHGEF37,ARHGEF39,ARHGEF4,ARHGEF6,CAMK2A,GATA2,GATA6,GNAI1,GNAO1,GNAS,GNAZ,GNB4,GNG12,GNG2,GNG7,GUCY1A1,ITPR1,ITPR3,MAPK11,MAPK3,MRAS,MYL6,MYL9,MYLK,NFKB1,NFKB2,PIK3CD,PIK3R1,PIK3R5,PLCD1,PLCD3,PLCE1,PLCG2,PLCL2,PRKCA,PRKCQ,PRKD1,RAC2,REL,RELB,RHOC,RHOU,RND1,RND2,RRAS,SRC

Role of NFAT in Cardiac Hypertroph	1,63E00	2,5E-01	-0,447	ADCY2,ADCY5,ADCY6,ADCY8,AKAP5,CACNA1A,CACNA1C,CACNA1E,CACNA1H,CACNA1I,CACNA1S,CACNG4,CAMK2A,GNAI1,GNAS,GNB4,GNG12,GNG2,GNG7,GUCY1A1,HDAC5,HDAC9,IL11,IL6,IL6ST,ITPR1,ITPR3,LIF,MAP2K3,MAPK11,MAPK3,MRAS,NFATC4,PIK3CD,PIK3R1,PIK3R5,PLCD1,PLCD3,PLCE1,PLCG2,PLCL2,PPP3CC,PRKCA,PRKCQ,PRKD1,RCAN2,RCAN3,RRAS,SHC2,SHC3,SOS2,SRC,TGFB2,TGFB3
ERK/MAPK Signaling	1,62E00	2,51E-01	1,067	ATF1,CREB3L4,DUSP1,DUSP2,DUSP9,ELF1,ELF3,ELF4,ESR1,ETS1,ETS2,H3-3A/H3-3B,ITGA1,ITGA2B,ITGA4,ITGA5,ITGA7,ITGAE,ITGB3,ITGB4,ITGB5,ITGB7,JMJD7-PLA2G4B,KSR1,MAPK3,MRAS,MYC,MYCN,PAK1,PAK6,PIK3CD,PIK3R1,PIK3R5,PLA2G4A,PLA2G4C,PLA2G6,PLCG2,PPARG,PPM1J,PPP1R3C,PPP2R1B,PPP2R2A,PPP2R2B,PPP2R3A,PRKCA,PTK2B,RAC2,RRAS,SOS2,SRC,STAT1,VRK2

P2Y Purigenic Receptor Signaling F	1,62E00	2,67E-01	-1,134	ADCY2,ADCY5,ADCY6,ADCY8,CREB3L4,GNAI1,GNB4,GNG12,GNG2,GNG7,GUCY1A1,ITGA2B,ITGB3,MAPK3,MRAS,MYC,NFKB1,NFKB2,P2RY1,P2RY11,P2RY2,PIK3CD,PIK3R1,PIK3R5,PLCD1,PLCD3,PLCE1,PLCG2,PLCL2,PRKCA,PRKCQ,PRKD1,REL,RELB,RRAS
CREB Signaling in Neurons	1,62E00	2,27E-01	-1,008	ADCY2,ADCY5,ADCY6,ADCY8,ADGRA2,ADGRB2,ADGRB3,ADGRE5,ADGRG1,ADGRL3,ADGRV1,ADORA2A,ADORA2B,ADRA1A,ADRA2B,ADRB1,BDKRB2,C3AR1,C5AR1,CACNA1A,CACNA1C,CACNA1E,CACNA1H,CACNA1I,CACNA1S,CACNG4,CAMK2A,CCR1,CCR3,CELSR2,CHRM4,CNR1,CREB3L4,DRD4,EDNRB,F2RL1,FGFR2,FGFR3,FGFR4,FZD1,FZD5,FZD9,GABBR1,GABBR2,GALR2,GHR,GIPR,GNAI1,GNAO1,GNAS,GNAZ,GNB4,GNG12,GNG2,GNG7,GPER1,GPR137B,GPR137C,GPR139,GPR141,GPR146,GPR157,GPR162,GPR176,GPR180,GPR27,GPR63,GPR88,GRIA1,GRID2,GRIK1,GRIK3,GRM8,GRPR,GTF2B,GUCY1A1,HGF,HRH2,ITPR1,ITPR3,KISS1R,LGR4,LPAR1,LPAR2,LPAR3,LPAR4,LTB4R,LTB4R2,MAPK3,MCH
Effects of PIP2 hydrolysis	1,61E00	3,7E-01	1,265	DGKA,DGKB,DGKE,DGKG,DGKI,ITPR1,ITPR3,MGLL,PRKCQ,RASGRP1

BMAL1:CLOCK,NPAS2 activates c	1,61E00	3,7E-01	1,265	BHLHE41,BMAL2,DBP,HELZ2,NAMPT,NOCT,NPAS2,PPARA,SMARCD3,TGS1
Pyroptosis	1,61E00	3,7E-01	2,530	BAK1,CASP1,CASP4,GSDMD,GSDME,IL1A,IL1B,IRF1,IRF2,TP63
RHOA Signaling	1,61E00	2,71E-01	-1,826	ACTA1,ACTA2,ACTB,ACTG1,ACTG2,CDC42EP1,CDC42EP2,DLC1,LIMK2,LPAR1,LPAR2,LPAR3,LPAR4,MYL6,MYL9,MYLK,MYLK3,NRP2,PFN2,PIP5KL1,PLD1,PLEKHG5,PTK2B,RHPN2,SEMA3F,SEPTIN1,SEPTIN11,SEPTIN3,SEPTIN4,SEPTIN5,SEPTIN7,SEPTIN8
Trafficking and processing of endos	1,59E00	4,62E-01	2,449	CTSL,CTSS,HSP90B1,LGMN,TLR3,UNC93B1
Apoptosis Signaling	1,59E00	2,77E-01	0,392	BAK1,BCL2A1,BID,BIRC3,CAPN5,CAPN9,CASP6,CASP7,CASP8,FAS,IKBKE,MAPK3,MRAS,NAIP,NFKB1,NFKB2,NFKBIA,NFKBIB,NFKBIE,PLCG2,PRKCA,PRKCQ,REL,RELB,RRAS,SPTAN1,TNF,TNFRSF1B
PEDF Signaling	1,58E00	2,86E-01	0,943	CASP7,CASP8,CFLAR,FAS,GDNF,IKBKE,MAPK11,MAPK3,MRAS,NFKB1,NFKB2,NFKBIA,NFKBIB,NFKBIE,PIK3CD,PIK3R1,PIK3R5,PPARG,REL,RELB,RRAS,SERPINF1,TCF7L1,ZEB1

PPARα/RXRα Activation	1,58E00	2,54E-01	-2,121	ACVR2A,ADCY2,ADCY5,ADCY6,ADCY8,BCL3,CHD5,GHR,GNAS,GPD1,GUCY1A1,HELZ2,HSP90AA1,HSP90B1,IKBKE,IL1B,IL1R1,IL1RAP,IL6,IRS1,ITGB5,JAK2,LPL,MAP2K3,MAPK3,MRAS,NFKB1,NFKB2,NFKBIA,NFKBIB,NFKBIE,PLCD1,PLCD3,PLCE1,PLCG2,PLCL2,PPARA,PRKCA,REL,RELB,RRAS,SOS2,TGFB2,TGFB3,TGFBR3,TGS1
Ethanol Degradation IV	1,57E00	0.4	-1,414	ACSS1,ALDH1A3,ALDH2,ALDH3A1,ALDH3B1,ALDH4A1,CAT,CYGB

FAK Signaling	1,57E00	2,24E-01	1,678	ACVR2A,ADGRA2,ADGRB2,ADGRB3,ADGRE5,ADGRG1,ADGRL3,ADGRV1,ADORA2A,ADORA2B,ADRA1A,ADRA2B,ADRB1,APC,ARHGEF6,BCAR3,BDKRB2,C3AR1,C5AR1,CAPN5,CAPN9,CCND1,CCR1,CCR3,CELSR2,CHRM4,CNR1,COL1A1,DRD4,EDNRB,EFNA1,EFNA5,EFNB1,EFNB3,ELF1,ELF3,ELF4,ETS1,ETS2,F2RL1,FZD1,FZD5,FZD9,GABBR1,GABBR2,GALR2,GHR,GIPR,GPER1,GPR137B,GPR137C,GPR139,GPR141,GPR146,GPR157,GPR162,GPR176,GPR180,GPR27,GPR63,GPR88,GRM8,GRPR,HRH2,IL10RA,IL12RB1,IL15RA,IL17RC,IL17RD,IL17RE,IL18R1,IL1R1,IL20RA,IL4R,IL6ST,IL7R,ITGA1,ITGA2B,ITGA4,ITGA5,ITGA7,ITGAE,ITGB3,ITGB4,ITGB5,ITGB7,KISS1R,LGR4,LPAR1,LPAR2,LPAR3,LPAR4,LTB4R,LTB4R2,
IL-9 Signaling	1,57E00	3,43E-01	1,897	BCL3,IRS1,NFKB1,NFKB2,PIK3CD,PIK3R1,PIK3R5,REL,RELB,STAT1,STAT5A,TNF
FAT10 Signaling Pathway	1,57E00	3,09E-01	2,449	NUB1,PSMA2,PSMA3,PSMA5,PSMA6,PSMB10,PSMB8,PSMB9,PSMC4,PSMD11,PSME1,PSME2,PSMF1,SQSTM1,TNF,UBD,UBE2Z

Class A/1 (Rhodopsin-like receptors	1,56E00	2,39E-01	2,357	ACKR2,ADORA2A,ADORA2B,ADRA1A,ADRA2B,ADRB1,APLN,BDKRB2,C3,C3AR1,C5,C5AR1,CCL2,CCL20,CCL5,CCR1,CCR3,CHRM4,CNR1,CORT,CX3CL1,CXCL1,CXCL10,CXCL12,CXCL16,CXCL2,CXCL3,CXCL5,CXCL8,CXCR4,DRD4,ECE1,EDN2,EDNRB,F2RL1,GAL,GALR2,GNRH2,GPER1,GRPR,HRH2,KISS1R,LPAR1,LPAR2,LPAR3,LPAR4,LTB4R,LTB4R2,MCHR1,NMU,NPB,NPS,NPW,NTSR1,NTSR2,OPRL1,OXTR,P2RY1,P2RY11,P2RY2,PLPPR1,PLPPR3,PLPPR5,PROK2,PSAP,PTAFR,QRFPR,S1PR1,SAA1,SSTR2,SSTR3,TACR2
Netrin Signaling	1,56E00	2,56E-01	0,762	ABLIM2,ADORA2B,AKAP5,CACNA1A,CACNA1C,CACNA1E,CACNA1H,CACNA1I,CACNA1S,CACNG4,CAMK2A,CRMP1,DPYSL4,DRAXIN,ENAH,FMO1,GOSR1,GRIA1,ITPR1,ITPR3,MAPK3,NAPA,NEO1,NFAT5,NFATC2,NFATC4,NFKB1,NFKB2,PAK1,PGK1,PIK3CD,PIK3R1,PIK3R5,PLCG2,PPP3CC,PRKCA,PRKCQ,PRKD1,PRKG1,RAC2,RYR2,SNAP25,UNC5A

B-WICH complex positively regulate	1,56E00	3,19E-01	2,840	ACTB,DDX21,H2AC6,H2AZ2,H2BC11,H2BC12,H2BC17,H2BC21,H2BC26,H2BC5,KAT2A,POLR1B,POLR1G,POLR1H,POLR2H
GABAergic Receptor Signaling Path	1,54E00	2,63E-01	-1,667	ABAT,ADCY2,ADCY5,ADCY6,ADCY8,ALDH5A1,CACNA1A,CACNA1C,CACNA1E,CACNA1H,CACNA1I,CACNA1S,CACNG4,GABBR1,GABBR2,GABRB3,GABRE,GABRG3,GABRP,GAD1,GAD2,GNAI1,GNAO1,GNB4,GNG12,GNG2,GNG7,GUCY1A1,ITPR1,ITPR3,NLGN3,NRXN2,NRXN3,SLC32A1,SLC38A3,SLC6A1
Metallothioneins bind metals	1,54E00	0.5	2,236	MT1F,MT1G,MT1H,MT1X,MT2A
TCF dependent signaling in respons	1,54E00	2,51E-01	2,309	AMER1,APC,AXIN2,CAV1,CTNNBIP1,DACT1,FRAT1,FRAT2,FZD1,FZD5,H2AC6,H2AZ2,H2BC11,H2BC12,H2BC17,H2BC21,H2BC26,H2BC5,HECW1,KREMEN2,LGR4,MYC,PPP2R1B,PSMA2,PSMA3,PSMA5,PSMA6,PSMB10,PSMB8,PSMB9,PSMC4,PSMD11,PSME1,PSME2,PSMF1,RUNX3,SOX13,SOX4,SOX6,SOX7,SOX9,TCF7L1,TERT,TLE3,TLE5,TLE6,WNT3,WNT5A

Ephrin B Signaling	1,54E00	2,92E-01	-1,698	CXCL12,CXCR4,EFNB1,EFNB3,EPHB2,GNAI1,GNAO1,GNAS,GNAZ,GNB4,GNG12,GNG2,GNG7,ITSN1,KALRN,MAPK3,MRAS,PAK1,RAC2,RGS3,VAV3
CDC42 Signaling	1,54E00	2,58E-01	-1,213	APC,APC2,ARHGEF6,B2M,CDC42EP2,EXOC3L1,FGD1,FGD3,FNBP1L,HLA-A,HLA-B,HLA-C,HLA-DOB,HLA-DQA1,HLA-DRA,HLA-DRB1,HLA-DRB5,HLA-E,HLA-F,HLA-G,IQGAP2,IQGAP3,ITGA1,ITGA2B,ITGA4,ITGA5,ITGA7,ITGAE,ITGB3,ITGB4,ITGB5,ITGB7,LIMK2,MAPK11,MYL6,MYL9,MYLK,PAK1,SRC,WAS
Xenobiotic Metabolism CAR Signali	1,54E00	2,56E-01	-0,926	ABCC1,ALDH1A3,ALDH1L2,ALDH2,ALDH3A1,ALDH3B1,ALDH4A1,ALDH5A1,ALDH6A1,CHST10,CHST11,CHST15,CITED2,FMO1,GSTA4,GSTM2,GSTM3,GSTM4,GSTO1,GSTO2,GSTT2/GSTT2B,HS3ST1,HS3ST3A1,HS3ST4,HS3ST5,HSP90AA1,HSP90B1,MAP2K3,MAP2K5,MAPK3,NOS2,PPM1J,PPP2R1B,PPP2R2A,PPP2R2B,PPP2R3A,PRKCA,PRKCQ,PRKD1,SRC,UGT8,UST
Other interleukin signaling	1,53E00	3,75E-01	1,667	CSF1,CSF3,IL32,IL34,PTPRZ1,SDC1,SNAP25,STX3,STXBP2
Interleukin-6 family signaling	1,53E00	3,75E-01	2,333	CLCF1,CNTFR,IL11,IL6,IL6ST,JAK2,LIF,OSMR,STAT1

Glutathione-mediated Detoxification	1,53E00	3,75E-01	-1,414	ANPEP,GSTA4,GSTM2,GSTM3,GSTM4,GSTO1,GSTO2,GSTT2/GSTT2B,PTGES
TNFR2 non-canonical NF-kB pathw	1,53E00	2,94E-01	4,025	BIRC3,CD40,PSMA2,PSMA3,PSMA5,PSMA6,PSMB10,PSMB8,PSMB9,PSMC4,PSMD11,PSME1,PSME2,PSMF1,TNF,TNFRSF11A,TNFRSF1B,TNFSF12,TNFSF13B,TRAF3
Oxytocin Signaling Pathway	1,52E00	2,42E-01	-1,250	ABCC9,ATP2B2,CACNA1A,CACNA1C,CACNA1E,CACNA1S,CACNG4,CAMKK1,CCL5,CREB3L4,CXCL8,GAD1,GNAI1,GNAO1,GNB4,GNG12,GNG2,GNG7,GUCY1A1,GUCY1A2,IL6,ITPR1,ITPR3,JMJD7-PLA2G4B,KCNT2,LPL,MAP2K5,MAPK11,MAPK3,MAPK4,MRAS,MYH10,MYH14,MYH7B,MYL6,MYL9,MYLK,MYO10,MYO5C,NFKB1,NFKB2,NPR3,OXTR,PIK3CD,PIK3R1,PIK3R5,PLA2G4A,PLA2G4C,PLA2G6,PPARA,PPARG,PPP3CC,PRKCA,PRKCQ,PRKD1,PRKG1,PTGS2,REL,RELB,RRAS,SHC2,SHC3,SMARCC2,SOS2

p75 NTR receptor-mediated signalli	1,52E00	2,76E-01	-2,502	ABR,APH1B,ARHGEF10,ARHGEF16,ARHGEF17,ARHGEF26,ARHGEF37,ARHGEF39,ARHGEF4,ARHGEF6,FGD1,FGD3,ITSN1,KALRN,MAGED1,MCF2L,MYD88,NFKB1,NFKBIA,NGFR,OBSCN,PLEKHG2,PLEKHG5,RIPK2,SOS2,SQSTM1,VAV3
Actin Cytoskeleton Signaling	1,51E00	2,45E-01	-2,082	ACTA1,ACTA2,ACTB,ACTG1,ACTG2,APC,APC2,ARHGAP24,ARHGEF4,ARHGEF6,DIAPH2,FGD1,FGD3,FGF19,FGF22,FLNA,FN1,GNG12,IQGAP2,IQGAP3,ITGA1,ITGA2B,ITGA4,ITGA5,ITGA7,ITGAE,ITGB3,ITGB4,ITGB5,ITGB7,LIMK2,MAPK3,MATK,MRAS,MYH10,MYH14,MYH7B,MYL6,MYL9,MYLK,MYLK3,MYO10,MYO5C,PAK1,PAK6,PDGFA,PDGFB,PFN2,PIK3CD,PIK3R1,PIK3R5,RAC2,RRAS,SOS2,SSH3,VAV3,WAS
Oxidative Stress Induced Senescen	1,51E00	2,84E-01	1,091	CBX2,CBX4,CBX8,CDKN2A,CDKN2B,CDKN2C,H2AC6,H2AZ2,H2BC11,H2BC12,H2BC17,H2BC21,H2BC26,H2BC5,MAP2K3,MAPK11,MAPK3,MDM2,MOV10,PHC3,SUZ12,TNIK,TNRC6C

PIP3 activates AKT signaling	1,5E00	2,61E-01	-0,333	BTC,ERBB4,ESR1,FGF19,FGF22,FGFR2,FGFR3,FGFR4,FLT3LG,FOXO4,FOXO6,GAB1,HGF,IER3,IL1RAP,IRS1,MAPK3,MDM2,MET,MYD88,NR4A1,NRG2,PDGFA,PDGFB,PDGFRB,PHLPP1,PIK3AP1,PIK3CD,PIK3R1,PPP2R1B,PRR5,PRR5L,RAC2,RICTOR,SRC,TGFA
Reelin Signaling in Neurons	1,5E00	2,61E-01	-0,928	APBB1,APOE,ARHGEF10,ARHGEF16,ARHGEF17,ARHGEF26,ARHGEF37,ARHGEF39,ARHGEF4,ARHGEF6,CAMK2A,CDK5R1,CNR1,DAB1,DCX,HCK,ITGA5,LRP8,LYN,MAP1B,MAP2K3,MAP2K5,MAPK3,MAPK8IP1,MAPK8IP2,MAPK8IP3,MAPT,NECTIN3,PAFAH1B3,PDK2,PDK3,PDK4,PIK3CD,PIK3R1,PIK3R5,SRC
Nicotinate metabolism	1,5E00	4,12E-01	1,134	BST1,CD38,NADK,NMNAT1,NNMT,NT5E,PTGS2

Synaptic Long Term Depression	1,48E00	2,5E-01	-0,302	CACNA1A,CACNA1C,CACNA1E,CACNA1H,CACNA1I,CACNA1S,CACNG4,GAD1,GNAI1,GNAO1,GNAS,GNAZ,GRIA1,GRID2,GRM8,GUCY1A1,GUCY1A2,ITPR1,ITPR3,LYN,MAPK3,MRAS,NOS2,NPR3,PLA2G4A,PLA2G4C,PLA2G6,PLAAT2,PLAAT4,PLB1,PLCD1,PLCD3,PLCE1,PLCG2,PLCL2,PPM1J,PPP2R1B,PPP2R2A,PPP2R2B,PPP2R3A,PRKCA,PRKCQ,PRKD1,PRKG1,RRAS,RYR2,SMARCC2
Leukocyte Extravasation Signaling	1,48E00	2,51E-01	0,316	ACTA1,ACTA2,ACTB,ACTG1,ACTG2,CLDN1,CLDN5,CTNNA1,CTNND1,CXCL12,CXCR4,DLC1,GNAI1,ICAM1,ITGA4,JAM2,MAPK11,MMP14,MMP15,MMP16,MMP24,MMP25,MYL6,PIK3CD,PIK3R1,PIK3R5,PLCG2,PRKCA,PRKCQ,PRKD1,PTK2B,RAC2,RASGRP1,RASSF5,SELPLG,SRC,TEC,THY1,TIMP1,TIMP2,TIMP3,TIMP4,VAV3,VCAM1,WAS

Sertoli Cell-Sertoli Cell Junction Sig	1,48E00	2,44E-01	-1,192	A2M,ACTA1,ACTA2,ACTB,ACTG1,ACTG2,AR,CDH22,CDH24,CDH8,CGN,CLDN1,CLDN5,CREB3L4,CTNNA1,DLG1,ICAM1,IL1A,IL1R1,IL1RAP,JAM2,LAMA1,LAMA2,LAMA3,LAMA5,LAMB2,LAMB3,MAP2K3,MAP3K6,MAP3K8,MAPK11,MAPK3,MRAS,MYO7A,NECTIN2,NECTIN3,NFKB1,NFKB2,NGFR,PAK1,PAK6,PIK3CD,PIK3R1,PIK3R5,RRAS,SPTAN1,SRC,TGFB3,TIMP1,TNF,TNFRSF1B,TUBA1A,TUBB2B,TUBB3,TUBB4A,WAS,YBX3
CDK5 Signaling	1,47E00	2,68E-01	-1,000	ADCY2,ADCY5,ADCY6,ADCY8,CABLES1,CACNA1A,CDK5R1,FOSB,GNAS,GUCY1A1,LAMA1,LAMA2,LAMA3,LAMA5,LAMB3,MAPK11,MAPK3,MAPK4,MAPT,MRAS,NGFR,NTRK2,PPM1J,PPP1R1B,PPP1R3C,PPP2R1B,PPP2R2A,PPP2R2B,PPP2R3A,RRAS

Sperm Motility	1,47E00	2,43E-01	0,000	CACNA1H,CACNA1I,EPHA5,EPHB2,ERBB4,FGFR2,FGFR3,FGFR4,FLT3LG,GNAI1,GNAO1,GNAS,GNAZ,GNB4,GNG12,GNG2,GNG7,GUCY1A1,GUCY1A2,HCK,ITPR1,ITPR3,JAK2,LMTK3,LYN,MAP2K3,MAP2K5,MATK,MET,MRAS,NTRK2,PDE1A,PDE1C,PDE2A,PDGFRB,PLA2G4A,PLA2G4C,PLA2G6,PLAAT2,PLAAT4,PLB1,PLCD1,PLCD3,PLCE1,PLCG2,PLCL2,PRKCA,PRKCQ,PRKD1,PRKG1,PTK2B,RET,ROR1,ROS1,SRC,TEC,TEK,TYRO3
NFKBIE Signaling Pathway	1,46E00	2,9E-01	2,236	ANKRD44,CD40,CD70,CXCL8,DYSF,IKBKE,NFKB1,NFKB2,NFKBIA,NFKBIB,NFKBIE,NGFR,REL,RELB,TNF,TNFRSF1B,TNFSF10,TNFSF12,TNFSF13B,TNFSF9

Systemic Lupus Erythematosus in T	1,45E00	2,44E-01	3,434	B2M,CASP1,CASP4,CASP6,CASP7,CASP8,CASQ1,CD70,CREB3L4,ELF1,ESR1,FAS,GNAI1,HLA-A,HLA-B,HLA-C,HLA-DOB,HLA-DQA1,HLA-DRA,HLA-DRB1,HLA-DRB5,HLA-E,HLA-F,HLA-G,ICOSLG/LOC102723996,IL23A,IL6,ITPR1,MAP2K3,MAP2K5,MAPK3,MR1,MRAS,NFATC2,NOS2,ORAI1,PIK3CD,PIK3R1,PIK3R5,PPM1J,PPP2R1B,PPP2R2A,PPP2R2B,PPP2R3A,PPP3CC,RAC2,RHOC,RHOU,RND1,RND2,RRAS,SELPLG,SOS2,STIM1
Production of Nitric Oxide and Reac	1,44E00	2,5E-01	2,214	APOE,APOL1,CAT,IFNGR2,IKBKE,IRF1,JAK2,MAP3K6,MAP3K8,MAPK11,MAPK3,NFKB1,NFKB2,NFKBIA,NFKBIB,NFKBIE,NGFR,NOS2,PCYOX1,PIK3CD,PIK3R1,PIK3R5,PLCG2,PPARA,PPM1J,PPP1R3C,PPP2R1B,PPP2R2A,PPP2R2B,PPP2R3A,PRKCA,PRKCQ,PRKD1,RAC2,REL,RELB,RHOC,RHOU,RND1,RND2,SERPINA1,STAT1,TLR4,TNF,TNFRSF1B

PKCθ Signaling in T Lymphocytes	1,44E00	2,51E-01	2,828	CACNA1A,CACNA1C,CACNA1E,CACNA1H,CACNA1I,CACNA1S,CACNG4,CAMK2A,HLA-A,HLA-B,HLA-DOB,HLA-DQA1,HLA-DRA,HLA-DRB1,HLA-DRB5,IKBKE,ITPR1,ITPR3,LCP2,MAP3K6,MAP3K8,MAPK3,MRAS,NFAT5,NFATC2,NFATC4,NFKB1,NFKB2,NFKBIA,NFKBIB,NFKBIE,PIK3CD,PIK3R1,PIK3R5,PLCG2,PPP3CC,PRKCQ,RAC2,REL,RELB,RRAS,SOS2,VAV3
Myelination Signaling Pathway	1,43E00	2,35E-01	0,000	ACVR2A,APC,APH1B,ASCL1,BMP7,BMP8B,CNP,CREB3L4,DLC1,EGR2,ERBB4,FGFR2,FGFR3,FGFR4,FZD1,FZD5,FZD9,GLI2,HCK,HDAC5,HDAC9,HES5,ID2,ILK,IRS1,ITGB3,ITGB4,ITGB5,ITGB7,LAMA1,LAMA2,LAMA3,LAMA5,LAMB2,LAMB3,LYN,MAPK3,MRAS,MTHFD2,MYRF,NFATC2,NFATC4,NFKB1,NFKB2,NGFR,NTRK2,OLIG1,OLIG2,PDGFA,PDGFB,PDGFRB,PIK3CD,PIK3R1,PIK3R5,PLCG2,PLP1,POU3F1,PPP3CC,PTK2B,PTPRA,RRAS,SMAD9,SOS2,SOX8,SRC,WNT10A,WNT10B,WNT11,WNT2B,WNT3,WNT5A,WNT7B,WNT9B,XAF1
Choline Biosynthesis III	1,42E00	4,29E-01	0,447	CHPT1,PCYT1B,PLD1,PLD2,PLD5,PLD6

Phenylalanine Degradation IV (Mam	1,42E00	4,29E-01	0,000	ALDH2,HPD,IL4I1,MAOB,SLC27A2,SMOX
CTLA4 Signaling in Cytotoxic T Lym	1,41E00	2,47E-01	-2,832	AP1S2,B2M,CLTCL1,HCK,HLA-A,HLA-B,HLA-C,HLA-DOB,HLA-DQA1,HLA-DRA,HLA-DRB1,HLA-DRB5,HLA-E,HLA-F,HLA-G,IDO1,LCP2,LYN,MAPK11,MAPK3,MR1,MRAS,NFAT5,NFATC2,NFATC4,NFKB1,NFKB2,PIK3CD,PIK3R1,PIK3R5,PLCG2,PLD1,PLD2,PLD5,PLD6,PPM1J,PPP2R1B,PPP2R2A,PPP2R2B,PPP2R3A,PRKCQ,RAC2,RRAS,SRC,TGFB2,TGFB3,VAV3
RHOGDI Signaling	1,41E00	2,44E-01	2,921	ACTA1,ACTA2,ACTB,ACTG1,ACTG2,ARHGEF10,ARHGEF16,ARHGEF17,ARHGEF4,ARHGEF6,CDH22,CDH24,CDH8,DLC1,ESR1,GNAI1,GNAO1,GNAS,GNAZ,GNB4,GNG12,GNG2,GNG7,ITGA1,ITGA2B,ITGA4,ITGA5,ITGA7,ITGAE,ITGB3,ITGB4,ITGB5,ITGB7,LIMK2,MRAS,MYH10,MYH14,MYH7B,MYL6,MYL9,MYO10,MYO5C,PAK1,PAK6,PIP5KL1,PRKCA,RAC2,RHOC,RHOU,RND1,RND2,SRC

Role of Tissue Factor in Cancer	1,4E00	2,46E-01	2,021	ACVR2A,CCN1,CCN2,CSF1,CXCL1,CXCL8,F2RL1,FLNC,HCK,HGF,IKBKE,IL1B,ITGB3,JAK2,LIMK2,LYN,MAP2K3,MAP2K5,MAPK11,MAPK3,MAPK4,MET,MRAS,NFKB1,NFKB2,NGFR,PIK3CD,PIK3R1,PIK3R5,PLAUR,PPM1J,PPP2R2A,PPP2R3A,PRKCA,PTGS2,PTK2B,RRAS,SRC,STAT5A,TFPI,TGFA,TGFB2,TGFB3,TGFBR3,TNF,TNFRSF1B,VEGFC,ZEB1
PTEN Signaling	1,37E00	2,53E-01	-0,577	CCND1,FGFR2,FGFR3,FGFR4,FOXO4,FOXO6,GHR,IKBKE,ILK,INPPL1,ITGA1,ITGA2B,ITGA4,ITGA5,ITGA7,ITGAE,ITGB3,ITGB4,ITGB5,ITGB7,MAGI2,MAPK3,MRAS,NFKB1,NFKB2,NGFR,NTRK2,PDGFRB,PIK3CD,PIK3R1,PIK3R5,RAC2,REL,RELB,RRAS,SOS2,TGFBR3,TNFRSF11A
Cell surface interactions at the vasc	1,37E00	2,55E-01	1,667	ANGPT1,ATP1B1,ATP1B2,CAV1,CD47,CD74,CEACAM1,COL1A1,CXADR,EPCAM,ESAM,FN1,GRB14,ITGA4,ITGA5,ITGB3,JAM2,L1CAM,LYN,PIK3R1,PROCR,PROS1,SDC1,SDC2,SDC3,SDC4,SELPLG,SLC16A3,SLC3A2,SLC7A11,SLC7A5,SLC7A6,SRC,TEK,TNFRSF10A,TNFRSF10D

IL-8 Signaling	1,37E00	2,44E-01	1,581	ANGPT1,CCND1,CCND2,CCND3,CXCL1,CXCL8,GNAI1,GNAO1,GNAS,GNAZ,GNB4,GNG12,GNG2,GNG7,ICAM1,IKBKE,IRAK2,ITGB3,LASP1,LIMK2,MAPK3,MRAS,MYL9,NFKB1,NFKBIA,NFKBIB,NFKBIE,PGF,PIK3CD,PIK3R1,PIK3R5,PLD1,PLD2,PLD5,PLD6,PRKCA,PRKCQ,PRKD1,PTGS2,PTK2B,RAC2,RHOC,RHOU,RND1,RND2,RRAS,SRC,TEK,VCAM1,VEGFC
Transport of inorganic cations/anion	1,36E00	2,67E-01	0,577	CTNS,SLC12A6,SLC12A7,SLC15A3,SLC1A2,SLC1A5,SLC24A1,SLC24A3,SLC25A22,SLC26A11,SLC32A1,SLC34A2,SLC38A3,SLC38A5,SLC3A2,SLC43A2,SLC4A3,SLC4A4,SLC4A8,SLC6A15,SLC6A20,SLC6A6,SLC7A11,SLC7A2,SLC7A5,SLC7A6,SLC9A6
Erythrocytes take up carbon dioxide	1,35E00	4,55E-01	-0,447	AQP1,CA2,CA4,CYB5R2,CYB5RL
NAD biosynthesis II (from tryptopha	1,35E00	4,55E-01	0,447	IDO1,KYNU,NMNAT1,QPRT,TDO2
Regulation of the Epithelial Mesenc	1,34E00	2,74E-01	0,447	APC,APH1B,AXIN2,FZD1,FZD5,FZD9,GLI2,LOX,NFKB1,NFKB2,PTCH1,REL,RELB,SNAI2,TCF7L1,WNT10A,WNT10B,WNT11,WNT2B,WNT3,WNT5A,WNT7B,WNT9B

Paxillin Signaling	1,34E00	2,64E-01	-1,147	ACTA1,ACTA2,ACTB,ACTG1,ACTG2,ARHGEF6,ITGA1,ITGA2B,ITGA4,ITGA5,ITGA7,ITGAE,ITGB3,ITGB4,ITGB5,ITGB7,MAPK11,MRAS,PAK1,PAK6,PIK3CD,PIK3R1,PIK3R5,PTK2B,PTPN12,RRAS,SOS2,SRC
Adipogenesis pathway	1,33E00	2,56E-01	0,000	ACVR2A,BMP7,CEBPB,CEBPD,DDIT3,DLK1,EGR2,FGFR2,FGFR3,FGFR4,FZD1,FZD5,FZD9,HDAC5,HDAC9,KAT2A,KAT6B,KAT7,KLF5,LPL,NFATC4,NOCT,NR1D1,PPARG,PPIP5K1,RBP1,SIRT2,SLC2A4,SMAD9,SOX9,TNF,TXNIP,WNT10B,WNT5A
Cardiac β-adrenergic Signaling	1,33E00	2,47E-01	-1,890	ADCY2,ADCY5,ADCY6,ADCY8,ADRB1,AKAP12,AKAP5,AKAP6,CACNA1A,CACNA1C,CACNA1E,CACNA1S,CACNG4,ENPP1,GDPD1,GNAI1,GNAO1,GNAS,GNAZ,GNB4,GNG12,GNG2,GNG7,GUCY1A1,MPPED2,MRAS,PDE10A,PDE1A,PDE1C,PDE2A,PDE3A,PDE6D,PDE8B,PDE9A,PKIA,PLD6,PPM1J,PPP1R3C,PPP2R1B,PPP2R2A,PPP2R2B,PPP2R3A,RYR2
Vitamin-C Transport	1,33E00	3,64E-01	1,414	GLRX,GSTO1,GSTO2,NXN,SELENOT,SLC2A4,STOM,TXNDC2

Transcriptional regulation of granulo	1,32E00	3,04E-01	2,138	CEBPB,GATA2,H2AC6,H2AZ2,H2BC11,H2BC12,H2BC17,H2BC21,H2BC26,H2BC5,KLF5,MYC,PML,RUNX1
WNT ligand biogenesis and trafficki	1,31E00	3,46E-01	-0,333	WLS,WNT10A,WNT10B,WNT11,WNT2B,WNT3,WNT5A,WNT7B,WNT9B
T Cell Receptor Signaling	1,31E00	2,44E-01	3,618	B2M,DUSP5,HLA-A,HLA-B,HLA-C,HLA-DOB,HLA-DQA1,HLA-DRA,HLA-DRB1,HLA-DRB5,HLA-E,HLA-F,HLA-G,ICAM1,ICOSLG/LOC102723996,IKBKE,LCP2,MAP2K3,MAPK11,MAPK3,MR1,MRAS,NFAT5,NFATC2,NFATC4,NFKB1,NFKB2,NFKBIA,NFKBIB,NFKBIE,PAG1,PIK3CD,PIK3R1,PIK3R5,PLCG2,PPP3CC,PRKCQ,PTK2B,RASGRP1,REL,RELB,RRAS,SHC3,SOS2,TCF7L1,TNF,VAV3
T Cell Exhaustion Signaling Pathwa	1,31E00	2,47E-01	1,890	ACVR2A,CD274,FOXP1,HLA-A,HLA-B,HLA-C,HLA-DOB,HLA-DQA1,HLA-DRA,HLA-DRB1,HLA-DRB5,HLA-E,HLA-F,HLA-G,IL10RA,IL12RB1,IL6,IRF9,JAK2,LGALS9,MAPK3,MRAS,NFAT5,NFATC2,NFATC4,PDCD1LG2,PIK3CD,PIK3R1,PIK3R5,PLCG2,PPM1J,PPP2R1B,PPP2R2A,PPP2R2B,PPP2R3A,PRDM1,PRKCQ,RRAS,STAT1,STAT2,TGFBR3,TNFRSF14

PRC2 methylates histones and DN/	1,3E00	3,33E-01	1,897	DNMT3B,H2AC6,H2AZ2,H2BC11,H2BC12,H2BC17,H2BC21,H2BC26,H2BC5,SUZ12
Angiopoietin Signaling	1,3E00	2,76E-01	-1,291	ANGPT1,ANGPTL1,GRB14,IKBKE,MRAS,NFKB1,NFKB2,NFKBIA,NFKBIB,NFKBIE,PAK1,PAK6,PIK3CD,PIK3R1,PIK3R5,REL,RELB,RRAS,STAT5A,TEK,TNIP1
Macropinocytosis Signaling	1,3E00	2,76E-01	1,291	CSF1,HGF,ITGA5,ITGB3,ITGB4,ITGB5,ITGB7,MET,MRAS,PAK1,PDGFA,PDGFB,PIK3CD,PIK3R1,PIK3R5,PLCG2,PRKCA,PRKCQ,PRKD1,RRAS,SRC
Transcriptional Regulation by NPAS	1,3E00	3,24E-01	-1,265	ARNT2,CDK5R1,KCNIP3,MAGED1,MAPK3,MDM2,MOV10,NAMPT,NPAS4,RET,TNRC6C
p53 Signaling	1,3E00	2,65E-01	0,447	BBC3,CASP6,CCND1,CCND2,CDKN2A,COQ8A,DRAM1,FAS,GADD45G,HDAC9,MDM2,PERP,PIK3CD,PIK3R1,PIK3R5,PMAIP1,PML,PPP1R13B,RPRM,SCO2,SERPINE2,SNAI2,THBS1,TNFRSF10A,TP53INP1,TP63

Supplementary Table 7: Ingenuity pathway analysis of DEGs in EPEA vs Control condition (p<0.05, z-score>|2|)

Ingenuity Canonical Pathways	-log(p-value)	Ratio	z-score	Molecules
Integrin cell surface interactions	8,11E00	1,71E-01	-3,207	CDH1,COL1A2,COL3A1,COL5A1,COL5A2,COL6A2,COL6A3,FBN1,FN1,ICAM1,ITGA5,ITGB3,VCAM1,VWF
Elastic fibre formation	7,29E00	2,33E-01	-2,530	EMILIN1,EMILIN2,FBN1,FBN2,FN1,ITGA5,ITGB3,LOXL2,MFAP4,MFAP5
Assembly of collagen fibrils and other multimeric structures	6,9E00	1,86E-01	-2,111	COL12A1,COL14A1,COL1A2,COL3A1,COL5A1,COL5A2,COL6A2,COL6A3,LOXL2,PCOLCE,PXDN
Collagen biosynthesis and modifying enzymes	6,32E00	1,64E-01	-2,111	ADAMTS2,COL12A1,COL14A1,COL1A2,COL25A1,COL3A1,COL5A1,COL5A2,COL6A2,COL6A3,PCOLCE
Collagen chain trimerization	6,11E00	2,05E-01	-1,667	COL12A1,COL14A1,COL1A2,COL25A1,COL3A1,COL5A1,COL5A2,COL6A2,COL6A3
Pathogen Induced Cytokine Storm Signaling Pathway	5,93E00	7,57E-02	-3,545	CCR1,CDH1,CGAS,COL12A1,COL1A2,COL25A1,COL3A1,COL5A1,COL5A2,COL6A2,COL6A3,CXCL12,HLA-DMB,HLA-DPB1,HLA-DQB2,HLA-DRB5,IL1B,IL1R1,PRDM1,RYR2,SRGN,TLR4,VEGFC

Extracellular matrix organization	5,89E00	1,24E-01	-3,051	BCAN,BGN,COL1A2,COL3A1,COL5A1,COL5A2,COL6A2,COL6A3,FN1,ITGA5,ITGB3,SERPINE1,TNXB
Syndecan interactions	5,61E00	2,59E-01	-2,646	COL1A2,COL3A1,COL5A1,COL5A2,FN1,ITGB3,SDC4
Collagen degradation	5,19E00	1,61E-01	-1,667	COL12A1,COL14A1,COL1A2,COL25A1,COL3A1,COL5A1,COL5A2,COL6A2,COL6A3
Pulmonary Fibrosis Idiopathic Signaling Pathway	4,66E00	6,71E-02	-2,524	ACTG2,ACVR2A,AXL,CCN4,CDH1,COL12A1,COL1A2,COL25A1,COL3A1,COL5A1,COL5A2,COL6A2,COL6A3,CXCL12,FN1,IL1B,SERPINE1,STAT6,WNT11,WNT5A,WNT5B
Response to elevated platelet cytosolic Ca2+	4,35E00	9,6E-02	-2,887	A2M,ECM1,FN1,IGF2,ITGB3,MMRN1,SERPINA3,SERPINE1,SRGN,TOR4A,VEGFC,VWF
Osteoarthritis Pathway	4,09E00	7,27E-02	-2,138	ALPL,CASP10,CASP4,CCN4,DKK1,DLX5,EPAS1,FN1,GREM1,IL1B,IL1R1,ITGA5,ITGB3,SDC4,TLR4,VEGFC
Coagulation System	4,02E00	1,88E-01	0,816	A2M,F3,PLAUR,SERPINE1,TFPI,VWF
Role of Osteoclasts in Rheumatoid Arthritis Signaling Pathway	3,88E00	6,47E-02	-2,357	CAMK4,CDH1,COL12A1,COL1A2,COL25A1,COL3A1,COL5A1,COL5A2,COL6A2,COL6A3,IL1B,IL1R1,ITGA5,ITGB3,RAC2,SFRP4,TLR4,VAV3

IL-4 Signaling	3,83E00	7,57E-02	-3,207	COL12A1,COL1A2,COL25A1,COL3A1,COL5A1,COL5A2,COL6A2,COL6A3,HLA-DMB,HLA-DPB1,HLA-DQB2,HLA-DRB5,STAT6,TGM2
IL-10 Signaling	3,78E00	8,39E-02	1,732	CCN4,CCR1,HLA-DMB,HLA-DPB1,HLA-DQB2,HLA-DRB5,HMOX1,ICAM1,IL1B,IL1R1,PRDM1,TLR4
Transcriptional Regulatory Network in Embryonic Stem Cells	3,28E00	7,41E-02	0,000	ACVR2A,HOXB1,IGF2,INHBA,ISL1,NOG,RFX4,TBX3,WNT11,WNT5A,WNT5B,ZIC3
GP6 Signaling Pathway	3,28E00	8,47E-02	-2,333	COL12A1,COL1A2,COL25A1,COL3A1,COL5A1,COL5A2,COL6A2,COL6A3,ITGB3,VAV3
Degradation of the extracellular matrix	3,27E00	1,17E-01	-1,890	A2M,ADAMTS9,BCAN,CDH1,FBN1,FBN2,FN1
Neuroinflammation Signaling Pathway	3,23E00	5,9E-02	-1,155	ACVR2A,CXCL12,GABRQ,HLA-DMB,HLA-DPB1,HLA-DQB2,HLA-DRB5,HMOX1,ICAM1,IL1B,IL1R1,JMJD7-PLA2G4B,NFATC4,RAC2,SLC1A2,TLR4,VCAM1
RHOGDI Signaling	3,21E00	6,57E-02	1,414	ACTG2,CDH1,CDH13,CDH6,DLC1,GNG2,GNG4,ITGA5,ITGB3,MYH3,MYL9,MYO18A,PAK3,RAC2

ILK Signaling	3,14E00	6,77E-02	-1,897	ACTG2,CDH1,DSP,FLNC,FN1,ITGB3,KRT18,MYH3,MYL9,MYO18A,RAC2,SNAI2,VEGFC
Beta-catenin independent WNT signaling	3,13E00	8,82E-02	-0,333	GNG2,GNG4,ITPR3,RAC2,ROR1,ROR2,WNT11,WNT5A,WNT5B
Wound Healing Signaling Pathway	3,07E00	6,36E-02	-2,673	ACVR2A,COL12A1,COL1A2,COL25A1,COL3A1,COL5A1,COL5A2,COL6A2,COL6A3,FN1,IL1B,IL1R1,SNAI2,VEGFC
Inflammasome pathway	2,94E00	0.2	-2,000	IL1B,NEK7,NLRP1,TLR4
Tumor Microenvironment Pathway	2,62E00	6,55E-02	-3,162	COL1A2,COL3A1,CXCL12,FGF5,FN1,ICAM1,IGF2,IL1B,ITGA5,ITGB3,VEGFC
Signaling by PDGF	2,6E00	1,03E-01	-2,449	COL3A1,COL5A1,COL5A2,COL6A2,COL6A3,STAT6
Activin Inhibin Signaling Pathway	2,59E00	6,15E-02	-1,155	ACVR2A,CDH1,COL1A2,COL3A1,GATA2,IL1B,IL1R1,INHBA,PMEPA1,SERPINE1,SNAI2,TLR4
Dilated Cardiomyopathy Signaling Pathway	2,57E00	6,85E-02	0,378	ACTG2,CACNG7,CAMK4,ITPR3,MYH3,MYL9,MYO18A,PDE3A,RYR2,TNNT1
NCAM signaling for neurite out-growth	2,52E00	0.1	-2,449	COL3A1,COL5A1,COL5A2,COL6A2,COL6A3,SPTBN5
Hepatic Fibrosis Signaling Pathway	2,42E00	4,73E-02	-2,828	ACVR2A,CACNG7,CNR1,COL1A2,COL3A1,ICAM1,IL1B,IL1R1,ITGA5,ITGB3,MYL9,RAC2,SERPINE1,TLR4,VCAM1,VEGFC,WNT11,WNT5A,WNT5B

Calcium Signaling	2,41E00	5,85E-02	-2,121	ATP2A1,CACNG7,CAMK4,CHRNA9,ITPR3,MYH3,MYL9,MYO18A,NFATC4,RYR2,SLC8A1,TNNT1
O-linked glycosylation	2,39E00	7,48E-02	-2,828	ADAMTS2,ADAMTS9,ADAMTSL1,B3GNT9,GALNT5,GALNT6,MUC16,ST3GAL1
Semaphorin interactions	2,38E00	9,38E-02	-0,816	CRMP1,MYL9,PAK3,PLXND1,SEMA6D,SEMA7A
Role of Osteoblasts in Rheumatoid Arthritis Signaling Pathway	2,31E00	5,69E-02	-1,508	ACVR2A,ALPL,CXCL12,DKK1,DLX5,IL1B,SFRP4,STAT6,VEGFC,WNT11,WNT5A,WNT5B
Interferon gamma signaling	2,3E00	7,95E-02	-1,134	HLA-DPB1,HLA-DQB2,HLA-DRB5,ICAM1,MT2A,TRIM2,VCAM1
Neutrophil Extracellular Trap Signaling Pathway	2,28E00	4,79E-02	0,728	CASP10,CASP4,CCR1,COL12A1,COL1A2,COL25A1,COL3A1,COL5A1,COL5A2,COL6A2,COL6A3,IL1B,ITPR3,MT-ND3,PLAAT1,RAC2,TLR4
Regulation of Insulin-like Growth Factor (IGF) transport and uptake by IGFBPs	2,2E00	6,96E-02	-1,414	FBN1,FN1,IGF2,IGFBP4,IGFBP5,PAPPA,STC2,VWA1
Calcium-induced T Lymphocyte Apoptosis	2,19E00	8,57E-02	-2,000	ATP2A1,HLA-DMB,HLA-DPB1,HLA-DQB2,HLA-DRB5,ITPR3
Formation of Fibrin Clot (Clotting Cascade)	2,17E00	1,25E-01	-2,000	A2M,F3,TFPI,VWF
Multiple Sclerosis Signaling Pathway	2,09E00	5,56E-02	-2,714	HLA-DMB,HLA-DPB1,HLA-DQB2,HLA-DRB5,IL1B,IL7R,MASP1,NLRP1,PARP8,SLC8A1,TLR4

GPVI-mediated activation cascade	2,08E00	1,18E-01	-1,000	COL1A2,PDPN,RAC2,VAV3
TR/RXR Activation	2,07E00	6,61E-02	-2,828	ATP2A1,CAMK4,COL6A3,ITGA5,ITGB3,RAB3B,SLC16A3,VEGFC
HOTAIR Regulatory Pathway	2,06E00	6,12E-02	-1,414	CDH1,COL1A2,COL3A1,ICAM1,SNAI2,TLR4,WNT11,WNT5A,WNT5B
Actin Cytoskeleton Signaling	1,98E00	5,15E-02	-0,707	ACTG2,ARHGAP24,FGF5,FN1,ITGA5,ITGB3,MYH3,MYL9,MYO18A,PAK3,RAC2,VAV3
IL-8 Signaling	1,98E00	5,37E-02	-1,667	CDH1,GNG2,GNG4,HMOX1,ICAM1,ITGB3,MYL9,RAC2,TEK,VCAM1,VEGFC
Th2 Pathway	1,95E00	6,3E-02	-2,646	ACVR2A,CCR1,HLA-DMB,HLA-DPB1,HLA-DQB2,HLA-DRB5,ICAM1,STAT6
IL-17A Signaling in Fibroblasts	1,89E00	7,41E-02	-1,633	COL1A2,COL3A1,CXCL12,FN1,IL1B,VCAM1
PCP (Planar Cell Polarity) Pathway	1,88E00	8,47E-02	-1,000	CELSR1,ROR2,WNT11,WNT5A,WNT5B
Interleukin-4 and Interleukin-13 signaling	1,87E00	6,6E-02	-1,134	COL1A2,FN1,HMOX1,ICAM1,IL1B,STAT6,VCAM1
Signaling by VEGF	1,85E00	6,54E-02	-0,816	AXL,CYBA,ITGB3,ITPR3,PAK3,VAV3,VEGFC
Dendritic Cell Maturation	1,81E00	5,29E-02	-3,162	COL1A2,COL3A1,HLA-DMB,HLA-DPB1,HLA-DQB2,HLA-DRB5,ICAM1,IL1B,LTBR,TLR4
Pyroptosis Signaling Pathway	1,75E00	6,9E-02	-2,449	CASP4,IL1B,IL1R1,NEK7,NLRP1,TLR4

Phospholipase C Signaling	1,73E00	4,59E-02	-1,414	AHNAK,CAMK4,GNG2,GNG4,HMOX1,ITGA5,ITGB3,ITPR3,JMJD7-PLA2G4B,MYL9,NFATC4,RAC2,TGM2
Human Embryonic Stem Cell Pluripotency	1,72E00	5,13E-02	-0,632	ACVR2A,FUT4,INHBA,NOG,NTRK2,TBX3,WNT11,WNT5A,WNT5B,ZIC3
TEC Kinase Signaling	1,72E00	5,13E-02	0,000	ACTG2,GNG2,GNG4,ITGA5,ITGB3,PAK3,RAC2,STAT6,TLR4,VAV3
Cell surface interactions at the vascular wall	1,7E00	5,67E-02	-2,828	COL1A2,FN1,ITGA5,ITGB3,PSG4,SDC4,SLC16A3,TEK
Semaphorin Neuronal Repulsive Signaling Pathway	1,7E00	5,67E-02	0,000	BCAN,CRMP1,ITGA5,ITGB3,MYL9,PAK3,PLXND1,SEMA6D
Interleukin-10 signaling	1,69E00	9,09E-02	-2,000	CCR1,ICAM1,IL1B,IL1R1
GPER1 signaling	1,69E00	9,09E-02	0,000	FN1,GNG2,GNG4,ITGA5
Cell junction organization	1,69E00	6,67E-02	-2,449	CDH1,CDH13,CDH6,CLDN11,FLNC,SDK2
Acute Phase Response Signaling	1,66E00	5,26E-02	-1,633	A2M,FN1,HMOX1,IL1B,IL1R1,SERPINA3,SERPINE1,SERPINF1,VWF
PKCθ Signaling in T Lymphocytes	1,66E00	5,26E-02	-1,342	CACNG7,HLA-DMB,HLA-DPB1,HLA-DQB2,HLA-DRB5,ITPR3,NFATC4,RAC2,VAV3
Binding and Uptake of Ligands by Scavenger Receptors	1,66E00	8,89E-02	-1,000	COL1A2,COL3A1,COLEC12,MASP1
L1CAM interactions	1,62E00	5,88E-02	-0,378	ANK1,ANK3,CD24,DNM3,ITGA5,ITGB3,SPTBN5

ROBO SLIT Signaling Pathway	1,6E00	5,83E-02	0,378	CDH1,MYL9,NRXN3,PAK3,SLIT3,SRGAP1,VEGFC
Immunoregulatory interactions between a Lymphoid and a non-Lymphoid cell	1,55E00	5,69E-02	-1,890	CDH1,COL1A2,COL3A1,COLEC12,ICAM1,MICB,VCAM1
Leukocyte Extravasation Signaling	1,55E00	5,03E-02	0,000	ACTG2,CLDN11,CXCL12,CYBA,DLC1,ICAM1,RAC2,VAV3,VCAM1
Xenobiotic Metabolism AHR Signaling Pathway	1,54E00	6,94E-02	-2,236	ALDH3A1,CYP1B1,IL1B,MGST1,NQO1
Post-translational protein phosphorylation	1,51E00	6,06E-02	-0,816	FBN1,FN1,IGFBP4,IGFBP5,STC2,VWA1
S100 Family Signaling Pathway	1,5E00	3,55E-02	-1,400	AHNAK,CACNG7,CCR1,CDH1,CELSR1,CNR1,DLC1,GPR155,GPRC5A,IL1B,ITPR3,JMJD7-PLA2G4B,MC1R,NTRK2,OXTR,RAC2,RGR,RYR2,SERPINF1,TLR4,VCAM1,VEGFC,WNT11,WNT5A,WNT5B
Cardiac conduction	1,45E00	5,43E-02	-2,236	ATP1A2,ATP2A1,CACNG7,ITPR3,MME,RYR2,SLC8A1
Apoptotic execution phase	1,45E00	7,69E-02	-1,000	CDH1,DSP,H1-0,PKP1
Oxytocin in Brain Signaling Pathway	1,42E00	4,76E-02	1,667	CACNG7,GNG2,GNG4,IL1B,ITPR3,NLRP1,OXTR,PLAAT1,TLR4
CSDE1 Signaling Pathway	1,4E00	7,41E-02	-1,000	FN1,SDC4,SNAI2,SYNCRIP
Cardiac Hypertrophy Signaling (Enhanced)	1,4E00	3,7E-02	-2,500	ACVR2A,ATP2A1,CACNG7,FGF5,GNG2,GNG4,IL1B,IL1R1,IL7R,ITGA5,ITGB3,ITPR3,NFATC4,PDE3A,PDE8B,RYR2,WNT11,WNT5A,WNT5B

Glycosaminoglycan metabolism	1,39E00	6,33E-02	-1,342	BCAN,BGN,PAPSS2,SDC4,ST3GAL1
Signaling by MET	1,39E00	6,33E-02	-2,236	COL1A2,COL3A1,COL5A1,COL5A2,FN1
Crosstalk between Dendritic Cells and Natural Killer Cells	1,39E00	6,33E-02	-2,000	ACTG2,HLA-DRB5,LTBR,MICB,TLR4
Regulation of Actin-based Motility by Rho	1,38E00	5,66E-02	-0,447	ACTG2,ITGA5,ITGB3,MYL9,PAK3,RAC2
Role of Tissue Factor in Cancer	1,35E00	4,62E-02	-1,667	ACVR2A,F3,FGF5,FLNC,IL1B,ITGB3,PLAUR,TFPI,VEGFC
Macrophage Alternative Activation Signaling Pathway	1,33E00	4,79E-02	-2,121	EPAS1,HLA-DMB,HLA-DPB1,HLA-DQB2,HLA-DRB5,IL1B,STAT6,TLR4
Netrin Signaling	1,31E00	4,76E-02	-2,121	ABLIM3,CACNG7,CRMP1,ITPR3,NFATC4,RAC2,RYR2,STX1B
Signaling by Rho Family GTPases	1,31E00	4,21E-02	-1,134	ACTG2,CDH1,CDH13,CDH6,GNG2,GNG4,ITGA5,ITGB3,MYL9,PAK3,RAC2
Th1 Pathway	1,3E00	5,41E-02	-2,449	HLA-DMB,HLA-DPB1,HLA-DQB2,HLA-DRB5,ICAM1,NFATC4
WNT/β-catenin Signaling	1,3E00	4,73E-02	0,000	ACVR2A,CDH1,DKK1,KREMEN2,SFRP4,WNT11,WNT5A,WNT5B
Regulation of the Epithelial Mesenchymal Transition in Development Pathway	1,3E00	5,95E-02	-0,447	CDH1,SNAI2,WNT11,WNT5A,WNT5B

Supplementary Table 8: Ingenuity pathway analysis of DEGs in DHEA vs Control condition (p<0.05, z-score>|2|)

Ingenuity Canonical Pathways	-log(p-value)	Ratio	z-score	Molecules
Extracellular matrix organization	7,43E00	1,33E-01	-2,673	BCAN,BGN,COL1A2,COL3A1,COL5A1,COL5A2,COL6A2,COL6A3,DMD,FN1,ITGB3,LUM,SERPINE1,TNXB
Pathogen Induced Cytokine Stor	6,97E00	7,57E-02	-3,545	CDH1,CGAS,COL1A2,COL25A1,COL3A1,COL5A1,COL5A2,COL6A2,COL6A3,CXCL12,GSDMD,HLA-DMB,HLA-DPB1,HLA-DQB1,HLA-DRB5,IL1B,IL1R1,PRDM1,RYR2,SRGN,TLR4,TNFRSF1B,VEGFC
Integrin cell surface interactions	6,91E00	1,46E-01	-3,464	CDH1,COL1A2,COL3A1,COL5A1,COL5A2,COL6A2,COL6A3,FBN1,FN1,ITGA10,ITGB3,LUM
Assembly of collagen fibrils and	6,47E00	1,69E-01	-1,897	COL14A1,COL1A2,COL3A1,COL5A1,COL5A2,COL6A2,COL6A3,LOXL2,PCOLCE,PXDN
Elastic fibre formation	5,6E00	1,86E-01	-2,121	EMILIN1,EMILIN2,FBN1,FBN2,FBN3,FN1,ITGB3,LOXL2
Collagen chain trimerization	5,52E00	1,82E-01	-1,414	COL14A1,COL1A2,COL25A1,COL3A1,COL5A1,COL5A2,COL6A2,COL6A3
Collagen biosynthesis and modif	5,01E00	1,34E-01	-1,667	COL14A1,COL1A2,COL25A1,COL3A1,COL5A1,COL5A2,COL6A2,COL6A3,PCOLCE

Syndecan interactions	4,8E00	2,22E-01	-2,449	COL1A2,COL3A1,COL5A1,COL5A2,FN1,ITGB3
Collagen degradation	4,71E00	1,43E-01	-1,414	COL14A1,COL1A2,COL25A1,COL3A1,COL5A1,COL5A2,COL6A2,COL6A3
Degradation of the extracellular r	4,49E00	1,33E-01	-1,414	A2M,ADAMTS9,BCAN,CDH1,FBN1,FBN2,FBN3,FN1
Pulmonary Fibrosis Idiopathic Si	4,48E00	6,07E-02	-2,828	ACVR2A,AXL,CCN4,CDH1,COL1A2,COL25A1,COL3A1,COL5A1,COL5A2,COL6A2,COL6A3,CXCL12,FN1,IL11RA,IL1B,SERPINE1,STAT6,WNT5A,WNT5B
Response to elevated platelet cy	4,22E00	8,8E-02	-2,714	A2M,ECM1,FN1,IGF2,ITGB3,MMRN1,SERPINA3,SERPINE1,SRGN,TOR4A,VEGFC
Role of Osteoclasts in Rheumato	3,6E00	5,76E-02	-2,000	CDH1,COL1A2,COL25A1,COL3A1,COL5A1,COL5A2,COL6A2,COL6A3,IL1B,IL1R1,ITGB3,RAC2,SFRP4,TLR4,TNFRSF1B,VAV3
Coagulation System	3,32E00	1,56E-01	0,447	A2M,F3,PLAUR,SERPINE1,TFPI
IL-4 Signaling	3,29E00	6,49E-02	-2,887	COL1A2,COL25A1,COL3A1,COL5A1,COL5A2,COL6A2,COL6A3,HLA-DMB,HLA-DPB1,HLA-DQB1,HLA-DRB5,STAT6
RHOGDI Signaling	3,27E00	6,1E-02	1,633	CDH1,CDH13,CDH6,DLC1,GNAO1,GNG2,ITGA10,ITGB3,MYH3,MYL9,MYO18A,PAK3,RAC2

Wound Healing Signaling Pathw	3,14E00	5,91E-02	-2,496	ACVR2A,COL1A2,COL25A1,COL3A1,COL5A1,COL5A2,COL6A2,COL6A3,FN1,IL1B,IL1R1,TNFRSF1B,VEGFC
Osteoarthritis Pathway	3,14E00	5,91E-02	-1,265	CASP10,CCN4,DKK1,DLX5,FN1,GREM1,IL1B,IL1R1,ITGA10,ITGB3,TLR4,TNFRSF1B,VEGFC
GP6 Signaling Pathway	3,09E00	7,63E-02	-2,121	COL1A2,COL25A1,COL3A1,COL5A1,COL5A2,COL6A2,COL6A3,ITGB3,VAV3
Activin Inhibin Signaling Pathwa	3,09E00	6,15E-02	-1,155	ACVR2A,CDH1,COL1A2,COL3A1,GATA2,IL1B,IL1R1,INHBA,PMEPA1,SERPINE1,TLR4,TNFRSF1B
IL-10 Signaling	3,08E00	6,99E-02	1,897	CCN4,HLA-DMB,HLA-DPB1,HLA-DQB1,HLA-DRB5,HMOX1,IL1B,IL1R1,PRDM1,TLR4
Dilated Cardiomyopathy Signalin	3,01E00	6,85E-02	0,707	ADCY9,CACNG7,DMD,ITPR3,MYH3,MYL9,MYO18A,PDE3A,RYR2,TNNT1
Neuroinflammation Signaling Pa	2,97E00	5,21E-02	-1,897	ACVR2A,CXCL12,GABRE,GABRQ,HLA-DMB,HLA-DPB1,HLA-DQB1,HLA-DRB5,HMOX1,IL1B,IL1R1,PSEN2,RAC2,SLC1A2,TLR4
Signaling by PDGF	2,9E00	1,03E-01	-2,449	COL3A1,COL5A1,COL5A2,COL6A2,COL6A3,STAT6

Beta-catenin independent WNT :	2,88E00	7,84E-02	-1,414	GNAO1,GNG2,ITPR3,RAC2,ROR1,ROR2,WNT5A,WNT5B
Semaphorin interactions	2,67E00	9,38E-02	-0,816	CRMP1,MYL9,PAK3,PLXND1,SEMA6D,SEMA7A
GABAergic Receptor Signaling F	2,64E00	6,57E-02	0,333	ADCY9,CACNG7,GABRE,GABRQ,GNAO1,GNG2,ITPR3,NRXN3,SLC12A5
ILK Signaling	2,63E00	5,73E-02	-2,121	CDH1,DSP,FLNC,FN1,ITGB3,KRT18,MYH3,MYL9,MYO18A,RAC2,VEGFC
Regulation of Insulin-like Growth	2,55E00	6,96E-02	-1,414	C4A/C4B,FBN1,FN1,IGF2,IGFBP4,IGFBP5,PAPPA,STC2
Acute Phase Response Signaling	2,5E00	5,85E-02	-1,890	A2M,C4A/C4B,FN1,HMOX1,IL1B,IL1R1,SERPINA3,SERPINE1,SERPINF1,TNFRSF1B
Hepatic Fibrosis Signaling Pathw	2,33E00	4,23E-02	-3,357	ACVR2A,CACNG7,CNR1,COL1A2,COL3A1,IL1B,IL1R1,ITGA10,ITGB3,MYL9,RAC2,SERPINE1,TLR4,TNFRSF1B,VEGFC,WNT5A,WNT5B
Signaling by MET	2,22E00	7,59E-02	-2,449	COL1A2,COL3A1,COL5A1,COL5A2,FN1,MUC20
Dendritic Cell Maturation	2,2E00	5,29E-02	-3,162	COL1A2,COL3A1,HLA-DMB,HLA-DPB1,HLA-DQB1,HLA-DRB5,IL1B,LTBR,TLR4,TNFRSF1B
Transcriptional Regulatory Netwo	2,16E00	5,56E-02	-0,333	ACVR2A,IGF2,INHBA,ISL1,RFX4,TBX3,WNT5A,WNT5B,ZIC3
Signaling by VEGF	2,15E00	6,54E-02	-0,816	AXL,CYBA,ITGB3,ITPR3,PAK3,VAV3,VEGFC

NCAM signaling for neurite out-g	2,09E00	8,33E-02	-2,236	COL3A1,COL5A1,COL5A2,COL6A2,COL6A3
Multiple Sclerosis Signaling Path	2,06E00	5,05E-02	-2,530	HLA-DMB,HLA-DPB1,HLA-DQB1,HLA-DRB5,IL1B,IL7R,MASP1,NLRP1,PARP8,TLR4
Semaphorin Neuronal Repulsive	2,03E00	5,67E-02	-0,378	BCAN,CRMP1,ITGA10,ITGB3,MYL9,PAK3,PLXND1,SEMA6D
Pyroptosis Signaling Pathway	2,02E00	6,9E-02	-2,449	GSDMD,IL1B,IL1R1,NLRP1,TLR4,TNFRSF1B
Role of Osteoblasts in Rheumato	1,88E00	4,74E-02	-1,667	ACVR2A,CXCL12,DKK1,DLX5,IL1B,SFRP4,STAT6,VEGFC,WNT5A,WNT5B
Calcium-induced T Lymphocyte	1,82E00	7,14E-02	-2,000	HLA-DMB,HLA-DPB1,HLA-DQB1,HLA-DRB5,ITPR3
Acetylcholine Receptor Signaling	1,79E00	4,84E-02	-1,000	ADCY9,CACNG7,CASP10,CHRFAM7A,CHRNA1,GNAO1,HMOX1,ITPR3,PSEN2
Th2 Pathway	1,77E00	5,51E-02	-1,890	ACVR2A,HLA-DMB,HLA-DPB1,HLA-DQB1,HLA-DRB5,PSEN2,STAT6
Ephrin B Signaling	1,77E00	6,94E-02	-1,000	CXCL12,GNAO1,GNG2,RAC2,VAV3
Neutrophil Extracellular Trap Sig	1,77E00	3,94E-02	0,535	CASP10,COL1A2,COL25A1,COL3A1,COL5A1,COL5A2,COL6A2,COL6A3,GSDMD,IL1B,ITPR3,MT-ND3,RAC2,TLR4

Post-translational protein phosph	1,76E00	6,06E-02	-0,816	C4A/C4B,FBN1,FN1,IGFBP4,IGFBP5,STC2
Oxytocin in Brain Signaling Path	1,75E00	4,76E-02	1,000	CACNG7,GNAO1,GNG2,IL1B,ITPR3,NLRP1,OXTR,SLC12A5,TLR4
CXCR4 Signaling	1,68E00	4,91E-02	0,000	ADCY9,CXCL12,GNAO1,GNG2,ITPR3,MYL9,PAK3,RAC2
Role of Tissue Factor in Cancer	1,67E00	4,62E-02	-1,667	ACVR2A,F3,FLNC,IL1B,ITGB3,PLAUR,TFPI,TNFRSF1B,VEGFC
TEC Kinase Signaling	1,67E00	4,62E-02	0,000	GNAO1,GNG2,ITGA10,ITGB3,PAK3,RAC2,STAT6,TLR4,VAV3
ID1 Signaling Pathway	1,65E00	4,57E-02	0,333	ACVR2A,CHRFAM7A,CHRNA1,ETS1,FN1,IGF2,TFAP2A,TNFRSF1B,VEGFC
Apoptotic execution phase	1,64E00	7,69E-02	-1,000	CDH1,DSP,H1-0,PKP1
Immunogenic Cell Death Signali	1,63E00	6,41E-02	-2,236	CGAS,HSPA1A/HSPA1B,IL1B,TLR4,TNFRSF1B
Interleukin-4 and Interleukin-13 s	1,63E00	5,66E-02	-1,633	COL1A2,FN1,HMOX1,IL1B,STAT6,TNFRSF1B
O-linked glycosylation	1,61E00	5,61E-02	-2,449	ADAMTS6,ADAMTS9,ADAMTSL1,GALNT5,GALNT6,MUC20
Actin Cytoskeleton Signaling	1,61E00	4,29E-02	-0,816	ARHGAP24,FN1,ITGA10,ITGB3,MYH3,MYL9,MYO18A,PAK3,RAC2,VAV3
Crosstalk between Dendritic Cell	1,61E00	6,33E-02	-2,236	HLA-DRB5,LTBR,MICB,TLR4,TNFRSF1B
Tumor Microenvironment Pathwa	1,61E00	4,76E-02	-2,646	COL1A2,COL3A1,CXCL12,FN1,IGF2,IL1B,ITGB3,VEGFC

WNT/β-catenin Signaling	1,6E00	4,73E-02	-0,816	ACVR2A,CDH1,DKK1,GNAO1,KREMEN2,SFRP4,WNT5A,WNT5B
LXR/RXR Activation	1,58E00	5,5E-02	2,449	C4A/C4B,IL1B,IL1R1,SERPINF1,TLR4,TNFRSF1B
IL-17A Signaling in Fibroblasts	1,57E00	6,17E-02	-2,236	COL1A2,COL3A1,CXCL12,FN1,IL1B
PKCθ Signaling in T Lymphocyt	1,57E00	4,68E-02	-1,342	CACNG7,HLA-DMB,HLA-DPB1,HLA-DQB1,HLA-DRB5,ITPR3,RAC2,VAV3
IL-8 Signaling	1,55E00	4,39E-02	-1,890	CDH1,GNAO1,GNG2,HMOX1,ITGB3,MYL9,RAC2,TEK,VEGFC
Calcium Signaling	1,55E00	4,39E-02	-0,447	CACNG7,CHRFAM7A,CHRNA1,ITPR3,MYH3,MYL9,MYO18A,RYR2,TNNT1
eNOS Signaling	1,51E00	4,86E-02	0,000	ADCY9,AQP4,CHRFAM7A,CHRNA1,HSPA1A/HSPA1B,ITPR3,VEGFC
ABRA Signaling Pathway	1,49E00	5,88E-02	-1,342	ABLIM3,DMD,MYL9,SMTN,VEGFC
Signaling by ERBB4	1,49E00	6,9E-02	-1,000	CXCL12,ERBB4,GABRQ,PSEN2
IL-15 Production	1,48E00	5,22E-02	-0,816	AXL,ERBB4,NTRK2,ROR1,ROR2,TEK
HOTAIR Regulatory Pathway	1,46E00	4,76E-02	-1,633	CDH1,COL1A2,COL3A1,KMT2A,TLR4,WNT5A,WNT5B
ROBO SLIT Signaling Pathway	1,4E00	0.05	0,816	CDH1,MYL9,NRXN3,PAK3,SLIT3,VEGFC
Cell junction organization	1,4E00	5,56E-02	-2,236	CDH1,CDH13,CDH6,CLDN11,FLNC
Gustation Pathway	1,4E00	4,32E-02	-2,121	ADCY9,CACNG7,GABRE,GABRQ,ITPR3,KCNN4,PDE3A,TAS2R4

Macrophage Classical Activation	1,39E00	4,58E-02	-1,890	HLA-DMB,HLA-DPB1,HLA-DQB1,HLA-DRB5,IL1B,STAT6,TLR4
Cardiac Hypertrophy Signaling (E	1,38E00	3,31E-02	-2,673	ACVR2A,ADCY9,CACNG7,GNG2,IL11RA,IL1B,IL1R1,IL7R,ITGA10,ITGB3,ITPR3,PDE3A,PDE8B,RYR2,TNFRSF1B,WNT5A,WNT5B
FAK Signaling	1,37E00	3,16E-02	-2,683	ACVR2A,CDH1,CNR1,COL1A2,COL3A1,ECM1,ELF4,ETS1,ETS2,GPR135,GPRC5A,IL11RA,IL1R1,IL7R,ITGA10,ITGB3,MC1R,OXTR,PAK3,RGR
Gαs Signaling	1,36E00	4,88E-02	-1,342	ADCY9,CNR1,GNAO1,GNG2,MC1R,RYR2
Signaling by Rho Family GTPas	1,33E00	3,83E-02	-1,342	CDH1,CDH13,CDH6,GNAO1,GNG2,ITGA10,ITGB3,MYL9,PAK3,RAC2
SNARE Signaling Pathway	1,32E00	4,76E-02	-1,633	ADCY9,CPLX2,MYH3,MYL9,MYO18A,SYT14
Role of Chondrocytes in Rheuma	1,3E00	4,72E-02	-2,449	CXCL12,FN1,IL1B,IL1R1,TNFRSF1B,VEGFC

Supplementary Table 9: DEGs in IL6 vs Control conditions (log2FoldChange ≤ -0.59 or ≥ +0.59, adjusted p-value< 0.1)

Gene symbol	Gene name	log2FoldChange	padj
RGPD6	RANBP2 like and GRIP domain containing 6	-3.116487292	0.004069498
DNAJB14	DnaJ heat shock protein family (Hsp40) member B14	-0.67836014	0.004365676
NAIP	NLR family apoptosis inhibitory protein	0.68097623	0.075142651
MFSD6	major facilitator superfamily domain containing 6	1.020866721	0.041961134
CSF1R	colony stimulating factor 1 receptor	6.601059037	0.000704699
ADAMTS1	ADAM metallopeptidase with thrombospondin type 1 motif 1	15.51907473	0.02407535
IKBKGP1	inhibitor of nuclear factor kappa B kinase subunit gamma pseudogene 1	18.47407302	0.000599706
LOC102724843	uncharacterized LOC102724843	18.63293719	0.000517501
DNAAF4-CCPG1	DNAAF4-CCPG1 readthrough (NMD candidate)	23.75320883	0.000000102
ZRSR2P1	ZRSR2 pseudogene 1	24.2673793	5.34E-08
BIVM-ERCC5	BIVM-ERCC5 readthrough	29.68134116	7.23E-13

Supplementary Table 10: DEGs in EPEA +IL6 vs IL6 conditions (log2FoldChange ≤ -0.59 or ≥ +0.59, adjusted p-value< 0.1)

Gene symbol	Gene name	log2FoldChange	padj
UTP14C	UTP14C small subunit processome component	-22.2994572	0.000240699
FLRT1	fibronectin leucine rich transmembrane protein 1	-20.98923537	0.000200988
CHRM4	cholinergic receptor muscarinic 4	-20.46412496	6.4E-09
ADAMTS1	ADAM metallopeptidase with thrombospondin type 1 motif 1	-16.23530954	0.04878835
TMEM183BP	transmembrane protein 183B; pseudogene	-15.83857956	0.067649322
TBCE	tubulin folding cofactor E	-0.722622178	0.013720005
CEMIP	cell migration inducing hyaluronidase 1	1.013795612	0.099898836
KCNB1	potassium voltage-gated channel subfamily B member 1	2.784191759	0.013720005
SLC43A3	solute carrier family 43 member 3	3.189041144	0.018778945

Supplementary Table 11: DEGs in DHEA +IL6 vs IL6 conditions (log2FoldChange ≤ -0.59 or ≥ +0.59, adjusted p-value< 0.1)

Gene symbol	Gene name	log2FoldChange	padj
PMS2P14	PMS1 homolog 2; mismatch repair system component pseudogene 14	-6.245473403	0.025665323
SMG1P4	SMG1 pseudogene 4	-5.679596023	0.0000826
SYCP2	synaptonemal complex protein 2	-2.564118581	0.095745341
SAP30L-AS1	SAP30L antisense RNA 1 (head to head)	-2.534297954	0.065064433
LINC00887	long intergenic non-protein coding RNA 887	-2.16602297	0.064529384
NPHP3-ACAD11	NPHP3-ACAD11 readthrough (NMD candidate)	-2.084241224	0.090038384
ERC2	ELKS/RAB6-interacting/CAST family member 2	-1.34758674	0.031791664
STIMATE-MUSTN1	STIMATE-MUSTN1 readthrough	-1.224838675	0.06512372
RGPD8	RANBP2 like and GRIP domain containing 8	-1.151762242	0.081983021
FAM156B	family with sequence similarity 156 member B	-0.928058576	0.025665323
CHDH	choline dehydrogenase	-0.914076659	0.06512372
ATP6V1G2-DDX39B	ATP6V1G2-DDX39B readthrough (NMD candidate)	-0.903157333	0.074916193
PPAN-P2RY11	PPAN-P2RY11 readthrough	-0.833626961	0.051032656
DDX60L	DExD/H-box 60 like	-0.826566747	0.049324733
SMG1P1	SMG1 pseudogene 1	-0.777096987	0.031589201
NPIPB4	nuclear pore complex interacting protein family member B4	-0.77456588	0.031589201
GRAMD1A	GRAM domain containing 1A	-0.720360607	0.031589201
GTF2IRD2	GTF2I repeat domain containing 2	-0.709017363	0.090038384
MFAP3L	microfibril associated protein 3 like	-0.676035233	0.081983021
CASP1	caspase 1	-0.675718915	0.090038384
KRT80	keratin 80	-0.627827491	0.099003388
SFI1	SFI1 centrin binding protein	-0.595712441	0.0015178
ANOS1	anosmin 1	0.590423671	0.067340601
NFATC4	nuclear factor of activated T cells 4	0.59699886	0.031589201
MGLL	monoglyceride lipase	0.614345697	0.074916193
GPX3	glutathione peroxidase 3	0.614709643	0.093366755
S1PR1	sphingosine-1-phosphate receptor 1	0.620103757	0.080595701
FAM107A	family with sequence similarity 107 member A	0.624868415	0.011597606

FAM222A	family with sequence similarity 222 member A	0.656047353	0.081983021
DLL3	delta like canonical Notch ligand 3	0.709065119	0.064529384
DNAH9	dynein axonemal heavy chain 9	0.738850737	0.06911808
PDLIM3	PDZ and LIM domain 3	0.774744619	0.090038384
ACSBG1	acyl-CoA synthetase bubblegum family member 1	0.859721669	0.044246031
PPP2R2B	protein phosphatase 2 regulatory subunit Bbeta	0.862228891	0.011597606
ISLR	immunoglobulin superfamily containing leucine rich repeat	0.913939178	0.064529384
RGS8	regulator of G protein signaling 8	0.920384106	0.027637195
INSYN1	inhibitory synaptic factor 1	0.931331096	0.074916193
ATP1A2	ATPase Na+/K+ transporting subunit alpha 2	0.973201145	0.00000834
DLGAP1	DLG associated protein 1	0.992087497	0.035171294
SATB1	SATB homeobox 1	1.061650762	0.090038384
FAM181B	family with sequence similarity 181 member B	1.062269836	0.045563446
PPARA	peroxisome proliferator activated receptor alpha	1.062571067	0.031589201
DISP3	dispatched RND transporter family member 3	1.122488063	0.07053823
NRXN3	neurexin 3	1.177981384	0.090038384
PDK4	pyruvate dehydrogenase kinase 4	1.220809483	0.004026237
NYNRIN	NYN domain and retroviral integrase containing	1.296565273	0.031589201
TTC23L	tetratricopeptide repeat domain 23 like	1.407764678	0.063030378
PHETA1	PH domain containing endocytic trafficking adaptor 1	1.440973964	0.077113411
DIO3	iodothyronine deiodinase 3	1.542706717	0.094645104
PDZD2	PDZ domain containing 2	1.749996737	0.004709653
FOXP2	forkhead box P2	1.940039127	0.090038384
HMGCS2	3-hydroxy-3-methylglutaryl-CoA synthase 2	3.920411178	0.090038384

Supplementary Table 12: DEGs in Acetate+IL1β vs IL1β conditions (log2FoldChange ≤ -0.59 or ≥ +0.59, adjusted p-value< 0.1)

Gene symbol	Gene name	log2FoldChange	padj
SPECC1L-ADORA2A	SPECC1L-ADORA2A readthrough (NMD candidate)	-29.92854917	2.81E-14
ASDURF	ASNSD1 upstream open reading frame	-19.75891686	0.0000245
ZCWPW1	zinc finger CW-type and PWWP domain containing 1	-2.634364821	0.052035499
PCDH19	protocadherin 19	-1.081637854	0.011730538
H2BC19P	H2B clustered histone 19, pseudogene	1.23412786	0.019273568
ANKRD18A	ankyrin repeat domain 18A	1.355193547	0.0000763
FRY	FRY microtubule binding protein	1.44025236	0.0070252
ADAMTS4	ADAM metallopeptidase with thrombospondin type 1 motif 4	1.625745223	0.052035499
ZNF761	zinc finger protein 761	3.505894115	0.040269432
EFCC1	EF-hand and coiled-coil domain containing 1	5.157004227	0.011730538

Supplementary Table 13: DEGs in Propionate +IL1β vs IL1β conditions (log2FoldChange ≤ -0.59 or ≥ +0.59, adjusted p-value< 0.1)

Gene symbol	Gene name	log2FoldChange	padj
NOMO3	NODAL modulator 3	0.969898112	0.021704171
FRY	FRY microtubule binding protein	1.364194859	0.039131626
TPTEP2-CSNK1E	TPTEP2-CSNK1E readthrough	-4.275917466	0.083792808

Supplementary Table 14: DEGs in Butyrate +IL1β vs IL1β conditions (log2FoldChange ≤ -0.59 or ≥ +0.59, adjusted p-value< 0.1)

Gene symbol	Gene name	log2FoldChange	padj
SNHG18	small nucleolar RNA host gene 18	-1.968739261	0.0571391
RORA	RAR related orphan receptor A	-1.523775388	0.0571391
PCDH19	protocadherin 19	-0.896179142	0.0067709
TERF1	telomeric repeat binding factor 1	1.279351981	0.0085542

Supplementary Table 15: DEGs in Acetate vs Control conditions (log2FoldChange ≤ -0.59 or ≥ +0.59, adjusted p-value< 0.1)

Gene symbol	Gene name	log2FoldChange	padj
IFIT2	interferon induced protein with tetratricopeptide repeats 2	21.53656946	0.0000195
SPECC1L-ADORA2A	SPECC1L-ADORA2A readthrough (NMD candidate)	20.5673532	8.69E-11
P2RY6	pyrimidinergic receptor P2Y6	5.887850359	0.01614904
ZIC3	Zic family member 3	4.196683274	0.02748943
MGAT4C	MGAT4 family member C	3.358121679	0.00022108
FBN3	fibrillin 3	3.290393898	0.01718593
GJB2	gap junction protein beta 2	2.968514348	0.00116254
CPLX2	complexin 2	2.968443055	0.00054796
MYT1	myelin transcription factor 1	2.854367866	0.00901194
ATP13A4	ATPase 13A4	2.595923268	0.02777095
ACTA1	actin alpha 1; skeletal muscle	2.361939703	0.08626892
NPL	N-acetylneuraminate pyruvate lyase	2.319026913	0.0000117
SLC12A5	solute carrier family 12 member 5	2.078370558	0.07781258
SERPINA3	serpin family A member 3	1.990515068	0.01963396
MASP1	MBL associated serine protease 1	1.978290022	0.03726958
ADGRA1	adhesion G protein-coupled receptor A1	1.933472379	0.06114151
CILK1	ciliogenesis associated kinase 1	1.909135378	0.01158838
ADCY7	adenylate cyclase 7	1.880932131	0.08069377
NKILA	NF-kappaB interacting lncRNA	1.877115034	0.09013342
KLHL4	kelch like family member 4	1.845627442	0.04939393
VAV3	vav guanine nucleotide exchange factor 3	1.782733042	0.0019481
NTRK2	neurotrophic receptor tyrosine kinase 2	1.76197758	0.0000161
RGS8	regulator of G protein signaling 8	1.710355664	0.00000977
DENND1C	DENN domain containing 1C	1.652608571	0.00827144
PKP1	plakophilin 1	1.595597193	0.0000005
ARID3A	AT-rich interaction domain 3A	1.583229428	0.00177686
DNM3	dynamin 3	1.577355425	0.00084314
RGR	retinal G protein coupled receptor	1.529039612	0.025168
NRXN3	neurexin 3	1.47930703	0.04581267
SORCS2	sortilin related VPS10 domain containing receptor 2	1.404888458	2.07E-07
SPRN	shadow of prion protein	1.350506854	0.00084314

DIPK1C	divergent protein kinase domain 1C	1.344740399	0.00083161
TMEM45A	transmembrane protein 45A	1.32626713	0.0671312
NDRG2	NDRG family member 2	1.275445166	0.0014726
PMEPA1	prostate transmembrane protein; androgen induced 1	1.265804482	1.53E-10
CHRNA1	cholinergic receptor nicotinic alpha 1 subunit	1.235013792	0.06991997
ZFR2	zinc finger RNA binding protein 2	1.232394602	0.09013342
PCDH1	protocadherin 1	1.211274182	0.0000374
COL14A1	collagen type XIV alpha 1 chain	1.201864762	0.0000114
CRMP1	collapsin response mediator protein 1	1.176222225	2.76E-10
ATCAY	ATCAY kinesin light chain interacting caytaxin	1.174914997	0.03514418
NFE2L3	NFE2 like bZIP transcription factor 3	1.166056116	0.0000585
HFM1	helicase for meiosis 1	1.152753857	0.04063876
C4B	complement C4B (Chido blood group)	1.151387341	0.00413597
SERPINF1	serpin family F member 1	1.135660782	0.0000482
NALF2	NALCN channel auxiliary factor 2	1.13390766	0.06513245
TOX3	TOX high mobility group box family member 3	1.122790696	0.0000578
DLGAP1	DLG associated protein 1	1.116823609	0.00143547
YPEL2	yippee like 2	1.104348309	0.08372789
ANGPTL1	angiopoietin like 1	1.070853199	6.56E-07
HOXB3	homeobox B3	1.069941366	0.0002451
HBA1	hemoglobin subunit alpha 1	1.043027535	0.0000353
SPOCK1	SPARC (osteonectin); cwcv and kazal like domains proteoglycan 1	1.016281256	0.00894676
RASL11B	RAS like family 11 member B	0.999481816	0.03692849
RGS16	regulator of G protein signaling 16	0.996006186	0.0000145
CACNG7	calcium voltage-gated channel auxiliary subunit gamma 7	0.977219343	0.00000058
SLC26A7	solute carrier family 26 member 7	0.967038217	0.09000172
ROR1	receptor tyrosine kinase like orphan receptor 1	0.965697417	0.05726825
ENTREP2	endosomal transmembrane epsin interactor 2	0.951898848	0.03932234
IGFBP5	insulin like growth factor binding protein 5	0.95139694	7.28E-10

FILIP1L	filamin A interacting protein 1 like	0.946157664	0.0076844
EFHD1	EF-hand domain family member D1	0.937797316	0.00723802
HDC	histidine decarboxylase	0.928578191	0.08805201
LRRC3	leucine rich repeat containing 3	0.923589642	0.03421785
SSPN	sarcospan	0.920815023	0.06027869
KREMEN2	kringle containing transmembrane protein 2	0.91084716	0.0113907
SCD5	stearoyl-CoA desaturase 5	0.906623847	7.06E-10
DLX5	distal-less homeobox 5	0.896927207	0.04262772
SULF2	sulfatase 2	0.887617117	5.16E-07
GIN1	gypsy retrotransposon integrase 1	0.877059184	0.09235063
IDE	insulin degrading enzyme	0.875660663	0.01191995
C1orf50	chromosome 1 open reading frame 50	0.840666253	0.00110778
CPLX1	complexin 1	0.823614837	0.06277331
TNFRSF19	TNF receptor superfamily member 19	0.814924066	0.0000791
ANK3	ankyrin 3	0.796853738	0.00808171
LEFTY2	left-right determination factor 2	0.780239153	0.06136354
PLP1	proteolipid protein 1	0.779211375	0.02002264
LCORL	ligand dependent nuclear receptor corepressor like	0.773771281	0.00622287
YBX2	Y-box binding protein 2	0.769742798	0.07832046
TRIM2	tripartite motif containing 2	0.758485168	0.02107128
EPHB3	EPH receptor B3	0.756050164	2.33E-09
RNF180	ring finger protein 180	0.752823943	0.0056636
MFAP4	microfibril associated protein 4	0.752305712	0.01614904
INSM1	INSM transcriptional repressor 1	0.74612794	0.02507761
RCOR2	REST corepressor 2	0.742618116	0.00177686
ZNF175	zinc finger protein 175	0.736325986	0.01246043
ZNF641	zinc finger protein 641	0.735452512	0.03282077
PTPN4	protein tyrosine phosphatase non-receptor type 4	0.734054149	0.02868726
KCNN4	potassium calcium-activated channel subfamily N member 4	0.719588245	0.02519254
ACTG2	actin gamma 2; smooth muscle	0.718540754	0.04857671
PCDH19	protocadherin 19	0.716659978	0.00853634
TMEM67	transmembrane protein 67	0.712728815	0.01215381
SHROOM2	shroom family member 2	0.710009807	0.00140492
TSPAN15	tetraspanin 15	0.704742754	0.02504204
ELAVL3	ELAV like RNA binding protein 3	0.704445069	0.09235063

LRRTM2	leucine rich repeat transmembrane neuronal 2	0.685269816	0.06136354
PRDM1	PR/SET domain 1	0.682539609	0.09594457
VWA1	von Willebrand factor A domain containing 1	0.68149849	0.01635612
VCAM1	vascular cell adhesion molecule 1	0.677810719	0.04376008
PSEN2	presenilin 2	0.6737772	0.03053702
MAST1	microtubule associated serine/threonine kinase 1	0.669590701	0.0321347
GNG2	G protein subunit gamma 2	0.666911881	0.00052687
ESPN	espin	0.663013186	0.02084655
ND3	NADH dehydrogenase subunit 3	0.658820229	1.15E-07
GNG4	G protein subunit gamma 4	0.658320203	9.99E-11
PAK3	p21 (RAC1) activated kinase 3	0.655784736	0.00364201
NOG	noggin	0.650635598	0.03638431
SRD5A3	steroid 5 alpha-reductase 3	0.64734214	0.04959465
ZBTB7B	zinc finger and BTB domain containing 7B	0.647191824	0.00058202
MTND2P28	MT-ND2 pseudogene 28	0.646850559	0.0020682
GRHL3	grainyhead like transcription factor 3	0.646827591	0.00908952
HS3ST3A1	heparan sulfate-glucosamine 3-sulfotransferase 3A1	0.637784966	0.04198316
ZNF414	zinc finger protein 414	0.636325977	0.00024448
AUTS2	activator of transcription and developmental regulator AUTS2	0.635435263	0.04347805
CD24	CD24 molecule	0.632187814	0.00439191
BMP2	bone morphogenetic protein 2	0.632152233	0.00083544
JAKMIP2-AS1	JAKMIP2 antisense RNA 1	0.629130493	0.00610983
DEPTOR	DEP domain containing MTOR interacting protein	0.628307176	0.02160898
ARHGEF3	Rho guanine nucleotide exchange factor 3	0.607583072	0.03720798
ZNF814	zinc finger protein 814	0.59837412	0.0113907
POLR2J3	RNA polymerase II subunit J3	0.595554551	0.03208864
DST	dystonin	-0.590772764	0.0000101
SFXN2	sideroflexin 2	-0.592615455	0.02125914
ZBTB38	zinc finger and BTB domain containing 38	-0.594000667	0.01025122
MYO18A	myosin XVIIIA	-0.594974074	1.82E-09
DMXL1	Dmx like 1	-0.595317203	0.00165415
SDC4	syndecan 4	-0.596143004	0.00027478
SDK1	sidekick cell adhesion molecule 1	-0.596206754	0.00195295

PURPL	p53 upregulated regulator of p53 levels	-0.598324896	0.04558155
MDN1	midasin AAA ATPase 1	-0.600695812	0.00385567
FRMD6	FERM domain containing 6	-0.600887794	0.0838361
HOPX	HOP homeobox	-0.601166447	0.03340367
SYNCRIP	synaptotagmin binding cytoplasmic RNA interacting protein	-0.604893915	0.00595029
NQO1	NAD(P)H quinone dehydrogenase 1	-0.607669351	0.00395449
NBPF9	NBPF member 9	-0.614372954	0.00560527
PREX1	phosphatidylinositol-3;4;5-trisphosphate dependent Rac exchange factor 1	-0.614543129	0.00336526
FTH1	ferritin heavy chain 1	-0.617961042	0.0000957
HERC2	HECT and RLD domain containing E3 ubiquitin protein ligase 2	-0.621608819	0.00979956
TLE3	TLE family member 3; transcriptional corepressor	-0.622993857	0.00385567
PARVB	parvin beta	-0.625371695	0.08663997
AFG3L1P	AFG3 like matrix AAA peptidase subunit 1; pseudogene	-0.626894128	0.00384297
PPP1R1B	protein phosphatase 1 regulatory inhibitor subunit 1B	-0.627161776	0.00051886
HEG1	heart development protein with EGF like domains 1	-0.633678363	0.00108277
ITPR3	inositol 1;4;5-trisphosphate receptor type 3	-0.633682095	0.01529598
NRP1	neuropilin 1	-0.636456821	0.00041272
EEF1A1	eukaryotic translation elongation factor 1 alpha 1	-0.636488488	0.00000762
MDGA1	MAM domain containing glycosylphosphatidylinositol anchor 1	-0.639875244	0.00030338
LOXL2	lysyl oxidase like 2	-0.640912391	0.00112093
SHC3	SHC adaptor protein 3	-0.64384832	0.00000105
CSPG4	chondroitin sulfate proteoglycan 4	-0.645651317	0.0081395
PLEKHG4B	pleckstrin homology and RhoGEF domain containing G4B	-0.647064746	6.05E-08
PCNT	pericentrin	-0.647378007	0.03601117
NOTCH2NLA	notch 2 N-terminal like A	-0.648539909	0.00714428
PGM5	phosphoglucomutase 5	-0.649438479	0.06303099
DGKH	diacylglycerol kinase eta	-0.650206217	0.08769536

PI15	peptidase inhibitor 15	-0.651872506	3.15E-10
HSPA1A	heat shock protein family A (Hsp70) member 1A	-0.652969726	0.00699741
GABRQ	gamma-aminobutyric acid type A receptor subunit theta	-0.654110629	0.0000469
SEMA5A	semaphorin 5A	-0.654468179	0.00012827
OPLAH	5-oxoprolinase; ATP-hydrolysing	-0.660413969	0.03720798
MYBL1	MYB proto-oncogene like 1	-0.661649571	0.00404973
PHF11	PHD finger protein 11	-0.665358979	0.03422178
NAV2	neuron navigator 2	-0.6660002	0.00177141
KMT2A	lysine methyltransferase 2A	-0.668859728	0.01755798
CYP7B1	cytochrome P450 family 7 subfamily B member 1	-0.66949317	0.05443472
DNMT1	DNA methyltransferase 1	-0.669874094	0.00350927
ETV4	ETS variant transcription factor 4	-0.670880201	0.00164773
MACF1	microtubule actin crosslinking factor 1	-0.671511692	0.00000189
FBN1	fibrillin 1	-0.676011134	0.09152139
STK17B	serine/threonine kinase 17b	-0.684063876	0.03818164
RFX4	regulatory factor X4	-0.685680483	0.00084314
MSH5	mutS homolog 5	-0.686371644	0.00498346
FBN2	fibrillin 2	-0.686879806	0.0000152
NBPF14	NBPF member 14	-0.687356783	0.00000339
ITGB3	integrin subunit beta 3	-0.688121781	0.01295802
ANGPTL4	angiopoietin like 4	-0.690062616	0.03307611
B3GALT1	beta-1;3-galactosyltransferase 1	-0.694078268	0.0007643
HLA-DMB	major histocompatibility complex; class II; DM beta	-0.698362903	0.08453359
ALMS1	ALMS1 centrosome and basal body associated protein	-0.69970133	0.03178763
NCKAP5	NCK associated protein 5	-0.702904419	0.04493203
LOC100288637	OTU deubiquitinase 7A pseudogene	-0.705957948	0.06535091
ADAMTS9	ADAM metallopeptidase with thrombospondin type 1 motif 9	-0.706344703	0.01142929
CYP1B1	cytochrome P450 family 1 subfamily B member 1	-0.707306811	0.07482084
CASQ1	calsequestrin 1	-0.709809947	0.00760092
F3	coagulation factor III; tissue factor	-0.711022664	2.81E-08
ECHDC3	enoyl-CoA hydratase domain containing 3	-0.71218386	0.01463083
GNAO1	G protein subunit alpha o1	-0.719462833	0.01176159
CLTCL1	clathrin heavy chain like 1	-0.720720531	0.01529598

B3GALT5	beta-1;3-galactosyltransferase 5	-0.7220856	0.00972148
NBEA	neurobeachin	-0.723481805	0.0034653
SMG1P3	SMG1 pseudogene 3	-0.725237799	0.00439373
FKBP11	FKBP prolyl isomerase 11	-0.726333128	0.03083868
FIGN	fidgetin; microtubule severing factor	-0.728455934	0.05808404
UTRN	utrophin	-0.729573161	0.00000493
A2M	alpha-2-macroglobulin	-0.730453413	0.0027729
45508	argonaute RISC component 4	-0.733448113	0.05737956
CUL9	cullin 9	-0.7348549	0.07339222
ALPL	alkaline phosphatase; biomineralization associated	-0.738719923	0.04049795
SAP30L	SAP30 like	-0.739049553	0.04760112
CADM2	cell adhesion molecule 2	-0.739579401	0.00428356
CDH1	cadherin 1	-0.742093724	0.000025
COL5A1	collagen type V alpha 1 chain	-0.744242833	0.00898349
CARD16	caspase recruitment domain family member 16	-0.746288968	0.05122919
TTLL3	tubulin tyrosine ligase like 3	-0.751058885	0.00125497
SLC1A2	solute carrier family 1 member 2	-0.75136651	1.82E-09
PLAUR	plasminogen activator; urokinase receptor	-0.753751819	0.00036138
GPR135	G protein-coupled receptor 135	-0.758971991	0.0599564
FOXJ1	forkhead box J1	-0.77011619	0.0000109
ATP1A2	ATPase Na+/K+ transporting subunit alpha 2	-0.772691098	0.06371269
ITGA5	integrin subunit alpha 5	-0.777825367	4.59E-09
OBSCN	obscurin; cytoskeletal calmodulin and titin-interacting RhoGEF	-0.778384984	0.09583143
ETS2	ETS proto-oncogene 2; transcription factor	-0.779034011	0.03449353
AMBN	ameloblastin	-0.780435243	0.07494061
CDH6	cadherin 6	-0.783589761	4.37E-07
ERBB4	erb-b2 receptor tyrosine kinase 4	-0.784291829	0.00077465
RALYL	RALY RNA binding protein like	-0.786245065	0.06535091
NBPF19	NBPF member 19	-0.786332474	0.00385399
TEK	TEK receptor tyrosine kinase	-0.791379284	0.00112189
UNC80	unc-80 homolog; NALCN channel complex subunit	-0.794455763	0.07497111
NEK11	NIMA related kinase 11	-0.795217276	0.06465539
TMEM91	transmembrane protein 91	-0.798891371	0.09351567
FAM156A	family with sequence similarity 156 member A	-0.81425324	0.08058883

RBPMS2	RNA binding protein; mRNA processing factor 2	-0.817848934	0.01728172
MFAP5	microfibril associated protein 5	-0.824776104	0.0008291
VPS13D	vacuolar protein sorting 13 homolog D	-0.830065575	3.59E-07
ASH1L	ASH1 like histone lysine methyltransferase	-0.830230482	0.03737727
CNR1	cannabinoid receptor 1	-0.831070279	0.03602223
NPIPB5	nuclear pore complex interacting protein family member B5	-0.833236664	0.00061164
ANKRD36	ankyrin repeat domain 36	-0.8389734	0.01453481
FAM229A	family with sequence similarity 229 member A	-0.839237883	0.03057082
ENPP2	ectonucleotide pyrophosphatase/phosphodiesterase 2	-0.841937866	0.00178744
ATOH8	atonal bHLH transcription factor 8	-0.849187594	0.00124591
IL11RA	interleukin 11 receptor subunit alpha	-0.851983195	0.06952769
ZNF483	zinc finger protein 483	-0.85555748	0.0000305
AQP4	aquaporin 4	-0.857734131	7.82E-13
KANTR	KANTR integral membrane protein	-0.86333906	0.00066601
SDK2	sidekick cell adhesion molecule 2	-0.865384867	0.04276629
SH3BP1	SH3 domain binding protein 1	-0.867080928	0.01818839
TGFBI	transforming growth factor beta induced	-0.870995404	0.00717461
SORL1	sortilin related receptor 1	-0.883204798	5.74E-09
TBC1D2	TBC1 domain family member 2	-0.883954869	0.00294476
HKDC1	hexokinase domain containing 1	-0.8902618	0.0000721
XIST	X inactive specific transcript	-0.891150745	0.02772468
FAM181A	family with sequence similarity 181 member A	-0.891793602	0.09191504
PDE8B	phosphodiesterase 8B	-0.897033669	0.00898349
CFAP126	cilia and flagella associated protein 126	-0.900869733	0.03913007
FMN1	formin 1	-0.902744669	0.03282077
SEPT5-GP1BB	SEPT5-GP1BB readthrough	-0.904711066	0.04493203
DLC1	DLC1 Rho GTPase activating protein	-0.905137034	0.06535091
B3GAT2	beta-1;3-glucuronyltransferase 2	-0.907674742	0.01336082
SEMA6D	semaphorin 6D	-0.915167459	1.82E-09

LINC00174	long intergenic non-protein coding RNA 174	-0.917945916	0.02412916
SFRP4	secreted frizzled related protein 4	-0.920631721	0.0000241
KMT2D	lysine methyltransferase 2D	-0.92231263	0.0000243
TNXB	tenascin XB	-0.923346595	0.07089868
DOK6	docking protein 6	-0.925361386	0.00000787
IER3	immediate early response 3	-0.927987873	7.36E-10
EPAS1	endothelial PAS domain protein 1	-0.93154345	0.05533372
MIRLET7IHG	MIRLET7I host gene	-0.935932883	0.01576599
B3GNT9	UDP-GlcNAc:betaGal beta-1;3-N-acetylglucosaminyltransferase 9	-0.937896318	0.01264331
MICAL2	microtubule associated monooxygenase; calponin and LIM domain containing 2	-0.950530364	0.0000512
TLR4	toll like receptor 4	-0.950904072	0.00415975
XYLT1	xylosyltransferase 1	-0.96155136	1.34E-07
DRC1	dynein regulatory complex subunit 1	-0.965921965	0.0000262
FAT3	FAT atypical cadherin 3	-0.966655979	0.00244048
C6orf118	chromosome 6 open reading frame 118	-0.968749238	0.00051886
PHLDB2	pleckstrin homology like domain family B member 2	-0.973994274	0.0893268
MIR924HG	MIR924 host gene	-0.978323324	0.09380891
SMTN	smoothelin	-0.982719049	0.0000117
BDNF	brain derived neurotrophic factor	-0.983841721	0.04493061
TC2N	tandem C2 domains; nuclear	-0.994719274	0.00054703
TRERF1	transcriptional regulating factor 1	-0.995391827	0.00364201
SYT14	synaptotagmin 14	-1.004350318	0.06375581
LY6H	lymphocyte antigen 6 family member H	-1.012146462	0.00163521
NWD1	NACHT and WD repeat domain containing 1	-1.01511181	0.04493061
TRPV3	transient receptor potential cation channel subfamily V member 3	-1.015679948	0.00045482
CD101	CD101 molecule	-1.019059768	0.02330651
RN7SL1	RNA component of signal recognition particle 7SL1	-1.024992331	0.03167251
KIAA1614	KIAA1614	-1.030751718	0.00234913

SYNE2	spectrin repeat containing nuclear envelope protein 2	-1.039633807	6.72E-16
TSHZ3	teashirt zinc finger homeobox 3	-1.045463695	0.0640319
CFAP92	cilia and flagella associated protein 92 (putative)	-1.050150541	0.03515403
FHIP1A	FHF complex subunit HOOK interacting protein 1A	-1.050403261	0.00729092
INSYN2B	inhibitory synaptic factor family member 2B	-1.067006284	0.01982646
CCDC80	coiled-coil domain containing 80	-1.067755629	0.00060235
COPB2-DT	COPB2 divergent transcript	-1.068652749	0.04493061
ELMOD1	ELMO domain containing 1	-1.078779241	3.41E-13
RYR2	ryanodine receptor 2	-1.086358219	8.23E-09
MC1R	melanocortin 1 receptor	-1.095825449	0.0103698
ARHGEF28	Rho guanine nucleotide exchange factor 28	-1.118333665	0.0742532
CXCL12	C-X-C motif chemokine ligand 12	-1.118583894	0.00010242
DNAH6	dynein axonemal heavy chain 6	-1.141221865	0.00045482
TNFAIP8	TNF alpha induced protein 8	-1.15149916	0.09113907
SIAH3	siah E3 ubiquitin protein ligase family member 3	-1.152069826	0.00000146
AHNAK	AHNAK nucleoprotein	-1.164404571	3.14E-15
DNAH1	dynein axonemal heavy chain 1	-1.167955969	0.03557257
WNT5B	Wnt family member 5B	-1.168137481	1.45E-08
TMEM132E	transmembrane protein 132E	-1.176697848	1.82E-07
ANKRD36C	ankyrin repeat domain 36C	-1.177374924	0.07953808
ANKRD63	ankyrin repeat domain 63	-1.190451473	0.00062686
RGS4	regulator of G protein signaling 4	-1.199237268	0.04954755
TCIRG1	T cell immune regulator 1; ATPase H+ transporting V0 subunit a3	-1.202195308	0.07313376
G0S2	G0/G1 switch 2	-1.208785705	0.00452326
IGFBP4	insulin like growth factor binding protein 4	-1.216091503	1.36E-07
EMILIN1	elastin microfibril interfacer 1	-1.216297335	0.0009853
MGST1	microsomal glutathione S-transferase 1	-1.221897312	0.00341174
VSTM4	V-set and transmembrane domain containing 4	-1.223244377	0.03913007
NPIPB4	nuclear pore complex interacting protein family member B4	-1.23326352	0.00035354

ADAMTSL1	ADAMTS like 1	-1.233464556	0.00546432
SERPINE1	serpin family E member 1	-1.245501459	6.46E-17
ST3GAL1	ST3 beta-galactoside alpha-2;3-sialyltransferase 1	-1.246393134	0.02160898
MMRN1	multimerin 1	-1.248176956	1.29E-14
FUT9	fucosyltransferase 9	-1.25114686	1.27E-09
HMGA2	high mobility group AT-hook 2	-1.27921681	7.05E-07
CHRFAM7A	CHRNA7 (exons 5-10) and FAM7A (exons A-E) fusion	-1.282956966	0.00904779
STC2	stanniocalcin 2	-1.297866649	7.98E-12
IL1R1	interleukin 1 receptor type 1	-1.305539879	0.03737727
PTGS1	prostaglandin-endoperoxide synthase 1	-1.308743419	0.00862672
LOC100129534	small nuclear ribonucleoprotein polypeptide N pseudogene	-1.324579629	0.06034561
LINC00488	long intergenic non-protein coding RNA 488	-1.3393711	0.04237805
TNFRSF1B	TNF receptor superfamily member 1B	-1.349355582	0.03076337
EPM2A-DT	EPM2A divergent transcript	-1.361337645	0.06930617
LINC01123	long intergenic non-protein coding RNA 1123	-1.365319756	0.07111331
SCEL	sciellin	-1.378241589	0.05538103
PNPLA7	patatin like phospholipase domain containing 7	-1.386917731	0.00227312
NBPF20	NBPF member 20	-1.39766468	1.2E-11
EMP1	epithelial membrane protein 1	-1.406260474	3.15E-10
AMN	amnion associated transmembrane protein	-1.407598463	0.06166153
PCOLCE	procollagen C-endopeptidase enhancer	-1.419481553	0.00000386
COL5A2	collagen type V alpha 2 chain	-1.450545795	0.0000393
KRT18	keratin 18	-1.475889603	2.46E-09
EHBP1L1	EH domain binding protein 1 like 1	-1.511692865	7.95E-11
AHRR	aryl hydrocarbon receptor repressor	-1.517605343	0.01372577
SCAND3	SCAN domain containing 3	-1.528878964	0.00173113
MICB	MHC class I polypeptide-related sequence B	-1.541787293	0.04493203
RPL23AP21	ribosomal protein L23a pseudogene 21	-1.543125439	0.09370744
KCNQ1OT1	KCNQ1 opposite strand/antisense transcript 1	-1.544706914	0.00290798
SLFN5	schlafen family member 5	-1.544884263	0.0106299
TRABD2A	TraB domain containing 2A	-1.549569721	0.0613675

GRIN2D	glutamate ionotropic receptor NMDA type subunit 2D	-1.578314755	0.09950516
MYRF	myelin regulatory factor	-1.584744498	0.01599138
CAVIN2	caveolae associated protein 2	-1.584830613	0.0202952
HECW2	HECT; C2 and WW domain containing E3 ubiquitin protein ligase 2	-1.592333348	0.00230444
GALNT5	polypeptide N-acetylgalactosaminyltransferase 5	-1.593731793	3.01E-19
ESRG	embryonic stem cell related	-1.597939793	0.00070055
GDF10	growth differentiation factor 10	-1.605296108	0.00230993
FLG	filaggrin	-1.612074559	5.96E-16
CHODL	chondrolectin	-1.623722324	0.03131473
SKAP2	src kinase associated phosphoprotein 2	-1.636059254	0.07359776
CLDN11	claudin 11	-1.645996431	0.0021636
JCAD	junctional cadherin 5 associated	-1.649076058	0.00012827
MT2A	metallothionein 2A	-1.682780988	1.67E-22
STXBP6	syntaxin binding protein 6	-1.694278014	0.07111331
TFPI	tissue factor pathway inhibitor	-1.69678949	8.79E-07
PAX8-AS1	PAX8 antisense RNA 1	-1.706929311	0.04376153
MOK	MOK protein kinase	-1.72626367	1.17E-11
BICC1	BicC family RNA binding protein 1	-1.726749335	0.00015342
CAPG	capping actin protein; gelsolin like	-1.739419111	0.07186516
H1-0	H1,0 linker histone	-1.742048023	6.72E-16
RCN3	reticulocalbin 3	-1.753819497	0.00112093
MDGA2	MAM domain containing glycosylphosphatidylinositol anchor 2	-1.779077161	0.07313376
C2orf50	chromosome 2 open reading frame 50	-1.787341424	0.07359776
IFNE	interferon epsilon	-1.794821028	0.03680103
OTULINL	OTU deubiquitinase with linear linkage specificity like	-1.804125878	0.00810863
DSP	desmoplakin	-1.858776945	4.84E-10
SLIT3	slit guidance ligand 3	-1.871883637	7.27E-13
FN1	fibronectin 1	-1.873543357	5.98E-42
CCR1	C-C motif chemokine receptor 1	-1.878385594	0.0818272
FAT4	FAT atypical cadherin 4	-1.879234481	0.02361696
QPRT	quinolinate phosphoribosyltransferase	-1.882390258	0.00300366
FLNC	filamin C	-1.886257179	1.82E-31

CHST8	carbohydrate sulfotransferase 8	-1.897515737	0.09113907
SEMA7A	semaphorin 7A (John Milton Hagen blood group)	-1.913591187	0.0000122
ALDH3A1	aldehyde dehydrogenase 3 family member A1	-1.924232283	0.0000158
MRGPRF	MAS related GPR family member F	-1.927797138	0.000015
MYL9	myosin light chain 9	-1.930364374	2.91E-10
IL7R	interleukin 7 receptor	-1.936142127	0.00128265
GABRE	gamma-aminobutyric acid type A receptor subunit epsilon	-1.974861074	0.01728172
DNER	delta/notch like EGF repeat containing	-1.999067756	0.00000986
OXTR	oxytocin receptor	-2.028620627	0.0000227
SOD3	superoxide dismutase 3	-2.036535396	0.06034561
TAS2R4	taste 2 receptor member 4	-2.073195743	0.05394064
ADAMTS6	ADAM metallopeptidase with thrombospondin type 1 motif 6	-2.076343116	0.00177686
ITGA10	integrin subunit alpha 10	-2.102106326	0.03956611
CSTF3-DT	CSTF3 divergent transcript	-2.121426409	0.04276629
CD68	CD68 molecule	-2.129483639	0.01728172
HMOX1	heme oxygenase 1	-2.135593706	0.00065281
TMC6	transmembrane channel like 6	-2.155463996	0.02180923
ETS1	ETS proto-oncogene 1; transcription factor	-2.16172155	2.34E-07
GATA2	GATA binding protein 2	-2.169962978	0.0000288
VWF	von Willebrand factor	-2.186470952	0.03262779
PAPPA	pappalysin 1	-2.187173053	0.00014358
TMEM154	transmembrane protein 154	-2.19697456	0.00000589
MAK	male germ cell associated kinase	-2.214500409	0.09985667
LINC02133	long intergenic non-protein coding RNA 2133	-2.226190335	0.08805201
DLGAP2	DLG associated protein 2	-2.24378167	0.09540359
ABCA13	ATP binding cassette subfamily A member 13	-2.245413177	0.00024199
TGM2	transglutaminase 2	-2.252186429	0.02848277
COL6A2	collagen type VI alpha 2 chain	-2.328217135	1.85E-36
CDCP1	CUB domain containing protein 1	-2.353450283	0.00244048
CPZ	carboxypeptidase Z	-2.383829432	4.44E-12
COL1A2	collagen type I alpha 2 chain	-2.400950398	9.74E-14
TAS2R5	taste 2 receptor member 5	-2.403498924	0.08805201
MRS2	magnesium transporter MRS2	-2.425076599	0.07975729
STAT6	signal transducer and activator of transcription 6	-2.466986772	0.05548472

GREM1	gremlin 1; DAN family BMP antagonist	-2.49385433	4.15E-39
TOR4A	torsin family 4 member A	-2.511250749	0.00015874
SLC22A1	solute carrier family 22 member 1	-2.561594046	0.08805201
GPNMB	glycoprotein nmb	-2.577919101	0.00513946
ANPEP	alanyl aminopeptidase; membrane	-2.578687276	0.00000278
PRSS12	serine protease 12	-2.62010266	1.98E-27
CYBA	cytochrome b-245 alpha chain	-2.646637909	0.02174844
MYPN	myopalladin	-2.656615087	0.00112093
NEAT1	nuclear paraspeckle assembly transcript 1	-2.668900157	1.66E-14
DUXAP10	double homeobox A pseudogene 10	-2.680683815	0.06034561
ABI3BP	ABI family member 3 binding protein	-2.682043162	0.04276629
SH2D4A	SH2 domain containing 4A	-2.692444823	0.00054796
RGPD6	RANBP2 like and GRIP domain containing 6	-2.716263844	0.02244691
LRTOMT	leucine rich transmembrane and O-methyltransferase domain containing	-2.765565475	0.0935329
TNNT1	troponin T1; slow skeletal type	-2.775221425	0.06287813
LRRK1	leucine rich repeat kinase 1	-2.785801831	0.00606573
BNC1	basonuclin zinc finger protein 1	-2.810506268	0.00221368
THRB	thyroid hormone receptor beta	-2.824080715	0.07999567
ISL1	ISL LIM homeobox 1	-2.904196861	0.06527057
CLMP	CXADR like membrane protein	-2.904697215	0.00000203
SLC16A7	solute carrier family 16 member 7	-2.914118655	0.00487389
TBX15	T-box transcription factor 15	-2.983520749	0.02025434
CASP4	caspase 4	-3.075527416	0.00843923
FGF5	fibroblast growth factor 5	-3.185087096	4.14E-08
GOLGA8N	golgin A8 family member N	-3.207692481	0.00096331
TMEM200A	transmembrane protein 200A	-3.275756164	0.00733117
ECM1	extracellular matrix protein 1	-3.32507039	2.25E-20
SLC43A3	solute carrier family 43 member 3	-3.328882105	0.00072082
WNT5A	Wnt family member 5A	-3.397843481	9.13E-13
COL12A1	collagen type XII alpha 1 chain	-3.436294689	1.71E-20
ROR2	receptor tyrosine kinase like orphan receptor 2	-3.452667595	0.02582071
PARP8	poly(ADP-ribose) polymerase family member 8	-3.61470367	0.00313516
SLC16A3	solute carrier family 16 member 3	-3.661872383	9.7E-22

EMILIN2	elastin microfibril interfacer 2	-3.687473932	0.0000746
RAB3B	RAB3B; member RAS oncogene family	-3.739531931	3.03E-11
HTR1F	5-hydroxytryptamine receptor 1F	-3.775600711	0.05530858
LY6K	lymphocyte antigen 6 family member K	-3.806745373	1.67E-08
RAC2	Rac family small GTPase 2	-4.110538945	0.00104658
BGN	biglycan	-4.182321966	0.00000226
FAM167A	family with sequence similarity 167 member A	-4.191361017	0.0000086
INHBA	inhibin subunit beta A	-4.232375957	0.00367542
CASP10	caspase 10	-4.277422672	0.02772468
PRRX2	paired related homeobox 2	-4.300158217	0.00116254
LTBR	lymphotoxin beta receptor	-4.364833472	2.91E-08
PEAR1	platelet endothelial aggregation receptor 1	-4.485274737	0.00031397
HTATIP2	HIV-1 Tat interactive protein 2	-4.548058564	0.04377784
ABLIM3	actin binding LIM protein family member 3	-4.622756529	0.0000149
GALNT6	polypeptide N-acetylgalactosaminyltransferase 6	-4.644696095	0.00341174
CDK15	cyclin dependent kinase 15	-4.796340625	0.00051709
GPRC5A	G protein-coupled receptor class C group 5 member A	-4.950151857	4.14E-08
NLRP1	NLR family pyrin domain containing 1	-5.111576541	0.03076337
NCAM2	neural cell adhesion molecule 2	-5.162489963	0.00155225
WNK4	WNK lysine deficient protein kinase 4	-5.225913045	9.75E-07
TFAP2A	transcription factor AP-2 alpha	-5.261723485	0.00395359
AOX1	aldehyde oxidase 1	-5.296882833	3.3E-12
VRK2	VRK serine/threonine kinase 2	-5.401414422	0.00233831
PSG4	pregnancy specific beta-1-glycoprotein 4	-5.48452631	0.0019481
CFH	complement factor H	-5.686147776	0.01289126
LINC01116	long intergenic non-protein coding RNA 1116	-5.71934334	0.00898349
CCBE1	collagen and calcium binding EGF domains 1	-5.723241133	4.98E-09
FOXD1	forkhead box D1	-5.802025585	0.00000401
IL1B	interleukin 1 beta	-5.845224372	0.00169394
FOXF1	forkhead box F1	-5.885026995	0.05246979
VEGFC	vascular endothelial growth factor C	-6.007990155	8.05E-07

ENG	endoglin	-6.016686177	0.00012827
CLEC14A	C-type lectin domain containing 14A	-6.435208505	0.00062686
MME	membrane metalloendopeptidase	-6.445688988	0.00016032
HOXA9	homeobox A9	-6.479136049	0.00088655
SHOX2	SHOX homeobox 2	-6.541901784	0.07543068
QNG1	Q-nucleotide N-glycosylase 1	-6.584443171	0.00099386
FLI1	Fli-1 proto-oncogene; ETS transcription factor	-6.669224095	0.00156699
CCN4	cellular communication network factor 4	-6.692372546	0.0444207
FOXL1	forkhead box L1	-6.709924427	0.00038723
IGF2	insulin like growth factor 2	-6.825344828	0.00000541
FXYD5	FXYD domain containing ion transport regulator 5	-6.907125035	6.46E-17
EBF3	EBF transcription factor 3	-7.11578629	0.00622287
COL13A1	collagen type XIII alpha 1 chain	-7.149336128	0.00096331
SLC17A9	solute carrier family 17 member 9	-7.338674203	0.00000547
MT1E	metallothionein 1E	-7.40443634	0.00023118
ADAMTS2	ADAM metallopeptidase with thrombospondin type 1 motif 2	-7.464508301	2.99E-07
CDH13	cadherin 13	-7.589487266	3.41E-13
TBX18	T-box transcription factor 18	-7.647711397	0.000015
COL6A3	collagen type VI alpha 3 chain	-7.67379628	0.02094244
KRT34	keratin 34	-7.727535944	0.0000624
GPAT2	glycerol-3-phosphate acyltransferase 2; mitochondrial	-7.819253551	8.53E-08
FOXC2	forkhead box C2	-8.209990406	8.33E-08
NR2F2	nuclear receptor subfamily 2 group F member 2	-8.210136219	8.99E-28
SERPINB2	serpin family B member 2	-8.232475512	2.52E-09
COL3A1	collagen type III alpha 1 chain	-8.424678831	1.38E-11
SRGN	serglycin	-8.957290765	0.00045482
MLPH	melanophilin	-8.959249973	1.7E-11
DKK1	dickkopf WNT signaling pathway inhibitor 1	-9.757886984	5.57E-26
UTP14C	UTP14C small subunit processome component	-12.08739729	0.04376008

Supplementary Table 16: DEGs in Propionate vs Control conditions (log2FoldChange ≤ -0.59 or ≥ +0.59, adjusted p-value< 0.1)

Gene symbol	Gene name	log2FoldChange	padj
SPECC1L-ADORA2A	SPECC1L-ADORA2A readthrough (NMD candidate)	19.55423776	0.000222547
EIF3EP1	EIF3E pseudogene 1	5.99655321	0.029722914
ZIC3	Zic family member 3	4.354399421	0.024628722
MGAT4C	MGAT4 family member C	2.893973058	0.000455991
GJB2	gap junction protein beta 2	2.661619846	0.008793845
CPLX2	complexin 2	2.560625299	0.000306217
HEY1	hes related family bHLH transcription factor with YRPW motif 1	2.524269803	0.032080103
ATP13A4	ATPase 13A4	2.488124736	0.049811817
COL25A1	collagen type XXV alpha 1 chain	2.466307289	0.09334829
RLBP1	retinaldehyde binding protein 1	2.427049238	0.045051648
KLHL4	kelch like family member 4	2.251346097	0.017616062
MYT1	myelin transcription factor 1	2.197615446	0.048099313
NKILA	NF-kappaB interacting lncRNA	2.136668713	0.024759488
NPL	N-acetylneuraminate pyruvate lyase	2.113252645	0.000158557
CHRD	chordin	2.022917755	0.048857788
SERPINA3	serpin family A member 3	1.938034357	0.06464515
CILK1	ciliogenesis associated kinase 1	1.900979965	0.002801111
RGS8	regulator of G protein signaling 8	1.861954267	1.03E-09
DENND1C	DENN domain containing 1C	1.859689194	0.002456797
NTRK2	neurotrophic receptor tyrosine kinase 2	1.783091273	0.00000255
VAV3	vav guanine nucleotide exchange factor 3	1.728427532	0.007475476
NTNG2	netrin G2	1.687824864	0.022039454
DNM3	dynamin 3	1.574733628	0.004770561
PKP1	plakophilin 1	1.529305782	0.0000154
MMP7	matrix metallopeptidase 7	1.523648847	0.044298128
DIPK1C	divergent protein kinase domain 1C	1.450778216	0.001011018
SORCS2	sortilin related VPS10 domain containing receptor 2	1.441955001	0.000182755
RGR	retinal G protein coupled receptor	1.404663903	0.064569115
ATCAY	ATCAY kinesin light chain interacting caytaxin	1.341230174	0.018831437
RBMS3	RNA binding motif single stranded interacting protein 3	1.315109677	0.009108902
NDRG2	NDRG family member 2	1.284745636	0.003207756
PCDH1	protocadherin 1	1.281617848	0.0000234
COL14A1	collagen type XIV alpha 1 chain	1.235854463	0.000108619
C4B	complement C4B (Chido blood group)	1.204699774	0.007217214
SPRN	shadow of prion protein	1.191618637	0.010247798

SERPINF1	serpin family F member 1	1.187066586	0.00000267
PMEPA1	prostate transmembrane protein; androgen induced 1	1.186475984	4.65E-15
CRMP1	collapsin response mediator protein 1	1.175170724	1.61E-08
DLGAP1	DLG associated protein 1	1.163238769	0.00013599
SPOCK1	SPARC (osteonectin); cwcv and kazal like domains proteoglycan 1	1.139072073	0.000195144
ZSCAN31	zinc finger and SCAN domain containing 31	1.135555766	0.00030684
ANGPTL1	angiopoietin like 1	1.135096085	7.34E-08
CACNG7	calcium voltage-gated channel auxiliary subunit gamma 7	1.134839914	0.0000266
ENTREP2	endosomal transmembrane epsin interactor 2	1.1233177	0.029424253
ARID3A	AT-rich interaction domain 3A	1.101648465	0.083678663
PRDM1	PR/SET domain 1	1.073792177	0.00092507
DLX5	distal-less homeobox 5	1.067171983	0.000143409
ZNF175	zinc finger protein 175	1.037400199	0.044471927
SEZ6L	seizure related 6 homolog like	1.021070594	0.027078423
RASL11B	RAS like family 11 member B	0.984444238	0.066575977
RGS16	regulator of G protein signaling 16	0.97919153	0.0000505
RNF180	ring finger protein 180	0.968402437	0.000232053
TOX3	TOX high mobility group box family member 3	0.966128112	0.00000848
SCD5	stearoyl-CoA desaturase 5	0.965197395	7.8E-11
HBA1	hemoglobin subunit alpha 1	0.948656573	0.001070789
NFE2L3	NFE2 like bZIP transcription factor 3	0.943756637	0.00936936
ACVR2A	activin A receptor type 2A	0.942463752	0.000000025
ROR1	receptor tyrosine kinase like orphan receptor 1	0.936993191	0.049990406
INSM1	INSM transcriptional repressor 1	0.931951735	0.008762485
GIN1	gypsy retrotransposon integrase 1	0.903330066	0.084026798
PCDH19	protocadherin 19	0.87916551	0.000232053
IGFBP5	insulin like growth factor binding protein 5	0.878503513	0.00000117
FBXO25	F-box protein 25	0.858502996	0.022637051
ZSCAN12	zinc finger and SCAN domain containing 12	0.857976745	0.075252089
HDC	histidine decarboxylase	0.844370161	0.030045816
BCAN	brevican	0.841455614	0.085630174
HOXB3	homeobox B3	0.838504938	0.009108902
PAK3	p21 (RAC1) activated kinase 3	0.836078744	0.000851461
SFMBT2	Scm like with four mbt domains 2	0.815707276	0.023745047
TMEM178B	transmembrane protein 178B	0.783025402	0.089644718
ANK3	ankyrin 3	0.779522577	0.034699358
MFAP4	microfibril associated protein 4	0.764879221	0.030674058

SP4	Sp4 transcription factor	0.759953242	0.00000536
LYN	LYN proto-oncogene; Src family tyrosine kinase	0.751521784	0.065421117
TNFRSF19	TNF receptor superfamily member 19	0.743446149	0.000945972
ST8SIA5	ST8 alpha-N-acetyl-neuraminide alpha-2;8-sialyltransferase 5	0.724432741	0.032297887
PTPN4	protein tyrosine phosphatase non-receptor type 4	0.709884968	0.083907218
DAB1	DAB adaptor protein 1	0.702945155	0.005273836
ACTG2	actin gamma 2; smooth muscle	0.702396915	0.013305858
ARHGAP42	Rho GTPase activating protein 42	0.694071961	0.002008231
DMRTA2	DMRT like family A2	0.682627452	0.048583318
JAKMIP2-AS1	JAKMIP2 antisense RNA 1	0.68101684	0.000491784
SULF2	sulfatase 2	0.674772732	0.00105745
CD24	CD24 molecule	0.671077683	0.014155304
ANOS1	anosmin 1	0.669256691	0.078599729
MAST1	microtubule associated serine/threonine kinase 1	0.66804746	0.065437803
GNG2	G protein subunit gamma 2	0.666274555	0.000208572
MYH14	myosin heavy chain 14	0.665834472	0.083557694
VWA1	von Willebrand factor A domain containing 1	0.659415508	0.019429798
FBXL16	F-box and leucine rich repeat protein 16	0.651694071	0.024308206
ESPN	espin	0.650935322	0.048857788
MCCC2	methylcrotonyl-CoA carboxylase subunit 2	0.64486275	0.0000123
LCORL	ligand dependent nuclear receptor corepressor like	0.643977485	0.076903707
ND3	NADH dehydrogenase subunit 3	0.620362668	0.00000044
ZNF782	zinc finger protein 782	0.618192914	0.083833237
PXDN	peroxidasin	0.598583647	0.003785688
GNG4	G protein subunit gamma 4	0.596439776	0.000120673
JADE3	jade family PHD finger 3	0.594751972	0.000851461
TSPAN15	tetraspanin 15	0.594645928	0.0864399
MIR99AHG	mir-99a-let-7c cluster host gene	-0.590046757	0.072950995
ZBTB38	zinc finger and BTB domain containing 38	-0.590944997	0.038578032
S100A6	S100 calcium binding protein A6	-0.593440629	0.093971583
ZNF69	zinc finger protein 69	-0.600884142	0.089644718
MAFF	MAF bZIP transcription factor F	-0.602706961	0.041104144
ECHDC3	enoyl-CoA hydratase domain containing 3	-0.616839231	0.053778685
HSPA1A	heat shock protein family A (Hsp70) member 1A	-0.617032793	0.072603787
GPR161	G protein-coupled receptor 161	-0.621877752	0.036011072

ZC3H8	zinc finger CCCH-type containing 8	-0.622016342	0.011622399
FBN2	fibrillin 2	-0.624978754	0.001394388
SDC4	syndecan 4	-0.628128121	0.000963785
TMPRSS5	transmembrane serine protease 5	-0.629012906	0.068643454
AXL	AXL receptor tyrosine kinase	-0.630031878	0.00000347
GABRQ	gamma-aminobutyric acid type A receptor subunit theta	-0.630445039	0.004709368
ADAMTS9	ADAM metallopeptidase with thrombospondin type 1 motif 9	-0.630872569	0.059565382
HEG1	heart development protein with EGF like domains 1	-0.640937837	0.005050609
NBEA	neurobeachin	-0.641538542	0.009385939
CSPG4	chondroitin sulfate proteoglycan 4	-0.643499574	0.044298128
F3	coagulation factor III; tissue factor	-0.651806388	0.000000037
SEZ6	seizure related 6 homolog	-0.65228565	0.008001587
FRMD6	FERM domain containing 6	-0.652923267	0.082958784
MYO18A	myosin XVIIIA	-0.661510912	0.000000108
SLC1A2	solute carrier family 1 member 2	-0.663212776	0.0000733
PDE8B	phosphodiesterase 8B	-0.667083021	0.074101048
ITGB3	integrin subunit beta 3	-0.675834213	0.026193131
LRP2	LDL receptor related protein 2	-0.677201171	0.003747153
RFX4	regulatory factor X4	-0.678538656	0.004470017
LRRC15	leucine rich repeat containing 15	-0.68357222	0.005245087
WLS	Wnt ligand secretion mediator	-0.695914009	0.000566849
NRP1	neuropilin 1	-0.700717351	0.000640358
CARD16	caspase recruitment domain family member 16	-0.702163854	0.01758875
PDE3A	phosphodiesterase 3A	-0.721276441	0.007525103
UTRN	utrophin	-0.728306515	0.006402039
ITGA5	integrin subunit alpha 5	-0.730753144	0.000000603
TTLL3	tubulin tyrosine ligase like 3	-0.737439329	0.028540646
A2M	alpha-2-macroglobulin	-0.738483756	0.017637801
CDH6	cadherin 6	-0.746514519	0.00091543
ALPL	alkaline phosphatase; biomineralization associated	-0.751705032	0.070486917
CASQ1	calsequestrin 1	-0.754673858	0.004182743
FOXJ1	forkhead box J1	-0.755190246	0.000013
SH3BP1	SH3 domain binding protein 1	-0.779846239	0.061219745
GSDMD	gasdermin D	-0.787658266	0.047201552
ZNF483	zinc finger protein 483	-0.788890145	0.001246445
LOXL2	lysyl oxidase like 2	-0.789467765	0.0000275
CYP1B1	cytochrome P450 family 1 subfamily B member 1	-0.791020074	0.002401341
PLAUR	plasminogen activator; urokinase receptor	-0.793093844	0.00025733

SEMA6D	semaphorin 6D	-0.793513002	0.000146382
ENPP2	ectonucleotide pyrophosphatase/phosphodiesterase 2	-0.796877982	0.013305858
COCH	cochlin	-0.80645136	0.009331676
B3GAT2	beta-1;3-glucuronyltransferase 2	-0.806629721	0.040264482
OPLAH	5-oxoprolinase; ATP-hydrolysing	-0.814359655	0.017601987
C6orf118	chromosome 6 open reading frame 118	-0.822550562	0.001445053
TRIM71	tripartite motif containing 71	-0.825163157	0.091019941
TES	testin LIM domain protein	-0.832202284	0.06873589
SMTN	smoothelin	-0.833701354	0.000169629
TLR4	toll like receptor 4	-0.839866213	0.020386961
RBPMS2	RNA binding protein; mRNA processing factor 2	-0.842563758	0.072950995
ERBB4	erb-b2 receptor tyrosine kinase 4	-0.845425198	0.005561936
DTX4	deltex E3 ubiquitin ligase 4	-0.848593135	0.026919622
ATP1A2	ATPase Na+/K+ transporting subunit alpha 2	-0.853522537	0.059565382
RBMS1	RNA binding motif single stranded interacting protein 1	-0.85527312	0.095581524
ETS2	ETS proto-oncogene 2; transcription factor	-0.857409511	0.071096906
PGM5	phosphoglucomutase 5	-0.863663335	0.005134773
SYNE2	spectrin repeat containing nuclear envelope protein 2	-0.864222692	0.001209279
TEK	TEK receptor tyrosine kinase	-0.86712624	0.000053
CNR1	cannabinoid receptor 1	-0.880394892	0.042439128
VPS13D	vacuolar protein sorting 13 homolog D	-0.880743538	0.000221051
DGKH	diacylglycerol kinase eta	-0.884174727	0.016385912
TBC1D2	TBC1 domain family member 2	-0.889955693	0.007033131
COLEC12	collectin subfamily member 12	-0.898514956	0.054695482
CXCL12	C-X-C motif chemokine ligand 12	-0.902607261	0.033347712
TRERF1	transcriptional regulating factor 1	-0.904842676	0.073062139
B3GNT9	UDP-GlcNAc:betaGal beta-1;3-N-acetylglucosaminyltransferase 9	-0.909127951	0.046096858
SORL1	sortilin related receptor 1	-0.928969824	0.000000077
TRPV3	transient receptor potential cation channel subfamily V member 3	-0.93193381	0.014271042
JHY	junctional cadherin complex regulator	-0.934939683	0.012076277
COL5A1	collagen type V alpha 1 chain	-0.937169395	0.002378501
AHNAK	AHNAK nucleoprotein	-0.946290026	0.000186588
SEPT5-GP1BB	SEPT5-GP1BB readthrough	-0.948013538	0.05573078
CDC42BPG	CDC42 binding protein kinase gamma	-0.957182065	0.066533368
ATP8B1	ATPase phospholipid transporting 8B1	-0.969811194	0.05573078
ANKRD63	ankyrin repeat domain 63	-0.973335542	0.007013255
HKDC1	hexokinase domain containing 1	-0.976739986	1.05E-08

B3GALT1	beta-1;3-galactosyltransferase 1	-0.981012696	0.0000183
FAM229A	family with sequence similarity 229 member A	-0.990832638	0.056698906
FMN1	formin 1	-0.999555341	0.01600292
ATOH8	atonal bHLH transcription factor 8	-1.001776927	0.00105745
INSYN2B	inhibitory synaptic factor family member 2B	-1.010206158	0.040481275
XYLT1	xylosyltransferase 1	-1.017200038	9.25E-08
SFRP4	secreted frizzled related protein 4	-1.021892241	1.71E-08
BDNF	brain derived neurotrophic factor	-1.034242591	0.048607747
CTSH	cathepsin H	-1.040351866	0.070763544
TRMT9B	tRNA methyltransferase 9B (putative)	-1.041617077	0.043071444
IER3	immediate early response 3	-1.043415438	3.04E-09
RYR2	ryanodine receptor 2	-1.063274961	0.00000594
PPP1R13L	protein phosphatase 1 regulatory subunit 13 like	-1.064782735	0.061219745
FHIP1A	FHF complex subunit HOOK interacting protein 1A	-1.094705633	0.01943836
FAT3	FAT atypical cadherin 3	-1.095802646	0.06554146
CFAP126	cilia and flagella associated protein 126	-1.099207704	0.017262338
EMILIN1	elastin microfibril interfacer 1	-1.121460292	0.000296909
MFAP5	microfibril associated protein 5	-1.123515344	5.48E-11
TGFBI	transforming growth factor beta induced	-1.127651789	0.000572922
SIAH3	siah E3 ubiquitin protein ligase family member 3	-1.14541262	0.000534459
G0S2	G0/G1 switch 2	-1.146984742	0.007524202
CTDSPL	CTD small phosphatase like	-1.157406348	0.021033999
IL1R1	interleukin 1 receptor type 1	-1.160183124	0.018189222
FAM181A	family with sequence similarity 181 member A	-1.170719456	0.0000611
ADAMTSL1	ADAMTS like 1	-1.174559778	0.000108619
TMEM132E	transmembrane protein 132E	-1.177450178	0.00000502
TC2N	tandem C2 domains; nuclear	-1.184115495	0.00000698
IGFBP4	insulin like growth factor binding protein 4	-1.1922162	0.00000719
CHRFAM7A	CHRNA7 (exons 5-10) and FAM7A (exons A-E) fusion	-1.197704355	0.021039484
MMRN1	multimerin 1	-1.20700287	4.23E-12
WNT5B	Wnt family member 5B	-1.208108913	0.0000956
AHRR	aryl hydrocarbon receptor repressor	-1.229866914	0.093994822
COL5A2	collagen type V alpha 2 chain	-1.239012275	0.000372242
SERPINE1	serpin family E member 1	-1.244156412	5.56E-16
HMGA2	high mobility group AT-hook 2	-1.244461815	0.00000176
FANCD2	FA complementation group D2	-1.260763664	0.0008312
ELMOD1	ELMO domain containing 1	-1.261111801	2.88E-12

FUT9	fucosyltransferase 9	-1.28374134	0.00000898
EHBP1L1	EH domain binding protein 1 like 1	-1.289962802	0.003398746
TGIF2-RAB5IF	TGIF2-RAB5IF readthrough	-1.294982341	0.072603787
SLC6A16	solute carrier family 6 member 16	-1.298846891	0.019429798
EPAS1	endothelial PAS domain protein 1	-1.309760428	0.0000744
EMP1	epithelial membrane protein 1	-1.320873205	6.73E-08
SCEL	sciellin	-1.325859403	0.077793248
PCOLCE	procollagen C-endopeptidase enhancer	-1.332214335	0.0000375
SLFN5	schlafen family member 5	-1.350038636	0.013305858
LOC100505715	uncharacterized LOC100505715	-1.360767702	0.066575977
TFPI	tissue factor pathway inhibitor	-1.371213026	0.000275771
ST3GAL1	ST3 beta-galactoside alpha-2;3-sialyltransferase 1	-1.390370825	0.007020432
CHST9	carbohydrate sulfotransferase 9	-1.417844552	0.099059758
CAVIN2	caveolae associated protein 2	-1.450613233	0.052206194
STC2	stanniocalcin 2	-1.452071574	8.27E-20
PAPSS2	3'-phosphoadenosine 5'-phosphosulfate synthase 2	-1.453230067	0.061219745
CCDC80	coiled-coil domain containing 80	-1.458169724	0.000158557
MGST1	microsomal glutathione S-transferase 1	-1.472391942	6.44E-08
GALNT5	polypeptide N-acetylgalactosaminyltransferase 5	-1.488323114	5.29E-19
NMRAL1	NmrA like redox sensor 1	-1.496726291	0.061115359
MYRF	myelin regulatory factor	-1.502122865	0.055954936
GATA2	GATA binding protein 2	-1.525243147	0.048576989
NWD1	NACHT and WD repeat domain containing 1	-1.528435951	0.051421754
LINC00488	long intergenic non-protein coding RNA 488	-1.54538282	0.059430669
PTGS1	prostaglandin-endoperoxide synthase 1	-1.549664171	0.061044181
RCN3	reticulocalbin 3	-1.609186136	0.006497908
DSP	desmoplakin	-1.612627848	5.78E-11
ABCA13	ATP binding cassette subfamily A member 13	-1.613397196	0.052151615
SCAND3	SCAN domain containing 3	-1.619314762	0.003176595
RPL23AP21	ribosomal protein L23a pseudogene 21	-1.619570775	0.068275371
JCAD	junctional cadherin 5 associated	-1.624876831	0.00211148
CACNA1C	calcium voltage-gated channel subunit alpha1 C	-1.625901022	0.000777549
KRT18	keratin 18	-1.628302254	1.19E-11
FLG	filaggrin	-1.630328229	1.25E-11
LRRC2	leucine rich repeat containing 2	-1.649262534	0.000118063
ESRG	embryonic stem cell related	-1.676542423	0.022453324
SLIT3	slit guidance ligand 3	-1.678658946	6.43E-10
MT2A	metallothionein 2A	-1.691475903	2.14E-17

MOK	MOK protein kinase	-1.727677918	1.36E-12
IL7R	interleukin 7 receptor	-1.728944442	0.028760334
ALDH3A1	aldehyde dehydrogenase 3 family member A1	-1.733305896	0.00000184
CHODL	chondrolectin	-1.748458207	0.011079704
HECW2	HECT; C2 and WW domain containing E3 ubiquitin protein ligase 2	-1.754849005	6.43E-10
OTULINL	OTU deubiquitinase with linear linkage specificity like	-1.775622611	0.020175937
TRIM38	tripartite motif containing 38	-1.778295383	0.051421754
CHRM3	cholinergic receptor muscarinic 3	-1.802263736	0.000838186
LINC01123	long intergenic non-protein coding RNA 1123	-1.816580315	0.012127522
H1-0	H1,0 linker histone	-1.842450666	1.25E-11
MYL9	myosin light chain 9	-1.885145652	1.51E-08
FLNC	filamin C	-1.894059012	9.56E-25
TRABD2A	TraB domain containing 2A	-1.90720477	0.014360216
HMOX1	heme oxygenase 1	-1.957062444	0.004770561
WNT5A	Wnt family member 5A	-1.959254595	0.006265138
GDF10	growth differentiation factor 10	-1.963714528	0.000224118
DNER	delta/notch like EGF repeat containing	-1.967251318	0.0000166
OXTR	oxytocin receptor	-2.023615581	0.000108619
CLDN11	claudin 11	-2.02932517	0.000118063
TCEA3	transcription elongation factor A3	-2.055504865	0.020175937
VWF	von Willebrand factor	-2.06261614	0.038374167
FN1	fibronectin 1	-2.063024795	1.21E-27
CPZ	carboxypeptidase Z	-2.066276926	0.000221868
ADAM8	ADAM metallopeptidase domain 8	-2.081927715	0.074108323
GOLGA8N	golgin A8 family member N	-2.095682979	0.007190743
BICC1	BicC family RNA binding protein 1	-2.103407609	1.97E-10
ALDH1L2	aldehyde dehydrogenase 1 family member L2	-2.108610108	0.019960387
TMEM200B	transmembrane protein 200B	-2.112165922	0.079948634
SEMA7A	semaphorin 7A (John Milton Hagen blood group)	-2.130250279	0.000000207
WDR38	WD repeat domain 38	-2.135145263	0.079372489
PRSS12	serine protease 12	-2.138859108	2.26E-08
CDCP1	CUB domain containing protein 1	-2.169739606	0.012805742
TBX15	T-box transcription factor 15	-2.181579282	0.064011145
PSORS1C1	psoriasis susceptibility 1 candidate 1	-2.241720467	0.037955163
LRRK1	leucine rich repeat kinase 1	-2.24348155	0.050109658
COL6A2	collagen type VI alpha 2 chain	-2.255908392	2.77E-36
SOD3	superoxide dismutase 3	-2.257298442	0.083608897
TGM2	transglutaminase 2	-2.316224955	0.022166955
PROX1-AS1	PROX1 antisense RNA 1	-2.318415823	0.049628884

MRGPRF	MAS related GPR family member F	-2.369452149	0.000000501
TOR4A	torsin family 4 member A	-2.387234058	0.000808998
TAS2R4	taste 2 receptor member 4	-2.401937561	0.06634276
NEAT1	nuclear paraspeckle assembly transcript 1	-2.43350759	3.69E-11
PDZD2	PDZ domain containing 2	-2.499484742	0.004725076
CYBA	cytochrome b-245 alpha chain	-2.502146555	0.012811129
ETS1	ETS proto-oncogene 1; transcription factor	-2.613299671	6.1E-17
COL1A2	collagen type I alpha 2 chain	-2.620946812	2.22E-13
CLMP	CXADR like membrane protein	-2.635761777	0.001352818
ABI3BP	ABI family member 3 binding protein	-2.647623174	0.032970365
CAPG	capping actin protein; gelsolin like	-2.65990195	0.000299538
GREM1	gremlin 1; DAN family BMP antagonist	-2.694239499	2.06E-51
RGPD6	RANBP2 like and GRIP domain containing 6	-2.697461208	0.093971583
BNC1	basonuclin zinc finger protein 1	-2.702765965	0.0000611
ECM1	extracellular matrix protein 1	-2.707589381	2.89E-11
GPNMB	glycoprotein nmb	-2.740375066	0.027253378
PAPPA	pappalysin 1	-2.809978215	0.00000591
SH2D4A	SH2 domain containing 4A	-2.964207746	0.0000306
EMILIN2	elastin microfibril interfacer 2	-3.028754405	0.015997784
FGF5	fibroblast growth factor 5	-3.059255543	0.000000015
MYPN	myopalladin	-3.060264266	0.000455991
SLITRK2	SLIT and NTRK like family member 2	-3.118793676	0.0336506
MME	membrane metalloendopeptidase	-3.203662614	0.049628884
LRTOMT	leucine rich transmembrane and O-methyltransferase domain containing	-3.209907438	0.061687786
ANPEP	alanyl aminopeptidase; membrane	-3.26623619	7.02E-19
COPZ2	COPI coat complex subunit zeta 2	-3.279345824	0.047730873
ROR2	receptor tyrosine kinase like orphan receptor 2	-3.40876797	0.001639327
THRB	thyroid hormone receptor beta	-3.410558722	0.054118863
STAT6	signal transducer and activator of transcription 6	-3.423484703	0.00115597
RAB3B	RAB3B; member RAS oncogene family	-3.444344507	3.1E-14
MRS2	magnesium transporter MRS2	-3.499154532	0.049672897
DUXAP10	double homeobox A pseudogene 10	-3.541508485	0.000414225
PEAR1	platelet endothelial aggregation receptor 1	-3.57671162	0.005050609
FAM167A	family with sequence similarity 167 member A	-3.737162411	0.002962313
HTATIP2	HIV-1 Tat interactive protein 2	-3.898257147	0.004526725
PID1	phosphotyrosine interaction domain containing 1	-3.941643226	0.03677841
LY6K	lymphocyte antigen 6 family member K	-3.943134453	1.2E-09

SLC17A9	solute carrier family 17 member 9	-3.998423745	0.095336582
ARHGAP24	Rho GTPase activating protein 24	-4.130757013	0.074129506
LTBR	lymphotoxin beta receptor	-4.157187953	0.00000092
COL12A1	collagen type XII alpha 1 chain	-4.186382853	3.36E-35
RAC2	Rac family small GTPase 2	-4.263852501	0.000799625
FOXD1	forkhead box D1	-4.265528626	1.89E-09
INHBA	inhibin subunit beta A	-4.286906426	0.004770561
BGN	biglycan	-4.434294731	0.00000383
SLC16A3	solute carrier family 16 member 3	-4.444934354	2.82E-35
ALDH1A2	aldehyde dehydrogenase 1 family member A2	-4.466792099	0.098632598
CASP10	caspase 10	-4.636361333	0.017616062
APBB1IP	amyloid beta precursor protein binding family B member 1 interacting protein	-4.727243338	0.028564827
GPRC5A	G protein-coupled receptor class C group 5 member A	-4.742358702	0.00000944
NLRP1	NLR family pyrin domain containing 1	-4.769995042	0.012805742
RET	ret proto-oncogene	-4.808234763	0.032168755
VEGFC	vascular endothelial growth factor C	-4.885757774	0.0000611
WNK4	WNK lysine deficient protein kinase 4	-4.909146574	0.00002
CDH13	cadherin 13	-4.956600807	3.39E-08
ABLIM3	actin binding LIM protein family member 3	-5.019314033	0.000013
TFAP2A	transcription factor AP-2 alpha	-5.06506535	0.000737583
IGF2	insulin like growth factor 2	-5.073489363	0.0000288
CCBE1	collagen and calcium binding EGF domains 1	-5.309381432	3.17E-08
SLC43A3	solute carrier family 43 member 3	-5.322180729	1.44E-08
AOX1	aldehyde oxidase 1	-5.485179519	2.22E-13
GALNT6	polypeptide N-acetylgalactosaminyltransferase 6	-5.51826934	0.000474679
PRRX2	paired related homeobox 2	-5.523615948	0.000102429
SERPINB7	serpin family B member 7	-5.608388183	0.050109658
IL1B	interleukin 1 beta	-5.666394234	0.004984351
PARP8	poly(ADP-ribose) polymerase family member 8	-5.82844013	0.002330864
COL3A1	collagen type III alpha 1 chain	-5.885124444	0.000000414
CPED1	cadherin like and PC-esterase domain containing 1	-6.095434999	0.000619503
FOXF1	forkhead box F1	-6.118810432	0.05817289
FLI1	Fli-1 proto-oncogene; ETS transcription factor	-6.156457433	0.005273836
CLEC14A	C-type lectin domain containing 14A	-6.376038471	0.000315656
MSC-AS1	MSC antisense RNA 1	-6.55657622	0.092505465
FXYD5	FXYD domain containing ion transport regulator 5	-6.565270314	0.009424349

CCN4	cellular communication network factor 4	-6.633670476	0.070763544
ENG	endoglin	-6.655651074	0.00000112
MT1E	metallothionein 1E	-6.728044131	0.0000749
KRTAP2-3	keratin associated protein 2-3	-6.775540412	0.044471927
ADAMTS2	ADAM metallopeptidase with thrombospondin type 1 motif 2	-6.786740509	0.000272322
KRT34	keratin 34	-6.937680077	0.000252147
FOXC2	forkhead box C2	-6.98542122	0.00000428
HOXA9	homeobox A9	-7.02800998	0.012479541
EBF3	EBF transcription factor 3	-7.055362721	0.010638723
VRK2	VRK serine/threonine kinase 2	-7.056977425	0.002644522
FOXL1	forkhead box L1	-7.185084417	0.000696645
MLPH	melanophilin	-7.42227051	0.000000284
NR2F2	nuclear receptor subfamily 2 group F member 2	-7.583592014	1.35E-18
DKK1	dickkopf WNT signaling pathway inhibitor 1	-7.841304257	0.003534961
COL6A3	collagen type VI alpha 3 chain	-8.20387443	0.015997784
TBX3	T-box transcription factor 3	-9.004571751	1.12E-11
ADAMTS1	ADAM metallopeptidase with thrombospondin type 1 motif 1	-9.907509421	8.63E-18

Supplementary Table 17: DEGs in Butyrate vs Control conditions (log2FoldChange ≤ -0.59 or ≥ +0.59, adjusted p-value< 0.1)

Gene symbol	Gene name	log2FoldChange	padj
IFIT2	interferon induced protein with tetratricopeptide repeats 2	19.27546025	0.000220884
CCDC87	coiled-coil domain containing 87	4.239118314	0.091855234
ZIC3	Zic family member 3	4.027733319	0.049203367
ATP13A4	ATPase 13A4	2.980856121	0.031358385
HSPB8	heat shock protein family B (small) member 8	2.970176538	0.036223653
MGAT4C	MGAT4 family member C	2.842458562	0.006189732
CPLX2	complexin 2	2.765891912	0.011641998
MASP1	MBL associated serine protease 1	2.747889558	0.00153318
MYT1	myelin transcription factor 1	2.571653303	0.011510876
B3GALT2	beta-1;3-galactosyltransferase 2	2.492004252	0.034726991
NPL	N-acetylneuraminate pyruvate lyase	2.412014678	0.000101346
COL25A1	collagen type XXV alpha 1 chain	2.29626399	0.023174271
GJB2	gap junction protein beta 2	2.197001254	0.057059998
NKILA	NF-kappaB interacting lncRNA	2.009741702	0.051245481
CHRD	chordin	1.942261259	0.034846971
NTNG2	netrin G2	1.840591923	0.082045233
NRXN3	neurexin 3	1.803640715	0.003320638
NTRK2	neurotrophic receptor tyrosine kinase 2	1.798885026	0.00000511
RGS8	regulator of G protein signaling 8	1.777775654	0.000000314
DNM3	dynamin 3	1.748154695	0.001080979
DENND1C	DENN domain containing 1C	1.729806486	0.006532541
KLHL4	kelch like family member 4	1.697040842	0.053252438
ANKRD44	ankyrin repeat domain 44	1.647301429	0.082355632
SERPINA3	serpin family A member 3	1.638782486	0.084490342
TUBB4A	tubulin beta 4A class IVa	1.608338355	0.04928197
TEC	tec protein tyrosine kinase	1.57295043	0.052306091
RGR	retinal G protein coupled receptor	1.490126619	0.042146463
LDLRAD4	low density lipoprotein receptor class A domain containing 4	1.439640237	0.000852794
MMP7	matrix metallopeptidase 7	1.408719527	0.064108749
SPOCK1	SPARC (osteonectin); cwcv and kazal like domains proteoglycan 1	1.366606061	0.014496833
ELFN2	extracellular leucine rich repeat and fibronectin type III domain containing 2	1.356720138	0.07435086
DIPK1C	divergent protein kinase domain 1C	1.286923113	0.003669949
PCDH1	protocadherin 1	1.27648167	0.00000772
SSPN	sarcospan	1.257792153	0.000000728
SPNS2	SPNS lysolipid transporter 2; sphingosine-1-phosphate	1.255945013	0.093376081

DLGAP1	DLG associated protein 1	1.254093852	0.006814399
SPRN	shadow of prion protein	1.237884885	0.004453574
SORCS2	sortilin related VPS10 domain containing receptor 2	1.225929159	0.0000411
CRMP1	collapsin response mediator protein 1	1.196126091	1.54E-11
PMEPA1	prostate transmembrane protein; androgen induced 1	1.175304424	1.75E-09
ST18	ST18 C2H2C-type zinc finger transcription factor	1.166728106	0.008108295
STMN4	stathmin 4	1.165718775	0.041351096
ENTREP2	endosomal transmembrane epsin interactor 2	1.162271772	0.006945267
CACNG7	calcium voltage-gated channel auxiliary subunit gamma 7	1.159562824	5.35E-14
RBMS3	RNA binding motif single stranded interacting protein 3	1.137925655	0.087007686
SERPINF1	serpin family F member 1	1.125480197	0.0000504
NALF2	NALCN channel auxiliary factor 2	1.111319884	0.062750654
WNT11	Wnt family member 11	1.103885152	0.074221569
PKP1	plakophilin 1	1.079141319	0.005269991
NDRG2	NDRG family member 2	1.066669032	0.008060018
RGS16	regulator of G protein signaling 16	1.054540086	0.00000219
SHC2	SHC adaptor protein 2	1.032687174	0.044496432
BCAN	brevican	1.023376005	0.014456155
SCD5	stearoyl-CoA desaturase 5	0.981758327	6.89E-13
RNF180	ring finger protein 180	0.977084776	0.000481794
COL14A1	collagen type XIV alpha 1 chain	0.972998353	0.001935248
SCUBE2	signal peptide; CUB domain and EGF like domain containing 2	0.972811653	0.084490342
PRDM1	PR/SET domain 1	0.962367481	0.001044055
APCDD1	APC down-regulated 1	0.952253447	0.000918911
NFE2L3	NFE2 like bZIP transcription factor 3	0.935808297	0.001456946
IGFBP5	insulin like growth factor binding protein 5	0.911523161	0.000000306
ANGPTL1	angiopoietin like 1	0.903455294	0.0000266
TOX3	TOX high mobility group box family member 3	0.894742208	0.000000229
DLX5	distal-less homeobox 5	0.8864272	0.040660471
HBA1	hemoglobin subunit alpha 1	0.859413558	0.00042559
PXMP4	peroxisomal membrane protein 4	0.850432173	0.060973434
PCDH19	protocadherin 19	0.837124815	0.000149039
HOXB3	homeobox B3	0.827539107	0.017952908
GNG2	G protein subunit gamma 2	0.821192506	0.00000312
PAK3	p21 (RAC1) activated kinase 3	0.787009139	0.003573652
CPLX1	complexin 1	0.779233296	0.059785915
RIMS3	regulating synaptic membrane exocytosis 3	0.774940389	0.019645256

ZC3H6	zinc finger CCCH-type containing 6	0.771929667	0.077913922
JAKMIP2-AS1	JAKMIP2 antisense RNA 1	0.769301237	0.00000865
INSM1	INSM transcriptional repressor 1	0.764448727	0.004456501
ZNF175	zinc finger protein 175	0.758192903	0.087660838
CD24	CD24 molecule	0.751822491	0.002669214
LCORL	ligand dependent nuclear receptor corepressor like	0.744425303	0.06818824
ND3	NADH dehydrogenase subunit 3	0.740092022	2.97E-11
TNFRSF19	TNF receptor superfamily member 19	0.732742083	0.000361252
MFAP4	microfibril associated protein 4	0.718903048	0.029170177
MTND2P28	MT-ND2 pseudogene 28	0.716115631	0.000131591
FILIP1L	filamin A interacting protein 1 like	0.714238802	0.093600037
ST8SIA5	ST8 alpha-N-acetyl-neuraminide alpha-2;8-sialyltransferase 5	0.711879556	0.025029823
TLL2	tolloid like 2	0.696751316	0.06353504
MTND1P23	MT-ND1 pseudogene 23	0.692512821	0.000359713
GRIA4	glutamate ionotropic receptor AMPA type subunit 4	0.687024015	0.015938896
GNG4	G protein subunit gamma 4	0.679348937	7.08E-13
EPHB3	EPH receptor B3	0.67584129	0.00000865
ND2	NADH dehydrogenase subunit 2	0.674415421	0.000265216
SHROOM2	shroom family member 2	0.673817496	0.006664317
KCNN4	potassium calcium-activated channel subfamily N member 4	0.673076676	0.077908834
TMEM178B	transmembrane protein 178B	0.670799147	0.069942756
NOG	noggin	0.670322011	0.008530605
ATP6	ATP synthase F0 subunit 6	0.668918278	0.001356963
SFMBT2	Scm like with four mbt domains 2	0.668549207	0.090130949
RASSF4	Ras association domain family member 4	0.663945341	0.000910493
C1orf50	chromosome 1 open reading frame 50	0.65454935	0.008015994
MAST1	microtubule associated serine/threonine kinase 1	0.651109058	0.005240114
MTATP6P1	MT-ATP6 pseudogene 1	0.643105289	0.000912888
TSPAN15	tetraspanin 15	0.634861973	0.053716803
ND5	NADH dehydrogenase subunit 5	0.633137139	0.000906629
DMD	dystrophin	0.625214087	0.011756707
ANKRD49	ankyrin repeat domain 49	0.622246037	0.058697242
ATP8	ATP synthase F0 subunit 8	0.620622016	0.000315512
PHYHIPL	phytanoyl-CoA 2-hydroxylase interacting protein like	0.618258423	0.003343171

COX2	cytochrome c oxidase subunit II	0.617015477	0.000136551
PSEN2	presenilin 2	0.603088727	0.049534407
NEFH	neurofilament heavy chain	0.601590303	0.012150728
SLX1A-SULT1A3	SLX1A-SULT1A3 readthrough (NMD candidate)	0.598495957	0.004880769
ARRB1	arrestin beta 1	0.590372378	0.08316825
B3GAT1	beta-1;3-glucuronyltransferase 1	-0.590956056	0.001028633
MACF1	microtubule actin crosslinking factor 1	-0.595412636	0.00000187
SGK1	serum/glucocorticoid regulated kinase 1	-0.596461045	0.058697242
TTLL3	tubulin tyrosine ligase like 3	-0.598741374	0.02891968
HLA-DRB1	major histocompatibility complex; class II; DR beta 1	-0.599797014	0.036357123
HLA-DPA1	major histocompatibility complex; class II; DP alpha 1	-0.600404682	0.07435086
AXL	AXL receptor tyrosine kinase	-0.603128832	0.000000382
TLE3	TLE family member 3; transcriptional corepressor	-0.605996157	0.096164878
MAFF	MAF bZIP transcription factor F	-0.609807735	0.004275277
GABRQ	gamma-aminobutyric acid type A receptor subunit theta	-0.612047297	3.38E-09
FIGN	fidgetin; microtubule severing factor	-0.616568064	0.073741421
SDK1	sidekick cell adhesion molecule 1	-0.617010107	1.47E-14
ADCY9	adenylate cyclase 9	-0.619155856	0.000101346
CLTCL1	clathrin heavy chain like 1	-0.621729733	0.046038603
HEG1	heart development protein with EGF like domains 1	-0.622572324	0.0000202
UBR4	ubiquitin protein ligase E3 component n-recognin 4	-0.623754235	0.004082228
AFG3L1P	AFG3 like matrix AAA peptidase subunit 1; pseudogene	-0.629352694	0.004513691
FTH1	ferritin heavy chain 1	-0.629713542	0.0000315
S100A6	S100 calcium binding protein A6	-0.629915524	0.000793411
ITGA5	integrin subunit alpha 5	-0.635519501	0.0000443
SDC4	syndecan 4	-0.639480757	0.0000142
PLEKHG4B	pleckstrin homology and RhoGEF domain containing G4B	-0.641378095	1.03E-18
HLA-DRB5	major histocompatibility complex; class II; DR beta 5	-0.648011415	0.01745214
ZBTB38	zinc finger and BTB domain containing 38	-0.648843492	0.011542277
NBEA	neurobeachin	-0.648860229	0.001935248
DRD4	dopamine receptor D4	-0.649479055	0.019226047
NRP1	neuropilin 1	-0.651715377	0.000774865
CADM2	cell adhesion molecule 2	-0.653163582	0.053028861
ALMS1	ALMS1 centrosome and basal body associated protein	-0.654462894	0.00062745

FAM124A	family with sequence similarity 124 member A	-0.658623348	0.0000981
CASQ1	calsequestrin 1	-0.66578604	0.008119276
EPPK1	epiplakin 1	-0.66762129	0.039913957
NBPF19	NBPF member 19	-0.667726207	0.021319497
MSH5	mutS homolog 5	-0.670801962	0.081598227
MYO18A	myosin XVIIIA	-0.671673585	0.00000622
B3GALT5	beta-1;3-galactosyltransferase 5	-0.673149215	0.037135406
NR4A3	nuclear receptor subfamily 4 group A member 3	-0.67408733	0.092229013
NOTCH2NLA	notch 2 N-terminal like A	-0.675152562	0.006717914
NBPF14	NBPF member 14	-0.675625372	0.000000795
PDE3A	phosphodiesterase 3A	-0.676279033	0.022362486
WDR62	WD repeat domain 62	-0.67685652	0.05863414
PI15	peptidase inhibitor 15	-0.678934759	1.16E-14
STK17B	serine/threonine kinase 17b	-0.679681539	0.053676927
KANTR	KANTR integral membrane protein	-0.682360809	0.016444093
ADAMTS9	ADAM metallopeptidase with thrombospondin type 1 motif 9	-0.68458394	0.006036323
CBR3	carbonyl reductase 3	-0.686136619	0.057913617
ELF4	E74 like ETS transcription factor 4	-0.687486896	0.009852804
NPIPB5	nuclear pore complex interacting protein family member B5	-0.692848098	0.006705238
MBOAT1	membrane bound O-acyltransferase domain containing 1	-0.694863725	0.047550758
45508	argonaute RISC component 4	-0.69685112	0.097305877
SMG1P3	SMG1 pseudogene 3	-0.700132474	0.002669214
SLC1A2	solute carrier family 1 member 2	-0.700258536	3.01E-13
RALYL	RALY RNA binding protein like	-0.701186108	0.066824481
RFX4	regulatory factor X4	-0.702558787	0.003941679
WLS	Wnt ligand secretion mediator	-0.703125951	0.00000224
SRP14-DT	SRP14 divergent transcript	-0.705126948	0.089702747
KMT2D	lysine methyltransferase 2D	-0.715903195	0.00000197
ARMCX1	armadillo repeat containing X-linked 1	-0.716490465	0.06261563
ANGPTL4	angiopoietin like 4	-0.717648183	0.019689789
NCKAP5	NCK associated protein 5	-0.720071987	0.031442255
KDM7A	lysine demethylase 7A	-0.720752867	0.069680447
TIRAP	TIR domain containing adaptor protein	-0.729268662	0.094237511
CCDC188	coiled-coil domain containing 188	-0.732588717	0.095415138
LRRC15	leucine rich repeat containing 15	-0.739400845	0.001326944
CSPG4	chondroitin sulfate proteoglycan 4	-0.74193952	0.000524831
ADM	adrenomedullin	-0.742244301	0.064078554
TLR4	toll like receptor 4	-0.752568762	0.052630316
S100A4	S100 calcium binding protein A4	-0.752765774	0.015638232

FBN2	fibrillin 2	-0.753706262	9.23E-11
PHF11	PHD finger protein 11	-0.755642602	0.056244152
A2M	alpha-2-macroglobulin	-0.758641734	0.000742319
KCNAB2	potassium voltage-gated channel subfamily A regulatory beta subunit 2	-0.765352144	0.041780508
MIRLET7IHG	MIRLET7I host gene	-0.765795339	0.067793766
HLA-DPB1	major histocompatibility complex; class II; DP beta 1	-0.766263328	0.048040577
GRIN2B	glutamate ionotropic receptor NMDA type subunit 2B	-0.766364027	0.005269991
NBPF10	NBPF member 10	-0.768168791	0.004545368
LY6H	lymphocyte antigen 6 family member H	-0.770130913	0.064285199
PRX	periaxin	-0.772913735	0.086222278
HLA-DMB	major histocompatibility complex; class II; DM beta	-0.776106441	0.001501489
MFAP5	microfibril associated protein 5	-0.77896506	0.001657291
B3GALT1	beta-1;3-galactosyltransferase 1	-0.780740379	0.000314359
TENT5B	terminal nucleotidyltransferase 5B	-0.784096472	3.24E-09
FKBP11	FKBP prolyl isomerase 11	-0.7859221	0.056494146
TRERF1	transcriptional regulating factor 1	-0.787213701	0.064586961
CNTNAP3B	contactin associated protein family member 3B	-0.789741924	0.066365152
DGKH	diacylglycerol kinase eta	-0.790486672	0.002520073
GNAO1	G protein subunit alpha o1	-0.791928831	0.000000157
COCH	cochlin	-0.796918092	0.019234264
SORL1	sortilin related receptor 1	-0.805633636	1.77E-11
SMG1P1	SMG1 pseudogene 1	-0.820518156	0.005952437
NFATC4	nuclear factor of activated T cells 4	-0.820532175	0.027609382
MYBL1	MYB proto-oncogene like 1	-0.821712255	0.010307886
NEK7	NIMA related kinase 7	-0.823987362	0.00932838
CYP1B1	cytochrome P450 family 1 subfamily B member 1	-0.834503813	0.00062229
SEMA6D	semaphorin 6D	-0.84271222	0.000000492
CDH1	cadherin 1	-0.844012744	0.0000233
TBC1D2	TBC1 domain family member 2	-0.847028637	0.002097253
DTX4	deltex E3 ubiquitin ligase 4	-0.861959782	0.000136627
SH3BP1	SH3 domain binding protein 1	-0.862453181	0.005240114
JHY	junctional cadherin complex regulator	-0.862623638	0.036307091
CDH6	cadherin 6	-0.863715368	6.53E-10
SECTM1	secreted and transmembrane 1	-0.865170399	0.062692737
RBPMS2	RNA binding protein; mRNA processing factor 2	-0.865580784	0.00276424
CKLF	chemokine like factor	-0.865825466	0.071250697

ENPP2	ectonucleotide pyrophosphatase/phosphodiesterase 2	-0.874213065	0.002651388
EPAS1	endothelial PAS domain protein 1	-0.875283166	0.053232096
HKDC1	hexokinase domain containing 1	-0.875930769	5.91E-08
FOXJ1	forkhead box J1	-0.87863929	2.32E-09
ATOH8	atonal bHLH transcription factor 8	-0.884584963	0.009125786
C6orf118	chromosome 6 open reading frame 118	-0.88803165	0.001192706
ALPL	alkaline phosphatase; biomineralization associated	-0.888540372	0.006523542
CNR1	cannabinoid receptor 1	-0.903416681	0.026804735
NEK11	NIMA related kinase 11	-0.903658355	0.027321221
LINC01508	long intergenic non-protein coding RNA 1508	-0.903682922	0.072727577
IER3	immediate early response 3	-0.90606437	0.00000043
CARD16	caspase recruitment domain family member 16	-0.911621301	0.002936073
PARVB	parvin beta	-0.911790178	0.006486135
VPS13D	vacuolar protein sorting 13 homolog D	-0.918308352	4.66E-08
CAMK4	calcium/calmodulin dependent protein kinase IV	-0.919088459	0.025246043
B3GALT4	beta-1;3-galactosyltransferase 4	-0.921044644	0.094766195
FBN1	fibrillin 1	-0.924183403	0.000475264
CRISPLD2	cysteine rich secretory protein LCCL domain containing 2	-0.928174087	0.091840003
LOXL2	lysyl oxidase like 2	-0.932754469	2.35E-08
COLEC12	collectin subfamily member 12	-0.934771651	0.034447591
FAM181A	family with sequence similarity 181 member A	-0.937332052	0.00740872
PLAUR	plasminogen activator; urokinase receptor	-0.950779292	0.000000337
DRC1	dynein regulatory complex subunit 1	-0.953965034	0.035124773
PNPLA7	patatin like phospholipase domain containing 7	-0.965547353	0.018996881
LIN9	lin-9 DREAM MuvB core complex component	-0.969921594	0.053976391
SFRP4	secreted frizzled related protein 4	-0.972337664	7.61E-09
FHIP1A	FHF complex subunit HOOK interacting protein 1A	-0.972767728	0.044556992
WNT5B	Wnt family member 5B	-0.974904715	0.004038673
NFRKB	nuclear factor related to kappaB binding protein	-0.976346977	0.083402638
CFAP92	cilia and flagella associated protein 92 (putative)	-0.976831256	0.052531192
TEK	TEK receptor tyrosine kinase	-0.981151628	0.00000112
CD101	CD101 molecule	-0.982862468	0.024155275
FRMD6	FERM domain containing 6	-0.985256785	0.002575884

SLC1A1	solute carrier family 1 member 1	-0.986521051	0.073706741
MICAL2	microtubule associated monooxygenase; calponin and LIM domain containing 2	-0.995669156	0.000207293
XYLT1	xylosyltransferase 1	-1.003256598	3.83E-26
TRPV3	transient receptor potential cation channel subfamily V member 3	-1.003498671	0.004880769
CDC42BPG	CDC42 binding protein kinase gamma	-1.008549206	0.023579307
ADAMTSL1	ADAMTS like 1	-1.012669783	0.003626417
CXCL12	C-X-C motif chemokine ligand 12	-1.015771734	0.000230177
EMILIN1	elastin microfibril interfacer 1	-1.03080843	0.000273155
RGS4	regulator of G protein signaling 4	-1.03771263	0.096582085
ITGB3	integrin subunit beta 3	-1.039720223	0.000140337
SDK2	sidekick cell adhesion molecule 2	-1.045041973	0.000604802
SLC6A16	solute carrier family 6 member 16	-1.046245395	0.058697242
ZNF483	zinc finger protein 483	-1.05255682	0.00000847
G0S2	G0/G1 switch 2	-1.053792612	0.01825753
NPIPB4	nuclear pore complex interacting protein family member B4	-1.057501683	0.0000227
PCDH17	protocadherin 17	-1.058174352	0.087796696
SLFN5	schlafen family member 5	-1.064222049	0.039761973
ARHGEF28	Rho guanine nucleotide exchange factor 28	-1.064403536	0.064884086
TGFBI	transforming growth factor beta induced	-1.071272168	0.000528062
TC2N	tandem C2 domains; nuclear	-1.07218143	0.0000107
SCART1	scavenger receptor family member expressed on T cells 1	-1.074969297	0.099973857
B3GNT9	UDP-GlcNAc:betaGal beta-1;3-N-acetylglucosaminyltransferase 9	-1.075541991	0.003719212
PTGS1	prostaglandin-endoperoxide synthase 1	-1.07727359	0.093376081
KIAA1614	KIAA1614	-1.081619653	0.001479432
TMEM132E	transmembrane protein 132E	-1.090249069	1.97E-08
STC2	stanniocalcin 2	-1.090804312	2.89E-09
PRKN	parkin RBR E3 ubiquitin protein ligase	-1.095681552	0.053028861
AHNAK	AHNAK nucleoprotein	-1.096638901	3.81E-24
FMN1	formin 1	-1.103936678	0.004604597
SYNE2	spectrin repeat containing nuclear envelope protein 2	-1.109001795	0.00000155
RN7SL2	RNA component of signal recognition particle 7SL2	-1.111331952	0.022013074
SIAH3	siah E3 ubiquitin protein ligase family member 3	-1.123718949	0.0000314
ANKRD63	ankyrin repeat domain 63	-1.138235077	0.001935248

IL1R1	interleukin 1 receptor type 1	-1.140915507	0.011528861
FRMD4B	FERM domain containing 4B	-1.141572005	0.015203543
RN7SL1	RNA component of signal recognition particle 7SL1	-1.142208358	0.028082087
ELMOD1	ELMO domain containing 1	-1.143716116	6.05E-11
PCOLCE	procollagen C-endopeptidase enhancer	-1.147964927	0.000812899
SMTN	smoothelin	-1.154683244	0.00000424
TNXB	tenascin XB	-1.176986513	0.013209416
SERPINE1	serpin family E member 1	-1.216138634	3.45E-19
ST3GAL1	ST3 beta-galactoside alpha-2;3-sialyltransferase 1	-1.218003274	0.051869205
CTDSPL	CTD small phosphatase like	-1.218511737	0.011653588
RNF213-AS1	RNF213 antisense RNA 1	-1.219825759	0.05833215
SLC6A6	solute carrier family 6 member 6	-1.222284911	0.019226047
UPK3BL1	uroplakin 3B like 1	-1.226253465	0.021685798
ADAMTSL3	ADAMTS like 3	-1.228382163	0.053252438
PHLDB2	pleckstrin homology like domain family B member 2	-1.229322972	0.00723546
GRAMD1C	GRAM domain containing 1C	-1.229516699	0.051738145
COL5A2	collagen type V alpha 2 chain	-1.237357431	0.0000162
TNFRSF1B	TNF receptor superfamily member 1B	-1.241795697	0.084494074
SCEL	sciellin	-1.263682615	0.067634618
EHBP1L1	EH domain binding protein 1 like 1	-1.264287192	0.0000159
QPRT	quinolinate phosphoribosyltransferase	-1.265639229	0.090130949
RYR2	ryanodine receptor 2	-1.269305517	6.63E-08
IGFBP4	insulin like growth factor binding protein 4	-1.273043101	2.35E-08
SYT14	synaptotagmin 14	-1.277819144	0.002123313
EMP1	epithelial membrane protein 1	-1.292135013	5.58E-12
SLX1B	SLX1 homolog B; structure-specific endonuclease subunit	-1.320871022	0.091178737
MMRN1	multimerin 1	-1.324301493	1.84E-33
HMGA2	high mobility group AT-hook 2	-1.325242681	0.00000036
VIPR1	vasoactive intestinal peptide receptor 1	-1.333684017	0.063073745
CFAP126	cilia and flagella associated protein 126	-1.343876272	0.001265276
FUT9	fucosyltransferase 9	-1.404632628	1.77E-09
NBPF20	NBPF member 20	-1.423803693	2.31E-08
ATP2A1	ATPase sarcoplasmic/endoplasmic reticulum Ca2+ transporting 1	-1.426564487	0.081598227
CAVIN2	caveolae associated protein 2	-1.433269626	0.097902177

CYSRT1	cysteine rich tail 1	-1.452143176	0.023748558
CHRM3	cholinergic receptor muscarinic 3	-1.460631854	0.020206035
RCN3	reticulocalbin 3	-1.467384712	0.013784924
HLA-DQB2	major histocompatibility complex; class II; DQ beta 2	-1.469174217	0.051245481
C2orf50	chromosome 2 open reading frame 50	-1.471951334	0.082944247
GDF10	growth differentiation factor 10	-1.474921354	0.026658392
KCNQ1OT1	KCNQ1 opposite strand/antisense transcript 1	-1.477972801	0.004426217
KCNMB3	potassium calcium-activated channel subfamily M regulatory beta subunit 3	-1.498358563	0.076359793
RGPD8	RANBP2 like and GRIP domain containing 8	-1.52441076	0.001903809
OTULINL	OTU deubiquitinase with linear linkage specificity like	-1.526242717	0.055145908
CCDC80	coiled-coil domain containing 80	-1.535876317	0.0000595
HECW2	HECT; C2 and WW domain containing E3 ubiquitin protein ligase 2	-1.544953475	0.0000302
CHST9	carbohydrate sulfotransferase 9	-1.545402163	0.044391536
MGST1	microsomal glutathione S-transferase 1	-1.545851875	0.000000356
JCAD	junctional cadherin 5 associated	-1.55198521	0.000918945
KLF2	KLF transcription factor 2	-1.556841501	0.08316825
IL7R	interleukin 7 receptor	-1.558585156	0.060698472
SLIT3	slit guidance ligand 3	-1.559182816	0.000000772
CEMIP	cell migration inducing hyaluronidase 1	-1.562799909	0.046591067
CHRFAM7A	CHRNA7 (exons 5-10) and FAM7A (exons A-E) fusion	-1.574484796	0.021970411
NWD1	NACHT and WD repeat domain containing 1	-1.576797682	0.026506123
GALNT5	polypeptide N-acetylgalactosaminyltransferase 5	-1.580845345	1.41E-21
SEMA7A	semaphorin 7A (John Milton Hagen blood group)	-1.624558678	0.001364956
MOK	MOK protein kinase	-1.662354055	1.32E-09
ESRG	embryonic stem cell related	-1.675426001	0.001747672
CHODL	chondrolectin	-1.703454781	0.013753546
ALDH1L2	aldehyde dehydrogenase 1 family member L2	-1.704259489	0.086222278
FLG	filaggrin	-1.731981463	0.000000314
ABCA13	ATP binding cassette subfamily A member 13	-1.733754833	0.020855861
TFPI	tissue factor pathway inhibitor	-1.736827038	0.00000452
CLDN11	claudin 11	-1.737855565	0.00157883
MICB	MHC class I polypeptide-related sequence B	-1.738454447	0.001241091

DSP	desmoplakin	-1.745760702	1.48E-09
MT2A	metallothionein 2A	-1.746901333	7.52E-24
APOBEC3F	apolipoprotein B mRNA editing enzyme catalytic subunit 3F	-1.748014775	0.044370854
TRIM38	tripartite motif containing 38	-1.762682068	0.076538238
SCAND3	SCAN domain containing 3	-1.775415294	0.000338554
FN1	fibronectin 1	-1.811083832	4.7E-22
KRT18	keratin 18	-1.818179676	1.24E-13
FLNC	filamin C	-1.829515527	7.57E-37
AHRR	aryl hydrocarbon receptor repressor	-1.851671178	0.002713187
OXTR	oxytocin receptor	-1.854499086	0.000115772
SOD3	superoxide dismutase 3	-1.870641624	0.092315919
VWF	von Willebrand factor	-1.890586916	0.034726991
TGIF2-RAB5IF	TGIF2-RAB5IF readthrough	-1.923662375	0.006117209
MYL9	myosin light chain 9	-1.941612064	1.08E-09
TMEM200B	transmembrane protein 200B	-1.946995983	0.09112746
ALDH3A1	aldehyde dehydrogenase 3 family member A1	-1.952094737	0.000000549
FTCD	formimidoyltransferase cyclodeaminase	-1.966929791	0.069191344
SKAP2	src kinase associated phosphoprotein 2	-1.999524483	0.096992126
TAS2R4	taste 2 receptor member 4	-2.002755992	0.056614014
BICC1	BicC family RNA binding protein 1	-2.006632106	3.24E-09
H1-0	H1,0 linker histone	-2.025705185	2.92E-25
GOLGA8N	golgin A8 family member N	-2.037174769	0.008766612
DNER	delta/notch like EGF repeat containing	-2.040894612	0.0000102
ADAMTS6	ADAM metallopeptidase with thrombospondin type 1 motif 6	-2.06203454	0.000103233
CPZ	carboxypeptidase Z	-2.064227388	7.39E-11
HMOX1	heme oxygenase 1	-2.099910891	0.000916341
CD68	CD68 molecule	-2.17864244	0.041698271
MYRF	myelin regulatory factor	-2.190456955	0.009425836
BNC1	basonuclin zinc finger protein 1	-2.196908222	0.001049223
TOR4A	torsin family 4 member A	-2.245826589	0.000634039
CLMP	CXADR like membrane protein	-2.295869256	0.00236188
HMSD	histocompatibility minor serpin domain containing	-2.303758821	0.039509271
GYPC	glycophorin C (Gerbich blood group)	-2.316719086	0.092229013
NEAT1	nuclear paraspeckle assembly transcript 1	-2.333603449	1.27E-09
MRGPRF	MAS related GPR family member F	-2.377016154	2.31E-08
FGF5	fibroblast growth factor 5	-2.380211379	0.000505984
DOCK10	dedicator of cytokinesis 10	-2.417925884	0.016055076

SH2D4A	SH2 domain containing 4A	-2.420595211	0.000793411
FAT4	FAT atypical cadherin 4	-2.470070612	0.07677989
CDCP1	CUB domain containing protein 1	-2.494503215	0.016055076
GATA2	GATA binding protein 2	-2.495369957	0.000025
COL1A2	collagen type I alpha 2 chain	-2.552859642	2.73E-08
GPNMB	glycoprotein nmb	-2.573267929	0.091770031
ETS1	ETS proto-oncogene 1; transcription factor	-2.587820456	2.86E-15
ADORA2A	adenosine A2a receptor	-2.588559056	0.006539747
COL6A2	collagen type VI alpha 2 chain	-2.594045668	1.5E-44
CASP4	caspase 4	-2.61806449	0.044751041
TBX15	T-box transcription factor 15	-2.685401464	0.033624491
TSHZ2	teashirt zinc finger homeobox 2	-2.711447802	0.048697434
PDZD2	PDZ domain containing 2	-2.722599706	0.002123313
COPZ2	COPI coat complex subunit zeta 2	-2.741771812	0.091331616
EMILIN2	elastin microfibril interfacer 2	-2.745101429	0.029570049
DUXAP10	double homeobox A pseudogene 10	-2.751779074	0.01842632
GREM1	gremlin 1; DAN family BMP antagonist	-2.75840016	2.16E-82
PRSS12	serine protease 12	-2.813766276	9.64E-25
PAPPA	pappalysin 1	-2.843959135	0.000000746
STING1	stimulator of interferon response cGAMP interactor 1	-3.016464224	0.074451115
ECM1	extracellular matrix protein 1	-3.029417202	3.58E-13
ANPEP	alanyl aminopeptidase; membrane	-3.049404353	8E-10
AOX1	aldehyde oxidase 1	-3.073419765	0.009508937
CAPG	capping actin protein; gelsolin like	-3.096721111	0.00000938
ROR2	receptor tyrosine kinase like orphan receptor 2	-3.228858495	0.002097253
STAT6	signal transducer and activator of transcription 6	-3.247810765	0.0029331
TGM2	transglutaminase 2	-3.256977592	0.00113779
HTATIP2	HIV-1 Tat interactive protein 2	-3.292261013	0.06261563
KRT19	keratin 19	-3.547791064	0.087660838
MYPN	myopalladin	-3.609321869	0.000017
PEAR1	platelet endothelial aggregation receptor 1	-3.618954118	0.009645042
FAP	fibroblast activation protein alpha	-3.660768447	0.004208243
RAC2	Rac family small GTPase 2	-3.712979983	0.008921334
WNT5A	Wnt family member 5A	-3.81293252	1.01E-15
BGN	biglycan	-3.830238995	0.0000129
FOXD1	forkhead box D1	-3.901180847	0.00000234
ABI3BP	ABI family member 3 binding protein	-3.960684228	0.004604597
TRPV2	transient receptor potential cation channel subfamily V member 2	-3.983577173	0.009645042
RAB3B	RAB3B; member RAS oncogene family	-4.016217727	2.79E-18

SLC16A3	solute carrier family 16 member 3	-4.079058292	4.49E-17
CYBA	cytochrome b-245 alpha chain	-4.128483548	0.0000756
COL12A1	collagen type XII alpha 1 chain	-4.235967324	1.25E-14
MRS2	magnesium transporter MRS2	-4.279335497	0.010526444
MUC16	mucin 16; cell surface associated	-4.422210842	0.010307886
LTBR	lymphotoxin beta receptor	-4.759844613	0.00000031
CFH	complement factor H	-4.762719415	0.06818824
CLEC14A	C-type lectin domain containing 14A	-4.788844963	0.016269463
HTR1F	5-hydroxytryptamine receptor 1F	-4.858420823	0.027179161
PID1	phosphotyrosine interaction domain containing 1	-4.86611875	0.005835084
FAM167A	family with sequence similarity 167 member A	-4.870659493	2.69E-08
GPRC5A	G protein-coupled receptor class C group 5 member A	-4.902135389	0.00000337
DUXAP8	double homeobox A pseudogene 8	-4.939849004	0.025366798
LY6K	lymphocyte antigen 6 family member K	-5.095392321	1.98E-08
ABLIM3	actin binding LIM protein family member 3	-5.162670453	0.0000487
FLI1	Fli-1 proto-oncogene; ETS transcription factor	-5.234894166	0.035737896
SLC43A3	solute carrier family 43 member 3	-5.29098937	5.37E-10
WNK4	WNK lysine deficient protein kinase 4	-5.312923467	0.00000235
PSG4	pregnancy specific beta-1-glycoprotein 4	-5.371007298	0.028139488
INHBA	inhibin subunit beta A	-5.4893178	0.004382516
QNG1	Q-nucleotide N-glycosylase 1	-5.497478503	0.011510876
PARP8	poly(ADP-ribose) polymerase family member 8	-5.557602878	0.011620821
CCBE1	collagen and calcium binding EGF domains 1	-5.565664608	8.05E-08
VEGFC	vascular endothelial growth factor C	-5.740091558	0.00000284
TFAP2A	transcription factor AP-2 alpha	-5.798390955	0.00049777
CGAS	cyclic GMP-AMP synthase	-5.860882335	0.007837524
COL3A1	collagen type III alpha 1 chain	-5.897669134	0.084863115
IGF2	insulin like growth factor 2	-6.044099843	0.0000147
KRTAP2-3	keratin associated protein 2-3	-6.226157563	0.027032163
ENG	endoglin	-6.226645889	4.2E-27
CDK15	cyclin dependent kinase 15	-6.37357148	0.000337285
MSC-AS1	MSC antisense RNA 1	-6.486610862	0.084591143
FOXF1	forkhead box F1	-6.495871885	0.08102998
PRRX2	paired related homeobox 2	-6.540123899	0.0000211
CCN4	cellular communication network factor 4	-6.560921769	0.067038656
IL1B	interleukin 1 beta	-6.577482349	0.000574537

CDH13	cadherin 13	-6.652764857	1.32E-11
LINC01116	long intergenic non-protein coding RNA 1116	-6.722627395	0.039101569
KRT34	keratin 34	-6.7260602	0.00068118
HOXA9	homeobox A9	-6.956127062	0.014496833
EBF3	EBF transcription factor 3	-6.984012611	0.012709665
VRK2	VRK serine/threonine kinase 2	-6.992705716	0.003194051
SLC17A9	solute carrier family 17 member 9	-7.186400937	0.0000123
MT1E	metallothionein 1E	-7.277079059	0.000524711
FOXC2	forkhead box C2	-8.073048539	0.000000324
GPAT2	glycerol-3-phosphate acyltransferase 2; mitochondrial	-8.51865072	1.01E-08
SERPINB2	serpin family B member 2	-8.579301257	2.89E-09
MLPH	melanophilin	-8.827724115	9.23E-11
FXYD5	FXYD domain containing ion transport regulator 5	-8.876023692	1.57E-17
TBX3	T-box transcription factor 3	-8.938560939	2.95E-11
NR2F2	nuclear receptor subfamily 2 group F member 2	-9.838572475	0.00085242
ADAMTS1	ADAM metallopeptidase with thrombospondin type 1 motif 1	-9.841071533	2.79E-17
DKK1	dickkopf WNT signaling pathway inhibitor 1	-9.932861223	3.68E-21
SRGN	serglycin	-10.16645497	1.02E-20
COL6A3	collagen type VI alpha 3 chain	-10.5433185	1.28E-33
UTP14C	UTP14C small subunit processome component	-29.98963013	2E-11

Supplementary Table 18: Ingenuity pathway analysis of DEGs in Acetate vs Control condition (p<0.05, z-score>|2|)

Ingenuity Canonical Pathways	-log(p-value)	Ratio	z-score	Molecules
Elastic fibre formation	9,23E00	2,79E-01	-1,732	BMP2,EMILIN1,EMILIN2,FBN1,FBN2,FBN3,FN1,ITGA5,ITGB3,LOXL2,MFAP4,MFAP5
Integrin cell surface interactions	8,58E00	1,83E-01	-3,357	CDH1,COL13A1,COL1A2,COL3A1,COL5A1,COL5A2,COL6A2,COL6A3,FBN1,FN1,ITGA10,ITGA5,ITGB3,VCAM1,VWF
Assembly of collagen fibrils and (	6,54E00	1,86E-01	-2,714	COL12A1,COL14A1,COL1A2,COL3A1,COL5A1,COL5A2,COL6A2,COL6A3,DST,LOXL2,PCOLCE
Collagen biosynthesis and modif	5,96E00	1,64E-01	-2,714	ADAMTS2,COL12A1,COL13A1,COL14A1,COL1A2,COL3A1,COL5A1,COL5A2,COL6A2,COL6A3,PCOLCE
Osteoarthritis Pathway	5,9E00	9,09E-02	-2,183	ALPL,BMP2,CASP10,CASP4,CASQ1,CCN4,DKK1,DLX5,EPAS1,FN1,GREM1,IL1B,IL1R1,ITGA10,ITGA5,ITGB3,SDC4,TLR4,TNFRSF1B,VEGFC
Collagen chain trimerization	5,81E00	2,05E-01	-2,333	COL12A1,COL13A1,COL14A1,COL1A2,COL3A1,COL5A1,COL5A2,COL6A2,COL6A3
Syndecan interactions	5,37E00	2,59E-01	-2,646	COL1A2,COL3A1,COL5A1,COL5A2,FN1,ITGB3,SDC4

Collagen degradation	4,9E00	1,61E-01	-2,333	COL12A1,COL13A1,COL14A1,COL1A2,COL3A1,COL5A1,COL5A2,COL6A2,COL6A3
Extracellular matrix organization	4,75E00	1,14E-01	-3,464	BGN,COL1A2,COL3A1,COL5A1,COL5A2,COL6A2,COL6A3,FN1,ITGA5,ITGB3,SERPINE1,TNXB
Response to elevated platelet cy	4,65E00	1,04E-01	-2,496	A2M,ECM1,FN1,IGF2,ITGB3,LEFTY2,MMRN1,SERPINA3,SERPINE1,SRGN,TOR4A,VEGFC,VWF
RHOGDI Signaling	4,37E00	7,98E-02	0,632	ACTA1,ACTG2,ARHGEF3,CDH1,CDH13,CDH6,DLC1,GNAO1,GNG2,GNG4,ITGA10,ITGA5,ITGB3,MYL9,MYO18A,PAK3,RAC2
Pathogen Induced Cytokine Stor	4,32E00	6,91E-02	-3,710	CCR1,CDH1,COL12A1,COL13A1,COL1A2,COL3A1,COL5A1,COL5A2,COL6A2,COL6A3,CXCL12,FTH1,HLA-DMB,IL1B,IL1R1,PRDM1,RYR2,SRGN,TLR4,TNFRSF1B,VEGFC
Pulmonary Fibrosis Idiopathic Si	4,13E00	6,71E-02	-3,300	ACTA1,ACTG2,CCN4,CDH1,COL12A1,COL13A1,COL1A2,COL3A1,COL5A1,COL5A2,COL6A2,COL6A3,CXCL12,EPHB3,FN1,IL11RA,IL1B,SERPINE1,STAT6,WNT5A,WNT5B
Cell junction organization	3,95E00	1,11E-01	-3,162	CADM2,CDH1,CDH13,CDH6,CLDN11,DST,FLNC,PARVB,SDK1,SDK2

Role of Osteoclasts in Rheumato	3,9E00	6,83E-02	-2,982	CDH1,COL12A1,COL13A1,COL1A2,COL3A1,COL5A1,COL5A2,COL6A2,COL6A3,IL1B,IL1R1,ITGA5,ITGB3,RAC2,SFRP4,SHC3,TLR4,TNFRSF1B,VAV3
Coagulation System	3,82E00	1,88E-01	0,816	A2M,F3,PLAUR,SERPINE1,TFPI,VWF
ILK Signaling	3,3E00	7,29E-02	-0,632	ACTA1,ACTG2,BMP2,CDH1,DSP,FLNC,FN1,ITGB3,KRT18,MYL9,MYO18A,PARVB,RAC2,VEGFC
Regulation of Insulin-like Growth	3,08E00	8,7E-02	-1,265	AMBN,C4A/C4B,FBN1,FN1,IGF2,IGFBP4,IGFBP5,PAPPA,STC2,VWA1
Degradation of the extracellular n	3,05E00	1,17E-01	-1,890	A2M,ADAMTS9,CDH1,FBN1,FBN2,FBN3,FN1
GABAergic Receptor Signaling F	3,05E00	8,03E-02	0,302	ADCY7,CACNG7,GABRE,GABRQ,GNAO1,GNG2,GNG4,GRIN2D,ITPR3,NRXN3,SLC12A5
GP6 Signaling Pathway	3	8,47E-02	-3,000	COL12A1,COL13A1,COL1A2,COL3A1,COL5A1,COL5A2,COL6A2,COL6A3,ITGB3,VAV3
Semaphorin interactions	2,88E00	1,09E-01	-1,134	CRMP1,MYL9,NRP1,PAK3,SEMA5A,SEMA6D,SEMA7A
Beta-catenin independent WNT :	2,87E00	8,82E-02	-1,000	GNAO1,GNG2,GNG4,ITPR3,RAC2,ROR1,ROR2,WNT5A,WNT5B
TEC Kinase Signaling	2,75E00	6,67E-02	0,378	ACTA1,ACTG2,GNAO1,GNG2,GNG4,ITGA10,ITGA5,ITGB3,PAK3,RAC2,STAT6,TLR4,VAV3

O-linked glycosylation	2,73E00	8,41E-02	-3,000	ADAMTS2,ADAMTS6,ADAMTS9,ADAMTSL1,B3GNT9,GALNT5,GALNT6,SEMA5A,ST3GAL1
Wound Healing Signaling Pathw	2,73E00	6,36E-02	-3,742	COL12A1,COL13A1,COL1A2,COL3A1,COL5A1,COL5A2,COL6A2,COL6A3,FN1,IL1B,IL1R1,SHC3,TNFRSF1B,VEGFC
Agrin Interactions at Neuromusc	2,73E00	1,03E-01	0,447	ACTA1,ACTG2,CHRNA1,ERBB4,PAK3,RAC2,UTRN
Ephrin B Signaling	2,58E00	9,72E-02	-0,447	CXCL12,EPHB3,GNAO1,GNG2,GNG4,RAC2,VAV3
Signaling by Rho Family GTPas	2,46E00	5,75E-02	-0,333	ACTA1,ACTG2,ARHGEF3,CDH1,CDH13,CDH6,GNAO1,GNG2,GNG4,ITGA10,ITGA5,ITGB3,MYL9,PAK3,RAC2
Semaphorin Neuronal Repulsive	2,42E00	7,09E-02	-1,000	CRMP1,CSPG4,ITGA10,ITGA5,ITGB3,MYL9,NRP1,PAK3,SEMA5A,SEMA6D
Signaling by PDGF	2,42E00	1,03E-01	-2,449	COL3A1,COL5A1,COL5A2,COL6A2,COL6A3,STAT6
Post-translational protein phosph	2,38E00	8,08E-02	-0,707	AMBN,C4A/C4B,FBN1,FN1,IGFBP4,IGFBP5,STC2,VWA1
Tumor Microenvironment Pathwa	2,35E00	6,55E-02	-3,162	COL1A2,COL3A1,CSPG4,CXCL12,FGF5,FN1,IGF2,IL1B,ITGA5,ITGB3,VEGFC
Dilated Cardiomyopathy Signalin	2,32E00	6,85E-02	1,342	ACTA1,ACTG2,ADCY7,CACNG7,ITPR3,MYL9,MYO18A,RYR2,TNNT1,UTRN

Acute Phase Response Signaling	2,29E00	6,43E-02	-1,890	A2M,C4A/C4B,FN1,HMOX1,IL1B,IL1R1,SERPINA3,SERPINE1,SERPINF1,TNFRSF1B,VWF
GPER1 signaling	2,27E00	1,14E-01	0,447	ADCY7,FN1,GNG2,GNG4,ITGA5
Regulation of Actin-based Motility	2,2E00	7,55E-02	0,000	ACTA1,ACTG2,ITGA10,ITGA5,ITGB3,MYL9,PAK3,RAC2
Ephrin Receptor Signaling	2,2E00	5,97E-02	-0,707	CXCL12,EPHB3,GNAO1,GNG2,GNG4,GRIN2D,ITGA10,ITGA5,ITGB3,PAK3,RAC2,VEGFC
G alpha (z) signalling events	2,1E00	1,04E-01	1,342	ADCY7,GNG2,GNG4,RGS16,RGS4
Neuroinflammation Signaling Pa	2,07E00	5,21E-02	-1,897	BDNF,CXCL12,GABRE,GABRQ,GRIN2D,HLA-DMB,HMOX1,IDE,IL1B,IL1R1,PSEN2,RAC2,SLC1A2,TLR4,VCAM1
Formation of Fibrin Clot (Clotting	2,04E00	1,25E-01	-2,000	A2M,F3,TFPI,VWF
IL-4 Signaling	2,04E00	5,95E-02	-3,317	COL12A1,COL13A1,COL1A2,COL3A1,COL5A1,COL5A2,COL6A2,COL6A3,HLA-DMB,STAT6,TGM2
Hepatic Fibrosis Signaling Pathw	2,04E00	4,73E-02	-3,638	CACNG7,CNR1,COL1A2,COL3A1,FTH1,IL1B,IL1R1,ITGA10,ITGA5,ITGB3,MYL9,RAC2,SERPINE1,TLR4,TNFRSF1B,VCAM1,VEGFC,WNT5A,WNT5B
Acetylcholine Receptor Signaling	2,03E00	5,91E-02	0,302	ADCY7,CACNG7,CASP10,CASP4,CASQ1,CHRFAM7A,CHRNA1,GNAO1,HMOX1,ITPR3,PSEN2

EPH-Ephrin signaling	2	7,61E-02	0,378	ARHGEF28,CLTCL1,EPHB3,MYL9,PAK3,PSEN2,VAV3
Oxytocin in Brain Signaling Path	1,98E00	5,82E-02	0,905	CACNG7,GNAO1,GNG2,GNG4,IL1B,ITPR3,NLRP1,OXTR,PTGS1,SLC12A5,TLR4
Xenobiotic Metabolism AHR Sig	1,96E00	8,33E-02	-1,633	AHRR,ALDH3A1,CYP1B1,IL1B,MGST1,NQO1
G alpha (i) signalling events	1,94E00	5,33E-02	-0,277	ADCY7,CCR1,CNR1,CXCL12,GNG2,GNG4,HTR1F,RGR,RGS16,RGS4,RGS8,TAS2R4,TAS2R5
Neutrophil Extracellular Trap Sig	1,93E00	4,79E-02	1,213	CASP10,CASP4,CASQ1,CCR1,COL12A1,COL13A1,COL1A2,COL3A1,COL5A1,COL5A2,COL6A2,COL6A3,IL1B,ITPR3,MT-ND3,RAC2,TLR4
Opioid Signaling Pathway	1,93E00	5,15E-02	-0,577	ADCY7,CACNG7,CLTCL1,GNAO1,GNG2,GNG4,GRIN2D,ITPR3,RAC2,RGS16,RGS4,RGS8,RYR2,SLC12A5
IL-10 Signaling	1,91E00	6,29E-02	1,000	BMP2,CCN4,CCR1,HLA-DMB,HMOX1,IL1B,IL1R1,PRDM1,TLR4
Activin Inhibin Signaling Pathwa	1,88E00	5,64E-02	-1,508	CDH1,COL1A2,COL3A1,GATA2,IL1B,IL1R1,INHBA,PMEPA1,SERPINE1,TLR4,TNFRSF1B
Synaptogenesis Signaling Pathw	1,87E00	4,93E-02	0,258	ADCY7,BDNF,CDH1,CDH13,CDH6,CPLX1,CPLX2,EPHB3,GRIN2D,LRRTM2,NRXN3,NTRK2,SHC3,STXBP6,SYT14

Huntington's Disease Signaling	1,86E00	5,05E-02	1,342	BDNF,CASP10,CASP4,CASQ1,CLTCL1,CPLX2,DNM3,GNG2,GNG4,HSPA1A/HSPA1B,POLR2J2/POLR2J3,RCOR2,SHC3,TGM2
Endocannabinoid Neuronal Syna	1,85E00	6,16E-02	-0,816	ADCY7,CACNG7,CNR1,DNAH1,GNAO1,GNG2,GNG4,GRIN2D,ITPR3
Neurexins and neuroligins	1,8E00	8,77E-02	0,447	DLGAP1,DLGAP2,GRIN2D,LRRTM2,NRXN3
Crosstalk between Dendritic Cell	1,78E00	7,59E-02	-2,000	ACTA1,ACTG2,LTBR,MICB,TLR4,TNFRSF1B
G alpha (12/13) signalling events	1,75E00	7,5E-02	0,816	ARHGEF3,GNG2,GNG4,OBSCN,PREX1,VAV3
IL-8 Signaling	1,74E00	5,37E-02	-1,414	CDH1,GNAO1,GNG2,GNG4,HMOX1,ITGB3,MYL9,RAC2,TEK,VCAM1,VEGFC
Calcium Signaling	1,74E00	5,37E-02	-0,378	ACTA1,CACNG7,CASQ1,CHRFAM7A,CHRNA1,GRIN2D,ITPR3,MYL9,MYO18A,RYR2,TNNT1
IL-17A Signaling in Fibroblasts	1,73E00	7,41E-02	-1,633	COL1A2,COL3A1,CXCL12,FN1,IL1B,VCAM1
Actin Cytoskeleton Signaling	1,72E00	5,15E-02	0,000	ACTA1,ACTG2,FGF5,FN1,ITGA10,ITGA5,ITGB3,MYL9,MYO18A,PAK3,RAC2,VAV3
NCAM signaling for neurite out-g	1,71E00	8,33E-02	-2,236	COL3A1,COL5A1,COL5A2,COL6A2,COL6A3
Interleukin-4 and Interleukin-13 s	1,69E00	6,6E-02	-1,134	COL1A2,FN1,HMOX1,IL1B,STAT6,TNFRSF1B,VCAM1
Vasopressin regulates renal wate	1,67E00	9,76E-02	1,000	ADCY7,AQP4,GNG2,GNG4

Signaling by VEGF	1,67E00	6,54E-02	-0,816	CYBA,ITGB3,ITPR3,NRP1,PAK3,VAV3,VEGFC
Role of Osteoblasts in Rheumato	1,65E00	5,21E-02	-1,508	ALPL,BMP2,CXCL12,DKK1,DLX5,IL1B,SFRP4,STAT6,VEGFC,WNT5A,WNT5B
ABRA Signaling Pathway	1,64E00	7,06E-02	-2,449	ABLIM3,ACTA1,ACTG2,MYL9,SMTN,VEGFC
Pyroptosis Signaling Pathway	1,59E00	6,9E-02	-2,449	CASP4,IL1B,IL1R1,NLRP1,TLR4,TNFRSF1B
CXCR4 Signaling	1,58E00	5,52E-02	-0,816	ADCY7,CXCL12,GNAO1,GNG2,GNG4,ITPR3,MYL9,PAK3,RAC2
Interleukin-10 signaling	1,57E00	9,09E-02	-2,000	CCR1,IL1B,IL1R1,TNFRSF1B
Opioid Signalling	1,55E00	6,74E-02	0,447	ADCY7,GNG2,GNG4,ITPR3,NBEA,PPP1R1B
Gαi Signaling	1,54E00	5,76E-02	-0,447	ADCY7,CNR1,GNAO1,GNG2,GNG4,HTR1F,RGS4,SHC3
S100 Family Signaling Pathway	1,54E00	3,83E-02	-2,117	ADGRA1,AHNAK,BDNF,CACNG7,CCR1,CDH1,CNR1,DLC1,ETV4,GPR135,GPRC5A,HTR1F,IL1B,ITPR3,MC1R,NTRK2,OXTR,P2RY6,RAC2,RGR,RYR2,SERPINF1,TLR4,VCAM1,VEGFC,WNT5A,WNT5B
Binding and Uptake of Ligands b	1,54E00	8,89E-02	-1,000	COL1A2,COL3A1,FTH1,MASP1
Cell surface interactions at the va	1,51E00	5,67E-02	-2,828	COL1A2,FN1,ITGA5,ITGB3,PSG4,SDC4,SLC16A3,TEK
Endocannabinoid Cancer Inhibiti	1,48E00	5,59E-02	-2,121	ADCY7,CASP10,CASP4,CASQ1,CDH1,CNR1,GNAO1,VEGFC

ID1 Signaling Pathway	1,48E00	5,08E-02	0,000	BMP2,CHRFAM7A,CHRNA1,ETS1,FN1,IGF2,TFAP2A,TGM2,TNFRSF1B,VEGFC
Molecular Mechanisms of Cance	1,48E00	3,69E-02	-1,095	ADCY7,ADGRA1,ARHGEF3,BMP2,CASP10,CCR1,CDH1,CNR1,GNAO1,GNG2,GNG4,GPR135,GPRC5A,HTR1F,IL11RA,IL1B,IL1R1,IL7R,ITGA10,ITGA5,ITGB3,MC1R,OXTR,P2RY6,PAK3,PSEN2,RAC2,RGR,SHC3,WNT5A
Phospholipase C Signaling	1,47E00	4,59E-02	0,000	ADCY7,AHNAK,ARHGEF3,GNG2,GNG4,HMOX1,ITGA10,ITGA5,ITGB3,ITPR3,MYL9,RAC2,TGM2
L1CAM interactions	1,45E00	5,88E-02	-0,378	ANK3,CD24,DNM3,ITGA10,ITGA5,ITGB3,NRP1
TR/RXR Activation	1,42E00	5,79E-02	-2,646	COL6A3,ITGA5,ITGB3,RAB3B,SLC16A3,THRB,VEGFC
Serotonin Receptor Signaling	1,4E00	4,08E-02	-1,414	ADCY7,BDNF,CACNG7,CDH1,GNAO1,GNG2,GNG4,HMOX1,IL1B,ITGB3,KCNN4,PAK3,PLAUR,PTGS1,RAC2,RYR2,TGM2,VWF
Gαs Signaling	1,38E00	5,69E-02	-0,447	ADCY7,CNR1,GNAO1,GNG2,GNG4,MC1R,RYR2

FAK Signaling	1,38E00	3,79E-02	-2,858	ADGRA1,CCR1,CDH1,CNR1,COL1A2,COL3A1,ECM1,ETS1,ETS2,ETV4,GPR135,GPRC5A,HTR1F,IL11RA,IL1R1,IL7R,ITGA10,ITGA5,ITGB3,MC1R,OXTR,P2RY6,PAK3,RGR
Leukocyte Extravasation Signalir	1,36E00	5,03E-02	0,707	ACTA1,ACTG2,CLDN11,CXCL12,CYBA,DLC1,RAC2,VAV3,VCAM1
Apoptotic execution phase	1,34E00	7,69E-02	-1,000	CDH1,DSP,H1-0,PKP1
Docosahexaenoic Acid (DHA) Si	1,34E00	4,64E-02	-0,905	ADCY7,BDNF,HMOX1,IL1B,ITPR3,NTRK2,PSEN2,SERPINF1,STXBP6,SYT14,TNFRSF1B
SNARE Signaling Pathway	1,34E00	5,56E-02	-0,378	ADCY7,CPLX1,CPLX2,MYL9,MYO18A,STXBP6,SYT14
Role of Chondrocytes in Rheuma	1,32E00	5,51E-02	-2,646	CXCL12,FN1,IL1B,IL1R1,ITGA5,TNFRSF1B,VEGFC
Endothelin-1 Signaling	1,3E00	4,92E-02	-3,000	ADCY7,CASP10,CASP4,CASQ1,GNAO1,HMOX1,ITPR3,PTGS1,SHC3

Supplementary Table 19: Ingenuity pathway analysis of DEGs in Propionate vs Control condition (p<0.05, z-score>|2|)

Ingenuity Canonical Pathways	-log(p-value)	Ratio	z-score	Molecules
Assembly of collagen fibrils and oth	7,55E00	1,86E-01	-2,111	COL12A1,COL14A1,COL1A2,COL3A1,COL5A1,COL5A2,COL6A2,COL6A3,LOXL2,PCOLCE,PXDN
Collagen biosynthesis and modifyin	6,96E00	1,64E-01	-2,111	ADAMTS2,COL12A1,COL14A1,COL1A2,COL25A1,COL3A1,COL5A1,COL5A2,COL6A2,COL6A3,PCOLCE
Elastic fibre formation	6,74E00	2,09E-01	-2,333	EMILIN1,EMILIN2,FBN2,FN1,ITGA5,ITGB3,LOXL2,MFAP4,MFAP5
Collagen chain trimerization	6,65E00	2,05E-01	-1,667	COL12A1,COL14A1,COL1A2,COL25A1,COL3A1,COL5A1,COL5A2,COL6A2,COL6A3
Syndecan interactions	6,04E00	2,59E-01	-2,646	COL1A2,COL3A1,COL5A1,COL5A2,FN1,ITGB3,SDC4
Extracellular matrix organization	5,77E00	1,14E-01	-2,887	BCAN,BGN,COL1A2,COL3A1,COL5A1,COL5A2,COL6A2,COL6A3,FN1,ITGA5,ITGB3,SERPINE1
Collagen degradation	5,71E00	1,61E-01	-1,667	COL12A1,COL14A1,COL1A2,COL25A1,COL3A1,COL5A1,COL5A2,COL6A2,COL6A3
GP6 Signaling Pathway	5,24E00	1,02E-01	-2,111	APBB1IP,COL12A1,COL1A2,COL25A1,COL3A1,COL5A1,COL5A2,COL6A2,COL6A3,ITGB3,LYN,VAV3
Integrin cell surface interactions	5,16E00	1,22E-01	-3,162	COL1A2,COL3A1,COL5A1,COL5A2,COL6A2,COL6A3,FN1,ITGA5,ITGB3,VWF

Pulmonary Fibrosis Idiopathic Signa	5,08E00	6,39E-02	-2,828	ACTG2,ACVR2A,ADAMTS1,AXL,CCN4,COL12A1,COL1A2,COL25A1,COL3A1,COL5A1,COL5A2,COL6A2,COL6A3,CXCL12,FN1,IL1B,SERPINE1,STAT6,WNT5A,WNT5B
Osteoarthritis Pathway	4,86E00	7,27E-02	-2,138	ALPL,CASP10,CASQ1,CCN4,DKK1,DLX5,EPAS1,FN1,GREM1,IL1B,IL1R1,ITGA5,ITGB3,SDC4,TLR4,VEGFC
Coagulation System	4,38E00	1,88E-01	0,816	A2M,F3,PLAUR,SERPINE1,TFPI,VWF
Response to elevated platelet cytos	4,26E00	8,8E-02	-2,714	A2M,ECM1,FN1,IGF2,ITGB3,MMRN1,SERPINA3,SERPINE1,TOR4A,VEGFC,VWF
Role of Osteoclasts in Rheumatoid A	4,17E00	6,12E-02	-2,500	ADAM8,COL12A1,COL1A2,COL25A1,COL3A1,COL5A1,COL5A2,COL6A2,COL6A3,IL1B,IL1R1,ITGA5,ITGB3,RAC2,SFRP4,TLR4,VAV3
Degradation of the extracellular mat	3,65E00	1,17E-01	-1,890	A2M,ADAM8,ADAMTS1,ADAMTS9,BCAN,FBN2,FN1
Semaphorin interactions	3,48E00	1,09E-01	-0,378	CRMP1,MYH14,MYL9,NRP1,PAK3,SEMA6D,SEMA7A
Pathogen Induced Cytokine Storm S	3,23E00	5,26E-02	-3,000	COL12A1,COL1A2,COL25A1,COL3A1,COL5A1,COL5A2,COL6A2,COL6A3,CXCL12,GSDMD,IL1B,IL1R1,PRDM1,RYR2,TLR4,VEGFC
Wound Healing Signaling Pathway	3,18E00	5,91E-02	-2,496	ACVR2A,COL12A1,COL1A2,COL25A1,COL3A1,COL5A1,COL5A2,COL6A2,COL6A3,FN1,IL1B,IL1R1,VEGFC

Xenobiotic Metabolism AHR Signali	3,16E00	9,72E-02	-1,890	AHRR,ALDH1A2,ALDH1L2,ALDH3A1,CYP1B1,IL1B,MGST1
Tumor Microenvironment Pathway	3,13E00	6,55E-02	-3,162	COL1A2,COL3A1,CSPG4,CXCL12,FGF5,FN1,IGF2,IL1B,ITGA5,ITGB3,VEGFC
Signaling by PDGF	2,92E00	1,03E-01	-2,449	COL3A1,COL5A1,COL5A2,COL6A2,COL6A3,STAT6
Glycosaminoglycan metabolism	2,92E00	8,86E-02	-1,890	B3GAT2,BCAN,BGN,CSPG4,PAPSS2,SDC4,ST3GAL1
Integrin signaling	2,87E00	1,67E-01	-2,000	APBB1IP,FN1,ITGB3,VWF
NCAM signaling for neurite out-grov	2,84E00	0.1	-2,449	CACNA1C,COL3A1,COL5A1,COL5A2,COL6A2,COL6A3
Role of Osteoblasts in Rheumatoid	2,83E00	5,69E-02	-1,508	ACVR2A,ALPL,CTSH,CXCL12,DKK1,DLX5,IL1B,SFRP4,STAT6,VEGFC,WNT5A,WNT5B
RHOGDI Signaling	2,8E00	5,63E-02	0,816	ACTG2,CDH13,CDH6,GNG2,GNG4,ITGA5,ITGB3,MYH14,MYL9,MYO18A,PAK3,RAC2
O-linked glycosylation	2,78E00	7,48E-02	-2,828	ADAMTS1,ADAMTS2,ADAMTS9,ADAMTSL1,B3GNT9,GALNT5,GALNT6,ST3GAL1
ILK Signaling	2,67E00	5,73E-02	-0,707	ACTG2,DSP,FLNC,FN1,ITGB3,KRT18,MYH14,MYL9,MYO18A,RAC2,VEGFC
TEC Kinase Signaling	2,61E00	5,64E-02	0,378	ACTG2,GNG2,GNG4,ITGA5,ITGB3,LYN,PAK3,RAC2,STAT6,TLR4,VAV3
Semaphorin Neuronal Repulsive Si	2,59E00	6,38E-02	-0,333	BCAN,CRMP1,CSPG4,ITGA5,ITGB3,MYL9,NRP1,PAK3,SEMA6D
Regulation of Insulin-like Growth Fa	2,58E00	6,96E-02	-0,707	C4A/C4B,FN1,IGF2,IGFBP4,IGFBP5,PAPPA,STC2,VWA1

IL-15 Production	2,58E00	6,96E-02	-0,707	AXL,ERBB4,LYN,NTRK2,RET,ROR1,ROR2,TEK
Acute Phase Response Signaling	2,54E00	5,85E-02	-1,633	A2M,C4A/C4B,FN1,HMOX1,IL1B,IL1R1,SERPINA3,SERPINE1,SERPINF1,VWF
Neutrophil Extracellular Trap Signali	2,53E00	4,51E-02	1,500	CASP10,CASQ1,COL12A1,COL1A2,COL25A1,COL3A1,COL5A1,COL5A2,COL6A2,COL6A3,GSDMD,IL1B,LYN,MT-ND3,RAC2,TLR4
Dilated Cardiomyopathy Signaling F	2,48E00	6,16E-02	-0,447	ACTG2,CACNA1C,CACNG7,MYH14,MYL9,MYO18A,PDE3A,RYR2,UTRN
Actin Cytoskeleton Signaling	2,48E00	5,15E-02	0,000	ACTG2,ARHGAP24,FGF5,FN1,ITGA5,ITGB3,MYH14,MYL9,MYO18A,PAK3,RAC2,VAV3
Formation of Fibrin Clot (Clotting Ca	2,4E00	1,25E-01	-2,000	A2M,F3,TFPI,VWF
Hepatic Fibrosis Signaling Pathway	2,38E00	4,23E-02	-3,500	ACVR2A,CACNA1C,CACNG7,CNR1,COL1A2,COL3A1,IL1B,IL1R1,ITGA5,ITGB3,MYL9,RAC2,SERPINE1,TLR4,VEGFC,WNT5A,WNT5B
GPVI-mediated activation cascade	2,3E00	1,18E-01	0,000	COL1A2,LYN,RAC2,VAV3
IL-4 Signaling	2,29E00	5,41E-02	-2,530	COL12A1,COL1A2,COL25A1,COL3A1,COL5A1,COL5A2,COL6A2,COL6A3,STAT6,TGM2
Beta-catenin independent WNT sig	2,29E00	6,86E-02	-0,378	GNG2,GNG4,RAC2,ROR1,ROR2,WNT5A,WNT5B
Signaling by VEGF	2,18E00	6,54E-02	-1,134	AXL,CYBA,ITGB3,NRP1,PAK3,VAV3,VEGFC
Activin Inhibin Signaling Pathway	2,14E00	5,13E-02	-1,265	ACVR2A,COL1A2,COL3A1,GATA2,IL1B,IL1R1,INHBA,PMEPA1,SERPINE1,TLR4

Role of Tissue Factor in Cancer	2,14E00	5,13E-02	-1,265	ACVR2A,F3,FGF5,FLNC,IL1B,ITGB3,LYN,PLAUR,TFPI,VEGFC
Cell surface interactions at the vasc	2,05E00	5,67E-02	-2,121	COL1A2,FN1,ITGA5,ITGB3,LYN,SDC4,SLC16A3,TEK
GPER1 signaling	1,9E00	9,09E-02	0,000	FN1,GNG2,GNG4,ITGA5
TR/RXR Activation	1,9E00	5,79E-02	-2,646	COL6A3,ITGA5,ITGB3,RAB3B,SLC16A3,THRB,VEGFC
Agrin Interactions at Neuromuscula	1,89E00	7,35E-02	0,000	ACTG2,ERBB4,PAK3,RAC2,UTRN
Post-translational protein phosphory	1,78E00	6,06E-02	0,000	C4A/C4B,FN1,IGFBP4,IGFBP5,STC2,VWA1
Oxytocin in Brain Signaling Pathway	1,78E00	4,76E-02	1,000	CACNA1C,CACNG7,GNG2,GNG4,IL1B,NLRP1,OXTR,PTGS1,TLR4
Transcriptional Regulatory Network	1,72E00	4,94E-02	-0,707	ACVR2A,IGF2,INHBA,RFX4,TBX3,WNT5A,WNT5B,ZIC3
Serotonin Receptor Signaling	1,68E00	3,63E-02	-1,500	BDNF,CACNA1C,CACNG7,GNG2,GNG4,HMOX1,IL1B,ITGB3,LYN,PAK3,PLAUR,PTGS1,RAC2,RYR2,TGM2,VWF
ID1 Signaling Pathway	1,68E00	4,57E-02	-0,333	ACVR2A,CHRFAM7A,ETS1,FN1,IGF2,LYN,TFAP2A,TGM2,VEGFC
Regulation of Actin-based Motility by	1,65E00	5,66E-02	-0,447	ACTG2,ITGA5,ITGB3,MYL9,PAK3,RAC2
Signaling by MET	1,63E00	6,33E-02	-2,236	COL1A2,COL3A1,COL5A1,COL5A2,FN1
IL-17A Signaling in Fibroblasts	1,59E00	6,17E-02	-2,236	COL1A2,COL3A1,CXCL12,FN1,IL1B
ABRA Signaling Pathway	1,51E00	5,88E-02	-2,236	ABLIM3,ACTG2,MYL9,SMTN,VEGFC
HEY1 Signaling Pathway	1,5E00	4,79E-02	0,378	ACVR2A,ERBB4,GATA2,HEY1,MFAP5,NR2F2,VEGFC
Pyroptosis Signaling Pathway	1,47E00	5,75E-02	-2,236	GSDMD,IL1B,IL1R1,NLRP1,TLR4

LPS/IL-1 Mediated Inhibition of RXF	1,45E00	4,17E-02	-1,000	ALDH1A2,ALDH1L2,ALDH3A1,IL1B,IL1R1,MGST1,PAPSS2,SOD3,TLR4
L1CAM interactions	1,44E00	5,04E-02	0,000	ANK3,CD24,DNM3,ITGA5,ITGB3,NRP1
Autism Signaling Pathway	1,41E00	3,78E-02	-2,111	ALDH1A2,ALDH1L2,ALDH3A1,BDNF,CACNA1C,CACNG7,IGF2,IL1B,NTRK2,WNT5A,WNT5B
EPH-Ephrin signaling	1,38E00	5,43E-02	1,342	LYN,MYH14,MYL9,PAK3,VAV3
Fcγ Receptor-mediated Phagocytos	1,38E00	5,43E-02	0,447	ACTG2,HMOX1,LYN,RAC2,VAV3
Phagosome Formation	1,36E00	3,12E-02	-2,236	APBB1IP,CHRM3,CNR1,COLEC12,FN1,GPR161,GPRC5A,HMOX1,ITGA5,ITGB3,LYN,MYH14,MYL9,MYO18A,OXTR,PAK3,RAC2,RGR,TLR4,VAV3
Signaling by Rho Family GTPases	1,35E00	3,83E-02	-0,816	ACTG2,CDH13,CDH6,GNG2,GNG4,ITGA5,ITGB3,MYL9,PAK3,RAC2
Role of Chondrocytes in Rheumatoi	1,32E00	4,72E-02	-2,449	CXCL12,FN1,IL1B,IL1R1,ITGA5,VEGFC
Human Embryonic Stem Cell Plurip	1,31E00	4,1E-02	-0,707	ACVR2A,BDNF,INHBA,NTRK2,TBX3,WNT5A,WNT5B,ZIC3
Cardiac conduction	1,3E00	4,65E-02	-2,000	ATP1A2,CACNA1C,CACNG7,CASQ1,MME,RYR2

Supplementary Table 20: Ingenuity pathway analysis of DEGs in Butyrate vs Control condition (p<0.05, z-score>|2|)

Ingenuity Canonical Pathways	-log(p-value)	Ratio	z-score	Molecules
Pathogen Induced Cytokine Storm Signaling Pathway	7,83E00	8,88E-02	-4,041	CDH1,CGAS,CKLF,COL12A1,COL1A2,COL25A1,COL3A1,COL5A2,COL6A2,COL6A3,CXCL12,FTH1,HLA-DMB,HLA-DPA1,HLA-DPB1,HLA-DQB2,HLA-DRB1,HLA-DRB5,IL1B,IL1R1,PRDM1,RYR2,SRGN,STING1,TLR4,TNFRSF1B,VEGFC
Elastic fibre formation	7,08E00	2,33E-01	-2,530	EMILIN1,EMILIN2,FBN1,FBN2,FN1,ITGA5,ITGB3,LOXL2,MFAP4,MFAP5
Assembly of collagen fibrils and other multimeric structures	5,72E00	1,69E-01	-1,897	COL12A1,COL14A1,COL1A2,COL3A1,COL5A2,COL6A2,COL6A3,LOXL2,PCOLCE,TLL2
Extracellular matrix organization	5,64E00	1,24E-01	-2,496	BCAN,BGN,COL1A2,COL3A1,COL5A2,COL6A2,COL6A3,DMD,FN1,ITGA5,ITGB3,SERPINE1,TNXB
Collagen biosynthesis and modifying enzymes	5,2E00	1,49E-01	-1,265	COL12A1,COL14A1,COL1A2,COL25A1,COL3A1,COL5A2,COL6A2,COL6A3,PCOLCE,TLL2
Integrin cell surface interactions	5,2E00	1,34E-01	-3,317	CDH1,COL1A2,COL3A1,COL5A2,COL6A2,COL6A3,FBN1,FN1,ITGA5,ITGB3,VWF
Collagen chain trimerization	4,92E00	1,82E-01	-1,414	COL12A1,COL14A1,COL1A2,COL25A1,COL3A1,COL5A2,COL6A2,COL6A3
Degradation of the extracellular matrix	4,75E00	1,5E-01	-1,667	A2M,ADAMTS1,ADAMTS9,BCAN,CDH1,FBN1,FBN2,FN1,TLL2

Osteoarthritis Pathway	4,36E00	7,73E-02	-2,324	ALPL,CASP4,CASQ1,CCN4,DKK1,DLX5,EPAS1,FN1,GREM1,IL1B,IL1R1,ITGA5,ITGB3,SDC4,TLR4,TNFRSF1B,VEGFC
Syndecan interactions	4,33E00	2,22E-01	-2,449	COL1A2,COL3A1,COL5A2,FN1,ITGB3,SDC4
Pulmonary Fibrosis Idiopathic Signaling Pathway	4,33E00	6,71E-02	-1,789	ADAMTS1,AXL,CCN4,CDH1,COL12A1,COL1A2,COL25A1,COL3A1,COL5A2,COL6A2,COL6A3,CXCL12,EPHB3,FN1,IL1B,PRKN,SERPINE1,STAT6,WNT11,WNT5A,WNT5B
IL-4 Signaling	4,15E00	8,11E-02	-3,357	COL12A1,COL1A2,COL25A1,COL3A1,COL5A2,COL6A2,COL6A3,HLA-DMB,HLA-DPA1,HLA-DPB1,HLA-DQB2,HLA-DRB1,HLA-DRB5,STAT6,TGM2
Collagen degradation	4,13E00	1,43E-01	-1,414	COL12A1,COL14A1,COL1A2,COL25A1,COL3A1,COL5A2,COL6A2,COL6A3
Response to elevated platelet cytosolic Ca2+	4,13E00	9,6E-02	-2,887	A2M,ECM1,FN1,IGF2,ITGB3,MMRN1,SERPINA3,SERPINE1,SRGN,TOR4A,VEGFC,VWF
Role of Osteoclasts in Rheumatoid Arthritis Signaling Pathway	4,08E00	6,83E-02	-2,065	CAMK4,CDH1,COL12A1,COL1A2,COL25A1,COL3A1,COL5A2,COL6A2,COL6A3,IL1B,IL1R1,ITGA5,ITGB3,RAC2,SFRP4,SHC2,TEC,TLR4,TNFRSF1B

IL-10 Signaling	3,57E00	8,39E-02	2,309	CCN4,HLA-DMB,HLA-DPA1,HLA-DPB1,HLA-DQB2,HLA-DRB1,HLA-DRB5,HMOX1,IL1B,IL1R1,PRDM1,TLR4
Neuroinflammation Signaling Pathway	3,41E00	6,25E-02	-2,309	CXCL12,GABRQ,GRIN2B,HLA-DMB,HLA-DPA1,HLA-DPB1,HLA-DQB2,HLA-DRB1,HLA-DRB5,HMOX1,IL1B,IL1R1,NFATC4,PSEN2,RAC2,SLC1A2,TIRAP,TLR4
Cell junction organization	3,36E00	0.1	-3,000	CADM2,CDH1,CDH13,CDH6,CLDN11,FLNC,PARVB,SDK1,SDK2
Coagulation System	2,95E00	1,56E-01	1,342	A2M,PLAUR,SERPINE1,TFPI,VWF
O-linked glycosylation	2,82E00	8,41E-02	-3,000	ADAMTS1,ADAMTS6,ADAMTS9,ADAMTSL1,ADAMTSL3,B3GNT9,GALNT5,MUC16,ST3GAL1
Calcium-induced T Lymphocyte Apoptosis	2,73E00	0.1	-2,000	ATP2A1,HLA-DMB,HLA-DPA1,HLA-DPB1,HLA-DQB2,HLA-DRB1,HLA-DRB5
Dendritic Cell Maturation	2,51E00	6,35E-02	-3,464	COL1A2,COL3A1,HLA-DMB,HLA-DPA1,HLA-DPB1,HLA-DQB2,HLA-DRB1,HLA-DRB5,IL1B,LTBR,TLR4,TNFRSF1B
Macrophage Alternative Activation Signaling Pathway	2,47E00	6,59E-02	-2,714	ADORA2A,EPAS1,HLA-DMB,HLA-DPA1,HLA-DPB1,HLA-DQB2,HLA-DRB1,HLA-DRB5,IL1B,STAT6,TLR4

Tumor Microenvironment Pathway	2,45E00	6,55E-02	-3,162	COL1A2,COL3A1,CSPG4,CXCL12,FGF5,FN1,IGF2,IL1B,ITGA5,ITGB3,VEGFC
WNT ligand biogenesis and trafficking	2,42E00	1,54E-01	-1,000	WLS,WNT11,WNT5A,WNT5B
Wound Healing Signaling Pathway	2,41E00	5,91E-02	-2,496	COL12A1,COL1A2,COL25A1,COL3A1,COL5A2,COL6A2,COL6A3,FN1,IL1B,IL1R1,SHC2,TNFRSF1B,VEGFC
Gαs Signaling	2,41E00	7,32E-02	-1,890	ADCY9,ADORA2A,CHRM3,CNR1,GNAO1,GNG2,GNG4,RYR2,VIPR1
Beta-catenin independent WNT signaling	2,38E00	7,84E-02	-0,707	GNAO1,GNG2,GNG4,RAC2,ROR2,WNT11,WNT5A,WNT5B
Multiple Sclerosis Signaling Pathway	2,35E00	6,06E-02	-2,887	GRIN2B,HLA-DMB,HLA-DPA1,HLA-DPB1,HLA-DQB2,HLA-DRB1,HLA-DRB5,IL1B,IL7R,MASP1,PARP8,TLR4
GPER1 signaling	2,32E00	1,14E-01	-0,447	ADCY9,FN1,GNG2,GNG4,ITGA5
Binding and Uptake of Ligands by Scavenger Receptors	2,28E00	1,11E-01	-1,342	COL1A2,COL3A1,COLEC12,FTH1,MASP1
Semaphorin interactions	2,27E00	9,38E-02	-0,816	CRMP1,MYL9,NRP1,PAK3,SEMA6D,SEMA7A
Hepatic Fibrosis Signaling Pathway	2,18E00	4,73E-02	-3,771	CACNG7,CNR1,COL1A2,COL3A1,FTH1,IL1B,IL1R1,ITGA5,ITGB3,MYL9,RAC2,SERPINE1,TIRAP,TLR4,TNFRSF1B,VEGFC,WNT11,WNT5A,WNT5B
Interferon gamma signaling	2,17E00	7,95E-02	-2,646	HLA-DPA1,HLA-DPB1,HLA-DQB2,HLA-DRB1,HLA-DRB5,MT2A,TRIM38
G alpha (z) signalling events	2,16E00	1,04E-01	0,447	ADCY9,GNG2,GNG4,RGS16,RGS4

Th1 Pathway	2,16E00	7,21E-02	-2,121	HLA-DMB,HLA-DPA1,HLA-DPB1,HLA-DQB2,HLA-DRB1,HLA-DRB5,NFATC4,PSEN2
RHOGDI Signaling	2,11E00	5,63E-02	1,633	CDH1,CDH13,CDH6,GNAO1,GNG2,GNG4,ITGA5,ITGB3,MYL9,MYO18A,PAK3,RAC2
EPH-Ephrin signaling	2,07E00	7,61E-02	-0,378	ARHGEF28,CLTCL1,EPHB3,GRIN2B,MYL9,PAK3,PSEN2
Gαi Signaling	2,07E00	6,47E-02	0,000	ADCY9,CNR1,DRD4,GNAO1,GNG2,GNG4,HTR1F,RGS4,SHC2
Neutrophil Extracellular Trap Signaling Pathway	2,06E00	4,79E-02	1,213	CASP4,CASQ1,COL12A1,COL1A2,COL25A1,COL3A1,COL5A2,COL6A2,COL6A3,IL1B,MT-ATP6,MT-ND2,MT-ND3,MT-ND5,RAC2,TIRAP,TLR4
Costimulation by the CD28 family	2,05E00	8,45E-02	-1,633	HLA-DPA1,HLA-DPB1,HLA-DQB2,HLA-DRB1,HLA-DRB5,PAK3
Granzyme A Signaling	2,05E00	8,45E-02	-0,816	FN1,H1-0,IL1B,MT-ND2,MT-ND3,MT-ND5
Opioid Signaling Pathway	2,04E00	5,15E-02	-0,832	ADCY9,ARRB1,CACNG7,CAMK4,CLTCL1,GNAO1,GNG2,GNG4,GRIN2B,RAC2,RGS16,RGS4,RGS8,RYR2
Semaphorin Neuronal Repulsive Signaling Pathway	2,03E00	6,38E-02	-0,333	BCAN,CRMP1,CSPG4,ITGA5,ITGB3,MYL9,NRP1,PAK3,SEMA6D
Ephrin B Signaling	2,02E00	8,33E-02	-1,000	CXCL12,EPHB3,GNAO1,GNG2,GNG4,RAC2

Xenobiotic Metabolism AHR Signaling Pathway	2,02E00	8,33E-02	-1,633	AHRR,ALDH1L2,ALDH3A1,CYP1B1,IL1B,MGST1
ILK Signaling	2,02E00	5,73E-02	-1,414	CDH1,DSP,FLNC,FN1,ITGB3,KRT18,MYL9,MYO18A,PARVB,RAC2,VEGFC
MHC class II antigen presentation	2	6,78E-02	-1,414	DNM3,HLA-DMB,HLA-DPA1,HLA-DPB1,HLA-DQB2,HLA-DRB1,HLA-DRB5,TUBB4A
GP6 Signaling Pathway	2	6,78E-02	-2,121	COL12A1,COL1A2,COL25A1,COL3A1,COL5A2,COL6A2,COL6A3,ITGB3
Activin Inhibin Signaling Pathway	1,98E00	5,64E-02	-1,508	CDH1,COL1A2,COL3A1,GATA2,IL1B,IL1R1,INHBA,PMEPA1,SERPINE1,TLR4,TNFRSF1B
Acute Phase Response Signaling	1,94E00	5,85E-02	-1,890	A2M,FN1,HMOX1,IL1B,IL1R1,SERPINA3,SERPINE1,SERPINF1,TNFRSF1B,VWF
TR/RXR Activation	1,94E00	6,61E-02	-2,828	ATP2A1,CAMK4,COL6A3,ITGA5,ITGB3,RAB3B,SLC16A3,VEGFC
Signaling by NOTCH1	1,91E00	7,89E-02	-0,816	ARRB1,DNER,DTX4,NBEA,PSEN2,TLE3
Ephrin Receptor Signaling	1,88E00	5,47E-02	-0,707	CXCL12,EPHB3,GNAO1,GNG2,GNG4,GRIN2B,ITGA5,ITGB3,PAK3,RAC2,VEGFC
PD-1, PD-L1 cancer immunotherapy pathway	1,88E00	0.07	1,000	HLA-DMB,HLA-DPA1,HLA-DPB1,HLA-DQB2,HLA-DRB1,HLA-DRB5,TNFRSF1B
Glycosaminoglycan metabolism	1,84E00	7,59E-02	-1,633	B3GAT1,BCAN,BGN,CSPG4,SDC4,ST3GAL1
Crosstalk between Dendritic Cells and Natural Killer Cells	1,84E00	7,59E-02	-2,449	HLA-DRB1,HLA-DRB5,LTBR,MICB,TLR4,TNFRSF1B

Calcium Signaling	1,83E00	5,37E-02	-1,000	ATP2A1,CACNG7,CAMK4,CASQ1,CHRFAM7A,GRIA4,GRIN2B,MYL9,MYO18A,NFATC4,RYR2
Th2 Pathway	1,82E00	6,3E-02	-2,121	HLA-DMB,HLA-DPA1,HLA-DPB1,HLA-DQB2,HLA-DRB1,HLA-DRB5,PSEN2,STAT6
Signaling by PDGF	1,82E00	8,62E-02	-2,236	COL3A1,COL5A2,COL6A2,COL6A3,STAT6
Macrophage Classical Activation Signaling Pathway	1,81E00	5,88E-02	-2,333	HLA-DMB,HLA-DPA1,HLA-DPB1,HLA-DQB2,HLA-DRB1,HLA-DRB5,IL1B,STAT6,TLR4
Role of Osteoblasts in Rheumatoid Arthritis Signaling Pathway	1,74E00	5,21E-02	-1,508	ALPL,CXCL12,DKK1,DLX5,IL1B,SFRP4,STAT6,VEGFC,WNT11,WNT5A,WNT5B
Signaling by VEGF	1,73E00	6,54E-02	-1,134	AXL,CYBA,ITGB3,NRP1,PAK3,SHC2,VEGFC
Neurovascular Coupling Signaling Pathway	1,73E00	5,19E-02	-2,111	ADORA2A,CACNG7,CHRM3,GABRQ,GRIA4,GRIN2B,KCNMB3,KCNN4,PTGS1,RYR2,SLC1A2
Regulation of the Epithelial Mesenchymal Transition in Development Pathway	1,72E00	7,14E-02	0,000	CDH1,PSEN2,S100A4,WNT11,WNT5A,WNT5B
G alpha (i) signalling events	1,68E00	4,92E-02	-0,577	ADCY9,CNR1,CXCL12,DRD4,GNG2,GNG4,HTR1F,RGR,RGS16,RGS4,RGS8,TAS2R4
Transcriptional Regulatory Network in Embryonic Stem Cells	1,67E00	5,56E-02	-0,333	IGF2,INHBA,NOG,RFX4,TBX3,WNT11,WNT5A,WNT5B,ZIC3
Gαq Signaling	1,67E00	5,56E-02	-0,816	CHRM3,GNAO1,GNG2,GNG4,HMOX1,NFATC4,RAC2,RGS16,RGS4

G alpha (s) signalling events	1,66E00	5,88E-02	-0,707	ADCY9,ADM,ADORA2A,ARRB1,GNG2,GNG4,PDE3A,VIPR1
CTLA4 Signaling in Cytotoxic T Lymphocytes	1,65E00	5,26E-02	1,897	CLTCL1,HLA-DMB,HLA-DPA1,HLA-DPB1,HLA-DQB2,HLA-DRB1,HLA-DRB5,HMOX1,NFATC4,RAC2
Pyroptosis Signaling Pathway	1,65E00	6,9E-02	-2,449	CASP4,IL1B,IL1R1,NEK7,TLR4,TNFRSF1B
GABAergic Receptor Signaling Pathway (Enhanced)	1,65E00	5,84E-02	0,707	ADCY9,CACNG7,GABRQ,GNAO1,GNG2,GNG4,GRIN2B,NRXN3
MSP-RON Signaling in Macrophages Pathway	1,64E00	6,25E-02	2,646	HLA-DMB,HLA-DPA1,HLA-DPB1,HLA-DQB2,HLA-DRB1,HLA-DRB5,TLR4
TEC Kinase Signaling	1,58E00	5,13E-02	-0,447	GNAO1,GNG2,GNG4,ITGA5,ITGB3,PAK3,RAC2,STAT6,TEC,TLR4
Regulation of Insulin-like Growth Factor (IGF) transport and uptake by IGFBPs	1,58E00	6,09E-02	-1,890	FBN1,FN1,IGF2,IGFBP4,IGFBP5,PAPPA,STC2
Cell surface interactions at the vascular wall	1,58E00	5,67E-02	-2,828	COL1A2,FN1,ITGA5,ITGB3,PSG4,SDC4,SLC16A3,TEK
Cytosolic sensors of pathogen-associated DNA	1,55E00	8,7E-02	-2,000	CGAS,DTX4,STAT6,STING1
cAMP-mediated signaling	1,54E00	4,85E-02	-2,714	ADCY9,ADORA2A,CAMK4,CHRM3,CNR1,DRD4,GNAO1,HTR1F,PDE3A,RGS4,VIPR1
Colorectal Cancer Metastasis Signaling	1,54E00	4,69E-02	-0,333	ADCY9,ARRB1,CDH1,GNAO1,GNG2,GNG4,RAC2,TLR4,VEGFC,WNT11,WNT5A,WNT5B

PKCθ Signaling in T Lymphocytes	1,53E00	5,26E-02	-2,236	CACNG7,HLA-DMB,HLA-DPA1,HLA-DPB1,HLA-DQB2,HLA-DRB1,HLA-DRB5,NFATC4,RAC2
ICOS-ICOSL Signaling in T Helper Cells	1,53E00	5,93E-02	-2,000	HLA-DMB,HLA-DPA1,HLA-DPB1,HLA-DQB2,HLA-DRB1,HLA-DRB5,NFATC4
TCR signaling	1,51E00	5,88E-02	-1,890	CD101,HLA-DPA1,HLA-DPB1,HLA-DQB2,HLA-DRB1,HLA-DRB5,PAK3
Serotonin Receptor Signaling	1,51E00	4,08E-02	-1,414	ADCY9,ARRB1,CACNG7,CDH1,GNAO1,GNG2,GNG4,HMOX1,IL1B,ITGB3,KCNN4,PAK3,PLAUR,PTGS1,RAC2,RYR2,TGM2,VWF
Dilated Cardiomyopathy Signaling Pathway	1,5E00	5,48E-02	-0,378	ADCY9,CACNG7,CAMK4,DMD,MYL9,MYO18A,PDE3A,RYR2
Endocannabinoid Neuronal Synapse Pathway	1,5E00	5,48E-02	0,378	ADCY9,CACNG7,CNR1,GNAO1,GNG2,GNG4,GRIA4,GRIN2B
Class B/2 (Secretin family receptors)	1,49E00	6,32E-02	0,000	ADM,GNG2,GNG4,VIPR1,WNT11,WNT5A
IL-8 Signaling	1,46E00	4,88E-02	-1,890	CDH1,GNAO1,GNG2,GNG4,HMOX1,ITGB3,MYL9,RAC2,TEK,VEGFC
Role of NFAT in Regulation of the Immune Response	1,43E00	4,83E-02	-2,236	GNAO1,GNG2,GNG4,HLA-DMB,HLA-DPA1,HLA-DPB1,HLA-DQB2,HLA-DRB1,HLA-DRB5,NFATC4
Apoptotic execution phase	1,38E00	7,69E-02	-1,000	CDH1,DSP,H1-0,PKP1

Role of Chondrocytes in Rheumatoid Arthritis Signaling Pathway	1,38E00	5,51E-02	-2,646	CXCL12,FN1,IL1B,IL1R1,ITGA5,TNFRSF1B,VEGFC
Keratinization	1,37E00	6,58E-02	-1,342	DSP,FLG,KRT18,KRT19,PKP1
Synaptogenesis Signaling Pathway	1,35E00	4,28E-02	0,277	ADCY9,CDH1,CDH13,CDH6,CPLX1,CPLX2,EPHB3,GRIA4,GRIN2B,NRXN3,NTRK2,SHC2,SYT14
Acetylcholine Receptor Signaling Pathway	1,34E00	4,84E-02	-1,000	ADCY9,CACNG7,CASP4,CASQ1,CHRFAM7A,CHRM3,GNAO1,HMOX1,PSEN2
Immunogenic Cell Death Signaling Pathway	1,33E00	6,41E-02	-2,236	CGAS,IL1B,STING1,TLR4,TNFRSF1B
G-Protein Coupled Receptor Signaling	1,3E00	3,6E-02	-1,633	ADCY9,ADORA2A,ARRB1,CAMK4,CHRM3,CNR1,DRD4,GNAO1,GNG2,GNG4,GPRC5A,HTR1F,KCNN4,MYL9,NFATC4,OXTR,PAK3,PDE3A,RAC2,RGR,RGS16,RGS4,SHC2,VIPR1

Supplementary Table 21: DEGs in Acetate + IL6 vs IL6 conditions (log2FoldChange ≤ -0.59 or ≥ +0.59, adjusted p-value< 0.1)

Gene symbol	Gene name	log2FoldChange	padj
DCLRE1C	DNA cross-link repair 1C	0.631783188	0.04249314
ARMC9	armadillo repeat containing 9	-0.603777855	0.016973288
KIN	Kin17 DNA and RNA binding protein	-0.705343929	0.067327023
MX2	MX dynamin like GTPase 2	-1.906496134	0.037795504
CYP24A1	cytochrome P450 family 24 subfamily A member 1	-15.95015813	0.00000275
PARP8	poly(ADP-ribose) polymerase family member 8	-17.76659932	0.000000309
PTGER4	prostaglandin E receptor 4	-17.87783646	8.67E-17
IKBKGP1	inhibitor of nuclear factor kappa B kinase subunit gamma pseudogene 1	-19.95564291	0.0000856
PTPRCAP	protein tyrosine phosphatase receptor type C associated protein	-21.35538005	0.000000797
GARIN1A	golgi associated RAB2 interactor 1A	-21.42636879	1.91E-15
PWAR5	Prader Willi/Angelman region RNA 5	-26.66021081	1.32E-61
ADAMTS1	ADAM metallopeptidase with thrombospondin type 1 motif 1	-27.30775264	4.29E-10
CSF3	colony stimulating factor 3	-30	2.76E-28

Supplementary Table 22: DEGs in Propionate +IL6 vs IL6 conditions (log2FoldChange ≤ -0.59 or ≥ +0.59, adjusted p-value< 0.1)

Gene symbol	Gene name	log2FoldChange	padj
LOC102723360	uncharacterized LOC102723360	1.53675299	0.022925
C2orf72	chromosome 2 open reading frame 72	-0.687254212	0.050399
KALRN	kalirin RhoGEF kinase	-1.531154881	0.020165
CXCL10	C-X-C motif chemokine ligand 10	-2.915453464	0.021514

Supplementary Table 23: DEGs in Butyrate+IL6 vs IL6 conditions (log2FoldChange ≤ -0.59 or ≥ +0.59, adjusted p-value< 0.1)

Gene symbol	Gene name	log2FoldChange	padj
LOC653653	adaptor related protein complex 1 subunit	3.754707562	0.020097
ZNF468	zinc finger protein 468	-0.595629506	0.002274
GTF2IRD2	GTF2I repeat domain containing 2	-0.84627577	0.020097
SMG1P4	SMG1 pseudogene 4	-7.299758852	0.051381
